# *bHLH92* from sheepgrass acts as a negative regulator of anthocyanin/proanthocyandin accumulation and influences seed dormancy

**DOI:** 10.1093/jxb/ery335

**Published:** 2018-09-18

**Authors:** Pincang Zhao, Xiaoxia Li, Junting Jia, Guangxiao Yuan, Shuangyan Chen, Dongmei Qi, Liqin Cheng, Gongshe Liu

**Affiliations:** 1 Key Laboratory of Plant Resources, Institute of Botany, Chinese Academy of Sciences, Beijing, China; 2 College of management science and engineering, Hebei University of Economics and Business, China

**Keywords:** *LcbHLH92*, *Leymus chinensis*, proanthocyanidins, seed color, seed dormancy

## Abstract

Sheepgrass (*Leymus chinensis*) is an important native forage grass, and it is widely grown in north China. Differential dormancy exists in sheepgrass germplasms with different seed colors. To decipher and find potential genes underlying this phenomenon, we compared the transcript profiles of yellow seeds with weak dormancy and brown seeds with strong dormancy. We identified a transcription factor gene *LcbHLH92* which is negatively correlated with those of anthocyanin/proanthocyanidin-specific pathway genes, *anthocyanidin synthase* (*ANS*) and *anthocyanidin reductase* (*ANR*). *LcbHLH92* had two transcripts, *LcbHLH92a* and *LcbHLH92b*, and their expression could be induced by abscisic acid, cold, and NaCl. Overexpression of *LcbHLH92a* or *LcbHLH92b* in Arabidopsis significantly inhibited the transcript levels of *dihydroflavonol reductase* (*DFR*) and *ANS* genes in leaves and seeds, which resulted in a decrease in anthocyanins and proanthocyanidins, respectively. Importantly, transgenic Arabidopsis seeds with a yellow color showed a higher germination rate than did the wild-type with a brown seed color. Moreover, LcbHLH92a and LcbHLH92b repressed the transcription of *Transparent Testa8*, *ANS*, *DFR*, and *ANR*, possibly by elevating the transcript levels of jasmonate-ZIM domain proteins through binding to their promoters. Together, our results demonstrate that LcbHLH92a and LcbHLH92b are negative regulators of the anthocyanin/proanthocyanidin pathway and influence seed dormancy.

## Teaser

*LcbHLH92*, a pleiotropic gene from sheepgrass, negatively regulates anthocyanins/proanthocyandins and reduces seed dormancy in transgenic Arabidopsis.

## Introduction

Seed dormancy or germination is a combined result of co-action between embryo growth potential and the surrounding tissue restraints (Koornneef *et al.*, 2002). Plant seeds have evolved diverse dormancy mechanisms to adapt to their living habitats. In *Arabidopsis thaliana*, the reduced dormancy of *transparent testa* (*tt*) mutants indicates the contribution of the testa structure and pigments to seed dormancy (Debeaujon and Koornneef, 2000; Debeaujon *et al.*, 2000). Seed coat pigments are usually flavonoid compounds, proanthocyanidins and flavonol glycosides in *A. thaliana*, or phlobaphenes in cereal pericarp (Finkelstein *et al.*, 2008). The testa of *tt* mutant seeds appears thinner and has superior permeability; thus it is easily penetrated by exogenous water, oxygen, and endogenous abscisic acid (ABA) (Debeaujon *et al.*, 2000). In Arabidopsis, mutants of *tt8* (*AtbHLH42*) and *tt2* (*AtMYB123*) confer reduced dormancy and yellow testa to seeds by reducing proanthocyanidin accumulation and further *de novo* biogenesis of ABA in the germination stage (Debeaujon *et al.*, 2000; Nesi et al., 2000, 2001; Jia *et al.*, 2012). In addition, TT2, TRANSPARENT TESTA GLABRA1 (TTG1), and TT8 form a ternary protein complex [a type of MYB–bHLH–WD40 (MBW) protein complex] that regulates *dihydroflavonol reductase* (*DFR*) and *BANYULS* (*BAN*) expression in immature seeds to determine the accumulation of proanthocyanidins (Nesi *et al.*, 2000, 2001; Baudry *et al.*, 2004). JAZs also act as negative regulators of the flavonoid pathway through interactions with MBW complexes (Qi *et al.*, 2011, 2014; Xie *et al.*, 2016).

The differentiation of seed dormancy has been associated with seed morphologies such as pericarp (or testa) color, as also revealed in monocots in several previous studies. In barley mutants, deficiencies in total phenols and proanthocyanidins in grains result in a shorter dormancy and greater water absorption ability (Wu *et al.*, 1996). In wheat (*Triticum aestivum* L.), red-grained lines show a longer dormancy than white-grained near-isogenic or mutant lines (Flintham, 2000; Warner *et al.*, 2000; Himi *et al.*, 2002, 2011; Gu *et al.*, 2011). Moreover, the *R* gene, a MYB-type transcription factor, for grain color enhances grain dormancy by increasing the sensitivity of embryos to ABA in wheat (Flintham, 2000; Warner *et al.*, 2000; Himi *et al.*, 2002; Himi and Noda, 2005). In rice, a pleiotropic gene, *SD7-1* [basic helix–loop–helix (bHLH)-type, LOC_ Os07g11020], associates seed dormancy and pericarp color by promoting the biosynthesis of ABA and pigments (Sweeney *et al.*, 2006; Gu *et al.*, 2011). Mapping of some loci that affect both seed color and dormancy in rice has revealed a very tight linkage or a single locus controlling the two characteristics (Ji *et al.*, 2006).

Sheepgrass [*Leymus chinensis* (Trin.) Tzvel] is a native perennial forage grass in China with high economic and ecological value. Sheepgrass shows high palatability and strong adaptability to drought, saline–alkali and/or low fertility soils (Chen *et al.*, 2013; Sun *et al.*, 2013). Due to these properties, sheepgrass has been widely used in natural grassland restoration and artificial grassland establishment. Sheepgrass has become the top native forage grass, with a total area of grasslands measuring 220 000 km^2^ in China and ~420 000 km^2^ in Asia (Bai *et al.*, 2010). However, the low germination rate (10–20%) with deep dormancy affects rapid and uniform seed germination in sheepgrass production and further blocks its wide application and the attainment of its expected value (Ma *et al.*, 2010*b*). Approaches such as fluctuating temperature or the application of plant hormones, removal of the hull (palea and lemma), and mechanical resistance have been used in dormancy breaking and germination (Ma *et al.*, 2010*a*; Hu *et al.*, 2012; He *et al.*, 2016). Although our understanding of seed dormancy and germination is well advanced in some model plants and important crops (Himi *et al.*, 2011; Jia *et al.*, 2012), little is known about the molecular mechanism of seed dormancy in sheepgrass, and it will be important to select genes related to germination or dormancy for molecular breeding.

In the investigation of sheepgrass seed dormancy, we found that yellow-seed germplasm (Y) showed weak dormancy, whereas brown-seed germplasm (B) showed strong dormancy. According to previous studies on gramineous plants, it is likely that seed dormancy in sheepgrass could be correlated with proanthocyanidin accumulation and controlled by unidentified regulators. In our previous studies, many stress-induced genes were identified through transcriptome sequencing of sheepgrass, and some genes were found to increase stress tolerance of transgenic plants significantly (Li *et al.*, 2013; Gao *et al.*, 2016). In the present study, we found that at least 6 out of 19 *bHLH* transcription factor genes were differentially expressed in two distinct germplasms at different seed developing stages by comparing the global transcripts. These six *bHLH* genes were also negatively correlated with *anthocyanidin synthase* (*ANS*) and *anthocyanidin reductase* (*ANR*), two key genes in the proanthocyanidin biosynthesis pathway (Xie *et al.*, 2003; Dixon *et al.*, 2005). One of them, *bHLH92*, had two transcripts, and the overexpression of its transcripts in *A. thaliana* inhibited anthocyanin accumulation in leaves and proanthocyanidins in the seed coat. Interestingly, the seeds of transgenic *A. thaliana* germinated more rapidly than those of the wild-type (WT) controls, suggesting that *LcbHLH92a* and *LcbHLH92b* promote seed germination and reduce seed dormancy. Based on quantitative real-time PCR (qRT-PCR) and ChIP analyses, we found that jasmonate-ZIM domain protein (*JAZ*) genes were significantly activated by LcbHLH92a and LcbHLH92b. Taken together, we demonstrated that LcbHLH92a and LcbHLH92b are negative regulators of the anthocyanin/proanthocyanidin pathway and can enhance seed germination in transgenic plants. The present study enriches our current understanding of the flavonoid regulatory mechanism and provides potential genes for forage and other crop quality improvement and seed dormancy.

## Materials and methods

### Sheepgrass seed germination assay and abiotic stress treatments

For the seed germination tests of different sheepgrass germplasm, seeds were germinated on filter paper at 28 °C/12 h under light, followed by 16 °C/12 h under dark in an illumination incubator. To examine abiotic stress, sheepgrass seeds (Zhongke No. 2) were planted into a plastic pot (~390 ml, 7 cm×7 cm×8 cm) containing soil nutrients (Pindstrup Substrate, Denmark) and vermiculite (130 ml/260 ml, v/v) in a greenhouse at 22–27 °C with a 16 h light/8 h dark photoperiod.

Two-week-old sheepgrass (Zhongke No. 2) seedlings were used for different abiotic stress treatments. For ABA, salt, and mannitol treatments, the plastic pots were submerged in 100 μmol l^–1^ ABA, 200 mmol l^–1^ NaCl, and 300 mmol l^–1^ mannitol, respectively. For cold treatment, seedlings were incubated at 4 °C. Plant samples were collected at 0, 1, 2, 4, 8, and 12 h after the treatments. All samples were immediately frozen in liquid nitrogen and subsequently stored at –80 °C for qRT-PCR analysis as mentioned above.

### RNA isolation, transcriptome sequencing, and qRT-PCR

Sheepgrass seeds used for transcriptome sequencing were collected at 14 d and 28 d after pollination and stored at –80 °C for RNA isolation. Samples were ground in liquid nitrogen and total RNA was extracted using a DP437 kit (TIANGEN, Beijing, China) and purified with a RP3202 kit (BioTeke, Beijing, China) according to the manufacturer’s instructions. Purified RNA was used for transcriptome sequencing employing an IlluminaHiseq™2500 platform at Novogene Company (Beijing, China).

Total RNA from spikelet, leaf, sheath, flag leaf, rhizome, root, and pollen of sheepgrass was extracted using the TRIzol Kit (TaKaRa, Dalian, China). First-strand cDNA was synthesized using the PrimeScript™ RT reagent Kit (TaKaRa) according to the manufacturer’s instructions. qRT-PCR was performed in triplicate according to the SYBR PremixExTaq™ protocol (TaKaRa) on a LightCycler480 Real-Time PCR System (Roche, Rotkreuz, Switzerland) using a 45 cycle program (95 °C for 5 s and 68 °C for 30 s). qRT-PCR data were analyzed using the 2^−ΔΔCT^ method (Livak and Schmittgen, 2001). Heatmaps were plotted using the pheatmap program (the R package offers functions for drawing clustered heatmaps stroed in CRAN: https://cran.r-project.org/) under the statistical environment R (version 3.20). All primers used in the present study are listed in Supplementary Table S5 at *JXB* online.

### Identification of differentially expressed genes and transcription factors

The differentially expressed genes (DEGs) were identified from the sequenced transcriptome of immature or matures seeds by DEseq with the criterion ‘Padj<0.05’ (Anders and Huber, 2010). To identify transcription factors that were potentially involved in the developmental regulation of sheepgrass seeds, transcription factors from *Oryza sativa* subsp. *japonica* and *A. thaliana* from the Plant Transcription Factor Database (http://planttfdb.cbi.pku.edu.cn/) were used to construct a local database. This database was then searched with the nucleotide sequences of DEGs by BLASTX at a threshold of 1e-10. The BLASTX results were extracted and counted using Perl scripts. To predict the possible functions and biological pathways of these DEGs, the genes were annotated using the following databases: the NR database, SwissProt database, Kyoto Encyclopedia of Genes and Genomes (KEGG), and Clusters of Orthologous Groups database.

### Correlation analysis of structural genes with transcription factors

A correlation analysis of transcription factors with *ANR* and *ANS* was performed at the transcript level to identify transcription factors that were possibly involved in flavonoid metabolism. Spearman and Pearson correlation analyses were performed using the FPKM (fragments per kilobase of exon per million fragments) value of each gene in R environmental language (version 3.20). Transcription factors with correlation coefficient values ≥0.7 or ≤ –0.7 and *P*<0.05 were considered to have a significant expression correlation with flavonoid pathway genes.

### Phylogenetic analysis of LcbHLH92 proteins

LcbHLH92 homologs were identified by sequence similarity by searching the NCBI database using the BLASTX program. Selected amino acid sequences were aligned using DNAMAN software (version 7.0). A phylogenetic tree was generated using the maximum likelihood (ML) method based on the JTT matrix-based model, gamma distribution (5), 1000 bootstrap replications, and complete deletion of gaps in MEGA software version 6.0 (Jones *et al.*, 1992; Tamura *et al.*, 2013). The numbers on the branches indicate the percentage of bootstrap support from 1000 replicates. The branch length represents the divergence distance.

### Transformation of *LcbHLH92* in Arabidopsis

To generate transgenic plants, the ORFs of *LcbHLH92a* and *LcbHLH92b* were inserted into the pSN1301 vector (Ge *et al.*, 2004) under the control of the *Cauliflower mosaic virus* 35S (CaMV 35S) promoter. Constructs pSN1301-*LcbHLH92a* or pSN1301-*LcbHLH92b* were introduced into *Agrobacterium* strain GV3101 using a freeze–thaw method (Raviraja and Sridhar, 2007) and further transformed into *A. thaliana* (Columbia-0) using a floral dipping method as previously described (Clough and Bent, 1998). Positive transgenic plants were selected on 30 ml of half-strength Muashige and Skoog (1/2 MS) solid medium supplemented with 50 mg l^–1^ hygromycin and further confirmed by PCR. We obtained 15 and 20 independent transgenic lines for *LcbHLH92a* and *LcbHLH92b*, respectively, and all transgenic lines showed a similar phenotype with yellow seed coat color (Supplementary Fig. S7). One representative line for each construct was selected for further study. Seeds of T_3_ transgenic *A. thaliana* were used for the germination assay, seed color observation, and quantification of anthocyanins and proanthocyanidins. *Arabidopsis thaliana* seeds were surface-sterilized with 10% NaClO for 10 min, followed by washing five times with sterile water, and then were geminated on solid MS medium at 23 °C with a light/dark period of 16 h/8 h. T_4_ transgenic seedlings were used for physiological and qRT-PCR analysis. In addition, T_3_ transgenic *A*. *thaliana* seeds were germinated directly on filter paper to calculate the germination rate.

### Subcellular localization and transcriptional activity assay

To generate the subcellular localization constructs, ORFs of *LcbHLH92a* or *LcbHLH92b* were cloned into the pCAMBIA1302 vector to express fusion proteins with green fluorescent protein (GFP). Tobacco leaves were infiltrated by *Agrobacterium tumefaciens* strain EHA105 harboring the pCAMBIA1302-LcbHLH92a or pCAMBIA1302-LcbHLH92b-GFP construct. GFP fluorescence in tobacco leaves was imaged using a Multiphoton Laser Scanning Microscope (Olympus FV1000MPE) after incubation for 2–3 d in a growth chamber. The pCAMBIA1302-GFP vector was used as a control.

To investigate the transcription activation activity qualitatively, the ORFs were cloned into the pBridge vector (Takatsu *et al.*, 2001) to obtain pBD-LcbHLH92a and pBD-LcbHLH92a, and subsequently transformed into yeast strain AH109, which contains the LacZ reporter system. Positive transformants were selected on SD medium (synthetic medium plus dextrose, Difco) lacking Trp and further confirmed by PCR. The β-galactosidase (LacZ reporter) assay was performed on sterile filter paper according to Laporte *et al.* (1999). Transformants were grown at 30 °C for 2–4 d and assayed for β-galactosidase activity. Yeast colonies were transferred directly onto sterile filter paper and grown overnight in selective medium for the liquid culture assays. Filters were assayed for β-galactosidase activity in Z-buffer containing 5-bromo-4-chloro-3-indolyl-d-galactoside. For qualitative and quantitative detection of the transcription activity, the ORFs were cloned into the pRT-BD vector system (Liao *et al.*, 2008), and the dual-luciferase reporter assay system (Promega, Madison, WI, USA) was used to detect the transcription activation activity, as previously described (Gao *et al.*, 2016).

### Extraction and quantification of anthocyanins and proanthocyanidins

Anthocyanins were extracted from fresh tissue using methanol containing 1% HCl and measured using an ultraviolet spectrophotometer (2600 UV/VIS). The formula (*A*_530-0.25_×*A*_657_) per gram fresh weight was used to quantify the relative anthocyanin content (Xie *et al.*, 2016). Extractable and non-extractable proanthocyanidin levels were determined by heating with butanol/HCl (95:5, v/v) at 550 nm as previously described (Pang *et al.*, 2007; Jiang *et al.*, 2015).

### Electrophoretic mobility shift and chromatin immunoprecipitation assays

To detect the in vitro binding activity of LcbHLH92a and LcbHLH92b to the E-box/G-box motif (CANNTG), an EMSA was performed. The recombinant proteins (pET30a-LcbHLH92a or pET30a-LcbHLH92b) induced by isopropyl-β-d-galactopyranoside (IPTG; working on 1 mmol l^–1^) at 37°C for 3 h from *Escherichia coli* BL21 (DE3) were incubated with biotin-labeled probes (BP-F/BP-R, listed in Supplementary Table S5).

The ChIP assay was performed using the EpiQuik Plant ChIP kit (Epigentek, Brooklyn, NY, USA) with an anti-FLAG antibody (TRANSGEN BIOTECH, Beijing, China) in triple biological replicates. The enrichment of the DNA fragments was measured by qRT-PCR using the SYBR PrimeScript PCR Kit (TaKaRa) on a LightCycler480 System (Roche). The relative concentration of the DNA fragments was calculated using the comparative Ct method, in which the Ct value of the first primer pair (P1) in LcbHLH92a-input and LcbHLH92b-input was set as ‘1’.

### Statistical analysis

One-way ANOVA was used to compare differences between samples. The sample variability was indicated as the SD of the mean. *P*-values <0.001 (***), <0.01 (**), or <0.05 (*) were considered significant.

### Accession numbers

Sequence data for genes in this article can be found at National Center for Biotechnology Information under the following accession numbers: *LcbHLH92* (KY000711), *LcbHLH92a* (KY000709), *LcbHLH92b* (KY000710), and transcriptome sequence (SRP130835).

## Results

### Characteristics of sheepgrass seeds

**Fig. 1.**
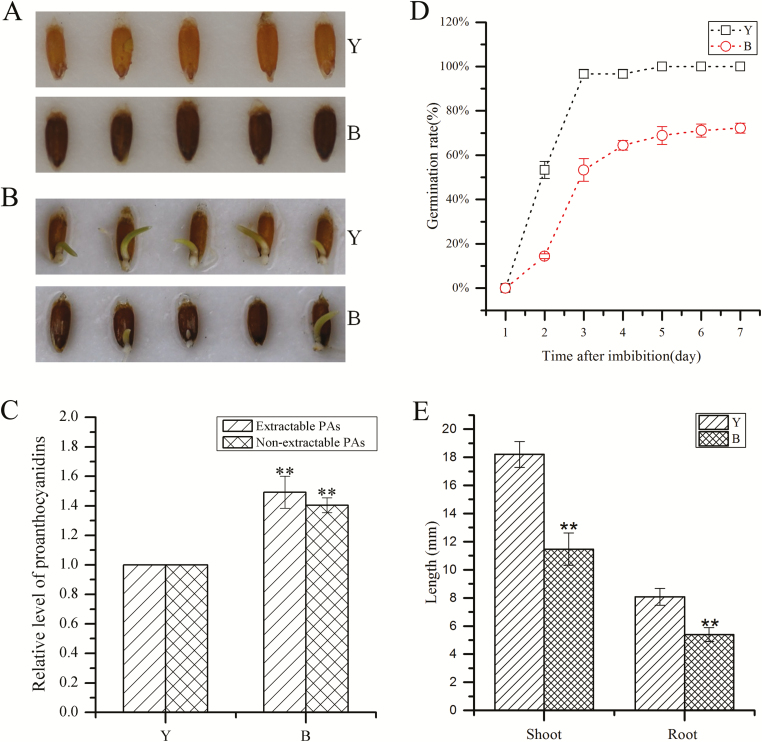
Seed morphology and germination assay of two sheepgrass germplasms. (A) Morphology of sheepgrass cultivar seeds with a yellow seed coat (Y, upper panel) and seeds with a brown seed coat (B, lower panel). (B) Germination of sheepgrass seeds 4 d after imbibition. Both seeds with a yellow seed coat (Y, upper panel) and seeds with a brown seed coat (B, lower panel) were germinated at 28 °C/12 h and 16°C/12 h under dark conditions. (C) Relative proanthocyanin content of sheepgrass germplasm with a yellow seed coat (Y) and cultivars with a brown seed coat (B). Data indicate the mean ±SD of three biological replicates (*n*=3, **P<0.01). (D) Germination rate of sheepgrass germplasms with a yellow seed coat (Y) and seeds with a brown seed coat (B). Data indicate the mean ±SD of three biological replicates. (E) Shoot and root length of two sheepgrass germplasms on the sixth day after imbibition. Data indicate the mean ±SD of 30 biological replicates (*n*=30, **P<0.01).

During the investigation of sheepgrass seed germination, we found that sheepgrass germplasm displayed either a yellow or brown seed coat color (Fig. 1A), which may have resulted from different accumulation levels of proanthocyanidins. Therefore, we measured the proanthocyanidin content and found that brown seeds (B) accumulated significantly higher levels of proanthocyanidins than yellow seeds (Y; Fig. 1C, *P*<0.01), in both extractable and non-extractable fractions.

During germination, 50% of the yellow seeds germinated on the second day after imbibition, whereas <20% of the brown seeds germinated (Fig. 1D). Even at 7 d after imbibition, the germination rate of brown seeds was still lower than that of yellow seeds (Fig. 1D). Moreover, the average lengths of both shoots and roots were significantly greater in yellow-seed germplasm than in brown-seed germplasm (Fig. 1B, 1E, *P*<0.01). These results indicate that yellow seeds with less proanthocyanidins germinated more quickly than brown seeds.

### Transcriptome analysis of developing *L. chinensis* seeds

**Fig. 2.**
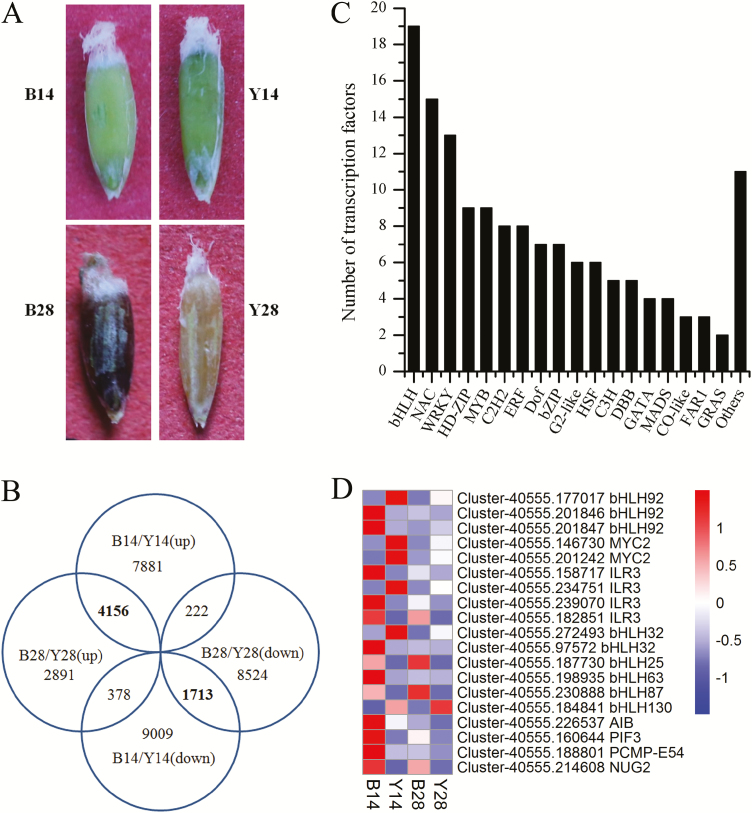
Transcriptome analysis of sheepgrass developing seeds. (A) Phenotype of sheepgrass developing seeds 14 d (B14 and Y14) and 28 d after pollination (B28 and Y28). (B) Venn diagram showing the numbers of differentially expressed genes (DEGs) in sheepgrass developing seeds at 14 d (Y14 and B14) and 28 d after pollination (Y28 and B28). (C) Numbers of differentially expressed transcription factors. (D) Relative expression levels of differentially expressed bHLH transcription factors in sheepgrass developing seeds by heatmap analysis.

To investigate globally the molecular mechanism of seed color formation in sheepgrass, green seeds at 14 d (immature) and 28 d (mature) after pollination with brown-seed germplasm (B14 and B28) and yellow-seed germplasm (Y14 and Y28; Fig. 2A) were selected and used for transcriptome analysis. This analysis revealed that >30 000 genes were differentially expressed >2-fold (Fig. 2B). Among these DEGs, 4156 were up-regulated and 1713 were down-regulated in both B14 and B28 compared with Y14 and Y28 (Fig. 2B; Supplementary Table S1), indicating that the expression differences of these genes resulted from differences in germplasm but not from differences in development stage.

Among the 5869 DEGs, a number of flavonoid biosynthetic pathway genes were highly expressed in the brown-seed germplasm but not the yellow-seed germplasm. These genes included *ANS*, *ANR*, and a proanthocyanidin precursor (epicatechin 3′-*O*-glucoside) transporter protein TT12 (Supplementary Fig. S1). In addition, 144 transcription factors from 30 families were significantly differentially expressed, including 19 members from the bHLH family, 15 from the NAC family, and 13 from the WRKY family (Fig. 2C; Supplementary Table S2). A correlation analysis of transcription factors with *ANS* and *ANR* was then performed at the transcript level by the Pearson and Spearman method (Supplementary Table S3). Finally, 24 transcription factors with the criterion of a correlation coefficient *r* ≥0.7 or *r* ≤ –0.7, and *P* ≤0.05 were acquired. Among them, a *bHLH92* gene (Cluster-40555.177017, annotated as *LcbHLH92*), a homolog of Arabidopsis *bHLH92*, was highly (4-fold) expressed in yellow-seed germplasm (Fig. 2D; Supplementary Table S4). Moreover, the expression level of *LcbHLH92* was significantly negatively correlated with those of *ANR* and *ANS* genes by both Pearson and Spearman correlation analysis (Supplementary Table S3), suggesting a negative relationship of *LcbHLH92* to the expression of *ANR* and *ANS*. Additionally, *LcbHLH92*, another two bHLH, three ERF, and four WRKY transcription factors were overexpressed in Arabidopsis for further functional characterization (Supplementary Table S4). However, only *bHLH92*-overexpressing Arabidopsis showed a phenotype in the control of seed color and stress tolerance, which was further selected for in-depth functional investigation.

### Cloning and sequence analysis of *LcbHLH92*

**Fig. 3.**
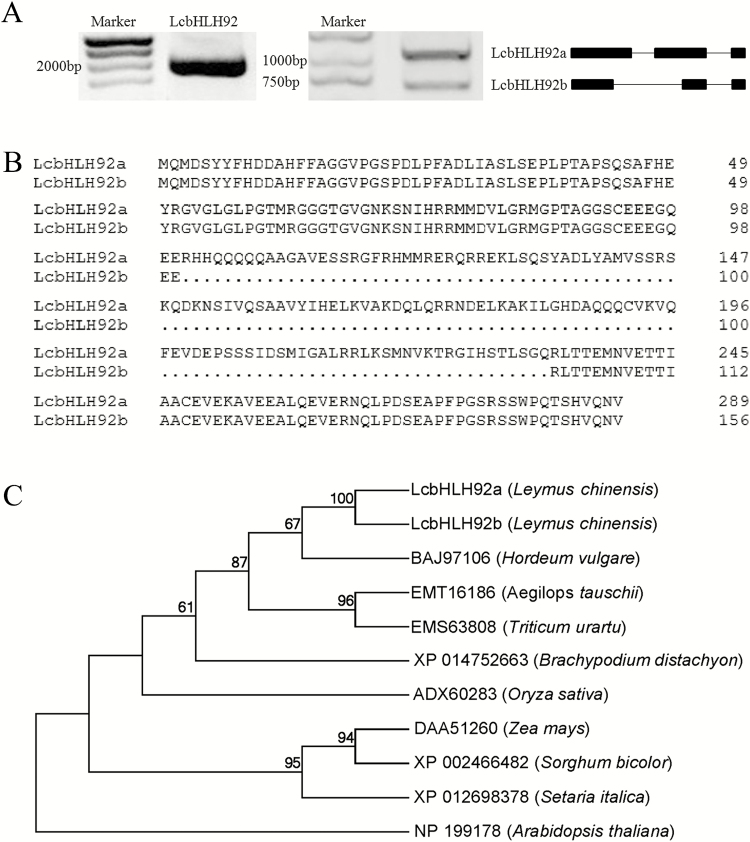
Characterization and sequence analysis of *LcbHLH92* and its two transcripts. (A) PCR products of the *LcbHLH92* gene amplified from DNA detected via 1% agarose gel electrophoresis (left). Two transcripts, *LcbHLH92a* and *LcbHLH92b*, of the *LcbHLH92* gene with 1% agarose gel electrophoresis (middle). Schematic structures of *LcbHLH92a* and *LcbHLH92b* transcripts (right). (B) Sequence alignment of predicted amino acid sequences of *LcbHLH92a* and *LcbHLH92b*. The sequence alignment was performed by using the DNAMAN software (version 7.0). (C) Phylogenetic relationship of LcbHLH92 to its homolog, HLH92, in other plant species. The phylogenetic tree was generated using the maximum likelihood (ML) method with MEGA software (version 6.0), and the numbers near the branches are bootstrap value from 1000 replicates.

To characterize the function of *LcbHLH92* further, its genomic sequence and encoding region were amplified by PCR. The genomic DNA sequence of *LcbHLH92* was amplified as a single product, which was 2045 bp in length (Fig. 3A, left), but two PCR products were obtained when total cDNA was used as template (Fig. 3A, middle). Sequence comparison between the genomic DNA and cDNA revealed that *LcbHLH92* had two transcripts, namely *LcbHLH92a* (1081 bp) and *LcbHLH92b* (682 bp), as a result of the pre-mRNA splice and joining at different sites (Fig. 3A, right). The ORF of *LcbHLH92a* was 873 bp and encoded 290 amino acids, while the ORF of *LcbHLH92b* was 474 bp and encoded 157 amino acids. In comparison with LcbHLH92a, the deduced LcbHLH92b protein lacked a 132 amino acid fragment in the first and second exon (101–233, Fig. 3A, right, Fig. 3B). Both proteins contain a conserved bHLH domain (Supplementary Fig. S2). The DNA and cDNA sequences of the *LcbHLH92* genes were deposited in the NCBI database under accession numbers KY000711 for *LcbHLH92*, KY000709 for *LcbHLH92a*, and KY000710 for *LcbHLH92b*.

Multiple sequence alignment revealed that the deduced LcbHLH92a and LcbHLH92b proteins shared high identity with their homologs from monocot plants *Hordeum vulgare* (89%), *Triticum urartu* (80%), *Aegilops tauschii* (77%), and *Brachypodium distachyon* (71%) (Supplementary Fig. S3). Additionally, sequence alignment of LcbHLH92a, LcbHLH92b, and qSD7-1 (accession number: ABB17166) protein sequences revealed that they showed only 25.32% identity, and *LcbHLH92a* and *LcbHLH92b* were mapped to a partial sequence (accession number: CP018159) of chromosome 3 by blastn to *O. sativa* (indica cultivar group, taxid: 39946), which indicated that the *LcbHLH92* gene is not an ortholog of rice qSD7-1. Furthermore, phylogenetic analysis showed that LcbHLH92a and LcbHLH92b had a close relationship with their homologs from *H. vulgare* and *T. urartu* which are both from Gramineae (Fig. 3C).These findings indicated that bHLH92 from these Gramineae plant species may share a similar function that remains to be identified.

### Expression analysis of *LcbHLH92* in different *L. chinensis* germplasms

**Fig. 4.**
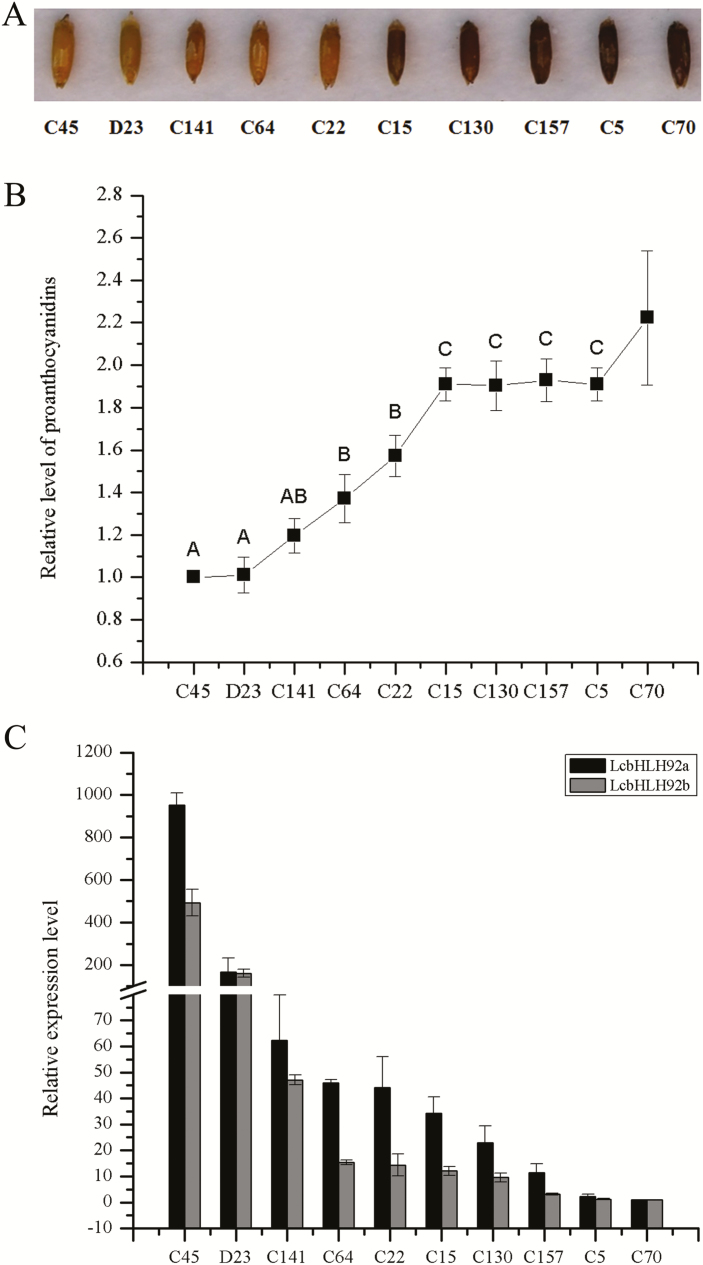
Expression level of *LcbHLH92* and proanthocyanidin analysis in different germplasms of sheepgrass. (A) Seed morphology of different sheepgrass germplasms. (B) Relative proanthocyanidin content in different germplasms of sheepgrass at 28 d after pollination. Data indicate mean ±SDs of three biological replicates. (C) Relative expression level of *LcbHLH92a* and *LcbHLH92b* in different germplasms of sheepgrass at 28 d after pollination. Data indicate mean ±SDs of three technical replicates.

To investigate further the potential relationship of seed coat color and proanthocyanidin content to *LcbHLH92a* and *LcbHLH92b* expression levels in sheepgrass germplasms, the proanthocyanidin content in different germplasms was measured at 28 d after pollination (Fig. 4A). The proanthocyanidin content in several yellow-seed germplasms was significantly lower than in brown-seed germplasms (Fig. 4B). Moreover, qRT-PCR revealed that the relative expression levels of both *LcbHLH92a* and *LcbHLH92b* were higher in yellow-seed than in brown-seed germplasm (Fig. 4C). Additionally, the seed germination test with three type color seeds (yellow, medium, and brown) showed that yellow seeds (N14, N4, C141, and K8) had the highest germination rate, followed by medium type color seeds (C54, S17, S10, and S18); brown seeds (C5, S3, and S2) had the lowest germination rate in the same environments (Supplementary Fig. S4A). Pearson and Spearman correlation analysis revealed that seed germination and seed color had a strong negative correlation (correlation coefficient < –0.8 and *P*<0.01 in Supplementary Fig. S4B). Furthermore, *bHLH92* (Cluster-40555.177017) from sheepgrass had a negative correlation with *ANR* and *ANS* at the transcript level (*r* < –0.7, *P*<0.05 in Supplementary Table S3). These results together indicated that *LcbHLH92* had a negative correlation with the accumulation of proanthocyanidins in sheepgrass seeds.

### Expression profiles and subcellular location of *LcbHLH92*

**Fig. 5.**
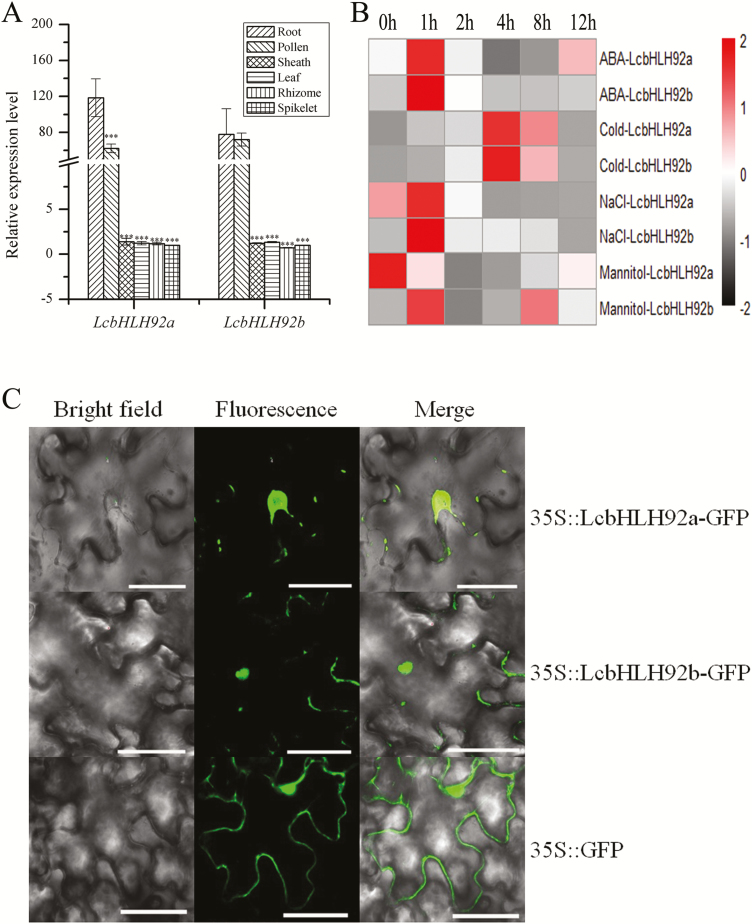
Expression profile and subcellular location of *LcbHLH92a* and *LcbHLH92b*. (A) Relative expression levels of *LcbHLH92a* and *LcbHLH92b* in roots, pollen, sheaths, leaves, rhizomes, and spikelets by qRT-PCR analysis. Data indicate mean ±SDs of three technical replicates (***P<0.001). (B) Expression profile of *LcbHLH92a* and *LcbHLH92b* under ABA, cold, NaCl, and mannitol treatments at 0, 1, 2, 4, 8, and 12 h by qRT-PCR analysis. (C) Subcellular location of *LcbHLH92a* and *LcbHLH92b* in tobacco epidermal cells fused with GFP. Scale bar=60 μm.

To investigate the expression profiles of *LcbHLH92a* and *LcbHLH92b*, qRT-PCR was performed in various tissues with gene-specific primers. The transcripts of both *LcbHLH92a* and *LcbHLH92b* were detected in sheath, leaf, rhizome, and spikelet, but the expression level was very low (Fig. 5A). In contrast, *LcbHLH92a* and *LcbHLH92b* were both more highly expressed in roots and pollen compared with the other tissues (Fig. 5A). Both *LcbHLH92a* and *LcbHLH92b* clearly showed a similar expression pattern in these tested tissues.

Previous studies have shown that plant bHLH transcription factors are involved in abiotic stress tolerance (De Geyter *et al.*, 2012; Ji *et al.*, 2013; Liu *et al.*, 2015). *LcbHLH92* shows a high identity with *AtbHLH92* (Supplementary Fig. S3), and previous studies have indicated that the transcript abundance of *AtbHLH92* increases in response to NaCl, dehydration, mannitol, and cold treatments, and overexpression of *AtbHLH92* moderately improves the tolerance of transgenic Arabidopsis to NaCl and osmotic stresses (Jiang *et al.*, 2009). To compare the function of *LcbHLH92* with *AtbHLH92*, the expression levels of *LcbHLH92a* and *LcbHLH92b* were determined under different treatments, and the resistance of transgenic lines to drought and osmotic stress treatment was also tested (Supplementary Figs S9–S11). The results showed that the expression levels of *LcbHLH92a* and *LcbHLH92b* could be induced by different stress treatments. Their transcript levels could be rapidly induced by ABA and NaCl treatment at 1 h and by cold treatment at 4 h (Fig. 5B). The transcript levels of *LcbHLH92b* were induced by mannitol at 1 h, but mannitol did not induce *LcbHLH92a* transcription (Fig. 5B). These results suggested that *LcbHLH92a* and *LcbHLH92b* could be transiently induced by abiotic stresses, and they responded similarly to ABA, NaCl, and cold treatments, but differently to mannitol treatment, suggesting some differences between *LcbHLH92* and *AtbHLH92* regarding abiotic stress responses.

To investigate the subcellular localization of *LcbHLH92a* and *LcbHLH92b*, their ORFs were fused to GFP and expressed in tobacco epidermal cells under control of the CaMV 35S promoter. A green fluorescence signal was detected in the nucleus for the 35S::LcbHLH92a–GFP and 35S::LcbHLH92b–GFP fusion proteins (Fig. 5C, upper and middle panels) but in the cytosol for the vector control 35S::GFP protein (Fig. 5C, lower panel). Furthermore, the transcriptional activity assay of LcbHLH92 proteins confirmed that LcbHLH92a and LcbHLH92b had transcriptional activity (Suppelementary Figs S5, S6). These results suggest that LcbHLH92a and LcbHLH92b function in the nucleus by activating target downstream genes.

### *LcbHLH92* inhibits anthocyanin biosynthesis in transgenic Arabidopsis leaves

**Fig. 6.**
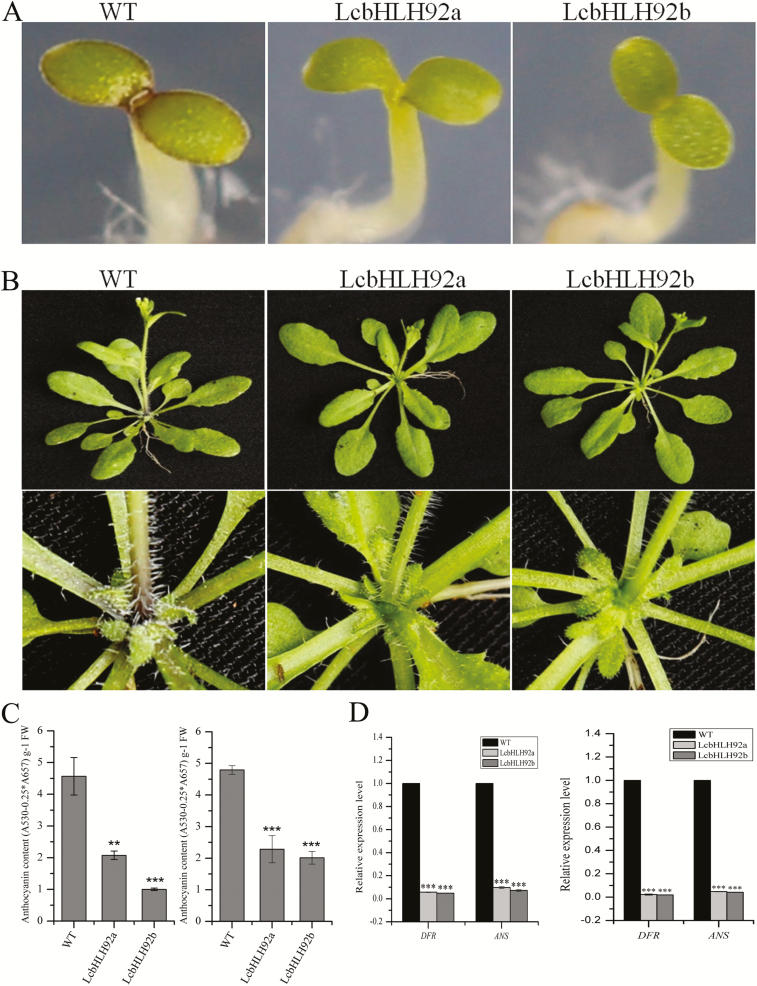
Effects of *LcbHLH92* overexpression on leaf morphology of transgenic *Arabidopsis thaliana*. (A) Cotyledon morphology of 4-day-old seedlings of transgenic *A. thaliana* grown on MS medium. (B) Leaf morphology of 21-day-old plants of transgenic *A. thaliana* grown in soil (upper panels) and the enlarged images of the basal leaves (lower panels). (C) Anthocyanin content of 4-day-old (left) and 21-day-old leaves (right). Data indicate mean ±SDs of three biological replicates (*n*=3, **P<0.01 and ***P<0.001). (D) Relative expression levels of *DFR* and *ANS* in 4-day-old (left) and 21-day-old leaves (right). Data indicate mean ±SDs of three biological replicates (*n*=3, ***P<0.001).

To characterize the *in vivo* function of *LcbHLH92* functionally, its two transcripts were overexpressed separately in Arabidopsis driven by the CaMV 35S promoter. High expression levels of *LcbHLH92a* and *LcbHLH92b* were confirmed by RT-PCR (Supplementary Fig. S8). Purple pigments could be observed in cotyledon and rosette leaves in WT Arabidopsis (Fig. 6A, B, left panels), whereas purple pigments disappeared in transgenic lines overexpressing either *LcbHLH92a* or *LcbHLH92b* (Fig. 6A, B, middle and right panels). Quantitative analysis revealed that anthocyanin content in transgenic *A. thaliana* was reduced by >2-fold in comparison with WT cotyledon (Fig. 6C, left) and seedlings (Fig. 6C, right). Transcript analysis revealed that the expression levels of both *DFR* and *ANS*, the two key structural genes in the anthocyanin pathway, were significantly inhibited by *LcbHLH92a* and *LcbHLH92b* in both cotyledon (Fig. 6D, left) and seedlings (Fig. 6D, right) of transgenic Arabidopsis. These results demonstrated that *LcbHLH92a* and *LcbHLH92b* inhibited the expression levels of *DFR* and *ANS*, which in turn resulted in a decrease in anthocyanins in the leaves of transgenic Arabidopsis.

### *LcbHLH92* inhibits proanthocyanidin biosynthesis and promotes seed germination of transgenic Arabidopsis

**Fig. 7.**
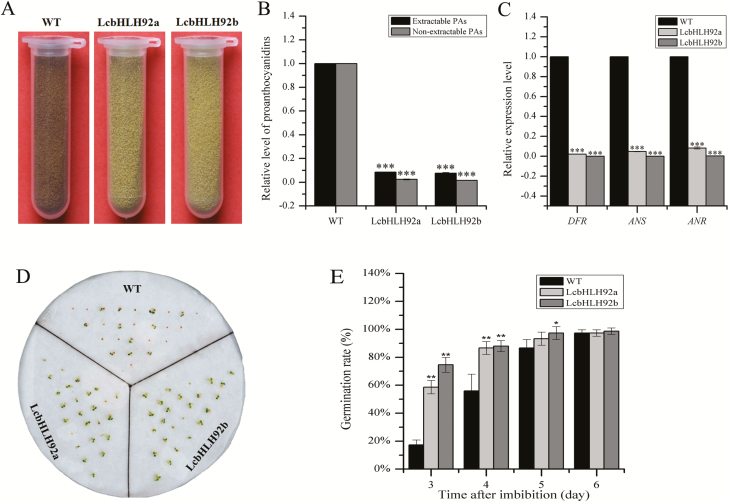
Effect of *LcbHLH92* overexpression on seed pigmentation of transgenic Arabidopsis. (A) Seed morphology of wild-type and transgenic Arabidopsis overexpressing *LcbHLH92.* (B) Relative proanthocyanidin content of wild-type and transgenic line seeds. Data indicate mean ±SDs of three biological replicates (*n*=3, ***P<0.001). (C) Relative expression level of *DFR*, *ANS*, and *ANR* in developing seeds (7 d after pollination). Data indicate mean ±SDs of three biological replicates (*n*=3, ***P<0.001). (D) Germination assay of wild-type and transgenic Arabidopsis overexpressing *LcbHLH92a* or *LcbHLH92b* genes on filter paper. (E) Germination rates of wild-type and transgenic Arabidopsis at different time points. Data indicate mean ±SDs of three biological replicates (*n*=3, *P<0.05 and **P<0.01).

We found that the seed coat of the transgenic Arabidopsis displayed a yellow color, which was in sharp contrast to the brown seed coat of the WT Arabidopsis (Fig. 7A). Because the seed coat mainly consists of proanthocyanidins that confer a brown color to the testa in Arabidopsis, we measured the proanthocyanidin content in both transgenic lines and the WT control. The results revealed that both the extractable and non-extractable proanthocyanidin portions were significantly reduced in the seeds of the transgenic lines (*P*<0.001; Fig. 7B), which is consistent with their yellow seed color. The relative expression levels of *DFR*, *ANS*, and *ANR* were significantly down-regulated in immature seeds (7 d after pollination) in transgenic Arabidopsis in comparison with the WT control (*P*<0.001; Fig. 7C). Taken together, these results demonstrated that *LcbHLH92* functioned as a negative regulator in proanthocyanidin biosynthesis in Arabidopsis seeds. We obtained 15 and 20 independent transgenic lines for *LcbHLH92a* and *LcbHLH92b* separately, and all the transgenic lines showed a similar phenotype with yellow seed coat color (Supplementary Fig. S7). These results indicated that the yellow seeds arose from the overexpression of *LcbHLH92a* or *LcbHLH92b* because the T-DNA insertion is randomly distributed throughout the genome (Franzmann *et al.*, 1995).

To investigate any potential effects of *LcbHLH92* overexpression on seed germination, rates of seed germination were measured, and the results showed that seeds from the transgenic lines germinated more rapidly than those of the WT (Fig. 7D). More than 50% and 80% of the seeds of transgenic lines germinated on day 3 and day 4, but only <20% and 60% of the WT germinated on the same days, respectively (Fig. 7E). Taken together, this finding demonstrated that the overexpression of *LcbHLH92* resulted in a higher seed germination rate.

### *LcbHLH92* inhibits anthocyanin biosynthesis under MeJA and drought treatments

**Fig. 8.**
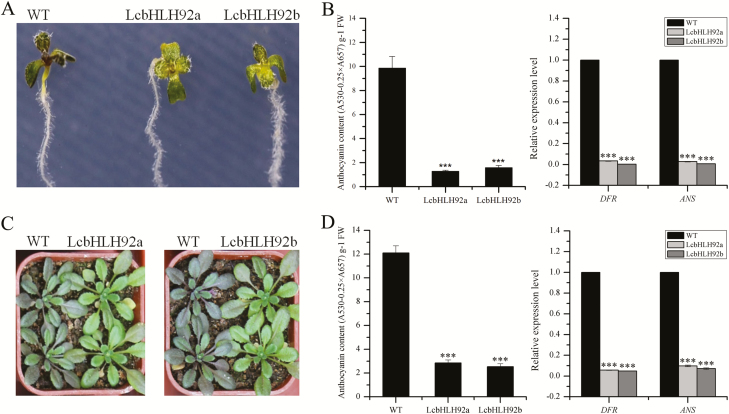
Effects of *LcbHLH92* overexpression on transgenic Arabidopsis under MeJA and drought treatment. (A) Leaf morphology of wild-type and transgenic Arabidopsis under MeJA (100 µmol l^–1^) treatment. (B) Anthocyanin content (left) and relative expression level of *DFR* and *ANS* (right) in wild-type and transgenic Arabidopsis under MeJA (100 µmol l^–1^) treatment. Data indicate mean ±SDs of three biological replicates (*n*=3, ***P<0.001). (C) Leaf morphology of wild-type and transgenic Arabidopsis under drought treatment. (D) Anthocyanin content (left) and relative expression level of *DFR* and *ANS* (right) in wild-type and transgenic Arabidopsis under drought treatment. Data indicate mean ±SDs of three biological replicates (*n*=3, ***P<0.001).

As shown in Fig. 5B, the expression levels of *LcbHLH92a* and *LcbHLH92b* were both induced by the abiotic stress response, and we found that WT Arabidopsis leaves accumulated more purple pigments, but not the transgenic Arabidopsis overexpressing *LcbHLH92a* or *LcbHLH92b* under methyl jasmonate (MeJA; 100 µmol l^–1^) and drought treatment (Fig. 8A, C). The anthocyanin content was 10-fold lower in transgenic lines than in the WT (Fig. 8B, left), which is consistent with the reduced relative expression levels of both *DFR* and *ANS* (Fig. 8B, right). Furthermore, both the anthocyanin content and expression levels of *DFR* and *ANS* were significantly inhibited in transgenic Arabidopsis in comparison with the WT control (Fig. 8D). These results together indicated that *LcbHLH92* inhibited anthocyanin biosynthesis in transgenic Arabidopsis, even under MeJA and drought treatments.

### LcbHLH92 enhances the transcripts levels of jasmonate-ZIM domain (JAZ) proteins

**Fig. 9.**
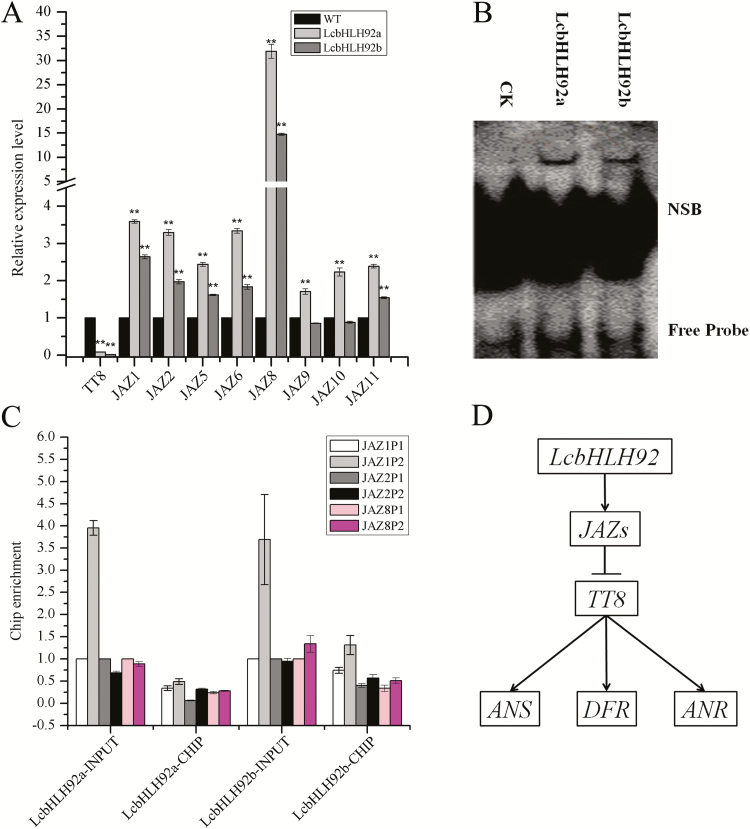
Exploration of the mechanism of *LcbHLH92* in Arabidopsis. (A) Relative expression level of *TT8* and *JAZ* genes in the wild-type and transgenic Arabidopsis under MeJA treatment. Data indicate mean ±SDs of three biological replicates (*n*=3, **P<0.01). (B) The binding of LcbHLH92a and LcbHLH92b to their target element *in vitro* revealed by EMSA; NSB, non-specific binding. (C) The detection of LcbHLH92a and LcbHLH92b binding to the promoters of *AtJAZ1*, *AtJAZ2*, and *AtJAZ8* by qRT-PCR. (D) Hypothetical regulatory network of LcHLH92 in transgenic Arabidopsis.

In a previous study, JAZ proteins were shown to interact with TT8 and MYB75 to regulate jasmonate-mediated anthocyanin accumulation (Qi *et al.*, 2011). As LcbHLH92a and LcbHLH92b are also involved in anthocyanin inhibition, they were also investigated for a potential relationship with *JAZ* genes, including *AtJAZ1*, *AtJAZ2*, *AtJAZ6*, *AtJAZ8*, *AtJAZ9*, *AtJAZ10*, and *AtJAZ11*, together with *TT8*. The expression of *TT8* was significantly inhibited, whereas the *JAZ* genes were all activated by *LcbHLH92a* and *LcbHLH92b* in transgenic Arabidopsis (Fig. 9A). In particular, the expression levels of *AtJAZ1*, *AtJAZ2*, *AtJAZ6*, and *AtJAZ8* were much higher than those of the other *JAZ* genes (Fig. 9A). Furthermore, the core DNA sequence motif recognized by bHLH proteins is a consensus hexanucleotide sequence known as the E/G-box (5'-CANNTG/CACGTG-3') (Xu *et al.*, 2015). To validate this deduction, the putative promoters (2000 bp upstream of the transcription start site) of *AtJAZ1*, *AtJAZ2*, *AtJAZ5*, *AtJAZ6*, *AtJAZ8*, *AtJAZ9*, *AtJAZ10*, and *AtJAZ11* were analyzed using Perl scripts to identify the G-box or E-box (Supplementary Table S6). We confirmed the direct binding of LcbHLH92 to the G-box using EMSA (Fig. 9B). A shifted DNA–protein complex band was detected when the fusion protein of LcbHLH92 was cultivated with probe, whereas no shift was observed in the absence of the fusion protein (Fig. 9B). In addition, the ChIP-qPCR showed that the promoters were specifically enriched by the anti-FLAG antibody (fused to LcbHLH92a or LcbHLH92b; Fig. 9C), which further confirmed the interaction between LcbHLH92a or LcbHLH92b with the promoters of target JAZ DNA *in vivo*. The combined analysis using genetic and molecular approaches strongly supported that LcbHLH92a and LcbHLH92b could directly bind to the promoters of *JAZ* genes and promote their expression. Taken together, we speculate that LcbHLH92 proteins inhibit the expression of *TT8* by activating *JAZ* genes, which in turn inhibit downstream target genes in the flavonoid regulation network, such as *ANS*, *DFR*, and *ANR* (Fig. 9D).

## Discussion

### *LcHLH92*, an interesting transcription factor gene from sheepgrass

Plant bHLH transcription factors are involved in multiple biological processes, such as abiotic stress tolerance, pollen development, and secondary metabolism (De Geyter *et al.*, 2012; Ji *et al.*, 2013; Jiang *et al.*, 2015). In *A. thaliana*, the transcript abundance of *AtbHLH92* increases in response to NaCl, dehydration, mannitol, and cold treatments, as the overexpression of *AtbHLH92* moderately improves the tolerance of transgenic Arabidopsis to NaCl and osmotic stresses (Jiang *et al.*, 2009). In *Brassica rapa*, the transcription of *BrbHLH92* can be induced by heat (Dong *et al.*, 2015). These previous studies have demonstrated that bHLH92 transcription factors are involved in stress responses. In the present study, we found two transcripts of *LcbHLH92a* and *LcbHLH92b* from sheepgrass that contain alternative first and second exons (Fig. 3A). Both *LcbHLH92a* and *LcbHLH92b* were predominantly expressed in root and pollen, and significantly induced by ABA, NaCl, and cold treatments (Fig. 5A, B), indicating that *LcbHLH92a* and *LcbHLH92b* are also involved in abiotic stress responses like *bHLH92* from other plant species. Furthermore, the two isoforms responded differently to osmotic stress treatment, as the expression level of *LcbHLH92b*, but not of *LcbHLH92a*, was significantly induced by mannitol treatment in leaf (Fig. 5B), which indicates that *LcbHLH92b* is widely involved in abiotic stress responses. Similarly, *CsLIS/NES-1* and *CsLIS/NES-2*, two transcription isoforms of *CsLIS/NES*, catalyze rerolidol and linalool biosynthesis, respectively, in tea plant (Liu *et al.*, 2018). In *Lotus japonicus*, one of the two splicing variants of *DFR* shows enzymatic activity, and the other one does not (Shimada *et al.*, 2005), which is different from *LcbHLH92*, as both transcripts are functional. These results together indicate that post-transcriptional processing is critical for *LcbHLH92* gene functional diversity. In addition, the expression level of *LcbHLH92* is negatively related to the proanthocyanidin content in seeds (Figs 1, 4). These studies indicate that *LcbHLH92* may play multiple roles in sheepgrass. Phylogenetic analysis of bHLH92 proteins revealed that sheepgrass has a close relationship with *H. vulgare* and *T. urartu* (Fig. 3C); therefore, similar functions may occur in monocots, which will require further investigation. However, *LcbHLH92* functions as a negative regulator of the anthocyanin/proanthocyanidin pathway, which has not been previously reported in sheepgrass and other plant species.

### LcHLH92 acts as a key repressor of the anthocyanin/proanthocyanidin pathway

Although the underlying mechanism of *LcbHLH92* in the regulation of proanthocyanidin biosynthesis in sheepgrass and other monocot species is not clear, transcriptome analysis of developing sheepgrass seeds revealed that *LcbHLH92* (Cluster-40555.177017) was expressed at a higher level in yellow seeds than in brown seeds (Fig. 2D), and yellow seeds had a lower proanthocyanidin content (Figs 1, 4). The main structural genes controlling proanthocyanidin synthesis are *CHS*, *CHI*, *DFR*, and *ANS* in wheat and barley (Himi *et al.*, 2005, 2012), whereas the transcript levels of *ANS* and *ANR* were lower in yellow seeds of sheepgrass (Supplementary Fig. S1). Our results indicated a negative correlation between the transcription levels of *LcbHLH92* and *ANR*, as revealed by Pearson and Spearman analyses (Supplementary Table S3). Subcellular location and transcriptional activity analyses of *LcbHLH92* indicated that it mainly functioned as a regulator (Fig. 5C; Supplementary Fig. S5). These results together suggest that *LcbHLH92* may inhibit the transcription of *ANS* and/or *ANR*.

Since genetic transformation in sheepgrass is still very difficult (Wang *et al.*, 2009), the hypothesis concerning the functions of *LcbHLH92* was verified in Arabidopsis overexpressing *LcbHLH92a* or *LcbHLH92b*. In accordance with the phenomenon in sheepgrass, Arabidopsis seeds overexpressing *LcbHLH92a* or *LcbHLH92b* displayed a yellow seed coat and lower proanthocyanidin content than the WT seeds , and the relative expression levels of *DFR*, *ANS*, and *ANR* were significantly down-regulated in immature seeds of transgenic plant (Fig. 7). Furthermore, WT Arabidopsis showed purple pigments in cotyledon and rosette leaves, while the purple pigments disappeared in transgenic lines overexpressing *LcbHLH92* (Fig. 6). Moreover, the leaves of the WT Arabidopsis accumulated more purple pigments, but those of transgenic Arabidopsis plants were still green under MeJA and drought treatments (Fig. 8). Further analysis of the anthocyanin content and expression levels of *DFR* and *ANS* indicated that *LcbHLH92a* and *LcbHLH92b* inhibited the expression levels of *DFR* and *ANS*, which in turn resulted in a decrease in anthocyanins in the leaves of transgenic Arabidopsis (Figs 6, 8). In wheat, two transcription factor genes were characterized, *TaPpm1* (purple pericarp-MYB 1) and *TaPpb1* (purple pericarp-bHLH 1), which co-regulate the anthocyanin biosynthesis in purple pericarp of wheat (Jiang *et al.*, 2018). In Arabidopsis, MYBL2 binds directly to TT8 protein and suppresses the expression of *DFR* (Matsui *et al.*, 2008). Therefore, our results suggest that *LcbHLH92* is a negative regulator of the anthocyanin/proanthocyanidin pathway in transgenic Arabidopsis.

### *LcHLH92* enhances seed germination

In this study, the yellow-seed sheepgrass germplasm had lower proanthocyanidin content and germinated more quickly than brown seeds (Fig. 1B, C), indicating that seeds with different seed color from various germplasms have different germination rates in sheepgrass. *TaMYB10* and *HvMYB10* control grain colors by regulating proanthocyanidin synthesis through restricting *DFR* expression in the developing seeds of wheat and barley, respectively, thus affecting grain dormancy (Himi et al., 2011, 2012). However, the phenomenon has not been reported for bHLH transcription factors, which promote seed germination by inhibiting proanthocyanidin biosynthesis in monocot species. Therefore, *LcHLH92* is a candidate target for genetic manipulation in new germplasm production of pre-harvest sprouting tolerance or in breaking seed dormancy by overexpression of the *LcHLH92* gene. In addition, Arabidopsis seeds overexpressing *LcbHLH92a* or *LcbHLH92b* display a yellow seed coat, lower proanthocyanidin content, and a higher germination rate than WT seeds (Fig. 7). Previous studies suggest that the absence or reduction of proanthocyanidins in the endothelium of the *tt* mutant seeds is related to reduced seed dormancy in *A. thaliana* (Debeaujon *et al.*, 2000). TT2, TTG1, and TT8 regulate *DFR* and *ANR* expression to determine the accumulation of proanthocyanidins in immature seeds (Nesi et al., 2000, 2001; Baudry *et al.*, 2004). Mutations in any of the MYB or bHLH domain proteins in the complex can result in a lack of pigmentation in the flower and seed coat (Nesi *et al.*, 2000, 2001; Baudry *et al.*, 2004). Interestingly, TT8 autoregulates its expression through the formation of different MBW protein complexes with several R2R3-MYBs and TTG1 (Baudry *et al.*, 2006; Xu *et al.*, 2013). Previous studies have revealed that seed germination can be suppressed by proanthocyanidins by increasing testa mechanical resistance, which restrains radicle elongation and promotes *de novo* biogenesis of ABA, inducing and maintaining seed dormancy (Debeaujon *et al.*, 2000; Winkel-Shirley, 2001; Finkelstein *et al.*, 2008; Jia *et al.*, 2012; Chen *et al.*, 2014; MacGregor *et al.*, 2015). Thus, our results imply that *LcbHLH92* plays a key role in promoting seed germination and reducing seed dormancy.

### The possible mechanistic model of *LcHLH92* in transgenic Arabidopsis

Previous studies indicate that *TT8* is expressed in both the endothelium and epidermal cells of the seed (Haughn and Chaudhury, 2005), and TT8 forms the MBW complex with TT2 and TTG1 to promote proanthocyanidin biosynthesis through activation of the *DFR*, *LDOX*, and *BAN* genes (Baudry *et al.*, 2004; Routaboul *et al.*, 2006; Xu *et al.*, 2014). JAZ proteins inhibit TT8 expression, resulting in reduced anthocyanin accumulation (Qi *et al.*, 2011). In the present study, we used ChIP and qPCR analysis to reveal that LcbHLH92a and LcbHLH92b bound to the promoters of JAZ-encoding genes to activate their transcription (Fig. 9C). Moreover, the expression of *TT8* was significantly inhibited, whereas the *JAZ* genes were all activated by *LcbHLH92a* and *LcbHLH92b* in transgenic Arabidopsis (Fig. 9A). Therefore, we suggest that the transcription of *TT8* may be suppressed by abundant JAZ protein accumulation. Consequently, the accumulation of proanthocyanidins in transgenic plant was reduced under low expression levels of *DFR*, *ANR*, and *ANS* (Fig. 7A, B, C). Overall, we speculate a possible model for how anthocyanins/proanthocyanidins are regulated by the transcription factor gene *LcbHLH92* in Arabidopsis (Fig. 9D). High levels of *LcbHLH92* activate *JAZ* genes, which inhibit the expression level of *TT8* to down-regulate the structural genes *DFR* and *ANS*. As a consequence, anthocyanin and proanthocyanidin levels are decreased in leaves and seeds of transgenic Arabidopsis, respectively (Figs 6–8).

### Conclusion

This work extends our understanding of the regulation of the anthocyanin/proanthocyanidin pathway, especially in monocots. We first identified a bHLH-type transcription factor gene, *LcbHLH92*, in sheepgrass, and found that its expression level is negatively correlated with seed color and *ANS* and *ANR* transcript levels. Overexpression of *LcbHLH92* in *A. thaliana* results in reduced accumulation of anthocyanins and proanthocyanidins compared with the WT in leaves and seeds, respectively, and further promotes seed germination. Together, these results indicate that *LcbHLH92* is a valuable locus for genetic manipulation in the future.

## Supplementary data

Supplementary data are available at *JXB* online.

Fig. S1. Relative expression levels of genes involved in the flavonoid pathway.

Fig. S2. Protein structure prediction of LcbHLH92a and LcbHLH92b by SWISS- MODEL.

Fig. S3. Multiple sequence alignment of LcbHLH92a and LcbHLH92b with their orthologs.

Fig. S4. Germination rate of different germplasms with different seed coat colors.

Fig. S5. Transcriptional activation assay of LcbHLH92a and LcbHLH92b in Arabidopsis protoplasts.

Fig. S6. Transcriptional activation assay of LcbHLH92a and LcbHLH92b in yeast.

Fig. S7. Seed coat color of different transgenic lines and wild type Arabidopsis.

Fig. S8. Relative expression levels of *LcbHLH92a* and *LcbHLH92b* in transgenic Arabidopsis by RT-PCR analysis.

Fig. S9. Drought stress resistance assay of transgenic lines under natural conditions.

Fig. S10. Natural drought stress assay of transgenic lines at the flowering stage.

Fig. S11. Osmotic tress assay of transgenic lines under 300 mM mannitol treatment

Table S1. Differentially expressed genes (DEGs).

Table S2. Putative transcription factors from DEGs.

Table S3. Correlation analysis of transcription factor genes with *ANR* and *ANS* at the transcript level.

Table S4. Selected transcription factors.

Table S5. Primer sequences used in the present study.

Table S6. Promoter sequence of some *JAZ* genes.

Supplementary Figures S1-S11

Supplementary Tables S1-S6

## Author contributions

GSL and LQC conceived the original screening and research plans; PCZ, XXL, and LQC performed most of the experiments; PCZ and XXL analyzed the data and wrote the article; JJT, GXY, SYC, and DMQ provided assistance with this research.

## Abbreviations:

ABA: abscisic acid
ANR: anthocyanidin reductase
ANS: anthocyanidin synthase
bHLH: basic helix–loop–helix
CaMV: *Cauliflower mosaic virus*
DEG: differentially expressed gene
DFR: dihydroflavonol 4-reductase
GFP: green fluorescent protein
JAZ: Jasmonate-ZIM domain protein
MBW: MYB–bHLH–WD40 protein complex
MeJA: methyl jasmonate
MS: Murashige and Skoog
MYB: MYB DNA-binding domain protein
qRT-PCR: real-time quantitative PCR
tt: transparent testa
TT8: Transparent Testa8
TTG1: TRANSPARENT TESTA GLABRA1
WT: wild type
