# Corrigendum: bHLH92 from sheepgrass acts as a negative regulator of anthocyanin/proanthocyandin accumulation and influences seed dormancy

**DOI:** 10.1093/jxb/erz017

**Published:** 2019-03-11

**Authors:** Pincang Zhao, Xiaoxia Li, Junting Jia, Guangxiao Yuan, Shuangyan Chen, Dongmei Qi, Liqin Cheng, Gongshe Liu

**Affiliations:** 1 Key Laboratory of Plant Resources, Institute of Botany, Chinese Academy of Sciences, Beijing, China; 2 College of management science and engineering, Hebei University of Economics and Business, China

*Journal of Experimental Botany*, Volume 70, Issue 1, 1 January 2019, Pages 269–284, https://doi.org/10.1093/jxb/ery335

In the discussion section of this paper references to LcHLH92 have been corrected to LcbHLH92. The authors apologize for the error.

