# Supplementary material for: bHLH92 from sheepgrass acts as a negative regulator of anthocyanin/proanthocyandin accumulation and influences seed dormancy
Source: J Exp Bot. 2018 Sep 18;70(1):269–84. doi: 10.1093/jxb/ery335 (PMC6354636; doi:10.1093/jxb/ery335)
Supplement: Supplementary Figures S1-S11 [file ery335_suppl_supplementary_figures_s1-s11.pdf]

Supplemental Figures

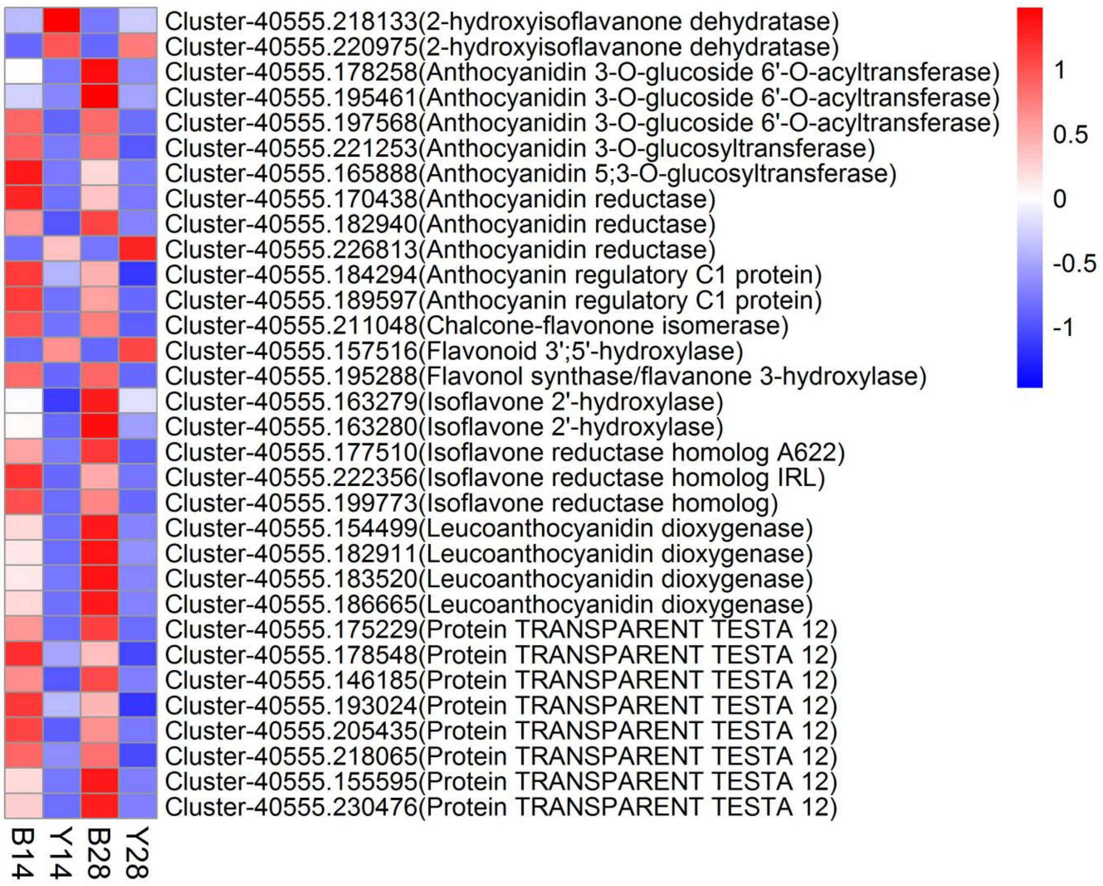

Figure S1. Relative expression levels of genes involved in flavonoid pathway.
The average FPKM value was used to plot the heatmap in R environment by using pheatmap package.

A

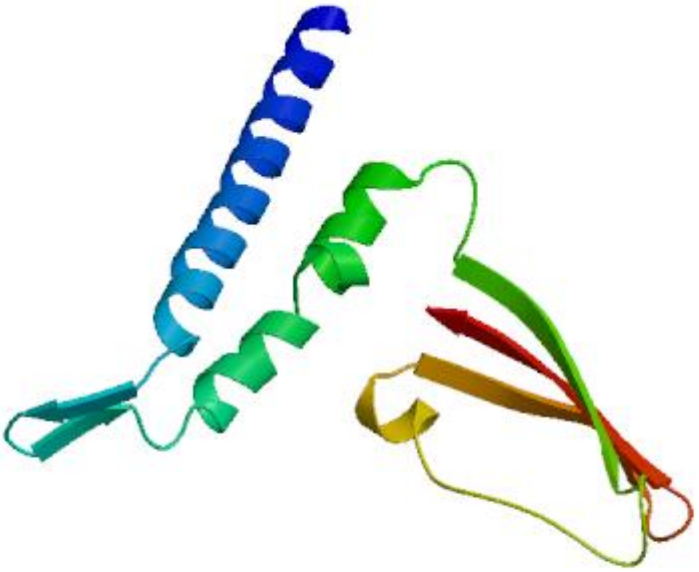

B

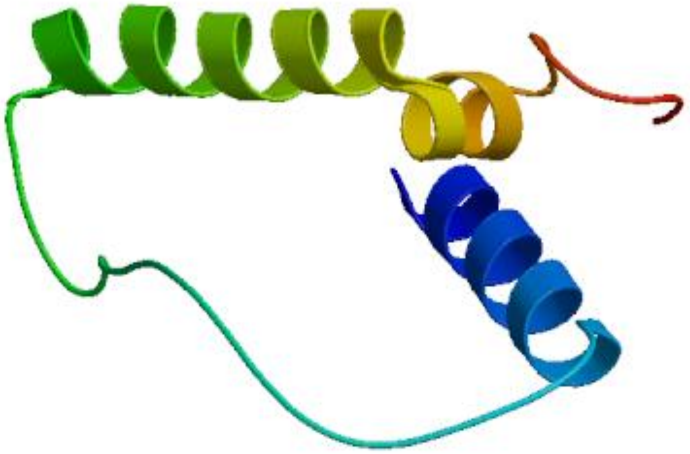

Figure S2 Protein structure prediction of LcbHLH92a and LcbHLH92b by SWISS- MODEL.
A: The structure of LcbHLH92a; B: The structure of LcbHLH92b

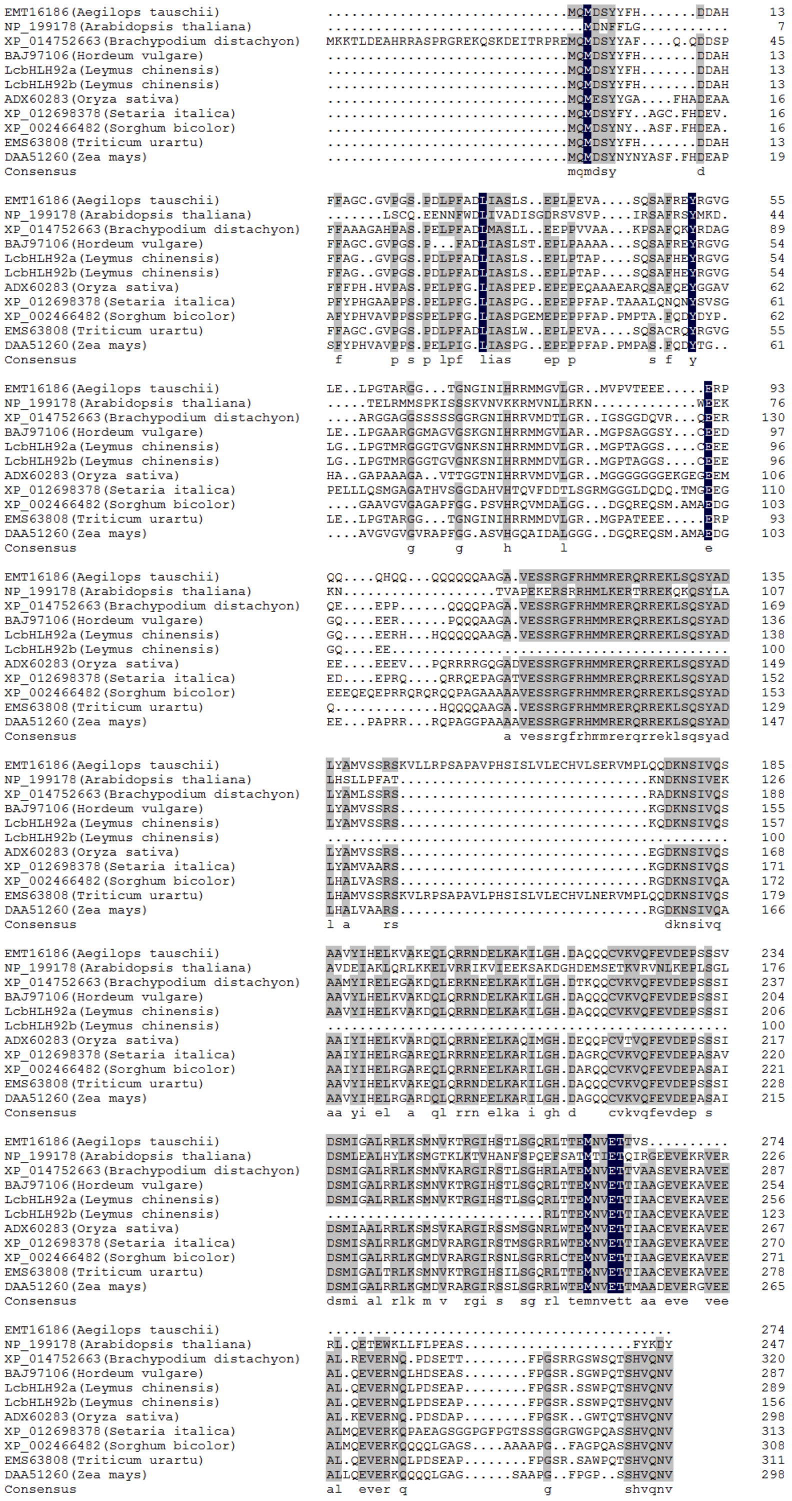

Figure S3. Multiple sequence alignment of LcbHLH92a and LcbHLH92b with their orthologs. Amino acid sequences were aligned using DNAMAN software (7.0). Identical amino acids were indicated in dark background and amino acids with > 75% sequence similarity were indicated in gray background.

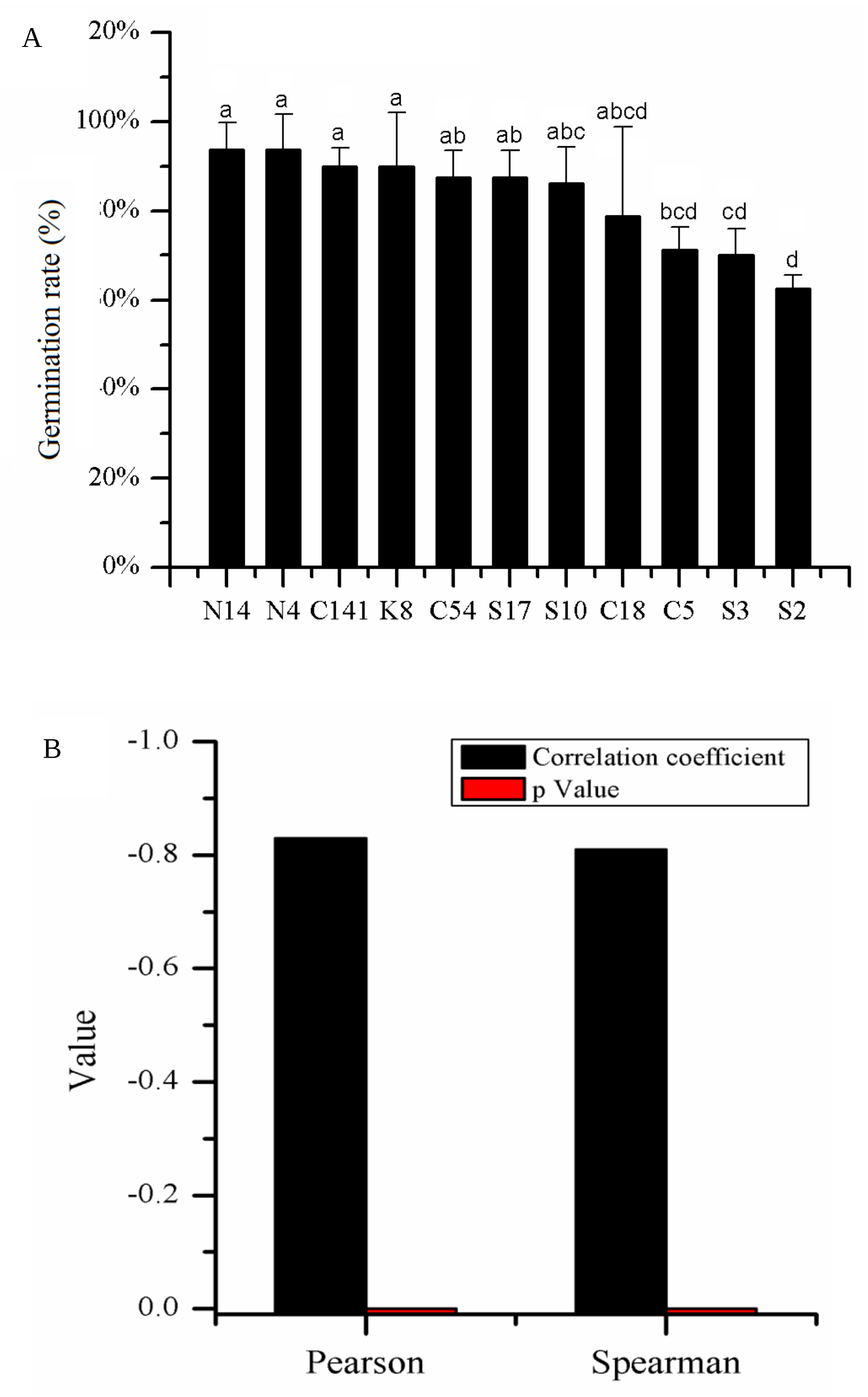

Figure S4. Germination rate of different germplasms with different seed coat colors

A: N14, N4, C141 and K8 are yellow seed coat; C5, S3 and S2 are brown seed coat; C54, S17, S10, S18 are medium type color.

B: Correlation analysis between germination rate and seed coat color by Pearson and Spearman method, $p < 0.01$.

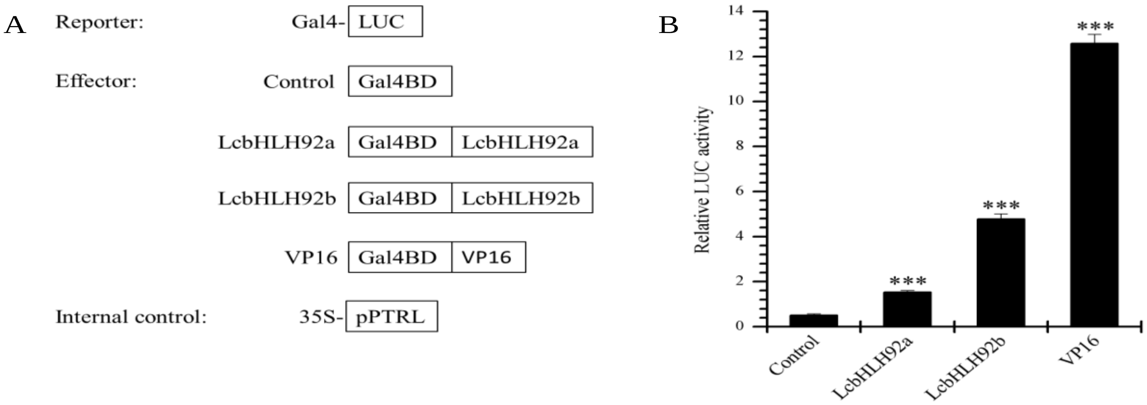

Figure S5. Transcriptional activation assay of LcbHLH92a and LcbHLH92b in *Arabidopsis* protoplast.

(A) Schematic diagram of constructs used in the present study.

(B) Transcript activity of LcbHLH92a or LcbHLH92b to reporter gene as revealed by relative LUC activity. Relative LUC activity was determined by the ratio of LUC activity to pPTRL levels. Gal-BD and GAL-BD-VP16 were used as negative and positive control, respectively. Data indicate mean ± standard deviations of three biological replicates. (n = 3, *** means $p < 0.001$)

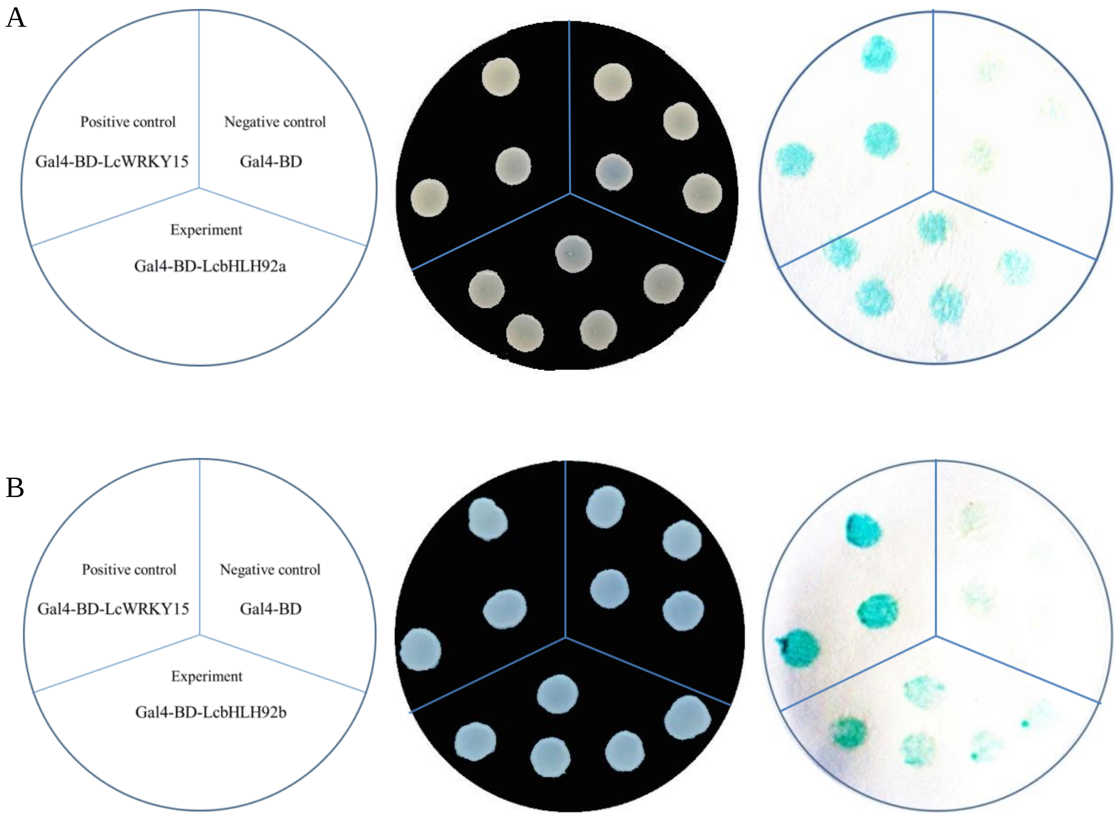

Figure S6. Transcriptional activation assay of LcbHLH92a and LcbHLH92b in yeast.

(A-B) The transcriptional activation activity of LcbHLH92a (A) and LcbHLH92a (B). Left panels: the position indication of yeast cells; Middle panels: the recombinants in AH109 on SD/-trp medium; Right panels: β-galactosidase activity revealed by x-gluc coloring.

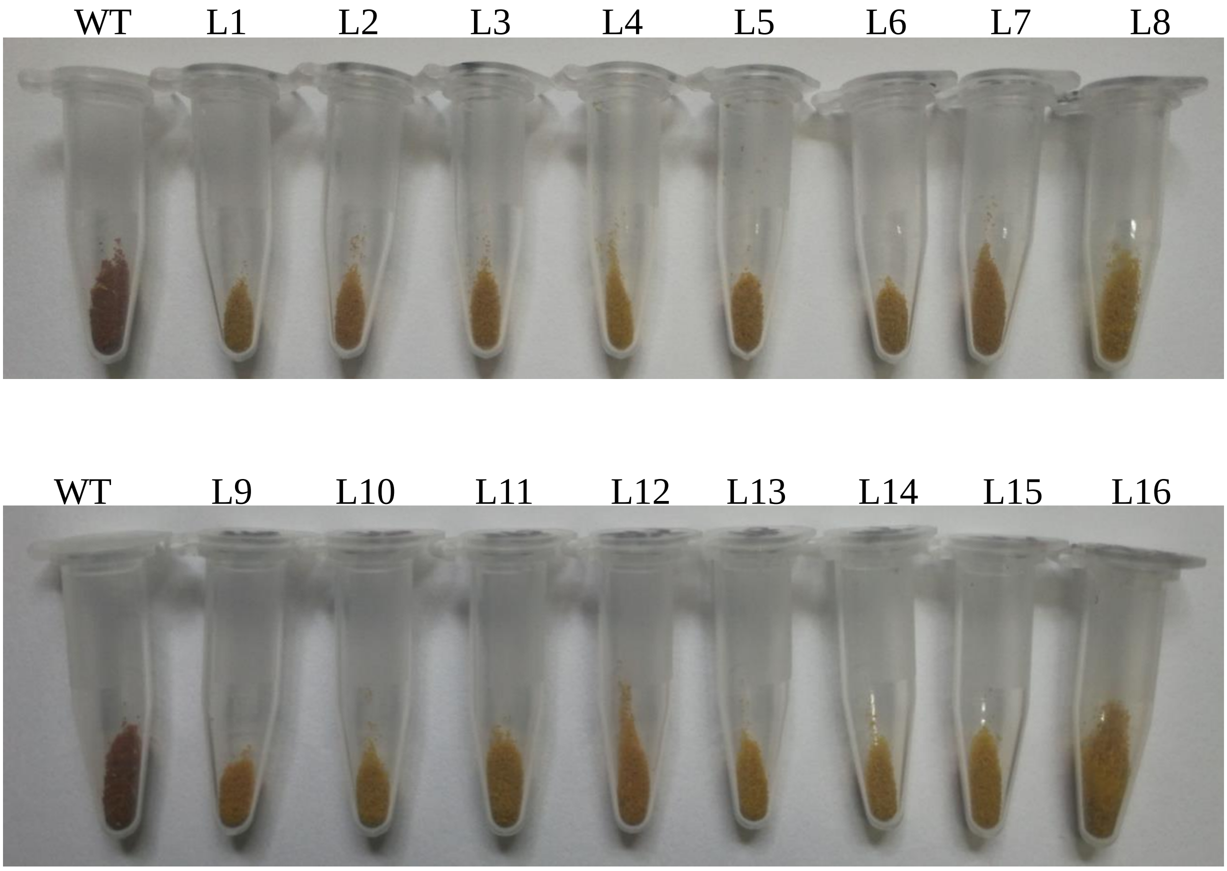

Figure S7. Seed coat color of different transgenic lines and wild type *Arabidopsis*
WT: wild type;
L1, L2, L3, L4, L5, L6, L7, L8: overexpression of *LcbHLH92a*;
L9, L10, L11, L12, L13, L14, L15, L16: overexpression of *LcbHLH92b*.

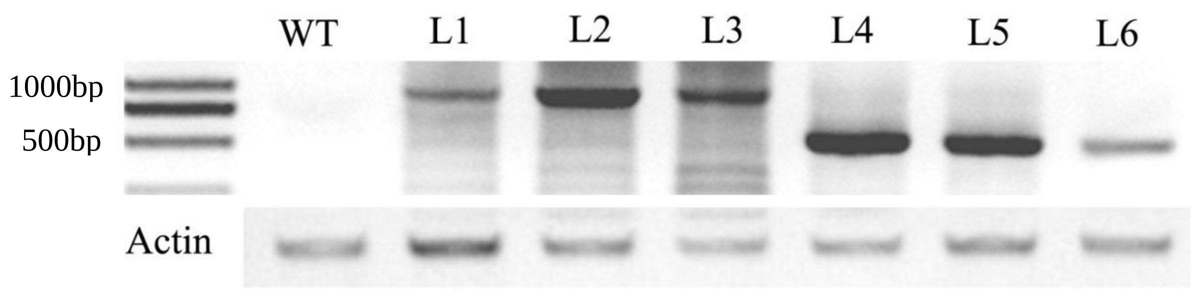

Figure S8. Relative expression levels of *LcbHLH92a* and *LcbHLH92b* in transgenic *Arabidopsis* by RT-PCR analysis.

L1, L2, L3 were transgenic *Arabidopsis* over-expressing *LcbHLH92a* and L4, L5, L6 were transgenic *Arabidopsis* over-expressing *LcbHLH92b*.

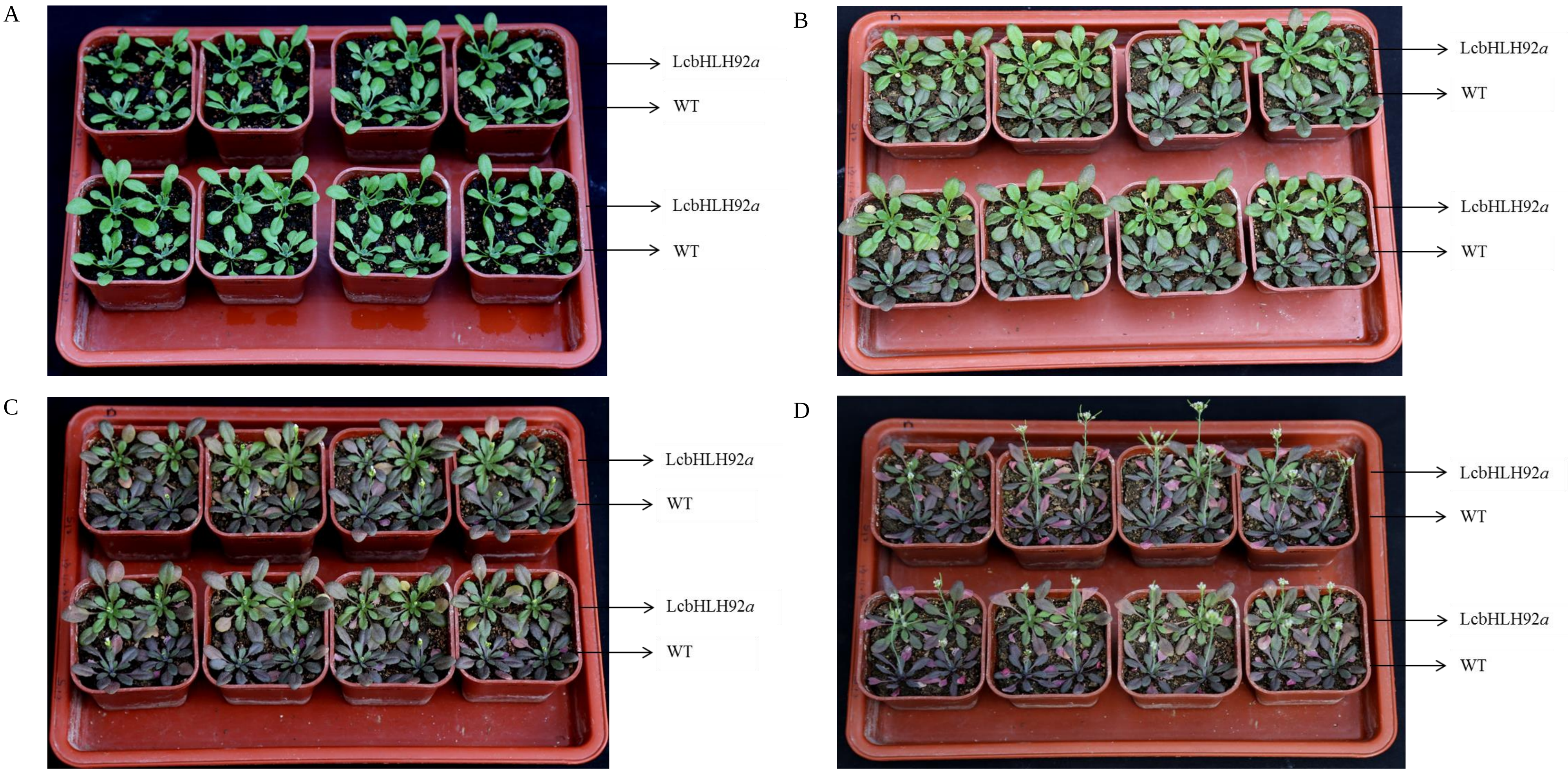

Figure S9. Drought stress resistance assay of transgenic lines under natural conditions

A: Control check

B: Drought stress (without watering) for 7 days

C: Drought stress (without watering) for 10 days

D: Drought stress (without watering) for 15 days

A

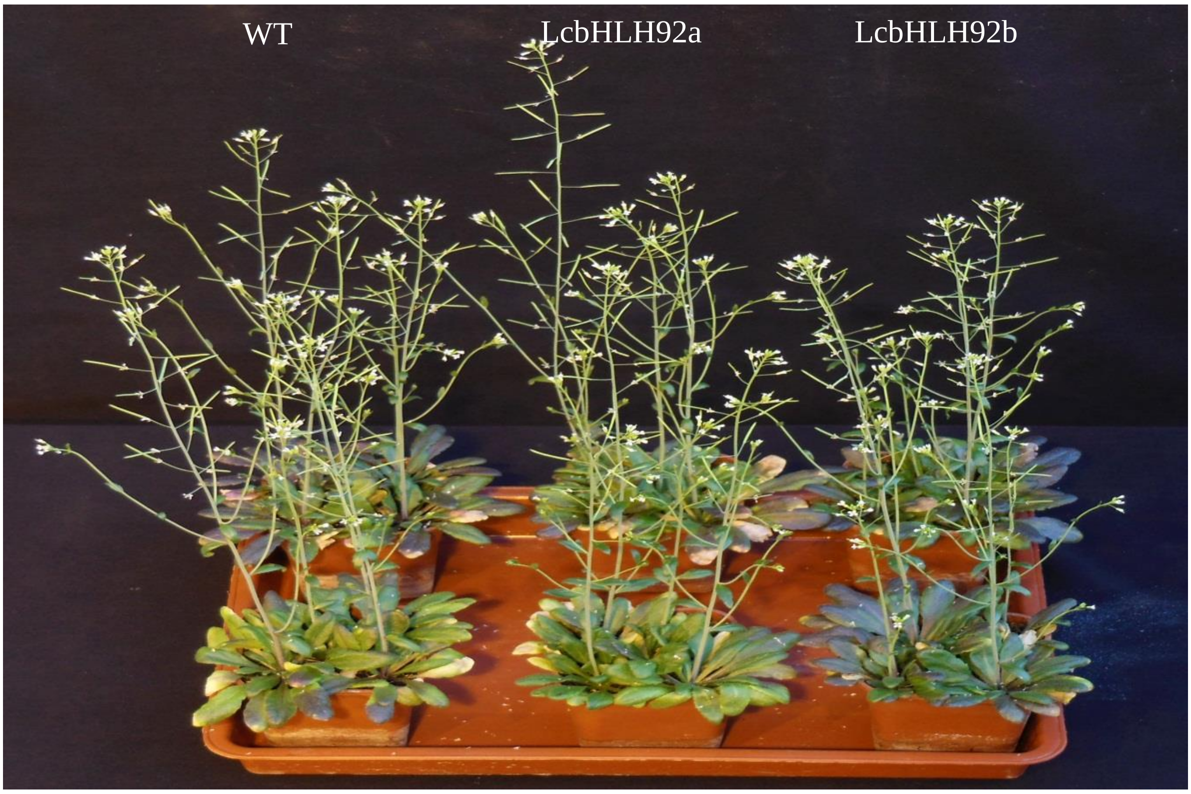

B

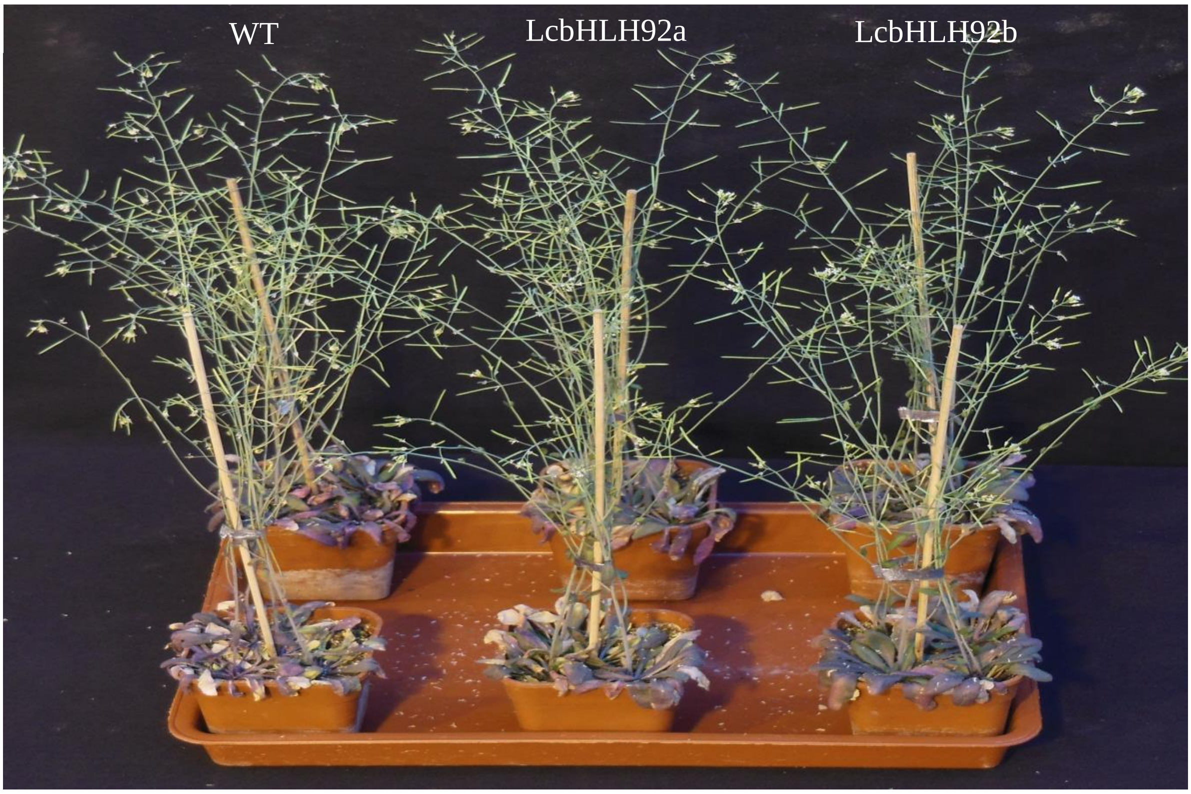

Figure S10. Natural drought stress assay of transgenic lines at flowering stage

A: Control check

B: Drought stress (without watering) for 7 days

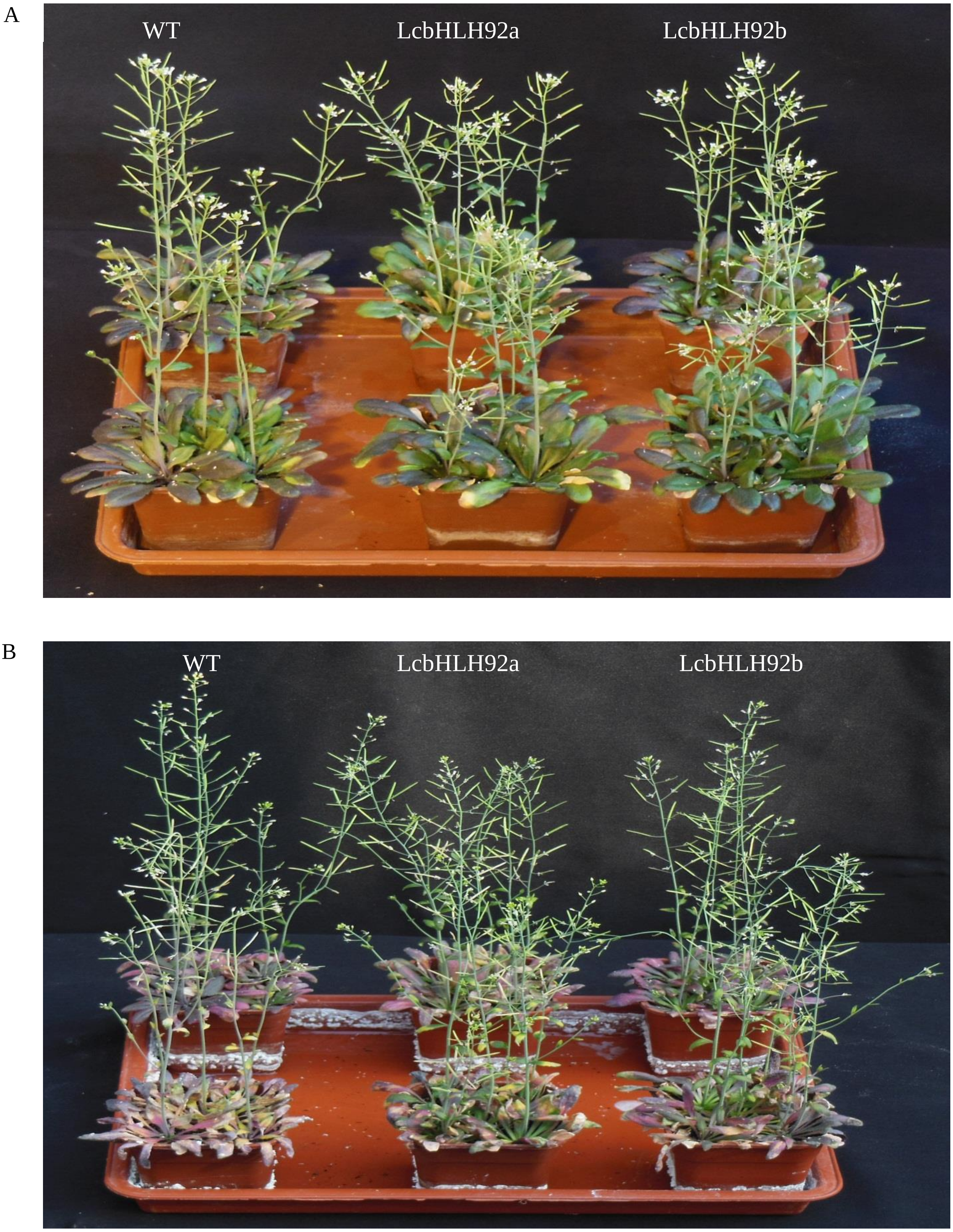

Figure S11. Osmotic tress assay of transgenic lines under 300mM mannitol treatment

A: Control check

B: Osmotic stress for 4 days
