# Supplementary material for: bHLH92 from sheepgrass acts as a negative regulator of anthocyanin/proanthocyandin accumulation and influences seed dormancy
Source: J Exp Bot. 2018 Sep 18;70(1):269–84. doi: 10.1093/jxb/ery335 (PMC6354636; doi:10.1093/jxb/ery335)
Supplement: Supplementary Tables S1-S6 [file ery335_suppl_supplementary_tables_s1-s6.pdf]

# *LcbHLH92* from sheepgrass acts as a negative regulator of anthocyanin/proanthocyandin accumulation and influences seed dormancy

Pincang Zhao[1, 2, #], Xiaoxia Li[1, #], Junting Jia[1], Guangxiao Yuan[1], Shuangyan Chen[1], Dongmei Qi[1], Liqin Cheng[1, *], Gongshe Liu[1, *]

1. Key Laboratory of Plant Resources, Institute of Botany, Chinese Academy of Sciences, Beijing, China
2. College of management science and engineering, Hebei University of Economics and Business, China

Table legends:

Table S1 Defferentially expressed genes (DEGs)

Table S2 Putative transcription factors from DEGs

Table S3 Correlation analysis of *TF*s with *ANR* and *ANS* at transcript level

Table S4 Selected transcription factors

Table S5 Primer sequences used in the present study

Table S6 Promoter sequence of some JAZs genes

# Table S1: Defferentially expressed genes(DEGs)

| Gene | fpkm_Y_14_1 | fpkm_Y_14_2 | fpkm_B_14_1 | fpkm_B_14_2 | fpkm_Y_28_1 | fpkm_Y_28_2 | fpkm_B_28_1 | fpkm_B_28_2 | log2FC(B14/Y14) | Padj | log2FC(B28/Y28) | Padj | Gene Length |
|---|---|---|---|---|---|---|---|---|---|---|---|---|---|
| Cluster-40555.190242 | 94.54 | 67.84 | 0 | 0.09 | 22.91 | 16.74 | 0 | 0 | -11.19 | 9.45E-49 | 0.00 | 1.77E-28 | 909 |
| Cluster-40555.46607 | 45.83 | 54.98 | 0 | 0.04 | 29.74 | 37.27 | 0 | 0 | -11.07 | 4.44E-45 | 0.00 | 1.84E-34 | 1211 |
| Cluster-40555.137723 | 174.61 | 171.35 | 0.17 | 0 | 3.84 | 2.82 | 0.14 | 0 | -10.92 | 2.08E-85 | -5.56 | 1.62E-02 | 634 |
| Cluster-40555.95265 | 130.71 | 151.86 | 0.15 | 0 | 1.06 | 1 | 0.04 | 0.08 | -10.82 | 1.87E-74 | -4.01 | 3.89E-02 | 1535 |
| Cluster-40555.184261 | 188.74 | 216.23 | 0 | 0.26 | 2.79 | 3.22 | 0.31 | 0 | -10.65 | 8.65E-68 | -4.34 | 1.23E-02 | 793 |
| Cluster-40555.192135 | 129.96 | 117.54 | 0.18 | 0 | 147.38 | 157.91 | 0 | 0 | -10.23 | 3.54E-158 | 0.00 | 1.10E-130 | 2515 |
| Cluster-40555.42955 | 25.46 | 24.98 | 0.05 | 0 | 2.31 | 1.68 | 0 | 0.08 | -9.98 | 3.09E-55 | -5.55 | 2.02E-05 | 1587 |
| Cluster-40555.304048 | 90.57 | 103.29 | 0.18 | 0 | 16.27 | 20.53 | 0 | 0 | -9.93 | 1.92E-49 | 0.00 | 3.84E-15 | 594 |
| Cluster-40555.302884 | 182.13 | 145.82 | 0.2 | 0.13 | 119.16 | 104.55 | 0.49 | 0.34 | -9.85 | 1.23E-72 | -8.01 | 4.25E-49 | 576 |
| Cluster-40555.192086 | 46.45 | 51.97 | 0 | 0.11 | 18.38 | 21.57 | 0.49 | 0 | -9.64 | 1.71E-73 | -6.39 | 1.57E-32 | 1344 |
| Cluster-40555.273142 | 85.4 | 85.51 | 0.19 | 0 | 3.61 | 3.16 | 0.33 | 0.18 | -9.53 | 1.61E-89 | -3.60 | 7.55E-04 | 1122 |
| Cluster-40555.160257 | 50.51 | 64.04 | 0.09 | 0.06 | 18.88 | 19.36 | 0 | 0.23 | -9.48 | 5.27E-30 | -7.29 | 1.04E-25 | 949 |
| Cluster-40555.186703 | 66.72 | 75.43 | 0 | 0.16 | 31.12 | 32.28 | 0.1 | 0 | -9.46 | 1.70E-62 | -9.67 | 3.71E-39 | 926 |
| Cluster-40555.184480 | 46.27 | 92.08 | 0 | 0.17 | 20.23 | 19.71 | 0 | 0.19 | -9.40 | 5.24E-07 | -7.59 | 1.11E-36 | 1337 |
| Cluster-40555.46485 | 219.19 | 185.62 | 0.33 | 0.32 | 4.42 | 6.23 | 0.14 | 0.14 | -9.15 | 7.07E-89 | -5.21 | 6.73E-04 | 639 |
| Cluster-40555.197461 | 64.48 | 93.53 | 0 | 0.25 | 5.63 | 7.19 | 0.82 | 0 | -9.12 | 5.14E-19 | -3.95 | 1.11E-10 | 2296 |
| Cluster-40555.195154 | 35.67 | 29.61 | 0 | 0.1 | 5.91 | 4.01 | 0.26 | 0.26 | -9.12 | 3.91E-57 | -4.13 | 3.83E-08 | 1407 |
| Cluster-40555.46394 | 26.38 | 17.57 | 0.05 | 0.03 | 4.58 | 3.31 | 0.04 | 0 | -9.03 | 9.00E-26 | -7.67 | 2.24E-12 | 1596 |
| Cluster-40555.188679 | 12.56 | 10.96 | 0 | 0.05 | 1.84 | 1.81 | 0 | 0 | -8.88 | 3.09E-37 | 0.00 | 1.60E-08 | 2055 |
| Cluster-40555.191383 | 345.07 | 281.58 | 1.44 | 0 | 285.55 | 208.65 | 2.79 | 14.69 | -8.67 | 4.88E-136 | -4.73 | 5.68E-28 | 956 |
| Cluster-40555.198090 | 266.98 | 237.07 | 1.18 | 0 | 61.9 | 47.25 | 1.34 | 5.34 | -8.60 | 1.90E-127 | -3.94 | 1.51E-25 | 977 |
| Cluster-40555.189052 | 138.19 | 225.76 | 0.29 | 0.57 | 16.74 | 18.88 | 0.6 | 0.56 | -8.55 | 6.98E-12 | -4.91 | 5.43E-31 | 2057 |
| Cluster-40555.203552 | 23.7 | 18.61 | 0.16 | 0 | 5.26 | 7.22 | 0 | 0.15 | -8.54 | 5.28E-24 | -6.03 | 9.19E-08 | 847 |
| Cluster-40555.158104 | 23.41 | 23.61 | 0.16 | 0 | 10.22 | 8.71 | 0 | 0.12 | -8.53 | 5.03E-24 | -7.46 | 1.60E-11 | 784 |
| Cluster-40555.166464 | 29.2 | 19.68 | 0 | 0.1 | 15.86 | 16.99 | 0.39 | 0.23 | -8.53 | 3.41E-24 | -5.72 | 2.98E-19 | 945 |
| Cluster-40555.302720 | 150.95 | 142.4 | 0.51 | 0.23 | 330.79 | 301.51 | 0.18 | 0.24 | -8.51 | 4.40E-109 | -10.48 | 5.19E-114 | 1115 |
| Cluster-40555.200743 | 69.33 | 66.17 | 0 | 0.33 | 16.2 | 10.64 | 0.1 | 0 | -8.47 | 1.12E-57 | -8.23 | 2.91E-16 | 846 |
| Cluster-40555.243392 | 145.87 | 149.66 | 0.78 | 0 | 55 | 57.1 | 0 | 0 | -8.45 | 9.55E-23 | 0.00 | 2.28E-10 | 337 |
| Cluster-40555.137927 | 38.72 | 42.51 | 0.22 | 0 | 2.36 | 2.36 | 0 | 0.56 | -8.43 | 9.74E-75 | -3.00 | 1.38E-03 | 1670 |

| | | | | | | | | | | | | | |
|---|---|---|---|---|---|---|---|---|---|---|---|---|---|
| Cluster-40555.181778 | 58.89 | 80.75 | 0 | 0.38 | 28.66 | 32.09 | 0 | 0 | -8.40 | 7.41E-21 | 0.00 | 1.53E-53 | 1340 |
| Cluster-40555.165028 | 9.91 | 13.91 | 0 | 0.07 | 2.94 | 2.72 | 0.18 | 0 | -8.26 | 5.35E-16 | -4.98 | 1.32E-08 | 1983 |
| Cluster-40555.220853 | 34.64 | 38.11 | 0.13 | 0.09 | 0.38 | 0.49 | 0.05 | 0.09 | -8.25 | 5.80E-112 | -2.64 | 2.24E-02 | 6030 |
| Cluster-40555.225333 | 6.98 | 3.88 | 0.05 | 0 | 0.37 | 0.68 | 0 | 0 | -8.18 | 8.66E-10 | 0.00 | 2.48E-02 | 2057 |
| Cluster-40555.211220 | 13.79 | 13.2 | 0 | 0.09 | 51.27 | 32.99 | 0.59 | 0 | -8.08 | 9.38E-32 | -7.09 | 2.65E-19 | 1604 |
| Cluster-40555.152937 | 9.26 | 8.22 | 0 | 0.06 | 1.73 | 1.57 | 0.05 | 0 | -8.04 | 4.13E-22 | -6.43 | 5.31E-05 | 1616 |
| Cluster-40555.231692 | 11.23 | 7.8 | 0 | 0.07 | 3.83 | 2.78 | 0.26 | 0.36 | -8.02 | 1.73E-11 | -3.42 | 1.12E-02 | 877 |
| Cluster-40555.217026 | 298.98 | 232.34 | 1.32 | 0.65 | 5.72 | 8.01 | 0.32 | 0.46 | -7.94 | 2.04E-99 | -4.08 | 2.10E-07 | 1057 |
| Cluster-40555.44266 | 11.4 | 16.07 | 0 | 0.1 | 61.71 | 31.38 | 0.39 | 0 | -7.94 | 7.90E-11 | -7.86 | 8.05E-08 | 658 |
| Cluster-40555.165745 | 91.33 | 114.31 | 0 | 0.77 | 9.45 | 9.67 | 1.21 | 2.52 | -7.92 | 4.07E-33 | -2.29 | 1.00E-05 | 1361 |
| Cluster-40555.179912 | 51.69 | 45.93 | 0.33 | 0 | 11.95 | 11.07 | 0.53 | 0.27 | -7.89 | 6.74E-29 | -4.77 | 2.05E-06 | 585 |
| Cluster-40555.166909 | 20.62 | 26.75 | 0 | 0.21 | 6.26 | 4.54 | 0 | 0.29 | -7.88 | 3.12E-20 | -4.99 | 1.26E-06 | 926 |
| Cluster-40555.46023 | 157.92 | 110.82 | 0.58 | 0.5 | 241.48 | 187.19 | 0.32 | 0.84 | -7.82 | 2.00E-39 | -8.45 | 1.59E-68 | 582 |
| Cluster-40555.238204 | 53.51 | 46.51 | 0 | 0.43 | 10.69 | 13.47 | 0.72 | 0.38 | -7.71 | 1.14E-67 | -4.43 | 1.53E-15 | 1387 |
| Cluster-40555.44009 | 2.48 | 2.23 | 0.02 | 0 | 1.52 | 1.67 | 0 | 0 | -7.56 | 1.77E-12 | 0.00 | 4.95E-11 | 2928 |
| Cluster-40555.227816 | 157.22 | 152.08 | 0.84 | 0.62 | 4.23 | 2.4 | 0.22 | 0 | -7.56 | 2.85E-95 | -4.89 | 2.99E-04 | 967 |
| Cluster-40555.187415 | 979.02 | 856.45 | 5.68 | 3.21 | 323.17 | 332.46 | 1.12 | 1.51 | -7.56 | 8.82E-143 | -7.89 | 4.09E-85 | 753 |
| Cluster-40555.237088 | 49.49 | 45.88 | 0 | 0.42 | 6.14 | 5.76 | 0.15 | 0.69 | -7.55 | 5.26E-44 | -3.72 | 5.95E-05 | 876 |
| Cluster-40555.45921 | 57.12 | 50.83 | 0.06 | 0.45 | 2.99 | 2.71 | 0.05 | 0.11 | -7.54 | 5.14E-64 | -5.06 | 3.27E-05 | 1225 |
| Cluster-40555.216634 | 54.53 | 82.46 | 0.42 | 0.27 | 28.63 | 27.96 | 0.35 | 1.46 | -7.52 | 1.42E-10 | -4.89 | 3.32E-13 | 557 |
| Cluster-40555.215536 | 11.48 | 8.91 | 0 | 0.11 | 4.42 | 4.37 | 0.69 | 0.52 | -7.51 | 2.66E-15 | -2.80 | 2.46E-03 | 1065 |
| Cluster-40555.144559 | 118.63 | 105.18 | 0.83 | 0.31 | 15.42 | 6.07 | 0 | 0 | -7.50 | 6.99E-80 | 0.00 | 2.82E-04 | 926 |
| Cluster-40555.304349 | 11.25 | 8.27 | 0 | 0.1 | 6.88 | 4.7 | 0 | 0 | -7.49 | 8.64E-08 | 0.00 | 4.28E-06 | 676 |
| Cluster-40555.181131 | 9.42 | 6.8 | 0 | 0.06 | 4.67 | 4.57 | 0.37 | 0.87 | -7.48 | 5.48E-15 | -2.87 | 2.43E-04 | 1253 |
| Cluster-40555.254739 | 11.84 | 8.39 | 0.12 | 0 | 8.71 | 8.57 | 0 | 0 | -7.47 | 1.11E-11 | 0.00 | 6.97E-13 | 849 |
| Cluster-40555.194398 | 121.21 | 76.03 | 0.35 | 0.63 | 62.46 | 55.24 | 0.77 | 0.3 | -7.46 | 3.06E-19 | -6.77 | 3.66E-42 | 803 |
| Cluster-40555.164105 | 43.23 | 38.8 | 0.12 | 0.33 | 3.89 | 4.72 | 0.44 | 0.4 | -7.45 | 5.39E-60 | -3.27 | 2.19E-05 | 1402 |
| Cluster-40555.45471 | 9.78 | 13.04 | 0 | 0.12 | 2.08 | 3.09 | 0.31 | 0 | -7.40 | 3.39E-14 | -4.04 | 1.22E-02 | 918 |
| Cluster-40555.43144 | 14.51 | 16.5 | 0.11 | 0.07 | 6.92 | 4.31 | 0.17 | 0.27 | -7.36 | 3.28E-17 | -4.58 | 1.29E-05 | 848 |
| Cluster-40555.174558 | 14.57 | 14.52 | 0.16 | 0 | 20.14 | 20.45 | 0.06 | 0 | -7.30 | 3.26E-35 | -9.15 | 2.26E-47 | 1700 |
| Cluster-40555.302905 | 1.53 | 1.6 | 0 | 0.02 | 0.98 | 0.51 | 0.12 | 0.05 | -7.30 | 8.84E-07 | -3.20 | 4.03E-02 | 2512 |
| Cluster-40555.45796 | 11.88 | 10.44 | 0 | 0.13 | 3.33 | 3.15 | 0 | 0 | -7.28 | 1.16E-06 | 0.00 | 2.00E-02 | 572 |

| | | | | | | | | | | | | | |
|---|---|---|---|---|---|---|---|---|---|---|---|---|---|
| Cluster-40555.304216 | 4.3 | 5.92 | 0.07 | 0 | 0.8 | 1.13 | 0 | 0 | -7.26 | 4.52E-10 | 0.00 | 2.21E-02 | 1251 |
| Cluster-40555.147435 | 10.08 | 8.91 | 0 | 0.15 | 8.63 | 4.61 | 0.29 | 0.21 | -7.24 | 1.77E-06 | -4.85 | 2.99E-04 | 617 |
| Cluster-40555.195994 | 5.68 | 4.88 | 0 | 0.07 | 4.73 | 5.15 | 0 | 0 | -7.23 | 2.96E-23 | 0.00 | 2.42E-28 | 2806 |
| Cluster-40555.44084 | 22.23 | 26.66 | 0 | 0.29 | 5.97 | 5.16 | 0.25 | 0 | -7.21 | 5.08E-18 | -5.43 | 1.78E-04 | 668 |
| Cluster-40555.210108 | 31.61 | 22.53 | 0 | 0.33 | 22.98 | 19.2 | 0 | 0 | -7.17 | 5.50E-12 | 0.00 | 1.46E-12 | 502 |
| Cluster-40555.158541 | 35.78 | 33.53 | 0.37 | 0.1 | 4.7 | 5.4 | 0.1 | 0 | -7.16 | 5.78E-30 | -6.62 | 9.67E-06 | 767 |
| Cluster-40555.230102 | 12.36 | 10.23 | 0 | 0.16 | 4.25 | 1.84 | 0 | 0 | -7.16 | 6.49E-12 | 0.00 | 2.00E-03 | 830 |
| Cluster-40555.205278 | 75.1 | 77.96 | 0.62 | 0.33 | 19.76 | 23.09 | 1.33 | 0 | -7.14 | 2.77E-83 | -4.99 | 2.46E-27 | 1405 |
| Cluster-40555.166106 | 6.12 | 5 | 0 | 0.04 | 8.33 | 10.54 | 0 | 0 | -7.07 | 1.04E-05 | 0.00 | 4.74E-13 | 806 |
| Cluster-40555.153051 | 7.28 | 6.22 | 0.09 | 0 | 4.05 | 3.34 | 0.08 | 0 | -7.05 | 1.33E-16 | -6.55 | 1.43E-10 | 1570 |
| Cluster-40555.40810 | 2.51 | 2.06 | 0 | 0.03 | 2.68 | 2.19 | 0 | 0 | -7.05 | 1.28E-05 | 0.00 | 3.03E-08 | 1566 |
| Cluster-40555.212039 | 116.04 | 196.12 | 1.29 | 0.92 | 13.14 | 13.11 | 0.54 | 0.5 | -7.03 | 2.13E-08 | -4.55 | 3.26E-17 | 1267 |
| Cluster-40555.248899 | 34.47 | 27.87 | 0 | 0.43 | 2.19 | 1.36 | 0 | 0.23 | -7.02 | 1.74E-41 | -3.89 | 1.32E-02 | 1194 |
| Cluster-40555.100438 | 34.18 | 52.51 | 0.38 | 0.25 | 101.82 | 81.22 | 1.32 | 0.35 | -6.98 | 2.02E-07 | -6.72 | 2.36E-25 | 421 |
| Cluster-40555.176194 | 9.71 | 8.89 | 0 | 0.13 | 16.82 | 10.69 | 1.6 | 0.17 | -6.97 | 2.46E-10 | -3.90 | 1.37E-07 | 864 |
| Cluster-40555.172349 | 2.45 | 3.02 | 0 | 0.03 | 6.31 | 3.92 | 0.05 | 0 | -6.91 | 5.62E-10 | -7.60 | 2.67E-13 | 2231 |
| Cluster-40555.186571 | 29.17 | 33.94 | 0.17 | 0.33 | 7.63 | 8.7 | 0 | 0 | -6.88 | 1.49E-20 | 0.00 | 8.62E-08 | 634 |
| Cluster-40555.153555 | 15.53 | 16.82 | 0.07 | 0.17 | 5.92 | 6.29 | 0.07 | 0.22 | -6.82 | 9.65E-20 | -5.28 | 3.87E-08 | 961 |
| Cluster-40555.175994 | 40.93 | 52.17 | 0.48 | 0.27 | 12.95 | 12.85 | 0.46 | 1.27 | -6.79 | 1.25E-26 | -3.80 | 5.69E-19 | 2000 |
| Cluster-40555.46501 | 6.01 | 5.86 | 0 | 0.1 | 2.98 | 3.02 | 0.24 | 0.5 | -6.79 | 4.66E-09 | -2.94 | 1.09E-02 | 1096 |
| Cluster-40555.132056 | 50.52 | 69.8 | 0 | 0.97 | 53.37 | 74.2 | 2.91 | 3.27 | -6.76 | 1.66E-04 | -4.38 | 9.79E-04 | 290 |
| Cluster-40555.305010 | 21.79 | 26.15 | 0.1 | 0.31 | 12.11 | 7.29 | 0.87 | 0.57 | -6.74 | 1.89E-25 | -3.69 | 6.54E-06 | 907 |
| Cluster-40555.157034 | 99.77 | 99.02 | 1.74 | 0 | 19.41 | 18.65 | 1.18 | 0.94 | -6.73 | 1.94E-29 | -4.10 | 6.54E-05 | 439 |
| Cluster-40555.148636 | 24.9 | 34.59 | 0.14 | 0.35 | 3.06 | 3.44 | 0.12 | 0 | -6.71 | 1.86E-12 | -5.76 | 6.07E-03 | 691 |
| Cluster-40555.45514 | 12.77 | 13.76 | 0 | 0.23 | 5.55 | 3.92 | 0.79 | 0.41 | -6.68 | 2.92E-12 | -2.93 | 1.37E-02 | 781 |
| Cluster-40555.174654 | 61.34 | 61.62 | 0.96 | 0.19 | 4.3 | 4.82 | 0.24 | 0 | -6.64 | 2.66E-63 | -5.32 | 8.02E-08 | 1162 |
| Cluster-40555.145957 | 4.65 | 2.41 | 0 | 0.06 | 1.73 | 1.08 | 0.11 | 0.08 | -6.64 | 4.89E-06 | -3.74 | 1.30E-02 | 1538 |
| Cluster-40555.44269 | 5.94 | 4.13 | 0.09 | 0 | 5.86 | 4.43 | 0.08 | 0.31 | -6.63 | 2.70E-06 | -4.61 | 7.69E-06 | 920 |
| Cluster-40555.158224 | 3.95 | 4.01 | 0 | 0.07 | 4.62 | 5.06 | 0.23 | 0.09 | -6.63 | 5.35E-08 | -4.87 | 3.32E-09 | 1371 |
| Cluster-40555.90296 | 10.89 | 11.75 | 0 | 0.21 | 5.06 | 5.83 | 0.27 | 0.28 | -6.61 | 6.74E-08 | -4.27 | 2.02E-03 | 647 |
| Cluster-40555.191986 | 4.18 | 3.24 | 0 | 0.06 | 2.98 | 4.42 | 0.54 | 0.4 | -6.60 | 3.94E-24 | -2.93 | 2.74E-04 | 4362 |
| Cluster-40555.213017 | 59.2 | 73.52 | 0.56 | 0.64 | 23.91 | 19.92 | 0.2 | 0.55 | -6.59 | 3.73E-26 | -5.92 | 1.11E-22 | 874 |

| | | | | | | | | | | | | | |
|---|---|---|---|---|---|---|---|---|---|---|---|---|---|
| Cluster-40555.40429 | 6.83 | 3.93 | 0 | 0.1 | 3.52 | 2.22 | 0 | 0.13 | -6.59 | 6.50E-04 | -5.35 | 2.40E-02 | 667 |
| Cluster-40555.101724 | 10.86 | 12.44 | 0.23 | 0 | 5.69 | 4.23 | 0.19 | 0.1 | -6.57 | 8.70E-12 | -5.05 | 4.98E-05 | 801 |
| Cluster-40555.131939 | 4.09 | 3.31 | 0.09 | 0 | 2.57 | 3.25 | 0.62 | 0.46 | -6.56 | 5.90E-06 | -2.43 | 4.80E-02 | 1105 |
| Cluster-40555.200353 | 16.97 | 19.04 | 0.35 | 0 | 7.63 | 6.85 | 0 | 0 | -6.56 | 2.53E-41 | 0.00 | 5.91E-26 | 1820 |
| Cluster-40555.305751 | 16.17 | 13.36 | 0.22 | 0.07 | 8.86 | 5.06 | 0.64 | 0.47 | -6.53 | 9.37E-15 | -3.58 | 1.71E-04 | 821 |
| Cluster-40555.110252 | 46.25 | 41.55 | 0.5 | 0.36 | 3.47 | 2.9 | 0.92 | 0.27 | -6.50 | 3.96E-59 | -2.38 | 7.87E-03 | 1458 |
| Cluster-80827.0 | 7.53 | 6.65 | 0 | 0.11 | 3.57 | 2.94 | 0.53 | 0 | -6.50 | 2.86E-07 | -3.57 | 1.63E-02 | 836 |
| Cluster-40555.125883 | 10.98 | 10.26 | 0.2 | 0 | 3.57 | 3.85 | 0 | 0 | -6.50 | 2.73E-25 | 0.00 | 2.04E-13 | 1677 |
| Cluster-40555.218526 | 4.22 | 4.64 | 0 | 0.1 | 10.26 | 10.15 | 0 | 0.29 | -6.49 | 3.39E-07 | -6.05 | 1.48E-17 | 1166 |
| Cluster-40555.40609 | 10.2 | 7.96 | 0 | 0.19 | 4.88 | 2.27 | 0.08 | 0.16 | -6.47 | 2.12E-10 | -4.82 | 4.14E-03 | 920 |
| Cluster-40555.305219 | 8.02 | 5.77 | 0.14 | 0 | 5.21 | 5.88 | 0 | 0 | -6.47 | 1.49E-05 | 0.00 | 5.07E-06 | 690 |
| Cluster-40555.308354 | 2.32 | 1.51 | 0 | 0.04 | 2.65 | 2.57 | 0.73 | 0.2 | -6.46 | 1.51E-03 | -2.44 | 3.18E-02 | 1298 |
| Cluster-40555.167379 | 8.57 | 8.36 | 0.08 | 0.07 | 3.88 | 1.91 | 0.09 | 0 | -6.45 | 1.25E-19 | -5.67 | 3.23E-04 | 1638 |
| Cluster-40555.186444 | 32.55 | 53.07 | 0.64 | 0.25 | 6.26 | 10.01 | 0.18 | 0 | -6.45 | 4.99E-08 | -6.68 | 8.73E-07 | 911 |
| Cluster-40555.189514 | 24.94 | 22.18 | 0 | 0.46 | 9.18 | 8.83 | 2.73 | 1.5 | -6.44 | 4.40E-31 | -2.04 | 5.95E-04 | 1171 |
| Cluster-40555.124490 | 94.83 | 108.08 | 0.67 | 1.51 | 26.24 | 19.67 | 2.79 | 1.36 | -6.43 | 1.39E-37 | -3.42 | 3.60E-07 | 554 |
| Cluster-40555.199319 | 47.58 | 28.35 | 0.83 | 0 | 15.2 | 12.53 | 0 | 0 | -6.42 | 3.21E-13 | 0.00 | 5.81E-30 | 1246 |
| Cluster-40555.173414 | 65.28 | 58.17 | 0 | 1.21 | 14.21 | 15.48 | 0 | 0 | -6.42 | 6.03E-16 | 0.00 | 1.07E-05 | 417 |
| Cluster-40555.151899 | 82.87 | 83.58 | 0.96 | 0.82 | 15.55 | 13.15 | 1.5 | 3.48 | -6.41 | 2.08E-61 | -2.46 | 3.75E-06 | 954 |
| Cluster-40555.228606 | 2.63 | 7.41 | 0 | 0.11 | 0.8 | 0.53 | 0 | 0 | -6.41 | 2.40E-02 | 0.00 | 8.49E-04 | 2495 |
| Cluster-40555.223088 | 16.42 | 20.82 | 0.24 | 0.16 | 5.25 | 6.5 | 0.71 | 0.84 | -6.41 | 2.12E-16 | -2.87 | 5.74E-03 | 766 |
| Cluster-40555.207191 | 10.2 | 11.95 | 0.15 | 0.09 | 19.19 | 14.96 | 0.24 | 0.63 | -6.40 | 1.33E-08 | -5.22 | 1.39E-12 | 685 |
| Cluster-40555.302653 | 25.02 | 20.98 | 0.08 | 0.4 | 6.52 | 4.35 | 0.15 | 0.37 | -6.40 | 2.97E-27 | -4.27 | 1.23E-06 | 1045 |
| Cluster-40555.221212 | 14.44 | 13.77 | 0.32 | 0 | 7.76 | 8.04 | 0.2 | 1.15 | -6.39 | 2.82E-40 | -3.45 | 1.27E-13 | 2236 |
| Cluster-40555.209761 | 3.93 | 3.42 | 0 | 0.08 | 1.22 | 0.84 | 0.12 | 0 | -6.39 | 2.44E-23 | -4.03 | 4.44E-06 | 4351 |
| Cluster-40555.135747 | 75.86 | 100.5 | 1.93 | 0 | 19.19 | 19.48 | 2.13 | 0 | -6.39 | 6.68E-20 | -4.15 | 1.78E-16 | 1044 |
| Cluster-40555.38561 | 7.78 | 10.88 | 0.09 | 0.11 | 2.14 | 2.81 | 0.07 | 0 | -6.38 | 8.38E-11 | -6.04 | 1.17E-03 | 952 |
| Cluster-40555.272518 | 6.48 | 7.9 | 0 | 0.15 | 27.34 | 27.47 | 0.1 | 0 | -6.38 | 1.30E-06 | -9.08 | 3.52E-29 | 777 |
| Cluster-40555.186143 | 86.83 | 117.8 | 2.1 | 0.25 | 19.74 | 27.71 | 0 | 0 | -6.32 | 1.70E-17 | 0.00 | 4.69E-18 | 1070 |
| Cluster-40555.54071 | 20.02 | 16.6 | 0 | 0.42 | 4.31 | 4.43 | 0.21 | 0 | -6.31 | 2.64E-17 | -5.61 | 3.60E-05 | 847 |
| Cluster-40555.171315 | 2.09 | 3.6 | 0.06 | 0 | 1.24 | 0.93 | 0.29 | 0.08 | -6.31 | 2.74E-06 | -2.52 | 1.60E-02 | 3013 |
| Cluster-40555.180075 | 164.93 | 163.65 | 2.11 | 1.7 | 57.12 | 48.33 | 0.88 | 0.46 | -6.29 | 2.24E-60 | -6.24 | 8.18E-29 | 612 |

| | | | | | | | | | | | | | |
|---|---|---|---|---|---|---|---|---|---|---|---|---|---|
| Cluster-40555.284887 | 17.98 | 18.31 | 0 | 0.38 | 6.24 | 5.72 | 0 | 0 | -6.29 | 4.71E-17 | 0.00 | 6.05E-09 | 840 |
| Cluster-40555.205648 | 9.87 | 18.99 | 0 | 0.32 | 33.71 | 42.78 | 0.4 | 0 | -6.28 | 1.16E-04 | -7.59 | 1.36E-29 | 1810 |
| Cluster-40555.193192 | 88.96 | 117.19 | 2.49 | 0 | 13.57 | 14.85 | 0 | 0.96 | -6.27 | 5.34E-22 | -4.78 | 4.61E-29 | 2293 |
| Cluster-40555.244247 | 30.81 | 27.58 | 0.69 | 0 | 18.69 | 17.18 | 0 | 0.32 | -6.27 | 1.92E-09 | -6.76 | 5.72E-07 | 438 |
| Cluster-40555.189703 | 173.64 | 201.76 | 2.34 | 2.1 | 154.37 | 166.87 | 1.82 | 1.1 | -6.26 | 7.47E-48 | -6.72 | 2.68E-75 | 1447 |
| Cluster-40555.217920 | 1.19 | 1.16 | 0 | 0.04 | 1.55 | 1.22 | 0 | 0.14 | -6.25 | 4.93E-03 | -4.17 | 2.68E-03 | 1704 |
| Cluster-40555.219881 | 27.46 | 25.97 | 0.25 | 0.4 | 2.75 | 2.75 | 0.18 | 0.66 | -6.24 | 3.41E-35 | -2.67 | 1.67E-02 | 1208 |
| Cluster-40555.247879 | 81.1 | 75.11 | 1.11 | 0.77 | 14 | 13.14 | 1.13 | 0.87 | -6.23 | 3.70E-59 | -3.70 | 1.00E-10 | 980 |
| Cluster-40555.165785 | 294.58 | 558.37 | 4.26 | 6.11 | 2.62 | 3.15 | 0 | 0 | -6.23 | 2.96E-05 | 0.00 | 1.60E-11 | 1812 |
| Cluster-40555.208774 | 5.93 | 3.67 | 0 | 0.12 | 1.56 | 2.2 | 0 | 0 | -6.22 | 2.91E-11 | 0.00 | 1.23E-08 | 2015 |
| Cluster-40555.227160 | 3.05 | 1.78 | 0 | 0.06 | 3.17 | 2.62 | 0 | 0.15 | -6.22 | 6.24E-03 | -5.17 | 5.68E-04 | 959 |
| Cluster-40555.27333 | 3.01 | 2.91 | 0 | 0.07 | 3.18 | 3.35 | 0.18 | 0.19 | -6.22 | 6.03E-03 | -4.09 | 6.43E-03 | 825 |
| Cluster-40555.8896 | 7.05 | 9 | 0.07 | 0.13 | 3.25 | 3.53 | 0.66 | 0.39 | -6.21 | 1.02E-12 | -2.63 | 9.00E-03 | 1184 |
| Cluster-40555.303069 | 4.14 | 5.29 | 0.17 | 0 | 5.48 | 3.98 | 0 | 0 | -6.21 | 1.38E-04 | 0.00 | 3.15E-06 | 779 |
| Cluster-40555.39926 | 8.7 | 5.97 | 0 | 0.18 | 8.56 | 8.03 | 0.38 | 0.23 | -6.20 | 2.02E-08 | -4.71 | 3.14E-09 | 939 |
| Cluster-40555.179540 | 561.64 | 353.56 | 0 | 11.08 | 175.39 | 106.87 | 29.82 | 49.6 | -6.19 | 2.54E-16 | -1.75 | 1.58E-02 | 772 |
| Cluster-40555.66926 | 9.82 | 8.39 | 0.21 | 0 | 2.74 | 1.67 | 0.44 | 0 | -6.17 | 9.92E-13 | -3.26 | 3.65E-02 | 1048 |
| Cluster-40555.42037 | 5.3 | 5.16 | 0 | 0.13 | 1.46 | 1.28 | 0.11 | 0 | -6.16 | 3.39E-08 | -4.62 | 2.72E-02 | 1180 |
| Cluster-40555.233666 | 6.14 | 5.43 | 0 | 0.15 | 1.7 | 2.52 | 0.18 | 0 | -6.15 | 1.62E-05 | -4.50 | 4.70E-02 | 813 |
| Cluster-40555.200150 | 255.37 | 295.85 | 3.14 | 4.03 | 51.48 | 51.59 | 3.74 | 5.44 | -6.12 | 8.13E-46 | -3.43 | 3.93E-21 | 1080 |
| Cluster-40555.197848 | 6.83 | 6.88 | 0 | 0.18 | 2.81 | 5.1 | 0.42 | 0.38 | -6.12 | 3.28E-17 | -3.25 | 1.74E-02 | 1852 |
| Cluster-40555.195189 | 6.75 | 5.9 | 0.16 | 0 | 3.81 | 2.22 | 0.22 | 0.1 | -6.11 | 2.51E-19 | -4.24 | 5.07E-06 | 2081 |
| Cluster-40555.198181 | 11.18 | 9.69 | 0.28 | 0 | 8.18 | 8.53 | 0 | 0 | -6.10 | 4.01E-36 | 0.00 | 9.21E-40 | 2673 |
| Cluster-40555.18892 | 2.64 | 2.34 | 0 | 0.06 | 2.32 | 1.35 | 0.16 | 0.04 | -6.10 | 2.67E-05 | -4.10 | 1.53E-03 | 1499 |
| Cluster-40555.190667 | 68.95 | 59.18 | 0 | 1.62 | 9.81 | 7.69 | 0.55 | 0 | -6.10 | 3.69E-44 | -5.07 | 2.92E-09 | 861 |
| Cluster-40555.165376 | 19.99 | 28.15 | 0.35 | 0.3 | 14.53 | 13.01 | 0.43 | 0.44 | -6.09 | 1.08E-13 | -4.92 | 6.88E-30 | 2383 |
| Cluster-40555.38726 | 3.87 | 5.66 | 0.08 | 0.05 | 3.29 | 3.52 | 0 | 0.33 | -6.08 | 9.76E-07 | -4.26 | 2.24E-04 | 1047 |
| Cluster-40555.168603 | 13.83 | 21.28 | 0.21 | 0.27 | 22.85 | 15.12 | 0.46 | 0 | -6.08 | 6.95E-07 | -6.14 | 2.34E-11 | 558 |
| Cluster-40555.88685 | 15.21 | 14.55 | 0.31 | 0.1 | 6.86 | 8.07 | 0 | 0.4 | -6.07 | 1.64E-10 | -5.15 | 9.95E-06 | 664 |
| Cluster-40555.44830 | 3.71 | 1.39 | 0 | 0.07 | 3.42 | 1.53 | 0 | 0.18 | -6.07 | 2.21E-02 | -4.68 | 1.67E-02 | 857 |
| Cluster-40555.86060 | 21.25 | 15.22 | 0.08 | 0.4 | 34.52 | 24.64 | 0.39 | 1.04 | -6.07 | 1.42E-21 | -5.26 | 4.93E-24 | 1048 |
| Cluster-40555.153068 | 6.33 | 4.83 | 0.17 | 0 | 3.65 | 3.3 | 0 | 0.12 | -6.06 | 2.67E-15 | -5.64 | 6.09E-11 | 1855 |

| | | | | | | | | | | | | | |
|---|---|---|---|---|---|---|---|---|---|---|---|---|---|
| Cluster-40555.155535 | 11.6 | 13.47 | 0.35 | 0 | 6.97 | 5.53 | 0 | 0 | -6.06 | 1.05E-18 | 0.00 | 5.64E-14 | 1141 |
| Cluster-40555.205766 | 2.41 | 1.47 | 0 | 0.04 | 3.34 | 1.81 | 0 | 0.12 | -6.06 | 1.52E-07 | -5.16 | 1.04E-05 | 2586 |
| Cluster-40555.161334 | 11.64 | 9.73 | 0.12 | 0.17 | 3.65 | 3.63 | 0.32 | 0.33 | -6.05 | 3.75E-31 | -3.45 | 3.56E-08 | 2344 |
| Cluster-40555.189792 | 61.06 | 57.88 | 0.8 | 0.81 | 7.29 | 3.55 | 0.2 | 0 | -6.05 | 6.15E-61 | -5.79 | 1.31E-04 | 1330 |
| Cluster-40555.91887 | 2.01 | 1.43 | 0 | 0.05 | 3.61 | 4.09 | 0.18 | 0.09 | -6.05 | 1.42E-02 | -4.63 | 1.12E-05 | 1117 |
| Cluster-40555.150241 | 0.66 | 0.52 | 0.02 | 0 | 0.67 | 0.73 | 0.04 | 0 | -6.04 | 5.38E-04 | -5.07 | 6.67E-05 | 3940 |
| Cluster-40555.193763 | 80.69 | 72.41 | 1.6 | 0.54 | 10.86 | 10.62 | 0.25 | 0.46 | -6.04 | 1.02E-73 | -4.79 | 5.71E-18 | 1454 |
| Cluster-40555.304739 | 8.09 | 8.01 | 0.04 | 0.19 | 3.16 | 3.87 | 0.03 | 0 | -6.02 | 1.06E-22 | -8.02 | 1.66E-15 | 2154 |
| Cluster-40555.172510 | 39.67 | 29.74 | 0.54 | 0.44 | 15.57 | 10.56 | 0.67 | 0.16 | -6.02 | 6.01E-30 | -4.90 | 1.08E-12 | 869 |
| Cluster-40555.197873 | 10.51 | 8.11 | 0 | 0.25 | 6.26 | 5.31 | 0 | 0 | -6.01 | 3.29E-32 | 0.00 | 9.81E-33 | 2906 |
| Cluster-40555.237054 | 10.38 | 13.6 | 0.21 | 0.15 | 9.39 | 9.73 | 0.17 | 0 | -6.01 | 1.14E-08 | -7.25 | 3.66E-09 | 678 |
| Cluster-40555.156004 | 12.49 | 7.84 | 0 | 0.29 | 5.23 | 4.05 | 0.16 | 0.22 | -6.00 | 6.58E-13 | -4.52 | 2.60E-13 | 2251 |
| Cluster-40555.247681 | 2.55 | 1.83 | 0 | 0.06 | 1.57 | 0.89 | 0.1 | 0.08 | -5.97 | 3.15E-09 | -3.72 | 2.22E-04 | 2840 |
| Cluster-40555.215483 | 6.54 | 4.89 | 0.16 | 0.01 | 11.05 | 9.83 | 0 | 0 | -5.96 | 7.14E-11 | 0.00 | 4.88E-26 | 1348 |
| Cluster-40555.304721 | 4 | 7.9 | 0 | 0.21 | 105.13 | 118.33 | 0 | 0 | -5.94 | 2.13E-02 | 0.00 | 4.71E-45 | 489 |
| Cluster-40555.139533 | 39.63 | 32.71 | 0.15 | 0.89 | 23.98 | 18.8 | 0.76 | 1.5 | -5.94 | 4.40E-36 | -4.16 | 2.95E-18 | 1060 |
| Cluster-40555.245835 | 723.05 | 746.56 | 9.54 | 11.56 | 39.43 | 32.79 | 4.51 | 2.53 | -5.93 | 7.93E-27 | -3.33 | 4.31E-02 | 304 |
| Cluster-40555.176446 | 5.75 | 7.82 | 0.2 | 0 | 2.93 | 4.35 | 0.52 | 0.4 | -5.93 | 3.48E-14 | -2.94 | 4.10E-04 | 2040 |
| Cluster-40555.204278 | 97.95 | 99.31 | 1.39 | 1.53 | 2.71 | 2.03 | 0.09 | 0.36 | -5.93 | 2.00E-76 | -3.29 | 2.51E-03 | 1400 |
| Cluster-40555.88317 | 1.39 | 1.11 | 0.02 | 0.01 | 1.36 | 1.56 | 0.29 | 0.25 | -5.92 | 6.84E-06 | -2.36 | 6.55E-03 | 2969 |
| Cluster-40555.264155 | 11.96 | 9.82 | 0.1 | 0.13 | 6.6 | 6.8 | 0.17 | 1.18 | -5.92 | 6.94E-06 | -3.21 | 1.57E-02 | 571 |
| Cluster-40555.177674 | 23.53 | 8.92 | 0.37 | 0 | 31.16 | 31.21 | 3.29 | 1.52 | -5.92 | 4.23E-03 | -3.66 | 8.42E-09 | 515 |
| Cluster-40555.234831 | 9.19 | 8.69 | 0.27 | 0 | 4.2 | 3.46 | 0.08 | 0.08 | -5.91 | 1.43E-10 | -5.57 | 2.91E-05 | 934 |
| Cluster-40555.176727 | 123.2 | 127.49 | 1.59 | 2.04 | 49.31 | 50.43 | 0 | 0 | -5.91 | 5.34E-08 | 0.00 | 3.74E-04 | 287 |
| Cluster-40555.305499 | 3.17 | 3.6 | 0 | 0.1 | 4.89 | 4.11 | 0.38 | 0.26 | -5.91 | 1.53E-04 | -3.73 | 8.33E-05 | 1053 |
| Cluster-40555.193802 | 5.33 | 6.26 | 0 | 0.14 | 14.14 | 9.36 | 0 | 0 | -5.90 | 1.54E-04 | 0.00 | 1.55E-13 | 721 |
| Cluster-40555.119956 | 4.06 | 2.5 | 0 | 0.11 | 1.74 | 1.83 | 0 | 0.13 | -5.89 | 1.82E-04 | -4.69 | 1.13E-02 | 1076 |
| Cluster-40555.305184 | 1.11 | 0.77 | 0 | 0.03 | 0.94 | 0.49 | 0 | 0 | -5.89 | 2.82E-02 | 0.00 | 1.60E-02 | 1697 |
| Cluster-40555.190564 | 26.66 | 27.79 | 0 | 0.83 | 7.09 | 6 | 0.18 | 0 | -5.88 | 1.47E-34 | -6.01 | 3.33E-13 | 1280 |
| Cluster-40555.186338 | 21.26 | 21.23 | 0.39 | 0.27 | 3.96 | 3.65 | 0.42 | 0.61 | -5.87 | 1.03E-52 | -2.80 | 5.81E-07 | 2624 |
| Cluster-40555.169879 | 25.82 | 30.35 | 0.17 | 0.71 | 2.93 | 1.68 | 0 | 0.14 | -5.86 | 2.10E-28 | -4.91 | 3.11E-03 | 999 |
| Cluster-40555.199348 | 6.3 | 8.55 | 0 | 0.24 | 14.73 | 19.69 | 2.23 | 1.34 | -5.86 | 3.04E-02 | -3.29 | 2.16E-03 | 434 |

| | | | | | | | | | | | | | |
|---|---|---|---|---|---|---|---|---|---|---|---|---|---|
| Cluster-40555.204499 | 8.45 | 7.67 | 0.2 | 0.06 | 6.11 | 5.94 | 0.1 | 0 | -5.84 | 1.88E-16 | -7.20 | 1.46E-16 | 1521 |
| Cluster-40555.203796 | 2.69 | 4.12 | 0 | 0.13 | 4.85 | 3.65 | 0 | 0.14 | -5.84 | 3.35E-02 | -5.79 | 2.50E-03 | 632 |
| Cluster-40555.302636 | 9.13 | 8.58 | 0.22 | 0.07 | 1.57 | 2.43 | 0 | 0.09 | -5.83 | 9.73E-09 | -5.36 | 2.49E-02 | 834 |
| Cluster-40555.186726 | 286.69 | 319.85 | 7.9 | 1.89 | 38.95 | 52.35 | 6.77 | 0.53 | -5.83 | 1.80E-62 | -3.62 | 1.47E-07 | 993 |
| Cluster-40555.136612 | 2.85 | 2.6 | 0.09 | 0 | 1.77 | 3.19 | 0.22 | 0.3 | -5.83 | 2.10E-03 | -3.20 | 3.92E-02 | 955 |
| Cluster-40555.209648 | 14.55 | 23.95 | 0 | 0.61 | 8 | 16.26 | 0.41 | 0.28 | -5.83 | 9.00E-07 | -5.08 | 2.94E-03 | 2814 |
| Cluster-40555.291897 | 3.93 | 6.73 | 0 | 0.17 | 8.4 | 8.29 | 0.16 | 0.68 | -5.82 | 3.00E-04 | -4.32 | 3.39E-06 | 730 |
| Cluster-40555.312228 | 2.47 | 2.1 | 0.09 | 0 | 1.15 | 1.03 | 0 | 0 | -5.82 | 2.30E-03 | 0.00 | 3.56E-02 | 1080 |
| Cluster-40555.41735 | 2.29 | 3.2 | 0.09 | 0 | 3.41 | 2.86 | 0.08 | 0.31 | -5.81 | 2.24E-03 | -3.94 | 3.08E-03 | 937 |
| Cluster-40555.129105 | 9.85 | 13.3 | 0 | 0.37 | 17.03 | 13.73 | 1 | 0.55 | -5.81 | 3.68E-02 | -4.26 | 1.43E-02 | 368 |
| Cluster-40555.44758 | 95.59 | 88.53 | 2.1 | 0.94 | 21.42 | 13.93 | 1.62 | 1.54 | -5.79 | 3.66E-42 | -3.41 | 2.90E-07 | 642 |
| Cluster-40555.207642 | 2.75 | 4.33 | 0.13 | 0 | 47.16 | 53.28 | 0 | 0 | -5.79 | 9.15E-08 | 0.00 | 6.81E-97 | 2826 |
| Cluster-40555.305045 | 6.76 | 3.79 | 0.08 | 0.08 | 1.95 | 1.91 | 0.06 | 0.19 | -5.78 | 4.15E-07 | -3.86 | 1.95E-02 | 1081 |
| Cluster-40555.303392 | 4.19 | 3.89 | 0.14 | 0 | 7.18 | 8.33 | 0.57 | 0.82 | -5.78 | 2.87E-03 | -3.41 | 3.17E-04 | 713 |
| Cluster-40555.43034 | 4.93 | 4.68 | 0.1 | 0.06 | 1.73 | 1.47 | 0 | 0.24 | -5.78 | 3.43E-10 | -3.60 | 7.02E-03 | 1531 |
| Cluster-40555.45021 | 12.82 | 12.62 | 0.17 | 0.25 | 8.94 | 5.54 | 0.25 | 0.21 | -5.78 | 7.33E-21 | -4.90 | 4.06E-09 | 1365 |
| Cluster-40555.202117 | 71.28 | 74.75 | 1.33 | 1.19 | 14.8 | 15.5 | 1.36 | 1.39 | -5.77 | 1.11E-41 | -3.37 | 1.23E-07 | 744 |
| Cluster-40555.222938 | 11.82 | 9.71 | 0 | 0.35 | 4.94 | 5.6 | 0.97 | 0.15 | -5.76 | 1.13E-20 | -3.19 | 7.07E-07 | 1616 |
| Cluster-40555.236667 | 6.86 | 3.47 | 0.1 | 0.07 | 10.02 | 7.76 | 0.11 | 0 | -5.76 | 7.08E-05 | -7.65 | 3.60E-12 | 852 |
| Cluster-40555.174865 | 16.2 | 12.6 | 0.09 | 0.42 | 12.26 | 17.62 | 0.37 | 1.37 | -5.76 | 3.49E-17 | -4.02 | 5.47E-07 | 1064 |
| Cluster-40555.295852 | 23.44 | 20.17 | 0.27 | 0.48 | 16.15 | 16.24 | 0.86 | 1.12 | -5.74 | 8.10E-48 | -3.96 | 3.20E-24 | 2373 |
| Cluster-40555.107085 | 1.65 | 1.71 | 0.06 | 0 | 1.87 | 2.3 | 0 | 0 | -5.74 | 3.56E-03 | 0.00 | 1.20E-05 | 1315 |
| Cluster-40555.171785 | 3.22 | 2.74 | 0 | 0.1 | 1.24 | 1.27 | 0 | 0 | -5.73 | 6.38E-04 | 0.00 | 1.70E-02 | 1062 |
| Cluster-40555.241912 | 70 | 31.88 | 0.4 | 1.28 | 29.54 | 37.47 | 6.49 | 6.94 | -5.73 | 9.67E-05 | -2.27 | 2.91E-03 | 416 |
| Cluster-40555.146730 | 5.14 | 5.86 | 0.11 | 0.07 | 1.24 | 1.95 | 0 | 0 | -5.72 | 3.56E-18 | 0.00 | 1.54E-07 | 2391 |
| Cluster-40555.45051 | 57.27 | 49.07 | 1 | 0.87 | 23.3 | 12.98 | 1.06 | 1.25 | -5.70 | 1.53E-36 | -3.89 | 6.34E-05 | 818 |
| Cluster-40555.174535 | 10.7 | 20.05 | 0 | 0.54 | 12.58 | 12.47 | 0.24 | 0 | -5.68 | 1.78E-03 | -6.69 | 4.38E-06 | 482 |
| Cluster-40555.307716 | 1.01 | 0.96 | 0.04 | 0 | 12.27 | 12.73 | 0 | 0 | -5.67 | 5.26E-03 | 0.00 | 2.53E-41 | 1972 |
| Cluster-40555.195239 | 26.49 | 26.12 | 0.41 | 0.53 | 6.53 | 4.31 | 0.11 | 0.24 | -5.66 | 7.70E-18 | -4.88 | 1.75E-04 | 712 |
| Cluster-40555.224750 | 2.45 | 2.76 | 0.1 | 0 | 1.36 | 1.59 | 0 | 0.03 | -5.66 | 2.09E-11 | -7.10 | 5.94E-09 | 2833 |
| Cluster-40555.201242 | 19.05 | 18.19 | 0.32 | 0.34 | 5.68 | 7.01 | 1.73 | 1.51 | -5.65 | 3.09E-51 | -1.91 | 1.29E-05 | 3062 |
| Cluster-40555.210151 | 2.88 | 2.58 | 0 | 0.09 | 8.05 | 7.27 | 0.5 | 1.59 | -5.65 | 7.98E-09 | -2.79 | 5.32E-10 | 2327 |

| | | | | | | | | | | | | | |
|---|---|---|---|---|---|---|---|---|---|---|---|---|---|
| Cluster-40555.239830 | 5.73 | 5.44 | 0.22 | 0 | 1.6 | 1.3 | 0 | 0 | -5.64 | 1.40E-08 | 0.00 | 3.04E-03 | 1155 |
| Cluster-40555.192719 | 33.11 | 20.5 | 0.3 | 0.67 | 6.38 | 6.61 | 0.71 | 0.97 | -5.64 | 2.54E-12 | -2.90 | 6.09E-07 | 1556 |
| Cluster-40555.95930 | 9.45 | 10.17 | 0.07 | 0.28 | 4.28 | 6.69 | 0.91 | 0.08 | -5.62 | 1.15E-12 | -3.48 | 1.11E-03 | 1100 |
| Cluster-40555.254050 | 10.82 | 8.65 | 0 | 0.36 | 5.45 | 7.75 | 0 | 0 | -5.62 | 1.38E-03 | 0.00 | 1.16E-03 | 488 |
| Cluster-40555.304868 | 4.22 | 3.74 | 0 | 0.14 | 2.61 | 3.6 | 0.28 | 0.1 | -5.62 | 1.40E-03 | -4.03 | 1.09E-02 | 815 |
| Cluster-40555.165499 | 31.94 | 32.22 | 1.14 | 0 | 28.6 | 17.16 | 0 | 1.06 | -5.62 | 1.08E-05 | -5.25 | 2.78E-04 | 362 |
| Cluster-40555.45048 | 2.05 | 2.15 | 0 | 0.09 | 1.23 | 1.04 | 0 | 0.23 | -5.61 | 2.80E-05 | -3.18 | 3.14E-02 | 1821 |
| Cluster-40555.161462 | 171.26 | 184.29 | 4.7 | 2.08 | 37.23 | 41.06 | 5.79 | 5.43 | -5.59 | 1.41E-64 | -2.74 | 3.86E-10 | 720 |
| Cluster-40555.174147 | 9.74 | 9.94 | 0.19 | 0.25 | 1.78 | 2.89 | 0 | 0 | -5.58 | 3.60E-06 | 0.00 | 3.88E-02 | 640 |
| Cluster-40555.196255 | 7.49 | 11.35 | 0 | 0.34 | 6.33 | 4.33 | 0 | 0.9 | -5.58 | 8.59E-08 | -3.42 | 1.83E-07 | 1500 |
| Cluster-40555.187033 | 13.07 | 11.34 | 0 | 0.46 | 12.53 | 12.68 | 0.25 | 0.24 | -5.55 | 2.43E-35 | -5.62 | 1.79E-36 | 2852 |
| Cluster-40555.143469 | 21.24 | 17.64 | 0.46 | 0.29 | 72.85 | 61.12 | 0.96 | 1.01 | -5.55 | 1.61E-08 | -6.03 | 1.65E-27 | 531 |
| Cluster-40555.50336 | 1.55 | 3.43 | 0 | 0.09 | 0.73 | 1.68 | 0 | 0 | -5.54 | 1.20E-02 | 0.00 | 1.51E-02 | 1501 |
| Cluster-40555.256112 | 2.29 | 2.17 | 0.07 | 0.03 | 1.32 | 1.91 | 0.21 | 0.31 | -5.53 | 8.16E-11 | -2.55 | 9.31E-04 | 3257 |
| Cluster-40555.200433 | 113.15 | 68.85 | 2.11 | 1.29 | 38.57 | 39 | 0.41 | 0.36 | -5.53 | 6.63E-14 | -6.78 | 5.76E-13 | 422 |
| Cluster-40555.161992 | 20.92 | 22.53 | 0.72 | 0.15 | 32.32 | 29.68 | 1.01 | 0.21 | -5.52 | 2.60E-09 | -5.62 | 3.08E-14 | 519 |
| Cluster-40555.195220 | 100.66 | 133.72 | 1.71 | 2.97 | 25.44 | 22.47 | 4.26 | 2.89 | -5.51 | 1.90E-16 | -2.68 | 2.76E-14 | 2082 |
| Cluster-40555.142360 | 65.22 | 83.78 | 1.38 | 1.61 | 52.29 | 73.53 | 11.99 | 7.19 | -5.50 | 3.35E-14 | -2.67 | 1.75E-04 | 483 |
| Cluster-40555.190982 | 181.53 | 152.31 | 1.7 | 5.08 | 59.21 | 58.96 | 0.72 | 3.54 | -5.49 | 1.93E-31 | -4.74 | 1.30E-13 | 419 |
| Cluster-40555.193706 | 19.33 | 17.28 | 0.1 | 0.61 | 6.95 | 6.26 | 1.57 | 0 | -5.49 | 1.88E-42 | -3.04 | 1.10E-10 | 2571 |
| Cluster-40555.234751 | 36.76 | 29.03 | 0.35 | 0.94 | 12.97 | 9.97 | 1.34 | 1.15 | -5.49 | 2.60E-41 | -3.13 | 7.82E-11 | 1470 |
| Cluster-40555.44524 | 0.79 | 1.34 | 0 | 0.05 | 0.52 | 0.69 | 0 | 0 | -5.49 | 2.99E-03 | 0.00 | 8.01E-03 | 2113 |
| Cluster-40555.148791 | 87.47 | 82.86 | 1.57 | 1.86 | 607.52 | 587.59 | 0.79 | 0.52 | -5.49 | 3.52E-53 | -9.77 | 1.68E-119 | 966 |
| Cluster-40555.176550 | 43.28 | 33.66 | 0.27 | 1.24 | 11.55 | 11.27 | 3.19 | 2.62 | -5.48 | 1.59E-22 | -1.90 | 1.48E-02 | 704 |
| Cluster-40555.195583 | 8.94 | 14.99 | 0.08 | 0.41 | 3.53 | 4.67 | 0.68 | 0.06 | -5.48 | 7.55E-06 | -3.44 | 4.72E-07 | 2239 |
| Cluster-40555.45616 | 5.46 | 3.87 | 0.07 | 0.12 | 1.2 | 0.73 | 0 | 0.03 | -5.47 | 1.39E-14 | -6.21 | 1.31E-04 | 2397 |
| Cluster-40555.218108 | 4.44 | 7.75 | 0.13 | 0.13 | 4.78 | 6.31 | 0 | 0.31 | -5.47 | 2.87E-04 | -5.21 | 5.15E-06 | 821 |
| Cluster-40555.142946 | 147.94 | 147.57 | 2.73 | 3.35 | 5.84 | 3.43 | 0.48 | 1.36 | -5.47 | 1.07E-69 | -2.26 | 1.27E-02 | 1034 |
| Cluster-40555.286735 | 17.11 | 25.03 | 0.31 | 0.57 | 1.74 | 3.06 | 0 | 0 | -5.46 | 2.52E-08 | 0.00 | 1.04E-02 | 723 |
| Cluster-40555.241750 | 93.76 | 121.14 | 2.79 | 1.72 | 40.32 | 30.63 | 2 | 4.36 | -5.46 | 1.92E-18 | -3.40 | 1.05E-17 | 1034 |
| Cluster-40555.168062 | 300.63 | 261.66 | 5.97 | 5.58 | 37.6 | 32.65 | 8.58 | 7.59 | -5.45 | 2.15E-65 | -2.05 | 4.50E-05 | 601 |
| Cluster-40555.193540 | 1.66 | 0.7 | 0 | 0.05 | 0.86 | 0.66 | 0.07 | 0.07 | -5.45 | 6.25E-03 | -3.42 | 2.74E-03 | 3635 |

| | | | | | | | | | | | | | |
|---|---|---|---|---|---|---|---|---|---|---|---|---|---|
| Cluster-40555.284462 | 11.75 | 7.09 | 0.29 | 0.1 | 6.56 | 4.6 | 0.97 | 0.74 | -5.44 | 1.22E-10 | -2.62 | 4.66E-07 | 2177 |
| Cluster-40555.46109 | 8.42 | 7.33 | 0.15 | 0.19 | 4.94 | 2.65 | 0.4 | 1.04 | -5.44 | 1.56E-10 | -2.31 | 3.13E-02 | 1128 |
| Cluster-40555.164282 | 18.48 | 11.7 | 0.65 | 0 | 6.29 | 4.49 | 1.06 | 0.51 | -5.44 | 1.11E-13 | -2.75 | 3.98E-05 | 1419 |
| Cluster-40555.211632 | 7.41 | 10.93 | 0.37 | 0 | 3.82 | 3.66 | 0.12 | 0 | -5.43 | 3.56E-09 | -6.29 | 1.01E-08 | 1345 |
| Cluster-40555.54148 | 20.41 | 21.34 | 0.19 | 0.68 | 10.77 | 10.49 | 1.39 | 2.21 | -5.42 | 4.05E-26 | -2.49 | 1.85E-06 | 1248 |
| Cluster-40555.197152 | 17.05 | 13.41 | 0.51 | 0.09 | 34.48 | 32.25 | 0.17 | 0 | -5.42 | 5.60E-16 | -8.79 | 2.46E-40 | 950 |
| Cluster-40555.136942 | 1.28 | 0.78 | 0 | 0.05 | 1.16 | 1.77 | 0.17 | 0 | -5.41 | 5.04E-03 | -4.16 | 2.92E-04 | 2129 |
| Cluster-40555.201049 | 6.63 | 8.54 | 0.36 | 0 | 14.39 | 10.49 | 1.2 | 0.45 | -5.41 | 1.43E-11 | -3.86 | 1.03E-12 | 1189 |
| Cluster-40555.218966 | 21.76 | 38.54 | 0 | 1.27 | 13.56 | 22.01 | 0 | 1.06 | -5.41 | 7.41E-05 | -4.99 | 6.49E-06 | 1542 |
| Cluster-40555.180228 | 17.56 | 22.91 | 0 | 0.86 | 6.34 | 8.09 | 1.91 | 0 | -5.40 | 2.46E-14 | -2.91 | 6.08E-05 | 1147 |
| Cluster-40555.190207 | 20.84 | 17.02 | 0.4 | 0.42 | 10.89 | 8.55 | 0.21 | 0.21 | -5.40 | 1.50E-14 | -5.54 | 8.47E-10 | 784 |
| Cluster-40555.187251 | 4.94 | 8.98 | 0.18 | 0.12 | 7.06 | 8.48 | 0 | 0.34 | -5.39 | 8.49E-05 | -5.44 | 7.64E-26 | 2583 |
| Cluster-40555.131891 | 14.77 | 23.75 | 0.22 | 0.66 | 53.14 | 47.45 | 0.24 | 0 | -5.39 | 1.13E-05 | -8.61 | 5.03E-37 | 670 |
| Cluster-40555.218775 | 10.44 | 9.59 | 0 | 0.44 | 8.78 | 12.17 | 0 | 0 | -5.37 | 4.01E-13 | 0.00 | 1.01E-15 | 1185 |
| Cluster-94075.0 | 0.79 | 0.3 | 0.02 | 0 | 0.6 | 0.6 | 0.06 | 0.04 | -5.37 | 2.69E-02 | -3.53 | 3.85E-02 | 2802 |
| Cluster-40555.174366 | 274.63 | 281.82 | 6.72 | 5.56 | 25.15 | 10.16 | 1.24 | 0.55 | -5.36 | 1.77E-60 | -4.23 | 3.01E-02 | 554 |
| Cluster-40555.86454 | 6.41 | 3.76 | 0.16 | 0.07 | 2.26 | 2.64 | 0.13 | 0.29 | -5.36 | 1.77E-08 | -3.50 | 5.55E-05 | 1886 |
| Cluster-40555.193715 | 82.19 | 58.26 | 1.32 | 1.77 | 14.66 | 12.57 | 2.82 | 1.13 | -5.35 | 3.97E-27 | -2.73 | 8.01E-06 | 822 |
| Cluster-40555.220100 | 260.1 | 332.11 | 7.01 | 6.3 | 139.24 | 120.42 | 9.87 | 21.37 | -5.34 | 2.47E-19 | -2.97 | 8.38E-21 | 973 |
| Cluster-40555.264258 | 1.88 | 1.77 | 0 | 0.08 | 0.97 | 1.54 | 0 | 0 | -5.34 | 3.26E-04 | 0.00 | 7.74E-05 | 1748 |
| Cluster-40555.125615 | 19.78 | 28.07 | 0.5 | 0.56 | 6.41 | 8.59 | 0.95 | 1.37 | -5.34 | 6.77E-10 | -2.61 | 8.68E-04 | 878 |
| Cluster-40555.281344 | 30.5 | 31.93 | 0.59 | 0.81 | 2.69 | 2.69 | 0.12 | 0.5 | -5.34 | 1.22E-33 | -3.06 | 7.23E-03 | 1197 |
| Cluster-40555.119825 | 29.59 | 20.48 | 0.45 | 0.66 | 3.96 | 4.01 | 0.11 | 0.22 | -5.33 | 6.90E-21 | -4.55 | 2.02E-06 | 1219 |
| Cluster-40555.125445 | 37.89 | 24.72 | 0.67 | 0.7 | 90.99 | 73.32 | 4.41 | 18.86 | -5.33 | 1.80E-15 | -2.73 | 1.21E-14 | 1329 |
| Cluster-40555.306404 | 2.02 | 1.72 | 0.05 | 0.03 | 1.29 | 1.11 | 0.04 | 0.09 | -5.32 | 1.22E-03 | -4.14 | 2.24E-02 | 1453 |
| Cluster-40555.230894 | 6.55 | 5.09 | 0.02 | 0.26 | 4.46 | 1.94 | 0.13 | 0.29 | -5.31 | 2.22E-11 | -3.86 | 2.61E-02 | 1639 |
| Cluster-40555.198012 | 5.22 | 9.5 | 0.24 | 0.11 | 4.56 | 6.15 | 0.38 | 1.26 | -5.31 | 2.69E-04 | -2.60 | 1.75E-03 | 1031 |
| Cluster-40555.313638 | 1.32 | 0.86 | 0 | 0.05 | 1.9 | 2.85 | 0 | 0.36 | -5.29 | 4.87E-04 | -3.63 | 1.19E-04 | 2701 |
| Cluster-40555.180831 | 64.5 | 50.43 | 1.45 | 1.24 | 68.65 | 65.51 | 7.11 | 8.18 | -5.29 | 1.17E-22 | -3.07 | 9.03E-13 | 559 |
| Cluster-40555.127354 | 13.05 | 17.82 | 0.15 | 0.57 | 77.08 | 84.73 | 0.37 | 0.38 | -5.29 | 1.00E-09 | -7.71 | 1.70E-47 | 682 |
| Cluster-40555.302712 | 7.1 | 3.72 | 0.13 | 0.12 | 5.07 | 5.33 | 1.1 | 1.02 | -5.28 | 1.87E-05 | -2.24 | 2.96E-03 | 1232 |
| Cluster-40555.234359 | 22.75 | 34 | 0 | 1.29 | 1.93 | 0.93 | 0 | 0 | -5.28 | 8.23E-08 | 0.00 | 7.81E-03 | 1051 |

| | | | | | | | | | | | | | |
|---|---|---|---|---|---|---|---|---|---|---|---|---|---|
| Cluster-40555.44294 | 12.09 | 11.77 | 0 | 0.53 | 5.48 | 4.37 | 0 | 0 | -5.27 | 1.25E-07 | 0.00 | 2.07E-05 | 695 |
| Cluster-40555.265647 | 11 | 8.92 | 0.29 | 0.18 | 14.52 | 18.33 | 0.63 | 0.49 | -5.26 | 7.48E-10 | -4.82 | 1.17E-15 | 907 |
| Cluster-40555.173407 | 3.29 | 1.98 | 0 | 0.12 | 2.09 | 2.1 | 0.13 | 0.07 | -5.26 | 3.58E-05 | -4.37 | 1.45E-04 | 1574 |
| Cluster-40555.168004 | 22.4 | 48.67 | 1.78 | 0 | 20.78 | 15.67 | 0 | 0 | -5.25 | 3.00E-03 | 0.00 | 9.00E-24 | 823 |
| Cluster-40555.256918 | 37.2 | 28.93 | 0.76 | 0.86 | 9.15 | 11.52 | 2.38 | 2.24 | -5.25 | 4.20E-19 | -2.10 | 1.15E-02 | 688 |
| Cluster-40555.145471 | 7.18 | 8.91 | 0.21 | 0.16 | 3.87 | 6.02 | 0.09 | 0.19 | -5.25 | 1.60E-20 | -5.15 | 1.15E-06 | 2201 |
| Cluster-40555.217602 | 6.28 | 8.52 | 0.22 | 0.16 | 13.57 | 10.78 | 0 | 0 | -5.25 | 9.98E-07 | 0.00 | 4.48E-17 | 830 |
| Cluster-40555.147304 | 3.74 | 2.67 | 0 | 0.16 | 2.12 | 1.41 | 0.1 | 0 | -5.25 | 2.47E-06 | -4.92 | 2.27E-04 | 1597 |
| Cluster-40555.196596 | 12.7 | 17.53 | 0.33 | 0.39 | 9.17 | 12.48 | 0.17 | 0.25 | -5.25 | 1.12E-11 | -5.66 | 3.96E-13 | 1516 |
| Cluster-40555.165455 | 10.04 | 7.03 | 0.37 | 0.07 | 3 | 3.06 | 0.53 | 0.66 | -5.25 | 2.79E-17 | -2.29 | 5.72E-03 | 1651 |
| Cluster-40555.195354 | 2.77 | 3.22 | 0.15 | 0 | 2.76 | 2.52 | 0.38 | 0.3 | -5.24 | 3.19E-04 | -2.90 | 2.30E-02 | 1096 |
| Cluster-40555.42223 | 13.89 | 12.44 | 0.35 | 0.28 | 3.58 | 4.56 | 0.68 | 0.33 | -5.23 | 6.15E-24 | -2.96 | 2.90E-05 | 1664 |
| Cluster-40555.44040 | 5.06 | 2.75 | 0.11 | 0.07 | 3.01 | 3.71 | 0.38 | 0 | -5.23 | 2.23E-03 | -4.15 | 6.36E-03 | 805 |
| Cluster-40555.97406 | 4.96 | 6.17 | 0 | 0.26 | 3.7 | 4.01 | 0.27 | 0.58 | -5.23 | 7.27E-04 | -3.18 | 3.72E-02 | 716 |
| Cluster-40555.179923 | 4.13 | 4.3 | 0.09 | 0.12 | 4.49 | 6.96 | 0.08 | 0.15 | -5.22 | 1.38E-04 | -5.60 | 2.10E-06 | 951 |
| Cluster-40555.134085 | 12.69 | 15.83 | 0 | 0.69 | 2.05 | 2.07 | 0 | 0.75 | -5.22 | 7.95E-19 | -2.36 | 5.64E-03 | 2122 |
| Cluster-40555.182040 | 7.96 | 5.92 | 0.2 | 0.12 | 3.7 | 3 | 0.19 | 0.09 | -5.21 | 6.98E-15 | -4.39 | 9.12E-08 | 1752 |
| Cluster-40555.168005 | 80.18 | 68.53 | 1.24 | 2.37 | 52.24 | 55.3 | 3.07 | 1.53 | -5.21 | 1.24E-41 | -4.49 | 2.19E-27 | 851 |
| Cluster-40555.154093 | 7.84 | 5.59 | 0.22 | 0.12 | 2.53 | 1.47 | 0.11 | 0.09 | -5.21 | 7.63E-14 | -4.11 | 2.23E-04 | 1674 |
| Cluster-40555.163448 | 195.56 | 155.13 | 3.6 | 4.98 | 82.76 | 69.42 | 7.83 | 9.52 | -5.20 | 4.59E-75 | -3.06 | 4.18E-21 | 1317 |
| Cluster-40555.306458 | 3.25 | 4.52 | 0.12 | 0.08 | 1 | 2.29 | 0 | 0 | -5.20 | 2.58E-03 | 0.00 | 4.36E-02 | 783 |
| Cluster-40555.185479 | 5.49 | 4.91 | 0.25 | 0 | 3.59 | 3.7 | 0.34 | 0 | -5.19 | 5.28E-06 | -4.30 | 4.11E-04 | 962 |
| Cluster-40555.227546 | 5.98 | 6.66 | 0.22 | 0.12 | 5.2 | 4.06 | 0.55 | 0 | -5.19 | 2.76E-03 | -4.23 | 2.04E-02 | 583 |
| Cluster-40555.230597 | 13.24 | 10.26 | 0 | 0.58 | 2.24 | 1.16 | 0.22 | 0.19 | -5.19 | 8.84E-18 | -3.06 | 2.41E-02 | 1447 |
| Cluster-40555.199701 | 7.21 | 8.91 | 0 | 0.39 | 9.95 | 9.81 | 1.22 | 0.09 | -5.18 | 1.09E-18 | -3.88 | 8.93E-17 | 2071 |
| Cluster-40555.154705 | 35.49 | 34.33 | 0.89 | 0.89 | 5.7 | 6.48 | 0.43 | 0 | -5.18 | 1.54E-22 | -4.87 | 2.04E-05 | 760 |
| Cluster-40555.194778 | 2.63 | 2.5 | 0 | 0.12 | 3.95 | 3.33 | 0 | 0.6 | -5.17 | 1.21E-03 | -3.48 | 2.14E-04 | 1202 |
| Cluster-40555.133412 | 17.57 | 10.46 | 0.46 | 0.23 | 1.37 | 1.65 | 0 | 0 | -5.16 | 2.15E-09 | 0.00 | 6.64E-04 | 1290 |
| Cluster-40555.226291 | 13.47 | 12.68 | 0 | 0.64 | 3.81 | 5.61 | 0.87 | 0.78 | -5.16 | 5.53E-13 | -2.43 | 1.03E-02 | 995 |
| Cluster-40555.148605 | 40.65 | 36.02 | 1.57 | 0.42 | 17.58 | 20.77 | 0.75 | 2.15 | -5.15 | 8.75E-39 | -3.65 | 7.06E-16 | 1205 |
| Cluster-40555.130242 | 69.35 | 62.47 | 0.67 | 2.73 | 23.1 | 11.68 | 2.46 | 3.08 | -5.13 | 2.68E-43 | -2.56 | 2.71E-02 | 1022 |
| Cluster-40555.196295 | 4.9 | 4.17 | 0.12 | 0.1 | 19.93 | 20.28 | 0 | 0 | -5.11 | 1.09E-09 | 0.00 | 5.38E-50 | 1704 |

| | | | | | | | | | | | | | |
|---|---|---|---|---|---|---|---|---|---|---|---|---|---|
| Cluster-40555.227814 | 63.49 | 112.34 | 1.69 | 2.97 | 3.47 | 1.96 | 0.38 | 0.31 | -5.11 | 9.83E-05 | -2.97 | 3.72E-02 | 967 |
| Cluster-40555.178963 | 80.42 | 76.65 | 0.91 | 3.15 | 16.26 | 15.21 | 0.78 | 0.55 | -5.11 | 6.19E-20 | -4.51 | 3.71E-05 | 463 |
| Cluster-40555.159459 | 87.57 | 68 | 0 | 3.87 | 23.5 | 24.62 | 2.2 | 4.05 | -5.10 | 1.51E-10 | -2.89 | 2.25E-02 | 360 |
| Cluster-40555.194143 | 157.63 | 113.42 | 3.14 | 4.02 | 78.56 | 72.01 | 13.2 | 10.52 | -5.09 | 1.71E-28 | -2.60 | 7.09E-17 | 1817 |
| Cluster-40555.156367 | 4.29 | 2.38 | 0.19 | 0 | 8.51 | 7.05 | 0.77 | 0.16 | -5.09 | 1.00E-03 | -3.97 | 2.82E-07 | 951 |
| Cluster-40555.204666 | 10.55 | 7.97 | 0 | 0.55 | 7.16 | 5.01 | 0.21 | 0.55 | -5.09 | 2.02E-03 | -3.75 | 3.09E-02 | 511 |
| Cluster-40555.298861 | 7.58 | 8.9 | 0.19 | 0.25 | 8.05 | 8.28 | 0.8 | 1 | -5.09 | 4.01E-04 | -3.12 | 6.08E-03 | 583 |
| Cluster-40555.207073 | 5.24 | 2.95 | 0.15 | 0.06 | 1.62 | 1.25 | 0.02 | 0.28 | -5.08 | 8.26E-07 | -3.26 | 1.01E-02 | 1750 |
| Cluster-40555.305167 | 28.25 | 30.12 | 1.1 | 0.47 | 4.99 | 3.56 | 0.71 | 0.27 | -5.08 | 7.91E-20 | -3.00 | 2.27E-02 | 764 |
| Cluster-40555.209849 | 16.78 | 17.5 | 0 | 0.96 | 14.26 | 8.94 | 0 | 0.86 | -5.07 | 1.95E-07 | -4.62 | 5.07E-06 | 567 |
| Cluster-40555.209712 | 10.91 | 7.75 | 0 | 0.48 | 2.9 | 1.61 | 0.48 | 0.13 | -5.06 | 7.46E-17 | -2.78 | 6.08E-03 | 1713 |
| Cluster-40555.173159 | 2.58 | 1.55 | 0 | 0.1 | 1.58 | 2.03 | 0.09 | 0.75 | -5.06 | 2.30E-05 | -2.00 | 4.35E-02 | 2082 |
| Cluster-40555.195197 | 0.71 | 1.57 | 0.05 | 0 | 5.75 | 7.1 | 0 | 0 | -5.06 | 1.52E-02 | 0.00 | 7.57E-28 | 2178 |
| Cluster-40555.38155 | 3.38 | 3.54 | 0 | 0.19 | 3.37 | 2.77 | 0.32 | 0.24 | -5.05 | 2.54E-03 | -3.39 | 1.49E-02 | 905 |
| Cluster-40555.195071 | 13.36 | 17.78 | 0.44 | 0.42 | 4.89 | 5.3 | 0.42 | 0.05 | -5.04 | 5.97E-14 | -4.37 | 5.79E-12 | 1947 |
| Cluster-40555.189422 | 80.27 | 72.55 | 2.75 | 1.47 | 34.32 | 41.54 | 1.09 | 4.94 | -5.04 | 1.72E-55 | -3.57 | 7.59E-15 | 1264 |
| Cluster-40555.211533 | 7.04 | 7.98 | 0 | 0.38 | 9.11 | 8.51 | 0.7 | 1.43 | -5.04 | 2.70E-06 | -2.97 | 5.53E-05 | 848 |
| Cluster-40555.198787 | 45.84 | 57.51 | 0.83 | 1.97 | 5.08 | 4.85 | 1.08 | 0.26 | -5.03 | 1.43E-18 | -2.88 | 5.60E-04 | 1120 |
| Cluster-40555.184193 | 8.25 | 7.09 | 0 | 0.42 | 6.34 | 6.13 | 0.57 | 0 | -5.03 | 1.16E-18 | -4.41 | 8.28E-16 | 2268 |
| Cluster-40555.190125 | 14.91 | 12.51 | 0.14 | 0.61 | 5.14 | 4.19 | 1.03 | 0.92 | -5.03 | 1.75E-17 | -2.17 | 5.57E-03 | 1275 |
| Cluster-40555.190916 | 1.97 | 2.09 | 0.11 | 0 | 0.46 | 0.37 | 0 | 0.05 | -5.03 | 3.44E-17 | -3.86 | 1.18E-03 | 6130 |
| Cluster-40555.137470 | 4.99 | 3.57 | 0.14 | 0.11 | 5.98 | 5.39 | 0.13 | 0.57 | -5.03 | 7.31E-03 | -3.86 | 1.44E-03 | 687 |
| Cluster-40555.178650 | 15.72 | 12.1 | 0.29 | 0.47 | 5.97 | 5.39 | 0.88 | 0.28 | -5.02 | 5.28E-38 | -3.24 | 1.96E-12 | 3142 |
| Cluster-40555.306386 | 6.92 | 4.22 | 0 | 0.3 | 8.27 | 5.1 | 1.23 | 0.44 | -5.02 | 3.38E-03 | -3.02 | 8.34E-03 | 653 |
| Cluster-40555.105101 | 7.53 | 8.02 | 0.33 | 0.11 | 2.66 | 2.05 | 0.37 | 0.28 | -5.02 | 1.50E-12 | -2.80 | 1.34E-02 | 1367 |
| Cluster-40555.237321 | 22.8 | 12.33 | 0 | 0.98 | 12.78 | 8.68 | 1.35 | 0.47 | -5.01 | 8.14E-06 | -3.49 | 2.70E-09 | 1095 |
| Cluster-40555.242043 | 24.57 | 25.37 | 0.35 | 1.09 | 7.12 | 6.39 | 1.37 | 1 | -5.00 | 2.25E-13 | -2.39 | 3.66E-02 | 662 |
| Cluster-40555.302334 | 9.31 | 9.66 | 0.23 | 0.3 | 4.91 | 4.42 | 0.2 | 0.25 | -5.00 | 8.24E-04 | -4.48 | 3.86E-02 | 524 |
| Cluster-40555.194651 | 1.76 | 1.83 | 0 | 0.11 | 3.36 | 3.74 | 0.1 | 0.11 | -5.00 | 3.63E-03 | -4.98 | 1.06E-07 | 1453 |
| Cluster-40555.187913 | 162.58 | 229.56 | 8.39 | 2.64 | 60.28 | 98.96 | 0 | 0 | -4.99 | 3.32E-03 | 0.00 | 2.50E-02 | 245 |
| Cluster-40555.219599 | 72.61 | 69.92 | 3.86 | 0.3 | 18.55 | 30.81 | 4.65 | 4.83 | -4.99 | 1.86E-53 | -2.33 | 3.07E-02 | 1188 |
| Cluster-40555.192342 | 40.6 | 41.1 | 0.99 | 1.37 | 8.43 | 7.55 | 2.3 | 1.81 | -4.98 | 2.07E-41 | -1.89 | 8.50E-04 | 1400 |

| | | | | | | | | | | | | | | |
|---|---|---|---|---|---|---|---|---|---|---|---|---|---|---|
| Cluster-40555.298862 | 16.77 | 10.43 | 0.43 | 0.35 | 8.67 | 8.22 | 0.53 | 0.95 | -4.98 | 8.67E-11 | -3.43 | 9.22E-10 | 1404 |
| Cluster-40555.180701 | 12.96 | 19.34 | 0 | 0.92 | 3.33 | 3.36 | 0 | 0.17 | -4.97 | 1.37E-07 | -5.19 | 1.50E-12 | 2375 |
| Cluster-40555.203363 | 99.01 | 70.09 | 1.19 | 3.69 | 17.12 | 19.12 | 3.97 | 2.62 | -4.96 | 4.33E-24 | -2.40 | 1.07E-05 | 801 |
| Cluster-40555.205293 | 8.2 | 6.44 | 0.18 | 0.25 | 1.21 | 1.79 | 0.47 | 0.24 | -4.95 | 5.25E-22 | -2.08 | 2.49E-02 | 2771 |
| Cluster-40555.177214 | 2.69 | 2.5 | 0.07 | 0.09 | 1.86 | 1.91 | 0.16 | 0 | -4.95 | 1.16E-03 | -4.50 | 4.89E-03 | 1193 |
| Cluster-40555.135462 | 3.45 | 3.01 | 0 | 0.18 | 2.11 | 1.05 | 0.16 | 0 | -4.95 | 6.75E-05 | -4.43 | 6.79E-03 | 1333 |
| Cluster-40555.231415 | 4.07 | 4.21 | 0 | 0.23 | 2.62 | 2.47 | 0.08 | 0 | -4.94 | 5.73E-04 | -6.02 | 1.24E-03 | 927 |
| Cluster-40555.256273 | 8.71 | 13.99 | 0.61 | 0.08 | 2.63 | 2.37 | 0 | 0.25 | -4.94 | 2.81E-06 | -4.30 | 1.46E-04 | 1361 |
| Cluster-40555.307417 | 3.83 | 2.82 | 0.12 | 0.06 | 0.98 | 1.47 | 0 | 0 | -4.94 | 1.10E-06 | | 3.51E-04 | 1601 |
| Cluster-40555.198525 | 32.91 | 29.78 | 1.4 | 0.48 | 10.22 | 10.58 | 1.39 | 2.03 | -4.93 | 7.46E-41 | -2.53 | 5.44E-08 | 1634 |
| Cluster-40555.177582 | 11.63 | 14.5 | 0.63 | 0.17 | 2.84 | 2.98 | 0.1 | 0.21 | -4.93 | 6.19E-11 | -4.33 | 8.68E-03 | 827 |
| Cluster-40555.152512 | 2.17 | 1.95 | 0.08 | 0.04 | 2.61 | 2.3 | 0.15 | 0.27 | -4.91 | 2.70E-12 | -3.50 | 1.37E-10 | 4484 |
| Cluster-40555.165165 | 4.17 | 5.89 | 0.37 | 0 | 3.6 | 1.75 | 0 | 0 | -4.91 | 3.03E-03 | 0.00 | 1.39E-02 | 653 |
| Cluster-99294.0 | 3.04 | 1.3 | 0.16 | 0 | 0.95 | 0.97 | 0 | 0 | -4.91 | 7.56E-03 | 0.00 | 3.52E-02 | 1191 |
| Cluster-40555.142361 | 133.87 | 202.81 | 2.89 | 7.27 | 131.55 | 196.54 | 18.41 | 18.35 | -4.91 | 2.89E-06 | -3.11 | 2.11E-04 | 452 |
| Cluster-40555.203684 | 12.56 | 20.85 | 0.54 | 0.49 | 10.09 | 8.74 | 0.64 | 2.04 | -4.90 | 3.59E-05 | -2.71 | 8.39E-05 | 873 |
| Cluster-40555.172594 | 24.37 | 21.52 | 1.45 | 0 | 2.11 | 0.85 | 0 | 0 | -4.89 | 1.93E-29 | 0.00 | 5.04E-03 | 1337 |
| Cluster-40555.214922 | 46.56 | 31.82 | 0.91 | 1.46 | 9.82 | 10.38 | 0.57 | 0.71 | -4.89 | 4.10E-18 | -3.91 | 1.16E-16 | 1929 |
| Cluster-40555.108315 | 9.11 | 8.3 | 0.32 | 0.21 | 24.07 | 25.41 | 0.83 | 0.59 | -4.89 | 1.45E-02 | -5.08 | 3.34E-08 | 451 |
| Cluster-40555.155945 | 6.29 | 3.09 | 0.08 | 0.21 | 3.21 | 2.22 | 0.61 | 0.34 | -4.88 | 3.05E-04 | -2.46 | 6.36E-04 | 2245 |
| Cluster-40555.112504 | 21.1 | 17.93 | 0.68 | 0.52 | 3.66 | 2 | 0.29 | 0.23 | -4.87 | 2.54E-24 | -3.34 | 1.66E-03 | 1329 |
| Cluster-40555.177472 | 23.88 | 22.82 | 0.92 | 0.52 | 5.96 | 3.58 | 0.29 | 1.01 | -4.86 | 4.10E-33 | -2.76 | 7.18E-04 | 1630 |
| Cluster-40555.119406 | 12.02 | 8.01 | 0 | 0.63 | 7.61 | 6.66 | 1.15 | 0.68 | -4.86 | 1.56E-04 | -2.96 | 1.79E-02 | 588 |
| Cluster-40555.233000 | 3.56 | 2.79 | 0.11 | 0.07 | 1.37 | 1.18 | 0.08 | 0 | -4.86 | 3.60E-09 | -5.07 | 6.21E-05 | 2296 |
| Cluster-40555.143098 | 1.89 | 0.86 | 0 | 0.09 | 2.05 | 1.69 | 0.31 | 0.09 | -4.86 | 8.78E-03 | -3.21 | 4.04E-03 | 1689 |
| Cluster-40555.229143 | 2.31 | 1.6 | 0 | 0.12 | 2.3 | 1.59 | 0.34 | 0.15 | -4.85 | 1.71E-04 | -2.93 | 2.66E-03 | 1917 |
| Cluster-40555.276398 | 1.26 | 1.05 | 0 | 0.06 | 1.38 | 1.23 | 0.13 | 0.25 | -4.85 | 8.63E-03 | -2.75 | 3.03E-02 | 1931 |
| Cluster-40555.142929 | 16.59 | 17.28 | 0.26 | 0.86 | 6.32 | 8.76 | 0 | 0.18 | -4.85 | 1.75E-12 | -6.26 | 3.11E-09 | 830 |
| Cluster-40555.136865 | 2.38 | 4.27 | 0.11 | 0.13 | 2.89 | 2.37 | 0.1 | 0.1 | -4.85 | 2.23E-03 | -5.02 | 2.31E-03 | 923 |
| Cluster-40555.230890 | 48.33 | 37.9 | 1.13 | 1.55 | 8.55 | 5.41 | 0.82 | 1.19 | -4.85 | 9.87E-34 | -2.70 | 1.04E-04 | 1075 |
| Cluster-40555.200479 | 79.31 | 53.52 | 2.66 | 1.54 | 47.15 | 29.74 | 0.71 | 0.72 | -4.84 | 3.89E-18 | -5.60 | 9.57E-13 | 811 |
| Cluster-40555.217911 | 14.58 | 11.07 | 0.43 | 0.39 | 4.4 | 2.16 | 0.15 | 0 | -4.83 | 1.18E-24 | -5.41 | 2.84E-04 | 1951 |

| | | | | | | | | | | | | | |
|---|---|---|---|---|---|---|---|---|---|---|---|---|---|
| Cluster-40555.169434 | 57.04 | 92.09 | 2.43 | 2.39 | 6.97 | 8.09 | 0.94 | 0.48 | -4.83 | 5.16E-06 | -3.35 | 4.29E-07 | 1157 |
| Cluster-40555.314682 | 6.67 | 6.5 | 0.43 | 0 | 18.8 | 22.76 | 0.6 | 0.12 | -4.83 | 1.88E-04 | -5.82 | 3.16E-16 | 691 |
| Cluster-40555.210969 | 23.02 | 27.97 | 1.33 | 0.34 | 2.24 | 1.48 | 0.72 | 0 | -4.82 | 1.91E-25 | -2.33 | 2.15E-02 | 3559 |
| Cluster-40555.180039 | 2.47 | 1.96 | 0 | 0.14 | 2.72 | 3.55 | 0.5 | 0.27 | -4.82 | 1.69E-10 | -2.99 | 1.27E-07 | 3981 |
| Cluster-40555.216663 | 2.32 | 3.67 | 0 | 0.19 | 1.78 | 1.47 | 0 | 0.07 | -4.82 | 1.11E-05 | -5.29 | 2.90E-10 | 3608 |
| Cluster-40555.227691 | 2.22 | 2.51 | 0.17 | 0 | 0.61 | 1.24 | 0 | 0 | -4.81 | 2.12E-04 | 0.00 | 1.09E-02 | 1439 |
| Cluster-40555.216427 | 5.6 | 3.31 | 0.29 | 0 | 4.3 | 2.49 | 0.3 | 0.35 | -4.80 | 1.03E-05 | -3.25 | 2.21E-03 | 1102 |
| Cluster-40555.303156 | 6.83 | 8.71 | 0.35 | 0.19 | 37.75 | 44.42 | 0.12 | 0.75 | -4.80 | 4.75E-10 | -6.50 | 1.55E-40 | 1172 |
| Cluster-40555.299592 | 4.94 | 6.14 | 0.12 | 0.24 | 10.02 | 8.05 | 1.22 | 1.16 | -4.80 | 4.83E-04 | -2.86 | 3.48E-04 | 762 |
| Cluster-40555.257805 | 9.68 | 7.31 | 0.37 | 0.16 | 13.09 | 9 | 2.33 | 1.02 | -4.79 | 5.71E-13 | -2.67 | 2.84E-07 | 1393 |
| Cluster-40555.229481 | 3.09 | 2.37 | 0.08 | 0.1 | 1.89 | 1.88 | 0.19 | 0 | -4.79 | 3.39E-03 | -4.26 | 1.77E-02 | 1056 |
| Cluster-40555.99984 | 2.41 | 1.59 | 0.13 | 0 | 1.98 | 2.29 | 0 | 0 | -4.79 | 6.40E-03 | 0.00 | 4.57E-05 | 1183 |
| Cluster-40555.175851 | 7.16 | 8.99 | 0.53 | 0 | 20.78 | 19.36 | 0 | 0.09 | -4.79 | 2.05E-15 | -8.93 | 2.69E-45 | 1617 |
| Cluster-40555.230948 | 2.48 | 4.52 | 0 | 0.22 | 1.85 | 2.02 | 0.44 | 0.17 | -4.79 | 1.42E-03 | -2.61 | 6.41E-03 | 2009 |
| Cluster-40555.186221 | 5.53 | 5.57 | 0 | 0.35 | 8.33 | 7.39 | 0.02 | 0.11 | -4.79 | 4.26E-08 | -7.29 | 7.53E-19 | 1372 |
| Cluster-40555.194295 | 1.78 | 1.82 | 0.12 | 0 | 3.43 | 3.17 | 1.25 | 0 | -4.78 | 1.19E-05 | -2.38 | 3.08E-04 | 2287 |
| Cluster-40555.192895 | 1024.08 | 895 | 19.42 | 43.11 | 394.13 | 354.41 | 28.37 | 43.31 | -4.78 | 6.31E-75 | -3.31 | 1.44E-26 | 658 |
| Cluster-40555.105157 | 11.4 | 12.85 | 0.23 | 0.58 | 3.16 | 3.95 | 0 | 0.23 | -4.78 | 4.95E-09 | -5.13 | 7.75E-04 | 814 |
| Cluster-40555.194189 | 4.25 | 2.76 | 0 | 0.23 | 0.66 | 1.19 | 0 | 0 | -4.76 | 6.07E-11 | 0.00 | 1.54E-05 | 4099 |
| Cluster-40555.117989 | 2.02 | 1.15 | 0.07 | 0.06 | 0.76 | 0.43 | 0.08 | 0 | -4.76 | 3.39E-05 | -3.73 | 4.88E-02 | 2564 |
| Cluster-40555.149377 | 27.3 | 31.76 | 0.85 | 1.1 | 3.49 | 2.92 | 0.85 | 0.37 | -4.75 | 2.67E-28 | -2.35 | 2.72E-02 | 1184 |
| Cluster-40555.192618 | 15.8 | 13.12 | 0.52 | 0.48 | 10.83 | 10.23 | 1.21 | 0.74 | -4.75 | 4.15E-19 | -3.38 | 1.09E-10 | 1362 |
| Cluster-40555.189232 | 38.5 | 30.26 | 1.3 | 1.02 | 14.49 | 14.64 | 0.24 | 0.14 | -4.75 | 3.95E-46 | -6.16 | 1.11E-35 | 2077 |
| Cluster-40555.179635 | 72.26 | 110.26 | 3.06 | 3.16 | 10.42 | 10.51 | 1.12 | 1.6 | -4.74 | 3.09E-07 | -2.88 | 3.11E-08 | 1305 |
| Cluster-40555.317813 | 2.78 | 1.66 | 0 | 0.15 | 2.03 | 1.27 | 0 | 0.06 | -4.74 | 1.54E-02 | -5.60 | 7.45E-03 | 1081 |
| Cluster-40555.177454 | 19.94 | 27.8 | 1.38 | 0.3 | 15.76 | 13.78 | 0 | 0.29 | -4.74 | 1.15E-09 | -6.56 | 1.95E-16 | 803 |
| Cluster-40555.186918 | 25.52 | 35.8 | 0.99 | 1.09 | 23.16 | 19.46 | 0.63 | 1.15 | -4.73 | 6.05E-09 | -4.57 | 2.84E-13 | 693 |
| Cluster-40555.305335 | 0.45 | 0.56 | 0 | 0.03 | 0.56 | 0.59 | 0.04 | 0 | -4.73 | 1.53E-02 | -5.32 | 4.25E-04 | 3799 |
| Cluster-40555.138650 | 10.28 | 12.56 | 0.42 | 0.35 | 3.61 | 4.83 | 0.31 | 0.57 | -4.71 | 1.18E-17 | -3.16 | 1.66E-05 | 1509 |
| Cluster-40555.239614 | 50.57 | 56.43 | 2 | 1.78 | 11.8 | 10.29 | 0 | 0.24 | -4.71 | 4.23E-15 | -6.41 | 2.35E-05 | 484 |
| Cluster-40555.237532 | 2.32 | 4.3 | 0.15 | 0.08 | 1.43 | 1.14 | 0 | 0 | -4.70 | 6.91E-04 | 0.00 | 1.35E-09 | 3117 |
| Cluster-40555.263524 | 3.11 | 3.72 | 0.1 | 0.13 | 3.35 | 3.44 | 0 | 0 | -4.70 | 5.66E-03 | 0.00 | 4.06E-05 | 853 |

| | | | | | | | | | | | | | |
|---|---|---|---|---|---|---|---|---|---|---|---|---|---|
| Cluster-40555.45003 | 10.24 | 11.31 | 0.33 | 0.42 | 3.92 | 4.65 | 0.43 | 0.77 | -4.70 | 1.08E-10 | -2.81 | 3.81E-03 | 1011 |
| Cluster-40555.151851 | 8.93 | 9.66 | 0.13 | 0.47 | 2.13 | 2.87 | 0.11 | 0.32 | -4.70 | 4.18E-09 | -3.29 | 2.27E-02 | 1007 |
| Cluster-40555.161822 | 2.24 | 5.3 | 0 | 0.28 | 4.33 | 5.85 | 0 | 0 | -4.69 | 4.83E-02 | 0.00 | 9.43E-14 | 1321 |
| Cluster-40555.303679 | 10.75 | 15.25 | 0.22 | 0.69 | 13.05 | 9.2 | 1.07 | 0.92 | -4.69 | 7.93E-08 | -3.41 | 5.47E-07 | 833 |
| Cluster-40555.101278 | 40.97 | 40.35 | 1.45 | 1.44 | 7.62 | 6.37 | 2.17 | 2.37 | -4.68 | 9.15E-48 | -1.55 | 2.44E-03 | 2003 |
| Cluster-40555.192541 | 9.45 | 7.78 | 0.43 | 0.2 | 4.57 | 4.3 | 1.75 | 1.05 | -4.67 | 6.04E-20 | -1.59 | 8.50E-03 | 2202 |
| Cluster-40555.100208 | 9.55 | 11.43 | 0.16 | 0.59 | 3.7 | 2.85 | 0.31 | 0.27 | -4.67 | 9.08E-11 | -3.46 | 2.03E-03 | 1071 |
| Cluster-40555.216124 | 126.07 | 125.25 | 3.36 | 5.55 | 57.96 | 56.19 | 7.47 | 7.47 | -4.67 | 7.34E-50 | -2.86 | 3.62E-15 | 919 |
| Cluster-40555.198474 | 80.63 | 74.96 | 1.95 | 3.64 | 36.13 | 31.71 | 9.04 | 13.97 | -4.66 | 1.46E-25 | -1.50 | 5.90E-03 | 582 |
| Cluster-40555.144579 | 15.6 | 15.62 | 0 | 1.12 | 18.16 | 20.44 | 2.43 | 4.62 | -4.66 | 3.25E-09 | -2.38 | 1.85E-05 | 734 |
| Cluster-40555.65557 | 7.06 | 5.47 | 0.27 | 0.18 | 17.78 | 25.1 | 0.06 | 0 | -4.66 | 8.88E-08 | -9.56 | 1.02E-16 | 1163 |
| Cluster-40555.191739 | 34.4 | 42.6 | 1.47 | 1.34 | 14.8 | 9.11 | 1.19 | 1.03 | -4.66 | 1.33E-16 | -3.36 | 1.24E-05 | 682 |
| Cluster-40555.139511 | 71.31 | 104.11 | 3.49 | 2.9 | 89.87 | 111.09 | 4.59 | 5.77 | -4.65 | 7.29E-08 | -4.22 | 7.57E-18 | 685 |
| Cluster-40555.238890 | 96.39 | 66.73 | 2.64 | 3.24 | 32.55 | 29.36 | 5.73 | 6.97 | -4.64 | 7.24E-20 | -2.22 | 1.94E-07 | 866 |
| Cluster-40555.174782 | 22.12 | 21.64 | 0.82 | 0.79 | 11.36 | 10.26 | 1.16 | 1.09 | -4.64 | 7.33E-32 | -3.18 | 2.29E-12 | 1809 |
| Cluster-40555.160981 | 1.43 | 1.76 | 0.11 | 0 | 1.25 | 0.8 | 0.08 | 0.04 | -4.64 | 3.30E-06 | -4.05 | 4.75E-04 | 2832 |
| Cluster-40555.230451 | 127.59 | 96.43 | 2.25 | 5.82 | 152.2 | 163.06 | 6.35 | 8.81 | -4.64 | 2.35E-29 | -4.32 | 5.43E-30 | 546 |
| Cluster-40555.42142 | 9.38 | 8.69 | 0.46 | 0.21 | 3.81 | 2.66 | 0.75 | 0.06 | -4.63 | 2.19E-11 | -2.97 | 3.85E-03 | 1207 |
| Cluster-40555.139066 | 93.45 | 112.55 | 3.66 | 3.94 | 7 | 4.48 | 0.32 | 0.67 | -4.63 | 1.18E-19 | -3.46 | 1.82E-02 | 584 |
| Cluster-40555.172001 | 50.81 | 54.44 | 2.66 | 1.24 | 1.99 | 1.4 | 0 | 0.5 | -4.63 | 3.06E-59 | -2.63 | 2.03E-03 | 2507 |
| Cluster-40555.197795 | 48.3 | 30.93 | 1.53 | 1.52 | 17.08 | 11.23 | 2.78 | 0 | -4.62 | 9.52E-09 | -3.28 | 9.63E-03 | 430 |
| Cluster-40555.177740 | 45.88 | 47.88 | 1.33 | 2.11 | 6.5 | 5.49 | 1.4 | 0.42 | -4.62 | 3.45E-52 | -2.68 | 8.47E-08 | 2304 |
| Cluster-40555.207615 | 10.39 | 12.73 | 0.3 | 0.55 | 3.22 | 3.72 | 0 | 0.36 | -4.62 | 1.09E-16 | -4.10 | 2.01E-06 | 1488 |
| Cluster-40555.158304 | 47.47 | 40.17 | 2.65 | 0.61 | 27.6 | 19.66 | 3.49 | 1.82 | -4.62 | 1.04E-17 | -3.09 | 6.09E-07 | 586 |
| Cluster-40555.184841 | 4.72 | 6.7 | 0.43 | 0 | 7.38 | 8.15 | 0.36 | 1.15 | -4.61 | 2.85E-08 | -3.27 | 9.96E-08 | 1246 |
| Cluster-40555.181280 | 13.66 | 10.97 | 0.6 | 0.37 | 4.64 | 5.36 | 0.45 | 0.21 | -4.60 | 4.34E-09 | -3.90 | 5.43E-04 | 813 |
| Cluster-40555.241914 | 18.78 | 12.71 | 0.63 | 0.65 | 13.84 | 16.44 | 2.36 | 3.48 | -4.60 | 6.28E-07 | -2.33 | 2.15E-03 | 591 |
| Cluster-40555.158876 | 14.69 | 32.85 | 1.38 | 0.45 | 13.94 | 13.09 | 0.58 | 0.61 | -4.60 | 2.06E-02 | -4.44 | 8.97E-06 | 528 |
| Cluster-40555.165424 | 47.14 | 50.04 | 1.41 | 2.22 | 6.12 | 5.7 | 0.9 | 0.94 | -4.59 | 5.73E-38 | -2.59 | 1.69E-04 | 1250 |
| Cluster-40555.185621 | 18.7 | 18.24 | 0.72 | 0.68 | 2.61 | 3.13 | 0.11 | 0.13 | -4.58 | 8.27E-32 | -4.48 | 1.64E-08 | 2131 |
| Cluster-40555.212201 | 10.13 | 11.43 | 0.24 | 0.58 | 12.44 | 11.51 | 0.38 | 0 | -4.57 | 4.17E-21 | -5.94 | 5.61E-31 | 2103 |
| Cluster-40555.153158 | 25.63 | 20.13 | 0.35 | 1.35 | 33.4 | 32.5 | 1.2 | 0.95 | -4.57 | 2.89E-05 | -4.88 | 1.79E-09 | 437 |

| | | | | | | | | | | | | | |
|---|---|---|---|---|---|---|---|---|---|---|---|---|---|
| Cluster-40555.198925 | 42.53 | 42.78 | 1.66 | 1.65 | 42.08 | 34.47 | 1.47 | 1.21 | -4.56 | 2.11E-29 | -4.76 | 8.28E-28 | 984 |
| Cluster-40555.119980 | 15.47 | 13.19 | 0 | 1.08 | 8.28 | 6.42 | 0.11 | 0.98 | -4.56 | 5.08E-16 | -3.70 | 3.74E-09 | 1294 |
| Cluster-40555.184466 | 23.23 | 27.02 | 0.74 | 1.2 | 6.58 | 4.71 | 1.75 | 0.53 | -4.55 | 8.18E-15 | -2.25 | 4.72E-02 | 758 |
| Cluster-40555.242131 | 11.85 | 15.5 | 0.46 | 0.64 | 21.54 | 15.28 | 1.83 | 2.84 | -4.55 | 8.47E-12 | -2.90 | 2.73E-09 | 961 |
| Cluster-40555.142017 | 80.07 | 78.78 | 1.84 | 4.29 | 40.52 | 34.3 | 4.6 | 4.12 | -4.55 | 5.97E-29 | -3.04 | 1.02E-10 | 662 |
| Cluster-40555.238745 | 96.27 | 89.68 | 2.55 | 4.62 | 53.78 | 58.43 | 3.56 | 4.73 | -4.55 | 2.84E-51 | -3.69 | 2.46E-26 | 1326 |
| Cluster-40555.161069 | 2.59 | 4.85 | 0.26 | 0.04 | 1.77 | 2.22 | 0 | 0.42 | -4.55 | 9.77E-04 | -3.17 | 1.00E-04 | 2391 |
| Cluster-40555.225751 | 2.22 | 1.49 | 0.14 | 0 | 1.53 | 1.27 | 0 | 0.01 | -4.54 | 1.66E-03 | 0.00 | 1.60E-04 | 1531 |
| Cluster-40555.293025 | 5.75 | 10.92 | 0.4 | 0.26 | 4.71 | 4.12 | 0.17 | 0.18 | -4.54 | 5.89E-03 | -4.61 | 2.13E-02 | 567 |
| Cluster-40555.168556 | 15.45 | 10.15 | 0.27 | 0.72 | 6.54 | 7.33 | 0 | 0.32 | -4.53 | 1.96E-11 | -5.22 | 1.12E-09 | 1013 |
| Cluster-40555.232156 | 3.86 | 2.82 | 0.19 | 0.08 | 1.59 | 1.44 | 0.22 | 0.33 | -4.53 | 3.11E-12 | -2.42 | 2.65E-03 | 3114 |
| Cluster-40555.181621 | 603.84 | 532.93 | 23.07 | 21.61 | 69.43 | 56.6 | 19.85 | 22.04 | -4.53 | 5.62E-75 | -1.52 | 8.13E-06 | 1283 |
| Cluster-40555.42900 | 1.69 | 1.91 | 0.06 | 0.08 | 4.59 | 4.5 | 0.1 | 0 | -4.52 | 1.47E-02 | -6.46 | 6.83E-10 | 1258 |
| Cluster-40555.274535 | 71.07 | 54.8 | 2.14 | 2.82 | 59.39 | 48.84 | 4.92 | 4.68 | -4.52 | 1.31E-43 | -3.43 | 8.08E-27 | 2291 |
| Cluster-40555.181607 | 161.74 | 82.07 | 30.14 | 5.28 | 25 | 14.97 | 5.75 | 5.53 | -4.51 | 1.45E-05 | -1.74 | 1.40E-02 | 1465 |
| Cluster-40555.187793 | 121.99 | 87.87 | 4.62 | 3.67 | 21.56 | 14.51 | 5.64 | 6.39 | -4.51 | 2.04E-27 | -1.51 | 2.33E-02 | 661 |
| Cluster-40555.84728 | 9.63 | 3.55 | 0.28 | 0.22 | 31.28 | 37.14 | 0.11 | 0.11 | -4.51 | 3.58E-02 | -8.73 | 3.96E-40 | 929 |
| Cluster-40555.146556 | 23.01 | 18.42 | 0.81 | 0.84 | 8.39 | 7.71 | 0.71 | 0.68 | -4.51 | 2.34E-20 | -3.44 | 6.81E-08 | 1168 |
| Cluster-40555.161377 | 85.8 | 92.66 | 3.86 | 3.32 | 2.3 | 2.39 | 0 | 0.44 | -4.50 | 1.61E-49 | -3.32 | 7.28E-03 | 1219 |
| Cluster-40555.304597 | 6.4 | 5.14 | 0.25 | 0.21 | 7.38 | 4.72 | 0.8 | 0.97 | -4.50 | 3.71E-12 | -2.69 | 1.24E-05 | 1965 |
| Cluster-40555.303659 | 28.21 | 28.51 | 1.3 | 0.99 | 8.58 | 5.14 | 1.47 | 1.42 | -4.50 | 3.59E-17 | -2.18 | 1.95E-02 | 783 |
| Cluster-40555.177876 | 55.49 | 62.35 | 2.07 | 2.71 | 6.42 | 6.24 | 1.49 | 2.17 | -4.49 | 9.34E-41 | -1.73 | 5.86E-03 | 1434 |
| Cluster-40555.183596 | 1.38 | 1.12 | 0.06 | 0.04 | 2.27 | 1.67 | 0 | 0 | -4.48 | 1.65E-03 | 0.00 | 3.50E-10 | 2251 |
| Cluster-40555.200046 | 2.3 | 1.07 | 0 | 0.14 | 2.53 | 1.56 | 0.45 | 0 | -4.48 | 4.90E-03 | -3.12 | 4.13E-04 | 2666 |
| Cluster-40555.165163 | 15.29 | 13.46 | 1.1 | 0.09 | 8.27 | 6.79 | 0.8 | 0.24 | -4.48 | 7.30E-09 | -3.81 | 1.26E-04 | 709 |
| Cluster-40555.297075 | 122.87 | 97.37 | 5.41 | 3.65 | 27.52 | 19.4 | 7.35 | 7.35 | -4.47 | 3.70E-37 | -1.60 | 4.06E-03 | 687 |
| Cluster-40555.188528 | 17.33 | 15.21 | 0 | 1.29 | 6.5 | 6.7 | 0.43 | 1.47 | -4.47 | 7.19E-15 | -2.73 | 8.30E-05 | 1117 |
| Cluster-40555.252337 | 3.51 | 3.61 | 0.18 | 0.12 | 2.67 | 2.23 | 0.37 | 0.15 | -4.46 | 1.87E-03 | -3.18 | 4.66E-02 | 949 |
| Cluster-40555.238888 | 89.91 | 69.95 | 3.55 | 3 | 13.23 | 15.21 | 3.63 | 5.09 | -4.46 | 1.52E-38 | -1.63 | 4.64E-03 | 905 |
| Cluster-40555.43229 | 3.01 | 2.41 | 0.06 | 0.17 | 1.23 | 1.34 | 0.03 | 0 | -4.46 | 7.11E-08 | -5.89 | 2.01E-06 | 2620 |
| Cluster-40555.195386 | 17.48 | 21.92 | 1.35 | 0.44 | 21.25 | 17.92 | 0 | 0.9 | -4.45 | 1.89E-05 | -5.46 | 2.27E-07 | 458 |
| Cluster-40555.129199 | 40.33 | 27.78 | 1.42 | 1.37 | 45.66 | 36.75 | 0.89 | 0.15 | -4.45 | 7.84E-14 | -6.26 | 2.96E-24 | 608 |

| | | | | | | | | | | | | | |
|---|---|---|---|---|---|---|---|---|---|---|---|---|---|
| Cluster-40555.212669 | 0.62 | 0.63 | 0 | 0.05 | 3.08 | 1.74 | 0.12 | 0 | -4.44 | 1.40E-02 | -5.38 | 7.73E-07 | 3380 |
| Cluster-40555.190239 | 24.4 | 16.69 | 1.22 | 0.53 | 26.14 | 22.58 | 2.13 | 1.4 | -4.44 | 2.31E-16 | -3.73 | 5.91E-22 | 1641 |
| Cluster-40555.272611 | 1.01 | 0.72 | 0.07 | 0 | 0.87 | 0.61 | 0 | 0 | -4.44 | 3.40E-02 | 0.00 | 4.86E-03 | 1913 |
| Cluster-40555.230137 | 4.55 | 2.73 | 0.29 | 0 | 3.02 | 3.28 | 0 | 0.22 | -4.43 | 3.89E-06 | -4.75 | 1.22E-06 | 1453 |
| Cluster-40555.178824 | 11.42 | 10.63 | 0.19 | 0.73 | 10.05 | 14.87 | 0.91 | 2.71 | -4.43 | 1.10E-15 | -2.70 | 1.29E-03 | 1575 |
| Cluster-40555.98080 | 19.17 | 26.47 | 0.71 | 1.22 | 13.63 | 12.12 | 1.96 | 3.24 | -4.42 | 1.02E-08 | -2.23 | 4.95E-04 | 782 |
| Cluster-40555.159078 | 44.68 | 46.24 | 1.39 | 2.46 | 11 | 9.62 | 4.09 | 1.45 | -4.42 | 2.56E-25 | -1.84 | 9.37E-03 | 855 |
| Cluster-40555.118010 | 1.57 | 1.34 | 0.11 | 0.02 | 1.16 | 0.96 | 0.05 | 0.19 | -4.42 | 4.07E-07 | -3.03 | 5.14E-04 | 3842 |
| Cluster-40555.211287 | 95.44 | 122.94 | 4.99 | 4.37 | 20.88 | 23.23 | 2.25 | 2.75 | -4.42 | 1.52E-14 | -3.08 | 7.27E-13 | 1157 |
| Cluster-40555.197310 | 41 | 48.86 | 1.77 | 2.04 | 11.65 | 11.29 | 2.67 | 2.96 | -4.41 | 3.19E-21 | -1.97 | 3.24E-03 | 818 |
| Cluster-40555.126644 | 15.32 | 19.42 | 0.62 | 0.86 | 41.35 | 42.41 | 0.59 | 0.83 | -4.41 | 1.06E-13 | -5.81 | 2.04E-35 | 952 |
| Cluster-40555.211274 | 13.42 | 9.6 | 0.81 | 0.2 | 2.75 | 2.52 | 0.26 | 0.38 | -4.41 | 9.73E-21 | -2.97 | 8.94E-05 | 2109 |
| Cluster-40555.165373 | 47.6 | 48.27 | 2.08 | 2.02 | 52.73 | 48.82 | 2.38 | 2.85 | -4.40 | 1.97E-10 | -4.23 | 1.38E-11 | 438 |
| Cluster-40555.131408 | 9.48 | 8.51 | 0.48 | 0.3 | 6.39 | 6.88 | 0.45 | 0.46 | -4.40 | 1.75E-23 | -3.79 | 4.62E-16 | 2819 |
| Cluster-40555.132071 | 6.5 | 5.71 | 0.27 | 0.26 | 6.06 | 7.61 | 1.32 | 1.47 | -4.39 | 3.40E-07 | -2.23 | 8.27E-04 | 1181 |
| Cluster-40555.189241 | 7.81 | 9.57 | 0.47 | 0.3 | 2.86 | 3.98 | 0.01 | 0.23 | -4.39 | 1.84E-20 | -4.72 | 1.43E-09 | 4265 |
| Cluster-40555.171943 | 25.16 | 24.94 | 1.37 | 0.78 | 13.04 | 13.19 | 0.13 | 0.57 | -4.39 | 1.03E-26 | -5.14 | 4.40E-21 | 1365 |
| Cluster-40555.190810 | 2.34 | 1.63 | 0 | 0.16 | 3.08 | 1.99 | 0.39 | 0.24 | -4.38 | 4.54E-04 | -2.91 | 4.44E-04 | 1922 |
| Cluster-40555.225256 | 5.77 | 3.25 | 0 | 0.39 | 1.29 | 0.97 | 0.09 | 0 | -4.38 | 4.21E-05 | -4.67 | 2.17E-02 | 1414 |
| Cluster-40555.261762 | 12.65 | 9.65 | 0.64 | 0.31 | 4.34 | 3.23 | 0 | 0.39 | -4.38 | 6.79E-13 | -4.21 | 9.76E-06 | 1221 |
| Cluster-40555.174285 | 37.98 | 28.81 | 1.88 | 1.08 | 10.86 | 10.23 | 2.21 | 1.28 | -4.38 | 1.32E-23 | -2.53 | 4.14E-05 | 964 |
| Cluster-40555.143899 | 31.38 | 37.64 | 1.47 | 1.67 | 11.8 | 9.61 | 0.3 | 0.78 | -4.37 | 8.97E-14 | -4.24 | 1.08E-05 | 606 |
| Cluster-40555.107302 | 3.96 | 2.97 | 0.12 | 0.2 | 5.38 | 4 | 0.66 | 0.73 | -4.36 | 9.81E-05 | -2.67 | 3.68E-04 | 1323 |
| Cluster-40555.36997 | 11.86 | 18.78 | 1.08 | 0.31 | 7.45 | 4.64 | 0.1 | 0.61 | -4.36 | 2.44E-05 | -3.98 | 1.21E-04 | 776 |
| Cluster-40555.42851 | 12.28 | 15.75 | 0.64 | 0.61 | 3.01 | 3.31 | 1.09 | 0.54 | -4.36 | 3.92E-14 | -1.91 | 2.23E-02 | 1702 |
| Cluster-40555.161941 | 7.99 | 10.42 | 0.35 | 0.5 | 4.04 | 2.47 | 0.92 | 0.2 | -4.35 | 5.42E-12 | -2.49 | 6.24E-03 | 1391 |
| Cluster-40555.245339 | 9.32 | 8.14 | 0.31 | 0.44 | 1.7 | 1.7 | 0.13 | 0.14 | -4.35 | 1.07E-14 | -3.58 | 1.86E-03 | 1774 |
| Cluster-40555.202442 | 224.79 | 301.78 | 13.75 | 9.88 | 47.22 | 60.97 | 11.55 | 17.54 | -4.35 | 7.88E-11 | -1.83 | 3.81E-04 | 691 |
| Cluster-40555.177766 | 252.72 | 199.63 | 9.45 | 10.58 | 54.74 | 47.02 | 14.52 | 13.95 | -4.35 | 1.06E-54 | -1.77 | 2.78E-06 | 880 |
| Cluster-40555.171032 | 60.56 | 60.05 | 1.59 | 3.73 | 38.2 | 40.36 | 1.61 | 4.02 | -4.35 | 9.85E-28 | -3.74 | 7.27E-18 | 803 |
| Cluster-40555.180915 | 1.46 | 3.24 | 0 | 0.19 | 1.56 | 1.89 | 0 | 0 | -4.34 | 4.31E-02 | 0.00 | 4.52E-05 | 1404 |
| Cluster-40555.187930 | 52.77 | 50.98 | 2.47 | 2.16 | 76.8 | 62.24 | 3.56 | 4.86 | -4.34 | 5.86E-28 | -3.97 | 3.67E-26 | 862 |

| | | | | | | | | | | | | | |
|---|---|---|---|---|---|---|---|---|---|---|---|---|---|
| Cluster-40555.175918 | 29.69 | 36.16 | 1.31 | 1.73 | 8.47 | 10.87 | 0.12 | 0 | -4.34 | 5.15E-16 | -7.37 | 5.91E-10 | 705 |
| Cluster-40555.192995 | 7.98 | 7.89 | 0.73 | 0 | 4.92 | 4.27 | 0.47 | 0.87 | -4.33 | 7.23E-13 | -2.70 | 8.26E-05 | 1523 |
| Cluster-40555.172251 | 3.7 | 4.75 | 0 | 0.37 | 14.71 | 18.18 | 0.69 | 2 | -4.33 | 2.20E-06 | -3.54 | 4.38E-13 | 1534 |
| Cluster-40555.275175 | 16.65 | 12.48 | 0.84 | 0.48 | 7.87 | 6.99 | 1.75 | 3 | -4.33 | 9.34E-25 | -1.57 | 1.46E-03 | 2016 |
| Cluster-40555.117167 | 13.3 | 7.07 | 0.53 | 0.37 | 4.87 | 4.84 | 0.41 | 0.37 | -4.33 | 5.20E-05 | -3.68 | 5.42E-05 | 1038 |
| Cluster-40555.224440 | 76.5 | 65.1 | 2.73 | 3.69 | 30.81 | 29.09 | 3.34 | 6.32 | -4.32 | 5.40E-41 | -2.56 | 2.02E-11 | 1193 |
| Cluster-40555.196897 | 36.44 | 35 | 1.06 | 2.16 | 62.65 | 49.22 | 2.75 | 0 | -4.32 | 8.03E-21 | -5.33 | 2.26E-34 | 862 |
| Cluster-40555.196388 | 34.47 | 28.87 | 1.57 | 1.3 | 17.84 | 15.72 | 0.78 | 0.3 | -4.32 | 1.34E-38 | -4.92 | 7.44E-30 | 2022 |
| Cluster-40555.132807 | 1.55 | 1.72 | 0 | 0.16 | 1.49 | 1.12 | 0.25 | 0 | -4.32 | 7.89E-03 | -3.29 | 3.26E-02 | 1625 |
| Cluster-40555.159569 | 10.7 | 6.77 | 0.69 | 0.13 | 4.31 | 5.22 | 1.07 | 1.23 | -4.31 | 4.54E-10 | -1.99 | 1.77E-03 | 1731 |
| Cluster-40555.139047 | 3.7 | 5.53 | 0 | 0.41 | 4.5 | 5.27 | 0.19 | 0.52 | -4.31 | 8.05E-06 | -3.69 | 5.86E-12 | 2550 |
| Cluster-40555.190055 | 6.13 | 7.27 | 0.38 | 0.24 | 221.29 | 253.07 | 0.05 | 0 | -4.31 | 9.11E-06 | -12.56 | 2.18E-88 | 918 |
| Cluster-40555.258412 | 1.16 | 1.61 | 0.11 | 0.04 | 1.51 | 1.41 | 0.18 | 0 | -4.31 | 1.61E-02 | -4.16 | 5.90E-03 | 1527 |
| Cluster-40555.40551 | 4.21 | 3.48 | 0.21 | 0.14 | 1.63 | 1.73 | 0 | 0 | -4.31 | 5.14E-03 | 0.00 | 2.27E-02 | 836 |
| Cluster-40555.235587 | 13.3 | 9.3 | 0 | 1.01 | 3.23 | 3.32 | 0.3 | 0.64 | -4.31 | 3.85E-14 | -2.72 | 1.93E-04 | 1838 |
| Cluster-40555.95242 | 10.59 | 17.38 | 0.03 | 1.26 | 9.72 | 13.85 | 0.28 | 0.15 | -4.30 | 7.15E-04 | -5.89 | 2.76E-09 | 667 |
| Cluster-40555.185000 | 170.41 | 160.96 | 6.92 | 8.29 | 27.4 | 19.61 | 10.17 | 4.72 | -4.30 | 3.06E-39 | -1.60 | 8.96E-03 | 625 |
| Cluster-40555.163335 | 8.68 | 5 | 0.32 | 0.31 | 3.78 | 2.61 | 0.01 | 0.28 | -4.29 | 2.05E-06 | -4.40 | 1.29E-07 | 1739 |
| Cluster-40555.277437 | 2.63 | 2.35 | 0.11 | 0.12 | 1.35 | 2.06 | 0 | 0 | -4.29 | 5.68E-04 | 0.00 | 2.40E-05 | 1484 |
| Cluster-40555.203146 | 12.24 | 8.88 | 0.27 | 0.71 | 17.54 | 14 | 4.59 | 2.69 | -4.29 | 7.50E-06 | -2.06 | 1.08E-03 | 719 |
| Cluster-40555.151242 | 57.16 | 48.86 | 1.32 | 3.59 | 32.48 | 26.77 | 3.17 | 3.58 | -4.28 | 3.36E-28 | -3.08 | 7.32E-13 | 926 |
| Cluster-40555.199647 | 6.06 | 4.52 | 0.24 | 0.26 | 2.49 | 2.19 | 0.3 | 0.24 | -4.28 | 1.22E-11 | -3.01 | 1.61E-04 | 2186 |
| Cluster-40555.204739 | 156.23 | 122.51 | 8.64 | 4.5 | 22.55 | 18.3 | 4.03 | 8.5 | -4.27 | 1.08E-51 | -1.62 | 2.40E-05 | 1661 |
| Cluster-40555.204623 | 25.45 | 35.44 | 1.12 | 1.77 | 8.51 | 10.82 | 1.73 | 1.9 | -4.26 | 2.78E-09 | -2.35 | 3.57E-07 | 2519 |
| Cluster-40555.202788 | 5.86 | 3.25 | 0.18 | 0.26 | 6.33 | 6.64 | 0.68 | 1.52 | -4.26 | 4.44E-05 | -2.47 | 6.57E-05 | 1352 |
| Cluster-40555.224079 | 43.06 | 32.93 | 1.62 | 2 | 23.27 | 13.11 | 0 | 2.17 | -4.26 | 3.99E-22 | -3.94 | 5.71E-05 | 881 |
| Cluster-40555.304996 | 10.89 | 12.96 | 0.24 | 0.88 | 1.53 | 1.1 | 0 | 0 | -4.26 | 2.73E-10 | 0.00 | 1.77E-02 | 1028 |
| Cluster-40555.183604 | 19.86 | 34.36 | 1.41 | 1.16 | 25.33 | 23.88 | 1.53 | 1.27 | -4.25 | 6.65E-04 | -4.10 | 5.85E-13 | 695 |
| Cluster-40555.158146 | 3.32 | 2.97 | 0 | 0.29 | 4.28 | 2.64 | 0.33 | 0.66 | -4.24 | 5.57E-05 | -2.72 | 3.86E-04 | 1614 |
| Cluster-40555.307766 | 2.16 | 1.28 | 0 | 0.16 | 1.22 | 1.99 | 0.1 | 0.24 | -4.24 | 1.49E-03 | -3.25 | 4.19E-03 | 2004 |
| Cluster-40555.195958 | 9.04 | 6.62 | 0.29 | 0.44 | 8.66 | 6.04 | 0.49 | 0.74 | -4.23 | 1.34E-13 | -3.49 | 1.72E-11 | 1872 |
| Cluster-40555.209904 | 43.4 | 47.5 | 2.76 | 1.64 | 38.86 | 46.04 | 2.47 | 2.08 | -4.23 | 1.16E-35 | -4.17 | 1.52E-24 | 1323 |

| | | | | | | | | | | | | | |
|---|---|---|---|---|---|---|---|---|---|---|---|---|---|
| Cluster-40555.195923 | 11.19 | 17.97 | 1 | 0.45 | 12.89 | 22.09 | 1.12 | 0.17 | -4.22 | 2.66E-05 | -4.77 | 1.36E-04 | 1543 |
| Cluster-40555.83544 | 8.95 | 9.37 | 0.63 | 0.28 | 4.01 | 3.69 | 0.35 | 0.46 | -4.21 | 6.33E-16 | -3.17 | 3.72E-06 | 1830 |
| Cluster-40555.296084 | 1.53 | 1.65 | 0.13 | 0.04 | 2.79 | 4.13 | 0.75 | 0 | -4.21 | 2.61E-02 | -3.21 | 1.59E-03 | 1287 |
| Cluster-40555.183666 | 35.33 | 28.5 | 1.53 | 1.6 | 12.01 | 10.77 | 0.65 | 0.72 | -4.21 | 1.60E-23 | -3.96 | 2.48E-11 | 1075 |
| Cluster-40555.301525 | 13.44 | 10.05 | 0.42 | 0.72 | 9.81 | 6.68 | 0 | 0.89 | -4.21 | 3.45E-06 | -4.19 | 1.24E-05 | 702 |
| Cluster-40555.199840 | 5.14 | 4.22 | 0.18 | 0.34 | 11.39 | 10.79 | 0.15 | 0.15 | -4.20 | 2.02E-02 | -6.34 | 1.91E-09 | 659 |
| Cluster-40555.125626 | 3.2 | 2.76 | 0.25 | 0.05 | 24.25 | 24.85 | 0.94 | 1.32 | -4.20 | 4.24E-03 | -4.37 | 2.77E-20 | 1015 |
| Cluster-40555.131755 | 18.6 | 17.86 | 0.65 | 1.12 | 9.43 | 9.43 | 0.64 | 0.39 | -4.20 | 5.69E-10 | -4.08 | 1.43E-06 | 745 |
| Cluster-40555.164519 | 4.81 | 3.33 | 0 | 0.38 | 1.28 | 1.14 | 0.11 | 0 | -4.20 | 1.11E-10 | -4.32 | 6.14E-05 | 2805 |
| Cluster-40555.170875 | 6.43 | 4.64 | 0.24 | 0.3 | 3.76 | 3.8 | 0.92 | 0.5 | -4.20 | 7.83E-11 | -2.35 | 3.28E-04 | 2002 |
| Cluster-40555.198125 | 34.51 | 24.58 | 1.32 | 1.55 | 12.83 | 10.74 | 1.63 | 1.14 | -4.19 | 5.57E-20 | -3.04 | 8.40E-09 | 1178 |
| Cluster-40555.214553 | 16.6 | 17.76 | 0.99 | 0.74 | 8.54 | 7.14 | 0.18 | 1.28 | -4.19 | 5.07E-24 | -3.32 | 6.76E-11 | 1775 |
| Cluster-40555.305937 | 1.95 | 1.69 | 0.11 | 0.07 | 2.27 | 2.52 | 0.18 | 0.33 | -4.19 | 1.02E-02 | -3.16 | 4.17E-03 | 1375 |
| Cluster-40555.179299 | 4.43 | 5.06 | 0.31 | 0.17 | 12.24 | 14.51 | 0.45 | 0.8 | -4.18 | 5.81E-04 | -4.29 | 1.10E-11 | 898 |
| Cluster-40555.204966 | 78.77 | 95.77 | 5.71 | 3.17 | 26.37 | 24.74 | 0.94 | 1.8 | -4.18 | 2.26E-18 | -4.11 | 3.09E-15 | 780 |
| Cluster-40555.253678 | 56.6 | 60.91 | 2.47 | 3.39 | 28.6 | 34.51 | 5 | 4.46 | -4.18 | 1.03E-12 | -2.69 | 7.96E-05 | 460 |
| Cluster-40555.182881 | 17.29 | 18.77 | 1.48 | 0.36 | 23.32 | 16.3 | 0.46 | 0.97 | -4.17 | 1.19E-07 | -4.71 | 1.72E-10 | 594 |
| Cluster-40555.203367 | 4.03 | 3.96 | 0.27 | 0.13 | 9.96 | 10.02 | 0.05 | 0.23 | -4.17 | 1.95E-05 | -6.00 | 2.42E-19 | 1318 |
| Cluster-40555.200728 | 4.42 | 4.96 | 0.29 | 0.18 | 3.9 | 2.72 | 0 | 0.38 | -4.17 | 1.11E-04 | -3.91 | 7.87E-04 | 1026 |
| Cluster-40555.108354 | 8.11 | 8.82 | 0.43 | 0.39 | 4.75 | 4.93 | 0.81 | 0.04 | -4.17 | 6.27E-06 | -3.55 | 1.63E-03 | 829 |
| Cluster-40555.210671 | 1.25 | 1.61 | 0 | 0.15 | 1.06 | 1.78 | 0 | 0 | -4.17 | 6.16E-03 | 0.00 | 1.67E-05 | 1944 |
| Cluster-40555.248993 | 2.11 | 2.52 | 0 | 0.23 | 3.35 | 2.4 | 0.12 | 0.23 | -4.17 | 2.62E-06 | -3.92 | 3.86E-09 | 2686 |
| Cluster-40555.201639 | 182.41 | 185.9 | 6.86 | 11.78 | 202.9 | 198.27 | 8.36 | 2.89 | -4.16 | 1.85E-50 | -5.11 | 3.46E-51 | 1020 |
| Cluster-40555.226144 | 61.99 | 54.84 | 4.41 | 1.58 | 115.21 | 68.94 | 2.72 | 7.45 | -4.16 | 7.50E-26 | -4.09 | 3.08E-07 | 747 |
| Cluster-40555.46543 | 13.95 | 11 | 0 | 1.23 | 11.03 | 10.78 | 0.16 | 1.67 | -4.15 | 1.39E-04 | -3.49 | 2.09E-04 | 583 |
| Cluster-40555.245340 | 5.23 | 5.74 | 0.23 | 0.33 | 1.86 | 1.86 | 0.17 | 0.23 | -4.15 | 4.66E-08 | -3.12 | 9.73E-03 | 1517 |
| Cluster-40555.241915 | 25.29 | 19.47 | 1.03 | 1.26 | 24.25 | 23.7 | 4.78 | 5.01 | -4.15 | 5.47E-27 | -2.23 | 8.38E-10 | 1755 |
| Cluster-40555.183761 | 6.54 | 4.02 | 0.2 | 0.33 | 5.69 | 3.59 | 0.31 | 0.49 | -4.14 | 2.72E-02 | -3.49 | 3.99E-02 | 602 |
| Cluster-40555.200468 | 61.85 | 61.26 | 3.37 | 3.03 | 12.41 | 14.5 | 4.37 | 1.44 | -4.14 | 7.57E-23 | -2.17 | 3.17E-03 | 670 |
| Cluster-40555.205156 | 30.32 | 33.29 | 0 | 3.25 | 90.52 | 99.2 | 2.34 | 4.72 | -4.12 | 1.14E-10 | -4.68 | 8.72E-29 | 591 |
| Cluster-40555.158169 | 245.17 | 216.38 | 14.39 | 9.85 | 339.69 | 208.31 | 0.57 | 26.63 | -4.11 | 3.47E-52 | -4.22 | 3.17E-08 | 882 |
| Cluster-40555.198245 | 34.33 | 32.97 | 1.67 | 1.84 | 9.87 | 6.78 | 0.38 | 0.34 | -4.11 | 3.08E-24 | -4.43 | 2.05E-10 | 1101 |

| | | | | | | | | | | | | | |
|---|---|---|---|---|---|---|---|---|---|---|---|---|---|
| Cluster-40555.153795 | 8.87 | 16.82 | 0.64 | 0.72 | 28.95 | 18.12 | 0.13 | 0.42 | -4.11 | 7.36E-03 | -6.33 | 2.34E-12 | 645 |
| Cluster-40555.111039 | 23.14 | 15.02 | 1.38 | 0.68 | 17.66 | 11.04 | 1.25 | 2.3 | -4.11 | 5.02E-11 | -2.94 | 1.77E-05 | 1041 |
| Cluster-40555.184703 | 48.13 | 28.11 | 1.13 | 2.8 | 25.9 | 22.69 | 6.74 | 6.38 | -4.10 | 1.21E-06 | -1.83 | 1.05E-03 | 653 |
| Cluster-40555.45815 | 5.71 | 4.91 | 0.62 | 0 | 4.84 | 3.53 | 0.63 | 0.54 | -4.10 | 1.31E-04 | -2.73 | 1.38E-02 | 903 |
| Cluster-40555.115089 | 108.61 | 90.12 | 4.72 | 5.91 | 23.04 | 19.75 | 5.92 | 8.3 | -4.08 | 8.17E-44 | -1.51 | 3.98E-04 | 1216 |
| Cluster-40555.203611 | 18.12 | 10.02 | 0.4 | 1.1 | 9.59 | 7.14 | 1.29 | 0.63 | -4.07 | 4.69E-05 | -3.09 | 5.03E-07 | 1179 |
| Cluster-40555.191820 | 41.77 | 30.22 | 1.31 | 2.53 | 27.18 | 23.69 | 2.98 | 3.32 | -4.07 | 1.03E-20 | -2.94 | 3.06E-16 | 1806 |
| Cluster-40555.125090 | 84.82 | 83.35 | 3.83 | 5.26 | 14.09 | 13.52 | 2.73 | 2.24 | -4.07 | 7.87E-31 | -2.41 | 1.10E-04 | 783 |
| Cluster-40555.132852 | 1.58 | 2.84 | 0.12 | 0.12 | 1.82 | 2.33 | 0.16 | 0.31 | -4.07 | 6.80E-03 | -3.09 | 2.00E-02 | 1265 |
| Cluster-40555.302592 | 25.84 | 26.94 | 1.33 | 1.54 | 83.06 | 71.12 | 3.75 | 8.22 | -4.06 | 7.72E-13 | -3.61 | 1.41E-21 | 725 |
| Cluster-40555.195163 | 2.06 | 1.93 | 0 | 0.23 | 3.55 | 2.45 | 0.15 | 0.1 | -4.06 | 2.99E-02 | -4.40 | 1.11E-04 | 1200 |
| Cluster-40555.237462 | 0.88 | 0.92 | 0 | 0.09 | 1.05 | 0.42 | 0.05 | 0 | -4.05 | 3.01E-02 | -4.88 | 3.54E-02 | 2338 |
| Cluster-40555.231110 | 3.28 | 1.6 | 0.08 | 0.19 | 1.56 | 1.12 | 0.07 | 0 | -4.05 | 6.74E-03 | -5.69 | 8.37E-03 | 1264 |
| Cluster-40555.231574 | 3.35 | 2.4 | 0.25 | 0.08 | 1.83 | 1.48 | 0.22 | 0.23 | -4.05 | 1.58E-05 | -2.76 | 1.56E-02 | 1807 |
| Cluster-40555.160945 | 2.01 | 2.3 | 0 | 0.24 | 1.75 | 0.77 | 0 | 0 | -4.05 | 1.22E-02 | 0.00 | 3.31E-03 | 1289 |
| Cluster-40555.207611 | 3.46 | 3.42 | 0 | 0.37 | 5.63 | 3.48 | 0.36 | 0.36 | -4.05 | 2.76E-05 | -3.56 | 2.34E-06 | 1665 |
| Cluster-40555.180785 | 2.51 | 1.17 | 0 | 0.19 | 1.66 | 1.31 | 0.06 | 0 | -4.05 | 3.26E-02 | -5.86 | 3.26E-03 | 1278 |
| Cluster-40555.214817 | 1.85 | 1.16 | 0.11 | 0.07 | 1.1 | 0.83 | 0.12 | 0.14 | -4.05 | 1.53E-04 | -2.77 | 2.11E-02 | 2719 |
| Cluster-40555.265908 | 4.05 | 5.17 | 0.47 | 0 | 4.15 | 3.01 | 0 | 0 | -4.05 | 4.74E-03 | 0.00 | 2.57E-04 | 736 |
| Cluster-40555.205474 | 19.5 | 22.44 | 1.28 | 1.07 | 8.7 | 9.14 | 1.57 | 1.68 | -4.04 | 2.44E-12 | -2.37 | 1.93E-03 | 810 |
| Cluster-40555.102720 | 0.77 | 1.19 | 0 | 0.11 | 1.83 | 1.49 | 0.19 | 0.03 | -4.04 | 3.13E-02 | -3.91 | 1.69E-04 | 2136 |
| Cluster-40555.250830 | 1.67 | 1.65 | 0.11 | 0.08 | 2.87 | 3.24 | 0.23 | 0 | -4.04 | 1.80E-03 | -4.81 | 1.38E-08 | 1909 |
| Cluster-40555.117052 | 6.56 | 4.06 | 0.37 | 0.18 | 2.34 | 1.39 | 0 | 0.31 | -4.04 | 3.83E-05 | -3.42 | 4.38E-02 | 1059 |
| Cluster-40555.259822 | 17.13 | 18.33 | 0.4 | 1.54 | 25.86 | 34.69 | 0.69 | 2.58 | -4.03 | 3.05E-03 | -4.17 | 1.15E-06 | 415 |
| Cluster-40555.165856 | 55.29 | 42.68 | 2.28 | 2.95 | 60.86 | 68.69 | 6.44 | 1.2 | -4.02 | 4.09E-03 | -4.06 | 1.51E-04 | 307 |
| Cluster-40555.187888 | 19.02 | 16.55 | 0.72 | 1.25 | 10.9 | 8.29 | 1.94 | 1.22 | -4.01 | 2.22E-33 | -2.54 | 2.18E-11 | 3324 |
| Cluster-40555.235666 | 27.53 | 31.14 | 2.25 | 1.08 | 42.29 | 37.14 | 2.3 | 6.42 | -4.01 | 1.60E-23 | -3.10 | 2.87E-17 | 1167 |
| Cluster-40555.193768 | 60.87 | 58.34 | 3.9 | 2.88 | 11.06 | 11.85 | 2.32 | 2.2 | -4.00 | 2.38E-44 | -2.27 | 1.01E-07 | 1895 |
| Cluster-40555.222571 | 5.7 | 3.73 | 0.39 | 0.15 | 1.92 | 2.51 | 0.28 | 0.43 | -4.00 | 3.28E-06 | -2.59 | 3.05E-02 | 1359 |
| Cluster-40555.196571 | 108.66 | 72.22 | 5.63 | 4.62 | 19.31 | 16.89 | 4.44 | 5.36 | -4.00 | 1.15E-12 | -1.82 | 1.31E-05 | 1404 |
| Cluster-40555.220330 | 1.54 | 1.05 | 0.07 | 0.08 | 1.15 | 1.3 | 0.05 | 0.03 | -3.99 | 4.99E-03 | -4.89 | 2.29E-04 | 2178 |
| Cluster-40555.195541 | 2.67 | 2.74 | 0.11 | 0.19 | 3.87 | 3.22 | 0.2 | 0.21 | -3.98 | 1.01E-04 | -4.04 | 1.71E-07 | 1755 |

| | | | | | | | | | | | | | |
|---|---|---|---|---|---|---|---|---|---|---|---|---|---|
| Cluster-40555.154389 | 58.25 | 38.65 | 2.31 | 3.2 | 186.24 | 149.6 | 11.49 | 8.36 | -3.98 | 4.01E-12 | -4.01 | 2.69E-35 | 1034 |
| Cluster-40555.101206 | 5.32 | 6.18 | 0.35 | 0.3 | 5.7 | 3.75 | 0.15 | 0.16 | -3.98 | 1.18E-04 | -4.87 | 9.94E-06 | 929 |
| Cluster-81817.0 | 3.6 | 3.97 | 0.33 | 0.11 | 3.57 | 3.56 | 0.34 | 0.57 | -3.98 | 1.40E-03 | -2.89 | 9.69E-03 | 998 |
| Cluster-40555.184815 | 10.68 | 11.7 | 0.42 | 0.85 | 7.7 | 8.43 | 0.76 | 0.93 | -3.97 | 9.98E-09 | -3.19 | 4.73E-06 | 995 |
| Cluster-40555.177342 | 53.32 | 55.93 | 2.93 | 3.41 | 140.07 | 104.29 | 7.5 | 12.89 | -3.97 | 4.17E-29 | -3.50 | 6.26E-22 | 1019 |
| Cluster-40555.204769 | 3.89 | 6.9 | 0.24 | 0.38 | 11.63 | 10.48 | 0.45 | 0.31 | -3.97 | 1.91E-03 | -4.78 | 7.68E-25 | 2242 |
| Cluster-40555.43125 | 3.2 | 2.19 | 0.25 | 0.07 | 1.68 | 1.26 | 0.06 | 0 | -3.96 | 3.25E-07 | -5.43 | 1.38E-06 | 2522 |
| Cluster-40555.140420 | 26.43 | 26.09 | 0.98 | 2.06 | 62.22 | 39.24 | 7.44 | 7.38 | -3.95 | 8.45E-13 | -2.70 | 1.64E-05 | 762 |
| Cluster-40555.198850 | 7.43 | 7.2 | 0.51 | 0.33 | 1.97 | 0.89 | 0.14 | 0.05 | -3.95 | 7.70E-12 | -3.78 | 2.36E-02 | 1791 |
| Cluster-40555.227679 | 0.81 | 0.81 | 0 | 0.1 | 0.72 | 0.44 | 0 | 0 | -3.95 | 4.74E-02 | 0.00 | 3.66E-03 | 2424 |
| Cluster-40555.181005 | 38.97 | 33.69 | 2.48 | 1.79 | 59.07 | 62.87 | 6.38 | 2.06 | -3.95 | 1.45E-31 | -3.81 | 3.44E-29 | 1550 |
| Cluster-40555.281666 | 8.57 | 6.82 | 0.61 | 0.29 | 3.59 | 2.45 | 0.27 | 0.16 | -3.95 | 1.44E-08 | -3.79 | 3.57E-04 | 1243 |
| Cluster-40555.218088 | 12.33 | 20.2 | 0 | 1.9 | 7.4 | 8.31 | 0.98 | 0 | -3.94 | 9.30E-04 | -3.94 | 8.96E-08 | 1017 |
| Cluster-40555.195550 | 16.72 | 16.47 | 0.99 | 1.07 | 9.18 | 7.58 | 1.87 | 1.32 | -3.94 | 2.08E-08 | -2.31 | 8.85E-03 | 726 |
| Cluster-40555.137893 | 7.44 | 5.7 | 0.55 | 0.23 | 7.63 | 6.02 | 0 | 2.16 | -3.94 | 7.04E-12 | -2.54 | 3.06E-05 | 1954 |
| Cluster-40555.269679 | 37.53 | 23.77 | 1.8 | 1.77 | 26.42 | 24.75 | 2.21 | 2.72 | -3.94 | 2.24E-09 | -3.32 | 2.24E-14 | 1030 |
| Cluster-40555.166461 | 46.25 | 51.3 | 1.97 | 3.82 | 43.39 | 33.29 | 2.7 | 2.33 | -3.94 | 1.02E-18 | -3.84 | 3.16E-16 | 708 |
| Cluster-40555.206258 | 3.87 | 5.43 | 0.26 | 0.3 | 1.88 | 2.78 | 0.09 | 0 | -3.93 | 2.45E-07 | -5.64 | 2.99E-07 | 1895 |
| Cluster-40555.107301 | 0.67 | 0.92 | 0 | 0.1 | 2.23 | 3.13 | 0.54 | 0.63 | -3.92 | 4.82E-03 | -2.15 | 1.08E-03 | 3652 |
| Cluster-40555.199938 | 8.42 | 3.65 | 0.6 | 0.1 | 1.74 | 2.28 | 0 | 0.19 | -3.92 | 9.39E-03 | -4.34 | 1.07E-04 | 1666 |
| Cluster-40555.202845 | 17.36 | 25.33 | 0.81 | 1.71 | 15.05 | 14.08 | 2.1 | 1.89 | -3.91 | 8.77E-06 | -2.80 | 1.89E-05 | 715 |
| Cluster-40555.158744 | 36.03 | 32.88 | 2.98 | 1.19 | 21.65 | 17.95 | 2.71 | 2.43 | -3.91 | 3.80E-10 | -2.88 | 9.70E-05 | 529 |
| Cluster-40555.210511 | 445.54 | 528.37 | 44.97 | 14.57 | 940.27 | 1065.41 | 19.89 | 12.4 | -3.91 | 2.10E-20 | -5.91 | 1.65E-60 | 1013 |
| Cluster-40555.232142 | 8.85 | 8.6 | 0.65 | 0.38 | 9.5 | 11.61 | 3.28 | 1.42 | -3.91 | 4.82E-08 | -2.13 | 2.45E-04 | 1083 |
| Cluster-40555.194665 | 25.44 | 27.72 | 1.06 | 2.15 | 4.38 | 3.43 | 0 | 1.12 | -3.90 | 2.42E-27 | -2.68 | 5.79E-05 | 1779 |
| Cluster-40555.207792 | 23.97 | 20.14 | 1.64 | 1.07 | 4.67 | 3.68 | 0.39 | 0.86 | -3.90 | 6.36E-16 | -2.67 | 6.66E-03 | 1017 |
| Cluster-40555.200103 | 10.82 | 12.93 | 0.91 | 0.59 | 2.43 | 1.5 | 0.42 | 0 | -3.90 | 2.43E-15 | -3.18 | 5.55E-03 | 1581 |
| Cluster-40555.157578 | 23.41 | 17.46 | 1.26 | 1.21 | 7.24 | 5.14 | 0.8 | 1.1 | -3.89 | 1.36E-15 | -2.64 | 3.56E-04 | 1082 |
| Cluster-40555.302765 | 13.34 | 12.79 | 1.01 | 0.62 | 4.19 | 3.1 | 0.32 | 0.92 | -3.89 | 5.54E-11 | -2.47 | 1.79E-02 | 1057 |
| Cluster-40555.100373 | 2.28 | 2.63 | 0.2 | 0.09 | 8.34 | 7.82 | 0.13 | 0.1 | -3.89 | 1.20E-03 | -6.20 | 1.41E-18 | 1469 |
| Cluster-40555.190609 | 109.08 | 106.31 | 5.6 | 7.51 | 33.41 | 40.07 | 12.45 | 8.99 | -3.89 | 4.52E-37 | -1.72 | 2.13E-05 | 943 |
| Cluster-40555.115087 | 94.68 | 70.4 | 4.1 | 5.89 | 22.93 | 18.41 | 3.93 | 6.39 | -3.89 | 2.60E-26 | -1.92 | 7.75E-06 | 1131 |

| | | | | | | | | | | | | | |
|---|---|---|---|---|---|---|---|---|---|---|---|---|---|
| Cluster-40555.139874 | 513.51 | 530.24 | 30.56 | 33.1 | 750.73 | 581.59 | 68.4 | 80.33 | -3.89 | 7.03E-48 | -3.10 | 1.99E-24 | 555 |
| Cluster-40555.171060 | 5.87 | 6.8 | 0.48 | 0.38 | 5.43 | 5.18 | 0.78 | 0 | -3.88 | 2.32E-03 | -3.67 | 3.66E-03 | 700 |
| Cluster-40555.199654 | 32.74 | 26.99 | 3.76 | 0 | 17.66 | 14.16 | 2.06 | 2.42 | -3.88 | 1.51E-22 | -2.76 | 1.54E-08 | 1067 |
| Cluster-40555.194802 | 81.58 | 48.28 | 4.84 | 3.14 | 59.56 | 49.26 | 1.72 | 2.1 | -3.88 | 5.46E-07 | -4.76 | 1.17E-35 | 1166 |
| Cluster-40555.213732 | 9.27 | 8.88 | 0.25 | 0.84 | 1.26 | 2.03 | 0.08 | 0.23 | -3.87 | 1.08E-13 | -3.21 | 4.09E-03 | 1934 |
| Cluster-40555.133903 | 10.04 | 4.7 | 0.58 | 0.29 | 6.69 | 5.22 | 1.56 | 0.59 | -3.86 | 3.99E-03 | -2.41 | 4.66E-03 | 951 |
| Cluster-40555.202205 | 5.32 | 4.43 | 0.17 | 0.42 | 5.4 | 3.99 | 0.9 | 0.76 | -3.86 | 4.78E-11 | -2.43 | 1.90E-06 | 2626 |
| Cluster-40555.189690 | 138.35 | 148.4 | 10.56 | 7.49 | 129.15 | 122.31 | 17.41 | 12.47 | -3.86 | 1.70E-36 | -3.01 | 8.08E-19 | 728 |
| Cluster-40555.196681 | 8.93 | 14.12 | 0.88 | 0.62 | 4.74 | 4.62 | 0.13 | 0 | -3.85 | 1.17E-04 | -5.87 | 2.34E-06 | 911 |
| Cluster-40555.202059 | 2.9 | 3.08 | 0.33 | 0.04 | 1.82 | 2.48 | 0.31 | 0.13 | -3.84 | 6.26E-06 | -3.27 | 4.78E-04 | 1901 |
| Cluster-40555.239173 | 23.81 | 22.3 | 1.34 | 1.6 | 14.34 | 11.95 | 1.74 | 2.53 | -3.82 | 8.22E-14 | -2.55 | 8.87E-06 | 905 |
| Cluster-40555.144349 | 27.95 | 30.29 | 1.55 | 2.19 | 1.96 | 1.9 | 0.15 | 0.22 | -3.82 | 1.99E-21 | -3.27 | 2.60E-02 | 1209 |
| Cluster-40555.41980 | 1.73 | 1.53 | 0.22 | 0 | 3.37 | 5.43 | 0 | 0.03 | -3.81 | 1.39E-03 | -8.09 | 4.96E-08 | 1969 |
| Cluster-40555.91439 | 6.69 | 4.28 | 0 | 0.68 | 1.49 | 2.21 | 0 | 0.18 | -3.81 | 1.87E-04 | -4.17 | 1.67E-02 | 1069 |
| Cluster-40555.25105 | 1.46 | 1.38 | 0.09 | 0.09 | 1.95 | 1.1 | 0.23 | 0.2 | -3.80 | 2.86E-02 | -2.75 | 4.69E-02 | 1575 |
| Cluster-40555.188505 | 3.71 | 1.78 | 0 | 0.35 | 3.06 | 2.1 | 0 | 0 | -3.80 | 1.32E-02 | 0.00 | 6.55E-07 | 1296 |
| Cluster-40555.94793 | 0.93 | 1.8 | 0.18 | 0 | 2.45 | 1.74 | 0.2 | 0.16 | -3.80 | 1.74E-02 | -3.56 | 9.48E-04 | 1610 |
| Cluster-40555.109822 | 10.81 | 10.58 | 0.82 | 0.59 | 9.26 | 8.43 | 1.43 | 1.14 | -3.80 | 1.43E-15 | -2.71 | 9.91E-09 | 1840 |
| Cluster-40555.211514 | 78.94 | 73.29 | 6.17 | 3.76 | 24.09 | 19.17 | 1.62 | 4.96 | -3.80 | 1.21E-25 | -2.62 | 8.84E-07 | 721 |
| Cluster-40555.189228 | 95.78 | 100.3 | 8.16 | 4.77 | 36.79 | 39.4 | 3.37 | 4 | -3.79 | 2.03E-39 | -3.30 | 4.73E-18 | 1113 |
| Cluster-40555.197707 | 28.76 | 17.91 | 1.04 | 1.98 | 5.93 | 4.35 | 0.31 | 0.3 | -3.79 | 9.93E-08 | -4.00 | 7.04E-11 | 1928 |
| Cluster-40555.244327 | 23.13 | 27.61 | 2 | 1.49 | 3.09 | 5.41 | 0 | 0 | -3.79 | 2.85E-06 | 0.00 | 2.88E-02 | 484 |
| Cluster-40555.214246 | 2.57 | 1.5 | 0.26 | 0 | 1.41 | 1.1 | 0.35 | 0 | -3.79 | 1.06E-05 | -2.86 | 1.57E-03 | 3212 |
| Cluster-40555.26912 | 9.74 | 14.23 | 0.55 | 0.85 | 11.06 | 9.97 | 0 | 0.37 | -3.78 | 1.52E-02 | -5.99 | 7.22E-04 | 438 |
| Cluster-40555.176313 | 1.53 | 2.15 | 0 | 0.22 | 1.52 | 1.55 | 0 | 0 | -3.78 | 4.46E-02 | 0.00 | 7.87E-04 | 1255 |
| Cluster-40555.184430 | 4.83 | 8.64 | 0.4 | 0.47 | 5.04 | 5.8 | 0 | 0.17 | -3.78 | 4.50E-03 | -6.17 | 1.07E-08 | 1003 |
| Cluster-40555.213621 | 15.49 | 8.53 | 0.88 | 0.66 | 6.26 | 5.56 | 0.66 | 0.03 | -3.78 | 6.89E-05 | -4.03 | 2.68E-10 | 1624 |
| Cluster-40555.206014 | 79.79 | 84.48 | 11.17 | 0 | 49.04 | 34.4 | 3.22 | 12.75 | -3.78 | 5.48E-09 | -2.33 | 9.55E-03 | 354 |
| Cluster-40555.112068 | 6.17 | 10.14 | 0.22 | 0.86 | 7.99 | 14.01 | 0.37 | 0.78 | -3.77 | 1.36E-02 | -4.20 | 2.85E-03 | 539 |
| Cluster-40555.146480 | 1.99 | 1.24 | 0.07 | 0.17 | 1.38 | 1.09 | 0.06 | 0 | -3.77 | 3.02E-02 | -5.86 | 3.26E-03 | 1485 |
| Cluster-40555.58780 | 6.26 | 4.78 | 0.39 | 0.31 | 4.06 | 4.05 | 0.47 | 1.13 | -3.76 | 2.47E-05 | -2.27 | 1.37E-02 | 1138 |
| Cluster-40555.41746 | 11.71 | 8.01 | 0.44 | 0.85 | 9.29 | 9.93 | 0.55 | 1.74 | -3.76 | 4.88E-03 | -3.00 | 7.01E-03 | 541 |

| | | | | | | | | | | | | | |
|---|---|---|---|---|---|---|---|---|---|---|---|---|---|
| Cluster-40555.241919 | 16.7 | 13.95 | 1.15 | 0.82 | 18.08 | 15.07 | 3.84 | 7.4 | -3.76 | 3.36E-08 | -1.48 | 1.52E-02 | 774 |
| Cluster-40555.178140 | 7.86 | 5.27 | 0.61 | 0.29 | 3.59 | 3.72 | 1.05 | 1.5 | -3.76 | 2.50E-11 | -1.44 | 2.53E-02 | 2408 |
| Cluster-40555.195150 | 24.41 | 23.04 | 1.94 | 1.27 | 7.85 | 5.05 | 0.19 | 0 | -3.76 | 3.65E-19 | -5.90 | 6.11E-12 | 1226 |
| Cluster-40555.144403 | 55.05 | 52.59 | 2.63 | 4.55 | 16.43 | 11.64 | 4.76 | 3.37 | -3.76 | 5.30E-22 | -1.73 | 4.97E-03 | 828 |
| Cluster-40555.214281 | 2.93 | 3.52 | 0.29 | 0.12 | 1.77 | 2.62 | 0.17 | 0 | -3.76 | 6.05E-03 | -5.00 | 4.00E-03 | 1007 |
| Cluster-40555.188340 | 3.84 | 3.85 | 0.36 | 0.17 | 3.94 | 3.98 | 0.82 | 0.13 | -3.75 | 3.22E-07 | -3.03 | 3.52E-06 | 1973 |
| Cluster-40555.123009 | 6.19 | 7.02 | 0.31 | 0.59 | 4.07 | 4.32 | 0.59 | 0.71 | -3.75 | 1.25E-05 | -2.66 | 4.61E-03 | 1075 |
| Cluster-40555.202779 | 3.28 | 4.8 | 0.52 | 0.07 | 6.08 | 6.96 | 0.39 | 0.64 | -3.75 | 1.10E-03 | -3.55 | 7.80E-06 | 991 |
| Cluster-40555.221988 | 2.68 | 1.47 | 0.27 | 0 | 0.97 | 0.73 | 0 | 0.19 | -3.74 | 2.14E-04 | -3.09 | 4.62E-02 | 2211 |
| Cluster-40555.276157 | 1.64 | 1.61 | 0.25 | 0 | 0.94 | 1.93 | 0 | 0 | -3.74 | 2.45E-02 | 0.00 | 3.57E-03 | 1351 |
| Cluster-40555.91607 | 9.49 | 7.39 | 0.53 | 0.6 | 10.16 | 9.36 | 1.07 | 0.44 | -3.73 | 3.30E-06 | -3.62 | 5.87E-08 | 966 |
| Cluster-40555.193162 | 73.03 | 72.75 | 4.79 | 5.17 | 8.13 | 7.88 | 2.14 | 0.98 | -3.73 | 1.08E-24 | -2.29 | 9.89E-03 | 753 |
| Cluster-40555.146344 | 207.33 | 177.09 | 9.64 | 16.35 | 522.66 | 402.76 | 57.29 | 52.86 | -3.73 | 8.61E-42 | -3.00 | 5.07E-24 | 939 |
| Cluster-40555.185637 | 7.29 | 11.46 | 0.17 | 1.09 | 12.54 | 16.71 | 1.05 | 0.57 | -3.73 | 2.56E-04 | -4.15 | 6.14E-11 | 2303 |
| Cluster-40555.226430 | 3.78 | 4.07 | 0.24 | 0.29 | 2.08 | 1.96 | 0.24 | 0.16 | -3.73 | 7.62E-08 | -3.32 | 1.36E-04 | 2221 |
| Cluster-40555.166361 | 26.44 | 20.46 | 1.1 | 2.08 | 11.93 | 12.02 | 1.21 | 0.39 | -3.72 | 1.92E-23 | -3.87 | 6.94E-17 | 1764 |
| Cluster-40555.194649 | 83.9 | 57.73 | 2.98 | 6.6 | 85.03 | 66.38 | 8.41 | 8.11 | -3.72 | 2.40E-14 | -3.13 | 9.85E-15 | 592 |
| Cluster-40555.176582 | 3.71 | 1.95 | 0.37 | 0 | 2.27 | 2.18 | 0.13 | 0 | -3.72 | 8.87E-03 | -5.04 | 2.92E-03 | 1038 |
| Cluster-40555.188776 | 130.23 | 110.98 | 7.6 | 8.97 | 63.52 | 50.19 | 15.39 | 10.82 | -3.72 | 3.57E-38 | -2.05 | 5.06E-09 | 1038 |
| Cluster-40555.248411 | 16.97 | 15.82 | 1.14 | 1.11 | 13.21 | 15.63 | 0.97 | 1.54 | -3.71 | 6.65E-04 | -3.47 | 8.24E-04 | 476 |
| Cluster-40555.190426 | 3.17 | 6.51 | 0 | 0.66 | 13.75 | 16.04 | 0.17 | 0.22 | -3.71 | 4.39E-02 | -6.22 | 1.04E-42 | 3072 |
| Cluster-40555.172774 | 11.07 | 5.97 | 0.38 | 0.8 | 5.39 | 4.02 | 0.63 | 0.1 | -3.70 | 3.93E-04 | -3.68 | 5.60E-06 | 1264 |
| Cluster-40555.174077 | 5.69 | 8.61 | 0.64 | 0.39 | 13.33 | 18.79 | 0.42 | 0.43 | -3.70 | 1.97E-05 | -5.19 | 8.76E-11 | 1829 |
| Cluster-40555.229418 | 6.47 | 3.84 | 0.72 | 0 | 0.97 | 1.42 | 0 | 0.19 | -3.70 | 4.64E-06 | -3.54 | 2.69E-02 | 1608 |
| Cluster-40555.144139 | 14.84 | 13.29 | 1.18 | 0.8 | 4.36 | 3.87 | 0.77 | 0.7 | -3.69 | 3.72E-15 | -2.38 | 1.45E-03 | 1481 |
| Cluster-40555.305621 | 8.29 | 6.69 | 0.53 | 0.52 | 6.09 | 5.56 | 0.37 | 0.53 | -3.69 | 1.76E-05 | -3.63 | 3.10E-05 | 955 |
| Cluster-40555.197178 | 18.08 | 11.83 | 0.97 | 1.12 | 6.93 | 4.16 | 1.64 | 1.32 | -3.69 | 7.67E-10 | -1.83 | 1.08E-02 | 2630 |
| Cluster-40555.264178 | 7.52 | 12.42 | 1.25 | 0.2 | 12.21 | 7.81 | 0.53 | 1.13 | -3.68 | 1.68E-02 | -3.52 | 1.35E-02 | 458 |
| Cluster-40555.136935 | 3.05 | 2.97 | 0.11 | 0.32 | 4.23 | 4.56 | 0.15 | 0.73 | -3.68 | 2.73E-05 | -3.23 | 9.88E-08 | 2019 |
| Cluster-40555.153785 | 8.83 | 9.28 | 0.98 | 0.34 | 3.44 | 3.37 | 0.89 | 0.07 | -3.67 | 1.67E-16 | -2.78 | 1.71E-05 | 2292 |
| Cluster-40555.144224 | 1.25 | 0.74 | 0 | 0.13 | 2.46 | 1.78 | 0 | 0.3 | -3.67 | 3.98E-02 | -3.69 | 8.35E-06 | 2306 |
| Cluster-40555.184072 | 22.32 | 18.14 | 1.28 | 1.6 | 11.62 | 9.2 | 0.99 | 1.93 | -3.67 | 4.34E-06 | -2.70 | 7.59E-03 | 558 |

| | | | | | | | | | | | | | |
|---|---|---|---|---|---|---|---|---|---|---|---|---|---|
| Cluster-40555.230345 | 8.23 | 9.11 | 0 | 1.21 | 6.86 | 5.91 | 2.32 | 2.19 | -3.67 | 5.77E-13 | -1.44 | 6.16E-03 | 2165 |
| Cluster-40555.248007 | 2.62 | 2.46 | 0.22 | 0.15 | 1.76 | 1.6 | 0.05 | 0 | -3.67 | 1.72E-03 | -6.32 | 1.39E-04 | 1482 |
| Cluster-40555.107061 | 2.75 | 3 | 0 | 0.42 | 3.12 | 3.74 | 0.93 | 0.66 | -3.67 | 6.58E-03 | -2.04 | 4.11E-02 | 1244 |
| Cluster-40555.246698 | 22.01 | 25.4 | 2.02 | 1.4 | 11.63 | 13.32 | 0.39 | 0.54 | -3.66 | 2.22E-09 | -4.69 | 3.24E-08 | 658 |
| Cluster-40555.202734 | 5.89 | 8.04 | 0.27 | 0.74 | 4.03 | 3.3 | 0 | 0.6 | -3.66 | 3.86E-06 | -3.53 | 2.98E-04 | 1148 |
| Cluster-40555.159224 | 14.16 | 27.19 | 0 | 2.94 | 2.62 | 2.35 | 0 | 0 | -3.65 | 2.29E-02 | 0.00 | 8.95E-14 | 2428 |
| Cluster-40555.134250 | 6.47 | 4.79 | 0.49 | 0.29 | 19.98 | 20.97 | 0.73 | 0.59 | -3.65 | 1.88E-02 | -4.97 | 1.69E-12 | 637 |
| Cluster-40555.177698 | 1.37 | 1.34 | 0.19 | 0 | 0.5 | 0.67 | 0 | 0 | -3.65 | 4.75E-04 | 0.00 | 1.17E-03 | 2717 |
| Cluster-40555.78739 | 21.91 | 19.93 | 2.22 | 0.82 | 28.62 | 22.48 | 0.81 | 3.72 | -3.65 | 1.52E-04 | -3.42 | 1.20E-05 | 455 |
| Cluster-40555.173717 | 16.65 | 18.98 | 1.13 | 1.43 | 6.99 | 7.5 | 0.73 | 0.66 | -3.64 | 3.14E-20 | -3.33 | 2.57E-10 | 1811 |
| Cluster-40555.119919 | 35.07 | 28.52 | 3.32 | 1.31 | 111.73 | 91.96 | 5.08 | 13.5 | -3.64 | 1.90E-10 | -3.38 | 3.93E-19 | 595 |
| Cluster-40555.257614 | 0.83 | 1.13 | 0.16 | 0 | 1.81 | 1.65 | 0 | 0 | -3.64 | 1.26E-02 | 0.00 | 1.71E-09 | 2370 |
| Cluster-40555.175453 | 2.08 | 1.45 | 0.11 | 0.14 | 2.98 | 2.17 | 0.31 | 0.45 | -3.62 | 2.03E-02 | -2.63 | 6.91E-03 | 1502 |
| Cluster-40555.203329 | 20.68 | 14.37 | 1.18 | 1.4 | 6.02 | 4.84 | 1.38 | 0.35 | -3.62 | 9.90E-14 | -2.58 | 5.53E-05 | 1532 |
| Cluster-40555.198718 | 20.46 | 23.69 | 1.31 | 1.91 | 4.11 | 5.67 | 0.41 | 1.22 | -3.62 | 4.13E-10 | -2.47 | 4.08E-02 | 761 |
| Cluster-40555.190301 | 10.15 | 10.02 | 0.96 | 0.5 | 5.93 | 4.09 | 0.48 | 0.16 | -3.61 | 4.10E-07 | -4.01 | 4.94E-05 | 946 |
| Cluster-40555.299116 | 4.83 | 5.41 | 0.29 | 0.48 | 6.26 | 4.27 | 0.88 | 0.73 | -3.60 | 2.14E-02 | -2.63 | 4.37E-02 | 679 |
| Cluster-40555.206126 | 6.03 | 4.89 | 0.42 | 0.37 | 5.91 | 4.15 | 0.08 | 0.67 | -3.60 | 1.71E-03 | -3.58 | 5.17E-04 | 856 |
| Cluster-40555.205045 | 1.49 | 1.32 | 0 | 0.21 | 1.45 | 1.58 | 0 | 0.44 | -3.60 | 1.77E-02 | -2.66 | 1.51E-02 | 1999 |
| Cluster-40555.152959 | 115.44 | 96.28 | 7.59 | 8.21 | 276.27 | 249.31 | 5.43 | 13.14 | -3.60 | 1.12E-37 | -4.74 | 6.76E-50 | 1236 |
| Cluster-40555.43032 | 3.95 | 2.78 | 0.23 | 0.23 | 2.59 | 2.85 | 0.13 | 0.32 | -3.59 | 1.80E-03 | -3.58 | 1.49E-03 | 1223 |
| Cluster-40555.132461 | 1.56 | 1.08 | 0.13 | 0.07 | 1.13 | 1.43 | 0.33 | 0.1 | -3.59 | 1.60E-04 | -2.54 | 3.09E-03 | 3444 |
| Cluster-40555.100765 | 11.42 | 11.76 | 0.76 | 0.98 | 22.88 | 23.06 | 2.22 | 3.64 | -3.59 | 6.00E-04 | -2.90 | 2.67E-06 | 586 |
| Cluster-40555.40348 | 4.04 | 6.09 | 0.28 | 0.53 | 2.06 | 3.55 | 0.1 | 0.48 | -3.59 | 5.28E-04 | -3.12 | 2.47E-02 | 1022 |
| Cluster-40555.124314 | 12.11 | 10.53 | 0.87 | 0.84 | 49.6 | 25.89 | 2.93 | 1.34 | -3.58 | 2.00E-03 | -4.08 | 3.66E-04 | 544 |
| Cluster-40555.178875 | 153.7 | 175.53 | 12.92 | 12.26 | 23.27 | 22.95 | 7.71 | 10.14 | -3.57 | 1.18E-23 | -1.30 | 3.41E-02 | 670 |
| Cluster-40555.186761 | 16.38 | 15.19 | 1.24 | 1.17 | 3.38 | 4.18 | 0 | 0.49 | -3.57 | 7.91E-20 | -3.90 | 8.94E-09 | 2011 |
| Cluster-40555.205591 | 11.65 | 9.51 | 0.75 | 0.83 | 16.22 | 16.89 | 0.55 | 1.26 | -3.56 | 5.56E-08 | -4.12 | 2.20E-15 | 1062 |
| Cluster-40555.193007 | 7.49 | 8.87 | 0.81 | 0.44 | 27.59 | 24.48 | 1.73 | 0.83 | -3.56 | 3.08E-05 | -4.25 | 1.32E-17 | 866 |
| Cluster-40555.167374 | 41.35 | 37.88 | 3.52 | 2.75 | 14.33 | 22.99 | 3.2 | 4.46 | -3.55 | 2.23E-11 | -2.23 | 3.36E-02 | 584 |
| Cluster-40555.214110 | 5.24 | 3.88 | 0.47 | 0.22 | 5.14 | 6.72 | 0.5 | 0.96 | -3.54 | 2.51E-05 | -2.94 | 5.64E-06 | 1378 |
| Cluster-40555.238541 | 6.06 | 3.51 | 0.58 | 0.1 | 5.92 | 5.64 | 0.36 | 0.82 | -3.54 | 3.00E-03 | -3.28 | 4.48E-04 | 863 |

| | | | | | | | | | | | | | |
|---|---|---|---|---|---|---|---|---|---|---|---|---|---|
| Cluster-40555.154749 | 1.55 | 0.98 | 0 | 0.19 | 0.92 | 1.02 | 0.09 | 0 | -3.54 | 4.10E-02 | -4.40 | 8.71E-03 | 1921 |
| Cluster-40555.195065 | 618.52 | 478.48 | 42.93 | 41.53 | 156.32 | 160.94 | 65.54 | 41.93 | -3.54 | 1.92E-37 | -1.51 | 1.41E-04 | 457 |
| Cluster-40555.184810 | 138.93 | 117.06 | 13.15 | 6.86 | 45.81 | 31.78 | 6.34 | 11.45 | -3.54 | 3.08E-35 | -2.05 | 2.25E-05 | 890 |
| Cluster-40555.140934 | 67.98 | 70.69 | 5.72 | 5.12 | 8.34 | 7.4 | 2.46 | 2.03 | -3.54 | 8.81E-28 | -1.75 | 1.80E-02 | 983 |
| Cluster-40555.200928 | 11.57 | 6.79 | 0.62 | 0.79 | 9.27 | 8.82 | 1.32 | 0.89 | -3.54 | 1.75E-05 | -2.97 | 8.42E-09 | 1528 |
| Cluster-40555.194843 | 3.86 | 2.03 | 0 | 0.45 | 5.04 | 5.18 | 1.09 | 0.36 | -3.54 | 5.54E-03 | -2.77 | 9.01E-06 | 1694 |
| Cluster-40555.144315 | 4.94 | 3.88 | 0.31 | 0.37 | 7.13 | 6.21 | 0.69 | 0.79 | -3.54 | 3.72E-05 | -3.10 | 2.69E-07 | 1441 |
| Cluster-40555.216864 | 25.93 | 22.28 | 2.77 | 1.07 | 15.74 | 17.15 | 2.77 | 0.57 | -3.53 | 3.33E-15 | -3.28 | 1.89E-10 | 1004 |
| Cluster-40555.205906 | 12.72 | 11.31 | 1.33 | 0.56 | 2.24 | 2.01 | 0.08 | 0.09 | -3.53 | 5.53E-18 | -4.65 | 6.83E-07 | 2154 |
| Cluster-40555.167582 | 17.76 | 12.54 | 1.34 | 1.06 | 7.57 | 6.4 | 1.14 | 0.7 | -3.52 | 4.54E-15 | -2.85 | 7.60E-09 | 2068 |
| Cluster-40555.147489 | 38.48 | 24.26 | 2.81 | 2.09 | 7.28 | 5.14 | 0.36 | 0.38 | -3.52 | 2.61E-08 | -3.99 | 1.05E-02 | 546 |
| Cluster-40555.188012 | 29.94 | 14.11 | 3.16 | 0.34 | 7.32 | 6.05 | 1 | 1.33 | -3.51 | 4.71E-03 | -2.43 | 1.67E-02 | 726 |
| Cluster-40555.126199 | 2.44 | 2.57 | 0.36 | 0.06 | 3.9 | 4.49 | 0.28 | 0.14 | -3.51 | 5.88E-03 | -4.14 | 3.12E-06 | 1263 |
| Cluster-40555.224031 | 8.53 | 12.25 | 0.84 | 0.82 | 6.15 | 6.59 | 1.4 | 0.7 | -3.50 | 8.03E-06 | -2.54 | 1.11E-03 | 1010 |
| Cluster-40555.240850 | 11.3 | 10 | 0.97 | 0.74 | 3.65 | 5.18 | 0.73 | 0.3 | -3.50 | 4.02E-07 | -3.06 | 2.75E-03 | 958 |
| Cluster-40555.171496 | 6.28 | 5.49 | 0.5 | 0.45 | 1.6 | 1.72 | 0.43 | 0.19 | -3.49 | 4.03E-14 | -2.39 | 1.62E-03 | 3248 |
| Cluster-40555.141254 | 37.18 | 20.5 | 2.11 | 2.46 | 3.1 | 2.67 | 0 | 0.39 | -3.49 | 1.60E-04 | -3.95 | 1.02E-02 | 863 |
| Cluster-40555.176116 | 5.07 | 5.69 | 0.87 | 0 | 8.39 | 5.15 | 0 | 0 | -3.49 | 1.07E-04 | 0.00 | 5.30E-12 | 1019 |
| Cluster-40555.303913 | 1.41 | 0.85 | 0.19 | 0 | 0.68 | 0.31 | 0 | 0 | -3.49 | 2.77E-02 | 0.00 | 4.98E-02 | 1915 |
| Cluster-40555.191658 | 52.23 | 57.46 | 4.8 | 4.13 | 18.98 | 18.68 | 2.48 | 1.88 | -3.48 | 1.86E-38 | -3.05 | 2.13E-17 | 2461 |
| Cluster-40555.184776 | 9.21 | 7.62 | 0.69 | 0.66 | 4.91 | 3.63 | 0.62 | 1.05 | -3.48 | 4.36E-14 | -2.27 | 4.04E-05 | 2369 |
| Cluster-40555.170820 | 80.16 | 59.63 | 4.41 | 6.77 | 67.79 | 47.11 | 10.53 | 5.43 | -3.48 | 2.54E-12 | -2.79 | 2.15E-08 | 484 |
| Cluster-40555.304229 | 6.57 | 6.51 | 0.39 | 0.64 | 3.09 | 3.1 | 0 | 0.86 | -3.48 | 1.06E-04 | -2.77 | 2.31E-02 | 1000 |
| Cluster-40555.265675 | 8.43 | 10.04 | 1.18 | 0.35 | 8.64 | 13.56 | 0.7 | 0 | -3.47 | 1.45E-11 | -4.95 | 3.11E-06 | 1617 |
| Cluster-40555.68824 | 3.76 | 3.78 | 0.23 | 0.39 | 4.61 | 4.67 | 1.23 | 0.71 | -3.47 | 3.92E-03 | -2.20 | 1.60E-02 | 1076 |
| Cluster-40555.170414 | 28.98 | 26.59 | 2 | 2.58 | 8.66 | 5.36 | 0.83 | 1.78 | -3.47 | 1.78E-14 | -2.36 | 1.92E-03 | 945 |
| Cluster-40555.131696 | 4.63 | 4.69 | 0.33 | 0.43 | 10.16 | 5.03 | 0.83 | 0.29 | -3.46 | 1.04E-02 | -3.70 | 5.54E-03 | 815 |
| Cluster-40555.140801 | 56.1 | 41.42 | 2.23 | 5.65 | 169.85 | 110.86 | 5.57 | 6.2 | -3.46 | 9.23E-14 | -4.50 | 1.20E-12 | 648 |
| Cluster-40555.145850 | 39.11 | 40.98 | 1 | 5.47 | 15.13 | 12.03 | 2.4 | 2.76 | -3.46 | 4.81E-16 | -2.32 | 6.68E-05 | 864 |
| Cluster-40555.83855 | 5.22 | 7.03 | 0.57 | 0.45 | 2.21 | 2.3 | 0.28 | 0.32 | -3.46 | 1.88E-07 | -2.84 | 6.92E-03 | 1537 |
| Cluster-40555.131664 | 8.96 | 5.01 | 0.94 | 0.23 | 4.54 | 4.1 | 0.46 | 0.38 | -3.46 | 1.34E-04 | -3.26 | 7.19E-04 | 1038 |
| Cluster-40555.154102 | 3.47 | 2.97 | 0.22 | 0.34 | 4.84 | 4.87 | 0.07 | 0.17 | -3.46 | 6.93E-03 | -5.28 | 3.96E-08 | 1138 |

| | | | | | | | | | | | | | |
|---|---|---|---|---|---|---|---|---|---|---|---|---|---|
| Cluster-40555.193736 | 13.77 | 15.15 | 0 | 2.33 | 7.53 | 7.53 | 2.33 | 0.89 | -3.46 | 9.92E-11 | -2.18 | 4.05E-04 | 1262 |
| Cluster-40555.187041 | 21.28 | 15.03 | 1.05 | 1.92 | 21.66 | 18.73 | 1.56 | 1.42 | -3.46 | 1.53E-12 | -3.68 | 5.48E-16 | 1163 |
| Cluster-40555.250188 | 30.77 | 28.06 | 3.28 | 1.62 | 14.75 | 8.36 | 1.62 | 1.52 | -3.45 | 9.32E-09 | -2.81 | 1.93E-03 | 580 |
| Cluster-40555.151244 | 75.29 | 55.36 | 5.02 | 5.75 | 57.94 | 41.33 | 6.03 | 8.01 | -3.45 | 2.70E-21 | -2.75 | 1.94E-11 | 986 |
| Cluster-40555.46039 | 15.5 | 11.84 | 1.09 | 1.17 | 12.44 | 8.68 | 0.9 | 1.76 | -3.45 | 1.85E-06 | -2.91 | 5.42E-05 | 771 |
| Cluster-40555.214120 | 371.77 | 510.43 | 35.2 | 38.85 | 77.76 | 82.79 | 34.7 | 31.77 | -3.44 | 2.97E-07 | -1.21 | 2.02E-03 | 760 |
| Cluster-40555.157516 | 11.49 | 11.76 | 2.06 | 0 | 13.91 | 15.58 | 1.54 | 0 | -3.44 | 6.50E-06 | -4.23 | 1.88E-09 | 725 |
| Cluster-40555.157950 | 13.03 | 6.24 | 0.47 | 1.07 | 8.78 | 8.92 | 0.44 | 0.5 | -3.43 | 1.08E-02 | -4.22 | 1.87E-10 | 1111 |
| Cluster-40555.151120 | 2.59 | 5.2 | 0.19 | 0.49 | 1.88 | 2.99 | 0.27 | 0.15 | -3.43 | 4.34E-02 | -3.50 | 1.30E-03 | 1603 |
| Cluster-40555.237545 | 15.06 | 12 | 1.17 | 1.15 | 6.85 | 7.3 | 1.47 | 2.02 | -3.43 | 6.25E-08 | -1.97 | 1.44E-02 | 919 |
| Cluster-40555.185566 | 19.28 | 14.5 | 1.28 | 1.58 | 5.39 | 4.92 | 1.29 | 0.88 | -3.42 | 4.44E-22 | -2.20 | 1.42E-05 | 2500 |
| Cluster-40555.35887 | 2.18 | 3.69 | 0.17 | 0.33 | 70.45 | 38.44 | 0.23 | 0.19 | -3.42 | 5.14E-03 | -7.93 | 4.50E-11 | 1346 |
| Cluster-40555.247261 | 11.14 | 9.84 | 0.71 | 1.05 | 18.44 | 14.12 | 2.12 | 2.33 | -3.42 | 8.50E-06 | -2.79 | 2.87E-07 | 863 |
| Cluster-40555.162630 | 21.96 | 19.58 | 1.32 | 2.17 | 11.67 | 8.6 | 2.6 | 1.53 | -3.41 | 2.36E-17 | -2.23 | 4.67E-06 | 1517 |
| Cluster-40555.206883 | 12.96 | 10.67 | 0.83 | 1.14 | 12.51 | 13.38 | 3.18 | 2.41 | -3.41 | 9.52E-09 | -2.15 | 2.78E-05 | 1130 |
| Cluster-40555.33939 | 7.9 | 7.23 | 0.62 | 0.67 | 22.04 | 22.08 | 2.96 | 2.55 | -3.40 | 2.32E-02 | -2.95 | 9.37E-06 | 558 |
| Cluster-40555.163378 | 10.83 | 10.69 | 1.46 | 0.41 | 1.96 | 2.44 | 0.96 | 0.16 | -3.40 | 1.51E-19 | -1.92 | 2.20E-02 | 2752 |
| Cluster-40555.184043 | 19.68 | 22.26 | 1.53 | 2.08 | 6.52 | 8.16 | 0.65 | 1 | -3.40 | 3.49E-16 | -3.10 | 1.89E-07 | 1363 |
| Cluster-40555.132589 | 65.29 | 60.04 | 6.58 | 4.18 | 22.49 | 19.5 | 4.94 | 3.4 | -3.40 | 7.72E-19 | -2.27 | 5.21E-05 | 697 |
| Cluster-40555.110123 | 5.89 | 3.91 | 0.4 | 0.46 | 3.05 | 3.41 | 0.81 | 0.74 | -3.39 | 1.24E-05 | -2.00 | 2.31E-02 | 1510 |
| Cluster-40555.39581 | 1.03 | 0.74 | 0.14 | 0.01 | 2.16 | 2.54 | 0.37 | 0.25 | -3.39 | 2.21E-03 | -2.86 | 1.39E-06 | 3694 |
| Cluster-40555.235149 | 4.75 | 5.18 | 0.32 | 0.52 | 4.15 | 3.19 | 1.04 | 1.2 | -3.39 | 4.86E-08 | -1.63 | 1.31E-02 | 2147 |
| Cluster-40555.194456 | 144.12 | 125.8 | 11.48 | 11.79 | 176.45 | 132.72 | 11.95 | 23.9 | -3.39 | 7.09E-37 | -3.03 | 4.08E-18 | 1211 |
| Cluster-40555.209774 | 16.11 | 15.78 | 1.35 | 1.43 | 4.99 | 3.07 | 0.2 | 0.67 | -3.39 | 8.13E-16 | -3.08 | 4.31E-05 | 1649 |
| Cluster-40555.247610 | 15.03 | 12.25 | 0 | 2.3 | 5.82 | 5.69 | 1.66 | 0 | -3.39 | 7.53E-11 | -2.78 | 3.03E-05 | 1398 |
| Cluster-40555.226400 | 1.49 | 1.95 | 0.05 | 0.26 | 1.5 | 1.02 | 0.09 | 0.08 | -3.39 | 3.34E-02 | -3.91 | 1.64E-02 | 1542 |
| Cluster-40555.183080 | 12.29 | 9.01 | 1.26 | 0.6 | 5.86 | 5.24 | 0.91 | 0.45 | -3.38 | 1.51E-04 | -2.88 | 1.87E-02 | 688 |
| Cluster-40555.214488 | 3.28 | 2.8 | 0.26 | 0.26 | 2.32 | 2.08 | 0.61 | 0.67 | -3.37 | 8.52E-07 | -1.71 | 2.31E-02 | 2732 |
| Cluster-40555.208526 | 5.56 | 10.28 | 0.4 | 1.03 | 9.83 | 13.54 | 0.5 | 1.07 | -3.37 | 3.20E-02 | -3.89 | 3.58E-05 | 577 |
| Cluster-40555.208330 | 3.5 | 1.99 | 0.19 | 0.3 | 2.86 | 2.35 | 0.07 | 0.33 | -3.37 | 2.50E-04 | -3.65 | 6.89E-06 | 1990 |
| Cluster-40555.240068 | 6.14 | 4.94 | 0.47 | 0.5 | 42.61 | 26.28 | 1.81 | 1.61 | -3.37 | 2.82E-02 | -4.26 | 2.64E-08 | 657 |
| Cluster-40555.263763 | 2.64 | 2 | 0.26 | 0.16 | 0.9 | 1.45 | 0 | 0 | -3.36 | 1.85E-03 | 0.00 | 1.64E-04 | 1745 |

| | | | | | | | | | | | | | |
|---|---|---|---|---|---|---|---|---|---|---|---|---|---|
| Cluster-40555.209660 | 0.9 | 0.64 | 0.08 | 0.07 | 0.51 | 0.95 | 0.06 | 0 | -3.36 | 2.98E-02 | -4.75 | 4.79E-03 | 3076 |
| Cluster-40555.129827 | 6.02 | 3.96 | 0.41 | 0.46 | 1.58 | 1.87 | 0.28 | 0.23 | -3.35 | 4.45E-07 | -2.72 | 1.14E-02 | 1890 |
| Cluster-40555.157971 | 4.31 | 5.17 | 0.17 | 0.65 | 4.35 | 3.3 | 0.67 | 0.93 | -3.35 | 1.65E-05 | -2.17 | 3.20E-03 | 1607 |
| Cluster-40555.108213 | 9.48 | 16.59 | 0.89 | 1.44 | 8.73 | 11.07 | 1.94 | 1.15 | -3.35 | 7.85E-03 | -2.63 | 2.72E-04 | 813 |
| Cluster-40555.119917 | 4.73 | 3.47 | 0.71 | 0.12 | 18.69 | 15.2 | 0.95 | 3 | -3.35 | 3.28E-02 | -3.00 | 4.67E-07 | 733 |
| Cluster-40555.200699 | 29.17 | 30.08 | 3.41 | 1.91 | 4.05 | 3.74 | 1.26 | 0.84 | -3.35 | 9.31E-25 | -1.84 | 1.09E-02 | 1751 |
| Cluster-40555.121864 | 9.79 | 7.85 | 0.89 | 0.64 | 10.95 | 7.34 | 1.42 | 1.03 | -3.35 | 1.91E-06 | -2.84 | 4.83E-06 | 1060 |
| Cluster-40555.183915 | 243.4 | 295.17 | 20.54 | 27.66 | 757.76 | 653.5 | 35.57 | 20.5 | -3.34 | 9.76E-14 | -4.59 | 2.92E-48 | 756 |
| Cluster-40555.222454 | 4.78 | 3.51 | 0.26 | 0.47 | 5.06 | 3.36 | 1.52 | 0.67 | -3.34 | 8.32E-06 | -1.89 | 3.94E-03 | 1870 |
| Cluster-40555.179168 | 89.98 | 75.69 | 6.44 | 8.41 | 29.12 | 22.91 | 3.25 | 2.36 | -3.33 | 1.01E-31 | -3.15 | 6.91E-16 | 1363 |
| Cluster-40555.129359 | 1.57 | 1.26 | 0.25 | 0 | 0.86 | 1.05 | 0.13 | 0 | -3.33 | 1.10E-02 | -3.95 | 1.93E-02 | 1914 |
| Cluster-40555.183262 | 18.51 | 14.79 | 1.14 | 1.85 | 4.54 | 4.5 | 0.99 | 1.26 | -3.32 | 3.39E-16 | -1.94 | 2.54E-03 | 1766 |
| Cluster-40555.83599 | 9.16 | 7.81 | 0.63 | 0.87 | 2.79 | 2.58 | 0.67 | 0.11 | -3.32 | 7.10E-07 | -2.68 | 2.36E-02 | 1206 |
| Cluster-40555.157597 | 13.9 | 11.99 | 1.52 | 0.86 | 13.74 | 10.55 | 3.05 | 1.74 | -3.31 | 2.21E-15 | -2.28 | 5.87E-08 | 1888 |
| Cluster-40555.167722 | 343.19 | 368.09 | 39.57 | 25.94 | 309.53 | 299.31 | 45.07 | 69.68 | -3.31 | 1.09E-43 | -2.33 | 3.19E-15 | 1109 |
| Cluster-40555.190792 | 76.1 | 47.93 | 6.04 | 5.22 | 14.96 | 12.68 | 3.64 | 2.07 | -3.31 | 1.56E-07 | -2.22 | 4.94E-06 | 1201 |
| Cluster-40555.178870 | 8.72 | 8.67 | 1.15 | 0.47 | 7.68 | 6.41 | 0.94 | 1.45 | -3.31 | 3.08E-06 | -2.48 | 5.49E-04 | 1017 |
| Cluster-40555.156218 | 8.61 | 5.97 | 0.88 | 0.46 | 3.9 | 2.19 | 0.34 | 0.84 | -3.31 | 6.59E-07 | -2.27 | 2.07E-02 | 1308 |
| Cluster-40555.180401 | 1.52 | 1.28 | 0 | 0.25 | 3.24 | 3.16 | 0.32 | 1.14 | -3.30 | 2.13E-03 | -2.05 | 1.80E-04 | 3113 |
| Cluster-40555.146389 | 16.54 | 14.25 | 1.6 | 1.25 | 5.16 | 4.78 | 0.5 | 0.93 | -3.30 | 9.57E-10 | -2.68 | 1.99E-03 | 1032 |
| Cluster-40555.207494 | 2.25 | 2.05 | 0.12 | 0.27 | 11.12 | 11.97 | 0.29 | 0.82 | -3.30 | 5.44E-03 | -4.29 | 4.61E-19 | 1738 |
| Cluster-40555.171696 | 35.57 | 45.09 | 4.28 | 3.22 | 49.98 | 47.22 | 2.28 | 2.03 | -3.30 | 8.08E-11 | -4.43 | 1.02E-28 | 1011 |
| Cluster-40555.158928 | 1.79 | 2.19 | 0 | 0.37 | 2.77 | 4.57 | 0.51 | 0.96 | -3.30 | 3.20E-02 | -2.28 | 4.08E-02 | 1466 |
| Cluster-40555.152467 | 13.86 | 15.87 | 0.56 | 2.16 | 10.28 | 12.61 | 2.33 | 1.95 | -3.29 | 2.77E-04 | -2.37 | 9.12E-03 | 592 |
| Cluster-40555.159662 | 9.01 | 4.23 | 0.78 | 0.41 | 2.76 | 2.42 | 0.52 | 0.46 | -3.29 | 1.19E-02 | -2.37 | 3.92E-02 | 1276 |
| Cluster-40555.208893 | 7.37 | 11.38 | 1.17 | 0.64 | 6.19 | 7.02 | 0 | 2.11 | -3.28 | 4.14E-04 | -2.57 | 4.91E-03 | 789 |
| Cluster-40555.71394 | 2.35 | 2.47 | 0.22 | 0.24 | 3.53 | 2.98 | 0.14 | 0.76 | -3.28 | 2.76E-02 | -2.71 | 6.38E-03 | 1203 |
| Cluster-40555.91497 | 3.32 | 3.95 | 0.34 | 0.37 | 1.29 | 1.13 | 0.06 | 0 | -3.27 | 2.01E-04 | -4.96 | 4.92E-03 | 1585 |
| Cluster-40555.168852 | 6.13 | 6.16 | 0.64 | 0.53 | 2.6 | 3.65 | 0.28 | 0.32 | -3.26 | 1.71E-03 | -3.30 | 2.27E-02 | 855 |
| Cluster-40555.237747 | 1.51 | 1.73 | 0.18 | 0.1 | 2.35 | 1.33 | 0.11 | 0.14 | -3.26 | 4.53E-02 | -4.01 | 2.11E-03 | 1449 |
| Cluster-40555.45663 | 2.35 | 2.48 | 0.24 | 0.22 | 0.8 | 0.73 | 0 | 0 | -3.26 | 5.23E-03 | 0.00 | 2.00E-02 | 1537 |
| Cluster-40555.143406 | 4.75 | 3.13 | 0.58 | 0.17 | 1.61 | 1.71 | 0 | 0 | -3.25 | 1.80E-05 | 0.00 | 2.84E-06 | 1717 |

| | | | | | | | | | | | | | |
|---|---|---|---|---|---|---|---|---|---|---|---|---|---|
| Cluster-40555.223896 | 0.95 | 0.64 | 0.1 | 0.05 | 1.3 | 1.18 | 0.07 | 0.43 | -3.25 | 8.40E-03 | -2.22 | 5.63E-03 | 3753 |
| Cluster-40555.195975 | 3.77 | 3.22 | 0.5 | 0.16 | 12.32 | 8.87 | 0.7 | 0 | -3.24 | 2.73E-05 | -4.90 | 3.95E-21 | 1863 |
| Cluster-40555.295439 | 4.03 | 4.56 | 0.53 | 0.31 | 8.68 | 8.57 | 0.98 | 1.55 | -3.24 | 3.66E-05 | -2.69 | 1.51E-07 | 1553 |
| Cluster-40555.44081 | 4.06 | 2.86 | 0.34 | 0.33 | 3.97 | 2.97 | 0.32 | 0.14 | -3.24 | 1.56E-03 | -3.84 | 2.90E-05 | 1360 |
| Cluster-40555.195943 | 12.34 | 23.73 | 1.12 | 2.35 | 9.88 | 7.73 | 1.74 | 2.27 | -3.24 | 2.96E-02 | -2.05 | 4.17E-05 | 1574 |
| Cluster-40555.156126 | 3.66 | 3.08 | 0.27 | 0.39 | 9.14 | 9.55 | 0.15 | 0.12 | -3.24 | 4.26E-07 | -6.03 | 1.95E-33 | 2855 |
| Cluster-40555.247556 | 1.99 | 1.76 | 0.37 | 0 | 1.54 | 2.52 | 0 | 0.59 | -3.23 | 2.04E-05 | -2.67 | 3.99E-02 | 3089 |
| Cluster-40555.206210 | 32.75 | 35.11 | 5.19 | 1.47 | 44.25 | 35.5 | 3.27 | 8.72 | -3.23 | 2.19E-05 | -2.67 | 4.80E-05 | 427 |
| Cluster-40555.166875 | 53.1 | 42.68 | 5.37 | 3.9 | 14.96 | 13.22 | 2.66 | 3.35 | -3.22 | 9.17E-13 | -2.16 | 3.74E-03 | 628 |
| Cluster-40555.195611 | 1.86 | 2.59 | 0.16 | 0.27 | 2.02 | 2.96 | 0 | 0.13 | -3.22 | 5.42E-06 | -5.25 | 1.57E-08 | 4476 |
| Cluster-40555.234710 | 5.49 | 5.28 | 0 | 1.04 | 7.84 | 7.91 | 1.56 | 2.27 | -3.22 | 2.05E-03 | -1.97 | 3.86E-03 | 1039 |
| Cluster-40555.184301 | 147 | 183.29 | 22.74 | 9.93 | 149.44 | 172.13 | 11.1 | 10 | -3.22 | 6.67E-11 | -3.87 | 1.27E-28 | 681 |
| Cluster-40555.181508 | 131.82 | 179.42 | 13.47 | 17.16 | 44.26 | 43.51 | 14.7 | 14.11 | -3.21 | 3.73E-07 | -1.54 | 2.31E-05 | 1220 |
| Cluster-40555.163668 | 5.55 | 4.85 | 0.48 | 0.55 | 5.34 | 5.29 | 1.32 | 0.85 | -3.21 | 1.01E-04 | -2.23 | 1.95E-03 | 1294 |
| Cluster-40555.176538 | 10.09 | 19.3 | 1.64 | 1.26 | 9.03 | 11.77 | 0.12 | 0.36 | -3.21 | 2.77E-02 | -5.50 | 1.40E-09 | 739 |
| Cluster-40555.193381 | 7.91 | 9.86 | 0.9 | 0.87 | 9.16 | 7.56 | 0.77 | 0.59 | -3.21 | 1.05E-10 | -3.56 | 1.43E-12 | 1840 |
| Cluster-40555.190658 | 58.87 | 63.83 | 5.81 | 6.26 | 66.77 | 64.3 | 10.95 | 8.93 | -3.21 | 6.07E-35 | -2.66 | 3.70E-18 | 2634 |
| Cluster-40555.225055 | 347.95 | 378.23 | 36.2 | 35.66 | 73.31 | 70.32 | 21.18 | 19.54 | -3.20 | 1.42E-35 | -1.76 | 2.17E-06 | 749 |
| Cluster-40555.206541 | 2.48 | 1.71 | 0.22 | 0.18 | 1.63 | 1.58 | 0.26 | 0 | -3.20 | 4.34E-04 | -3.62 | 1.79E-04 | 2413 |
| Cluster-40555.188570 | 39.84 | 26.95 | 3.79 | 2.79 | 8.96 | 6.77 | 2.56 | 2.38 | -3.19 | 1.22E-10 | -1.59 | 1.53E-02 | 1170 |
| Cluster-40555.189626 | 73.29 | 73.59 | 4.73 | 9.57 | 59.09 | 44.77 | 14.71 | 8.03 | -3.19 | 4.42E-06 | -2.14 | 5.46E-03 | 363 |
| Cluster-40555.212433 | 1.06 | 1.08 | 0.21 | 0 | 7.02 | 7.99 | 0.01 | 0.39 | -3.19 | 6.11E-05 | -5.14 | 4.58E-34 | 4778 |
| Cluster-40555.176186 | 20.37 | 14.93 | 2.02 | 1.48 | 3.56 | 3.21 | 0.94 | 0.7 | -3.18 | 6.96E-13 | -2.00 | 3.98E-02 | 1298 |
| Cluster-40555.206007 | 14.8 | 14.88 | 2.2 | 0.78 | 13.64 | 14.99 | 2.36 | 2.64 | -3.18 | 1.26E-14 | -2.44 | 6.94E-09 | 1624 |
| Cluster-40555.151794 | 6.79 | 3.53 | 0.69 | 0.36 | 7.88 | 8.53 | 1.77 | 1.09 | -3.18 | 2.49E-02 | -2.48 | 6.17E-03 | 716 |
| Cluster-40555.132438 | 3.64 | 3.27 | 0.71 | 0 | 1.81 | 2.02 | 0 | 0 | -3.18 | 3.19E-03 | 0.00 | 2.75E-04 | 1133 |
| Cluster-40555.131031 | 8.63 | 10.46 | 1.45 | 0.5 | 24.34 | 17.14 | 1.67 | 3.61 | -3.18 | 1.19E-05 | -2.89 | 1.99E-09 | 900 |
| Cluster-40555.95067 | 10.23 | 7.47 | 0.48 | 1.22 | 58.02 | 37.91 | 1.52 | 1.67 | -3.18 | 3.03E-07 | -4.83 | 1.41E-13 | 1350 |
| Cluster-40555.169032 | 3.12 | 5.3 | 0.52 | 0.31 | 17.82 | 17.59 | 0.28 | 0.59 | -3.18 | 5.22E-03 | -5.27 | 1.00E-23 | 1211 |
| Cluster-40555.167247 | 67.32 | 74.26 | 6.45 | 7.77 | 34.07 | 30.2 | 8.42 | 11.17 | -3.18 | 9.29E-25 | -1.64 | 3.34E-05 | 1074 |
| Cluster-40555.321527 | 19.53 | 12.88 | 1.75 | 1.49 | 7.65 | 8.62 | 2.05 | 2.19 | -3.17 | 2.39E-07 | -1.88 | 2.31E-02 | 823 |
| Cluster-40555.191438 | 2723.68 | 2905.25 | 261.23 | 309.98 | 113.05 | 92.71 | 46 | 44.32 | -3.16 | 9.56E-44 | -1.12 | 2.25E-03 | 924 |

| | | | | | | | | | | | | | |
|---|---|---|---|---|---|---|---|---|---|---|---|---|---|
| Cluster-40555.156518 | 2.13 | 3.57 | 0.23 | 0.33 | 1.5 | 1.9 | 0.06 | 0.13 | -3.16 | 4.20E-03 | -4.01 | 2.33E-03 | 1536 |
| Cluster-40555.184579 | 48.11 | 63.17 | 5.87 | 5.58 | 42.3 | 44.7 | 5.96 | 4.24 | -3.15 | 6.46E-09 | -3.04 | 5.22E-21 | 2333 |
| Cluster-40555.176408 | 42.25 | 50.29 | 4.26 | 5.22 | 14.7 | 14.42 | 3.28 | 2.86 | -3.15 | 1.26E-15 | -2.19 | 2.73E-06 | 1249 |
| Cluster-40555.153020 | 90.5 | 74.07 | 8.01 | 8.82 | 142.32 | 123.08 | 21.07 | 14.07 | -3.15 | 1.65E-15 | -2.86 | 1.81E-15 | 572 |
| Cluster-40555.62724 | 4.95 | 4.14 | 0.69 | 0.27 | 5 | 3.44 | 0.87 | 0.71 | -3.14 | 1.69E-03 | -2.34 | 1.47E-02 | 1065 |
| Cluster-40555.190446 | 9.25 | 6.95 | 0.86 | 0.85 | 12.07 | 12.27 | 0.26 | 0.45 | -3.14 | 2.58E-06 | -5.05 | 8.46E-18 | 1222 |
| Cluster-40555.41538 | 2.09 | 1.56 | 0.24 | 0.14 | 2.57 | 2.81 | 0 | 0.11 | -3.14 | 3.37E-03 | -5.52 | 5.80E-10 | 2144 |
| Cluster-40555.241639 | 7.07 | 4.27 | 0 | 1.15 | 3.04 | 3.56 | 0.74 | 0 | -3.13 | 5.79E-03 | -3.09 | 1.83E-02 | 925 |
| Cluster-40555.194475 | 3.73 | 3.97 | 0.48 | 0.33 | 2.81 | 3.26 | 0.37 | 0.1 | -3.12 | 2.87E-07 | -3.61 | 4.76E-08 | 2567 |
| Cluster-40555.172382 | 11.19 | 9.85 | 1.39 | 0.81 | 4.41 | 4.49 | 0.34 | 0.87 | -3.12 | 7.25E-17 | -2.81 | 2.31E-08 | 2867 |
| Cluster-40555.278242 | 3.06 | 3.39 | 0.38 | 0.31 | 12.09 | 18.01 | 1.12 | 1.71 | -3.12 | 4.57E-05 | -3.36 | 5.21E-05 | 2081 |
| Cluster-40555.110309 | 8.96 | 8.1 | 1.09 | 0.7 | 3.57 | 2.65 | 0.69 | 0.94 | -3.11 | 3.71E-12 | -1.85 | 5.98E-03 | 2286 |
| Cluster-40555.117975 | 20.42 | 28.98 | 1.71 | 3.48 | 70 | 64.13 | 10.63 | 18.99 | -3.11 | 4.10E-05 | -2.10 | 1.07E-09 | 945 |
| Cluster-40555.193420 | 50.22 | 56.67 | 5.47 | 5.85 | 19.29 | 14.24 | 6.64 | 8.71 | -3.10 | 1.99E-24 | -1.05 | 3.37E-02 | 1369 |
| Cluster-40555.213022 | 11.33 | 13.52 | 1.65 | 0.99 | 4.24 | 3.74 | 0.76 | 1.49 | -3.10 | 2.24E-12 | -1.74 | 1.67E-02 | 1667 |
| Cluster-40555.248674 | 3.54 | 4.69 | 0.51 | 0.37 | 6.06 | 5.72 | 0.26 | 0.16 | -3.10 | 1.27E-03 | -4.74 | 2.06E-09 | 1227 |
| Cluster-40555.195268 | 163.93 | 101.93 | 14.62 | 13.31 | 43.72 | 38.25 | 12.34 | 7.61 | -3.09 | 4.53E-07 | -1.97 | 2.49E-05 | 623 |
| Cluster-40555.176553 | 303 | 275.19 | 32.02 | 29.34 | 95.55 | 78.42 | 32.16 | 30.58 | -3.09 | 2.75E-33 | -1.40 | 1.31E-04 | 770 |
| Cluster-40555.200291 | 95.4 | 75.15 | 8.49 | 9.57 | 172.16 | 120.13 | 12.58 | 8.85 | -3.09 | 4.03E-25 | -3.70 | 9.15E-15 | 1038 |
| Cluster-40555.216095 | 15.32 | 16.51 | 1.81 | 1.6 | 3.67 | 4.5 | 1.16 | 1.18 | -3.09 | 7.71E-12 | -1.76 | 4.24E-02 | 1342 |
| Cluster-40555.189335 | 3.7 | 2.75 | 0.44 | 0.25 | 2.98 | 2.59 | 0.08 | 0 | -3.09 | 2.33E-06 | -6.01 | 2.94E-13 | 2650 |
| Cluster-40555.240852 | 7.71 | 10.61 | 1.5 | 0.48 | 3.14 | 4.27 | 0.25 | 0.39 | -3.09 | 1.33E-03 | -3.47 | 4.55E-02 | 671 |
| Cluster-40555.164614 | 99.1 | 113.86 | 10.7 | 12.14 | 5.77 | 4.49 | 1.69 | 1.32 | -3.08 | 6.98E-21 | -1.72 | 3.37E-02 | 1222 |
| Cluster-40555.240701 | 18.88 | 16.04 | 1.83 | 1.9 | 25.06 | 15.98 | 2.85 | 3.63 | -3.08 | 4.45E-06 | -2.60 | 1.85E-05 | 700 |
| Cluster-40555.203395 | 101.33 | 107.72 | 13.25 | 9.27 | 35.2 | 27.22 | 8.95 | 8.65 | -3.08 | 1.75E-19 | -1.75 | 9.38E-04 | 611 |
| Cluster-40555.208100 | 78.42 | 49.61 | 5.79 | 7.68 | 86.47 | 76.1 | 24.41 | 16.84 | -3.08 | 4.94E-08 | -1.92 | 3.73E-05 | 452 |
| Cluster-40555.154115 | 33.27 | 37.68 | 2.3 | 5.29 | 66.01 | 56.71 | 7.3 | 5.46 | -3.07 | 4.84E-10 | -3.20 | 1.38E-15 | 689 |
| Cluster-40555.214855 | 635.2 | 844.26 | 80.62 | 80.07 | 203 | 196.94 | 81.32 | 75.72 | -3.07 | 1.25E-07 | -1.29 | 1.19E-04 | 887 |
| Cluster-40555.243885 | 7.65 | 8.35 | 0.8 | 0.95 | 8.25 | 8.42 | 1.99 | 1.4 | -3.07 | 5.70E-08 | -2.24 | 1.25E-05 | 1603 |
| Cluster-40555.197274 | 36.7 | 29.22 | 4.9 | 2.29 | 31.36 | 27.63 | 2.56 | 5.57 | -3.06 | 1.17E-25 | -2.77 | 1.74E-16 | 2128 |
| Cluster-40555.199353 | 76.32 | 41.34 | 5.37 | 6.95 | 85.85 | 78.42 | 12.32 | 9.77 | -3.06 | 1.52E-04 | -2.85 | 7.43E-07 | 374 |
| Cluster-40555.186962 | 25.39 | 30.98 | 6.43 | 1.23 | 41.91 | 39.03 | 2.64 | 7.21 | -3.06 | 3.77E-18 | -3.63 | 6.10E-30 | 3220 |

| | | | | | | | | | | | | | |
|---|---|---|---|---|---|---|---|---|---|---|---|---|---|
| Cluster-40555.188892 | 969.15 | 921.51 | 97.17 | 102.43 | 119.76 | 73.81 | 33.18 | 30.86 | -3.06 | 4.25E-24 | -1.57 | 4.04E-02 | 321 |
| Cluster-40555.193569 | 15.69 | 9.92 | 0.87 | 1.85 | 7.91 | 6.66 | 2.26 | 1.99 | -3.06 | 5.38E-06 | -1.73 | 3.96E-02 | 900 |
| Cluster-40555.109664 | 8.74 | 6.37 | 1.19 | 0.4 | 14.85 | 11.36 | 0.57 | 1.4 | -3.06 | 3.25E-03 | -3.67 | 2.46E-07 | 719 |
| Cluster-40555.181812 | 39.22 | 33.04 | 3.71 | 4.17 | 19.22 | 15.45 | 6.16 | 4.75 | -3.05 | 4.36E-23 | -1.60 | 5.14E-05 | 1832 |
| Cluster-40555.192854 | 6.75 | 4.85 | 0.6 | 0.66 | 5.77 | 4.47 | 0.66 | 0.89 | -3.05 | 5.59E-08 | -2.63 | 1.07E-06 | 2154 |
| Cluster-40555.194991 | 121.76 | 141.23 | 14.35 | 14.53 | 596.12 | 421.14 | 27.96 | 62.73 | -3.05 | 9.50E-19 | -3.40 | 8.12E-14 | 1014 |
| Cluster-40555.132064 | 15.22 | 12.9 | 1.73 | 1.34 | 30.7 | 40.43 | 1.07 | 2.65 | -3.05 | 1.22E-07 | -4.18 | 1.24E-11 | 972 |
| Cluster-40555.133747 | 25.33 | 34.61 | 2.34 | 4.17 | 19.67 | 18.85 | 1.56 | 2.84 | -3.05 | 9.68E-03 | -3.09 | 3.35E-02 | 364 |
| Cluster-40555.225976 | 12.86 | 15.15 | 1.76 | 1.32 | 7.13 | 6.6 | 0.9 | 0 | -3.05 | 1.38E-07 | -3.94 | 1.74E-06 | 956 |
| Cluster-40555.193849 | 51.99 | 38.99 | 5.57 | 4.41 | 46.06 | 37.96 | 10.22 | 12.03 | -3.05 | 2.23E-21 | -1.84 | 1.00E-07 | 1407 |
| Cluster-40555.114832 | 1.88 | 2.17 | 0.13 | 0.28 | 1.9 | 1.96 | 0.41 | 0.18 | -3.05 | 3.20E-02 | -2.69 | 2.89E-02 | 1484 |
| Cluster-40555.302810 | 1.19 | 0.96 | 0.09 | 0.14 | 2.08 | 1.05 | 0.11 | 0.08 | -3.05 | 4.70E-03 | -4.03 | 1.47E-03 | 3603 |
| Cluster-40555.196652 | 82.82 | 71.1 | 9.89 | 7.04 | 34.75 | 31.18 | 2.79 | 5.45 | -3.04 | 9.01E-15 | -2.93 | 3.95E-08 | 577 |
| Cluster-40555.218848 | 10.17 | 12.28 | 1.11 | 1.39 | 21.23 | 19.61 | 0.35 | 0.45 | -3.03 | 4.42E-05 | -5.56 | 3.00E-19 | 829 |
| Cluster-40555.206892 | 24.65 | 33.81 | 3.51 | 3.04 | 7.19 | 4.27 | 1.32 | 1.89 | -3.03 | 1.37E-06 | -1.75 | 2.44E-02 | 1334 |
| Cluster-40555.190624 | 15.43 | 13.8 | 1.48 | 1.78 | 7.22 | 8.06 | 1.33 | 2.33 | -3.01 | 1.42E-12 | -2.00 | 1.15E-04 | 1667 |
| Cluster-40555.197295 | 14.9 | 15.99 | 1.42 | 2.05 | 9.36 | 7.91 | 1.51 | 2.35 | -3.00 | 6.91E-12 | -2.07 | 5.63E-05 | 1513 |
| Cluster-40555.190220 | 197.85 | 174.14 | 26.88 | 15.47 | 223.56 | 217.17 | 19.63 | 46.03 | -3.00 | 2.65E-38 | -2.66 | 5.24E-20 | 2178 |
| Cluster-40555.184816 | 239.82 | 188.43 | 29.83 | 18.98 | 301.34 | 335.3 | 49.3 | 29.42 | -2.99 | 7.70E-34 | -2.96 | 5.10E-23 | 1167 |
| Cluster-40555.164871 | 17.23 | 21.16 | 2.42 | 1.94 | 15.26 | 14.71 | 2.92 | 2.78 | -2.99 | 5.09E-10 | -2.33 | 4.70E-06 | 1013 |
| Cluster-40555.204116 | 4.33 | 3.33 | 0.25 | 0.59 | 5.66 | 4.29 | 0.38 | 0.65 | -2.98 | 2.06E-03 | -3.18 | 6.89E-06 | 1398 |
| Cluster-40555.100209 | 32.95 | 26.37 | 3.05 | 3.69 | 10.41 | 9.49 | 2.29 | 2.26 | -2.98 | 1.06E-11 | -2.05 | 2.06E-03 | 905 |
| Cluster-40555.169139 | 15.33 | 12.45 | 2.05 | 1.16 | 3.81 | 3.27 | 0 | 1.25 | -2.98 | 5.20E-11 | -2.41 | 3.13E-03 | 1430 |
| Cluster-40555.197570 | 116.72 | 154.75 | 15.21 | 16.38 | 12.8 | 14.08 | 0 | 2.87 | -2.97 | 2.90E-07 | -3.15 | 1.84E-07 | 835 |
| Cluster-40555.145676 | 19.01 | 22.11 | 2.03 | 2.74 | 5.37 | 5.71 | 1.07 | 1.1 | -2.97 | 1.24E-17 | -2.29 | 3.43E-06 | 2468 |
| Cluster-40555.231170 | 3.55 | 2.79 | 0.3 | 0.43 | 2.17 | 1.65 | 0.47 | 0.91 | -2.97 | 5.97E-08 | -1.38 | 4.27E-02 | 3950 |
| Cluster-40555.182609 | 318.64 | 259.49 | 31.59 | 35.21 | 277.1 | 254.95 | 50.76 | 43.3 | -2.97 | 1.68E-32 | -2.44 | 8.12E-16 | 948 |
| Cluster-40555.131531 | 12.27 | 10.88 | 1.07 | 1.64 | 11.5 | 7.66 | 2.88 | 2.45 | -2.96 | 6.12E-06 | -1.78 | 7.07E-03 | 968 |
| Cluster-40555.175962 | 9.02 | 9.89 | 0.97 | 1.24 | 4.94 | 5.47 | 0.92 | 0.59 | -2.96 | 1.89E-03 | -2.73 | 3.05E-02 | 708 |
| Cluster-40555.231919 | 5.59 | 4.74 | 0.37 | 0.84 | 4.48 | 3.92 | 0.63 | 0.68 | -2.96 | 3.02E-02 | -2.62 | 4.62E-02 | 783 |
| Cluster-40555.172411 | 24.9 | 18.16 | 2.23 | 2.69 | 10.49 | 9.36 | 1.26 | 1.96 | -2.95 | 1.30E-07 | -2.53 | 6.70E-04 | 773 |
| Cluster-40555.188863 | 105.32 | 173.75 | 19.26 | 13.97 | 7.03 | 7.96 | 2.72 | 2.81 | -2.95 | 2.75E-03 | -1.37 | 1.09E-02 | 1867 |

| | | | | | | | | | | | | | |
|---|---|---|---|---|---|---|---|---|---|---|---|---|---|
| Cluster-40555.43910 | 3.26 | 4.77 | 0.99 | 0 | 1.37 | 0.46 | 0 | 0 | -2.95 | 3.36E-04 | 0.00 | 4.26E-02 | 1656 |
| Cluster-40555.159043 | 15.24 | 17.21 | 2.21 | 1.6 | 9.22 | 9.69 | 1.01 | 0.17 | -2.95 | 2.27E-04 | -4.05 | 1.54E-04 | 589 |
| Cluster-40555.189725 | 104.37 | 83.17 | 9.55 | 12.36 | 37.24 | 30.08 | 15.64 | 8.02 | -2.95 | 5.79E-28 | -1.45 | 9.82E-05 | 1493 |
| Cluster-40555.174463 | 21.52 | 24.17 | 2.7 | 2.71 | 16.93 | 17.61 | 2.41 | 2.64 | -2.95 | 4.97E-19 | -2.70 | 5.78E-13 | 2077 |
| Cluster-40555.190000 | 78.11 | 114.02 | 12.37 | 10.6 | 55.27 | 61.76 | 10.05 | 9.39 | -2.94 | 5.62E-05 | -2.53 | 1.12E-15 | 1999 |
| Cluster-40555.148098 | 3.16 | 4.88 | 0.24 | 0.7 | 12.45 | 10.59 | 2.13 | 0.69 | -2.94 | 1.48E-02 | -2.98 | 2.10E-07 | 1039 |
| Cluster-40555.240550 | 193.34 | 199.39 | 22.45 | 23.89 | 24.87 | 24.18 | 8.14 | 8.49 | -2.94 | 7.85E-22 | -1.50 | 3.00E-02 | 548 |
| Cluster-40555.145465 | 2.06 | 1.22 | 0 | 0.38 | 4.54 | 4 | 0.64 | 0.2 | -2.93 | 4.78E-03 | -3.31 | 2.15E-11 | 3417 |
| Cluster-40555.205978 | 10.11 | 18.78 | 1.28 | 2.16 | 12.89 | 22.56 | 2.19 | 0.65 | -2.93 | 4.38E-02 | -3.61 | 6.21E-03 | 592 |
| Cluster-40555.163599 | 34.77 | 27.46 | 3.25 | 4.1 | 12.96 | 10.71 | 2.79 | 2.21 | -2.93 | 8.61E-11 | -2.19 | 7.06E-04 | 832 |
| Cluster-40555.303301 | 6.57 | 6.57 | 0.88 | 0.7 | 3.53 | 2.65 | 0.83 | 0.38 | -2.93 | 3.87E-05 | -2.35 | 2.23E-02 | 1265 |
| Cluster-40555.191824 | 25.34 | 27.76 | 3.49 | 2.88 | 4.9 | 4.52 | 0.44 | 1.77 | -2.93 | 5.50E-16 | -1.99 | 5.81E-03 | 1395 |
| Cluster-40555.34173 | 1.87 | 1.49 | 0.14 | 0.25 | 2.87 | 3.02 | 0.18 | 0.42 | -2.93 | 2.14E-02 | -3.22 | 1.64E-05 | 1983 |
| Cluster-40555.235498 | 12.15 | 10.31 | 1.9 | 0.77 | 9.13 | 7.52 | 3.14 | 2.77 | -2.93 | 2.52E-09 | -1.42 | 1.51E-02 | 1420 |
| Cluster-40555.214392 | 10.13 | 16.9 | 1.15 | 2.1 | 20.89 | 30.37 | 4.25 | 4.27 | -2.93 | 8.43E-03 | -2.53 | 6.23E-04 | 1017 |
| Cluster-40555.240466 | 9.43 | 4.96 | 1.42 | 0.32 | 1.22 | 1.76 | 0 | 0 | -2.92 | 3.58E-03 | 0.00 | 1.27E-06 | 1967 |
| Cluster-40555.170199 | 17.88 | 22.21 | 2.64 | 2.22 | 2.96 | 2.46 | 0.12 | 0.16 | -2.91 | 4.02E-11 | -4.33 | 1.51E-07 | 2058 |
| Cluster-40555.210903 | 13.11 | 13.96 | 1.58 | 1.7 | 3.78 | 3.36 | 1.06 | 1.66 | -2.91 | 2.45E-20 | -1.31 | 1.10E-02 | 3958 |
| Cluster-40555.189563 | 14.59 | 11.04 | 1.76 | 1.35 | 9.79 | 9.5 | 1.47 | 0.68 | -2.89 | 9.78E-14 | -3.11 | 1.58E-12 | 2174 |
| Cluster-40555.197158 | 6.81 | 3.94 | 0.49 | 0.8 | 13.05 | 10.6 | 0.64 | 0.75 | -2.89 | 6.18E-04 | -4.02 | 2.32E-21 | 2355 |
| Cluster-40555.167007 | 5.74 | 3.1 | 0.25 | 0.81 | 5.67 | 4.66 | 1.03 | 1.16 | -2.89 | 7.98E-03 | -2.16 | 3.78E-03 | 1244 |
| Cluster-40555.222613 | 4.05 | 4.72 | 0.48 | 0.61 | 1.53 | 1.22 | 0.1 | 0.1 | -2.88 | 5.85E-04 | -3.90 | 1.79E-02 | 1438 |
| Cluster-40555.184040 | 266.82 | 219.21 | 26.09 | 33.45 | 376.02 | 279.9 | 76.87 | 39.76 | -2.88 | 6.17E-29 | -2.43 | 1.64E-11 | 891 |
| Cluster-40555.197035 | 226.62 | 219.18 | 33.89 | 21.56 | 697.36 | 599.58 | 55.15 | 75.48 | -2.87 | 1.20E-31 | -3.24 | 3.76E-28 | 1047 |
| Cluster-40555.185377 | 35.86 | 45 | 5.5 | 4.63 | 8.5 | 7.87 | 0.7 | 1.93 | -2.87 | 1.68E-09 | -2.57 | 6.30E-04 | 860 |
| Cluster-40555.167847 | 23.98 | 36.82 | 3.69 | 3.92 | 5.41 | 6.31 | 0.9 | 1.55 | -2.87 | 8.04E-04 | -2.21 | 2.24E-03 | 1176 |
| Cluster-40555.185777 | 20.52 | 18.09 | 1.84 | 2.93 | 10.47 | 10.19 | 1.67 | 1.62 | -2.87 | 2.15E-12 | -2.58 | 1.58E-07 | 1466 |
| Cluster-40555.194775 | 17.84 | 13.82 | 1.29 | 2.6 | 21.94 | 18.59 | 0.84 | 2.13 | -2.86 | 1.18E-15 | -3.68 | 2.04E-24 | 2499 |
| Cluster-40555.228548 | 2.15 | 1 | 0.15 | 0.22 | 5.96 | 4.51 | 0.72 | 0.74 | -2.86 | 4.69E-02 | -2.75 | 2.63E-07 | 2189 |
| Cluster-40555.114196 | 1.46 | 1.75 | 0.3 | 0.1 | 2.66 | 2.37 | 0.09 | 0 | -2.86 | 3.75E-03 | -5.77 | 3.94E-11 | 2505 |
| Cluster-40555.108605 | 2.7 | 4.55 | 0.61 | 0.31 | 1.63 | 1.56 | 0.02 | 0 | -2.86 | 6.79E-03 | -6.80 | 1.30E-06 | 2066 |
| Cluster-40555.152092 | 44.12 | 31.85 | 4.6 | 4.92 | 10.36 | 7.98 | 1.07 | 3.91 | -2.85 | 3.38E-14 | -1.78 | 4.27E-03 | 1045 |

| | | | | | | | | | | | | | |
|---|---|---|---|---|---|---|---|---|---|---|---|---|---|
| Cluster-40555.145230 | 4.55 | 4.4 | 1.13 | 0 | 13.61 | 16.3 | 0.91 | 2.03 | -2.85 | 3.55E-03 | -3.29 | 3.55E-10 | 1010 |
| Cluster-40555.225270 | 2.54 | 1.8 | 0.1 | 0.44 | 7.85 | 5.95 | 1.98 | 1.73 | -2.84 | 5.28E-05 | -1.82 | 4.47E-06 | 3826 |
| Cluster-40555.161885 | 5.71 | 6.45 | 0.43 | 1.12 | 2.47 | 2.08 | 0 | 0.18 | -2.84 | 1.86E-04 | -4.44 | 3.70E-04 | 1321 |
| Cluster-40555.146414 | 10.49 | 10.35 | 1.33 | 1.31 | 5.26 | 6.23 | 0.29 | 0.21 | -2.83 | 1.88E-09 | -4.50 | 3.04E-12 | 1722 |
| Cluster-40555.140819 | 7.21 | 6.42 | 1.09 | 0.68 | 16.95 | 15 | 1.46 | 2.64 | -2.83 | 6.09E-03 | -2.90 | 6.65E-07 | 786 |
| Cluster-40555.206771 | 21.2 | 21.64 | 3.21 | 2.27 | 3.25 | 3.59 | 0.38 | 0.64 | -2.83 | 2.21E-16 | -2.68 | 1.80E-04 | 1832 |
| Cluster-40555.184232 | 4.98 | 5.25 | 0.58 | 0.73 | 4 | 3.86 | 1.92 | 1.25 | -2.83 | 3.33E-10 | -1.25 | 2.07E-02 | 3659 |
| Cluster-40555.204060 | 3 | 3.95 | 0 | 0.89 | 15.02 | 17.84 | 1.96 | 4.76 | -2.83 | 3.35E-02 | -2.21 | 1.73E-06 | 1129 |
| Cluster-40555.192347 | 159.26 | 159.68 | 22.74 | 18.14 | 561.99 | 497.71 | 47.66 | 48.2 | -2.83 | 8.76E-28 | -3.40 | 4.27E-30 | 1029 |
| Cluster-40555.212324 | 27.74 | 20.93 | 3.39 | 2.84 | 8.49 | 6.84 | 1 | 1.59 | -2.82 | 5.94E-10 | -2.51 | 6.07E-04 | 935 |
| Cluster-40555.163260 | 113.68 | 121.84 | 14.93 | 15.5 | 16.21 | 15.56 | 2.26 | 1.79 | -2.81 | 5.67E-21 | -2.93 | 1.03E-06 | 766 |
| Cluster-40555.195912 | 4.62 | 5.75 | 0.65 | 0.72 | 7.79 | 7.12 | 0 | 0.34 | -2.81 | 3.60E-03 | -5.37 | 7.06E-11 | 1042 |
| Cluster-40555.211877 | 2.26 | 1.81 | 0.27 | 0.27 | 4.7 | 4.05 | 1.43 | 1.41 | -2.81 | 2.11E-05 | -1.55 | 7.17E-04 | 4042 |
| Cluster-40555.46068 | 8.31 | 11.63 | 0.46 | 2.1 | 6.95 | 4.4 | 0.39 | 0.13 | -2.81 | 7.03E-03 | -4.39 | 1.00E-03 | 659 |
| Cluster-40555.173360 | 12.63 | 14.2 | 1.41 | 2.07 | 8.96 | 9.11 | 3.57 | 1.32 | -2.81 | 1.30E-05 | -1.85 | 1.35E-02 | 877 |
| Cluster-40555.233485 | 12.62 | 8.43 | 1.66 | 1.08 | 5.38 | 5.08 | 1.41 | 0.7 | -2.81 | 3.26E-05 | -2.26 | 1.69E-02 | 947 |
| Cluster-40555.227841 | 68.6 | 63.73 | 9.65 | 7.56 | 17.68 | 18.46 | 5.43 | 7.01 | -2.81 | 1.08E-14 | -1.48 | 2.09E-02 | 703 |
| Cluster-40555.194999 | 6.02 | 11.25 | 1.21 | 1.07 | 4.1 | 6.39 | 0.86 | 0.27 | -2.80 | 3.47E-02 | -3.14 | 1.37E-03 | 1533 |
| Cluster-40555.169076 | 9.28 | 5.63 | 1.97 | 0 | 2.13 | 3.37 | 0 | 0 | -2.80 | 4.33E-04 | 0.00 | 6.57E-07 | 1266 |
| Cluster-40555.235291 | 12.53 | 16.65 | 1.72 | 2.09 | 4.62 | 4.49 | 0.82 | 0.31 | -2.80 | 2.24E-06 | -2.97 | 3.35E-03 | 944 |
| Cluster-40555.102956 | 21.39 | 21.93 | 1.86 | 3.74 | 38.95 | 33.61 | 4.19 | 2.83 | -2.80 | 9.38E-08 | -3.31 | 6.53E-15 | 856 |
| Cluster-40555.128647 | 5.17 | 4.33 | 0.5 | 0.69 | 5.8 | 7.44 | 0.52 | 0.72 | -2.79 | 3.42E-02 | -3.33 | 1.93E-04 | 836 |
| Cluster-40555.272493 | 5.59 | 5.95 | 0.31 | 1.18 | 2.22 | 1.81 | 0 | 0.4 | -2.79 | 6.27E-05 | -3.26 | 2.70E-03 | 1585 |
| Cluster-40555.187253 | 15.88 | 14.89 | 2.11 | 1.95 | 7.62 | 6.26 | 1.43 | 0.94 | -2.79 | 7.34E-12 | -2.50 | 3.99E-06 | 1669 |
| Cluster-40555.192623 | 7.38 | 6.35 | 0.97 | 0.84 | 13.53 | 17.87 | 0.38 | 0.65 | -2.79 | 4.81E-10 | -4.89 | 2.18E-14 | 2736 |
| Cluster-40555.197796 | 173.37 | 149.84 | 18.76 | 23.94 | 60.12 | 36.96 | 11.37 | 19.52 | -2.77 | 2.47E-23 | -1.57 | 3.49E-02 | 811 |
| Cluster-40555.205158 | 264.67 | 195.9 | 28.67 | 32.07 | 466.8 | 382.96 | 51.77 | 41.79 | -2.77 | 4.18E-17 | -3.12 | 7.34E-26 | 1170 |
| Cluster-40555.146440 | 93.63 | 79.71 | 13.12 | 9.66 | 81.31 | 83.84 | 5.92 | 8.07 | -2.77 | 1.32E-07 | -3.51 | 1.29E-10 | 396 |
| Cluster-40555.183742 | 1.71 | 2.87 | 0.5 | 0.16 | 2.75 | 2.21 | 0.51 | 0.36 | -2.77 | 3.46E-02 | -2.43 | 3.72E-02 | 1305 |
| Cluster-40555.92712 | 1.2 | 1.09 | 0.3 | 0 | 1.5 | 2.38 | 0.23 | 0.49 | -2.77 | 3.41E-02 | -2.35 | 2.22E-02 | 2239 |
| Cluster-40555.185587 | 8.7 | 9.63 | 0.69 | 1.73 | 21.94 | 19.06 | 0.16 | 1.38 | -2.76 | 5.49E-03 | -4.67 | 7.32E-14 | 716 |
| Cluster-40555.197659 | 8.91 | 6.07 | 0.72 | 1.27 | 8.98 | 7.04 | 1.78 | 1.13 | -2.76 | 1.10E-07 | -2.40 | 9.83E-09 | 2774 |

| | | | | | | | | | | | | | |
|---|---|---|---|---|---|---|---|---|---|---|---|---|---|
| Cluster-40555.307166 | 2.59 | 3.51 | 0.78 | 0.06 | 6.71 | 5.66 | 0 | 0 | -2.76 | 4.37E-02 | | 3.21E-11 | 969 |
| Cluster-40555.156450 | 41.5 | 51.65 | 6.15 | 6.39 | 28.16 | 21.26 | 6.12 | 6.68 | -2.76 | 5.93E-07 | -1.88 | 9.96E-03 | 491 |
| Cluster-40555.160170 | 3.2 | 3.28 | 0.3 | 0.59 | 6 | 6.15 | 0.81 | 1.87 | -2.75 | 1.98E-03 | -2.11 | 1.30E-04 | 1765 |
| Cluster-40555.107081 | 11.03 | 7.12 | 1.44 | 1.01 | 227.45 | 184.31 | 1.09 | 4.38 | -2.74 | 3.11E-03 | -6.15 | 1.62E-59 | 732 |
| Cluster-40555.204445 | 75.38 | 59.33 | 8.16 | 10.05 | 28.57 | 22.39 | 10.91 | 11.38 | -2.74 | 1.16E-18 | -1.12 | 1.75E-02 | 1053 |
| Cluster-40555.164505 | 11.03 | 8.8 | 0.74 | 1.82 | 9.14 | 8.59 | 0.41 | 0.69 | -2.74 | 3.16E-02 | -3.99 | 6.08E-04 | 563 |
| Cluster-40555.227222 | 5.96 | 3.88 | 0.82 | 0.52 | 9.38 | 12.18 | 2.43 | 2.02 | -2.73 | 1.08E-06 | -2.22 | 8.05E-06 | 2497 |
| Cluster-40555.202802 | 67.29 | 78.6 | 8.17 | 11.91 | 20.3 | 24.12 | 4.48 | 5.08 | -2.72 | 2.51E-14 | -2.15 | 6.09E-08 | 1360 |
| Cluster-40555.153524 | 5.41 | 4.84 | 0.76 | 0.65 | 19.37 | 13.86 | 2.21 | 2.68 | -2.71 | 2.90E-02 | -2.70 | 1.14E-06 | 817 |
| Cluster-40555.188138 | 240.07 | 182.68 | 30.28 | 28.6 | 88.55 | 84.14 | 45.86 | 43.02 | -2.70 | 7.93E-21 | -0.89 | 1.76E-02 | 2262 |
| Cluster-40555.220088 | 7.24 | 4.42 | 1.03 | 0.58 | 3.93 | 3.34 | 0.26 | 0.23 | -2.70 | 7.11E-05 | -3.83 | 9.82E-10 | 2444 |
| Cluster-40555.198349 | 15.59 | 17.35 | 2.14 | 2.48 | 10.28 | 11.98 | 4.92 | 4.29 | -2.69 | 2.98E-09 | -1.22 | 3.93E-02 | 1270 |
| Cluster-40555.226813 | 22.52 | 31.94 | 4.28 | 3.45 | 46.73 | 45.21 | 4.02 | 4.1 | -2.69 | 1.33E-04 | -3.44 | 1.94E-15 | 710 |
| Cluster-40555.237874 | 4.59 | 2.54 | 0.47 | 0.53 | 2.34 | 1.93 | 0.35 | 0.48 | -2.69 | 2.90E-03 | -2.30 | 9.17E-05 | 3967 |
| Cluster-40555.129438 | 12.63 | 13.59 | 1.39 | 2.28 | 35.75 | 36.82 | 7.85 | 5.85 | -2.69 | 3.07E-04 | -2.35 | 8.26E-08 | 749 |
| Cluster-40555.179654 | 39.21 | 36.32 | 5.82 | 4.91 | 42.28 | 46.99 | 4.13 | 10.3 | -2.68 | 2.97E-12 | -2.55 | 1.71E-11 | 911 |
| Cluster-40555.299386 | 14.08 | 14.61 | 1.79 | 2.22 | 4.52 | 3.64 | 0.92 | 0.52 | -2.68 | 7.46E-08 | -2.48 | 4.89E-03 | 1207 |
| Cluster-40555.44103 | 9.13 | 6.86 | 1.68 | 0.53 | 5.42 | 5.02 | 0.34 | 1.01 | -2.68 | 1.53E-03 | -2.86 | 5.01E-03 | 835 |
| Cluster-40555.180428 | 18.44 | 15.39 | 2.85 | 1.76 | 8.26 | 7.72 | 0.52 | 0.66 | -2.68 | 1.93E-02 | -3.69 | 4.63E-02 | 444 |
| Cluster-40555.172345 | 19.49 | 20.76 | 3.53 | 2.23 | 4.4 | 4.44 | 1.92 | 0.54 | -2.68 | 4.13E-12 | -1.79 | 2.52E-02 | 1410 |
| Cluster-40555.173962 | 11.53 | 12.69 | 2.17 | 1.31 | 22.07 | 16.23 | 6.09 | 5.17 | -2.67 | 1.36E-06 | -1.69 | 2.10E-04 | 1094 |
| Cluster-40555.169423 | 33.16 | 21.89 | 5.37 | 2.49 | 9.69 | 8.57 | 2.42 | 2.75 | -2.67 | 4.66E-07 | -1.75 | 1.68E-03 | 1302 |
| Cluster-40555.184421 | 379.36 | 394.02 | 54.92 | 56.51 | 84.13 | 74.43 | 36.81 | 42.04 | -2.66 | 2.55E-28 | -0.94 | 2.24E-02 | 903 |
| Cluster-40555.164584 | 16.64 | 11.32 | 1.43 | 2.58 | 23.88 | 20.38 | 2.58 | 3.27 | -2.65 | 9.92E-06 | -2.85 | 4.49E-10 | 967 |
| Cluster-40555.225326 | 34.42 | 28.75 | 4.04 | 5.04 | 13.82 | 12.55 | 2.91 | 2.55 | -2.65 | 5.44E-08 | -2.21 | 1.29E-03 | 718 |
| Cluster-40555.240106 | 4.91 | 2.62 | 0.67 | 0.39 | 10.81 | 9.06 | 2.72 | 3.59 | -2.64 | 5.88E-03 | -1.58 | 3.18E-03 | 1345 |
| Cluster-40555.142674 | 32.59 | 40.28 | 5.51 | 5.14 | 68.05 | 78.85 | 1.68 | 2 | -2.64 | 9.74E-09 | -5.26 | 6.50E-33 | 686 |
| Cluster-40555.221666 | 2.93 | 1.84 | 0.29 | 0.42 | 1.78 | 2.62 | 0.25 | 0.15 | -2.64 | 2.64E-02 | -3.35 | 1.33E-03 | 1611 |
| Cluster-40555.183228 | 55.07 | 58.88 | 8.7 | 7.89 | 60.47 | 70.88 | 7.12 | 8.25 | -2.64 | 8.93E-13 | -3.03 | 1.55E-15 | 742 |
| Cluster-40555.182933 | 27.23 | 43.92 | 2.84 | 7.52 | 12.99 | 11.56 | 4.62 | 3.46 | -2.64 | 1.45E-02 | -1.55 | 1.31E-02 | 932 |
| Cluster-40555.189145 | 9.86 | 11.77 | 1.2 | 1.98 | 27.21 | 21.44 | 2.49 | 3.96 | -2.63 | 2.95E-07 | -2.84 | 9.03E-14 | 1464 |
| Cluster-40555.186321 | 317.75 | 339.63 | 53 | 43.96 | 50.21 | 43.28 | 18.06 | 18.67 | -2.63 | 3.22E-27 | -1.28 | 1.91E-03 | 851 |

| | | | | | | | | | | | | | |
|---|---|---|---|---|---|---|---|---|---|---|---|---|---|
| Cluster-40555.181858 | 17.72 | 12.25 | 2.12 | 2.26 | 4.24 | 4.1 | 1.55 | 1.9 | -2.62 | 6.03E-09 | -1.21 | 4.23E-02 | 2824 |
| Cluster-40555.248918 | 6.11 | 4.18 | 0.59 | 0.89 | 4.24 | 4.22 | 0.94 | 1.09 | -2.62 | 7.87E-03 | -1.98 | 4.54E-02 | 1070 |
| Cluster-40555.219389 | 3.53 | 5.4 | 1.14 | 0.21 | 1.52 | 2.09 | 0 | 0 | -2.62 | 2.55E-03 | 0.00 | 1.08E-07 | 1896 |
| Cluster-40555.166777 | 9.38 | 8.45 | 1.7 | 0.98 | 12.5 | 7.75 | 3.33 | 3.36 | -2.62 | 6.01E-06 | -1.52 | 1.60E-02 | 1290 |
| Cluster-40555.132590 | 31.18 | 25.45 | 4.05 | 4.31 | 14.86 | 12.68 | 3.61 | 3.17 | -2.62 | 9.52E-14 | -1.96 | 1.36E-05 | 1424 |
| Cluster-40555.155056 | 54.33 | 42.83 | 6.57 | 7.7 | 10.9 | 9.64 | 2.08 | 3.02 | -2.62 | 7.36E-15 | -1.93 | 1.08E-03 | 1056 |
| Cluster-40555.174552 | 8.03 | 9.49 | 1.76 | 0.84 | 20.58 | 21.77 | 0.85 | 1.47 | -2.62 | 2.39E-04 | -4.08 | 2.97E-16 | 958 |
| Cluster-40555.195320 | 14.25 | 11.84 | 2.15 | 1.73 | 8.7 | 7.57 | 2.08 | 2.62 | -2.62 | 1.81E-09 | -1.71 | 1.03E-03 | 1619 |
| Cluster-40555.181962 | 106.81 | 92.92 | 17.82 | 12.01 | 121.02 | 106.85 | 14.33 | 15.75 | -2.61 | 2.83E-17 | -2.85 | 6.23E-17 | 735 |
| Cluster-40555.204958 | 10.19 | 9.1 | 0 | 2.79 | 14.19 | 14.63 | 2.23 | 1.92 | -2.60 | 8.11E-04 | -2.74 | 2.60E-07 | 982 |
| Cluster-40555.185794 | 81.84 | 73.95 | 12.51 | 10.82 | 37.44 | 23.4 | 12.13 | 11.57 | -2.60 | 6.48E-18 | -1.29 | 3.79E-02 | 957 |
| Cluster-40555.183147 | 403.99 | 426.5 | 62.82 | 62.33 | 539.24 | 579.02 | 63.68 | 119.7 | -2.59 | 1.50E-28 | -2.53 | 2.95E-18 | 1113 |
| Cluster-40555.192283 | 9.79 | 6.58 | 1.08 | 1.37 | 7.35 | 5.43 | 0.56 | 2.81 | -2.59 | 6.00E-07 | -1.82 | 1.25E-02 | 1805 |
| Cluster-40555.164863 | 23.94 | 21.05 | 3.65 | 3.09 | 17.74 | 16.47 | 5.74 | 4.98 | -2.59 | 1.29E-05 | -1.62 | 1.65E-02 | 667 |
| Cluster-40555.179200 | 3.56 | 3.26 | 0.47 | 0.58 | 5.5 | 6.21 | 0.31 | 1.11 | -2.59 | 4.58E-02 | -2.94 | 1.07E-04 | 1088 |
| Cluster-40555.205542 | 31.94 | 22.05 | 4.2 | 3.96 | 15.19 | 13.27 | 6.05 | 4.81 | -2.58 | 5.95E-09 | -1.32 | 8.34E-04 | 2550 |
| Cluster-40555.242098 | 58.8 | 36.15 | 7.61 | 6.62 | 35.04 | 24.78 | 10.21 | 10.41 | -2.58 | 1.48E-05 | -1.47 | 2.70E-02 | 512 |
| Cluster-40555.187123 | 8.33 | 7.01 | 2.38 | 0 | 26.18 | 28.27 | 0.2 | 1.06 | -2.58 | 1.45E-03 | -5.42 | 4.16E-23 | 830 |
| Cluster-40555.212985 | 17.69 | 20.43 | 2.94 | 2.89 | 16.13 | 16.41 | 1.99 | 3.33 | -2.57 | 1.49E-05 | -2.54 | 2.14E-05 | 740 |
| Cluster-40555.189143 | 36.89 | 28.1 | 5.34 | 4.59 | 37.02 | 38.14 | 7.62 | 11.33 | -2.56 | 2.47E-09 | -1.92 | 3.35E-06 | 828 |
| Cluster-40555.221385 | 13.24 | 11.45 | 1.75 | 2.06 | 7.82 | 8.34 | 1.99 | 2.71 | -2.56 | 7.81E-10 | -1.71 | 3.97E-04 | 1927 |
| Cluster-40555.126184 | 4.85 | 3.75 | 0.39 | 0.92 | 5.57 | 5.02 | 1.65 | 1.7 | -2.56 | 2.82E-05 | -1.59 | 2.04E-03 | 2497 |
| Cluster-40555.225344 | 12.7 | 9.84 | 1.92 | 1.49 | 6.96 | 5.65 | 0.45 | 1.3 | -2.56 | 2.34E-04 | -2.77 | 1.50E-03 | 862 |
| Cluster-40555.227426 | 12.95 | 18.3 | 2.37 | 2.51 | 15.52 | 17.21 | 1.61 | 1.2 | -2.55 | 1.28E-04 | -3.49 | 6.48E-17 | 1700 |
| Cluster-40555.118553 | 2.85 | 2.53 | 0.49 | 0.36 | 2.81 | 1.44 | 0.42 | 0.38 | -2.53 | 1.74E-02 | -2.35 | 4.70E-02 | 1553 |
| Cluster-40555.199057 | 3.91 | 5.06 | 0.47 | 0.93 | 1.75 | 0.93 | 0.15 | 0.2 | -2.53 | 1.89E-04 | -2.80 | 2.30E-02 | 1957 |
| Cluster-40555.185512 | 58.32 | 64.26 | 8.5 | 10.83 | 37.77 | 36.28 | 4.59 | 5.03 | -2.53 | 8.17E-18 | -2.88 | 1.64E-15 | 1307 |
| Cluster-40555.224844 | 3.85 | 2.98 | 0.61 | 0.45 | 2.81 | 2.11 | 0 | 0.38 | -2.52 | 8.49E-04 | -3.54 | 2.17E-05 | 1971 |
| Cluster-40555.210806 | 4.12 | 2.45 | 0.54 | 0.49 | 1.64 | 2.39 | 0.29 | 0.3 | -2.52 | 2.13E-03 | -2.75 | 4.92E-03 | 1870 |
| Cluster-40555.202959 | 14.63 | 14.24 | 0.46 | 4.08 | 36.84 | 33.51 | 4.45 | 6.35 | -2.52 | 1.38E-03 | -2.63 | 5.22E-09 | 718 |
| Cluster-40555.189035 | 517.74 | 544.38 | 82.3 | 86.61 | 134.65 | 130.34 | 56.83 | 70.64 | -2.51 | 1.44E-28 | -0.98 | 5.21E-03 | 1686 |
| Cluster-40555.165371 | 17.28 | 14.76 | 2.57 | 2.49 | 22.77 | 29.43 | 4.79 | 6.18 | -2.51 | 1.33E-02 | -2.20 | 1.29E-03 | 500 |

| | | | | | | | | | | | | | |
|---|---|---|---|---|---|---|---|---|---|---|---|---|---|
| Cluster-40555.152518 | 7.59 | 6.9 | 1.14 | 1.17 | 9.06 | 5.69 | 0.86 | 0.94 | -2.51 | 4.13E-04 | -2.92 | 1.57E-05 | 1180 |
| Cluster-40555.194830 | 2.32 | 4.1 | 1.01 | 0.04 | 5.39 | 3.45 | 0 | 0.37 | -2.50 | 4.14E-02 | -4.47 | 1.67E-09 | 2075 |
| Cluster-40555.208868 | 16.18 | 14.51 | 2.52 | 2.4 | 4.12 | 4.79 | 0.63 | 0.97 | -2.50 | 3.08E-11 | -2.40 | 7.35E-05 | 1942 |
| Cluster-40555.51978 | 7.24 | 10.67 | 2.33 | 0.63 | 14.09 | 7.78 | 0.33 | 0.34 | -2.49 | 2.93E-02 | -4.96 | 2.01E-05 | 578 |
| Cluster-40555.193577 | 38.1 | 29.83 | 5.25 | 5.67 | 16.77 | 15.66 | 5.16 | 5.54 | -2.49 | 6.02E-12 | -1.53 | 1.68E-03 | 1141 |
| Cluster-40555.192459 | 45.99 | 63.11 | 8.77 | 9.03 | 17.55 | 20.13 | 3.74 | 5.18 | -2.48 | 2.77E-05 | -2.01 | 2.55E-08 | 2218 |
| Cluster-40555.158465 | 7.07 | 7.75 | 1.6 | 0.88 | 9.67 | 7.81 | 0.36 | 0.14 | -2.48 | 3.43E-02 | -5.02 | 2.54E-06 | 661 |
| Cluster-40555.229398 | 19.21 | 17.25 | 3.21 | 2.74 | 1.42 | 0.76 | 0 | 0 | -2.48 | 2.32E-07 | 0.00 | 4.92E-02 | 1030 |
| Cluster-40555.210403 | 9.23 | 7.41 | 1.43 | 1.28 | 16.15 | 16.42 | 3.05 | 2.66 | -2.48 | 2.14E-06 | -2.45 | 1.38E-09 | 1672 |
| Cluster-40555.49723 | 2.06 | 1.63 | 0.45 | 0.16 | 1.29 | 0.94 | 0.14 | 0.15 | -2.47 | 1.98E-02 | -2.88 | 3.78E-02 | 2032 |
| Cluster-40555.107082 | 17.75 | 12.56 | 3.21 | 1.74 | 311.86 | 267.18 | 0.84 | 3.96 | -2.47 | 1.32E-03 | -6.83 | 1.08E-67 | 626 |
| Cluster-40555.206811 | 58.19 | 59.73 | 10.26 | 9.06 | 59.09 | 60.07 | 2.71 | 1.1 | -2.47 | 1.70E-09 | -4.92 | 3.28E-24 | 603 |
| Cluster-40555.209940 | 9.45 | 6.34 | 1.24 | 1.34 | 7.5 | 6.76 | 2.51 | 2.16 | -2.47 | 1.45E-06 | -1.54 | 3.60E-03 | 1864 |
| Cluster-40555.183597 | 156.83 | 159.8 | 25.69 | 26.21 | 157.15 | 114.35 | 37.93 | 34.95 | -2.47 | 2.49E-17 | -1.83 | 8.88E-08 | 657 |
| Cluster-40555.191709 | 3.15 | 2.34 | 0.36 | 0.54 | 3.33 | 2.71 | 1.02 | 0.91 | -2.47 | 9.27E-05 | -1.57 | 5.68E-03 | 3414 |
| Cluster-40555.213041 | 5.89 | 4.36 | 1.06 | 0.62 | 12.63 | 9.92 | 5.47 | 6.08 | -2.47 | 2.35E-09 | -0.89 | 4.13E-02 | 3946 |
| Cluster-40555.143230 | 4.39 | 4.25 | 0.29 | 1.13 | 3.6 | 4.22 | 0.69 | 0.72 | -2.47 | 3.40E-03 | -2.42 | 1.08E-03 | 1574 |
| Cluster-40555.171450 | 5.43 | 5.02 | 0.29 | 1.38 | 4.9 | 4.51 | 0.97 | 1.2 | -2.47 | 5.67E-03 | -2.06 | 8.77E-03 | 1268 |
| Cluster-40555.224352 | 8.21 | 6.02 | 1.4 | 0.92 | 5.16 | 5.19 | 1.27 | 1.6 | -2.46 | 8.05E-05 | -1.78 | 1.28E-02 | 1389 |
| Cluster-40555.292328 | 6.35 | 10.24 | 1.34 | 1.43 | 5.72 | 4.77 | 1.65 | 1.16 | -2.46 | 1.13E-02 | -1.82 | 1.92E-02 | 1235 |
| Cluster-40555.148131 | 41.13 | 28.02 | 5.11 | 6.21 | 14.93 | 12.12 | 0.17 | 0.35 | -2.46 | 1.12E-07 | -5.60 | 6.19E-20 | 1144 |
| Cluster-40555.177474 | 46.76 | 54.77 | 8.28 | 8.58 | 22.19 | 21.41 | 4.51 | 3.99 | -2.46 | 2.32E-13 | -2.30 | 2.04E-08 | 1253 |
| Cluster-40555.206724 | 22.95 | 20.04 | 2.85 | 4.23 | 12.17 | 8.93 | 2.59 | 1.95 | -2.45 | 4.61E-05 | -2.16 | 7.24E-03 | 699 |
| Cluster-40555.192471 | 33.21 | 27.47 | 3.16 | 6.83 | 89.92 | 79.01 | 16.65 | 16.55 | -2.45 | 1.24E-14 | -2.28 | 9.43E-14 | 1987 |
| Cluster-40555.186708 | 86.69 | 101.41 | 16.21 | 15.25 | 91.56 | 92.07 | 6.56 | 7.73 | -2.44 | 1.11E-13 | -3.61 | 8.62E-28 | 1188 |
| Cluster-40555.198444 | 11.77 | 7.56 | 1.36 | 1.82 | 14.83 | 12.01 | 5.08 | 7.27 | -2.44 | 3.57E-05 | -1.04 | 2.30E-02 | 2021 |
| Cluster-40555.194198 | 11.66 | 14.95 | 2.42 | 2.02 | 8.58 | 9.83 | 0.92 | 1.81 | -2.44 | 4.13E-07 | -2.70 | 9.97E-07 | 1273 |
| Cluster-40555.212507 | 80.48 | 88.19 | 16.73 | 11.64 | 291.24 | 273.21 | 17.97 | 28.22 | -2.44 | 1.32E-17 | -3.54 | 8.67E-31 | 994 |
| Cluster-40555.189709 | 38.28 | 37.84 | 6.21 | 6.58 | 90.7 | 70.85 | 7.9 | 10.34 | -2.43 | 7.37E-17 | -3.07 | 3.66E-23 | 1910 |
| Cluster-40555.221164 | 14.57 | 16.16 | 2.43 | 2.77 | 5.2 | 5.46 | 1.8 | 1.47 | -2.43 | 1.72E-10 | -1.64 | 5.44E-03 | 1911 |
| Cluster-40555.215049 | 57.59 | 59.84 | 9.16 | 10.64 | 20.22 | 19.39 | 5.81 | 3.47 | -2.42 | 1.02E-07 | -2.03 | 6.28E-03 | 537 |
| Cluster-40555.198938 | 27.12 | 28.54 | 4.54 | 4.91 | 9.98 | 12.36 | 3.33 | 3.34 | -2.42 | 6.26E-08 | -1.69 | 1.30E-02 | 860 |

| | | | | | | | | | | | | | |
|---|---|---|---|---|---|---|---|---|---|---|---|---|---|
| Cluster-40555.189267 | 13.81 | 22.13 | 2.57 | 3.54 | 15.44 | 13.76 | 1.41 | 1.19 | -2.41 | 1.29E-02 | -3.42 | 4.40E-12 | 1188 |
| Cluster-40555.195642 | 40.32 | 32.04 | 4.74 | 7.53 | 14.53 | 9.05 | 4.78 | 4.08 | -2.41 | 9.36E-11 | -1.34 | 3.61E-02 | 1060 |
| Cluster-40555.164408 | 30.95 | 38.03 | 5.24 | 6.57 | 24 | 21.91 | 6.67 | 5.01 | -2.41 | 6.17E-09 | -1.91 | 2.60E-07 | 1725 |
| Cluster-40555.171626 | 6.83 | 5.94 | 1.51 | 0.68 | 4.74 | 3.5 | 1.22 | 0.43 | -2.41 | 4.15E-08 | -2.27 | 2.29E-05 | 2702 |
| Cluster-40555.207205 | 37.56 | 26.82 | 6.2 | 4.79 | 27.02 | 21.33 | 6.58 | 7.09 | -2.40 | 9.49E-11 | -1.75 | 1.70E-05 | 1201 |
| Cluster-40555.230251 | 2.04 | 2.84 | 0.3 | 0.54 | 4.56 | 4.4 | 0.62 | 1.31 | -2.40 | 1.00E-03 | -2.13 | 1.30E-05 | 2976 |
| Cluster-40555.182099 | 8.52 | 6.87 | 0.86 | 1.77 | 6.99 | 3.77 | 0.5 | 1.33 | -2.40 | 6.14E-03 | -2.43 | 2.25E-02 | 931 |
| Cluster-40555.168429 | 21.16 | 20.12 | 3.32 | 3.77 | 20.98 | 16.31 | 7.32 | 8.7 | -2.40 | 3.87E-09 | -1.15 | 1.55E-02 | 1285 |
| Cluster-40555.198949 | 40.91 | 50.24 | 8.72 | 7.04 | 14.3 | 11.6 | 4.98 | 4.48 | -2.40 | 7.46E-09 | -1.39 | 3.57E-02 | 869 |
| Cluster-40555.220416 | 114.73 | 97.35 | 14.75 | 21.45 | 234.84 | 191.81 | 34.79 | 35.28 | -2.40 | 1.26E-11 | -2.54 | 1.24E-14 | 574 |
| Cluster-40555.193292 | 7.33 | 8.42 | 1.47 | 1.3 | 5.58 | 3.94 | 1.13 | 1.22 | -2.39 | 4.02E-04 | -1.94 | 2.23E-02 | 1156 |
| Cluster-40555.238395 | 5.82 | 4.99 | 0.42 | 1.4 | 3.79 | 2.94 | 0.25 | 0.5 | -2.39 | 3.09E-02 | -3.11 | 9.14E-03 | 967 |
| Cluster-40555.187226 | 23.98 | 19.15 | 4.36 | 3.11 | 24.01 | 18.17 | 3.02 | 2.76 | -2.39 | 1.17E-15 | -2.79 | 3.50E-16 | 2694 |
| Cluster-40555.189001 | 834.33 | 802.53 | 115.42 | 167.44 | 440.77 | 470.33 | 90.19 | 119.67 | -2.39 | 7.33E-25 | -2.05 | 3.94E-12 | 1060 |
| Cluster-40555.200916 | 1.76 | 2.2 | 0.13 | 0.55 | 1.64 | 1.82 | 0.26 | 0.3 | -2.38 | 5.14E-03 | -2.61 | 5.39E-04 | 3084 |
| Cluster-40555.228833 | 6.26 | 6.23 | 0.46 | 1.68 | 2.68 | 2.38 | 0.19 | 0 | -2.38 | 4.84E-04 | -4.66 | 1.35E-05 | 1548 |
| Cluster-40555.133769 | 56.42 | 64.09 | 12.66 | 8.37 | 179.39 | 134.68 | 18 | 20.29 | -2.38 | 9.86E-13 | -2.96 | 5.67E-20 | 819 |
| Cluster-40555.196752 | 17.62 | 18.52 | 3.53 | 2.8 | 13.79 | 13.97 | 3.79 | 3.2 | -2.38 | 3.15E-11 | -1.93 | 3.13E-06 | 1814 |
| Cluster-40555.189068 | 17.06 | 10.54 | 2.73 | 2.08 | 21.84 | 19.04 | 4.69 | 2.59 | -2.38 | 1.41E-04 | -2.43 | 3.62E-07 | 926 |
| Cluster-40555.179158 | 10.05 | 10.35 | 1.12 | 2.42 | 7.21 | 7.62 | 0.36 | 1.56 | -2.38 | 3.18E-08 | -2.85 | 6.63E-10 | 2234 |
| Cluster-40555.97154 | 12.19 | 6.12 | 1.63 | 1.55 | 5.3 | 4.33 | 1.66 | 0.89 | -2.38 | 3.34E-02 | -1.86 | 4.97E-03 | 1658 |
| Cluster-40555.158562 | 21.15 | 16.77 | 3.45 | 3.18 | 21.93 | 18.38 | 7.67 | 5.55 | -2.37 | 5.10E-04 | -1.55 | 1.84E-02 | 636 |
| Cluster-40555.170601 | 7.85 | 9.18 | 0.92 | 2.02 | 15.49 | 15.98 | 1.95 | 4.75 | -2.37 | 2.71E-04 | -2.15 | 1.73E-06 | 1238 |
| Cluster-40555.198238 | 12.8 | 9.45 | 2.27 | 1.56 | 10.2 | 8.41 | 2.04 | 0.73 | -2.36 | 1.25E-02 | -2.70 | 4.48E-03 | 634 |
| Cluster-40555.217924 | 5 | 4.01 | 0.42 | 1.13 | 2.45 | 1.82 | 0.31 | 0.44 | -2.35 | 4.50E-03 | -2.40 | 2.98E-02 | 1539 |
| Cluster-40555.191760 | 7.34 | 9.68 | 1.97 | 1.09 | 9.25 | 8.45 | 2.69 | 1.7 | -2.35 | 1.66E-02 | -1.95 | 3.57E-02 | 698 |
| Cluster-40555.200716 | 10.86 | 8.89 | 3.59 | 0 | 49.02 | 15.56 | 0 | 0 | -2.35 | 5.00E-03 | 0.00 | 1.36E-03 | 674 |
| Cluster-40555.215669 | 84.72 | 57.66 | 12.72 | 12.55 | 35.63 | 41.29 | 17.92 | 15.89 | -2.35 | 9.07E-08 | -1.13 | 1.26E-02 | 917 |
| Cluster-40555.194152 | 13.46 | 11.17 | 2.71 | 1.69 | 10.29 | 8.28 | 3.5 | 3.56 | -2.34 | 7.51E-08 | -1.32 | 1.32E-02 | 1599 |
| Cluster-40555.148419 | 10.85 | 12.66 | 2.99 | 1.3 | 15.18 | 14.19 | 6.32 | 2.34 | -2.34 | 4.84E-04 | -1.71 | 5.80E-03 | 819 |
| Cluster-40555.102492 | 23.17 | 22.99 | 4.89 | 3.43 | 7.51 | 7.59 | 0.86 | 0.92 | -2.34 | 2.41E-09 | -3.04 | 1.67E-06 | 1196 |
| Cluster-40555.178815 | 13.71 | 19.16 | 2.54 | 3.43 | 9.09 | 13.84 | 2.26 | 1.92 | -2.32 | 4.47E-04 | -2.41 | 5.50E-03 | 1207 |

| | | | | | | | | | | | | | |
|---|---|---|---|---|---|---|---|---|---|---|---|---|---|
| Cluster-40555.175889 | 9.27 | 10.89 | 2.35 | 1.37 | 8.61 | 8.29 | 2.77 | 1.63 | -2.32 | 8.40E-10 | -1.88 | 1.18E-05 | 2526 |
| Cluster-40555.38323 | 7.59 | 7.22 | 1.93 | 0.8 | 3.18 | 4.45 | 0.23 | 0 | -2.31 | 2.91E-02 | -5.04 | 3.14E-03 | 709 |
| Cluster-40555.193601 | 16.28 | 15.76 | 2.05 | 3.79 | 49.79 | 51.32 | 4.7 | 6.68 | -2.31 | 1.15E-06 | -3.08 | 8.08E-19 | 1235 |
| Cluster-40555.196011 | 6.15 | 5.07 | 0.96 | 1.11 | 12.33 | 11.37 | 5.23 | 4.39 | -2.31 | 1.35E-03 | -1.24 | 1.76E-02 | 1446 |
| Cluster-40555.181710 | 14.05 | 10.82 | 2.67 | 1.91 | 9.62 | 11.2 | 0.69 | 1.65 | -2.30 | 8.87E-06 | -3.07 | 2.68E-08 | 1183 |
| Cluster-40555.150888 | 9.38 | 8.93 | 1.89 | 1.49 | 6.49 | 8.42 | 1.84 | 1.98 | -2.30 | 1.19E-09 | -1.90 | 7.35E-05 | 2879 |
| Cluster-40555.165420 | 42.14 | 32.35 | 6.37 | 7.27 | 25.68 | 21.57 | 10.51 | 7.33 | -2.30 | 1.42E-09 | -1.35 | 5.25E-03 | 950 |
| Cluster-40555.218133 | 33.66 | 39.85 | 8.9 | 4.74 | 9.33 | 7.43 | 0.7 | 0 | -2.30 | 9.49E-10 | -4.55 | 2.10E-09 | 987 |
| Cluster-40555.206195 | 222.2 | 212.06 | 39.07 | 41.02 | 75.59 | 71.01 | 36.71 | 34.84 | -2.30 | 3.15E-20 | -0.97 | 1.67E-02 | 998 |
| Cluster-40555.226517 | 2.3 | 1.24 | 0.38 | 0.27 | 5.47 | 5.32 | 0.72 | 0.54 | -2.29 | 2.72E-02 | -3.03 | 5.58E-09 | 2342 |
| Cluster-40555.190419 | 15.68 | 9.77 | 3.05 | 1.66 | 434.97 | 350.2 | 29.31 | 13.2 | -2.29 | 2.34E-04 | -4.15 | 3.78E-41 | 1158 |
| Cluster-40555.203533 | 26.66 | 31.58 | 5.25 | 5.6 | 14.42 | 15.23 | 3.96 | 3.27 | -2.29 | 2.97E-11 | -1.97 | 3.85E-06 | 1554 |
| Cluster-40555.206680 | 63.36 | 95.26 | 16.87 | 13.01 | 20.33 | 15.64 | 8.16 | 5.91 | -2.28 | 2.36E-03 | -1.29 | 1.23E-03 | 2084 |
| Cluster-40555.208481 | 13.61 | 8.92 | 2.7 | 1.51 | 16.91 | 14.87 | 3.54 | 3.7 | -2.28 | 1.79E-05 | -2.06 | 8.18E-08 | 1986 |
| Cluster-40555.190723 | 15.6 | 13.79 | 2.91 | 2.59 | 15.45 | 15.16 | 2.49 | 2.44 | -2.28 | 5.58E-11 | -2.56 | 4.52E-12 | 2405 |
| Cluster-40555.142633 | 2.36 | 3.71 | 0.94 | 0.24 | 9.56 | 8.06 | 0.72 | 1.2 | -2.27 | 2.06E-02 | -3.13 | 8.06E-09 | 1395 |
| Cluster-40555.130221 | 2.49 | 1.47 | 0.75 | 0 | 4.33 | 3.83 | 0.65 | 1.22 | -2.27 | 3.39E-02 | -2.03 | 2.75E-03 | 1731 |
| Cluster-40555.199280 | 50.98 | 58.49 | 10.45 | 10.18 | 187.03 | 207.4 | 7.83 | 5.69 | -2.27 | 1.06E-15 | -4.81 | 1.07E-52 | 2644 |
| Cluster-40555.213968 | 24.93 | 18 | 4.48 | 3.59 | 20.22 | 14.56 | 3.53 | 1.61 | -2.27 | 3.18E-05 | -2.70 | 3.24E-06 | 753 |
| Cluster-40555.209780 | 44.57 | 59.7 | 9.51 | 9.42 | 58.48 | 42.91 | 9.65 | 9.22 | -2.27 | 6.96E-10 | -2.14 | 5.09E-07 | 826 |
| Cluster-40555.218711 | 10.57 | 6.91 | 1.13 | 2.13 | 7.57 | 5.31 | 1.71 | 0.89 | -2.27 | 2.73E-04 | -2.24 | 4.74E-04 | 1318 |
| Cluster-40555.157147 | 68.46 | 61.78 | 13.15 | 11.38 | 27.94 | 26.78 | 7.22 | 3.31 | -2.26 | 4.39E-09 | -2.33 | 2.39E-05 | 617 |
| Cluster-40555.185221 | 188.93 | 210.61 | 39.32 | 36.54 | 87.09 | 76.27 | 41.1 | 41.18 | -2.26 | 1.27E-19 | -0.92 | 1.89E-02 | 1210 |
| Cluster-40555.235127 | 5.96 | 6.66 | 1.32 | 1.1 | 4.24 | 5.03 | 0.73 | 1.19 | -2.26 | 3.97E-04 | -2.20 | 1.65E-03 | 1489 |
| Cluster-40555.175847 | 8.61 | 10.22 | 1.23 | 2.39 | 16.05 | 11.83 | 2.54 | 2.2 | -2.26 | 1.97E-03 | -2.50 | 2.74E-06 | 984 |
| Cluster-40555.182636 | 22.1 | 12.09 | 1.53 | 4.88 | 6.71 | 5.55 | 1.3 | 1.72 | -2.24 | 4.72E-02 | -1.95 | 6.09E-04 | 1677 |
| Cluster-40555.181884 | 19.58 | 30.8 | 4.89 | 4.87 | 41.98 | 56.25 | 11.22 | 5.76 | -2.24 | 8.91E-03 | -2.49 | 2.88E-05 | 3318 |
| Cluster-40555.266901 | 13.15 | 8.72 | 3.44 | 0.86 | 11.75 | 13.21 | 1.53 | 1.66 | -2.24 | 5.37E-03 | -2.89 | 6.65E-05 | 696 |
| Cluster-40555.216505 | 22.7 | 15.24 | 3.14 | 4.09 | 19.99 | 23.19 | 5.18 | 4.02 | -2.24 | 5.90E-06 | -2.17 | 1.12E-07 | 1289 |
| Cluster-40555.193270 | 3.32 | 2.84 | 0.79 | 0.41 | 7.52 | 6.76 | 2.34 | 1.14 | -2.23 | 4.14E-04 | -1.98 | 7.69E-06 | 2692 |
| Cluster-40555.246700 | 15.65 | 12.42 | 1.52 | 3.82 | 81.62 | 62.39 | 10.42 | 14 | -2.23 | 4.76E-03 | -2.49 | 1.95E-11 | 708 |
| Cluster-40555.179066 | 143.05 | 150.18 | 24.52 | 32.11 | 59.2 | 49.94 | 24.21 | 22.46 | -2.23 | 1.12E-15 | -1.16 | 5.63E-03 | 819 |

| | | | | | | | | | | | | | |
|---|---|---|---|---|---|---|---|---|---|---|---|---|---|
| Cluster-40555.180198 | 10.3 | 5.97 | 1.93 | 1.26 | 10.17 | 9.4 | 1.7 | 2.84 | -2.21 | 3.16E-02 | -2.04 | 1.20E-02 | 721 |
| Cluster-40555.189282 | 3.02 | 3.77 | 0.52 | 0.8 | 7.95 | 7.8 | 0.2 | 0.33 | -2.21 | 1.61E-03 | -4.86 | 1.65E-21 | 2345 |
| Cluster-40555.199648 | 45.14 | 36.83 | 8.89 | 7.31 | 15.59 | 12.7 | 4.1 | 5.58 | -2.20 | 4.85E-12 | -1.48 | 2.33E-03 | 1263 |
| Cluster-40555.207234 | 16.61 | 28.57 | 4.79 | 4.25 | 6.81 | 8.66 | 2.55 | 1.93 | -2.20 | 4.20E-02 | -1.73 | 1.79E-03 | 1550 |
| Cluster-40555.197036 | 8.24 | 11.72 | 3.01 | 1.04 | 73.56 | 67.41 | 3.85 | 4.06 | -2.20 | 6.41E-03 | -4.10 | 8.66E-25 | 730 |
| Cluster-40555.213068 | 4.12 | 3.67 | 1.35 | 0.22 | 8.85 | 10.69 | 0.41 | 0.36 | -2.19 | 2.55E-05 | -4.60 | 7.70E-23 | 2704 |
| Cluster-40555.259148 | 19.03 | 15.93 | 3.2 | 3.75 | 28.02 | 25.04 | 7.16 | 6.06 | -2.19 | 1.27E-10 | -1.94 | 1.27E-08 | 2288 |
| Cluster-40555.179790 | 18.65 | 21.48 | 2.54 | 5.46 | 25 | 24.43 | 4.85 | 4.04 | -2.18 | 5.64E-04 | -2.40 | 3.81E-06 | 703 |
| Cluster-40555.246009 | 8.47 | 11.05 | 2.27 | 1.72 | 5.18 | 5.65 | 1.77 | 1.59 | -2.17 | 8.67E-05 | -1.63 | 3.42E-02 | 1276 |
| Cluster-40555.228187 | 22.84 | 19.06 | 4.75 | 3.73 | 10.56 | 9.13 | 3.76 | 4.22 | -2.16 | 1.21E-08 | -1.23 | 3.33E-02 | 1389 |
| Cluster-40555.221483 | 19.57 | 14.51 | 5.86 | 1.19 | 3.19 | 5.76 | 0 | 0 | -2.15 | 1.66E-05 | 0.00 | 1.27E-05 | 1136 |
| Cluster-40555.192646 | 58.29 | 59.26 | 11.96 | 12.25 | 31.59 | 20.97 | 2.83 | 4.01 | -2.14 | 1.62E-08 | -2.86 | 2.12E-07 | 680 |
| Cluster-40555.135172 | 6.29 | 8.07 | 0.54 | 2.37 | 2.5 | 3.48 | 0 | 0 | -2.13 | 3.23E-02 | 0.00 | 6.86E-05 | 901 |
| Cluster-40555.199015 | 11.22 | 11.57 | 2.34 | 2.3 | 10.84 | 10.26 | 1.71 | 2.05 | -2.13 | 2.10E-02 | -2.43 | 4.17E-03 | 652 |
| Cluster-40555.195509 | 4.28 | 2.63 | 0.61 | 0.8 | 4.69 | 4.05 | 0.81 | 0.4 | -2.13 | 1.54E-03 | -2.80 | 8.51E-08 | 2746 |
| Cluster-40555.197936 | 5.69 | 2.99 | 0.89 | 0.88 | 8.2 | 7.53 | 1.07 | 1.36 | -2.13 | 3.41E-02 | -2.63 | 3.65E-07 | 1639 |
| Cluster-40555.140143 | 15.61 | 9.18 | 2.21 | 2.92 | 1.52 | 1.45 | 0 | 0 | -2.12 | 4.03E-03 | 0.00 | 3.06E-02 | 879 |
| Cluster-40555.180392 | 103.34 | 89.45 | 18.42 | 22.04 | 50.92 | 42.25 | 18.79 | 20.68 | -2.10 | 8.06E-13 | -1.17 | 5.15E-03 | 897 |
| Cluster-40555.167460 | 1.79 | 1.68 | 0.77 | 0 | 0.92 | 0.8 | 0 | 0 | -2.10 | 4.94E-02 | 0.00 | 1.26E-03 | 1911 |
| Cluster-40555.226306 | 22.09 | 17.75 | 4.23 | 4.15 | 42.59 | 42.19 | 8.08 | 7.57 | -2.10 | 9.67E-08 | -2.37 | 6.66E-12 | 1380 |
| Cluster-40555.45026 | 22.81 | 22.4 | 4.93 | 4.65 | 8.27 | 7.46 | 1.89 | 2.28 | -2.10 | 8.79E-05 | -1.85 | 4.86E-02 | 748 |
| Cluster-40555.195516 | 12.95 | 7.51 | 1.94 | 2.36 | 7.76 | 7 | 0 | 0.79 | -2.09 | 7.12E-03 | -4.11 | 4.63E-11 | 1374 |
| Cluster-40555.162723 | 33.6 | 55.73 | 8.93 | 10.22 | 52.71 | 35.82 | 11.66 | 3.12 | -2.09 | 3.60E-02 | -2.53 | 1.89E-05 | 3459 |
| Cluster-40555.250621 | 2.2 | 1.7 | 0 | 0.8 | 1.74 | 1.47 | 0 | 0.05 | -2.09 | 4.70E-02 | -5.70 | 7.03E-09 | 2965 |
| Cluster-40555.214296 | 63.77 | 60.75 | 12.59 | 13.77 | 175.87 | 119.26 | 15.54 | 27.04 | -2.09 | 4.47E-06 | -2.72 | 5.92E-08 | 515 |
| Cluster-40555.115611 | 9.41 | 6.64 | 1.83 | 1.57 | 5.96 | 6.44 | 1.95 | 1.9 | -2.09 | 3.30E-04 | -1.63 | 1.22E-02 | 1439 |
| Cluster-40555.229455 | 20.69 | 18.32 | 3.95 | 4.37 | 27.17 | 24.48 | 9.66 | 10.69 | -2.09 | 1.02E-10 | -1.27 | 4.28E-04 | 2381 |
| Cluster-40555.206810 | 20.2 | 18.99 | 4.19 | 4.2 | 14.22 | 16.33 | 3.1 | 2.99 | -2.08 | 2.18E-09 | -2.26 | 1.22E-08 | 1865 |
| Cluster-40555.145951 | 37.66 | 49.83 | 10.01 | 8.94 | 27.03 | 16.45 | 4.28 | 3.82 | -2.08 | 1.15E-04 | -2.35 | 7.06E-04 | 692 |
| Cluster-40555.205845 | 42.37 | 46.68 | 9.34 | 9.92 | 54.21 | 44.74 | 7.97 | 8.92 | -2.07 | 1.67E-09 | -2.48 | 7.24E-12 | 994 |
| Cluster-40555.171189 | 3 | 1.69 | 0.58 | 0.43 | 1.72 | 1.89 | 0.16 | 0 | -2.07 | 2.94E-02 | -4.41 | 2.41E-05 | 2084 |
| Cluster-40555.205319 | 62.71 | 63.91 | 14.2 | 13.22 | 90.03 | 90.83 | 20.78 | 17.99 | -2.07 | 5.32E-13 | -2.16 | 1.48E-11 | 1246 |

| | | | | | | | | | | | | | |
|---|---|---|---|---|---|---|---|---|---|---|---|---|---|
| Cluster-40555.207502 | 8.3 | 5.34 | 1.47 | 1.47 | 9.76 | 6.99 | 1.99 | 2.24 | -2.07 | 6.61E-04 | -1.91 | 2.11E-04 | 1579 |
| Cluster-40555.191938 | 19.08 | 15.94 | 3.78 | 3.8 | 46.27 | 44.18 | 8.13 | 6.37 | -2.06 | 1.21E-10 | -2.58 | 2.24E-16 | 2624 |
| Cluster-40555.215048 | 4.38 | 3.72 | 0.69 | 1.05 | 3.3 | 2.03 | 0.63 | 0.51 | -2.06 | 3.34E-04 | -2.16 | 1.58E-03 | 2823 |
| Cluster-40555.177017 | 8.2 | 5.54 | 1.59 | 1.41 | 3.72 | 3.22 | 1.7 | 1.02 | -2.06 | 7.12E-06 | -1.30 | 4.35E-02 | 2809 |
| Cluster-40555.245601 | 18.05 | 22.27 | 4.75 | 4.08 | 15.5 | 14.11 | 4.08 | 3.08 | -2.06 | 9.34E-07 | -1.99 | 3.90E-05 | 1156 |
| Cluster-40555.184068 | 25.34 | 35.96 | 6.06 | 7.4 | 10.69 | 11.81 | 2.46 | 3.72 | -2.06 | 1.96E-03 | -1.79 | 3.51E-04 | 1334 |
| Cluster-40555.198471 | 48.11 | 30.72 | 7.86 | 9.18 | 16.48 | 16.7 | 7.1 | 7.66 | -2.06 | 3.05E-04 | -1.10 | 1.09E-02 | 2004 |
| Cluster-40555.195056 | 5.95 | 8.52 | 1.14 | 2.01 | 7.46 | 7.64 | 2.59 | 2.72 | -2.06 | 5.99E-03 | -1.44 | 3.49E-03 | 2138 |
| Cluster-40555.193833 | 135.2 | 153.95 | 32.41 | 30.99 | 34.32 | 29.83 | 10.58 | 12.1 | -2.05 | 8.96E-14 | -1.43 | 4.22E-04 | 1078 |
| Cluster-40555.187034 | 15.35 | 11.84 | 0.23 | 5.6 | 29.38 | 12.28 | 0 | 0 | -2.05 | 3.25E-02 | 0.00 | 1.30E-07 | 3225 |
| Cluster-40555.212288 | 2.87 | 2.21 | 0.29 | 0.83 | 9.1 | 8.08 | 1.27 | 0.47 | -2.05 | 1.22E-02 | -3.24 | 2.86E-14 | 2715 |
| Cluster-40555.208878 | 23.33 | 24.3 | 4.93 | 5.57 | 14.48 | 14.64 | 2.44 | 2.28 | -2.04 | 2.31E-08 | -2.56 | 6.67E-09 | 1423 |
| Cluster-40555.170099 | 7.36 | 9.79 | 1.35 | 2.44 | 10.34 | 8.11 | 1.46 | 1.64 | -2.04 | 7.91E-03 | -2.50 | 8.87E-05 | 993 |
| Cluster-40555.184432 | 19.5 | 26.63 | 4.93 | 5.31 | 38.38 | 44.7 | 5.75 | 4.48 | -2.04 | 3.61E-04 | -2.97 | 2.48E-18 | 1674 |
| Cluster-40555.225773 | 9.38 | 11.19 | 2.18 | 2.38 | 3.05 | 2.93 | 0.02 | 0.92 | -2.04 | 5.66E-05 | -2.63 | 2.97E-03 | 1469 |
| Cluster-40555.145990 | 3.19 | 2.61 | 0.67 | 0.61 | 2.55 | 2.45 | 0.55 | 0.36 | -2.03 | 7.89E-03 | -2.38 | 1.36E-03 | 2312 |
| Cluster-40555.201180 | 5.78 | 3.67 | 1.16 | 0.93 | 5.65 | 8.33 | 1.35 | 0.98 | -2.03 | 3.63E-04 | -2.53 | 1.08E-03 | 2505 |
| Cluster-40555.169045 | 24.21 | 21.29 | 4.63 | 5.53 | 6.22 | 4.22 | 1.51 | 0.81 | -2.02 | 2.95E-08 | -2.13 | 1.24E-03 | 1507 |
| Cluster-40555.255927 | 12.66 | 6.88 | 1.74 | 2.57 | 22.93 | 20.21 | 3.86 | 3.69 | -2.02 | 3.75E-02 | -2.45 | 8.03E-08 | 971 |
| Cluster-40555.201561 | 3.46 | 4.14 | 1.09 | 0.64 | 3.82 | 4.11 | 0.29 | 1.22 | -2.02 | 2.72E-05 | -2.31 | 1.08E-06 | 3560 |
| Cluster-40555.195417 | 24.78 | 25.94 | 5.75 | 5.65 | 13.68 | 10.99 | 3.57 | 3.37 | -2.01 | 1.78E-06 | -1.76 | 2.08E-03 | 990 |
| Cluster-40555.169310 | 17.46 | 22.1 | 4.63 | 4.37 | 4.69 | 6.22 | 0.66 | 1.65 | -2.01 | 6.72E-05 | -2.18 | 2.53E-02 | 875 |
| Cluster-40555.154988 | 6.86 | 7.45 | 1.61 | 1.62 | 3.97 | 4.64 | 1.02 | 2.01 | -2.01 | 1.29E-04 | -1.44 | 3.75E-02 | 1862 |
| Cluster-40555.110164 | 16.87 | 14.54 | 4.18 | 2.94 | 38.42 | 29.96 | 4.06 | 3.61 | -2.01 | 1.38E-03 | -3.10 | 5.36E-12 | 764 |
| Cluster-40555.202689 | 119.43 | 163.02 | 35.39 | 28.88 | 48.99 | 49.1 | 22.08 | 15.41 | -2.01 | 1.26E-03 | -1.33 | 1.76E-02 | 503 |
| Cluster-40555.197956 | 73.96 | 102.52 | 19.41 | 20.76 | 4.41 | 5.37 | 1.38 | 0 | -2.00 | 8.87E-04 | -2.79 | 4.03E-04 | 1240 |
| Cluster-40555.222022 | 2.99 | 2.27 | 0.64 | 0.56 | 2.57 | 2.17 | 0.19 | 0.58 | -2.00 | 3.92E-03 | -2.52 | 1.13E-04 | 2902 |
| Cluster-40555.205603 | 43.57 | 43.4 | 9.73 | 10.04 | 12.2 | 11.99 | 1.04 | 1.28 | -2.00 | 6.14E-09 | -3.35 | 5.92E-09 | 1011 |
| Cluster-40555.167335 | 37.1 | 51.53 | 9.81 | 10.46 | 34.3 | 42.8 | 11.98 | 14.76 | -2.00 | 8.98E-04 | -1.46 | 4.73E-04 | 1518 |
| Cluster-40555.180874 | 19.85 | 21.6 | 7.57 | 2.01 | 20.49 | 14.52 | 2.5 | 1.56 | -1.99 | 3.07E-06 | -3.04 | 4.64E-11 | 1154 |
| Cluster-40555.116759 | 30.1 | 31.12 | 6.69 | 7.32 | 110.21 | 98.38 | 11.63 | 12.93 | -1.99 | 1.02E-06 | -3.02 | 6.62E-20 | 934 |
| Cluster-40555.197437 | 172.42 | 272.96 | 47.56 | 55.21 | 253.74 | 338.73 | 33.84 | 58.23 | -1.99 | 3.43E-02 | -2.62 | 7.03E-06 | 672 |

| | | | | | | | | | | | | | |
|---|---|---|---|---|---|---|---|---|---|---|---|---|---|
| Cluster-40555.235035 | 2.32 | 1.73 | 0.48 | 0.46 | 2.44 | 1.98 | 0.47 | 0 | -1.98 | 1.13E-02 | -3.19 | 2.71E-06 | 3132 |
| Cluster-40555.189918 | 1398.03 | 1094.35 | 268.92 | 300.3 | 804.25 | 721.67 | 222.87 | 167.03 | -1.98 | 1.48E-18 | -1.91 | 9.93E-11 | 1222 |
| Cluster-40555.127361 | 21.55 | 26.1 | 4.8 | 6.28 | 44.32 | 28.39 | 4.6 | 0.89 | -1.98 | 8.99E-06 | -3.69 | 6.25E-08 | 973 |
| Cluster-40555.203327 | 79.59 | 76.42 | 17.27 | 18.62 | 31.28 | 27.52 | 14.01 | 12.61 | -1.98 | 2.25E-10 | -1.08 | 3.68E-02 | 852 |
| Cluster-40555.306239 | 3.94 | 3.28 | 0.87 | 0.81 | 1.59 | 0.8 | 0.25 | 0 | -1.97 | 1.95E-02 | -3.23 | 4.08E-02 | 1695 |
| Cluster-40555.210185 | 13.12 | 10.75 | 3.04 | 2.48 | 7.66 | 7.42 | 3.46 | 1.88 | -1.97 | 2.51E-09 | -1.44 | 8.52E-04 | 3212 |
| Cluster-40555.189074 | 3.26 | 1.8 | 0.44 | 0.71 | 3.6 | 3.82 | 0.4 | 0.68 | -1.97 | 4.61E-02 | -2.70 | 2.09E-05 | 2094 |
| Cluster-40555.195597 | 32.48 | 30.02 | 6.86 | 7.66 | 18.96 | 35.75 | 2.59 | 4.51 | -1.97 | 2.16E-09 | -2.88 | 4.89E-02 | 1497 |
| Cluster-40555.119981 | 5.84 | 4.02 | 1.64 | 0.66 | 3.83 | 4.79 | 0.98 | 0.55 | -1.96 | 1.03E-02 | -2.47 | 1.64E-03 | 1352 |
| Cluster-40555.194427 | 8.93 | 9.84 | 1.87 | 2.49 | 32.87 | 23.09 | 2.08 | 4.29 | -1.96 | 2.28E-04 | -3.05 | 6.58E-11 | 1508 |
| Cluster-40555.210052 | 21.46 | 14.74 | 3.4 | 4.88 | 18.6 | 12.6 | 3.75 | 3.87 | -1.96 | 8.81E-04 | -1.95 | 6.95E-04 | 815 |
| Cluster-40555.190431 | 441.59 | 480.67 | 96.83 | 118.59 | 290.55 | 287.85 | 83.99 | 118.33 | -1.96 | 5.91E-17 | -1.44 | 3.47E-06 | 1101 |
| Cluster-40555.215628 | 6.73 | 9.13 | 2.24 | 1.48 | 7.06 | 3.55 | 0 | 0 | -1.96 | 4.75E-04 | 0.00 | 3.98E-07 | 1461 |
| Cluster-40555.192489 | 43.76 | 31.92 | 10.34 | 7.42 | 27.14 | 24.56 | 4.31 | 3.81 | -1.95 | 6.49E-09 | -2.60 | 4.22E-15 | 2694 |
| Cluster-40555.194971 | 11.88 | 13.59 | 4.34 | 1.72 | 22.44 | 23.88 | 3.65 | 2.89 | -1.94 | 2.79E-04 | -2.76 | 4.35E-10 | 1013 |
| Cluster-40555.144568 | 27.34 | 27.97 | 8.35 | 4.84 | 37.97 | 35.3 | 8.17 | 9.56 | -1.94 | 4.05E-05 | -1.98 | 6.40E-06 | 732 |
| Cluster-40555.235206 | 2.21 | 2.47 | 0.49 | 0.61 | 3.92 | 3.27 | 0.6 | 0.91 | -1.94 | 4.93E-02 | -2.17 | 7.35E-04 | 2095 |
| Cluster-40555.184710 | 12.8 | 11.91 | 3.18 | 2.7 | 5.28 | 5.61 | 1.93 | 0.84 | -1.93 | 5.23E-09 | -1.93 | 3.17E-05 | 3095 |
| Cluster-40555.168002 | 24.4 | 26.03 | 5.78 | 6.24 | 6.32 | 7.22 | 1.89 | 2.36 | -1.93 | 1.18E-08 | -1.60 | 5.72E-03 | 1618 |
| Cluster-40555.219781 | 1.67 | 1.61 | 0.42 | 0.35 | 1.78 | 1.13 | 0.21 | 0.15 | -1.93 | 6.50E-03 | -2.92 | 2.31E-05 | 4283 |
| Cluster-40555.202740 | 56.63 | 53.22 | 14.01 | 12.38 | 44.25 | 42.33 | 18.54 | 15.9 | -1.92 | 1.40E-11 | -1.27 | 6.40E-04 | 1419 |
| Cluster-40555.145249 | 13.57 | 13.34 | 4.14 | 2.45 | 12.88 | 13.14 | 6.33 | 3.01 | -1.90 | 5.10E-04 | -1.43 | 1.85E-02 | 991 |
| Cluster-40555.190141 | 76.79 | 60.52 | 16.34 | 16.97 | 58.98 | 50.13 | 24.94 | 24.45 | -1.90 | 2.67E-11 | -1.08 | 5.03E-03 | 1253 |
| Cluster-40555.190678 | 125.48 | 123.31 | 32.33 | 28.52 | 36.28 | 32.16 | 11.95 | 8.89 | -1.89 | 4.74E-16 | -1.65 | 7.05E-07 | 2469 |
| Cluster-40555.194916 | 53.95 | 76.26 | 15.07 | 16.9 | 13.99 | 14.87 | 6.7 | 2.06 | -1.89 | 4.09E-03 | -1.68 | 9.10E-03 | 797 |
| Cluster-40555.207617 | 24.91 | 26.02 | 5.98 | 6.52 | 17.28 | 13.46 | 5.08 | 4.79 | -1.89 | 1.61E-08 | -1.57 | 1.90E-04 | 1664 |
| Cluster-40555.203508 | 6.52 | 5.43 | 1.77 | 1.16 | 8.1 | 6.3 | 2.21 | 2.85 | -1.89 | 2.72E-05 | -1.43 | 1.44E-03 | 2756 |
| Cluster-40555.187095 | 1174.49 | 937.14 | 248.57 | 266.18 | 91.23 | 91.86 | 39.94 | 28.36 | -1.88 | 6.05E-16 | -1.37 | 5.50E-04 | 629 |
| Cluster-40555.149087 | 6.39 | 4.55 | 1.47 | 1.22 | 8.42 | 8.81 | 2.03 | 1.89 | -1.88 | 1.94E-03 | -2.08 | 1.18E-05 | 1855 |
| Cluster-40555.184781 | 15.18 | 13.16 | 3.28 | 3.69 | 23.02 | 22.69 | 1.67 | 2.2 | -1.88 | 2.63E-05 | -3.50 | 2.97E-18 | 1444 |
| Cluster-40555.176982 | 15.39 | 16.34 | 3.81 | 4.07 | 12.17 | 9.3 | 4.53 | 3.1 | -1.88 | 4.20E-04 | -1.43 | 3.19E-02 | 976 |
| Cluster-40555.188790 | 82.26 | 66.44 | 14.65 | 21.86 | 65.27 | 63.52 | 19.53 | 24.06 | -1.87 | 2.81E-12 | -1.49 | 5.26E-06 | 1755 |

| | | | | | | | | | | | | | |
|---|---|---|---|---|---|---|---|---|---|---|---|---|---|
| Cluster-40555.228496 | 4.29 | 4.18 | 0.45 | 1.6 | 3.06 | 2.8 | 0.88 | 0.52 | -1.87 | 1.84E-02 | -2.01 | 1.26E-02 | 1862 |
| Cluster-40555.190483 | 3.31 | 4.12 | 1.07 | 0.8 | 3.73 | 3.52 | 0.67 | 0.35 | -1.86 | 1.05E-02 | -2.79 | 4.46E-05 | 1918 |
| Cluster-40555.165498 | 188.15 | 175.47 | 37.94 | 51.1 | 156.04 | 129.11 | 58.43 | 59.7 | -1.86 | 1.89E-05 | -1.23 | 2.21E-02 | 361 |
| Cluster-40555.196064 | 12.72 | 12.23 | 3.64 | 2.63 | 12.29 | 10 | 4.66 | 4.07 | -1.86 | 1.87E-07 | -1.28 | 2.81E-03 | 2402 |
| Cluster-40555.210618 | 51.42 | 62.17 | 13.21 | 15.28 | 8.7 | 5.7 | 1.79 | 3 | -1.86 | 3.87E-07 | -1.50 | 1.61E-02 | 1383 |
| Cluster-40555.170283 | 2.32 | 3.03 | 0.57 | 0.78 | 2.49 | 1.85 | 0.25 | 0.62 | -1.86 | 2.51E-02 | -2.24 | 4.22E-03 | 2344 |
| Cluster-40555.95393 | 4.64 | 5.28 | 1.77 | 0.75 | 14.26 | 12.12 | 1.23 | 1.15 | -1.85 | 3.12E-02 | -3.40 | 4.92E-11 | 1165 |
| Cluster-40555.197572 | 20.56 | 22.79 | 5.28 | 5.63 | 4.19 | 4.36 | 0.96 | 0.92 | -1.85 | 6.65E-05 | -2.14 | 4.83E-02 | 952 |
| Cluster-40555.180213 | 35.1 | 33.91 | 8.77 | 8.61 | 8.67 | 7.61 | 1.61 | 0.78 | -1.85 | 4.05E-10 | -2.70 | 3.17E-08 | 1848 |
| Cluster-40555.170111 | 6.16 | 9.77 | 2.07 | 2.01 | 38.43 | 37.11 | 2.59 | 2.94 | -1.84 | 3.82E-02 | -3.70 | 3.30E-26 | 1785 |
| Cluster-40555.134529 | 2.3 | 1.53 | 0.55 | 0.42 | 1.38 | 1.7 | 0.28 | 0.49 | -1.84 | 1.77E-02 | -1.93 | 1.50E-02 | 3333 |
| Cluster-40555.151243 | 27.29 | 23.76 | 5.59 | 7.28 | 61.59 | 47.04 | 5.04 | 10.11 | -1.84 | 3.29E-05 | -2.75 | 1.79E-14 | 953 |
| Cluster-40555.126278 | 5.07 | 4.18 | 1.76 | 0.6 | 3.73 | 3.72 | 0 | 1.42 | -1.83 | 3.11E-02 | -2.28 | 1.03E-02 | 1241 |
| Cluster-40555.184051 | 9.2 | 5.83 | 2.03 | 1.78 | 10.94 | 11.24 | 2.18 | 1.91 | -1.83 | 1.19E-03 | -2.38 | 9.46E-08 | 1746 |
| Cluster-40555.174424 | 7.58 | 5.82 | 2.37 | 1.08 | 14.56 | 13.16 | 2.56 | 3.16 | -1.83 | 1.61E-03 | -2.20 | 3.35E-07 | 1547 |
| Cluster-40555.191029 | 30.38 | 23.97 | 6.04 | 7.76 | 11.31 | 9.03 | 4.05 | 2.91 | -1.83 | 2.25E-09 | -1.48 | 6.91E-04 | 2230 |
| Cluster-40555.170051 | 8.83 | 10.56 | 2.52 | 2.47 | 8.18 | 11.85 | 3.78 | 2.34 | -1.83 | 1.32E-05 | -1.67 | 3.33E-02 | 2168 |
| Cluster-40555.250160 | 23.33 | 15.13 | 4.92 | 4.84 | 25.83 | 23.96 | 10.03 | 13.02 | -1.83 | 3.30E-04 | -1.04 | 4.00E-02 | 1000 |
| Cluster-40555.158740 | 13.11 | 12.3 | 2.43 | 4.07 | 4.27 | 3.25 | 1.44 | 0.4 | -1.82 | 1.81E-04 | -1.96 | 2.14E-02 | 1435 |
| Cluster-40555.183211 | 13.44 | 11.73 | 2.73 | 3.7 | 20.12 | 15.6 | 4.11 | 4.7 | -1.82 | 4.46E-05 | -1.95 | 7.35E-07 | 1656 |
| Cluster-40555.185383 | 2.85 | 1.74 | 0.61 | 0.57 | 2.91 | 3.15 | 0.25 | 0 | -1.81 | 4.09E-02 | -4.56 | 3.24E-10 | 2454 |
| Cluster-40555.188015 | 46.25 | 35.89 | 11.26 | 10.03 | 17.96 | 16.92 | 6.72 | 4.34 | -1.81 | 7.32E-11 | -1.60 | 3.76E-05 | 2032 |
| Cluster-40555.226307 | 8.04 | 6.44 | 1.86 | 1.9 | 13.97 | 16.05 | 2.17 | 2.44 | -1.80 | 9.80E-05 | -2.64 | 1.54E-12 | 2382 |
| Cluster-40555.184254 | 538.91 | 591.66 | 139.01 | 156.05 | 742.34 | 730.92 | 197.76 | 193.69 | -1.80 | 1.06E-14 | -1.85 | 4.63E-10 | 960 |
| Cluster-40555.200455 | 89.46 | 92.76 | 20.63 | 26.88 | 17.63 | 14.84 | 7.94 | 6.56 | -1.80 | 1.32E-10 | -1.10 | 4.61E-02 | 1131 |
| Cluster-40555.199937 | 27.89 | 21.25 | 7.22 | 5.61 | 11.92 | 9.12 | 4.22 | 3.03 | -1.79 | 6.13E-07 | -1.47 | 6.16E-03 | 1359 |
| Cluster-40555.177318 | 15.62 | 14.3 | 5.37 | 2.54 | 34.87 | 24.48 | 12.12 | 7.62 | -1.79 | 4.82E-02 | -1.53 | 1.30E-02 | 552 |
| Cluster-40555.222685 | 138.12 | 130.3 | 36.35 | 33.7 | 445.21 | 390.19 | 40.26 | 49.61 | -1.79 | 1.44E-07 | -3.16 | 5.05E-23 | 504 |
| Cluster-40555.192007 | 285.25 | 246.72 | 75.56 | 63.88 | 123.15 | 132.46 | 40.19 | 44.6 | -1.79 | 5.17E-13 | -1.53 | 6.89E-06 | 797 |
| Cluster-40555.217028 | 27.07 | 17.63 | 5.46 | 6.04 | 40.34 | 50.91 | 8.08 | 11.22 | -1.79 | 6.36E-03 | -2.18 | 2.97E-06 | 628 |
| Cluster-40555.227150 | 7.93 | 5.99 | 1.55 | 2.11 | 15.85 | 14.57 | 3.81 | 2.54 | -1.79 | 1.52E-02 | -2.20 | 1.52E-06 | 1263 |
| Cluster-40555.133137 | 12.42 | 10.19 | 3.42 | 2.53 | 28.35 | 26.11 | 2.96 | 3.88 | -1.78 | 9.79E-03 | -2.92 | 7.73E-11 | 873 |

| | | | | | | | | | | | | | |
|---|---|---|---|---|---|---|---|---|---|---|---|---|---|
| Cluster-40555.190949 | 125.96 | 84.08 | 26.57 | 28.49 | 53.68 | 47.61 | 24.97 | 27.23 | -1.78 | 1.38E-04 | -0.89 | 2.74E-02 | 1709 |
| Cluster-40555.229374 | 16.09 | 15.01 | 3.53 | 4.64 | 40.74 | 38.44 | 6.99 | 6.18 | -1.78 | 1.30E-07 | -2.52 | 1.76E-15 | 2646 |
| Cluster-40555.242563 | 3.5 | 3.22 | 1.03 | 0.76 | 4.26 | 3.29 | 1.63 | 1.67 | -1.77 | 4.90E-04 | -1.12 | 4.45E-02 | 3766 |
| Cluster-40555.198124 | 19.65 | 17.57 | 4.51 | 5.37 | 6.53 | 4.93 | 0.55 | 0.4 | -1.77 | 3.88E-04 | -3.46 | 5.01E-05 | 986 |
| Cluster-40555.193409 | 29.99 | 29.01 | 7.89 | 7.83 | 10.89 | 9.62 | 3.76 | 5.21 | -1.77 | 2.28E-08 | -1.12 | 3.37E-02 | 1700 |
| Cluster-40555.151481 | 9.06 | 5.66 | 2.46 | 1.49 | 9.31 | 8.38 | 1.31 | 1.7 | -1.76 | 3.80E-03 | -2.49 | 2.74E-06 | 1394 |
| Cluster-40555.179837 | 8.87 | 10.42 | 2.57 | 2.66 | 12.01 | 12.48 | 3.79 | 3.56 | -1.75 | 4.60E-04 | -1.67 | 2.66E-04 | 1583 |
| Cluster-40555.196873 | 9.6 | 10.73 | 2.74 | 2.74 | 9.19 | 8.21 | 3.67 | 2.38 | -1.75 | 4.68E-03 | -1.46 | 3.26E-02 | 1105 |
| Cluster-40555.223927 | 43.07 | 37.96 | 11.38 | 10.35 | 64.44 | 51.11 | 24.74 | 22.76 | -1.75 | 8.88E-04 | -1.22 | 1.44E-02 | 547 |
| Cluster-40555.196020 | 7.66 | 8.04 | 1.09 | 3.11 | 5.32 | 6.38 | 0.23 | 0.58 | -1.74 | 1.53E-02 | -3.78 | 1.19E-07 | 1279 |
| Cluster-40555.209686 | 32.12 | 29.97 | 8.55 | 8.32 | 21.54 | 15.79 | 8.17 | 9.55 | -1.74 | 1.24E-08 | -1.00 | 2.42E-02 | 1808 |
| Cluster-40555.196476 | 37.27 | 29.39 | 9.12 | 8.97 | 12.9 | 12.12 | 4.96 | 5.36 | -1.74 | 2.16E-11 | -1.21 | 1.65E-03 | 3620 |
| Cluster-40555.212578 | 14.43 | 13.14 | 4.29 | 3.27 | 10.85 | 6.45 | 1.56 | 2.93 | -1.73 | 3.58E-08 | -1.86 | 2.16E-02 | 3169 |
| Cluster-40555.223228 | 49.58 | 59.14 | 16.05 | 13.96 | 372.98 | 356.83 | 36.31 | 44.81 | -1.73 | 1.18E-07 | -3.10 | 1.06E-25 | 1323 |
| Cluster-40555.174001 | 18.3 | 14.18 | 4.81 | 4.14 | 41.83 | 31.01 | 9.89 | 10.59 | -1.72 | 9.89E-06 | -1.76 | 3.53E-07 | 1637 |
| Cluster-40555.202492 | 66.63 | 89.96 | 21.8 | 21.58 | 31.81 | 37.71 | 10.82 | 9.06 | -1.72 | 1.48E-03 | -1.75 | 1.26E-05 | 1001 |
| Cluster-40555.157572 | 8.63 | 8.55 | 1.95 | 2.77 | 21.77 | 23.73 | 4.04 | 2.79 | -1.71 | 1.76E-02 | -2.68 | 3.98E-10 | 1104 |
| Cluster-40555.218027 | 10.69 | 8.99 | 3.01 | 2.46 | 10.82 | 10 | 4.96 | 3.91 | -1.71 | 1.19E-05 | -1.17 | 9.88E-03 | 2477 |
| Cluster-40555.178130 | 73.85 | 87.28 | 24.72 | 20.3 | 182.78 | 142.44 | 36.69 | 37.35 | -1.70 | 5.35E-07 | -2.07 | 4.10E-10 | 654 |
| Cluster-40555.283333 | 55.18 | 48.79 | 14.11 | 14.97 | 38.34 | 31.13 | 14.02 | 11.4 | -1.70 | 1.49E-06 | -1.39 | 1.68E-03 | 853 |
| Cluster-40555.232693 | 2.41 | 2.09 | 0.72 | 0.54 | 2.17 | 2.74 | 0.82 | 0.84 | -1.69 | 3.60E-02 | -1.52 | 4.16E-02 | 2734 |
| Cluster-40555.201585 | 3.17 | 2.08 | 0.47 | 0.99 | 10.07 | 7.91 | 2.2 | 1.76 | -1.69 | 4.98E-02 | -2.11 | 2.81E-07 | 2611 |
| Cluster-40555.155806 | 13.42 | 13.34 | 4.77 | 2.74 | 29.84 | 23.36 | 3.35 | 3.71 | -1.68 | 2.32E-02 | -2.85 | 1.69E-08 | 707 |
| Cluster-40555.194972 | 10.28 | 13.67 | 3.15 | 3.76 | 6.68 | 6.16 | 2.18 | 1.24 | -1.66 | 1.70E-03 | -1.86 | 3.33E-03 | 1404 |
| Cluster-40555.234771 | 6.87 | 9.28 | 2.8 | 1.9 | 5.33 | 5.26 | 0.74 | 2.04 | -1.65 | 3.30E-02 | -1.86 | 4.83E-02 | 958 |
| Cluster-40555.218307 | 7.19 | 6.21 | 0.84 | 2.99 | 14.13 | 10.38 | 0.68 | 1.66 | -1.65 | 8.97E-03 | -3.31 | 1.01E-15 | 2200 |
| Cluster-40555.238603 | 20.88 | 20.77 | 6.06 | 6.11 | 14.94 | 14.86 | 6.14 | 4.53 | -1.63 | 1.25E-04 | -1.43 | 5.58E-03 | 1148 |
| Cluster-40555.207301 | 27.29 | 33.52 | 8.66 | 9.27 | 13.98 | 15.59 | 7.11 | 6.18 | -1.63 | 1.00E-05 | -1.09 | 1.31E-02 | 2212 |
| Cluster-40555.200164 | 30.97 | 44.2 | 10.35 | 11.87 | 22.25 | 20.07 | 6.26 | 6.84 | -1.62 | 1.37E-02 | -1.62 | 4.32E-05 | 1498 |
| Cluster-40555.211808 | 3.12 | 2.49 | 0.9 | 0.76 | 2.08 | 2.03 | 0.69 | 0.22 | -1.62 | 3.50E-03 | -2.13 | 4.94E-04 | 3992 |
| Cluster-40555.181990 | 6.92 | 4.79 | 2.12 | 1.33 | 3.68 | 3.14 | 1.45 | 1.07 | -1.62 | 4.56E-04 | -1.38 | 3.25E-02 | 2675 |
| Cluster-40555.224146 | 19.07 | 22.11 | 5.53 | 6.64 | 13.15 | 12.04 | 4.34 | 3.59 | -1.62 | 9.22E-06 | -1.60 | 3.93E-04 | 1626 |

| | | | | | | | | | | | | | |
|---|---|---|---|---|---|---|---|---|---|---|---|---|---|
| Cluster-40555.189417 | 10.68 | 9.23 | 2.22 | 3.62 | 7.83 | 7.11 | 2.96 | 3.97 | -1.62 | 3.66E-05 | -1.03 | 3.66E-02 | 2853 |
| Cluster-40555.193825 | 114.89 | 119.73 | 32.44 | 37.01 | 372.94 | 275.97 | 55.13 | 26.1 | -1.62 | 3.11E-09 | -2.94 | 3.79E-15 | 1008 |
| Cluster-40555.195739 | 27.59 | 19.85 | 6.91 | 7.08 | 10.83 | 10.44 | 4.71 | 4.1 | -1.61 | 2.63E-06 | -1.21 | 1.93E-02 | 1681 |
| Cluster-40555.203797 | 9.03 | 9.55 | 2.83 | 2.68 | 6.36 | 5.9 | 1.34 | 1.3 | -1.61 | 3.14E-02 | -2.18 | 9.27E-03 | 957 |
| Cluster-40555.204564 | 16.32 | 25.22 | 6.47 | 5.98 | 13.3 | 12.39 | 6.23 | 6.25 | -1.61 | 4.58E-02 | -0.97 | 3.94E-02 | 2291 |
| Cluster-40555.171632 | 11.73 | 10.15 | 2.4 | 4.07 | 19.95 | 15.66 | 4.39 | 4.86 | -1.61 | 3.17E-03 | -1.87 | 7.29E-06 | 1402 |
| Cluster-40555.208505 | 44.96 | 62.89 | 15.95 | 16.64 | 42.13 | 44.46 | 14.73 | 4.91 | -1.61 | 1.70E-02 | -2.10 | 3.57E-03 | 422 |
| Cluster-40555.196567 | 25.21 | 21.48 | 6.5 | 7.41 | 18.33 | 17.26 | 6.77 | 5.51 | -1.60 | 2.12E-05 | -1.47 | 8.05E-04 | 1383 |
| Cluster-40555.218737 | 4.6 | 2.76 | 1.15 | 1.04 | 4.87 | 4.13 | 1.65 | 1.69 | -1.59 | 4.67E-02 | -1.36 | 4.57E-02 | 1911 |
| Cluster-40555.143181 | 7.94 | 8.48 | 2.41 | 2.52 | 39.95 | 35.21 | 5.85 | 6.07 | -1.59 | 1.10E-02 | -2.59 | 3.57E-13 | 1331 |
| Cluster-40555.188248 | 265.22 | 260.42 | 80.56 | 78.11 | 83.58 | 73.68 | 39.82 | 39.89 | -1.59 | 5.19E-11 | -0.91 | 2.51E-02 | 1031 |
| Cluster-40555.177564 | 3.38 | 3.41 | 1.21 | 0.85 | 6.39 | 4.97 | 0.76 | 1.19 | -1.58 | 4.07E-02 | -2.48 | 3.12E-06 | 2010 |
| Cluster-40555.179016 | 5.78 | 5.87 | 1.75 | 1.8 | 5.91 | 5.93 | 2.25 | 1.85 | -1.58 | 1.66E-04 | -1.47 | 9.07E-04 | 3559 |
| Cluster-40555.171449 | 15.53 | 13.63 | 3.35 | 5.44 | 16.74 | 14.41 | 3.23 | 4.88 | -1.58 | 6.73E-04 | -1.86 | 1.31E-05 | 1449 |
| Cluster-40555.141308 | 13.3 | 14.7 | 3.99 | 4.52 | 9.35 | 10.24 | 2.11 | 2.22 | -1.58 | 9.90E-03 | -2.11 | 1.33E-03 | 927 |
| Cluster-40555.234875 | 10.11 | 7.76 | 2.77 | 2.7 | 9.28 | 8.49 | 3.61 | 2.65 | -1.57 | 1.19E-02 | -1.44 | 1.86E-02 | 1265 |
| Cluster-40555.217971 | 57.14 | 71.47 | 21.4 | 18.65 | 34.08 | 43.63 | 12.99 | 14.91 | -1.55 | 4.40E-04 | -1.42 | 1.21E-02 | 540 |
| Cluster-40555.152588 | 11.14 | 8.45 | 3.32 | 2.76 | 17.04 | 21.01 | 2.25 | 3.58 | -1.55 | 2.21E-02 | -2.62 | 1.29E-08 | 1041 |
| Cluster-40555.140000 | 7.64 | 5.04 | 2.11 | 1.81 | 5.04 | 4.44 | 1.7 | 1.79 | -1.54 | 1.37E-03 | -1.38 | 9.11E-03 | 2852 |
| Cluster-40555.183704 | 33.39 | 27.26 | 8.93 | 9.91 | 6.57 | 5.28 | 1.87 | 2.13 | -1.54 | 1.68E-05 | -1.51 | 4.23E-02 | 1273 |
| Cluster-40555.225222 | 16.34 | 10.95 | 3.74 | 4.7 | 23.94 | 21.16 | 9.66 | 9.4 | -1.54 | 1.69E-03 | -1.17 | 2.26E-03 | 2222 |
| Cluster-40555.181060 | 86.56 | 100.44 | 24.54 | 33.86 | 75.93 | 67.33 | 35.65 | 37.65 | -1.54 | 8.23E-06 | -0.90 | 3.13E-02 | 1023 |
| Cluster-40555.219087 | 113.45 | 101.8 | 31.38 | 35.78 | 121.27 | 115.27 | 27.44 | 28.76 | -1.54 | 1.71E-09 | -2.01 | 5.40E-11 | 1626 |
| Cluster-40555.206277 | 19.24 | 11.5 | 4.01 | 5.53 | 16.86 | 18.46 | 6.31 | 6.66 | -1.53 | 4.19E-02 | -1.38 | 4.01E-03 | 1181 |
| Cluster-40555.190232 | 23.73 | 25.3 | 7.51 | 7.96 | 14.93 | 17.03 | 4.68 | 6.77 | -1.52 | 3.54E-07 | -1.41 | 2.20E-04 | 2475 |
| Cluster-40555.178291 | 9.13 | 5.79 | 2.81 | 1.92 | 14.86 | 15.37 | 0.36 | 0.3 | -1.52 | 2.14E-02 | -5.50 | 1.54E-23 | 1298 |
| Cluster-40555.169628 | 13.38 | 9.45 | 3.26 | 3.91 | 14.27 | 9.8 | 2.72 | 4.68 | -1.52 | 2.84E-02 | -1.62 | 5.80E-03 | 970 |
| Cluster-40555.218787 | 42.36 | 44.6 | 14.45 | 13.2 | 311.39 | 240.73 | 10.05 | 11.79 | -1.52 | 4.07E-05 | -4.59 | 6.98E-45 | 854 |
| Cluster-40555.181985 | 6.28 | 5.51 | 2.11 | 1.63 | 5.58 | 5.64 | 1.72 | 1.73 | -1.52 | 2.01E-03 | -1.63 | 1.22E-03 | 2488 |
| Cluster-40555.196869 | 71.8 | 68.47 | 20.84 | 22.91 | 89.09 | 105.47 | 41.75 | 27.78 | -1.52 | 4.65E-02 | -1.45 | 2.16E-02 | 356 |
| Cluster-40555.198630 | 10.19 | 6.15 | 3.09 | 2.07 | 7.39 | 6.33 | 2.32 | 3.37 | -1.52 | 2.26E-02 | -1.20 | 4.35E-02 | 1787 |
| Cluster-40555.175399 | 2.56 | 2.32 | 0.98 | 0.59 | 2.32 | 2 | 0.52 | 0.48 | -1.51 | 2.64E-03 | -2.05 | 9.54E-05 | 5091 |

| | | | | | | | | | | | | | |
|---|---|---|---|---|---|---|---|---|---|---|---|---|---|
| Cluster-40555.188584 | 1631.7 | 1614.18 | 529.72 | 503.35 | 876.7 | 720.77 | 367.12 | 350.84 | -1.51 | 2.97E-11 | -1.09 | 1.11E-03 | 658 |
| Cluster-40555.184521 | 10.18 | 10.41 | 4.82 | 1.84 | 10.81 | 11.88 | 2.09 | 4.6 | -1.50 | 9.97E-05 | -1.68 | 4.02E-05 | 3507 |
| Cluster-40555.168217 | 7.72 | 7.99 | 2.26 | 2.77 | 10.32 | 9.91 | 5.17 | 2.41 | -1.50 | 5.50E-03 | -1.36 | 6.04E-03 | 1785 |
| Cluster-40555.162965 | 49.85 | 54.74 | 17.37 | 16.23 | 12.22 | 10.15 | 2.32 | 2.7 | -1.50 | 6.11E-06 | -2.08 | 6.04E-04 | 939 |
| Cluster-40555.208506 | 86.01 | 79.24 | 25.53 | 27.25 | 56.93 | 52.7 | 16.57 | 10.19 | -1.50 | 3.16E-05 | -1.98 | 3.86E-06 | 618 |
| Cluster-40555.218365 | 33.2 | 37.66 | 12.04 | 10.8 | 30.47 | 30.34 | 13.04 | 11.78 | -1.50 | 2.34E-04 | -1.23 | 1.20E-02 | 824 |
| Cluster-40555.156059 | 22.5 | 26.68 | 6.9 | 8.95 | 14.27 | 12.37 | 4.79 | 4.46 | -1.49 | 1.99E-05 | -1.46 | 6.29E-04 | 1913 |
| Cluster-40555.197670 | 66.98 | 57.13 | 19.99 | 20.08 | 51.32 | 48.15 | 15.73 | 17.85 | -1.49 | 8.50E-08 | -1.50 | 1.31E-05 | 1523 |
| Cluster-40555.182111 | 39.12 | 36.55 | 12.46 | 12.09 | 21.91 | 17.03 | 10.11 | 10.05 | -1.48 | 4.48E-08 | -0.88 | 4.45E-02 | 2537 |
| Cluster-40555.49948 | 7.13 | 7.9 | 3 | 1.92 | 15.54 | 13.09 | 3.29 | 2.05 | -1.47 | 4.62E-02 | -2.36 | 2.26E-06 | 1102 |
| Cluster-40555.224225 | 28.59 | 24.92 | 8.64 | 8.87 | 23.9 | 19.48 | 9.84 | 9.93 | -1.47 | 7.45E-06 | -1.06 | 1.25E-02 | 1734 |
| Cluster-40555.235279 | 18.09 | 15.66 | 7.91 | 3.26 | 12.31 | 10.86 | 4.53 | 4.02 | -1.47 | 4.52E-04 | -1.37 | 1.48E-02 | 1198 |
| Cluster-40555.174000 | 9.86 | 6.88 | 2.16 | 3.3 | 18.85 | 17.09 | 6.42 | 7.65 | -1.46 | 1.10E-02 | -1.28 | 2.30E-03 | 1669 |
| Cluster-40555.209948 | 31.15 | 32.14 | 11.33 | 9.86 | 35.56 | 43.05 | 18.52 | 13.18 | -1.44 | 6.70E-08 | -1.26 | 1.09E-03 | 3004 |
| Cluster-40555.166833 | 78.22 | 74.22 | 23.73 | 27.15 | 34.67 | 34.43 | 15.67 | 14.41 | -1.44 | 7.49E-06 | -1.14 | 1.75E-02 | 842 |
| Cluster-40555.203016 | 30.51 | 26.87 | 9.21 | 9.99 | 69.42 | 61.33 | 32.7 | 35.23 | -1.44 | 4.60E-05 | -0.87 | 3.06E-02 | 1386 |
| Cluster-40555.188409 | 36.27 | 27.98 | 14.09 | 7.55 | 44.87 | 41.31 | 22.08 | 25.01 | -1.43 | 2.58E-05 | -0.80 | 4.65E-02 | 3400 |
| Cluster-40555.199409 | 10.93 | 11.94 | 3.33 | 4.35 | 13.19 | 13.34 | 4.21 | 5.31 | -1.43 | 7.82E-04 | -1.41 | 7.79E-04 | 2034 |
| Cluster-40555.179176 | 59.67 | 73.01 | 24.73 | 20.21 | 94.35 | 66.64 | 19.28 | 12.37 | -1.43 | 9.08E-05 | -2.28 | 5.48E-08 | 923 |
| Cluster-40555.205786 | 20.82 | 24.93 | 6.54 | 9.03 | 57.14 | 50.24 | 3.91 | 3.21 | -1.41 | 2.90E-03 | -3.84 | 4.93E-24 | 972 |
| Cluster-40555.181440 | 25.28 | 26.6 | 8.5 | 9.13 | 21.11 | 19.11 | 7.56 | 8.54 | -1.41 | 4.06E-04 | -1.25 | 8.77E-03 | 1130 |
| Cluster-40555.86210 | 8.16 | 5.4 | 2.18 | 2.38 | 9.7 | 7.62 | 3.83 | 2.63 | -1.41 | 1.18E-02 | -1.36 | 6.19E-03 | 1977 |
| Cluster-40555.202355 | 28.74 | 33.56 | 9.2 | 12.01 | 132.22 | 106.62 | 39.48 | 27.13 | -1.41 | 1.38E-04 | -1.78 | 2.22E-08 | 1196 |
| Cluster-40555.190257 | 153.25 | 161.71 | 49.74 | 58.12 | 134.75 | 101.13 | 27.04 | 25.37 | -1.41 | 1.02E-07 | -2.10 | 4.47E-11 | 1010 |
| Cluster-40555.202354 | 120.89 | 135.8 | 42.9 | 46.12 | 319 | 347.98 | 48.78 | 49.44 | -1.39 | 7.65E-06 | -2.70 | 3.34E-18 | 640 |
| Cluster-40555.157583 | 5.15 | 3.95 | 2.25 | 0.93 | 5.87 | 4.54 | 1.17 | 2.19 | -1.38 | 4.17E-02 | -1.55 | 1.18E-02 | 1777 |
| Cluster-40555.195646 | 30.68 | 39.05 | 12.89 | 11.54 | 24.6 | 24.38 | 5.96 | 5.88 | -1.38 | 1.14E-02 | -1.99 | 1.11E-03 | 581 |
| Cluster-40555.178975 | 41.88 | 58.57 | 17.24 | 18.18 | 11.33 | 12.76 | 2.08 | 0.8 | -1.37 | 3.08E-02 | -3.04 | 1.51E-05 | 752 |
| Cluster-40555.212583 | 12.62 | 10.39 | 3.79 | 4.3 | 46.84 | 42.45 | 8.28 | 11.46 | -1.36 | 4.29E-04 | -2.10 | 2.12E-11 | 2513 |
| Cluster-40555.206482 | 7.9 | 6.72 | 3.36 | 1.84 | 9.35 | 7.47 | 3.75 | 3.44 | -1.36 | 4.50E-03 | -1.16 | 2.68E-02 | 2033 |
| Cluster-40555.203558 | 81.67 | 82.6 | 30.37 | 27.89 | 64.58 | 56.93 | 36.31 | 29.94 | -1.36 | 3.28E-08 | -0.81 | 4.65E-02 | 2417 |
| Cluster-40555.165315 | 55.55 | 58.94 | 21.26 | 19.58 | 26.08 | 28.2 | 12.05 | 9.51 | -1.35 | 2.45E-06 | -1.27 | 1.96E-03 | 1375 |

| | | | | | | | | | | | | | |
|---|---|---|---|---|---|---|---|---|---|---|---|---|---|
| Cluster-40555.191828 | 2.37 | 2.59 | 0.94 | 0.83 | 2.23 | 1.51 | 0.68 | 0.69 | -1.35 | 8.48E-03 | -1.37 | 1.90E-02 | 5406 |
| Cluster-40555.159263 | 5.56 | 5.61 | 2.42 | 1.6 | 3.45 | 4.38 | 1.36 | 0.66 | -1.34 | 2.07E-02 | -1.91 | 4.27E-03 | 1971 |
| Cluster-40555.201415 | 20.11 | 15.31 | 7.51 | 5.18 | 13.4 | 13.36 | 0.76 | 2.28 | -1.34 | 5.81E-04 | -3.04 | 1.33E-11 | 1488 |
| Cluster-40555.203392 | 5.33 | 4.89 | 1.57 | 2.06 | 7.32 | 8.92 | 2.18 | 2.79 | -1.34 | 2.81E-02 | -1.64 | 2.91E-04 | 2338 |
| Cluster-40555.155974 | 18.79 | 21.99 | 6.15 | 8.49 | 29.89 | 20.17 | 10.9 | 10.62 | -1.33 | 3.54E-03 | -1.14 | 1.14E-02 | 1149 |
| Cluster-40555.170347 | 9.89 | 8.93 | 2.84 | 3.92 | 9.18 | 8.44 | 3.95 | 2.8 | -1.33 | 2.48E-03 | -1.32 | 4.11E-03 | 2417 |
| Cluster-40555.206503 | 6.7 | 6.1 | 2.82 | 1.79 | 5.92 | 6.25 | 1.43 | 2.45 | -1.33 | 2.16E-02 | -1.57 | 6.46E-03 | 1764 |
| Cluster-40555.205161 | 13.32 | 13.79 | 4.93 | 4.91 | 20.71 | 18.47 | 5.63 | 5.65 | -1.32 | 2.41E-02 | -1.73 | 1.97E-04 | 1037 |
| Cluster-40555.190095 | 36.14 | 29.04 | 11.72 | 11.97 | 20.45 | 16.5 | 6.78 | 7.17 | -1.32 | 1.47E-04 | -1.33 | 2.94E-03 | 1326 |
| Cluster-40555.162527 | 4.66 | 3.68 | 1.38 | 1.65 | 6.18 | 6.11 | 1.69 | 1.94 | -1.32 | 4.63E-02 | -1.68 | 4.59E-04 | 2514 |
| Cluster-40555.185655 | 692.79 | 624.18 | 217.2 | 261.72 | 311.43 | 316.67 | 164.96 | 158.1 | -1.31 | 1.06E-07 | -0.90 | 1.89E-02 | 691 |
| Cluster-40555.204350 | 4.87 | 2.99 | 1.41 | 1.44 | 1.82 | 1.96 | 0.31 | 0.57 | -1.30 | 4.30E-02 | -2.03 | 4.04E-03 | 3222 |
| Cluster-40555.182390 | 342.9 | 279.2 | 109.56 | 118.04 | 215.77 | 182.27 | 90.42 | 84.91 | -1.30 | 3.23E-06 | -1.12 | 3.06E-03 | 542 |
| Cluster-40555.194768 | 15.85 | 19.65 | 5.69 | 7.45 | 41.77 | 53.41 | 12.54 | 13.14 | -1.30 | 2.18E-03 | -1.83 | 4.06E-05 | 5628 |
| Cluster-40555.238971 | 4.7 | 4.52 | 1.72 | 1.73 | 4.76 | 5.36 | 2.28 | 1.41 | -1.28 | 2.52E-02 | -1.40 | 8.84E-03 | 2709 |
| Cluster-40555.197041 | 69.23 | 64.1 | 24.84 | 25.64 | 163.65 | 142.21 | 48.12 | 49.63 | -1.26 | 2.63E-06 | -1.58 | 2.97E-07 | 1875 |
| Cluster-40555.205707 | 23.15 | 24.09 | 9.31 | 8.85 | 27.87 | 20.23 | 12.37 | 12.18 | -1.24 | 1.88E-04 | -0.90 | 4.19E-02 | 1877 |
| Cluster-40555.170921 | 9.32 | 11.94 | 3.92 | 4.28 | 18.93 | 25.89 | 2.77 | 1.77 | -1.24 | 7.89E-03 | -3.26 | 4.53E-07 | 1822 |
| Cluster-40555.195918 | 9.97 | 7.12 | 4.39 | 2.25 | 38.14 | 27.5 | 6.46 | 4.86 | -1.23 | 3.01E-02 | -2.47 | 1.14E-10 | 1414 |
| Cluster-40555.193222 | 208.41 | 268.2 | 93.22 | 93.21 | 112.55 | 113.51 | 52.46 | 37.92 | -1.22 | 5.07E-03 | -1.26 | 1.99E-04 | 1325 |
| Cluster-40555.176426 | 92.77 | 71.63 | 32.41 | 32.02 | 127.9 | 125.56 | 49.12 | 52.62 | -1.20 | 8.52E-05 | -1.25 | 2.99E-04 | 942 |
| Cluster-40555.184437 | 19.94 | 14.3 | 6.51 | 7 | 26.03 | 18.89 | 7.15 | 6.22 | -1.19 | 1.50E-03 | -1.68 | 3.47E-06 | 2020 |
| Cluster-40555.172051 | 31.55 | 25.81 | 12.49 | 10.39 | 30.02 | 27.8 | 13.44 | 16.1 | -1.18 | 9.02E-05 | -0.90 | 3.12E-02 | 2121 |
| Cluster-40555.210563 | 17.87 | 22.06 | 6.74 | 9.36 | 27.83 | 32.18 | 8.83 | 13.54 | -1.17 | 2.81E-02 | -1.36 | 1.75E-03 | 991 |
| Cluster-40555.225819 | 11.55 | 8.56 | 5.24 | 3.05 | 10.5 | 8.97 | 4.12 | 3.65 | -1.14 | 3.61E-02 | -1.26 | 2.99E-02 | 1387 |
| Cluster-40555.186579 | 12.71 | 13.85 | 5.58 | 5.4 | 23.58 | 24.71 | 4.8 | 4.49 | -1.14 | 1.09E-03 | -2.31 | 3.22E-12 | 2870 |
| Cluster-40555.224907 | 52.63 | 41.04 | 25.34 | 13.36 | 72.6 | 50.17 | 24.63 | 26.71 | -1.14 | 2.49E-03 | -1.18 | 3.95E-03 | 1008 |
| Cluster-40555.193757 | 5.59 | 5.21 | 2.49 | 2 | 9.43 | 11.57 | 4.53 | 4.79 | -1.13 | 7.53E-03 | -1.11 | 6.46E-03 | 3962 |
| Cluster-40555.191584 | 299.8 | 362.06 | 124.52 | 151.4 | 167.03 | 165.61 | 82.85 | 75.63 | -1.13 | 2.23E-03 | -1.01 | 3.73E-03 | 2311 |
| Cluster-40555.203550 | 72.72 | 65.52 | 33 | 25.2 | 65.25 | 52.21 | 26.48 | 25.14 | -1.11 | 1.93E-03 | -1.12 | 1.17E-02 | 696 |
| Cluster-40555.181984 | 36.02 | 41.37 | 14.02 | 18.85 | 126.83 | 156.99 | 13.04 | 45.79 | -1.10 | 1.60E-02 | -2.19 | 1.39E-04 | 799 |
| Cluster-40555.205481 | 37.69 | 34.84 | 17.13 | 13.83 | 22.9 | 23.49 | 11.91 | 10.3 | -1.09 | 9.51E-04 | -1.00 | 3.52E-02 | 1346 |

| | | | | | | | | | | | | | |
|---|---|---|---|---|---|---|---|---|---|---|---|---|---|
| Cluster-40555.188010 | 88.66 | 95.54 | 49 | 30.52 | 393.83 | 282.76 | 72.16 | 86.49 | -1.08 | 1.62E-04 | -2.02 | 3.85E-08 | 943 |
| Cluster-40555.190913 | 35.24 | 43.51 | 17.2 | 16.94 | 44.09 | 48.25 | 15.53 | 12.24 | -1.07 | 2.72E-03 | -1.67 | 7.84E-07 | 1701 |
| Cluster-40555.177269 | 262.95 | 235.57 | 101.01 | 113.28 | 276.61 | 256.7 | 89.32 | 69.3 | -1.07 | 3.39E-05 | -1.69 | 4.19E-08 | 1112 |
| Cluster-40555.220604 | 25.76 | 20.38 | 9.32 | 10.73 | 50.54 | 37.22 | 10.47 | 8.6 | -1.05 | 1.24E-02 | -2.13 | 5.91E-10 | 1353 |
| Cluster-40555.181574 | 39.98 | 46.85 | 16.98 | 21.04 | 18.59 | 19.61 | 7.68 | 7.18 | -1.05 | 1.79E-03 | -1.30 | 1.31E-03 | 1874 |
| Cluster-40555.206274 | 115.75 | 87.5 | 36.39 | 51.78 | 125.49 | 142.72 | 55.95 | 56.67 | -1.05 | 5.12E-03 | -1.19 | 7.22E-04 | 938 |
| Cluster-40555.192626 | 46.67 | 35.72 | 17.34 | 18.54 | 53.46 | 39.9 | 19.97 | 20.27 | -1.05 | 9.99E-03 | -1.14 | 6.22E-03 | 908 |
| Cluster-40555.205556 | 17.69 | 16.03 | 6.79 | 7.95 | 15.66 | 14.83 | 3.29 | 3.17 | -1.05 | 4.09E-02 | -2.18 | 2.02E-06 | 1247 |
| Cluster-40555.185238 | 25.69 | 27.75 | 11.96 | 11.72 | 41.45 | 40.11 | 15.12 | 13.13 | -1.04 | 6.16E-04 | -1.47 | 9.94E-06 | 2597 |
| Cluster-40555.183967 | 10.82 | 12.42 | 4.44 | 5.89 | 8.93 | 9.98 | 4.04 | 4.94 | -1.03 | 1.27E-02 | -1.01 | 3.81E-02 | 2622 |
| Cluster-40555.188956 | 18.98 | 18.04 | 7.21 | 9.22 | 23.45 | 22.51 | 4.15 | 4.45 | -1.03 | 2.21E-02 | -2.35 | 7.04E-10 | 1499 |
| Cluster-40555.187537 | 124.83 | 138.74 | 55.81 | 61.7 | 295.36 | 278.33 | 110.81 | 109.4 | -1.03 | 3.87E-04 | -1.32 | 4.63E-05 | 926 |
| Cluster-40555.195108 | 38.81 | 30.1 | 15.47 | 15.19 | 32.79 | 33.13 | 11.58 | 12.28 | -1.02 | 3.45E-02 | -1.40 | 2.69E-03 | 788 |
| Cluster-40555.189351 | 58.17 | 65.33 | 26.55 | 28.95 | 29.26 | 28.69 | 10.46 | 14.21 | -1.02 | 2.09E-04 | -1.16 | 1.63E-03 | 2254 |
| Cluster-40555.206048 | 15.9 | 13.4 | 6.44 | 6.73 | 10.43 | 9.22 | 3.61 | 4.58 | -1.01 | 1.57E-02 | -1.19 | 1.46E-02 | 1979 |
| Cluster-40555.183981 | 40.4 | 46.7 | 21.64 | 18.07 | 46.11 | 43.46 | 18.18 | 25.05 | -1.00 | 2.16E-03 | -0.98 | 1.71E-02 | 1237 |
| Cluster-40555.152838 | 21.82 | 21.8 | 11.6 | 8.35 | 37.24 | 41.91 | 14.81 | 11.09 | -0.99 | 4.32E-02 | -1.56 | 1.03E-04 | 961 |
| Cluster-40555.189350 | 724.4 | 495.34 | 287.18 | 270.75 | 449.49 | 417.91 | 178.95 | 159.08 | -0.98 | 1.15E-02 | -1.30 | 5.40E-05 | 859 |
| Cluster-40555.182892 | 39.67 | 42.65 | 15.5 | 22.38 | 68.8 | 61 | 27.67 | 26.09 | -0.98 | 3.44E-03 | -1.20 | 4.49E-04 | 1893 |
| Cluster-40555.168531 | 10.74 | 10.54 | 5.67 | 4.31 | 7.98 | 8.98 | 2.26 | 1.88 | -0.96 | 1.31E-02 | -1.98 | 1.87E-06 | 2742 |
| Cluster-40555.198234 | 15.08 | 12.67 | 7.11 | 6.2 | 11.38 | 9.58 | 2.43 | 2.28 | -0.92 | 7.84E-03 | -2.09 | 2.14E-08 | 3249 |
| Cluster-40555.182705 | 134.27 | 164 | 73.91 | 72.01 | 25.08 | 27.57 | 9.85 | 10.94 | -0.90 | 9.04E-03 | -1.27 | 1.97E-03 | 1369 |
| Cluster-40555.194588 | 81.1 | 64.75 | 35.32 | 36.36 | 28.57 | 21.54 | 12.1 | 12.22 | -0.88 | 1.58E-03 | -0.97 | 1.48E-02 | 2409 |
| Cluster-40555.190451 | 189.58 | 163.6 | 90.25 | 82.76 | 182.99 | 185.51 | 76.14 | 72 | -0.88 | 1.58E-02 | -1.26 | 1.24E-03 | 477 |
| Cluster-40555.188521 | 111.6 | 76.13 | 45.6 | 46.78 | 154.59 | 128.24 | 68.49 | 56.65 | -0.87 | 4.78E-02 | -1.11 | 8.28E-04 | 2794 |
| Cluster-40555.190585 | 153.88 | 142.27 | 65.02 | 81.16 | 210.36 | 221.17 | 92.75 | 68.5 | -0.87 | 2.03E-02 | -1.37 | 1.17E-04 | 562 |
| Cluster-40555.182613 | 157.29 | 139.11 | 76.26 | 70.97 | 315.31 | 253.32 | 106.4 | 106.24 | -0.86 | 7.57E-03 | -1.35 | 4.23E-05 | 656 |
| Cluster-40555.190070 | 48.08 | 43.04 | 25.02 | 20.76 | 71.13 | 64.91 | 21.69 | 14.71 | -0.85 | 1.04E-02 | -1.84 | 2.70E-08 | 1314 |
| Cluster-40555.188179 | 38.57 | 43.74 | 18.84 | 22.52 | 54.83 | 50.13 | 24.02 | 21.35 | -0.85 | 1.34E-02 | -1.15 | 1.65E-03 | 1610 |
| Cluster-40555.193627 | 173.18 | 130.3 | 77.61 | 76.08 | 294.24 | 237.19 | 139.85 | 129.3 | -0.83 | 3.44E-03 | -0.91 | 1.16E-02 | 1182 |
| Cluster-40555.181011 | 11.61 | 11.01 | 5.96 | 5.66 | 6.92 | 5.45 | 3.48 | 2.17 | -0.82 | 4.24E-02 | -1.07 | 4.12E-02 | 3020 |
| Cluster-40555.173149 | 20.6 | 25.68 | 12.7 | 11.23 | 25.36 | 31.34 | 6.63 | 6.54 | -0.82 | 2.85E-02 | -2.05 | 2.21E-07 | 2317 |

| | | | | | | | | | | | | | |
|---|---|---|---|---|---|---|---|---|---|---|---|---|---|
| Cluster-40555.105731 | 2.18 | 1.14 | 0 | 0 | 0.95 | 0.75 | 0 | 0 | 0.00 | 1.98E-06 | 0.00 | 6.13E-04 | 2082 |
| Cluster-40555.106829 | 0.77 | 0.5 | 0 | 0 | 0.69 | 0.61 | 0 | 0 | 0.00 | 7.54E-03 | 0.00 | 1.26E-03 | 2463 |
| Cluster-40555.115561 | 2.04 | 1.72 | 0 | 0 | 1.46 | 1 | 0.25 | 0 | 0.00 | 1.69E-05 | -3.22 | 4.35E-02 | 1632 |
| Cluster-40555.12235 | 4.39 | 5.64 | 0 | 0 | 9.27 | 4.49 | 0 | 0 | 0.00 | 1.42E-02 | 0.00 | 3.69E-04 | 507 |
| Cluster-40555.124760 | 3.32 | 1.2 | 0 | 0 | 2.43 | 3.37 | 0 | 0.31 | 0.00 | 9.79E-03 | -4.02 | 1.63E-03 | 1037 |
| Cluster-40555.125526 | 2.98 | 2.49 | 0 | 0 | 2.34 | 2.76 | 0 | 0 | 0.00 | 2.16E-11 | 0.00 | 1.25E-12 | 2189 |
| Cluster-40555.127485 | 0.78 | 0.64 | 0 | 0 | 0.66 | 0.51 | 0.05 | 0 | 0.00 | 2.47E-04 | -4.45 | 6.44E-03 | 3144 |
| Cluster-40555.127856 | 3.37 | 5.06 | 0 | 0 | 8.64 | 7.93 | 0.6 | 1.86 | 0.00 | 1.99E-04 | -2.67 | 1.52E-03 | 751 |
| Cluster-40555.128381 | 1.09 | 1.01 | 0 | 0 | 0.31 | 0.21 | 0 | 0 | 0.00 | 1.14E-07 | 0.00 | 3.16E-02 | 3642 |
| Cluster-40555.130030 | 1.65 | 1.29 | 0 | 0 | 1.82 | 2.31 | 0 | 0 | 0.00 | 4.98E-03 | 0.00 | 2.50E-05 | 1256 |
| Cluster-40555.130307 | 13.15 | 14.29 | 0 | 0 | 12.06 | 8.86 | 0.18 | 0.14 | 0.00 | 6.63E-15 | -6.03 | 8.32E-11 | 767 |
| Cluster-40555.132196 | 5.46 | 4.56 | 0 | 0 | 11.1 | 6.13 | 0 | 0 | 0.00 | 9.92E-08 | 0.00 | 6.73E-09 | 981 |
| Cluster-40555.133050 | 2.42 | 1.52 | 0 | 0 | 1.09 | 0.58 | 0 | 0 | 0.00 | 2.91E-11 | 0.00 | 9.39E-06 | 2934 |
| Cluster-40555.133099 | 3.34 | 7.51 | 0 | 0 | 3.29 | 2.58 | 0 | 0 | 0.00 | 7.16E-03 | 0.00 | 4.77E-02 | 554 |
| Cluster-40555.133586 | 1.76 | 1.99 | 0 | 0 | 4.28 | 2.81 | 0.26 | 0 | 0.00 | 5.02E-08 | -4.76 | 1.13E-10 | 2239 |
| Cluster-40555.134082 | 8.12 | 8.92 | 0 | 0 | 1.56 | 2 | 0 | 0 | 0.00 | 1.11E-12 | 0.00 | 4.45E-03 | 950 |
| Cluster-40555.137580 | 17.19 | 11.46 | 0 | 0 | 4.23 | 5.29 | 0 | 0 | 0.00 | 5.14E-09 | 0.00 | 1.84E-03 | 557 |
| Cluster-40555.137588 | 14.48 | 19 | 0 | 0 | 1.11 | 1.31 | 0 | 0 | 0.00 | 7.12E-24 | 0.00 | 1.60E-03 | 1424 |
| Cluster-40555.139437 | 0.32 | 0.27 | 0 | 0 | 0.62 | 0.38 | 0 | 0 | 0.00 | 2.32E-02 | 0.00 | 6.05E-05 | 4108 |
| Cluster-40555.140087 | 9.89 | 14.74 | 0 | 0 | 0.98 | 0.88 | 0 | 0 | 0.00 | 6.96E-13 | 0.00 | 2.67E-02 | 1251 |
| Cluster-40555.140792 | 1.94 | 0.72 | 0 | 0 | 1.16 | 0.86 | 0.08 | 0.15 | 0.00 | 2.58E-03 | -3.16 | 1.23E-02 | 2406 |
| Cluster-40555.141940 | 0.54 | 0.45 | 0 | 0 | 0.86 | 1.01 | 0 | 0 | 0.00 | 4.02E-03 | 0.00 | 1.76E-07 | 3315 |
| Cluster-40555.144506 | 4.51 | 2.94 | 0 | 0 | 3.5 | 2.97 | 0 | 0 | 0.00 | 1.35E-04 | 0.00 | 7.63E-05 | 851 |
| Cluster-40555.144646 | 1.53 | 1.26 | 0 | 0 | 1.66 | 1.21 | 0 | 0 | 0.00 | 3.22E-03 | 0.00 | 5.36E-04 | 1365 |
| Cluster-40555.145387 | 1.98 | 2.28 | 0 | 0 | 3.12 | 3.81 | 0 | 0.72 | 0.00 | 1.25E-03 | -3.19 | 2.57E-03 | 1069 |
| Cluster-40555.145859 | 0.41 | 0.52 | 0 | 0 | 0.66 | 0.99 | 0.2 | 0 | 0.00 | 3.70E-02 | -3.03 | 4.11E-02 | 2522 |
| Cluster-40555.146384 | 8.2 | 5.78 | 0 | 0 | 7.44 | 5.68 | 0 | 0.08 | 0.00 | 2.89E-18 | -7.19 | 1.02E-17 | 1506 |
| Cluster-40555.146917 | 12.87 | 18.03 | 0 | 0 | 7.23 | 7.53 | 0.37 | 2.06 | 0.00 | 2.51E-18 | -2.51 | 2.50E-06 | 1575 |
| Cluster-40555.147227 | 0.68 | 1.2 | 0 | 0 | 1.17 | 1.57 | 0 | 0 | 0.00 | 2.78E-02 | 0.00 | 4.83E-04 | 1424 |
| Cluster-40555.149949 | 1.01 | 0.96 | 0 | 0 | 1 | 1.19 | 0 | 0 | 0.00 | 5.59E-04 | 0.00 | 2.19E-05 | 2167 |
| Cluster-40555.151095 | 2.07 | 2.4 | 0 | 0 | 22.79 | 14.45 | 0 | 0 | 0.00 | 2.72E-03 | 0.00 | 7.88E-18 | 963 |
| Cluster-40555.151396 | 16.13 | 13.29 | 0 | 0 | 44.93 | 61.44 | 1.25 | 0.71 | 0.00 | 4.95E-06 | -5.73 | 1.23E-11 | 453 |

| | | | | | | | | | | | | | |
|---|---|---|---|---|---|---|---|---|---|---|---|---|---|
| Cluster-40555.151984 | 2.92 | 2.93 | 0 | 0 | 0.89 | 1.28 | 0 | 0 | 0.00 | 1.77E-09 | 0.00 | 4.09E-04 | 1752 |
| Cluster-40555.151994 | 3.96 | 3.36 | 0 | 0 | 1.71 | 1.21 | 0 | 0 | 0.00 | 3.16E-06 | 0.00 | 6.45E-03 | 1058 |
| Cluster-40555.152801 | 0.95 | 2.18 | 0 | 0 | 1.03 | 1.4 | 0 | 0 | 0.00 | 9.52E-03 | 0.00 | 9.23E-03 | 1175 |
| Cluster-40555.153581 | 0.58 | 1.01 | 0 | 0 | 2.01 | 2.37 | 0 | 0.29 | 0.00 | 5.52E-03 | -3.81 | 1.86E-05 | 2036 |
| Cluster-40555.156559 | 1.36 | 1.6 | 0 | 0 | 2.57 | 1.15 | 0 | 0 | 0.00 | 1.73E-02 | 0.00 | 1.39E-03 | 1070 |
| Cluster-40555.157999 | 19.98 | 28.33 | 0 | 0 | 4.33 | 4.59 | 0.45 | 0.35 | 0.00 | 1.18E-17 | -3.45 | 6.12E-05 | 1179 |
| Cluster-40555.158820 | 2.67 | 2.09 | 0 | 0 | 5.36 | 5.63 | 0 | 0.2 | 0.00 | 1.25E-04 | -5.90 | 2.68E-10 | 1173 |
| Cluster-40555.158893 | 3.24 | 3.37 | 0 | 0 | 4.48 | 2.45 | 0.42 | 0 | 0.00 | 1.54E-03 | -4.06 | 9.85E-03 | 768 |
| Cluster-40555.159443 | 4.49 | 3.47 | 0 | 0 | 5.69 | 3.94 | 0.67 | 0.58 | 0.00 | 3.47E-10 | -2.88 | 3.76E-05 | 1451 |
| Cluster-40555.159486 | 1.47 | 1.11 | 0 | 0 | 1.32 | 1.96 | 0.59 | 0 | 0.00 | 2.05E-05 | -2.47 | 4.70E-02 | 2239 |
| Cluster-40555.159510 | 2.72 | 1.46 | 0 | 0 | 3.12 | 2.3 | 0.24 | 0.2 | 0.00 | 3.33E-07 | -3.52 | 1.16E-05 | 1970 |
| Cluster-40555.159961 | 6.17 | 8.7 | 0 | 0 | 2.2 | 3.56 | 0 | 0 | 0.00 | 6.61E-17 | 0.00 | 2.33E-08 | 2398 |
| Cluster-40555.160854 | 4.03 | 4.19 | 0 | 0 | 1.98 | 1.83 | 0 | 0 | 0.00 | 6.98E-07 | 0.00 | 9.28E-04 | 1034 |
| Cluster-40555.161306 | 0.82 | 0.79 | 0 | 0 | 1.28 | 2.29 | 0.26 | 0 | 0.00 | 3.24E-02 | -3.76 | 1.42E-02 | 1603 |
| Cluster-40555.161509 | 2.11 | 1.78 | 0 | 0 | 2.72 | 3.77 | 0.6 | 0.51 | 0.00 | 4.36E-04 | -2.46 | 1.29E-02 | 1258 |
| Cluster-40555.162821 | 0.86 | 1.1 | 0 | 0 | 0.43 | 0.77 | 0 | 0 | 0.00 | 5.90E-03 | 0.00 | 4.45E-02 | 1695 |
| Cluster-40555.163920 | 17.6 | 18.13 | 0 | 0 | 6.94 | 6.71 | 0.64 | 0.62 | 0.00 | 2.96E-25 | -3.35 | 2.51E-05 | 947 |
| Cluster-40555.164499 | 3.73 | 2.06 | 0 | 0 | 108.45 | 82.46 | 13.56 | 0 | 0.00 | 7.43E-05 | -3.78 | 9.28E-20 | 1060 |
| Cluster-40555.165378 | 2.21 | 2.32 | 0 | 0 | 1.33 | 1.65 | 0 | 0 | 0.00 | 3.51E-08 | 0.00 | 1.77E-06 | 1925 |
| Cluster-40555.165988 | 27.12 | 25.08 | 0 | 0 | 16.28 | 12.1 | 0.24 | 0 | 0.00 | 4.90E-27 | -7.11 | 1.31E-15 | 771 |
| Cluster-40555.166162 | 2.56 | 3.06 | 0 | 0 | 2.06 | 3.13 | 0 | 0 | 0.00 | 4.57E-18 | 0.00 | 7.47E-11 | 3281 |
| Cluster-40555.166351 | 1.47 | 2.54 | 0 | 0 | 3.48 | 3.15 | 0.42 | 0.48 | 0.00 | 2.77E-05 | -2.84 | 7.75E-04 | 1486 |
| Cluster-40555.166467 | 9.56 | 10.7 | 0 | 0 | 4.41 | 2.8 | 0 | 0 | 0.00 | 4.02E-38 | 0.00 | 3.93E-15 | 2218 |
| Cluster-40555.167370 | 29.36 | 26.79 | 0 | 0 | 1.97 | 1.62 | 0 | 0 | 0.00 | 2.19E-55 | 0.00 | 2.39E-05 | 1414 |
| Cluster-40555.168138 | 0.52 | 0.39 | 0 | 0 | 1.08 | 1.34 | 0.2 | 0.19 | 0.00 | 2.72E-06 | -2.59 | 1.36E-05 | 6739 |
| Cluster-40555.168638 | 2.4 | 1.69 | 0 | 0 | 1.33 | 1.24 | 0 | 0 | 0.00 | 2.26E-05 | 0.00 | 5.14E-04 | 1503 |
| Cluster-40555.169050 | 1.69 | 1.93 | 0 | 0 | 1.26 | 0.61 | 0 | 0 | 0.00 | 5.14E-07 | 0.00 | 4.91E-04 | 2052 |
| Cluster-40555.169380 | 12.32 | 9.63 | 0 | 0 | 2.63 | 2.1 | 0 | 0 | 0.00 | 1.12E-24 | 0.00 | 9.68E-07 | 1358 |
| Cluster-40555.169632 | 0.39 | 0.22 | 0 | 0 | 0.66 | 0.57 | 0 | 0 | 0.00 | 9.24E-03 | 0.00 | 4.60E-07 | 4678 |
| Cluster-40555.169847 | 17.09 | 14.07 | 0 | 0 | 6.23 | 6.93 | 0 | 0 | 0.00 | 1.60E-33 | 0.00 | 6.76E-18 | 1359 |
| Cluster-40555.170503 | 9.18 | 7.55 | 0 | 0 | 7.98 | 9.1 | 0.94 | 0.97 | 0.00 | 4.01E-17 | -3.09 | 1.89E-07 | 1225 |
| Cluster-40555.171664 | 5.38 | 1.87 | 0 | 0 | 2.33 | 1.65 | 0 | 0 | 0.00 | 3.22E-03 | 0.00 | 7.63E-06 | 1411 |

| | | | | | | | | | | | | | |
|---|---|---|---|---|---|---|---|---|---|---|---|---|---|
| Cluster-40555.173496 | 2.26 | 1.9 | 0 | 0 | 6 | 5.23 | 0.11 | 0 | 0.00 | 1.69E-05 | -6.48 | 1.92E-14 | 1497 |
| Cluster-40555.174642 | 5.19 | 3.06 | 0 | 0 | 6.89 | 5.12 | 0.8 | 0 | 0.00 | 2.39E-11 | -3.84 | 4.49E-11 | 1869 |
| Cluster-40555.175624 | 2.67 | 1.85 | 0 | 0 | 2.23 | 1.81 | 0.15 | 0.22 | 0.00 | 2.69E-04 | -3.26 | 2.64E-02 | 1164 |
| Cluster-40555.176325 | 2.52 | 2.44 | 0 | 0 | 4.18 | 3.84 | 0.53 | 1.08 | 0.00 | 1.76E-08 | -2.22 | 7.25E-04 | 1837 |
| Cluster-40555.177151 | 6.31 | 7.51 | 0 | 0 | 3.94 | 3.3 | 0 | 0.31 | 0.00 | 7.23E-16 | -4.53 | 1.51E-06 | 1328 |
| Cluster-40555.177376 | 3.81 | 3.27 | 0 | 0 | 6.35 | 2.87 | 0.44 | 0 | 0.00 | 4.64E-05 | -4.30 | 1.20E-02 | 933 |
| Cluster-40555.177437 | 4.49 | 7.13 | 0 | 0 | 4.14 | 2.19 | 0.48 | 0 | 0.00 | 2.72E-09 | -3.69 | 2.86E-03 | 1437 |
| Cluster-40555.178300 | 0.68 | 1.48 | 0 | 0 | 1.48 | 2.09 | 0.14 | 0 | 0.00 | 1.74E-03 | -4.64 | 1.88E-06 | 2389 |
| Cluster-40555.178604 | 2.63 | 1.37 | 0 | 0 | 3.49 | 2.82 | 0 | 0 | 0.00 | 1.14E-07 | 0.00 | 8.57E-22 | 3137 |
| Cluster-40555.179235 | 4.02 | 10.46 | 0 | 0 | 17.67 | 23.95 | 0 | 0 | 0.00 | 4.73E-04 | 0.00 | 6.85E-25 | 2052 |
| Cluster-40555.179326 | 0.37 | 0.63 | 0 | 0 | 0.92 | 1.22 | 0 | 0 | 0.00 | 2.49E-02 | 0.00 | 3.10E-06 | 2512 |
| Cluster-40555.179602 | 4.58 | 4.78 | 0 | 0 | 1.83 | 1.2 | 0 | 0 | 0.00 | 7.30E-06 | 0.00 | 3.67E-02 | 845 |
| Cluster-40555.180961 | 25.61 | 15.28 | 0 | 0 | 18.12 | 12.46 | 0.36 | 0.78 | 0.00 | 2.91E-06 | -4.68 | 8.51E-04 | 407 |
| Cluster-40555.180998 | 22.14 | 14.74 | 0 | 0 | 4.53 | 4.65 | 0.12 | 0.24 | 0.00 | 1.38E-25 | -4.50 | 6.75E-08 | 1330 |
| Cluster-40555.181023 | 3.75 | 4.63 | 0 | 0 | 2.59 | 3.71 | 0 | 0 | 0.00 | 2.98E-21 | 0.00 | 4.35E-13 | 2670 |
| Cluster-40555.181030 | 10.14 | 8.42 | 0 | 0 | 8.59 | 7.78 | 0 | 0.21 | 0.00 | 1.33E-15 | -6.26 | 2.32E-13 | 1049 |
| Cluster-40555.182515 | 3.19 | 5.36 | 0 | 0 | 3.23 | 2.88 | 0 | 0 | 0.00 | 2.61E-08 | 0.00 | 2.45E-14 | 2102 |
| Cluster-40555.183574 | 3.98 | 4.52 | 0 | 0 | 4.41 | 3.97 | 0 | 0 | 0.00 | 3.19E-25 | 0.00 | 2.09E-27 | 3135 |
| Cluster-40555.183649 | 13.29 | 16.65 | 0 | 0 | 3.57 | 3.15 | 0 | 0 | 0.00 | 2.17E-35 | 0.00 | 9.00E-20 | 2667 |
| Cluster-40555.183685 | 2.95 | 2.86 | 0 | 0 | 2.99 | 2.6 | 0 | 0 | 0.00 | 8.86E-03 | 0.00 | 3.51E-03 | 722 |
| Cluster-40555.183899 | 70.3 | 85.63 | 0 | 0 | 3.12 | 5.14 | 0.22 | 0 | 0.00 | 2.09E-32 | -5.67 | 9.90E-03 | 584 |
| Cluster-40555.183960 | 15.61 | 13.88 | 0 | 0 | 3.13 | 4.74 | 0 | 0 | 0.00 | 2.50E-39 | 0.00 | 1.24E-09 | 1667 |
| Cluster-40555.184310 | 26.41 | 20.59 | 0 | 0 | 9.43 | 6.31 | 0 | 0 | 0.00 | 4.64E-39 | 0.00 | 3.91E-17 | 1139 |
| Cluster-40555.184373 | 47.7 | 32.68 | 0 | 0 | 70.69 | 57.26 | 1.08 | 1.13 | 0.00 | 1.21E-35 | -5.78 | 3.44E-50 | 1328 |
| Cluster-40555.185389 | 1.14 | 1.42 | 0 | 0 | 0.44 | 0.73 | 0 | 0 | 0.00 | 3.34E-08 | 0.00 | 1.90E-04 | 3200 |
| Cluster-40555.186556 | 2.01 | 2.05 | 0 | 0 | 0.51 | 0.32 | 0 | 0 | 0.00 | 6.72E-10 | 0.00 | 2.39E-02 | 2506 |
| Cluster-40555.187097 | 9.72 | 14.39 | 0 | 0 | 10.29 | 8.21 | 0.53 | 0 | 0.00 | 1.19E-07 | -5.07 | 6.31E-05 | 551 |
| Cluster-40555.187165 | 10.49 | 20.7 | 0 | 0.01 | 0.74 | 1.48 | 0 | 0 | 0.00 | 3.42E-07 | 0.00 | 2.00E-03 | 1612 |
| Cluster-40555.187194 | 7.71 | 12.08 | 0 | 0 | 0.61 | 0.34 | 0 | 0 | 0.00 | 4.59E-13 | 0.00 | 1.03E-02 | 2540 |
| Cluster-40555.187500 | 4.82 | 6.39 | 0 | 0 | 11.07 | 11.55 | 0 | 0 | 0.00 | 1.05E-18 | 0.00 | 1.92E-38 | 1959 |
| Cluster-40555.187713 | 2.08 | 2.51 | 0 | 0 | 3.75 | 10.71 | 0 | 0 | 0.00 | 2.68E-02 | 0.00 | 1.91E-02 | 745 |
| Cluster-40555.187809 | 27.21 | 11.74 | 0 | 0 | 2.91 | 1.76 | 0 | 0 | 0.00 | 1.75E-07 | 0.00 | 4.32E-10 | 1932 |

| | | | | | | | | | | | | | |
|---|---|---|---|---|---|---|---|---|---|---|---|---|---|
| Cluster-40555.190977 | 8.69 | 1.74 | 0 | 0 | 3.59 | 2.95 | 0 | 0 | 0.00 | 3.83E-02 | 0.00 | 1.22E-13 | 1902 |
| Cluster-40555.191616 | 38.65 | 26.55 | 0 | 0 | 61.34 | 64.09 | 3.29 | 2.09 | 0.00 | 2.01E-37 | -4.49 | 6.59E-40 | 1901 |
| Cluster-40555.191756 | 1.43 | 4.56 | 0 | 0 | 5.75 | 4.6 | 0 | 0 | 0.00 | 2.52E-02 | 0.00 | 3.79E-14 | 1338 |
| Cluster-40555.191827 | 10.9 | 7.55 | 0 | 0 | 5.38 | 6.26 | 0 | 0 | 0.00 | 1.45E-13 | 0.00 | 2.54E-10 | 952 |
| Cluster-40555.192629 | 2.3 | 2.38 | 0 | 0 | 1.25 | 1.08 | 0 | 0.08 | 0.00 | 9.35E-10 | -4.75 | 3.94E-04 | 2189 |
| Cluster-40555.193082 | 12.9 | 20.27 | 0 | 0 | 24.11 | 20.29 | 0 | 0 | 0.00 | 8.51E-14 | 0.00 | 3.77E-60 | 2078 |
| Cluster-40555.193254 | 4.37 | 7.79 | 0 | 0 | 5.44 | 5.07 | 0 | 1.99 | 0.00 | 6.92E-09 | -2.29 | 3.83E-04 | 3487 |
| Cluster-40555.193777 | 10.97 | 10.32 | 0 | 0 | 6.98 | 7.1 | 0 | 0 | 0.00 | 3.72E-12 | 0.00 | 1.41E-09 | 790 |
| Cluster-40555.193897 | 0.65 | 1.6 | 0 | 0 | 8.39 | 7.29 | 0 | 0 | 0.00 | 5.30E-03 | 0.00 | 9.07E-37 | 2556 |
| Cluster-40555.194173 | 1.28 | 2.27 | 0 | 0 | 0.97 | 1 | 0 | 0 | 0.00 | 1.33E-05 | 0.00 | 1.78E-04 | 2038 |
| Cluster-40555.194300 | 9.69 | 15.84 | 0 | 0 | 2.57 | 2.68 | 0 | 0 | 0.00 | 6.66E-09 | 0.00 | 1.33E-04 | 952 |
| Cluster-40555.194660 | 0.82 | 0.87 | 0 | 0 | 1.22 | 1.49 | 0 | 0 | 0.00 | 2.59E-04 | 0.00 | 1.71E-08 | 2676 |
| Cluster-40555.194772 | 1.73 | 1.95 | 0 | 0 | 5.41 | 5.12 | 0 | 0 | 0.00 | 1.21E-07 | 0.00 | 1.04E-23 | 2168 |
| Cluster-40555.194886 | 11.91 | 10.65 | 0 | 0 | 6.99 | 2.23 | 0 | 0 | 0.00 | 1.49E-37 | 0.00 | 3.11E-03 | 1996 |
| Cluster-40555.194997 | 2.6 | 4.21 | 0 | 0 | 2.21 | 2.83 | 0.09 | 0 | 0.00 | 1.72E-06 | -5.41 | 2.13E-04 | 1134 |
| Cluster-40555.195193 | 2.06 | 1.31 | 0 | 0 | 17.02 | 16.97 | 1.9 | 0.07 | 0.00 | 1.33E-10 | -4.09 | 1.73E-28 | 3195 |
| Cluster-40555.196437 | 2.46 | 3.68 | 0 | 0 | 2.16 | 2.04 | 0 | 0 | 0.00 | 8.88E-06 | 0.00 | 1.16E-04 | 1132 |
| Cluster-40555.196602 | 4.51 | 5.54 | 0 | 0 | 2.06 | 3.07 | 0.2 | 0.24 | 0.00 | 3.13E-18 | -3.44 | 9.64E-05 | 1962 |
| Cluster-40555.197362 | 1.65 | 1.55 | 0 | 0 | 0.39 | 0.57 | 0 | 0 | 0.00 | 1.93E-09 | 0.00 | 2.43E-03 | 2975 |
| Cluster-40555.197978 | 0.56 | 0.54 | 0 | 0 | 2.11 | 1.66 | 0.24 | 0.38 | 0.00 | 2.36E-02 | -2.50 | 3.18E-03 | 2371 |
| Cluster-40555.198107 | 3.9 | 1.83 | 0 | 0 | 15.48 | 19.14 | 0 | 0 | 0.00 | 2.81E-04 | 0.00 | 5.13E-28 | 984 |
| Cluster-40555.198449 | 4.61 | 3.92 | 0 | 0 | 1.41 | 1.89 | 0 | 0 | 0.00 | 6.50E-16 | 0.00 | 1.46E-07 | 2015 |
| Cluster-40555.198926 | 10.85 | 10.25 | 0 | 0 | 6.18 | 5.28 | 0.2 | 0 | 0.00 | 4.21E-09 | -5.44 | 1.57E-04 | 662 |
| Cluster-40555.199040 | 0.69 | 1.18 | 0 | 0 | 0.95 | 0.72 | 0 | 0 | 0.00 | 2.76E-02 | 0.00 | 2.04E-02 | 1424 |
| Cluster-40555.199920 | 1.97 | 3.26 | 0 | 0 | 1.19 | 0.89 | 0 | 0 | 0.00 | 1.72E-07 | 0.00 | 5.79E-05 | 2120 |
| Cluster-40555.200316 | 0.75 | 1.19 | 0 | 0 | 2.09 | 1.48 | 0 | 0 | 0.00 | 2.47E-02 | 0.00 | 2.46E-05 | 1418 |
| Cluster-40555.200758 | 15.96 | 15.89 | 0 | 0 | 12.72 | 15.34 | 0 | 3.01 | 0.00 | 9.09E-51 | -3.12 | 1.85E-08 | 2062 |
| Cluster-40555.201603 | 2.51 | 4.47 | 0 | 0 | 3.88 | 2.29 | 0 | 0.25 | 0.00 | 4.56E-08 | -4.55 | 5.88E-07 | 3652 |
| Cluster-40555.201867 | 1.5 | 1.86 | 0 | 0 | 2.44 | 2.05 | 0.58 | 0 | 0.00 | 1.71E-08 | -2.91 | 9.88E-05 | 2588 |
| Cluster-40555.204568 | 21.33 | 22.62 | 0 | 0 | 5.71 | 5.54 | 0 | 0 | 0.00 | 1.19E-84 | 0.00 | 2.38E-33 | 3044 |
| Cluster-40555.204679 | 5.68 | 6.34 | 0 | 0 | 1.87 | 0.94 | 0 | 0 | 0.00 | 9.47E-16 | 0.00 | 2.37E-04 | 1484 |
| Cluster-40555.204824 | 10.53 | 14.04 | 0 | 0 | 8.65 | 9.15 | 0.17 | 0.14 | 0.00 | 1.89E-14 | -5.91 | 8.00E-10 | 804 |

| | | | | | | | | | | | | | |
|---|---|---|---|---|---|---|---|---|---|---|---|---|---|
| Cluster-40555.205050 | 0.58 | 0.29 | 0 | 0 | 3.84 | 2.62 | 0 | 0.08 | 0.00 | 7.31E-03 | -6.22 | 2.34E-18 | 3474 |
| Cluster-40555.205355 | 1.06 | 0.72 | 0 | 0 | 2.03 | 2.85 | 0 | 0 | 0.00 | 9.69E-05 | 0.00 | 3.74E-13 | 2798 |
| Cluster-40555.205536 | 1.88 | 1.34 | 0 | 0 | 4.39 | 4.69 | 0.14 | 0.07 | 0.00 | 2.29E-02 | -5.35 | 2.29E-06 | 979 |
| Cluster-40555.205957 | 1.22 | 1.05 | 0 | 0 | 2.89 | 2.64 | 0.26 | 0.71 | 0.00 | 1.21E-05 | -2.43 | 1.67E-04 | 2585 |
| Cluster-40555.206009 | 5.14 | 6.85 | 0 | 0 | 2.97 | 3 | 0 | 0 | 0.00 | 3.27E-17 | 0.00 | 2.33E-10 | 1601 |
| Cluster-40555.206049 | 18.3 | 14.52 | 0 | 0 | 6.33 | 5.44 | 0 | 0 | 0.00 | 1.65E-28 | 0.00 | 5.75E-13 | 1125 |
| Cluster-40555.206223 | 7.23 | 7.42 | 0 | 0 | 7.16 | 7.2 | 0 | 1.81 | 0.00 | 1.84E-12 | -2.91 | 2.69E-05 | 1051 |
| Cluster-40555.20644 | 6.9 | 3.64 | 0 | 0 | 5.26 | 6.94 | 0 | 0 | 0.00 | 4.74E-03 | 0.00 | 2.66E-04 | 545 |
| Cluster-40555.206687 | 5.33 | 5.15 | 0 | 0 | 2.99 | 5.03 | 0.29 | 1.13 | 0.00 | 1.02E-30 | -2.43 | 4.93E-02 | 3192 |
| Cluster-40555.207735 | 2.41 | 3.32 | 0 | 0 | 5.56 | 5.06 | 0 | 0 | 0.00 | 1.12E-04 | 0.00 | 2.12E-10 | 1022 |
| Cluster-40555.208480 | 1.5 | 2.85 | 0 | 0 | 2.85 | 1.52 | 0 | 0 | 0.00 | 2.26E-02 | 0.00 | 8.91E-03 | 780 |
| Cluster-40555.208550 | 7.46 | 5.29 | 0 | 0 | 10.56 | 9.66 | 0 | 0 | 0.00 | 5.17E-31 | 0.00 | 1.58E-45 | 2719 |
| Cluster-40555.209591 | 4.67 | 7.29 | 0 | 0 | 14.28 | 23.52 | 0 | 0 | 0.00 | 2.29E-10 | 0.00 | 1.01E-11 | 1769 |
| Cluster-40555.209694 | 6.51 | 4.34 | 0 | 0 | 3.3 | 3.76 | 0 | 1.09 | 0.00 | 2.42E-08 | -2.60 | 2.23E-02 | 979 |
| Cluster-40555.210334 | 66.48 | 55.15 | 0 | 0 | 171.1 | 153.13 | 5.84 | 4 | 0.00 | 4.93E-87 | -4.98 | 1.41E-50 | 1323 |
| Cluster-40555.211501 | 1.5 | 2.61 | 0 | 0 | 1.29 | 1.28 | 0 | 0 | 0.00 | 1.05E-04 | 0.00 | 1.90E-03 | 1320 |
| Cluster-40555.213722 | 0.34 | 0.67 | 0 | 0 | 1.9 | 2.48 | 0.74 | 0 | 0.00 | 5.01E-03 | -2.56 | 3.72E-03 | 3079 |
| Cluster-40555.213974 | 1.72 | 1.85 | 0 | 0 | 9.53 | 9.93 | 0 | 0 | 0.00 | 9.50E-10 | 0.00 | 3.99E-45 | 2776 |
| Cluster-40555.213998 | 1.76 | 1.8 | 0 | 0 | 3.71 | 3.44 | 0.88 | 0.38 | 0.00 | 6.05E-10 | -2.45 | 1.05E-05 | 2842 |
| Cluster-40555.214051 | 0.59 | 0.39 | 0 | 0 | 2.14 | 1.32 | 0.33 | 0.17 | 0.00 | 8.18E-04 | -2.70 | 1.80E-04 | 4035 |
| Cluster-40555.214133 | 3.81 | 4.68 | 0 | 0 | 7.55 | 9.47 | 0 | 0 | 0.00 | 6.05E-11 | 0.00 | 1.04E-23 | 1444 |
| Cluster-40555.214279 | 2.71 | 0.74 | 0 | 0 | 1.23 | 0.6 | 0 | 0 | 0.00 | 3.01E-02 | 0.00 | 3.39E-04 | 2071 |
| Cluster-40555.214354 | 1.5 | 2.56 | 0 | 0 | 3.15 | 2.04 | 0.09 | 0.08 | 0.00 | 1.17E-02 | -4.91 | 4.46E-03 | 887 |
| Cluster-40555.215348 | 2.13 | 2.49 | 0 | 0 | 2.06 | 2.3 | 0 | 0 | 0.00 | 2.04E-05 | 0.00 | 3.76E-06 | 1356 |
| Cluster-40555.215801 | 1.41 | 0.52 | 0 | 0 | 6.9 | 6.97 | 0 | 0 | 0.00 | 2.67E-02 | 0.00 | 5.23E-20 | 1450 |
| Cluster-40555.215999 | 4.75 | 3.48 | 0 | 0 | 5.12 | 4.11 | 0 | 0 | 0.00 | 1.03E-16 | 0.00 | 2.60E-21 | 2177 |
| Cluster-40555.216016 | 35.35 | 27.64 | 0 | 0 | 9.29 | 8.49 | 0 | 0 | 0.00 | 1.79E-42 | 0.00 | 3.74E-16 | 989 |
| Cluster-40555.216413 | 1.71 | 0.86 | 0 | 0 | 1.44 | 2.71 | 0 | 0.33 | 0.00 | 4.00E-04 | -3.54 | 2.40E-02 | 1802 |
| Cluster-40555.216543 | 5.7 | 4.99 | 0 | 0 | 2.66 | 1.55 | 0 | 0 | 0.00 | 9.70E-14 | 0.00 | 1.62E-06 | 1462 |
| Cluster-40555.216544 | 9.78 | 6.82 | 0 | 0 | 8.11 | 6.66 | 0.18 | 0.21 | 0.00 | 5.59E-05 | -5.26 | 4.42E-04 | 548 |
| Cluster-40555.216797 | 3.76 | 3.09 | 0 | 0 | 2.68 | 3.2 | 0 | 0 | 0.00 | 5.28E-18 | 0.00 | 4.01E-18 | 2750 |
| Cluster-40555.217937 | 8.54 | 11.81 | 0 | 0 | 1.99 | 2.68 | 0 | 0 | 0.00 | 2.47E-16 | 0.00 | 1.50E-06 | 1346 |

| | | | | | | | | | | | | | |
|---|---|---|---|---|---|---|---|---|---|---|---|---|---|
| Cluster-40555.218562 | 6.98 | 16.13 | 0 | 0 | 7.66 | 6.06 | 0.24 | 0.24 | 0.00 | 3.46E-03 | -4.96 | 3.40E-03 | 516 |
| Cluster-40555.219843 | 0.74 | 0.72 | 0 | 0 | 9.04 | 8.69 | 0.03 | 0.46 | 0.00 | 5.02E-04 | -5.02 | 1.40E-27 | 2891 |
| Cluster-40555.220660 | 0.56 | 0.98 | 0 | 0 | 0.86 | 0.94 | 0 | 0 | 0.00 | 3.42E-02 | 0.00 | 4.55E-03 | 1626 |
| Cluster-40555.220763 | 3.39 | 2.04 | 0 | 0 | 0.54 | 0.5 | 0 | 0 | 0.00 | 4.41E-11 | 0.00 | 1.72E-02 | 2177 |
| Cluster-40555.220975 | 1.88 | 1.41 | 0 | 0 | 1.75 | 1.16 | 0 | 0 | 0.00 | 1.63E-03 | 0.00 | 8.49E-04 | 1296 |
| Cluster-40555.221761 | 2.78 | 2.01 | 0 | 0 | 0.8 | 1.65 | 0 | 0 | 0.00 | 5.85E-09 | 0.00 | 1.78E-03 | 2011 |
| Cluster-40555.222902 | 0.75 | 1.36 | 0 | 0 | 0.8 | 0.34 | 0 | 0 | 0.00 | 3.57E-04 | 0.00 | 1.40E-02 | 2093 |
| Cluster-40555.223353 | 2.59 | 1.52 | 0 | 0 | 5.75 | 3.39 | 0 | 0 | 0.00 | 3.84E-03 | 0.00 | 9.38E-09 | 1004 |
| Cluster-40555.223612 | 2.47 | 2.03 | 0 | 0 | 1.19 | 1.88 | 0 | 0 | 0.00 | 3.73E-07 | 0.00 | 6.27E-06 | 1737 |
| Cluster-40555.225157 | 5.15 | 7.22 | 0 | 0 | 13.1 | 10.09 | 0.13 | 0.04 | 0.00 | 1.54E-13 | -6.99 | 2.11E-25 | 1416 |
| Cluster-40555.225593 | 9.04 | 5.9 | 0 | 0 | 2.5 | 2.23 | 0 | 0 | 0.00 | 4.55E-09 | 0.00 | 2.24E-03 | 831 |
| Cluster-40555.226141 | 1.77 | 1.96 | 0 | 0 | 2.21 | 2.34 | 0 | 0.29 | 0.00 | 1.00E-02 | -3.83 | 1.92E-02 | 960 |
| Cluster-40555.226507 | 7.3 | 7 | 0 | 0 | 3.17 | 4.07 | 0 | 0 | 0.00 | 6.66E-17 | 0.00 | 2.12E-10 | 1373 |
| Cluster-40555.226903 | 1.82 | 3.04 | 0 | 0 | 4.75 | 3.23 | 0 | 0.07 | 0.00 | 5.48E-04 | -6.77 | 4.78E-07 | 1031 |
| Cluster-40555.227108 | 1.27 | 0.48 | 0 | 0 | 1.19 | 1.02 | 0 | 0.16 | 0.00 | 5.10E-03 | -3.84 | 1.44E-03 | 2414 |
| Cluster-40555.22727 | 1.99 | 1.74 | 0 | 0 | 1.51 | 1.51 | 0 | 0 | 0.00 | 2.90E-02 | 0.00 | 3.52E-02 | 849 |
| Cluster-40555.227380 | 13.87 | 12.65 | 0 | 0 | 4.3 | 5.57 | 0 | 0 | 0.00 | 3.99E-49 | 0.00 | 2.44E-21 | 2334 |
| Cluster-40555.227436 | 11.36 | 10.15 | 0 | 0 | 2.89 | 3.02 | 0 | 0 | 0.00 | 3.05E-38 | 0.00 | 4.91E-14 | 2123 |
| Cluster-40555.227533 | 6.95 | 5.61 | 0 | 0 | 5.61 | 7.32 | 0.31 | 0.63 | 0.00 | 8.62E-26 | -3.71 | 2.34E-10 | 2273 |
| Cluster-40555.227933 | 1.47 | 1.72 | 0 | 0 | 0.62 | 0.69 | 0 | 0 | 0.00 | 1.70E-04 | 0.00 | 3.48E-02 | 1583 |
| Cluster-40555.228429 | 36.56 | 40.01 | 0 | 0 | 41.18 | 37.78 | 0 | 0 | 0.00 | 5.26E-04 | 0.00 | 2.11E-04 | 303 |
| Cluster-40555.228765 | 53.18 | 32.31 | 0 | 0 | 4.03 | 2.78 | 0.42 | 0 | 0.00 | 1.31E-19 | -3.94 | 1.53E-03 | 968 |
| Cluster-40555.229452 | 12.91 | 15.43 | 0 | 0 | 13.17 | 16.29 | 0 | 0 | 0.00 | 9.62E-44 | 0.00 | 3.26E-37 | 2399 |
| Cluster-40555.229521 | 0.53 | 0.34 | 0 | 0 | 1.08 | 0.6 | 0.07 | 0 | 0.00 | 4.33E-03 | -4.50 | 8.20E-05 | 3722 |
| Cluster-40555.229807 | 7.19 | 4.57 | 0 | 0 | 3.28 | 4.21 | 0 | 0.18 | 0.00 | 3.27E-06 | -5.04 | 1.46E-03 | 758 |
| Cluster-40555.230094 | 16.9 | 11.22 | 0 | 0 | 2.65 | 1.21 | 0 | 0 | 0.00 | 1.43E-15 | 0.00 | 1.85E-02 | 786 |
| Cluster-40555.230217 | 3.77 | 3.72 | 0 | 0 | 4.31 | 3.65 | 0 | 0.14 | 0.00 | 7.18E-06 | -5.70 | 4.62E-06 | 993 |
| Cluster-40555.230425 | 0.49 | 1.26 | 0 | 0 | 2.11 | 2.17 | 0 | 0 | 0.00 | 1.12E-02 | 0.00 | 3.35E-14 | 2856 |
| Cluster-40555.231371 | 2.33 | 4.74 | 0 | 0 | 2.64 | 3.64 | 0 | 0 | 0.00 | 5.08E-04 | 0.00 | 1.72E-06 | 1057 |
| Cluster-40555.231893 | 4.39 | 9.59 | 0 | 0 | 16.78 | 17.24 | 0.18 | 0 | 0.00 | 3.45E-03 | -7.54 | 2.80E-11 | 551 |
| Cluster-40555.233146 | 2.2 | 2.05 | 0 | 0 | 0.47 | 0.32 | 0 | 0 | 0.00 | 1.14E-12 | 0.00 | 1.01E-02 | 3037 |
| Cluster-40555.233944 | 0.88 | 1.94 | 0 | 0 | 1.04 | 1.25 | 0 | 0 | 0.00 | 6.28E-03 | 0.00 | 8.09E-03 | 1229 |

| | | | | | | | | | | | | | |
|---|---|---|---|---|---|---|---|---|---|---|---|---|---|
| Cluster-40555.234772 | 0.96 | 1.48 | 0 | 0 | 3.64 | 3.74 | 0.04 | 0 | 0.00 | 4.36E-03 | -7.43 | 2.01E-10 | 1462 |
| Cluster-40555.234806 | 4.78 | 3.06 | 0 | 0 | 1.68 | 0.84 | 0.12 | 0 | 0.00 | 5.17E-17 | -4.30 | 2.06E-03 | 2712 |
| Cluster-40555.235968 | 0.66 | 0.49 | 0 | 0 | 2.11 | 2.17 | 0.21 | 0 | 0.00 | 2.04E-03 | -4.30 | 7.79E-09 | 3080 |
| Cluster-40555.236846 | 10.04 | 14.61 | 0 | 0 | 3.21 | 3.96 | 0 | 0 | 0.00 | 4.05E-12 | 0.00 | 1.61E-05 | 867 |
| Cluster-40555.237203 | 2.66 | 1.93 | 0 | 0 | 0.97 | 0.93 | 0 | 0 | 0.00 | 9.83E-07 | 0.00 | 2.58E-03 | 1642 |
| Cluster-40555.237211 | 2.11 | 1.21 | 0 | 0 | 1.16 | 0.97 | 0 | 0 | 0.00 | 1.40E-03 | 0.00 | 8.45E-03 | 1306 |
| Cluster-40555.241204 | 1.64 | 2.12 | 0 | 0 | 1.5 | 0.98 | 0 | 0 | 0.00 | 1.14E-02 | 0.00 | 4.87E-02 | 939 |
| Cluster-40555.241418 | 1.13 | 1.37 | 0 | 0 | 1.38 | 1.26 | 0 | 0 | 0.00 | 1.94E-02 | 0.00 | 4.03E-03 | 1203 |
| Cluster-40555.241796 | 0.61 | 0.98 | 0 | 0 | 1.9 | 1.74 | 0.24 | 0 | 0.00 | 7.38E-05 | -3.85 | 7.60E-07 | 3119 |
| Cluster-40555.241917 | 0.63 | 0.63 | 0 | 0 | 2.45 | 3.81 | 0.3 | 0.36 | 0.00 | 7.97E-03 | -3.18 | 8.26E-04 | 2408 |
| Cluster-40555.242120 | 1.03 | 2.09 | 0 | 0 | 2.91 | 1.66 | 0 | 0.19 | 0.00 | 6.33E-04 | -4.56 | 1.58E-05 | 1700 |
| Cluster-40555.242302 | 3.3 | 3.09 | 0 | 0 | 5.16 | 6.54 | 0 | 0 | 0.00 | 1.89E-14 | 0.00 | 2.38E-25 | 2368 |
| Cluster-40555.243606 | 4.26 | 3.68 | 0 | 0 | 5.63 | 5.45 | 0 | 0 | 0.00 | 6.28E-09 | 0.00 | 1.09E-14 | 1305 |
| Cluster-40555.244071 | 0.95 | 0.65 | 0 | 0 | 0.82 | 0.81 | 0.1 | 0 | 0.00 | 5.28E-04 | -3.91 | 6.57E-03 | 2644 |
| Cluster-40555.245757 | 1.8 | 2.33 | 0 | 0 | 3.2 | 2.24 | 0 | 0.51 | 0.00 | 4.21E-04 | -3.33 | 3.28E-03 | 1192 |
| Cluster-40555.246418 | 1.56 | 3.17 | 0 | 0 | 1.79 | 2.17 | 0 | 0 | 0.00 | 5.01E-04 | 0.00 | 6.67E-06 | 1413 |
| Cluster-40555.246788 | 0.69 | 0.27 | 0 | 0 | 2.39 | 2.42 | 0.04 | 0.13 | 0.00 | 1.39E-02 | -4.70 | 2.00E-10 | 2914 |
| Cluster-40555.249075 | 0.42 | 0.2 | 0 | 0 | 2.59 | 0.98 | 0 | 0 | 0.00 | 2.00E-04 | 0.00 | 1.49E-04 | 7019 |
| Cluster-40555.253885 | 3.63 | 3.7 | 0 | 0.02 | 3.34 | 4.5 | 0 | 0.75 | 0.00 | 1.37E-03 | -3.22 | 2.73E-02 | 730 |
| Cluster-40555.260612 | 0.98 | 0.66 | 0 | 0 | 0.83 | 0.67 | 0 | 0.24 | 0.00 | 3.51E-05 | -2.52 | 4.25E-02 | 3245 |
| Cluster-40555.262728 | 0.62 | 0.27 | 0 | 0 | 1.87 | 0.94 | 0.04 | 0.04 | 0.00 | 4.13E-03 | -4.88 | 3.80E-04 | 3671 |
| Cluster-40555.264480 | 21.21 | 17.05 | 0 | 0 | 7.6 | 10.44 | 0.39 | 0 | 0.00 | 1.35E-05 | -5.53 | 1.95E-02 | 399 |
| Cluster-40555.265014 | 4.81 | 6.17 | 0 | 0 | 5.9 | 3.33 | 0.2 | 0.27 | 0.00 | 3.73E-21 | -4.19 | 1.68E-05 | 2087 |
| Cluster-40555.266253 | 0.41 | 0.24 | 0 | 0 | 0.61 | 0.35 | 0 | 0 | 0.00 | 6.68E-03 | 0.00 | 2.23E-05 | 4586 |
| Cluster-40555.268018 | 7.96 | 11.58 | 0 | 0 | 6.27 | 3.78 | 0.17 | 0.32 | 0.00 | 6.99E-11 | -4.16 | 1.27E-04 | 847 |
| Cluster-40555.269395 | 0.5 | 0.57 | 0 | 0 | 5.37 | 6.04 | 0.11 | 0 | 0.00 | 1.12E-02 | -6.74 | 3.08E-25 | 2664 |
| Cluster-40555.269551 | 0.8 | 0.45 | 0 | 0 | 0.8 | 1.32 | 0 | 0.26 | 0.00 | 3.42E-03 | -3.00 | 1.82E-02 | 2728 |
| Cluster-40555.269603 | 4.51 | 3.09 | 0 | 0 | 2.85 | 1.27 | 0 | 0 | 0.00 | 3.16E-05 | 0.00 | 2.47E-03 | 911 |
| Cluster-40555.271463 | 9.25 | 8.47 | 0 | 0 | 1.95 | 1.11 | 0 | 0 | 0.00 | 4.30E-11 | 0.00 | 3.71E-02 | 842 |
| Cluster-40555.272474 | 2.41 | 1.56 | 0 | 0 | 3.59 | 2.81 | 0.08 | 0.04 | 0.00 | 4.35E-05 | -5.60 | 8.79E-08 | 1475 |
| Cluster-40555.274669 | 3.83 | 10.54 | 0 | 0 | 3.52 | 4.91 | 0 | 0 | 0.00 | 6.02E-03 | 0.00 | 1.11E-06 | 875 |
| Cluster-40555.279411 | 1.37 | 1.15 | 0 | 0 | 2.02 | 1.92 | 0 | 0 | 0.00 | 2.59E-03 | 0.00 | 1.64E-06 | 1531 |

| | | | | | | | | | | | | | |
|---|---|---|---|---|---|---|---|---|---|---|---|---|---|
| Cluster-40555.301671 | 0.9 | 1.09 | 0 | 0 | 0.92 | 0.78 | 0 | 0 | 0.00 | 2.68E-02 | 0.00 | 2.32E-02 | 1379 |
| Cluster-40555.302122 | 1.02 | 0.9 | 0 | 0 | 6.53 | 6.48 | 1.59 | 1.75 | 0.00 | 6.17E-04 | -1.90 | 9.89E-05 | 2212 |
| Cluster-40555.303079 | 0.97 | 4.26 | 0 | 0 | 6.56 | 3.19 | 0 | 0 | 0.00 | 4.88E-02 | 0.00 | 1.58E-07 | 2629 |
| Cluster-40555.303167 | 1.78 | 2.58 | 0 | 0 | 1.32 | 1.18 | 0 | 0 | 0.00 | 9.32E-04 | 0.00 | 1.74E-02 | 1068 |
| Cluster-40555.303771 | 0.46 | 0.89 | 0 | 0 | 0.49 | 0.17 | 0 | 0 | 0.00 | 3.03E-04 | 0.00 | 4.29E-02 | 3754 |
| Cluster-40555.303844 | 5.03 | 4.8 | 0 | 0 | 5.47 | 6.71 | 0 | 0 | 0.00 | 4.48E-02 | 0.00 | 5.18E-03 | 467 |
| Cluster-40555.305478 | 1.67 | 1.77 | 0 | 0 | 2.17 | 2.86 | 0.06 | 0.01 | 0.00 | 3.52E-12 | -6.00 | 6.25E-15 | 3543 |
| Cluster-40555.305851 | 5.15 | 4.38 | 0 | 0 | 4.73 | 4.11 | 0 | 0 | 0.00 | 2.16E-07 | 0.00 | 3.41E-08 | 980 |
| Cluster-40555.307692 | 0.47 | 0.47 | 0 | 0 | 1.18 | 1.56 | 0 | 0 | 0.00 | 1.71E-02 | 0.00 | 4.01E-09 | 2807 |
| Cluster-40555.308189 | 1.4 | 1.41 | 0 | 0 | 2.35 | 1.93 | 0.25 | 0 | 0.00 | 2.12E-02 | -4.08 | 9.36E-03 | 1086 |
| Cluster-40555.309807 | 14.61 | 11.49 | 0 | 0 | 4.04 | 3.83 | 0 | 0 | 0.00 | 5.20E-07 | 0.00 | 2.30E-02 | 513 |
| Cluster-40555.310238 | 1.4 | 1.49 | 0 | 0 | 1.34 | 0.82 | 0 | 0 | 0.00 | 8.93E-03 | 0.00 | 2.08E-02 | 1183 |
| Cluster-40555.312244 | 3.63 | 2.63 | 0 | 0 | 7.08 | 5.9 | 0.11 | 0 | 0.00 | 2.16E-06 | -6.91 | 1.20E-13 | 1222 |
| Cluster-40555.314940 | 2.09 | 1.75 | 0 | 0 | 1.51 | 1.51 | 0 | 0 | 0.00 | 1.33E-03 | 0.00 | 1.90E-03 | 1162 |
| Cluster-40555.34229 | 1.54 | 1.51 | 0 | 0 | 5.3 | 3.96 | 0.15 | 0 | 0.00 | 4.48E-02 | -5.88 | 1.96E-06 | 924 |
| Cluster-40555.35605 | 1.02 | 2.32 | 0 | 0 | 1.98 | 1.16 | 0 | 0.15 | 0.00 | 1.11E-02 | -4.41 | 3.99E-02 | 1021 |
| Cluster-40555.39866 | 3 | 2.71 | 0 | 0 | 2.67 | 2.82 | 0 | 0 | 0.00 | 1.10E-05 | 0.00 | 1.39E-06 | 1193 |
| Cluster-40555.40009 | 1.02 | 0.93 | 0 | 0 | 1.42 | 0.55 | 0 | 0 | 0.00 | 2.90E-02 | 0.00 | 1.29E-02 | 1381 |
| Cluster-40555.40616 | 4.25 | 3.99 | 0 | 0 | 5.15 | 5.74 | 0 | 0 | 0.00 | 1.52E-02 | 0.00 | 4.98E-04 | 560 |
| Cluster-40555.40845 | 5.41 | 3.66 | 0 | 0 | 2.56 | 2.78 | 0 | 0 | 0.00 | 1.15E-05 | 0.00 | 5.81E-04 | 851 |
| Cluster-40555.40981 | 1.86 | 0.98 | 0 | 0 | 2.76 | 2.16 | 0 | 0.07 | 0.00 | 4.25E-02 | -6.00 | 6.45E-04 | 994 |
| Cluster-40555.41429 | 1.18 | 2.7 | 0 | 0 | 0.98 | 1.64 | 0 | 0 | 0.00 | 5.51E-03 | 0.00 | 5.80E-03 | 1151 |
| Cluster-40555.41754 | 1.96 | 1.09 | 0 | 0 | 3.1 | 3.44 | 0.23 | 0 | 0.00 | 5.32E-03 | -4.92 | 1.12E-05 | 1217 |
| Cluster-40555.41854 | 1.87 | 1.64 | 0 | 0 | 1.35 | 1.63 | 0 | 0 | 0.00 | 3.22E-02 | 0.00 | 3.00E-02 | 872 |
| Cluster-40555.42255 | 10.77 | 10.03 | 0 | 0 | 2.99 | 4.38 | 0 | 0.2 | 0.00 | 1.08E-11 | -5.10 | 9.78E-04 | 783 |
| Cluster-40555.42538 | 7.05 | 6.39 | 0 | 0 | 7.57 | 7.5 | 0 | 0 | 0.00 | 4.32E-07 | 0.00 | 1.90E-09 | 752 |
| Cluster-40555.42569 | 2.03 | 3.11 | 0 | 0 | 4.72 | 3.46 | 0.17 | 0.09 | 0.00 | 1.66E-07 | -4.88 | 2.25E-09 | 1600 |
| Cluster-40555.42716 | 138.82 | 115.52 | 0 | 0 | 27.39 | 14.08 | 0 | 0 | 0.00 | 1.68E-16 | 0.00 | 7.73E-03 | 318 |
| Cluster-40555.42817 | 7.92 | 3.3 | 0 | 0 | 1.91 | 4.28 | 0 | 0 | 0.00 | 1.02E-03 | 0.00 | 1.82E-02 | 636 |
| Cluster-40555.43105 | 8.08 | 14.63 | 0 | 0 | 18.79 | 24.92 | 0 | 0 | 0.00 | 2.60E-03 | 0.00 | 3.00E-07 | 397 |
| Cluster-40555.43526 | 2.83 | 2.12 | 0 | 0 | 1.4 | 1.65 | 0.12 | 0.06 | 0.00 | 1.55E-04 | -4.06 | 3.83E-02 | 1131 |
| Cluster-40555.43855 | 31.44 | 31.09 | 0 | 0 | 8.05 | 5.4 | 0 | 0 | 0.00 | 1.40E-11 | 0.00 | 1.01E-02 | 433 |

| | | | | | | | | | | | | | |
|---|---|---|---|---|---|---|---|---|---|---|---|---|---|
| Cluster-40555.44140 | 7.29 | 10.64 | 0 | 0 | 15.13 | 10.72 | 0.1 | 0 | 0.00 | 8.63E-07 | -7.40 | 2.65E-10 | 609 |
| Cluster-40555.44286 | 13.32 | 12.93 | 0 | 0 | 2.43 | 1.8 | 0 | 0 | 0.00 | 1.12E-24 | 0.00 | 6.75E-05 | 1173 |
| Cluster-40555.44715 | 6.76 | 3.15 | 0 | 0 | 1.53 | 1.24 | 0 | 0 | 0.00 | 4.63E-06 | 0.00 | 2.52E-04 | 1486 |
| Cluster-40555.45144 | 7.75 | 6.27 | 0 | 0 | 5.35 | 5.82 | 0.18 | 0.23 | 0.00 | 1.06E-18 | -4.71 | 2.63E-11 | 1528 |
| Cluster-40555.45172 | 2.89 | 1.79 | 0 | 0 | 1.55 | 1.48 | 0 | 0 | 0.00 | 3.10E-03 | 0.00 | 1.72E-02 | 931 |
| Cluster-40555.46078 | 9.87 | 11.64 | 0 | 0 | 3.41 | 2.44 | 0 | 0 | 0.00 | 5.09E-15 | 0.00 | 7.87E-05 | 910 |
| Cluster-40555.46193 | 3.51 | 3.42 | 0 | 0 | 2.35 | 1.67 | 0 | 0 | 0.00 | 7.57E-15 | 0.00 | 2.05E-10 | 2261 |
| Cluster-40555.51561 | 1.64 | 1.87 | 0 | 0 | 2.69 | 1.82 | 0 | 0.12 | 0.00 | 2.42E-03 | -5.18 | 5.19E-04 | 1165 |
| Cluster-40555.55485 | 1.82 | 0.76 | 0 | 0 | 4.03 | 3.9 | 0 | 0.47 | 0.00 | 3.74E-02 | -4.07 | 4.62E-05 | 1093 |
| Cluster-40555.56969 | 3.65 | 5.3 | 0 | 0 | 1.74 | 1.81 | 0 | 0.08 | 0.00 | 7.69E-07 | -5.49 | 1.35E-02 | 963 |
| Cluster-40555.57414 | 1.07 | 0.93 | 0 | 0 | 3.81 | 3.54 | 0 | 0 | 0.00 | 5.62E-03 | 0.00 | 1.49E-13 | 1703 |
| Cluster-40555.62826 | 4.37 | 4.26 | 0 | 0 | 4.39 | 4.85 | 0.25 | 0.26 | 0.00 | 9.80E-04 | -4.13 | 5.08E-03 | 673 |
| Cluster-40555.68054 | 2 | 1.29 | 0 | 0 | 3.05 | 2.29 | 0.13 | 1.27 | 0.00 | 5.43E-07 | -1.83 | 4.48E-02 | 2259 |
| Cluster-40555.69867 | 2.23 | 2.29 | 0 | 0 | 2.48 | 2.8 | 0.78 | 0.82 | 0.00 | 1.44E-09 | -1.66 | 3.66E-02 | 2211 |
| Cluster-40555.86567 | 4.85 | 2.43 | 0 | 0 | 1.98 | 0.88 | 0 | 0 | 0.00 | 1.15E-04 | 0.00 | 3.79E-02 | 879 |
| Cluster-40555.88820 | 27.54 | 30.55 | 0 | 0 | 5.49 | 7.83 | 0.23 | 0.25 | 0.00 | 7.42E-17 | -5.04 | 2.12E-03 | 533 |
| Cluster-40555.88955 | 86.69 | 77.73 | 0 | 0 | 37.27 | 36.22 | 0 | 0 | 0.00 | 2.43E-48 | 0.00 | 6.93E-27 | 590 |
| Cluster-40555.99532 | 2.32 | 1.9 | 0 | 0 | 1.47 | 1.06 | 0 | 0 | 0.00 | 8.56E-06 | 0.00 | 3.45E-04 | 1553 |
| Cluster-40555.99856 | 18.62 | 29.56 | 0 | 0 | 7.52 | 6.37 | 0.37 | 0 | 0.00 | 9.97E-10 | -5.19 | 2.05E-05 | 674 |
| Cluster-85789.0 | 1.09 | 1.5 | 0 | 0 | 1.2 | 0.84 | 0 | 0 | 0.00 | 2.43E-02 | 0.00 | 3.63E-02 | 1127 |
| Cluster-40555.189337 | 52.08 | 44.62 | 73.51 | 63.47 | 22.22 | 18.59 | 52.1 | 58.82 | 0.64 | 4.74E-02 | 1.52 | 4.08E-06 | 2025 |
| Cluster-40555.189804 | 504.02 | 492.1 | 744.17 | 682.87 | 160.11 | 148.96 | 473.83 | 433.75 | 0.66 | 2.07E-02 | 1.62 | 1.44E-07 | 776 |
| Cluster-40555.184462 | 15.54 | 12.8 | 19.93 | 20.76 | 8.16 | 6.7 | 14.58 | 17.04 | 0.67 | 4.57E-02 | 1.16 | 2.29E-03 | 3404 |
| Cluster-40555.181744 | 26.74 | 24.81 | 37.76 | 38.62 | 0.7 | 0.33 | 4.93 | 8.23 | 0.71 | 4.17E-02 | 3.77 | 5.99E-04 | 1598 |
| Cluster-40555.193141 | 30.53 | 33.06 | 49.38 | 46.27 | 2.13 | 2.04 | 12.16 | 15.77 | 0.72 | 2.08E-02 | 2.82 | 9.83E-09 | 2194 |
| Cluster-40555.185214 | 27.2 | 23.36 | 41.22 | 35.52 | 20.34 | 17.23 | 36.1 | 44.59 | 0.74 | 1.67E-02 | 1.18 | 9.15E-04 | 2821 |
| Cluster-40555.180687 | 20.7 | 23.82 | 32.65 | 35.37 | 5.64 | 7.37 | 23.64 | 22.62 | 0.75 | 1.70E-02 | 1.89 | 3.33E-08 | 2294 |
| Cluster-40555.195747 | 25.22 | 33.31 | 43.69 | 46.17 | 11.9 | 11.83 | 37.02 | 40.43 | 0.75 | 1.19E-02 | 1.77 | 4.29E-08 | 2435 |
| Cluster-40555.190889 | 140.62 | 139.81 | 215.39 | 218.96 | 121.93 | 135.81 | 227.76 | 256.67 | 0.78 | 1.39E-02 | 0.97 | 1.19E-02 | 566 |
| Cluster-40555.203157 | 21.74 | 14.94 | 28.92 | 27.84 | 16.85 | 10.77 | 38.7 | 33.71 | 0.78 | 1.64E-02 | 1.47 | 1.84E-05 | 2247 |
| Cluster-40555.193024 | 20.44 | 23.89 | 34.63 | 35.58 | 17.28 | 14.01 | 30.26 | 27.53 | 0.80 | 2.01E-02 | 0.95 | 2.78E-02 | 1575 |
| Cluster-40555.196653 | 113.54 | 110.15 | 175.24 | 178.27 | 54.07 | 54.93 | 105.54 | 90.62 | 0.80 | 9.17E-03 | 0.91 | 4.01E-02 | 644 |

| | | | | | | | | | | | | | |
|---|---|---|---|---|---|---|---|---|---|---|---|---|---|
| Cluster-40555.186934 | 28.43 | 17.07 | 37.93 | 33.77 | 25.38 | 18.4 | 37.13 | 46.73 | 0.81 | 1.06E-02 | 1.02 | 2.37E-02 | 2448 |
| Cluster-40555.197699 | 19.2 | 14.29 | 27.95 | 25.02 | 18.27 | 16.31 | 52.16 | 46.76 | 0.81 | 2.26E-02 | 1.58 | 1.65E-06 | 1878 |
| Cluster-40555.187270 | 224.56 | 314.34 | 470.64 | 394.57 | 6.79 | 2.76 | 56.53 | 61.32 | 0.81 | 2.26E-02 | 3.71 | 3.60E-28 | 1552 |
| Cluster-40555.183468 | 38.89 | 44.03 | 68.4 | 64.93 | 15.69 | 12.59 | 28.22 | 27.6 | 0.82 | 7.46E-03 | 1.05 | 1.43E-02 | 1426 |
| Cluster-40555.210670 | 10.98 | 12.3 | 18.06 | 19.4 | 3.21 | 3.04 | 7.72 | 11.13 | 0.82 | 2.78E-02 | 1.67 | 1.22E-02 | 1992 |
| Cluster-40555.181381 | 22.78 | 27.37 | 39.9 | 41.26 | 7.2 | 6.86 | 17.69 | 23.68 | 0.83 | 1.06E-02 | 1.63 | 1.11E-03 | 1587 |
| Cluster-40555.187635 | 38.19 | 44.1 | 64.61 | 68.44 | 10.21 | 12.07 | 49.4 | 42.78 | 0.83 | 3.51E-02 | 2.11 | 3.76E-07 | 684 |
| Cluster-40555.183573 | 187.14 | 209.45 | 319.29 | 327.55 | 113.71 | 88.45 | 184.17 | 239.95 | 0.85 | 2.09E-03 | 1.14 | 1.08E-02 | 664 |
| Cluster-40555.194134 | 17.13 | 11.9 | 22.9 | 24.27 | 12.45 | 8.12 | 23.85 | 16.91 | 0.85 | 4.99E-02 | 1.06 | 3.81E-02 | 1132 |
| Cluster-40555.181061 | 25.82 | 24.72 | 41.04 | 41.56 | 12.24 | 10.79 | 33.63 | 30.88 | 0.85 | 2.41E-02 | 1.55 | 1.29E-04 | 1010 |
| Cluster-40555.192911 | 9.18 | 5.92 | 11.85 | 12.72 | 3.22 | 3.36 | 6.51 | 8.1 | 0.85 | 2.35E-02 | 1.22 | 1.17E-02 | 2536 |
| Cluster-40555.197837 | 7.28 | 5.42 | 10.2 | 10.71 | 5.48 | 4.54 | 16.1 | 15.96 | 0.87 | 4.57E-02 | 1.75 | 3.00E-06 | 2234 |
| Cluster-40555.198076 | 26.91 | 28.63 | 46.21 | 46 | 12.21 | 11.48 | 26.7 | 26.72 | 0.87 | 4.36E-03 | 1.24 | 1.34E-03 | 1697 |
| Cluster-40555.201804 | 35.57 | 34.6 | 54.26 | 61.8 | 13.99 | 9.8 | 30.02 | 24.49 | 0.87 | 5.21E-03 | 1.27 | 3.18E-03 | 1147 |
| Cluster-40555.187179 | 28.23 | 38.33 | 60.98 | 50.52 | 7.16 | 7.7 | 15.93 | 18.41 | 0.87 | 1.32E-02 | 1.28 | 1.07E-02 | 1110 |
| Cluster-40555.187274 | 12.54 | 12.7 | 21.54 | 20.86 | 4.36 | 4.08 | 14.82 | 11.84 | 0.89 | 4.25E-03 | 1.73 | 1.52E-06 | 3146 |
| Cluster-40555.164806 | 5.76 | 5.73 | 10.05 | 9.28 | 3.72 | 2.42 | 5.68 | 6.65 | 0.89 | 2.48E-02 | 1.09 | 3.35E-02 | 3166 |
| Cluster-40555.187726 | 14.2 | 14.87 | 26.48 | 22.56 | 2.36 | 3.66 | 13.54 | 9.59 | 0.89 | 4.10E-02 | 1.99 | 6.01E-05 | 1223 |
| Cluster-40555.195343 | 8.69 | 7.92 | 13.7 | 14.24 | 7.69 | 7.41 | 13.86 | 15.57 | 0.89 | 4.54E-02 | 1.03 | 3.52E-02 | 1646 |
| Cluster-40555.170989 | 19.18 | 24.96 | 41.38 | 33.65 | 0.96 | 0.16 | 9.13 | 9.19 | 0.90 | 2.40E-02 | 4.13 | 8.56E-11 | 1107 |
| Cluster-40555.185337 | 9.53 | 5.31 | 12.85 | 12.2 | 1.62 | 1.12 | 3.65 | 4.68 | 0.91 | 2.07E-02 | 1.68 | 4.60E-03 | 2268 |
| Cluster-40555.209839 | 30.77 | 27.04 | 44.55 | 53.71 | 22.84 | 14.01 | 72.54 | 70.85 | 0.92 | 4.00E-02 | 2.03 | 2.27E-07 | 619 |
| Cluster-40555.183046 | 20.56 | 29.95 | 40.34 | 46.62 | 15.26 | 14.46 | 30.63 | 37.99 | 0.92 | 2.05E-02 | 1.27 | 4.41E-03 | 870 |
| Cluster-40555.186595 | 23.07 | 25 | 40.92 | 42.17 | 17.83 | 13.85 | 26.83 | 30.91 | 0.93 | 1.71E-03 | 0.94 | 2.70E-02 | 1905 |
| Cluster-40555.192366 | 16.86 | 21.66 | 32.74 | 34.04 | 6.64 | 8.46 | 14.37 | 17.89 | 0.93 | 2.10E-03 | 1.16 | 5.56E-03 | 2171 |
| Cluster-40555.208027 | 35.1 | 23.08 | 50.26 | 49.45 | 13.55 | 12.78 | 34.14 | 30.01 | 0.93 | 1.69E-03 | 1.35 | 2.93E-04 | 1526 |
| Cluster-40555.194100 | 7.27 | 4.5 | 10.4 | 9.81 | 4.04 | 2.51 | 6.45 | 6.33 | 0.93 | 1.10E-02 | 1.04 | 3.94E-02 | 3274 |
| Cluster-40555.172185 | 10.25 | 13.47 | 22.16 | 19.19 | 2.76 | 2.62 | 8.47 | 10.54 | 0.93 | 3.48E-02 | 1.89 | 3.21E-04 | 1344 |
| Cluster-40555.189802 | 5.38 | 5.08 | 9.4 | 8.77 | 3.55 | 3.94 | 9.87 | 6.44 | 0.94 | 1.69E-02 | 1.18 | 3.89E-02 | 3225 |
| Cluster-40555.163607 | 95.78 | 103.02 | 175.61 | 170.89 | 46.35 | 42.99 | 79.2 | 83.05 | 0.94 | 1.01E-03 | 0.93 | 3.43E-02 | 750 |
| Cluster-40555.199618 | 9.85 | 9.51 | 14.74 | 19 | 3.76 | 3.43 | 15.51 | 13.44 | 0.94 | 9.58E-03 | 2.08 | 9.04E-08 | 1978 |
| Cluster-40555.186475 | 11.97 | 11.77 | 21.82 | 19.86 | 9.75 | 7.97 | 15.66 | 17.5 | 0.95 | 1.26E-02 | 0.98 | 4.71E-02 | 1664 |

| | | | | | | | | | | | | | |
|---|---|---|---|---|---|---|---|---|---|---|---|---|---|
| Cluster-40555.188605 | 18.19 | 20.44 | 33.42 | 34.47 | 22.38 | 23.1 | 35.98 | 40.59 | 0.95 | 7.69E-04 | 0.82 | 4.70E-02 | 2748 |
| Cluster-40555.199735 | 18.86 | 13.32 | 27.92 | 28.39 | 9.39 | 6.61 | 18.65 | 18.51 | 0.96 | 1.32E-03 | 1.29 | 8.38E-04 | 2336 |
| Cluster-40555.183351 | 21.33 | 19.34 | 36.33 | 35.45 | 15.86 | 13.97 | 33.4 | 35.99 | 0.96 | 1.18E-03 | 1.29 | 2.93E-04 | 2081 |
| Cluster-40555.181865 | 10.92 | 12.42 | 21.18 | 20.24 | 4.14 | 3.47 | 21.18 | 18.37 | 0.96 | 2.35E-02 | 2.44 | 2.18E-09 | 1311 |
| Cluster-40555.193686 | 135.68 | 84.57 | 200.95 | 183.14 | 72.96 | 55.67 | 119.21 | 143.67 | 0.97 | 2.82E-03 | 1.10 | 1.20E-02 | 493 |
| Cluster-40555.191192 | 18 | 16.33 | 28.28 | 33.11 | 7.65 | 8.21 | 31.43 | 26.64 | 0.98 | 1.67E-02 | 1.93 | 3.98E-06 | 918 |
| Cluster-40555.169422 | 5.31 | 5.63 | 9.33 | 10.34 | 2.64 | 4.17 | 8.32 | 10.59 | 0.99 | 2.33E-02 | 1.53 | 1.69E-03 | 2154 |
| Cluster-40555.180897 | 24.23 | 19.51 | 39.71 | 39.12 | 8.78 | 8.72 | 22.73 | 22.63 | 0.99 | 1.90E-03 | 1.44 | 3.94E-04 | 1373 |
| Cluster-40555.188209 | 3224.13 | 1982.69 | 4822.02 | 4531.28 | 68.91 | 46.91 | 2940.17 | 4547.36 | 1.00 | 1.44E-05 | 6.09 | 1.35E-10 | 739 |
| Cluster-40555.173553 | 4.89 | 3.61 | 8.98 | 6.51 | 1.54 | 1.2 | 5.07 | 4.26 | 1.01 | 4.89E-02 | 1.84 | 6.95E-04 | 2286 |
| Cluster-40555.184105 | 7.89 | 8.93 | 16.49 | 14.4 | 3.66 | 3.75 | 9.79 | 8.48 | 1.01 | 2.19E-02 | 1.37 | 9.27E-03 | 1561 |
| Cluster-40555.189087 | 35.87 | 36.07 | 67.18 | 65.19 | 6.76 | 4.47 | 26.91 | 27.24 | 1.02 | 3.61E-03 | 2.34 | 6.28E-07 | 763 |
| Cluster-40555.192094 | 24.88 | 20.57 | 41.38 | 42.18 | 4.63 | 6.15 | 25.35 | 20.8 | 1.02 | 5.88E-04 | 2.15 | 4.26E-09 | 1596 |
| Cluster-40555.189625 | 109.57 | 97.83 | 172.77 | 207.98 | 71.41 | 59.27 | 161.96 | 136.81 | 1.02 | 1.00E-04 | 1.26 | 3.46E-04 | 745 |
| Cluster-40555.190864 | 60.93 | 56.11 | 114.01 | 102.5 | 14.99 | 15.56 | 35.1 | 46.04 | 1.03 | 1.80E-04 | 1.48 | 1.18E-03 | 1195 |
| Cluster-40555.177097 | 62.42 | 35 | 89.66 | 88.47 | 42.2 | 31.6 | 65 | 69.76 | 1.03 | 5.44E-04 | 0.94 | 3.20E-02 | 862 |
| Cluster-40555.185090 | 46.38 | 51.06 | 92.23 | 88.76 | 17.06 | 19.58 | 39.24 | 38.57 | 1.03 | 2.55E-04 | 1.15 | 3.95E-03 | 1176 |
| Cluster-40555.212871 | 3.42 | 4.3 | 7.85 | 6.57 | 0.76 | 2.5 | 7.26 | 6.52 | 1.03 | 3.23E-02 | 2.11 | 7.37E-07 | 2616 |
| Cluster-40555.170583 | 6.25 | 3.11 | 8.22 | 9.19 | 1.69 | 1.32 | 5.88 | 4.96 | 1.05 | 3.32E-02 | 1.91 | 1.08E-03 | 1732 |
| Cluster-40555.198339 | 7.77 | 8.45 | 15.97 | 14.74 | 1.8 | 1.97 | 5.55 | 6.09 | 1.06 | 4.05E-03 | 1.69 | 9.35E-04 | 2177 |
| Cluster-40555.216227 | 3.89 | 2.21 | 5.01 | 6.37 | 2.08 | 0.99 | 3.64 | 3.65 | 1.06 | 2.86E-02 | 1.33 | 3.71E-02 | 2661 |
| Cluster-40555.191445 | 24.85 | 18.34 | 42.67 | 38.78 | 35.59 | 34.11 | 63.61 | 53.74 | 1.06 | 7.05E-05 | 0.82 | 3.81E-02 | 2932 |
| Cluster-40555.173528 | 8.18 | 8.21 | 16.48 | 14.66 | 2.25 | 2.5 | 8.28 | 6.98 | 1.06 | 1.07E-02 | 1.75 | 7.38E-04 | 1641 |
| Cluster-40555.188894 | 13.29 | 13.99 | 26.47 | 25.56 | 4.1 | 2.75 | 16.54 | 14.58 | 1.07 | 1.00E-03 | 2.26 | 8.06E-09 | 1850 |
| Cluster-40555.188440 | 88.2 | 83.48 | 152.24 | 174.6 | 18.92 | 19.61 | 34.85 | 48.25 | 1.07 | 6.90E-06 | 1.18 | 4.63E-02 | 1764 |
| Cluster-40555.213218 | 1.53 | 2.06 | 3.74 | 3.21 | 0 | 0 | 4.21 | 2.39 | 1.08 | 2.20E-02 | Inf | 9.84E-13 | 5129 |
| Cluster-40555.191188 | 30.52 | 43.21 | 69.01 | 73.4 | 1.02 | 1 | 31 | 28.54 | 1.08 | 3.68E-03 | 4.98 | 1.86E-17 | 632 |
| Cluster-40555.213274 | 55.11 | 100.32 | 166.58 | 137.25 | 0.46 | 0.16 | 5.71 | 11.41 | 1.09 | 3.63E-02 | 4.88 | 4.21E-03 | 574 |
| Cluster-40555.193057 | 18.21 | 21.87 | 38.85 | 39.01 | 12.54 | 18.53 | 54.24 | 49.2 | 1.09 | 1.68E-03 | 1.79 | 2.63E-07 | 1096 |
| Cluster-40555.207626 | 5.66 | 5.73 | 11.91 | 10.17 | 0 | 0 | 8.96 | 5.89 | 1.09 | 4.02E-02 | Inf | 4.44E-18 | 1441 |
| Cluster-40555.184164 | 148.14 | 255.87 | 437.35 | 354.77 | 42.18 | 37.06 | 85.1 | 115.96 | 1.09 | 2.34E-02 | 1.41 | 5.59E-03 | 650 |
| Cluster-40555.188682 | 21.76 | 31.41 | 51.54 | 52.62 | 16.14 | 16.45 | 32.1 | 30.54 | 1.10 | 7.85E-05 | 1.01 | 1.13E-02 | 1825 |

| | | | | | | | | | | | | | |
|---|---|---|---|---|---|---|---|---|---|---|---|---|---|
| Cluster-40555.202151 | 34.98 | 46.68 | 83.28 | 76.82 | 3.39 | 5.04 | 19.71 | 27.9 | 1.10 | 6.63E-05 | 2.57 | 9.58E-05 | 1437 |
| Cluster-40555.182119 | 11.54 | 8.95 | 18.59 | 21.24 | 10.17 | 7.4 | 15.9 | 17.15 | 1.11 | 1.19E-03 | 0.99 | 4.08E-02 | 1761 |
| Cluster-40555.154825 | 22.12 | 17.25 | 39.32 | 37.43 | 3.4 | 2.87 | 10.5 | 12.98 | 1.11 | 3.53E-03 | 1.97 | 1.18E-03 | 896 |
| Cluster-40555.178573 | 11.1 | 12.33 | 21.88 | 24.08 | 7.08 | 10.37 | 36.24 | 35.62 | 1.11 | 1.10E-02 | 2.10 | 3.29E-08 | 1005 |
| Cluster-40555.177935 | 58.97 | 57 | 113.99 | 112.65 | 20.86 | 25.89 | 132.29 | 133.93 | 1.11 | 1.40E-03 | 2.57 | 8.75E-13 | 537 |
| Cluster-40555.208366 | 12.4 | 11.38 | 23.75 | 23.27 | 4.89 | 4.33 | 16.19 | 15.13 | 1.12 | 2.73E-03 | 1.83 | 3.00E-05 | 1335 |
| Cluster-40555.190364 | 8.28 | 6.28 | 14.41 | 14.29 | 5.61 | 4.14 | 11.92 | 11.9 | 1.12 | 9.84E-04 | 1.36 | 9.11E-04 | 2486 |
| Cluster-40555.175625 | 6.68 | 6.31 | 13.98 | 11.91 | 11.05 | 9.6 | 21.58 | 22.29 | 1.13 | 4.71E-02 | 1.16 | 1.46E-02 | 1132 |
| Cluster-40555.193620 | 12.06 | 8.88 | 20.81 | 20.75 | 8.23 | 8.98 | 23.74 | 22.29 | 1.13 | 3.34E-04 | 1.48 | 3.31E-05 | 2215 |
| Cluster-40555.176839 | 66.62 | 54.88 | 119.48 | 121.74 | 35.71 | 32.86 | 66.81 | 71.13 | 1.14 | 7.44E-05 | 1.07 | 1.04E-02 | 762 |
| Cluster-40555.188503 | 9.2 | 8.07 | 18.78 | 15.79 | 8.07 | 6.29 | 14.18 | 13.86 | 1.14 | 1.09E-03 | 1.04 | 2.23E-02 | 2172 |
| Cluster-40555.186899 | 21.82 | 23.25 | 47.43 | 42.86 | 2.47 | 1.13 | 10.51 | 9.58 | 1.14 | 6.55E-03 | 2.56 | 1.17E-03 | 704 |
| Cluster-40555.190181 | 4.94 | 4.75 | 8.72 | 10.7 | 8.83 | 6.65 | 18.63 | 18.8 | 1.14 | 4.83E-02 | 1.34 | 3.31E-03 | 1245 |
| Cluster-40555.190355 | 7.19 | 10.3 | 17.3 | 17.95 | 10.03 | 9.96 | 24.44 | 23.06 | 1.14 | 4.17E-04 | 1.31 | 2.49E-04 | 2519 |
| Cluster-40555.198249 | 6.54 | 8.54 | 14.18 | 16.33 | 2.25 | 1.85 | 9.17 | 11.72 | 1.15 | 6.33E-03 | 2.42 | 5.47E-07 | 1440 |
| Cluster-40555.202019 | 6.31 | 7.7 | 14.32 | 14.05 | 3.07 | 3.27 | 6.87 | 7.77 | 1.15 | 4.27E-03 | 1.27 | 2.32E-02 | 1763 |
| Cluster-40555.194012 | 5.39 | 6.75 | 12.32 | 12.34 | 2.4 | 1.3 | 6.21 | 5.54 | 1.15 | 1.79E-04 | 1.75 | 1.95E-05 | 4131 |
| Cluster-40555.191706 | 26.32 | 25.41 | 52.98 | 51.37 | 5.77 | 5.29 | 18.66 | 16.99 | 1.15 | 6.67E-04 | 1.76 | 5.19E-04 | 901 |
| Cluster-40555.193317 | 5.32 | 3.03 | 8.34 | 8.42 | 2.63 | 2.54 | 5.51 | 7.12 | 1.16 | 6.21E-03 | 1.37 | 7.24E-03 | 2353 |
| Cluster-40555.186228 | 8.77 | 4.96 | 14.86 | 12.79 | 2.52 | 1.43 | 7.56 | 8.02 | 1.16 | 5.75E-03 | 2.06 | 6.47E-05 | 1633 |
| Cluster-40555.193052 | 7.39 | 7.83 | 13.65 | 17.17 | 0 | 0 | 0.96 | 2.37 | 1.16 | 9.20E-03 | Inf | 1.13E-02 | 1244 |
| Cluster-40555.185107 | 8.54 | 11.9 | 22.03 | 19.86 | 6.21 | 6.86 | 15.6 | 16.53 | 1.16 | 3.87E-04 | 1.36 | 4.72E-04 | 2209 |
| Cluster-40555.187683 | 424.5 | 384.39 | 729.94 | 867.1 | 43.03 | 17.22 | 107.74 | 161.07 | 1.17 | 5.21E-05 | 2.21 | 1.13E-03 | 323 |
| Cluster-40555.187323 | 38.36 | 32.49 | 69.37 | 74.91 | 26.41 | 28.12 | 61.93 | 43.58 | 1.17 | 3.20E-05 | 1.01 | 1.66E-02 | 1121 |
| Cluster-40555.191437 | 6.58 | 7.1 | 14.26 | 13.83 | 4.95 | 4.31 | 13.48 | 10.63 | 1.18 | 1.71E-03 | 1.45 | 6.18E-04 | 2051 |
| Cluster-40555.174604 | 2.77 | 3.13 | 6.38 | 5.77 | 4.36 | 3.46 | 7.63 | 7.62 | 1.18 | 2.06E-02 | 1.04 | 4.99E-02 | 2442 |
| Cluster-40555.190162 | 13.44 | 17.47 | 32.98 | 30.87 | 3.84 | 2.29 | 8.57 | 9.04 | 1.18 | 2.64E-04 | 1.61 | 2.05E-03 | 1556 |
| Cluster-40555.186755 | 32.47 | 19 | 50.77 | 54.2 | 7.32 | 9.04 | 26.81 | 27.44 | 1.18 | 3.65E-05 | 1.79 | 2.59E-06 | 1291 |
| Cluster-40555.187029 | 95.98 | 80.14 | 202.73 | 160.28 | 26.25 | 25.86 | 82.06 | 83.12 | 1.18 | 5.34E-06 | 1.73 | 2.29E-07 | 1040 |
| Cluster-40555.191955 | 21.65 | 51.64 | 78.85 | 75.14 | 58.97 | 72.55 | 105.03 | 127.81 | 1.19 | 4.16E-02 | 0.89 | 2.61E-02 | 1859 |
| Cluster-40555.168003 | 5.04 | 7.55 | 13.52 | 12.94 | 1.65 | 0.65 | 4.99 | 5.14 | 1.20 | 4.71E-03 | 2.23 | 2.41E-04 | 1710 |
| Cluster-40555.186116 | 27.23 | 32.72 | 63.04 | 62.26 | 6.91 | 8.31 | 24.69 | 33.41 | 1.20 | 1.93E-05 | 2.00 | 1.81E-04 | 1405 |

| | | | | | | | | | | | | | |
|---|---|---|---|---|---|---|---|---|---|---|---|---|---|
| Cluster-40555.180936 | 6.21 | 11.25 | 17.98 | 18.81 | 11.2 | 11.11 | 27.4 | 22.68 | 1.20 | 1.74E-03 | 1.23 | 1.13E-03 | 1949 |
| Cluster-40555.225951 | 4.05 | 1.78 | 6.65 | 5.41 | 3.1 | 3.64 | 6.68 | 6.12 | 1.20 | 1.36E-02 | 0.99 | 4.79E-02 | 3299 |
| Cluster-40555.193929 | 4.13 | 3.68 | 7.92 | 8.42 | 0.76 | 0.96 | 2.61 | 2.46 | 1.21 | 2.91E-03 | 1.62 | 2.02E-02 | 2601 |
| Cluster-40555.215546 | 44.09 | 32.81 | 80.15 | 79.77 | 27.56 | 25.17 | 62.59 | 49.33 | 1.21 | 8.05E-05 | 1.15 | 5.47E-03 | 812 |
| Cluster-40555.191389 | 117.33 | 91.19 | 221.59 | 212.32 | 28.96 | 34.63 | 101.73 | 96.81 | 1.21 | 6.33E-05 | 1.69 | 4.02E-05 | 480 |
| Cluster-40555.204319 | 3.87 | 2.41 | 6.38 | 6.73 | 2.34 | 1.44 | 8.3 | 6.46 | 1.21 | 3.45E-02 | 2.03 | 7.83E-05 | 1712 |
| Cluster-40555.173667 | 15.07 | 13.25 | 31.68 | 27.87 | 13.75 | 17.02 | 41.47 | 34.86 | 1.21 | 1.41E-02 | 1.37 | 2.60E-03 | 715 |
| Cluster-40555.190777 | 7.92 | 15.32 | 27.2 | 22.5 | 10.04 | 13.15 | 27.58 | 27.66 | 1.22 | 1.12E-02 | 1.31 | 6.43E-04 | 1494 |
| Cluster-40555.185412 | 13.95 | 13.41 | 25.81 | 27.65 | 1.67 | 1.73 | 5.4 | 5.89 | 1.22 | 1.97E-03 | 1.82 | 2.64E-02 | 1047 |
| Cluster-40555.183632 | 12.38 | 10.1 | 26.89 | 20.49 | 7.52 | 5.92 | 18.11 | 18.31 | 1.22 | 1.22E-03 | 1.51 | 4.31E-04 | 1385 |
| Cluster-40555.211671 | 11.14 | 7.74 | 22.05 | 17.7 | 7.11 | 8.34 | 18.61 | 13.22 | 1.22 | 7.98E-03 | 1.10 | 4.79E-02 | 1054 |
| Cluster-40555.195768 | 9.5 | 7.66 | 20.72 | 15.7 | 15.54 | 12.69 | 24.93 | 26.99 | 1.22 | 1.06E-03 | 0.95 | 2.94E-02 | 1738 |
| Cluster-40555.208559 | 5.52 | 4.71 | 11.82 | 9.94 | 1.89 | 0.85 | 4.41 | 4.05 | 1.23 | 1.09E-02 | 1.70 | 2.27E-02 | 1571 |
| Cluster-40555.204918 | 8.24 | 9.21 | 19.35 | 17.86 | 2.52 | 3.12 | 13.38 | 12.51 | 1.23 | 1.18E-02 | 2.27 | 1.83E-05 | 997 |
| Cluster-40555.193244 | 5.16 | 2.54 | 8.73 | 7.52 | 3.81 | 3.78 | 8.2 | 7.62 | 1.23 | 1.45E-02 | 1.13 | 4.61E-02 | 1785 |
| Cluster-40555.211342 | 2.06 | 2.58 | 5.43 | 4.57 | 0.97 | 1.44 | 3.27 | 4.14 | 1.23 | 2.95E-02 | 1.68 | 4.04E-03 | 2446 |
| Cluster-40555.199540 | 5.55 | 4.74 | 11.66 | 10.4 | 4.86 | 3.47 | 10.82 | 9.74 | 1.24 | 1.60E-04 | 1.38 | 3.75E-04 | 3578 |
| Cluster-40555.191219 | 215.4 | 216.64 | 467.31 | 459.12 | 165.22 | 135.48 | 353.07 | 388.42 | 1.24 | 1.95E-07 | 1.37 | 1.91E-05 | 839 |
| Cluster-40555.210619 | 4.66 | 4.7 | 10.34 | 9.86 | 1.29 | 0.93 | 5.01 | 5.58 | 1.25 | 2.85E-03 | 2.32 | 1.16E-05 | 2097 |
| Cluster-40555.178274 | 45.96 | 48.11 | 99.98 | 102.05 | 17.24 | 13.57 | 185 | 131.36 | 1.25 | 1.09E-03 | 3.42 | 4.71E-11 | 484 |
| Cluster-40555.191714 | 6.91 | 6.11 | 16.36 | 11.78 | 5.72 | 5.04 | 13.28 | 12.07 | 1.25 | 3.04E-02 | 1.30 | 2.78E-02 | 1026 |
| Cluster-40555.190914 | 8.94 | 10.53 | 21.93 | 20.42 | 3.27 | 4.02 | 13.67 | 12.56 | 1.25 | 1.73E-05 | 1.90 | 9.93E-08 | 3046 |
| Cluster-40555.187871 | 950.32 | 924.71 | 2013.44 | 2038.03 | 21.35 | 7.06 | 178.25 | 260.11 | 1.26 | 3.42E-08 | 4.04 | 8.91E-08 | 630 |
| Cluster-40555.200351 | 7.38 | 5.77 | 15.3 | 13.14 | 7.39 | 6.38 | 16.56 | 15.22 | 1.26 | 2.92E-03 | 1.27 | 4.29E-03 | 1554 |
| Cluster-40555.212413 | 3.26 | 2.45 | 6.93 | 5.5 | 1.21 | 1.88 | 3.79 | 3.81 | 1.26 | 1.13E-02 | 1.36 | 2.75E-02 | 2416 |
| Cluster-40555.229217 | 7.94 | 13.23 | 22.46 | 24.29 | 4.92 | 4.09 | 16.74 | 19.26 | 1.27 | 1.92E-03 | 2.07 | 5.46E-06 | 1082 |
| Cluster-40555.182336 | 3.51 | 2.32 | 6.03 | 6.69 | 2.59 | 3.56 | 7.49 | 7.17 | 1.27 | 1.94E-02 | 1.31 | 1.42E-02 | 1842 |
| Cluster-40555.187023 | 7.15 | 10.16 | 20.65 | 17.67 | 2.4 | 2.77 | 7.15 | 9.39 | 1.27 | 7.12E-04 | 1.76 | 5.47E-04 | 1615 |
| Cluster-40555.193707 | 24.45 | 14.85 | 42.2 | 43.35 | 15.96 | 12.62 | 37.78 | 36.16 | 1.27 | 9.51E-07 | 1.44 | 2.05E-05 | 2357 |
| Cluster-40555.182608 | 60.74 | 68 | 136.82 | 145.37 | 54.98 | 43.71 | 377.69 | 277.31 | 1.28 | 6.35E-04 | 2.79 | 6.88E-10 | 427 |
| Cluster-40555.186824 | 6.03 | 6.61 | 12.87 | 14.97 | 2.75 | 1.68 | 12.85 | 14.19 | 1.28 | 3.37E-02 | 2.69 | 2.57E-06 | 879 |
| Cluster-40555.178548 | 1.9 | 2.7 | 5.01 | 5.24 | 1.37 | 1.41 | 3.89 | 3.58 | 1.28 | 2.06E-02 | 1.50 | 1.71E-02 | 2285 |

| | | | | | | | | | | | | | |
|---|---|---|---|---|---|---|---|---|---|---|---|---|---|
| Cluster-40555.192166 | 16.97 | 11.93 | 30.8 | 32.67 | 11.04 | 9.91 | 24.74 | 23.63 | 1.29 | 2.05E-04 | 1.27 | 4.04E-03 | 1128 |
| Cluster-40555.189534 | 4.4 | 3.93 | 9.84 | 8.62 | 3.64 | 2.41 | 7.04 | 7.04 | 1.29 | 6.58E-04 | 1.30 | 5.35E-03 | 2865 |
| Cluster-40555.184928 | 35.63 | 28.26 | 79.06 | 62.54 | 18.55 | 14.19 | 28.74 | 35.66 | 1.29 | 2.05E-07 | 1.05 | 7.64E-03 | 3053 |
| Cluster-40555.197784 | 16.81 | 17.35 | 37.86 | 38.01 | 11.43 | 9.67 | 34.83 | 24.18 | 1.29 | 2.75E-05 | 1.55 | 3.77E-04 | 1407 |
| Cluster-40555.187796 | 88.66 | 69.09 | 170.92 | 176.92 | 51.76 | 60.74 | 154.21 | 171.55 | 1.29 | 1.16E-06 | 1.59 | 1.42E-06 | 732 |
| Cluster-40555.185950 | 10.51 | 16.58 | 34.8 | 26.25 | 12.84 | 17.42 | 89.55 | 95.1 | 1.30 | 3.26E-03 | 2.67 | 8.51E-18 | 1444 |
| Cluster-40555.195704 | 3.99 | 4.02 | 8.28 | 9.6 | 3.61 | 3.1 | 12.61 | 11.4 | 1.30 | 1.02E-02 | 1.90 | 2.26E-05 | 1548 |
| Cluster-40555.217805 | 4.5 | 5.62 | 12.06 | 10.65 | 2.71 | 3.6 | 6.41 | 8.14 | 1.30 | 1.37E-03 | 1.27 | 1.37E-02 | 2065 |
| Cluster-40555.197115 | 11.85 | 11.49 | 26.99 | 25.52 | 2.23 | 2.46 | 14.3 | 12.72 | 1.31 | 8.11E-05 | 2.58 | 1.63E-09 | 1553 |
| Cluster-40555.208625 | 15.01 | 20.53 | 40.77 | 39.57 | 14.63 | 10.54 | 41.53 | 35.48 | 1.31 | 2.00E-02 | 1.68 | 2.47E-03 | 516 |
| Cluster-40555.228688 | 4.1 | 8.15 | 14.85 | 13.1 | 7.4 | 10.1 | 19.79 | 23.4 | 1.31 | 1.91E-02 | 1.36 | 3.03E-03 | 1043 |
| Cluster-40555.193785 | 1.84 | 1.53 | 3.62 | 3.94 | 2.23 | 2.03 | 6.54 | 6.01 | 1.31 | 2.01E-02 | 1.62 | 3.57E-04 | 2776 |
| Cluster-40555.173125 | 10.84 | 11.79 | 26.08 | 25.28 | 8.97 | 7.94 | 27.04 | 23.3 | 1.32 | 1.34E-04 | 1.64 | 1.84E-05 | 1420 |
| Cluster-40555.189095 | 109.56 | 173.5 | 303.76 | 342.33 | 8.69 | 5.37 | 218.15 | 162.13 | 1.32 | 1.16E-04 | 4.82 | 1.69E-24 | 553 |
| Cluster-40555.186424 | 51.75 | 49.22 | 115.38 | 112.83 | 27.56 | 20.74 | 65.34 | 65.19 | 1.32 | 3.04E-05 | 1.50 | 3.72E-04 | 603 |
| Cluster-40555.182118 | 6.36 | 3.9 | 11.69 | 11.42 | 4.59 | 4.95 | 8.9 | 10.93 | 1.32 | 2.71E-03 | 1.12 | 4.29E-02 | 1588 |
| Cluster-40555.200347 | 18.04 | 13.73 | 40.75 | 31.35 | 20.16 | 18.92 | 39.46 | 41.95 | 1.33 | 8.88E-06 | 1.13 | 2.37E-03 | 1784 |
| Cluster-40555.214951 | 37.8 | 26.88 | 74.36 | 71.9 | 10.13 | 10.6 | 40.5 | 38.43 | 1.33 | 6.62E-07 | 1.99 | 9.04E-09 | 1378 |
| Cluster-40555.212588 | 7.51 | 7.74 | 15.27 | 19.4 | 8.85 | 6.63 | 18.65 | 23.54 | 1.33 | 3.17E-02 | 1.52 | 8.28E-03 | 715 |
| Cluster-40555.216126 | 2.86 | 4.16 | 7.18 | 8.93 | 4.11 | 6.24 | 14.61 | 12.72 | 1.33 | 2.84E-02 | 1.45 | 2.27E-03 | 1291 |
| Cluster-40555.168563 | 14.04 | 10.61 | 27.93 | 28.12 | 6.45 | 7.61 | 19.17 | 17.34 | 1.33 | 2.75E-03 | 1.43 | 1.11E-02 | 796 |
| Cluster-40555.192621 | 5.69 | 8.81 | 15.99 | 17.51 | 0.98 | 1.86 | 3.98 | 3.8 | 1.34 | 1.09E-04 | 1.50 | 2.25E-02 | 1999 |
| Cluster-40555.190001 | 32.37 | 41.81 | 86.29 | 84.65 | 36.53 | 39.52 | 61.28 | 65.83 | 1.34 | 9.53E-08 | 0.81 | 4.86E-02 | 1963 |
| Cluster-40555.181021 | 9.07 | 8.3 | 18.83 | 20.89 | 7.76 | 8.35 | 21.63 | 22.68 | 1.34 | 1.05E-05 | 1.53 | 1.61E-05 | 2389 |
| Cluster-40555.185456 | 6.92 | 7.95 | 18.09 | 16.24 | 0 | 4.18 | 20.47 | 15.44 | 1.34 | 3.73E-02 | 3.11 | 2.09E-08 | 738 |
| Cluster-40555.216482 | 3.72 | 4.29 | 8.99 | 9.49 | 3.01 | 2.5 | 6.81 | 6.06 | 1.34 | 2.19E-04 | 1.29 | 5.80E-03 | 2949 |
| Cluster-40555.190846 | 2.01 | 2.05 | 4.08 | 5.25 | 1.29 | 2.61 | 10.2 | 9.82 | 1.34 | 8.88E-03 | 2.41 | 7.17E-10 | 2555 |
| Cluster-40555.204377 | 14.21 | 20.59 | 40.68 | 40.11 | 16.01 | 14.49 | 26.2 | 29.83 | 1.34 | 7.81E-06 | 0.95 | 3.45E-02 | 1480 |
| Cluster-40555.189806 | 4.22 | 7.33 | 14.66 | 12.45 | 0.41 | 0 | 3.28 | 3.16 | 1.35 | 2.66E-03 | 4.06 | 4.38E-06 | 1487 |
| Cluster-40555.196276 | 9.77 | 8.78 | 22.47 | 20.39 | 11.1 | 6.83 | 21.81 | 16.61 | 1.35 | 6.43E-04 | 1.17 | 1.54E-02 | 1226 |
| Cluster-40555.191369 | 26.37 | 26.56 | 58.39 | 63.85 | 10.18 | 11.74 | 41.14 | 52.48 | 1.36 | 8.05E-03 | 2.15 | 1.67E-04 | 445 |
| Cluster-40555.185472 | 6.52 | 10.06 | 18.53 | 20.34 | 4.33 | 4.52 | 13.87 | 17.5 | 1.36 | 2.69E-04 | 1.90 | 7.12E-06 | 1460 |

| | | | | | | | | | | | | | |
|---|---|---|---|---|---|---|---|---|---|---|---|---|---|
| Cluster-40555.183416 | 7.07 | 7.93 | 20.18 | 14.91 | 1.41 | 1.23 | 3.19 | 4.35 | 1.36 | 9.18E-05 | 1.59 | 1.47E-02 | 2139 |
| Cluster-40555.186887 | 11.72 | 15.36 | 32.36 | 31.01 | 0 | 7.55 | 23.13 | 17.55 | 1.36 | 9.51E-04 | 2.43 | 4.88E-04 | 856 |
| Cluster-40555.210422 | 3.39 | 3.97 | 9 | 8.28 | 5.54 | 5.15 | 9.21 | 12.54 | 1.36 | 1.04E-02 | 1.10 | 4.73E-02 | 1548 |
| Cluster-40555.182723 | 10.67 | 12.15 | 28.21 | 25.48 | 6.15 | 7.38 | 13.78 | 17.12 | 1.37 | 2.02E-05 | 1.26 | 3.74E-03 | 1708 |
| Cluster-40555.180077 | 4.54 | 6.5 | 13.42 | 12.65 | 1.57 | 1.65 | 7.36 | 6.12 | 1.37 | 3.08E-04 | 2.13 | 1.11E-05 | 2043 |
| Cluster-40555.155479 | 3.24 | 3.1 | 6.15 | 8.68 | 0.85 | 0.29 | 4.8 | 3.28 | 1.38 | 9.51E-03 | 2.89 | 5.21E-05 | 1571 |
| Cluster-40555.182554 | 11.57 | 9.97 | 23.66 | 26.88 | 2.62 | 1.17 | 6.33 | 7.7 | 1.38 | 1.61E-03 | 1.98 | 2.13E-02 | 855 |
| Cluster-40555.201107 | 11.37 | 19.43 | 36.78 | 36.76 | 22.94 | 21.86 | 45.2 | 36.09 | 1.38 | 1.27E-05 | 0.92 | 2.87E-02 | 1362 |
| Cluster-40555.181071 | 5.55 | 9.15 | 17.85 | 17.25 | 2.58 | 3.48 | 16.82 | 13.54 | 1.38 | 1.01E-02 | 2.37 | 7.93E-06 | 883 |
| Cluster-40555.180530 | 18.95 | 12.73 | 39.75 | 34.85 | 22.47 | 10.15 | 45.52 | 44.6 | 1.38 | 3.65E-05 | 1.55 | 3.57E-05 | 1117 |
| Cluster-40555.193075 | 8.52 | 10.62 | 24.27 | 21.54 | 6.82 | 6.46 | 15.37 | 14.28 | 1.39 | 9.89E-05 | 1.23 | 8.99E-03 | 1475 |
| Cluster-40555.149416 | 2.23 | 0.65 | 3 | 3.72 | 1.98 | 1.27 | 5.09 | 4.7 | 1.40 | 4.89E-02 | 1.67 | 7.26E-04 | 2862 |
| Cluster-40555.183711 | 30.98 | 31.77 | 81.54 | 68.48 | 0.71 | 0.88 | 15.92 | 16.29 | 1.40 | 1.65E-04 | 4.38 | 1.05E-08 | 596 |
| Cluster-40555.177094 | 8.41 | 9.11 | 20.99 | 21.1 | 10.2 | 9.63 | 21.29 | 21.86 | 1.40 | 1.52E-04 | 1.19 | 6.57E-03 | 1383 |
| Cluster-40555.226249 | 5.31 | 2.36 | 9.96 | 8.35 | 4.99 | 3.18 | 10.06 | 9.42 | 1.41 | 7.31E-04 | 1.33 | 3.68E-03 | 2165 |
| Cluster-40555.147038 | 1.89 | 1.25 | 3.89 | 3.68 | 0.99 | 0.89 | 3.15 | 3.08 | 1.41 | 1.96E-02 | 1.80 | 4.52E-03 | 2468 |
| Cluster-40555.191355 | 1.62 | 1.08 | 3.64 | 2.9 | 1.07 | 0.89 | 4.16 | 4.36 | 1.42 | 4.55E-02 | 2.20 | 5.60E-05 | 2368 |
| Cluster-40555.200787 | 1.84 | 1.56 | 4.77 | 3.5 | 2.09 | 1.85 | 5.44 | 4.52 | 1.42 | 1.39E-03 | 1.40 | 1.25E-03 | 4275 |
| Cluster-40555.184365 | 16.86 | 21.12 | 46 | 46.51 | 1.01 | 0.95 | 5.61 | 9.82 | 1.42 | 1.18E-06 | 3.05 | 1.02E-02 | 1404 |
| Cluster-40555.188354 | 87.46 | 85.14 | 215.9 | 203.18 | 106.92 | 115.96 | 196.93 | 229.43 | 1.42 | 1.90E-09 | 1.00 | 3.92E-03 | 1518 |
| Cluster-40555.191120 | 62.48 | 28.03 | 111.75 | 105.51 | 27.63 | 16.32 | 38.27 | 36.71 | 1.42 | 1.06E-05 | 0.85 | 4.37E-02 | 2636 |
| Cluster-40555.184358 | 5.2 | 5.21 | 14 | 11.42 | 3.02 | 3.09 | 7.26 | 7 | 1.42 | 3.60E-05 | 1.29 | 5.32E-03 | 2718 |
| Cluster-40555.194127 | 40.01 | 24.6 | 76.96 | 79.02 | 31.09 | 24.88 | 65.52 | 59.38 | 1.42 | 4.94E-08 | 1.23 | 6.21E-04 | 1323 |
| Cluster-40555.175892 | 2.48 | 3.07 | 6.32 | 7.22 | 2.15 | 1.62 | 4.12 | 4.78 | 1.42 | 8.28E-04 | 1.32 | 2.16E-02 | 2632 |
| Cluster-40555.186244 | 0.7 | 0.77 | 1.82 | 1.81 | 0.04 | 0.02 | 2.25 | 2.14 | 1.43 | 7.27E-03 | 6.16 | 3.20E-24 | 6116 |
| Cluster-40555.205932 | 7.1 | 6.2 | 17.11 | 15.37 | 3.19 | 3.41 | 15.09 | 13.54 | 1.43 | 6.15E-05 | 2.18 | 2.75E-08 | 1901 |
| Cluster-40555.165431 | 1.92 | 2.36 | 5.35 | 5.23 | 2.16 | 2.25 | 4.77 | 5.97 | 1.43 | 6.23E-03 | 1.35 | 1.28E-02 | 2349 |
| Cluster-40555.192985 | 26.12 | 23.99 | 63.47 | 59.67 | 17.33 | 18.79 | 38.18 | 39.29 | 1.44 | 2.27E-08 | 1.17 | 1.20E-03 | 1988 |
| Cluster-40555.188568 | 7.64 | 9.85 | 20.85 | 22.36 | 10.46 | 4.04 | 21.44 | 26.21 | 1.44 | 2.15E-04 | 1.81 | 5.82E-04 | 1202 |
| Cluster-40555.193271 | 7.04 | 8.87 | 21.15 | 18.34 | 8.14 | 7.11 | 15.17 | 12.82 | 1.44 | 7.16E-06 | 0.94 | 4.41E-02 | 2240 |
| Cluster-40555.205532 | 5.2 | 4.66 | 12 | 12.26 | 2.5 | 2.66 | 8.16 | 7.8 | 1.44 | 7.42E-03 | 1.68 | 1.02E-02 | 1096 |
| Cluster-40555.152096 | 9.49 | 14.48 | 32.35 | 27.46 | 5.83 | 4.22 | 14.47 | 17.46 | 1.44 | 1.45E-04 | 1.75 | 3.76E-04 | 1060 |

| | | | | | | | | | | | | | |
|---|---|---|---|---|---|---|---|---|---|---|---|---|---|
| Cluster-40555.185948 | 3.94 | 6.71 | 11.17 | 15.26 | 0 | 0 | 1.88 | 2.03 | 1.44 | 1.68E-02 | Inf | 5.49E-04 | 1000 |
| Cluster-40555.206444 | 4.45 | 3.34 | 9.46 | 9.65 | 5.03 | 4.17 | 13.9 | 12.19 | 1.44 | 4.91E-03 | 1.57 | 7.60E-04 | 1407 |
| Cluster-40555.194942 | 1.01 | 2.48 | 4.6 | 4.14 | 1.22 | 1.59 | 3.88 | 4.62 | 1.44 | 3.81E-02 | 1.65 | 9.32E-03 | 1921 |
| Cluster-40555.178611 | 6.98 | 6.6 | 16.33 | 17.34 | 2.77 | 2.98 | 7.96 | 7.37 | 1.45 | 6.02E-04 | 1.47 | 2.19E-02 | 1228 |
| Cluster-40555.201168 | 23.44 | 16.16 | 53.11 | 45.56 | 20.89 | 18.95 | 41.26 | 40.68 | 1.46 | 1.75E-09 | 1.11 | 1.29E-03 | 3888 |
| Cluster-40555.186822 | 2.81 | 3.55 | 8.59 | 7.45 | 2.38 | 2.5 | 7.76 | 9.33 | 1.46 | 1.96E-04 | 1.88 | 2.88E-06 | 2909 |
| Cluster-40555.192251 | 3.42 | 1.89 | 7.13 | 6.06 | 4.1 | 1.84 | 6.85 | 7.1 | 1.46 | 2.92E-03 | 1.32 | 1.39E-02 | 2115 |
| Cluster-40555.182593 | 19.29 | 16.08 | 42.96 | 45.22 | 29.9 | 28.15 | 66.81 | 74.38 | 1.47 | 1.81E-06 | 1.35 | 1.17E-04 | 1167 |
| Cluster-40555.194318 | 26.38 | 24.85 | 65.41 | 62.86 | 3.31 | 2.46 | 16.11 | 16.29 | 1.47 | 1.14E-05 | 2.58 | 9.22E-06 | 753 |
| Cluster-40555.181643 | 13.83 | 35.17 | 62.95 | 62.3 | 34.48 | 39.64 | 81.34 | 64.25 | 1.47 | 2.25E-03 | 1.03 | 1.38E-02 | 731 |
| Cluster-40555.188901 | 181.97 | 167.09 | 458.7 | 418.14 | 247.18 | 212.57 | 493.97 | 450.62 | 1.47 | 7.55E-11 | 1.11 | 7.35E-04 | 2230 |
| Cluster-40555.202559 | 4.79 | 5.14 | 11.83 | 13.18 | 4.49 | 2.85 | 8.81 | 9.33 | 1.47 | 3.26E-03 | 1.38 | 3.02E-02 | 1173 |
| Cluster-40555.207467 | 2.75 | 3.36 | 7.12 | 8.28 | 1.08 | 0.92 | 8.01 | 4.58 | 1.47 | 1.38E-04 | 2.72 | 2.08E-03 | 2861 |
| Cluster-40555.197328 | 1.52 | 0.87 | 3.65 | 2.34 | 0.23 | 0.33 | 1.29 | 1.28 | 1.47 | 4.99E-02 | 2.29 | 2.85E-02 | 2437 |
| Cluster-40555.191744 | 61.5 | 60.91 | 151.38 | 156.4 | 67.18 | 68.05 | 150.67 | 114.49 | 1.47 | 1.93E-08 | 1.03 | 5.27E-03 | 825 |
| Cluster-40555.195764 | 7.84 | 16.3 | 30.97 | 30.57 | 5.61 | 3.57 | 16.03 | 18.88 | 1.47 | 9.92E-04 | 2.00 | 4.06E-04 | 764 |
| Cluster-40555.220960 | 2.34 | 6.91 | 11.24 | 12.48 | 2.16 | 0.79 | 6.74 | 7.74 | 1.47 | 1.40E-02 | 2.39 | 1.34E-08 | 2965 |
| Cluster-40555.211646 | 4.07 | 2.96 | 10.03 | 7.8 | 2.01 | 3.52 | 5.55 | 7 | 1.48 | 5.22E-04 | 1.24 | 3.72E-02 | 2182 |
| Cluster-40555.171288 | 11.68 | 11.64 | 30.5 | 28.75 | 6.44 | 5.94 | 23.44 | 21.39 | 1.49 | 2.02E-04 | 1.92 | 2.66E-05 | 912 |
| Cluster-40555.197209 | 2.95 | 1.93 | 6.68 | 5.72 | 3.33 | 2.64 | 6.06 | 7.25 | 1.49 | 8.06E-04 | 1.24 | 1.19E-02 | 2711 |
| Cluster-40555.186864 | 23.96 | 35.37 | 79.39 | 73.02 | 26.35 | 32.41 | 61.56 | 63.7 | 1.49 | 2.05E-05 | 1.15 | 9.38E-03 | 639 |
| Cluster-40555.185457 | 2.36 | 2.84 | 7.84 | 5.56 | 1.59 | 1.32 | 8.2 | 7.43 | 1.49 | 6.85E-03 | 2.50 | 1.71E-07 | 1871 |
| Cluster-40555.188408 | 10.31 | 9.26 | 28.77 | 21.39 | 3.8 | 4.22 | 8.64 | 6.93 | 1.49 | 3.32E-06 | 1.02 | 3.56E-02 | 2841 |
| Cluster-40555.189902 | 1.87 | 1.52 | 3.77 | 4.84 | 1.13 | 1.04 | 3.59 | 2.97 | 1.50 | 1.79E-02 | 1.66 | 2.50E-02 | 1909 |
| Cluster-40555.206298 | 6.7 | 5.54 | 14.34 | 16.89 | 1.73 | 1.62 | 4.96 | 5.89 | 1.50 | 3.22E-05 | 1.78 | 2.72E-03 | 1728 |
| Cluster-40555.170341 | 2.8 | 1.63 | 4.83 | 6.37 | 2.66 | 1.43 | 6.19 | 4.56 | 1.50 | 2.89E-03 | 1.47 | 9.56E-03 | 2102 |
| Cluster-40555.186759 | 21.22 | 29.06 | 66.47 | 63.53 | 4.18 | 7.83 | 30.48 | 30.49 | 1.50 | 4.55E-05 | 2.39 | 1.20E-06 | 635 |
| Cluster-40555.194041 | 13.03 | 20 | 44.19 | 41.63 | 41.37 | 43.04 | 89.62 | 78.62 | 1.50 | 1.43E-07 | 1.06 | 2.63E-03 | 1671 |
| Cluster-40555.191583 | 26.81 | 24.79 | 62.07 | 71.03 | 10.92 | 7.53 | 27.29 | 36.4 | 1.51 | 8.57E-06 | 1.86 | 1.90E-04 | 679 |
| Cluster-40555.181568 | 11.75 | 12.66 | 28.19 | 35.07 | 0.36 | 0 | 3.75 | 5.7 | 1.51 | 5.27E-06 | 4.78 | 2.52E-06 | 1387 |
| Cluster-40555.188344 | 32.91 | 56.11 | 110.7 | 122.4 | 20.81 | 17.64 | 36.07 | 42.12 | 1.52 | 2.22E-06 | 1.10 | 9.13E-03 | 1120 |
| Cluster-40555.191719 | 3.35 | 2.12 | 6.96 | 7.15 | 1.77 | 0.98 | 3.58 | 3.89 | 1.52 | 1.21E-03 | 1.52 | 2.75E-02 | 2063 |

| | | | | | | | | | | | | | |
|---|---|---|---|---|---|---|---|---|---|---|---|---|---|
| Cluster-40555.191629 | 12.23 | 12.67 | 32.53 | 32.12 | 0 | 2.5 | 26.93 | 25.47 | 1.52 | 7.31E-05 | 4.41 | 6.64E-21 | 916 |
| Cluster-40555.154915 | 5.26 | 7.1 | 15.9 | 16.42 | 6.88 | 8.24 | 20.36 | 21.38 | 1.52 | 3.28E-05 | 1.53 | 6.06E-05 | 1744 |
| Cluster-40555.206529 | 0.63 | 0.96 | 1.96 | 2.22 | 0.66 | 0.49 | 2.46 | 2.27 | 1.52 | 2.26E-02 | 2.12 | 2.84E-04 | 3580 |
| Cluster-40555.171479 | 12.21 | 8.57 | 26.21 | 27.6 | 13.16 | 9.45 | 29.83 | 30.89 | 1.52 | 1.93E-05 | 1.50 | 1.77E-04 | 1163 |
| Cluster-40555.222755 | 2.93 | 3.78 | 8.87 | 8.79 | 2.07 | 2.99 | 11.21 | 10.28 | 1.53 | 5.23E-04 | 2.14 | 2.44E-07 | 2001 |
| Cluster-40555.192105 | 39.24 | 30.7 | 92.9 | 89.5 | 38.23 | 42.16 | 112.1 | 90.47 | 1.53 | 9.28E-10 | 1.39 | 1.44E-05 | 1603 |
| Cluster-40555.204329 | 2.82 | 3.28 | 8.72 | 7.29 | 2.69 | 2.79 | 5.85 | 7.18 | 1.53 | 4.44E-05 | 1.32 | 3.15E-03 | 3248 |
| Cluster-40555.184294 | 26.22 | 51.97 | 117.14 | 91.27 | 10.75 | 7.85 | 68.46 | 83.22 | 1.53 | 8.82E-04 | 3.10 | 1.06E-16 | 839 |
| Cluster-40555.192155 | 20.89 | 34.86 | 76.27 | 72.12 | 33.62 | 34.66 | 99.76 | 99.64 | 1.54 | 1.75E-09 | 1.61 | 2.45E-07 | 1883 |
| Cluster-40555.191018 | 3.06 | 3.47 | 8.02 | 9.21 | 3.11 | 2.72 | 6.9 | 5.11 | 1.54 | 8.80E-06 | 1.10 | 2.23E-02 | 3349 |
| Cluster-40555.208288 | 2.59 | 1.44 | 6 | 4.54 | 0 | 0.89 | 2.83 | 3.56 | 1.54 | 1.46E-02 | 2.88 | 6.14E-05 | 1769 |
| Cluster-40555.179753 | 9.77 | 10.17 | 26.47 | 26.25 | 3.75 | 1.03 | 10.67 | 11.5 | 1.54 | 3.42E-03 | 2.33 | 4.05E-03 | 658 |
| Cluster-40555.229529 | 6.5 | 5.36 | 14.64 | 16.6 | 6.38 | 6.95 | 14.7 | 15.15 | 1.54 | 5.00E-05 | 1.22 | 7.79E-03 | 1545 |
| Cluster-40555.227825 | 1.9 | 0.74 | 3.52 | 3.35 | 1.87 | 1.96 | 4.87 | 5.53 | 1.55 | 1.72E-02 | 1.50 | 5.07E-03 | 2305 |
| Cluster-40555.194284 | 1.89 | 1.33 | 4.32 | 4.15 | 1.47 | 1.31 | 4.11 | 3.55 | 1.55 | 1.03E-02 | 1.54 | 1.60E-02 | 2173 |
| Cluster-40555.143955 | 7.31 | 11.65 | 26.24 | 24.66 | 6.67 | 4.32 | 17.15 | 23.48 | 1.55 | 1.86E-03 | 1.97 | 5.08E-04 | 737 |
| Cluster-40555.196766 | 4.31 | 5.06 | 12.82 | 12.22 | 4.26 | 4.78 | 10.26 | 11.02 | 1.55 | 9.59E-05 | 1.30 | 5.75E-03 | 1814 |
| Cluster-40555.213557 | 5 | 3.14 | 11.23 | 10.29 | 6.7 | 5.18 | 14.32 | 11.8 | 1.55 | 3.01E-04 | 1.21 | 9.47E-03 | 1723 |
| Cluster-40555.157907 | 1.1 | 1.95 | 4.04 | 4.2 | 0.29 | 0.29 | 3.19 | 3.56 | 1.56 | 1.12E-03 | 3.61 | 1.30E-11 | 3389 |
| Cluster-40555.176285 | 11.15 | 12.16 | 32.12 | 30.37 | 10.95 | 12.71 | 44.94 | 49.53 | 1.56 | 4.22E-07 | 2.06 | 3.58E-10 | 1602 |
| Cluster-40555.169274 | 3.65 | 2.69 | 6.89 | 9.98 | 2.09 | 1.32 | 7.26 | 10.41 | 1.56 | 2.57E-02 | 2.47 | 4.22E-04 | 953 |
| Cluster-40555.212555 | 4.4 | 5.79 | 13.86 | 13.68 | 3.74 | 3.07 | 10.33 | 10.54 | 1.57 | 1.98E-03 | 1.68 | 3.59E-03 | 1091 |
| Cluster-40555.197568 | 2.02 | 6.43 | 12.46 | 10.74 | 4.13 | 4.86 | 9.44 | 13.53 | 1.57 | 7.38E-03 | 1.42 | 3.37E-02 | 1214 |
| Cluster-40555.183333 | 19.71 | 11.48 | 40.73 | 41.86 | 6.82 | 8.66 | 33.08 | 43.88 | 1.57 | 4.71E-05 | 2.37 | 2.52E-06 | 736 |
| Cluster-40555.185466 | 29.66 | 24.62 | 72.48 | 73.03 | 21.81 | 20.49 | 61.1 | 50.88 | 1.57 | 2.87E-09 | 1.47 | 1.88E-05 | 1368 |
| Cluster-40555.192764 | 2.45 | 2.14 | 6.04 | 6.33 | 7.06 | 5.76 | 11.78 | 12.81 | 1.57 | 4.21E-05 | 1.01 | 1.56E-02 | 3542 |
| Cluster-40555.157008 | 3.82 | 2.93 | 10.48 | 7.66 | 0.05 | 0 | 1.21 | 0.92 | 1.57 | 9.34E-03 | 5.56 | 2.74E-02 | 1220 |
| Cluster-40555.141702 | 5.49 | 4.98 | 14.03 | 14.05 | 3.14 | 4.17 | 11 | 10.33 | 1.57 | 1.06E-02 | 1.59 | 2.42E-02 | 817 |
| Cluster-40555.195250 | 2.3 | 2.65 | 6.84 | 6.69 | 2.29 | 2.63 | 5.69 | 6.59 | 1.58 | 7.45E-03 | 1.38 | 3.10E-02 | 1528 |
| Cluster-40555.192745 | 4.63 | 4.94 | 13.65 | 12.41 | 3.91 | 4.2 | 12.83 | 9.67 | 1.58 | 7.91E-05 | 1.53 | 6.30E-04 | 1728 |
| Cluster-40555.190966 | 96.75 | 140.55 | 315.86 | 334.26 | 139.6 | 145.47 | 485.76 | 473.07 | 1.58 | 1.90E-12 | 1.81 | 6.48E-10 | 2182 |
| Cluster-40555.173292 | 7.3 | 5.27 | 17.41 | 16.51 | 3.37 | 3.05 | 10.78 | 11.63 | 1.58 | 8.97E-04 | 1.88 | 1.15E-03 | 999 |

| | | | | | | | | | | | | | |
|---|---|---|---|---|---|---|---|---|---|---|---|---|---|
| Cluster-40555.131399 | 4.09 | 2.06 | 9.19 | 7.41 | 3.08 | 2.22 | 6.73 | 7.25 | 1.58 | 2.92E-03 | 1.48 | 1.40E-02 | 1506 |
| Cluster-40555.203251 | 2.54 | 2.19 | 6.68 | 6.18 | 4.28 | 3.98 | 12.28 | 10.62 | 1.59 | 1.33E-04 | 1.54 | 5.79E-05 | 2938 |
| Cluster-40555.196733 | 11.74 | 8.6 | 26.86 | 28.38 | 11.72 | 9.1 | 22.93 | 24.52 | 1.59 | 1.57E-08 | 1.26 | 7.28E-04 | 2278 |
| Cluster-40555.185764 | 29.07 | 22.02 | 72 | 65.89 | 37.59 | 26.9 | 71 | 67.08 | 1.59 | 7.75E-04 | 1.16 | 4.08E-02 | 448 |
| Cluster-40555.193665 | 14.86 | 15.47 | 42.87 | 40.27 | 16.98 | 15.18 | 33.41 | 31.8 | 1.59 | 3.80E-06 | 1.09 | 1.87E-02 | 947 |
| Cluster-40555.188467 | 4.63 | 12.32 | 22.92 | 24.43 | 7.92 | 10.75 | 19.12 | 17.6 | 1.60 | 6.58E-04 | 1.03 | 2.29E-02 | 1557 |
| Cluster-40555.179664 | 2.73 | 2.61 | 6.45 | 8.27 | 2.06 | 1.83 | 7.76 | 7.46 | 1.60 | 1.24E-03 | 2.04 | 4.55E-05 | 1736 |
| Cluster-40555.166556 | 3.95 | 9.87 | 18.12 | 20.74 | 2.8 | 3.58 | 9.72 | 8.56 | 1.60 | 1.21E-03 | 1.58 | 2.97E-02 | 884 |
| Cluster-40555.181430 | 7.23 | 15.44 | 33.25 | 30.3 | 29.76 | 31.97 | 58.99 | 61.8 | 1.61 | 3.16E-05 | 1.03 | 1.10E-02 | 954 |
| Cluster-40555.184729 | 4.61 | 3.37 | 11.71 | 10.34 | 2.65 | 2.67 | 12.52 | 12.91 | 1.61 | 1.85E-04 | 2.32 | 3.60E-08 | 1701 |
| Cluster-40555.175867 | 1.27 | 1.77 | 4.04 | 4.47 | 1.16 | 1.89 | 4.59 | 5.22 | 1.61 | 2.00E-02 | 1.76 | 3.54E-03 | 1773 |
| Cluster-40555.188618 | 8.98 | 5.05 | 21.46 | 17.17 | 1.43 | 1.64 | 6.94 | 8.85 | 1.61 | 2.45E-06 | 2.42 | 6.07E-07 | 1782 |
| Cluster-40555.210913 | 10.01 | 7.67 | 26.68 | 22.45 | 3.49 | 3.42 | 12.98 | 10.92 | 1.61 | 1.37E-05 | 1.85 | 2.16E-04 | 1212 |
| Cluster-40555.198018 | 4.14 | 4.18 | 13.06 | 10.2 | 11.06 | 8.21 | 17.95 | 18.27 | 1.62 | 4.68E-05 | 0.98 | 2.90E-02 | 2004 |
| Cluster-40555.208475 | 7.26 | 8.43 | 22.17 | 21.71 | 8.03 | 8.99 | 23.45 | 22.11 | 1.62 | 1.57E-07 | 1.48 | 3.87E-05 | 2117 |
| Cluster-40555.147135 | 2.27 | 1.92 | 6.25 | 5.45 | 0.42 | 0.17 | 2.17 | 1.61 | 1.63 | 2.58E-02 | 2.75 | 4.21E-02 | 1306 |
| Cluster-40555.181473 | 5.66 | 6.86 | 16.87 | 18.41 | 7.79 | 10.21 | 18.83 | 13.9 | 1.63 | 3.82E-07 | 0.92 | 3.87E-02 | 2199 |
| Cluster-40555.219095 | 2.51 | 3.04 | 8.21 | 7.47 | 3.26 | 3.16 | 7.43 | 7.78 | 1.63 | 4.08E-04 | 1.32 | 9.64E-03 | 2037 |
| Cluster-40555.214013 | 36.08 | 12.54 | 67.21 | 66.8 | 4.09 | 9.82 | 36.96 | 32.68 | 1.63 | 1.75E-05 | 2.36 | 1.41E-09 | 920 |
| Cluster-40555.180855 | 3.46 | 3.06 | 9.64 | 8.76 | 2.56 | 3.07 | 10.18 | 7.28 | 1.63 | 8.25E-04 | 1.69 | 7.35E-04 | 1582 |
| Cluster-40555.199095 | 1.27 | 0.98 | 2.82 | 3.43 | 1.37 | 1.15 | 3.9 | 3.5 | 1.63 | 1.48E-03 | 1.62 | 1.34E-03 | 3497 |
| Cluster-40555.171640 | 1.75 | 1.58 | 4.24 | 5.14 | 2.17 | 1.68 | 6.64 | 5.38 | 1.63 | 4.12E-04 | 1.71 | 1.32E-04 | 2945 |
| Cluster-40555.217715 | 1.06 | 0.83 | 2.39 | 2.91 | 0.22 | 0.35 | 2.05 | 1.63 | 1.63 | 3.37E-02 | 2.76 | 1.34E-03 | 2227 |
| Cluster-40555.186404 | 1.91 | 0.88 | 4.1 | 3.68 | 1.26 | 1.38 | 3.62 | 3.94 | 1.64 | 2.31E-03 | 1.58 | 4.32E-03 | 2778 |
| Cluster-40555.133165 | 9.17 | 4.54 | 18.71 | 19.44 | 8.85 | 14.43 | 35.24 | 36.67 | 1.64 | 1.05E-03 | 1.68 | 4.88E-05 | 831 |
| Cluster-40555.168371 | 5.46 | 9.38 | 19.97 | 22.29 | 1.6 | 1.96 | 8.33 | 4.92 | 1.64 | 1.11E-03 | 1.95 | 3.50E-02 | 794 |
| Cluster-40555.192183 | 44.31 | 70.79 | 172.84 | 157.11 | 35.7 | 34.3 | 74.74 | 81.89 | 1.64 | 2.05E-08 | 1.22 | 4.37E-03 | 586 |
| Cluster-40555.189142 | 18.15 | 17.97 | 52.42 | 50.33 | 4.61 | 1.58 | 27.53 | 25.2 | 1.65 | 1.29E-10 | 3.18 | 4.52E-20 | 2252 |
| Cluster-40555.195788 | 44.5 | 84.13 | 182.63 | 187.34 | 62.88 | 52.88 | 136.94 | 183.86 | 1.65 | 2.23E-08 | 1.54 | 1.92E-03 | 939 |
| Cluster-40555.185675 | 8.9 | 6.8 | 25.01 | 19.63 | 1.65 | 1.86 | 8.59 | 10.79 | 1.65 | 1.36E-08 | 2.53 | 2.74E-09 | 2754 |
| Cluster-40555.196152 | 12.38 | 15.84 | 41.74 | 39.22 | 3.53 | 6.63 | 11.84 | 15.07 | 1.65 | 1.29E-08 | 1.46 | 1.06E-02 | 1587 |
| Cluster-40555.230195 | 0.75 | 0.81 | 2.41 | 2.03 | 1.29 | 0.84 | 2.99 | 2.69 | 1.65 | 1.67E-02 | 1.49 | 1.38E-02 | 3278 |

| | | | | | | | | | | | | | |
|---|---|---|---|---|---|---|---|---|---|---|---|---|---|
| Cluster-40555.192140 | 11.43 | 14.49 | 35 | 39.45 | 5.24 | 6.68 | 26.62 | 15.96 | 1.66 | 8.95E-08 | 1.89 | 1.29E-02 | 1286 |
| Cluster-40555.197002 | 20.45 | 22.44 | 66.46 | 56.77 | 31.84 | 27.44 | 71 | 54.71 | 1.66 | 7.99E-08 | 1.15 | 2.91E-03 | 956 |
| Cluster-40555.213221 | 1.38 | 1.26 | 3.62 | 4 | 1.98 | 1.53 | 5.04 | 5.31 | 1.66 | 6.93E-03 | 1.64 | 2.43E-03 | 2231 |
| Cluster-40555.226537 | 0.61 | 3.61 | 6.72 | 5.78 | 0.32 | 0 | 3.84 | 3.73 | 1.67 | 3.93E-02 | 4.59 | 1.81E-06 | 1219 |
| Cluster-40555.206224 | 1.49 | 3.06 | 6.12 | 7.16 | 2.87 | 5.29 | 14.11 | 14.74 | 1.67 | 1.53E-02 | 1.88 | 4.03E-05 | 1231 |
| Cluster-40555.166349 | 1.8 | 1.63 | 5.44 | 4.39 | 2.42 | 1.15 | 9.23 | 6.3 | 1.67 | 3.22E-02 | 2.18 | 8.53E-05 | 1397 |
| Cluster-40555.195162 | 2.27 | 2.47 | 7.88 | 5.83 | 2.35 | 3.05 | 6.34 | 7.01 | 1.67 | 3.54E-03 | 1.37 | 1.96E-02 | 1645 |
| Cluster-40555.227206 | 1.02 | 1.01 | 3.03 | 2.82 | 1.22 | 0.69 | 2.89 | 2.89 | 1.67 | 2.48E-02 | 1.68 | 2.00E-02 | 2253 |
| Cluster-40555.203871 | 4.4 | 8.4 | 18.32 | 19.19 | 3.79 | 2.22 | 10.84 | 13.49 | 1.67 | 1.02E-03 | 2.10 | 6.85E-04 | 853 |
| Cluster-40555.199539 | 15.42 | 23.43 | 58.62 | 55.03 | 16.1 | 15.88 | 28.7 | 31.94 | 1.68 | 5.18E-10 | 0.99 | 1.67E-02 | 1661 |
| Cluster-40555.190445 | 11.1 | 9.09 | 29.32 | 29.11 | 11.18 | 9.12 | 20.36 | 21.74 | 1.68 | 1.06E-07 | 1.13 | 1.07E-02 | 1498 |
| Cluster-40555.202381 | 11.1 | 12.57 | 34.63 | 34.41 | 14.72 | 14.93 | 33.34 | 33.7 | 1.68 | 1.49E-06 | 1.24 | 3.11E-03 | 1039 |
| Cluster-40555.152088 | 3.51 | 2.18 | 7.93 | 8.51 | 5.83 | 5.27 | 11.98 | 10.23 | 1.68 | 2.97E-05 | 1.07 | 1.97E-02 | 2364 |
| Cluster-40555.186439 | 5.85 | 4.57 | 17.41 | 12.96 | 11.02 | 9.77 | 19.95 | 19.76 | 1.68 | 3.51E-06 | 1.00 | 2.07E-02 | 1956 |
| Cluster-40555.188720 | 9.46 | 7.26 | 25.1 | 23.55 | 8.66 | 8.77 | 16.86 | 16.24 | 1.69 | 5.41E-07 | 0.99 | 4.74E-02 | 1485 |
| Cluster-40555.194333 | 1.89 | 5.98 | 13.29 | 10.31 | 1.88 | 2.67 | 8.99 | 6.82 | 1.69 | 6.49E-03 | 1.84 | 1.23E-03 | 1305 |
| Cluster-40555.173955 | 42.93 | 37.12 | 111.62 | 120.33 | 7.42 | 9.29 | 91.87 | 84.42 | 1.69 | 2.19E-05 | 3.45 | 1.80E-12 | 417 |
| Cluster-40555.117546 | 0.53 | 0.77 | 2 | 1.86 | 1.69 | 1.35 | 3.67 | 3.16 | 1.69 | 2.59E-02 | 1.24 | 3.61E-02 | 3236 |
| Cluster-40555.195743 | 5.93 | 5.41 | 16.56 | 16.61 | 7.02 | 4.46 | 12.12 | 10.37 | 1.69 | 3.67E-08 | 1.05 | 2.10E-02 | 2680 |
| Cluster-40555.222175 | 9.12 | 9.02 | 27.75 | 25.49 | 8.8 | 7.39 | 17.11 | 19.04 | 1.70 | 1.57E-04 | 1.23 | 4.66E-02 | 804 |
| Cluster-40555.184277 | 16.69 | 17.54 | 47.66 | 52.89 | 3.48 | 0.61 | 8.76 | 9.25 | 1.70 | 4.41E-06 | 2.25 | 1.04E-02 | 698 |
| Cluster-40555.184804 | 7.66 | 7.06 | 23.03 | 20.31 | 4.71 | 4.14 | 16.09 | 17.47 | 1.70 | 4.20E-05 | 2.00 | 2.24E-05 | 1062 |
| Cluster-40555.192568 | 18.39 | 28.77 | 72.53 | 68.43 | 95.74 | 111.22 | 193.35 | 259.62 | 1.71 | 4.48E-09 | 1.20 | 2.31E-02 | 1027 |
| Cluster-40555.231584 | 1.06 | 1.1 | 3.42 | 3.01 | 1.44 | 1.6 | 4.68 | 3.95 | 1.71 | 3.44E-03 | 1.56 | 2.03E-03 | 2959 |
| Cluster-40555.189418 | 43.29 | 70.64 | 172.22 | 169.02 | 21.31 | 25.37 | 93.58 | 121.03 | 1.71 | 1.68E-12 | 2.27 | 7.29E-07 | 1316 |
| Cluster-40555.217374 | 2.37 | 1.23 | 5.84 | 4.76 | 0 | 0 | 4.21 | 4.4 | 1.71 | 8.33E-03 | Inf | 5.79E-16 | 1603 |
| Cluster-40555.202508 | 4.02 | 4.64 | 14.47 | 11.48 | 4.27 | 3.39 | 7.4 | 8.72 | 1.72 | 6.96E-08 | 1.15 | 9.85E-03 | 3297 |
| Cluster-40555.176213 | 8.26 | 10.04 | 28.93 | 25.97 | 3.95 | 6.87 | 19.27 | 22.57 | 1.72 | 2.63E-08 | 2.00 | 3.68E-08 | 1802 |
| Cluster-40555.191548 | 10.36 | 10.51 | 31.18 | 31.18 | 10.05 | 9.16 | 22.52 | 20.29 | 1.72 | 5.59E-11 | 1.22 | 7.40E-04 | 3000 |
| Cluster-40555.184020 | 7.83 | 5.55 | 17.48 | 21.95 | 4.92 | 3.43 | 12.1 | 17.12 | 1.72 | 1.10E-02 | 1.90 | 1.99E-02 | 575 |
| Cluster-40555.186503 | 325.95 | 285.76 | 1119.75 | 719.31 | 5.81 | 2.58 | 57.09 | 75.41 | 1.72 | 3.80E-03 | 4.07 | 2.04E-12 | 2199 |
| Cluster-40555.193906 | 1.09 | 2.11 | 5.32 | 4.4 | 2.04 | 2.63 | 5.19 | 4.91 | 1.72 | 1.00E-03 | 1.17 | 3.71E-02 | 2538 |

| | | | | | | | | | | | | | |
|---|---|---|---|---|---|---|---|---|---|---|---|---|---|
| Cluster-40555.224019 | 8.83 | 10.65 | 26.12 | 34.53 | 7.67 | 10.21 | 30.8 | 30.33 | 1.73 | 2.03E-06 | 1.76 | 1.27E-05 | 1021 |
| Cluster-40555.211065 | 1.53 | 1.16 | 3.54 | 4.52 | 1.77 | 1.96 | 4.89 | 5.05 | 1.74 | 8.11E-03 | 1.47 | 1.73E-02 | 1860 |
| Cluster-40555.193532 | 16.03 | 19.38 | 55.14 | 52.47 | 1.39 | 0.66 | 12.57 | 14.47 | 1.74 | 3.42E-11 | 3.80 | 2.41E-19 | 1942 |
| Cluster-40555.188139 | 684.19 | 670.81 | 1844.96 | 2024.79 | 290.85 | 144.06 | 1406.85 | 1080.27 | 1.74 | 1.95E-09 | 2.53 | 2.09E-07 | 267 |
| Cluster-40555.157661 | 7.88 | 6.65 | 26.1 | 17.97 | 2.42 | 1.28 | 5.16 | 5.25 | 1.74 | 1.71E-04 | 1.57 | 2.13E-02 | 1554 |
| Cluster-40555.212818 | 9.13 | 1.21 | 16.92 | 13.62 | 16.16 | 12.96 | 28.78 | 29.4 | 1.74 | 2.11E-02 | 1.07 | 2.19E-02 | 1052 |
| Cluster-40555.134740 | 3.57 | 3.65 | 10.91 | 10.99 | 3.26 | 3.24 | 6.56 | 8.01 | 1.74 | 7.82E-05 | 1.24 | 4.05E-02 | 1599 |
| Cluster-40555.183111 | 20.09 | 32.97 | 69.49 | 91.57 | 0.63 | 0 | 8 | 20.12 | 1.74 | 7.66E-03 | 5.55 | 4.71E-02 | 341 |
| Cluster-40555.214362 | 8.77 | 4.71 | 21.57 | 19.11 | 0.64 | 0.8 | 4.75 | 4.45 | 1.75 | 1.12E-06 | 2.69 | 8.62E-05 | 1459 |
| Cluster-40555.182298 | 14.64 | 12.66 | 44.27 | 39.05 | 26.88 | 28.33 | 63.77 | 57.41 | 1.75 | 1.35E-08 | 1.20 | 9.22E-04 | 1251 |
| Cluster-40555.194476 | 2.47 | 2.2 | 7.28 | 7 | 2.77 | 2.03 | 5.89 | 6.72 | 1.75 | 1.06E-04 | 1.47 | 4.31E-03 | 2200 |
| Cluster-40555.136004 | 4.62 | 10.29 | 22.49 | 23.73 | 0.47 | 0.62 | 14.94 | 14.66 | 1.75 | 1.74E-05 | 4.79 | 1.63E-17 | 1040 |
| Cluster-40555.199522 | 4.59 | 3.67 | 12.92 | 12.29 | 0 | 0.47 | 4.88 | 5.05 | 1.76 | 5.10E-06 | 4.45 | 1.20E-12 | 1865 |
| Cluster-40555.195584 | 6.08 | 1.94 | 13.4 | 10.85 | 2.32 | 1.86 | 10.04 | 8.77 | 1.76 | 1.25E-04 | 2.24 | 1.89E-08 | 2655 |
| Cluster-40555.179559 | 26.08 | 44.52 | 101.47 | 117.62 | 26.82 | 27.01 | 49.48 | 61.25 | 1.76 | 1.83E-07 | 1.11 | 1.66E-03 | 2609 |
| Cluster-40555.190415 | 9.32 | 9.05 | 28.91 | 27.7 | 12.19 | 15.9 | 26.79 | 23.54 | 1.76 | 3.48E-11 | 0.90 | 2.17E-02 | 3060 |
| Cluster-40555.178328 | 4.41 | 6.9 | 17.34 | 17.75 | 3.29 | 5.67 | 12.18 | 12.03 | 1.76 | 7.91E-05 | 1.49 | 6.39E-03 | 1083 |
| Cluster-40555.193261 | 9.88 | 12.89 | 35.94 | 34.84 | 11.99 | 9.19 | 22.2 | 26.43 | 1.77 | 8.15E-09 | 1.27 | 1.99E-03 | 1450 |
| Cluster-40555.194920 | 2.11 | 2.53 | 7.46 | 6.95 | 6.71 | 10.56 | 20.64 | 18.15 | 1.77 | 1.93E-02 | 1.22 | 1.26E-02 | 1038 |
| Cluster-40555.190271 | 43.9 | 50.24 | 142.45 | 149.82 | 55.25 | 73.78 | 138.03 | 123.26 | 1.77 | 4.90E-15 | 1.08 | 1.06E-03 | 3080 |
| Cluster-40555.208015 | 4.31 | 7.68 | 18.05 | 19.38 | 6.48 | 7.57 | 25.98 | 24.61 | 1.77 | 1.43E-04 | 1.91 | 8.16E-06 | 960 |
| Cluster-40555.196227 | 16.13 | 13.59 | 48.49 | 43.95 | 11.94 | 8.09 | 30.87 | 35.07 | 1.78 | 3.84E-11 | 1.80 | 1.84E-07 | 1888 |
| Cluster-40555.229301 | 19.88 | 17.77 | 55.79 | 61.12 | 13.73 | 15.95 | 32.11 | 32.25 | 1.78 | 4.98E-08 | 1.18 | 1.44E-02 | 798 |
| Cluster-40555.188183 | 133.57 | 168.57 | 479.87 | 455.31 | 17.66 | 9.11 | 54.52 | 89.63 | 1.78 | 2.57E-08 | 2.48 | 4.27E-03 | 335 |
| Cluster-40555.179417 | 0.98 | 1.05 | 3.54 | 2.85 | 3.13 | 3.14 | 6.22 | 5.71 | 1.78 | 2.88E-04 | 0.99 | 3.69E-02 | 4082 |
| Cluster-40555.170415 | 6.87 | 4.25 | 15.83 | 18.73 | 1.22 | 1.63 | 6.94 | 9.35 | 1.79 | 1.46E-04 | 2.56 | 1.70E-04 | 948 |
| Cluster-40555.224382 | 0.34 | 0.33 | 0.92 | 1.16 | 0.92 | 1 | 2.08 | 2.65 | 1.79 | 2.02E-02 | 1.38 | 9.88E-03 | 5117 |
| Cluster-40555.189460 | 25.27 | 25.73 | 77.69 | 82.47 | 44.66 | 43.86 | 79.33 | 87.73 | 1.79 | 1.30E-14 | 0.99 | 5.17E-03 | 3230 |
| Cluster-40555.185293 | 4.05 | 5.61 | 16.43 | 14.24 | 4.51 | 6.01 | 18.76 | 18.63 | 1.79 | 3.13E-05 | 1.89 | 4.84E-06 | 1319 |
| Cluster-40555.197153 | 2.05 | 2.16 | 7.28 | 6.03 | 2.51 | 3.08 | 13.62 | 9.37 | 1.79 | 1.25E-03 | 2.09 | 1.21E-05 | 1692 |
| Cluster-40555.172377 | 4.19 | 4.91 | 13.01 | 15.75 | 6.97 | 5.17 | 12.62 | 11.58 | 1.80 | 1.02E-08 | 1.07 | 1.31E-02 | 2873 |
| Cluster-40555.178586 | 8.78 | 23.81 | 64.65 | 40.83 | 6.39 | 7.29 | 15.7 | 15.89 | 1.80 | 4.11E-02 | 1.27 | 2.34E-02 | 960 |

| | | | | | | | | | | | | | |
|---|---|---|---|---|---|---|---|---|---|---|---|---|---|
| Cluster-40555.196515 | 2.09 | 2.34 | 7.45 | 6.64 | 2.01 | 1.26 | 4.21 | 3.92 | 1.80 | 7.96E-06 | 1.39 | 1.28E-02 | 2895 |
| Cluster-40555.208184 | 1.34 | 1.07 | 3.14 | 4.48 | 0.78 | 0.99 | 2.74 | 3.26 | 1.81 | 1.39E-03 | 1.83 | 3.16E-03 | 2618 |
| Cluster-40555.158278 | 3.68 | 5.79 | 15.46 | 15.01 | 3.19 | 2.61 | 10.08 | 9.58 | 1.81 | 2.47E-04 | 1.83 | 2.57E-03 | 1031 |
| Cluster-40555.196428 | 1.4 | 4.44 | 10.46 | 8.53 | 1.43 | 0.94 | 3.86 | 3.13 | 1.81 | 4.34E-04 | 1.64 | 1.40E-02 | 2156 |
| Cluster-40555.170684 | 7.43 | 5.66 | 21.39 | 20.26 | 8.84 | 8.73 | 17.53 | 16.89 | 1.81 | 1.14E-10 | 1.04 | 8.72E-03 | 3045 |
| Cluster-40555.174891 | 6.44 | 7.11 | 20.71 | 22.61 | 8.26 | 8.04 | 17.04 | 19.6 | 1.81 | 1.01E-08 | 1.24 | 2.34E-03 | 1894 |
| Cluster-40555.250411 | 1.18 | 1.14 | 3.4 | 3.99 | 1.02 | 0.89 | 4.61 | 3.63 | 1.82 | 2.08E-02 | 2.19 | 1.55E-03 | 1601 |
| Cluster-40555.177086 | 1.66 | 0.59 | 3.45 | 3.61 | 2.03 | 1.99 | 5.48 | 5.6 | 1.82 | 1.28E-02 | 1.53 | 9.78E-03 | 1808 |
| Cluster-40555.182592 | 5.18 | 4.25 | 15.65 | 14.47 | 5.81 | 6.49 | 23.06 | 18.76 | 1.82 | 6.00E-06 | 1.82 | 2.59E-06 | 1478 |
| Cluster-40555.208416 | 1.61 | 1.39 | 5.58 | 4 | 1.06 | 0.89 | 2.78 | 3.34 | 1.82 | 2.18E-03 | 1.71 | 1.95E-02 | 2061 |
| Cluster-40555.188864 | 25.23 | 23.43 | 74.94 | 80.61 | 10.27 | 8.63 | 19.59 | 17.68 | 1.82 | 1.35E-12 | 1.05 | 2.71E-02 | 1442 |
| Cluster-40555.179914 | 20.08 | 12.44 | 57.61 | 45.52 | 8.02 | 4.99 | 39.61 | 37.3 | 1.82 | 1.16E-04 | 2.64 | 1.16E-06 | 512 |
| Cluster-40555.196221 | 10.33 | 9.62 | 32.7 | 31.32 | 2.02 | 1.95 | 11.48 | 9.16 | 1.82 | 3.13E-08 | 2.43 | 2.15E-06 | 1262 |
| Cluster-40555.207390 | 2.19 | 1.25 | 6.52 | 4.44 | 0.07 | 0.22 | 1.34 | 1.78 | 1.82 | 7.12E-04 | 3.47 | 3.69E-05 | 2745 |
| Cluster-40555.196267 | 12.98 | 12.16 | 41.99 | 38.77 | 19.18 | 25.03 | 47.26 | 35.09 | 1.82 | 1.36E-09 | 0.95 | 1.85E-02 | 1373 |
| Cluster-40555.157577 | 7.44 | 5.69 | 22.41 | 19.54 | 5.94 | 4.78 | 14.37 | 14.32 | 1.83 | 1.17E-05 | 1.49 | 4.45E-03 | 1068 |
| Cluster-40555.139184 | 1.71 | 2.1 | 6.03 | 6.3 | 0.67 | 1.4 | 10.15 | 8.38 | 1.83 | 3.77E-02 | 3.21 | 4.98E-07 | 967 |
| Cluster-40555.188717 | 18.23 | 32.02 | 83.43 | 80.56 | 30.34 | 33.8 | 90.57 | 77.05 | 1.83 | 6.55E-09 | 1.44 | 8.16E-05 | 732 |
| Cluster-40555.223033 | 22.48 | 28.31 | 87.15 | 77.95 | 20.73 | 15.31 | 40.29 | 43.79 | 1.83 | 3.96E-10 | 1.29 | 2.02E-03 | 893 |
| Cluster-40555.187948 | 56.54 | 49.73 | 146.11 | 184.11 | 39.02 | 27.36 | 109.68 | 95.69 | 1.83 | 5.48E-04 | 1.66 | 3.54E-02 | 311 |
| Cluster-40555.187700 | 41.44 | 45.26 | 135.65 | 145.73 | 1.94 | 1.4 | 10.74 | 20 | 1.84 | 2.17E-13 | 3.29 | 1.64E-02 | 1056 |
| Cluster-40555.185451 | 4.71 | 7.39 | 23.11 | 16.86 | 5.02 | 5.21 | 14.01 | 14.33 | 1.84 | 4.04E-05 | 1.53 | 3.84E-03 | 1035 |
| Cluster-40555.185541 | 21.81 | 25.34 | 70.85 | 83.01 | 13.46 | 14.43 | 26.16 | 29.55 | 1.84 | 7.87E-12 | 1.06 | 5.72E-03 | 2167 |
| Cluster-40555.169389 | 0.61 | 1.54 | 3.14 | 3.97 | 1.6 | 2.01 | 8.82 | 7.71 | 1.85 | 1.33E-02 | 2.25 | 1.90E-06 | 1791 |
| Cluster-40555.185722 | 15.56 | 16.34 | 48.62 | 55.77 | 26.17 | 24.65 | 58.42 | 51.99 | 1.85 | 1.63E-12 | 1.18 | 7.26E-04 | 1779 |
| Cluster-40555.200336 | 4.58 | 3.58 | 13.11 | 13.62 | 6.02 | 4.86 | 11.5 | 10.29 | 1.86 | 9.46E-08 | 1.07 | 2.36E-02 | 2211 |
| Cluster-40555.204837 | 23.99 | 29.06 | 89.06 | 86.06 | 19.46 | 16.21 | 72.22 | 73.53 | 1.86 | 2.60E-10 | 2.10 | 2.66E-09 | 828 |
| Cluster-40555.196815 | 13.05 | 13.77 | 47 | 41.51 | 15.71 | 14.7 | 37.86 | 30.12 | 1.86 | 7.45E-13 | 1.22 | 4.59E-04 | 2493 |
| Cluster-40555.218065 | 1.04 | 1.01 | 3.1 | 3.66 | 0.52 | 0.35 | 3.32 | 3.27 | 1.86 | 3.99E-02 | 2.97 | 3.81E-04 | 1450 |
| Cluster-40555.184525 | 2.72 | 1.99 | 8.1 | 7.37 | 3.42 | 2.73 | 10.92 | 8.55 | 1.86 | 1.91E-04 | 1.72 | 2.32E-04 | 1713 |
| Cluster-40555.174785 | 4.61 | 2.96 | 13.08 | 11.79 | 2.46 | 1.76 | 7.05 | 7.22 | 1.86 | 8.27E-07 | 1.83 | 2.08E-04 | 1967 |
| Cluster-40555.195488 | 4.37 | 6.74 | 20.87 | 16.24 | 3.24 | 2.88 | 9.54 | 9.18 | 1.86 | 2.08E-08 | 1.68 | 8.50E-05 | 2294 |

| | | | | | | | | | | | | | |
|---|---|---|---|---|---|---|---|---|---|---|---|---|---|
| Cluster-40555.160587 | 4.08 | 2.18 | 9.43 | 10.96 | 1.75 | 2.01 | 8.28 | 8.18 | 1.86 | 2.44E-04 | 2.20 | 1.06E-04 | 1253 |
| Cluster-40555.195500 | 1.01 | 0.91 | 3.4 | 2.9 | 0.09 | 0 | 1.54 | 1.65 | 1.87 | 1.64E-02 | 5.39 | 8.88E-06 | 1944 |
| Cluster-40555.180429 | 3.82 | 3.69 | 12.46 | 12.45 | 7.75 | 9.55 | 19.24 | 17.7 | 1.87 | 2.45E-04 | 1.15 | 2.10E-02 | 1119 |
| Cluster-40555.215460 | 5.32 | 6.14 | 18.46 | 19.72 | 5.55 | 4.86 | 16.58 | 12.49 | 1.87 | 4.16E-04 | 1.55 | 1.43E-02 | 776 |
| Cluster-40555.185640 | 76.35 | 106.73 | 308.09 | 302.99 | 57.4 | 61.02 | 99.39 | 121.58 | 1.87 | 7.18E-15 | 0.96 | 1.40E-02 | 860 |
| Cluster-40555.187831 | 41.84 | 41.71 | 137.01 | 141.16 | 44.03 | 25.43 | 116.75 | 133.88 | 1.88 | 9.14E-14 | 1.93 | 1.88E-09 | 1044 |
| Cluster-40555.231238 | 2.04 | 1.68 | 7.09 | 5.36 | 2.67 | 3.27 | 7.71 | 7.1 | 1.88 | 4.61E-05 | 1.38 | 2.47E-03 | 2501 |
| Cluster-40555.207070 | 0.44 | 0.62 | 1.71 | 1.88 | 0.22 | 0.43 | 2.62 | 2 | 1.88 | 2.55E-03 | 2.86 | 7.41E-08 | 4337 |
| Cluster-40555.204625 | 3.86 | 6.51 | 18.73 | 16.24 | 3.84 | 3.11 | 12.27 | 14.88 | 1.88 | 1.58E-06 | 2.04 | 4.22E-06 | 1440 |
| Cluster-40555.195316 | 9.44 | 5.28 | 26.86 | 21.94 | 9.69 | 6.1 | 24.48 | 21.06 | 1.88 | 8.71E-07 | 1.60 | 2.13E-04 | 1117 |
| Cluster-40555.190238 | 14.93 | 14.24 | 50.67 | 47.16 | 18.51 | 15.56 | 60.15 | 53.68 | 1.88 | 2.20E-14 | 1.81 | 4.69E-09 | 3059 |
| Cluster-40555.225505 | 1.71 | 1.4 | 5.93 | 4.54 | 1.38 | 1.22 | 5.05 | 4.85 | 1.89 | 1.75E-03 | 1.99 | 7.24E-04 | 1820 |
| Cluster-40555.188627 | 13.32 | 19.36 | 57 | 53.3 | 23.03 | 20.86 | 119.89 | 100.61 | 1.89 | 2.63E-03 | 2.38 | 2.46E-07 | 396 |
| Cluster-40555.198083 | 3.98 | 2.01 | 10.78 | 9.11 | 5.5 | 6.1 | 12.9 | 12.45 | 1.89 | 1.53E-03 | 1.19 | 4.74E-02 | 1062 |
| Cluster-40555.230590 | 1.65 | 2.58 | 6.53 | 7.8 | 7.68 | 8.41 | 20.71 | 18.76 | 1.89 | 8.32E-04 | 1.35 | 1.14E-03 | 1459 |
| Cluster-40555.193631 | 7.51 | 11.35 | 32.76 | 31.17 | 6.25 | 12.38 | 29.94 | 27.72 | 1.89 | 1.76E-02 | 1.66 | 4.70E-02 | 420 |
| Cluster-40555.180538 | 5.51 | 6.14 | 19.27 | 20.04 | 4.78 | 3.7 | 10.09 | 11.17 | 1.89 | 1.77E-10 | 1.40 | 7.15E-04 | 2600 |
| Cluster-40555.131430 | 0.55 | 0.31 | 1.72 | 1.16 | 1.48 | 2.05 | 3.66 | 3.55 | 1.90 | 3.96E-03 | 1.09 | 2.43E-02 | 5184 |
| Cluster-40555.191703 | 24.68 | 72.63 | 180.21 | 156.11 | 70.47 | 82.54 | 251.13 | 243.67 | 1.90 | 9.16E-06 | 1.75 | 1.11E-07 | 546 |
| Cluster-40555.188591 | 1.76 | 2.02 | 6.22 | 6.48 | 3.47 | 3.3 | 9.04 | 9.25 | 1.90 | 9.68E-03 | 1.50 | 1.50E-02 | 1141 |
| Cluster-40555.184506 | 1.77 | 1.19 | 4.5 | 5.53 | 7.56 | 6.25 | 14.36 | 13.28 | 1.91 | 8.45E-06 | 1.07 | 8.33E-03 | 3159 |
| Cluster-40555.177659 | 0.73 | 0.76 | 2.51 | 2.56 | 0.77 | 0.95 | 3.85 | 3.84 | 1.91 | 8.20E-03 | 2.21 | 5.54E-05 | 2527 |
| Cluster-40555.176273 | 7.75 | 6.06 | 22.98 | 24.02 | 11.45 | 11.26 | 25.63 | 23.77 | 1.91 | 1.38E-04 | 1.18 | 4.09E-02 | 720 |
| Cluster-40555.191016 | 11.92 | 9.66 | 36.79 | 37.01 | 26.07 | 21.24 | 49.56 | 47.8 | 1.92 | 3.32E-13 | 1.11 | 1.68E-03 | 2433 |
| Cluster-40555.214586 | 0.79 | 0.67 | 2.32 | 2.73 | 1.41 | 1.13 | 3.79 | 4.07 | 1.93 | 3.64E-03 | 1.70 | 1.77E-03 | 2763 |
| Cluster-40555.199538 | 6.59 | 9.01 | 27.44 | 26.95 | 3.48 | 2.28 | 25.15 | 16.69 | 1.94 | 5.84E-03 | 2.93 | 1.51E-04 | 485 |
| Cluster-40555.186904 | 3.68 | 3.01 | 11.17 | 11.9 | 2.52 | 3.02 | 13.55 | 17.31 | 1.94 | 1.92E-02 | 2.54 | 1.10E-04 | 658 |
| Cluster-40555.191639 | 1.75 | 0 | 3.05 | 2.83 | 0.41 | 0 | 2.24 | 3.07 | 1.94 | 2.09E-02 | 3.79 | 3.16E-07 | 3171 |
| Cluster-40555.190025 | 2.03 | 2.12 | 7.82 | 6.61 | 2.64 | 2.5 | 9.51 | 12.74 | 1.94 | 5.30E-04 | 2.19 | 2.88E-05 | 1541 |
| Cluster-40555.189407 | 11.49 | 12.02 | 40.02 | 42.13 | 0.55 | 0.73 | 6.88 | 5.57 | 1.94 | 1.33E-08 | 3.33 | 3.65E-05 | 938 |
| Cluster-40555.161579 | 5.01 | 11.47 | 25.19 | 33.23 | 0.76 | 0.66 | 4.47 | 5.63 | 1.95 | 6.35E-04 | 2.90 | 3.61E-02 | 640 |
| Cluster-40555.175457 | 2.13 | 2.38 | 9.51 | 6.42 | 4.72 | 4.65 | 9.81 | 9.79 | 1.95 | 1.34E-04 | 1.13 | 7.75E-03 | 3099 |

| | | | | | | | | | | | | | |
|---|---|---|---|---|---|---|---|---|---|---|---|---|---|
| Cluster-40555.181981 | 17.98 | 11.89 | 54.32 | 49.56 | 37.63 | 31.17 | 64.71 | 66.25 | 1.95 | 5.74E-11 | 1.00 | 1.18E-02 | 1143 |
| Cluster-40555.217196 | 2.5 | 2.61 | 8.99 | 8.93 | 2.93 | 2.1 | 5.77 | 5.43 | 1.95 | 8.47E-07 | 1.23 | 2.86E-02 | 2361 |
| Cluster-40555.192901 | 4.06 | 3.57 | 11.18 | 15.26 | 0.26 | 0.09 | 7.33 | 7.38 | 1.95 | 1.56E-03 | 5.43 | 4.46E-08 | 790 |
| Cluster-40555.223955 | 1.83 | 1.4 | 6.14 | 5.27 | 0.92 | 0.49 | 2.88 | 3.37 | 1.96 | 4.61E-03 | 2.20 | 1.94E-02 | 1357 |
| Cluster-40555.220572 | 1.55 | 1.58 | 4.34 | 6.66 | 0 | 0 | 1.83 | 2.53 | 1.96 | 5.89E-03 | Inf | 4.68E-10 | 1881 |
| Cluster-40555.210881 | 2.7 | 3.42 | 10.75 | 10.95 | 3.24 | 3.21 | 14.23 | 11.47 | 1.96 | 1.45E-06 | 2.06 | 3.81E-07 | 1889 |
| Cluster-40555.189006 | 14.48 | 13.87 | 49.67 | 50.33 | 6.7 | 7.54 | 27.53 | 27.21 | 1.96 | 6.64E-13 | 2.01 | 1.96E-08 | 1608 |
| Cluster-40555.179367 | 4.12 | 3.76 | 14.47 | 13.5 | 1.33 | 1.36 | 6.06 | 6.76 | 1.96 | 3.63E-08 | 2.32 | 2.30E-06 | 2064 |
| Cluster-40555.139982 | 1.73 | 1.4 | 5.47 | 5.61 | 1.1 | 1.15 | 5.16 | 4.7 | 1.97 | 1.26E-03 | 2.21 | 4.09E-04 | 1610 |
| Cluster-40555.210249 | 0.73 | 0.62 | 2.12 | 2.66 | 1.13 | 0.64 | 5.17 | 4.67 | 1.97 | 2.63E-03 | 2.55 | 6.82E-08 | 2941 |
| Cluster-40555.187566 | 140.73 | 149.73 | 547.84 | 486.25 | 318.86 | 321.36 | 882.69 | 773.22 | 1.97 | 1.98E-17 | 1.43 | 2.94E-06 | 871 |
| Cluster-40555.199537 | 10.86 | 15.51 | 50.94 | 43.68 | 11.14 | 12.58 | 29.94 | 34.63 | 1.97 | 1.45E-12 | 1.51 | 2.22E-05 | 1697 |
| Cluster-40555.208161 | 17.53 | 13.42 | 58.31 | 51.56 | 12.05 | 12.57 | 40.81 | 40.21 | 1.97 | 2.03E-11 | 1.78 | 8.77E-07 | 1156 |
| Cluster-40555.131532 | 1.27 | 2.41 | 7.34 | 5.84 | 1.66 | 0.33 | 7.45 | 7.3 | 1.97 | 3.05E-02 | 3.01 | 7.39E-05 | 914 |
| Cluster-40555.188399 | 2.26 | 1.67 | 5.88 | 8.03 | 1.7 | 1.17 | 3.66 | 4.37 | 1.98 | 7.66E-05 | 1.56 | 6.52E-03 | 2602 |
| Cluster-40555.214235 | 2.63 | 1.73 | 7.57 | 7.88 | 1.03 | 0.91 | 5.51 | 3.98 | 1.98 | 1.51E-03 | 2.36 | 2.70E-03 | 1181 |
| Cluster-40555.214300 | 4.33 | 5.05 | 17.9 | 15.87 | 2.15 | 1.61 | 8.68 | 11.62 | 1.98 | 2.75E-07 | 2.51 | 3.95E-06 | 1501 |
| Cluster-40555.183605 | 5.97 | 7.81 | 24.7 | 25.01 | 8.4 | 8.79 | 16.67 | 20.61 | 1.98 | 4.32E-09 | 1.19 | 7.85E-03 | 1459 |
| Cluster-40555.189182 | 12.34 | 16.69 | 54.09 | 50.67 | 11.37 | 13.49 | 72.85 | 75.21 | 1.98 | 3.35E-13 | 2.64 | 3.04E-17 | 1657 |
| Cluster-40555.187959 | 4.21 | 3.22 | 13 | 13.6 | 2.25 | 2.93 | 15.85 | 17.69 | 1.99 | 1.46E-04 | 2.75 | 1.05E-08 | 1006 |
| Cluster-40555.168110 | 25.5 | 29.06 | 102.25 | 94.23 | 39.48 | 37.86 | 84.09 | 67.6 | 1.99 | 5.37E-09 | 1.03 | 2.87E-02 | 562 |
| Cluster-40555.189521 | 15.99 | 13.1 | 55.42 | 48.53 | 16.54 | 26.11 | 75.69 | 71.61 | 1.99 | 1.56E-07 | 1.84 | 7.22E-07 | 676 |
| Cluster-40555.171067 | 4.74 | 3.47 | 12.25 | 17.16 | 29.38 | 26.59 | 63.44 | 51.15 | 1.99 | 4.61E-03 | 1.09 | 1.90E-02 | 639 |
| Cluster-40555.218117 | 1.96 | 2.76 | 8.65 | 8.52 | 0.92 | 0.68 | 15.95 | 14.42 | 1.99 | 1.36E-04 | 4.29 | 1.69E-20 | 1464 |
| Cluster-40555.177618 | 5.31 | 6.72 | 20.2 | 23.39 | 1.3 | 0.97 | 6.18 | 4.61 | 1.99 | 2.49E-07 | 2.31 | 1.82E-03 | 1160 |
| Cluster-40555.230603 | 1.16 | 0.86 | 4.32 | 2.95 | 0.41 | 0.37 | 1.69 | 1.92 | 2.00 | 7.92E-03 | 2.25 | 3.37E-02 | 1812 |
| Cluster-40555.203696 | 2.48 | 0 | 4.64 | 4.03 | 0.59 | 1.1 | 3.67 | 4.2 | 2.01 | 1.34E-02 | 2.26 | 1.80E-03 | 1510 |
| Cluster-40555.180100 | 2.74 | 1.71 | 7.68 | 8.48 | 2.39 | 1.94 | 7.01 | 8.22 | 2.01 | 2.37E-05 | 1.88 | 2.33E-04 | 1716 |
| Cluster-40555.190657 | 15.73 | 19.27 | 65.9 | 63.29 | 37.56 | 41.91 | 106.1 | 106.64 | 2.02 | 1.39E-16 | 1.48 | 1.63E-06 | 2653 |
| Cluster-40555.202102 | 2.2 | 1.73 | 7.14 | 7.22 | 5.34 | 3.32 | 10.58 | 11.02 | 2.02 | 9.53E-04 | 1.39 | 1.14E-02 | 1300 |
| Cluster-40555.230084 | 8.02 | 11.58 | 40.93 | 31.84 | 6.41 | 4.93 | 35.95 | 31.58 | 2.02 | 3.32E-04 | 2.64 | 3.00E-06 | 518 |
| Cluster-40555.221890 | 2.17 | 2.1 | 7.36 | 8.39 | 3.86 | 2.95 | 9.75 | 9.5 | 2.02 | 1.32E-06 | 1.57 | 2.59E-04 | 2289 |

| | | | | | | | | | | | | | |
|---|---|---|---|---|---|---|---|---|---|---|---|---|---|
| Cluster-40555.176604 | 6.95 | 4.07 | 20.11 | 20.08 | 9.49 | 8.79 | 17.6 | 18.4 | 2.02 | 2.22E-11 | 1.05 | 1.15E-02 | 2337 |
| Cluster-40555.209112 | 0.3 | 0.51 | 1.46 | 1.54 | 0.27 | 0.91 | 1.71 | 1.94 | 2.02 | 1.51E-02 | 1.67 | 1.64E-02 | 3327 |
| Cluster-40555.181509 | 7.23 | 15.93 | 41.83 | 44.81 | 29.86 | 27.48 | 50.24 | 56.76 | 2.02 | 6.37E-11 | 0.97 | 1.55E-02 | 1354 |
| Cluster-40555.186812 | 6.92 | 11.69 | 35.27 | 34.31 | 0 | 0.58 | 5.85 | 5.45 | 2.03 | 2.35E-13 | 4.30 | 2.38E-15 | 2198 |
| Cluster-40555.189645 | 14.16 | 20.64 | 64.65 | 65.44 | 24.18 | 24.05 | 39.67 | 43.07 | 2.03 | 6.65E-16 | 0.85 | 4.15E-02 | 2070 |
| Cluster-40555.168502 | 2.16 | 0.66 | 5.58 | 4.81 | 1.38 | 0.79 | 4.15 | 5.22 | 2.04 | 9.60E-05 | 2.19 | 7.31E-05 | 2178 |
| Cluster-40555.196489 | 16.4 | 11.37 | 52.03 | 51.02 | 1.91 | 0.41 | 13.33 | 11.15 | 2.04 | 5.37E-09 | 3.53 | 2.40E-07 | 766 |
| Cluster-40555.145740 | 2.36 | 2.5 | 10.11 | 8.09 | 3.7 | 2.92 | 12.59 | 13.13 | 2.04 | 5.45E-04 | 2.03 | 3.80E-05 | 1181 |
| Cluster-40555.183061 | 5.86 | 8.41 | 31.21 | 22.74 | 1.67 | 0.98 | 11.11 | 11.85 | 2.04 | 3.49E-05 | 3.19 | 1.13E-05 | 717 |
| Cluster-40555.200120 | 2.56 | 2.76 | 9.76 | 10.34 | 0.21 | 0.14 | 1.23 | 1.57 | 2.05 | 2.58E-05 | 3.16 | 4.20E-02 | 1419 |
| Cluster-40555.192399 | 0.85 | 0.41 | 2.24 | 2.43 | 0 | 0 | 2.26 | 2.05 | 2.05 | 9.28E-03 | Inf | 1.91E-12 | 2310 |
| Cluster-40555.191951 | 7.15 | 5.92 | 25.77 | 23.28 | 8.27 | 7.78 | 16.48 | 16.52 | 2.05 | 8.14E-14 | 1.11 | 4.40E-03 | 3036 |
| Cluster-40555.197095 | 7.76 | 7.63 | 32.18 | 26.02 | 10.23 | 8.92 | 17.48 | 17.82 | 2.06 | 1.68E-12 | 0.95 | 3.35E-02 | 2133 |
| Cluster-40555.186606 | 18.67 | 24.49 | 71.23 | 92.22 | 57.36 | 46.14 | 137.99 | 102.08 | 2.06 | 1.79E-06 | 1.28 | 1.49E-04 | 1119 |
| Cluster-40555.167898 | 0.84 | 1.02 | 2.95 | 4.08 | 0.42 | 0.38 | 3.29 | 3.11 | 2.06 | 1.43E-02 | 3.09 | 2.31E-04 | 1475 |
| Cluster-40555.202248 | 1.26 | 0.55 | 4.06 | 2.68 | 1.55 | 0.52 | 4.76 | 4.18 | 2.06 | 7.89E-03 | 2.22 | 3.73E-04 | 1832 |
| Cluster-40555.181386 | 5.98 | 4.72 | 21.23 | 19.35 | 5.19 | 4.46 | 22.33 | 22.37 | 2.07 | 7.18E-13 | 2.28 | 6.31E-12 | 2918 |
| Cluster-40555.185128 | 5.89 | 4.99 | 21.61 | 19.7 | 14.21 | 12.91 | 21.49 | 24.41 | 2.07 | 1.87E-14 | 0.83 | 4.74E-02 | 3878 |
| Cluster-40555.247659 | 1.91 | 0.6 | 4 | 5.28 | 0.11 | 0.58 | 3.42 | 3.12 | 2.07 | 4.23E-02 | 3.23 | 6.26E-03 | 946 |
| Cluster-40555.193463 | 6.76 | 3.61 | 19.54 | 19.55 | 1.92 | 2.3 | 12.76 | 14.19 | 2.07 | 3.41E-14 | 2.74 | 2.39E-15 | 3570 |
| Cluster-40555.189221 | 17.2 | 22.75 | 70.92 | 81.57 | 19.88 | 11.96 | 72.41 | 64.09 | 2.07 | 1.39E-11 | 2.17 | 2.05E-09 | 789 |
| Cluster-40555.139427 | 0.56 | 0.28 | 1.82 | 1.41 | 0.72 | 0.63 | 2.59 | 1.99 | 2.07 | 1.85E-02 | 1.83 | 6.44E-03 | 2918 |
| Cluster-40555.167442 | 0.57 | 0.95 | 3.21 | 2.66 | 2.78 | 3.33 | 6.97 | 7.14 | 2.08 | 1.73E-03 | 1.27 | 7.22E-03 | 2602 |
| Cluster-40555.174135 | 71.97 | 107.02 | 353.86 | 337.13 | 39.33 | 35.16 | 79.99 | 89.5 | 2.08 | 2.85E-18 | 1.25 | 6.93E-04 | 867 |
| Cluster-40555.201105 | 2.88 | 4.39 | 12.31 | 15.67 | 7.94 | 6.13 | 16.95 | 14.23 | 2.08 | 1.19E-06 | 1.22 | 3.12E-03 | 2166 |
| Cluster-40555.155655 | 1.71 | 0.88 | 5.51 | 4.39 | 1.27 | 0.98 | 3.66 | 3.86 | 2.08 | 9.84E-05 | 1.81 | 2.97E-03 | 2249 |
| Cluster-40555.203869 | 0.64 | 1.01 | 3.58 | 2.78 | 0.81 | 0.46 | 2.24 | 2.71 | 2.08 | 3.71E-03 | 2.06 | 7.61E-03 | 2175 |
| Cluster-40555.205452 | 5.36 | 5.59 | 21.11 | 21 | 4.06 | 3.26 | 12.88 | 11.68 | 2.08 | 1.00E-06 | 1.82 | 9.76E-04 | 1012 |
| Cluster-40555.205709 | 1.46 | 1.56 | 4.92 | 6.73 | 1.64 | 2.28 | 10.13 | 9.4 | 2.09 | 3.44E-05 | 2.38 | 6.62E-09 | 2265 |
| Cluster-40555.196025 | 5.44 | 3.31 | 16.77 | 16.74 | 8.07 | 4.81 | 13.63 | 16.05 | 2.09 | 1.13E-08 | 1.29 | 4.96E-03 | 1620 |
| Cluster-40555.186248 | 20.62 | 21.41 | 80.3 | 81.95 | 31.57 | 25.69 | 86.22 | 75.91 | 2.09 | 4.99E-15 | 1.57 | 2.42E-06 | 1211 |
| Cluster-40555.188471 | 38.17 | 73.53 | 207.13 | 230.47 | 0.8 | 2.99 | 183.41 | 242.26 | 2.09 | 2.35E-12 | 6.85 | 7.34E-26 | 1287 |

| | | | | | | | | | | | | | |
|---|---|---|---|---|---|---|---|---|---|---|---|---|---|
| Cluster-40555.189092 | 1973.33 | 2256.91 | 8443.02 | 8020.34 | 2021.82 | 1116.92 | 8439.08 | 7295.51 | 2.10 | 1.30E-21 | 2.40 | 1.50E-16 | 482 |
| Cluster-40555.185706 | 55.89 | 99.95 | 330.06 | 287.84 | 75.03 | 49.57 | 215.01 | 202.58 | 2.11 | 5.37E-19 | 1.82 | 4.12E-09 | 1104 |
| Cluster-40555.181148 | 7.99 | 15.2 | 42.91 | 48.81 | 11.9 | 18.5 | 58.29 | 53.91 | 2.11 | 6.51E-09 | 1.93 | 2.21E-07 | 779 |
| Cluster-40555.240176 | 7.55 | 5.66 | 26.37 | 25 | 4.17 | 4.31 | 26.25 | 23.35 | 2.11 | 1.26E-03 | 2.61 | 5.18E-05 | 520 |
| Cluster-40555.185988 | 5.53 | 7.97 | 23.55 | 29.89 | 4.49 | 3.65 | 18.9 | 21.4 | 2.11 | 4.37E-06 | 2.39 | 7.61E-06 | 741 |
| Cluster-40555.208852 | 2.19 | 1.35 | 6.18 | 7.57 | 1.72 | 1.75 | 6.22 | 5.15 | 2.11 | 1.05E-06 | 1.77 | 3.47E-04 | 2334 |
| Cluster-40555.198839 | 14.01 | 18.99 | 62.92 | 67.45 | 38.6 | 37.9 | 112.14 | 90.89 | 2.12 | 2.54E-11 | 1.47 | 2.25E-05 | 820 |
| Cluster-40555.183607 | 9.84 | 5.16 | 29.87 | 28.72 | 0.1 | 0.93 | 44.23 | 46.37 | 2.12 | 4.55E-12 | 6.48 | 4.30E-57 | 1635 |
| Cluster-40555.193747 | 111.94 | 149.89 | 532.16 | 500.41 | 285.2 | 256.44 | 505.11 | 485.08 | 2.12 | 1.07E-13 | 0.92 | 2.80E-02 | 373 |
| Cluster-40555.163279 | 2.25 | 4.32 | 15.33 | 10.97 | 11.53 | 12.04 | 26.31 | 23.65 | 2.12 | 1.65E-05 | 1.15 | 7.85E-03 | 1286 |
| Cluster-40555.190081 | 3.44 | 6.18 | 18.34 | 20.11 | 10.17 | 10.66 | 18.52 | 20.53 | 2.12 | 4.42E-13 | 0.97 | 1.59E-02 | 2844 |
| Cluster-40555.192169 | 7.37 | 6.6 | 27.82 | 27.32 | 2.79 | 3.04 | 27.01 | 24.32 | 2.13 | 5.32E-07 | 3.21 | 4.36E-12 | 839 |
| Cluster-40555.177532 | 2.49 | 5.56 | 14.6 | 18.1 | 0.51 | 0.38 | 3.96 | 4.86 | 2.13 | 2.87E-03 | 3.25 | 4.68E-02 | 609 |
| Cluster-40555.189463 | 1.51 | 1.24 | 6.02 | 4.89 | 1.73 | 2.63 | 5.06 | 4.37 | 2.13 | 1.30E-06 | 1.16 | 2.67E-02 | 3031 |
| Cluster-40555.192178 | 16.92 | 30.93 | 106.08 | 87.74 | 38.84 | 29.68 | 65.08 | 78.21 | 2.13 | 1.89E-07 | 1.13 | 4.60E-02 | 453 |
| Cluster-40555.209282 | 2.55 | 1.77 | 10.1 | 7.1 | 1.39 | 0.55 | 4.68 | 3.95 | 2.14 | 2.13E-06 | 2.24 | 2.17E-04 | 1971 |
| Cluster-40555.203810 | 2.59 | 4.35 | 11.89 | 15.99 | 1.65 | 2.45 | 11.86 | 16.17 | 2.14 | 7.59E-04 | 2.83 | 5.52E-06 | 748 |
| Cluster-40555.187418 | 7.69 | 12.32 | 38.52 | 42.04 | 1.06 | 1.59 | 9.82 | 17.79 | 2.14 | 2.10E-15 | 3.44 | 7.43E-03 | 2101 |
| Cluster-40555.242300 | 1.92 | 1.66 | 6.79 | 7.43 | 5.32 | 4.56 | 15.62 | 12.65 | 2.14 | 3.23E-02 | 1.58 | 1.76E-02 | 742 |
| Cluster-40555.198276 | 4.37 | 0 | 8.83 | 8.04 | 0.01 | 0 | 4.82 | 1.99 | 2.14 | 2.00E-03 | Inf | 2.51E-05 | 3487 |
| Cluster-40555.187285 | 4.25 | 5.15 | 16.97 | 20.58 | 1.31 | 1.37 | 8.21 | 6.72 | 2.14 | 1.61E-08 | 2.54 | 7.49E-06 | 1354 |
| Cluster-40555.194347 | 5.98 | 5.35 | 22.59 | 22.71 | 7.77 | 5.52 | 25.1 | 26.39 | 2.14 | 2.81E-12 | 2.03 | 4.70E-09 | 2077 |
| Cluster-40555.196246 | 1.04 | 0.53 | 3.46 | 2.8 | 1.54 | 0.94 | 4.26 | 2.67 | 2.14 | 4.35E-03 | 1.55 | 3.23E-02 | 1979 |
| Cluster-40555.193416 | 6.08 | 2.78 | 18.96 | 16.19 | 1.9 | 2.1 | 9.44 | 9.12 | 2.14 | 2.94E-09 | 2.28 | 1.42E-06 | 1642 |
| Cluster-40555.194078 | 7.93 | 5.09 | 26.02 | 25.56 | 0.91 | 2.02 | 14.77 | 18.42 | 2.14 | 1.73E-04 | 3.57 | 7.21E-07 | 588 |
| Cluster-40555.207287 | 1.14 | 1.41 | 5.82 | 4.52 | 1.43 | 0 | 3.72 | 3.01 | 2.15 | 1.41E-02 | 2.35 | 3.01E-02 | 1114 |
| Cluster-40555.182350 | 5.67 | 3.08 | 20.22 | 14.82 | 6.59 | 4.34 | 23.66 | 19.71 | 2.15 | 1.19E-07 | 2.06 | 5.86E-08 | 1565 |
| Cluster-40555.187309 | 35.92 | 43.02 | 148.88 | 169.73 | 0.82 | 1.32 | 33.35 | 35.21 | 2.15 | 1.50E-18 | 5.04 | 7.02E-32 | 1111 |
| Cluster-40555.192916 | 26.13 | 24.92 | 107.89 | 97.72 | 0.88 | 0.51 | 14.25 | 12.19 | 2.15 | 1.26E-15 | 4.32 | 6.48E-14 | 1017 |
| Cluster-40555.139805 | 1.41 | 1.57 | 6.12 | 5.96 | 0.6 | 0.93 | 5.54 | 3.5 | 2.15 | 1.26E-02 | 2.65 | 4.32E-03 | 976 |
| Cluster-40555.185909 | 2.96 | 2.27 | 9.89 | 11.06 | 0.58 | 0.4 | 5.4 | 5.48 | 2.15 | 4.07E-03 | 3.54 | 1.08E-03 | 753 |
| Cluster-40555.175261 | 1.09 | 0.58 | 3.23 | 3.42 | 0.05 | 0 | 2.75 | 1.71 | 2.16 | 3.63E-02 | 6.64 | 2.29E-05 | 1236 |

| | | | | | | | | | | | | | |
|---|---|---|---|---|---|---|---|---|---|---|---|---|---|
| Cluster-40555.176728 | 0.95 | 1.48 | 5.48 | 4.42 | 2.92 | 3.8 | 8.64 | 9.53 | 2.16 | 4.89E-05 | 1.50 | 5.02E-04 | 2355 |
| Cluster-40555.188740 | 11.07 | 15.24 | 52.23 | 54.81 | 11.87 | 14.22 | 28.51 | 30.11 | 2.16 | 8.54E-17 | 1.23 | 7.61E-04 | 2017 |
| Cluster-40555.174206 | 18.75 | 17.81 | 74.72 | 73.31 | 16.69 | 14.2 | 38.59 | 40.43 | 2.16 | 2.48E-18 | 1.43 | 3.22E-05 | 2047 |
| Cluster-40555.195262 | 7.58 | 8.12 | 34.9 | 28.99 | 7.42 | 3.9 | 28.11 | 24.35 | 2.16 | 5.63E-11 | 2.29 | 2.00E-09 | 1325 |
| Cluster-40555.189411 | 250.6 | 543.75 | 1551.9 | 1713.63 | 264.41 | 214.25 | 402.87 | 531.35 | 2.16 | 4.91E-12 | 1.04 | 3.44E-02 | 946 |
| Cluster-40555.195106 | 8.65 | 6.51 | 30.38 | 30.84 | 10.6 | 8.83 | 28.32 | 24.64 | 2.16 | 1.96E-12 | 1.51 | 5.35E-05 | 1577 |
| Cluster-40555.197378 | 28.84 | 32.98 | 131.51 | 118.91 | 7.92 | 5.61 | 42.47 | 36.75 | 2.16 | 4.26E-08 | 2.61 | 9.72E-05 | 418 |
| Cluster-40555.231886 | 0.66 | 0.69 | 2.81 | 2.74 | 1.51 | 1.03 | 2.97 | 3.85 | 2.16 | 5.74E-04 | 1.51 | 1.08E-02 | 2858 |
| Cluster-40555.186727 | 19.5 | 22.14 | 80.48 | 89.28 | 49.77 | 37.63 | 73.08 | 79.88 | 2.16 | 6.30E-20 | 0.88 | 2.23E-02 | 2469 |
| Cluster-40555.188423 | 2.92 | 6.57 | 20.27 | 18.93 | 7.05 | 9.61 | 14.74 | 17.53 | 2.17 | 8.72E-11 | 1.02 | 2.28E-02 | 1931 |
| Cluster-40555.180789 | 1.22 | 3.07 | 10.6 | 7.18 | 0.76 | 0 | 2.73 | 3.25 | 2.17 | 6.09E-04 | 3.11 | 1.41E-04 | 1692 |
| Cluster-40555.222900 | 0.94 | 0.78 | 3.62 | 3.38 | 0.57 | 0.78 | 2.92 | 2.35 | 2.17 | 7.70E-04 | 2.02 | 4.87E-03 | 2212 |
| Cluster-40555.204242 | 1.35 | 1.31 | 4.82 | 6.02 | 1.04 | 1.27 | 5.67 | 5.04 | 2.17 | 6.32E-03 | 2.27 | 2.76E-03 | 1119 |
| Cluster-40555.215557 | 0.61 | 0.65 | 2.03 | 3.18 | 0.65 | 0.49 | 4.24 | 3.56 | 2.18 | 4.17E-03 | 2.84 | 4.46E-09 | 3454 |
| Cluster-40555.187721 | 10.17 | 6.52 | 33.48 | 34.62 | 4.79 | 3.77 | 15.06 | 12.63 | 2.18 | 3.84E-10 | 1.76 | 7.49E-04 | 1033 |
| Cluster-40555.189842 | 1084.16 | 1478.03 | 4746.68 | 5799.45 | 3036.04 | 2225.88 | 5739.15 | 5138.08 | 2.18 | 5.43E-10 | 1.11 | 7.26E-04 | 477 |
| Cluster-40555.181846 | 1.35 | 1.84 | 6.2 | 6.9 | 0.32 | 0.76 | 2.4 | 3.71 | 2.18 | 2.03E-04 | 2.57 | 6.09E-03 | 1487 |
| Cluster-40555.186202 | 39.03 | 44.06 | 180.32 | 163.22 | 58.65 | 51.57 | 203.98 | 174.98 | 2.18 | 4.09E-21 | 1.85 | 5.87E-10 | 2136 |
| Cluster-40555.211874 | 0.66 | 0.81 | 2.99 | 3.05 | 3.18 | 2.69 | 9.31 | 10.69 | 2.18 | 9.02E-06 | 1.84 | 3.24E-07 | 4066 |
| Cluster-40555.194257 | 3.88 | 5.11 | 16.8 | 20.43 | 1.49 | 3.32 | 25.27 | 24.84 | 2.19 | 4.64E-09 | 3.42 | 5.33E-19 | 1416 |
| Cluster-40555.213175 | 7.68 | 6.65 | 28.56 | 30.51 | 13.4 | 13.91 | 36.81 | 35.26 | 2.19 | 6.06E-14 | 1.46 | 2.17E-05 | 1929 |
| Cluster-40555.216017 | 12.25 | 24.52 | 83.78 | 70.71 | 0 | 0 | 14.26 | 15.2 | 2.19 | 9.58E-13 | Inf | 1.41E-22 | 853 |
| Cluster-40555.210110 | 1.7 | 1.84 | 7.19 | 7.4 | 1.49 | 0.62 | 4.41 | 4.66 | 2.19 | 4.42E-05 | 2.21 | 1.11E-03 | 1574 |
| Cluster-40555.134878 | 1.67 | 0.44 | 4.76 | 3.75 | 0.85 | 0.49 | 8.69 | 7.09 | 2.19 | 2.82E-02 | 3.62 | 6.10E-08 | 1074 |
| Cluster-40555.189546 | 243.32 | 255.62 | 959.51 | 1102.68 | 51.5 | 39.68 | 198.26 | 145.39 | 2.19 | 2.60E-19 | 1.98 | 1.12E-07 | 791 |
| Cluster-40555.198022 | 1.73 | 1.44 | 6.96 | 6.12 | 3.16 | 2.18 | 7.33 | 7.5 | 2.20 | 2.41E-03 | 1.54 | 2.72E-02 | 1129 |
| Cluster-40555.213035 | 2.82 | 3.08 | 12.38 | 12.38 | 3.01 | 4.08 | 11.92 | 8.79 | 2.20 | 9.43E-06 | 1.60 | 3.65E-03 | 1168 |
| Cluster-40555.205570 | 7.34 | 0 | 16.41 | 13.13 | 3.62 | 6.72 | 17.36 | 19.98 | 2.21 | 2.86E-03 | 1.89 | 1.53E-03 | 663 |
| Cluster-40555.173236 | 2.95 | 3.53 | 13.72 | 13.12 | 2.04 | 3.44 | 12.57 | 16.2 | 2.21 | 4.92E-02 | 2.47 | 1.06E-02 | 485 |
| Cluster-40555.261844 | 0.5 | 0.45 | 2.2 | 1.79 | 1.34 | 0.92 | 3.1 | 2.86 | 2.21 | 1.21E-02 | 1.48 | 3.83E-02 | 2391 |
| Cluster-40555.178412 | 0.16 | 0.86 | 2.09 | 2.27 | 0.49 | 0.65 | 2.51 | 2.52 | 2.21 | 2.00E-03 | 2.23 | 2.97E-04 | 2954 |
| Cluster-40555.173041 | 4.27 | 1.43 | 13.99 | 9.14 | 0.27 | 0.87 | 6.09 | 5.83 | 2.21 | 1.20E-02 | 3.51 | 4.08E-03 | 629 |

| | | | | | | | | | | | | | |
|---|---|---|---|---|---|---|---|---|---|---|---|---|---|
| Cluster-40555.203279 | 1.95 | 2.24 | 8.51 | 9.18 | 2.52 | 3.62 | 11.85 | 10.71 | 2.21 | 1.51E-04 | 1.93 | 1.75E-04 | 1174 |
| Cluster-40555.107046 | 0.14 | 0.52 | 1.26 | 1.61 | 0.39 | 0.3 | 1.92 | 2.96 | 2.21 | 4.39E-02 | 2.90 | 1.05E-03 | 2386 |
| Cluster-40555.140706 | 0.84 | 0.87 | 4.11 | 3.13 | 0.85 | 0.12 | 5.52 | 5.68 | 2.21 | 1.05E-02 | 3.65 | 2.24E-08 | 1487 |
| Cluster-40555.201743 | 4.12 | 0.97 | 11.27 | 9.81 | 0.49 | 0.13 | 13.22 | 8.71 | 2.22 | 1.30E-05 | 5.22 | 1.57E-12 | 1252 |
| Cluster-40555.161574 | 12.35 | 11.22 | 50.41 | 48.94 | 19.87 | 14.86 | 34.65 | 30.02 | 2.22 | 2.46E-12 | 0.96 | 4.76E-02 | 1008 |
| Cluster-40555.185999 | 3.37 | 5.96 | 18.93 | 20.88 | 0.93 | 2.08 | 24.19 | 21.3 | 2.22 | 3.93E-04 | 3.96 | 6.87E-11 | 622 |
| Cluster-40555.214994 | 0 | 0.64 | 1.51 | 1.31 | 1.17 | 0.96 | 2.92 | 2.52 | 2.22 | 1.69E-02 | 1.42 | 2.47E-02 | 3203 |
| Cluster-40555.166887 | 4.88 | 3.35 | 17.31 | 17.21 | 0.82 | 0.95 | 13.5 | 11.68 | 2.22 | 1.42E-04 | 3.88 | 3.83E-08 | 726 |
| Cluster-40555.184401 | 9.95 | 21.54 | 68.81 | 66.51 | 2.66 | 3.88 | 27.74 | 23.46 | 2.22 | 4.00E-14 | 3.02 | 4.93E-13 | 1035 |
| Cluster-40555.191173 | 11.2 | 18.8 | 64.92 | 64.02 | 6.2 | 3.48 | 25.24 | 30.44 | 2.23 | 1.38E-14 | 2.61 | 6.16E-11 | 1112 |
| Cluster-40555.192776 | 1.86 | 2.09 | 7.79 | 9.04 | 1.42 | 2.04 | 5.73 | 6.18 | 2.23 | 1.96E-09 | 1.85 | 3.18E-05 | 2865 |
| Cluster-40555.193728 | 2.7 | 7.21 | 21.55 | 21.26 | 5.87 | 8.12 | 26.81 | 25.08 | 2.23 | 1.45E-05 | 1.94 | 3.88E-05 | 767 |
| Cluster-40555.217373 | 3.61 | 4.34 | 17.92 | 16.16 | 7.8 | 10.33 | 17.84 | 17.24 | 2.23 | 5.16E-13 | 1.01 | 1.13E-02 | 2743 |
| Cluster-40555.179600 | 0.89 | 1.76 | 5.81 | 5.62 | 3.9 | 3 | 11.87 | 12.48 | 2.23 | 3.02E-02 | 1.89 | 2.24E-03 | 857 |
| Cluster-40555.198511 | 6.2 | 6.05 | 29.75 | 22.28 | 4.12 | 3.07 | 10.27 | 9.14 | 2.23 | 3.49E-07 | 1.50 | 4.32E-02 | 865 |
| Cluster-40555.184507 | 0.48 | 0.96 | 3.48 | 2.78 | 6.44 | 7.8 | 15.73 | 16.85 | 2.23 | 1.12E-08 | 1.26 | 1.67E-04 | 7061 |
| Cluster-40555.208125 | 4.65 | 4.27 | 19.13 | 19.01 | 4.13 | 4.65 | 10.75 | 13.32 | 2.24 | 2.12E-09 | 1.53 | 1.47E-03 | 1420 |
| Cluster-40555.249823 | 0.37 | 0 | 0.85 | 0.7 | 0 | 0.06 | 2.56 | 2.49 | 2.24 | 3.89E-02 | 6.34 | 1.81E-20 | 4122 |
| Cluster-40555.182771 | 6 | 4.51 | 22.32 | 22.58 | 3.79 | 2.56 | 11.67 | 8.92 | 2.24 | 4.79E-13 | 1.77 | 4.31E-05 | 2014 |
| Cluster-40555.177528 | 3.82 | 3.48 | 16.39 | 15.02 | 16.98 | 11.82 | 31.15 | 27.76 | 2.24 | 4.41E-12 | 1.11 | 3.12E-03 | 2485 |
| Cluster-40555.182308 | 2.92 | 3.14 | 12.96 | 13.23 | 2.09 | 1.73 | 8.56 | 8.69 | 2.25 | 8.77E-08 | 2.25 | 9.49E-06 | 1513 |
| Cluster-40555.200354 | 11.81 | 2.66 | 33.09 | 27.95 | 2.79 | 2.16 | 25.49 | 22.42 | 2.25 | 1.32E-08 | 3.34 | 1.13E-19 | 1781 |
| Cluster-40555.227322 | 1.06 | 0.85 | 4.43 | 3.83 | 1.77 | 1.97 | 4.72 | 5.28 | 2.25 | 3.50E-05 | 1.48 | 4.40E-03 | 2567 |
| Cluster-40555.205229 | 0.49 | 0.22 | 1.47 | 1.57 | 0.65 | 0.8 | 2.18 | 2.04 | 2.25 | 6.69E-03 | 1.61 | 2.08E-02 | 3113 |
| Cluster-40555.168029 | 1.3 | 1.12 | 5.21 | 5.22 | 1.7 | 1.89 | 3.95 | 4.19 | 2.25 | 3.99E-06 | 1.25 | 4.45E-02 | 2487 |
| Cluster-40555.268464 | 0.53 | 0.39 | 2.06 | 1.81 | 0 | 0.21 | 1.05 | 1.81 | 2.26 | 3.13E-02 | 3.84 | 3.16E-03 | 1942 |
| Cluster-40555.191919 | 0.38 | 0 | 0.67 | 0.88 | 0 | 0 | 0.55 | 1.76 | 2.26 | 2.76E-02 | Inf | 2.07E-02 | 4128 |
| Cluster-40555.194377 | 3.39 | 2.56 | 14.14 | 11.47 | 0.47 | 2.21 | 14.62 | 13.4 | 2.26 | 5.25E-03 | 3.43 | 2.05E-06 | 634 |
| Cluster-40555.190448 | 2.22 | 2.9 | 13.45 | 9.05 | 6.71 | 6.19 | 15.75 | 20.53 | 2.27 | 2.77E-05 | 1.57 | 4.89E-04 | 1440 |
| Cluster-40555.220761 | 0.43 | 0.73 | 2.68 | 2.47 | 0.18 | 0.28 | 1.97 | 1.66 | 2.27 | 7.24E-04 | 3.05 | 5.19E-05 | 2797 |
| Cluster-40555.203814 | 1.55 | 2.21 | 7.51 | 9.06 | 6.19 | 3.75 | 14.1 | 14.88 | 2.27 | 1.75E-05 | 1.62 | 3.33E-04 | 1430 |
| Cluster-40555.214618 | 0.63 | 0.45 | 2.44 | 2.33 | 0.32 | 0 | 3.16 | 2.67 | 2.27 | 3.00E-02 | 4.34 | 1.57E-06 | 1609 |

| | | | | | | | | | | | | | |
|---|---|---|---|---|---|---|---|---|---|---|---|---|---|
| Cluster-40555.233884 | 0.65 | 0.8 | 3.28 | 3.06 | 0.46 | 0.37 | 2.32 | 1.53 | 2.27 | 3.55E-04 | 2.27 | 4.09E-03 | 2494 |
| Cluster-40555.168512 | 0.81 | 0.62 | 3.41 | 2.87 | 2.3 | 1.15 | 4.86 | 3.88 | 2.28 | 6.38E-05 | 1.42 | 7.28E-03 | 3040 |
| Cluster-40555.188073 | 0.58 | 0.68 | 3.04 | 2.63 | 0.33 | 0.25 | 5.98 | 4.31 | 2.28 | 6.07E-03 | 4.28 | 3.18E-12 | 1889 |
| Cluster-40555.157336 | 0 | 1.22 | 3.06 | 2.5 | 0.08 | 0.02 | 3.21 | 2.76 | 2.28 | 3.86E-03 | 6.01 | 1.48E-12 | 2180 |
| Cluster-40555.175142 | 3.52 | 0.89 | 9.21 | 9.79 | 9.44 | 3.97 | 14.27 | 17.92 | 2.28 | 2.48E-06 | 1.36 | 4.90E-02 | 1446 |
| Cluster-40555.209407 | 6.09 | 4.28 | 26.74 | 18.99 | 12.58 | 9.74 | 22.08 | 23.64 | 2.28 | 1.79E-07 | 1.11 | 2.26E-02 | 1113 |
| Cluster-40555.194379 | 5.34 | 7.76 | 27.71 | 30.22 | 6.77 | 9.75 | 35.64 | 29.55 | 2.28 | 5.09E-06 | 2.03 | 2.24E-05 | 644 |
| Cluster-40555.151233 | 2 | 4.26 | 13.32 | 14.72 | 3.66 | 5.98 | 15.67 | 16.1 | 2.29 | 1.09E-09 | 1.77 | 4.54E-06 | 1848 |
| Cluster-40555.213103 | 0.43 | 0 | 0.97 | 0.88 | 0.2 | 0 | 0.82 | 0.92 | 2.29 | 1.63E-02 | 3.26 | 6.23E-04 | 4037 |
| Cluster-40555.240648 | 0.66 | 0.77 | 2.5 | 3.82 | 0.73 | 1.5 | 6.73 | 8.45 | 2.29 | 2.09E-03 | 2.81 | 6.35E-09 | 1850 |
| Cluster-40555.225000 | 2.47 | 4.22 | 15.8 | 14.25 | 9.05 | 9.79 | 16.62 | 18.41 | 2.29 | 1.38E-11 | 0.96 | 2.61E-02 | 2347 |
| Cluster-40555.194608 | 2.98 | 3.65 | 13.6 | 16.04 | 2.69 | 3.44 | 12.03 | 11.78 | 2.30 | 5.27E-07 | 2.02 | 7.56E-05 | 1150 |
| Cluster-40555.187797 | 14.72 | 15.86 | 68.85 | 67.86 | 25.37 | 23.27 | 46.41 | 42.92 | 2.30 | 2.24E-16 | 0.94 | 2.75E-02 | 1177 |
| Cluster-40555.207899 | 3.8 | 5.84 | 21.09 | 22.19 | 15.4 | 16.85 | 32.14 | 40.03 | 2.30 | 2.66E-12 | 1.23 | 9.21E-04 | 1797 |
| Cluster-40555.194407 | 6.19 | 4.62 | 25.59 | 22.52 | 0.2 | 0.17 | 20.06 | 20.19 | 2.30 | 3.05E-10 | 6.78 | 1.18E-34 | 1263 |
| Cluster-40555.181014 | 3.56 | 0 | 7.03 | 8.32 | 0.83 | 2.14 | 7.41 | 6.71 | 2.30 | 8.96E-04 | 2.29 | 1.32E-06 | 1986 |
| Cluster-40555.197445 | 4.41 | 5.11 | 20.71 | 22.02 | 1.54 | 1.41 | 11.07 | 13.37 | 2.30 | 1.49E-10 | 3.13 | 2.36E-11 | 1421 |
| Cluster-40555.172769 | 1.19 | 2.36 | 8.87 | 7.17 | 0.54 | 0.38 | 3.17 | 2.84 | 2.30 | 2.22E-07 | 2.78 | 3.70E-05 | 2178 |
| Cluster-40555.184284 | 4.86 | 5.44 | 25.84 | 20.96 | 6.3 | 5.24 | 13.11 | 12.11 | 2.31 | 1.72E-15 | 1.20 | 3.66E-03 | 2669 |
| Cluster-40555.189455 | 22.19 | 21.09 | 101.41 | 94.67 | 5.26 | 7.4 | 116.89 | 104.91 | 2.32 | 2.78E-19 | 4.19 | 1.65E-37 | 1279 |
| Cluster-40555.180061 | 14.62 | 10.1 | 49.48 | 61.57 | 12.88 | 12.27 | 64.37 | 52.94 | 2.32 | 3.47E-10 | 2.28 | 1.25E-10 | 914 |
| Cluster-40555.185904 | 8.21 | 8.47 | 35.76 | 38.58 | 21.72 | 20.44 | 91.11 | 82.64 | 2.32 | 3.09E-02 | 2.08 | 1.33E-03 | 340 |
| Cluster-40555.190194 | 3.2 | 3.73 | 14.91 | 16.5 | 0.72 | 1.3 | 3.06 | 4.1 | 2.32 | 2.39E-08 | 1.89 | 3.18E-02 | 1319 |
| Cluster-40555.206657 | 0.29 | 1.81 | 4.05 | 5.82 | 1.07 | 0.52 | 4.87 | 3.77 | 2.33 | 2.62E-02 | 2.48 | 1.97E-02 | 906 |
| Cluster-40555.198579 | 2.52 | 3.19 | 12.26 | 13.89 | 1.68 | 1.36 | 7.31 | 8.3 | 2.33 | 4.81E-08 | 2.44 | 5.97E-06 | 1459 |
| Cluster-40555.146851 | 0.84 | 0.17 | 2.36 | 2.12 | 0 | 0 | 3.09 | 2.58 | 2.33 | 3.98E-02 | Inf | 3.79E-10 | 1519 |
| Cluster-40555.169012 | 1.31 | 0.73 | 4.42 | 4.77 | 0.37 | 0.1 | 3.93 | 3.42 | 2.33 | 8.97E-05 | 4.11 | 5.76E-09 | 1922 |
| Cluster-40555.161868 | 9.28 | 13.09 | 51.63 | 51.42 | 4.86 | 4.3 | 29.55 | 20.78 | 2.34 | 3.94E-08 | 2.52 | 1.49E-05 | 577 |
| Cluster-40555.199759 | 1.42 | 0 | 3.53 | 2.75 | 0.41 | 1.57 | 3.56 | 3.24 | 2.34 | 8.72E-04 | 1.82 | 2.66E-03 | 2303 |
| Cluster-40555.187265 | 3.32 | 4.18 | 17.51 | 17.16 | 4.18 | 5.05 | 26.39 | 25.38 | 2.34 | 8.95E-08 | 2.55 | 1.52E-10 | 1133 |
| Cluster-40555.160292 | 2.68 | 1.47 | 9.78 | 9.35 | 0.86 | 0.98 | 7.58 | 6.03 | 2.35 | 6.62E-04 | 2.99 | 1.70E-04 | 887 |
| Cluster-40555.195001 | 2.03 | 2.44 | 10.67 | 10.12 | 1.31 | 2.06 | 8.49 | 7.94 | 2.35 | 3.17E-06 | 2.33 | 1.97E-05 | 1329 |

| | | | | | | | | | | | | | |
|---|---|---|---|---|---|---|---|---|---|---|---|---|---|
| Cluster-40555.169421 | 2.72 | 4.85 | 17.95 | 17.52 | 1.96 | 4.49 | 19.93 | 23.58 | 2.36 | 2.37E-12 | 2.81 | 8.21E-14 | 2055 |
| Cluster-40555.191873 | 13.52 | 15.35 | 65.58 | 69.08 | 14.07 | 14.43 | 94.7 | 105.24 | 2.36 | 6.51E-19 | 2.88 | 1.91E-20 | 1481 |
| Cluster-40555.200552 | 10.73 | 9.77 | 48.85 | 46.46 | 39.77 | 42.38 | 72.95 | 68.91 | 2.36 | 3.58E-16 | 0.85 | 3.58E-02 | 1400 |
| Cluster-40555.206718 | 3.77 | 5.77 | 24.41 | 20.39 | 8.53 | 7.57 | 31.89 | 25.53 | 2.36 | 1.95E-09 | 1.90 | 8.24E-07 | 1185 |
| Cluster-40555.188801 | 5.96 | 5.38 | 28.98 | 24.12 | 4.33 | 3.04 | 9.25 | 9.29 | 2.36 | 3.97E-18 | 1.41 | 5.58E-04 | 3130 |
| Cluster-40555.139603 | 0 | 0.64 | 1.78 | 1.38 | 0.06 | 0 | 0.79 | 0.94 | 2.37 | 1.59E-03 | 4.95 | 1.82E-06 | 4057 |
| Cluster-40555.200581 | 2.29 | 2.23 | 9.75 | 11.35 | 2.55 | 3.36 | 9.98 | 7.48 | 2.37 | 1.02E-05 | 1.61 | 7.56E-03 | 1153 |
| Cluster-40555.209116 | 2.7 | 4.27 | 18.18 | 15.01 | 5.07 | 3.31 | 10.08 | 8.91 | 2.37 | 3.55E-09 | 1.25 | 2.77E-02 | 1445 |
| Cluster-40555.189038 | 14.49 | 20.92 | 87.31 | 80.3 | 5.28 | 4.06 | 41.28 | 47.65 | 2.37 | 2.16E-19 | 3.32 | 1.95E-21 | 1375 |
| Cluster-40555.160644 | 0.5 | 0.2 | 1.67 | 1.6 | 0.64 | 0.39 | 2.99 | 1.94 | 2.38 | 2.94E-02 | 2.34 | 2.70E-03 | 2061 |
| Cluster-40555.185643 | 5.38 | 10.18 | 38.53 | 35.66 | 3.2 | 2.43 | 16.03 | 15.58 | 2.38 | 2.50E-13 | 2.56 | 1.05E-08 | 1240 |
| Cluster-40555.232977 | 1.97 | 1.88 | 9.39 | 8.69 | 2.82 | 1.85 | 5.34 | 6.34 | 2.38 | 3.61E-06 | 1.40 | 4.62E-02 | 1440 |
| Cluster-40555.148780 | 0.32 | 0.55 | 1.97 | 2.19 | 0.86 | 0.61 | 4.52 | 4.22 | 2.38 | 1.03E-02 | 2.64 | 5.11E-06 | 2075 |
| Cluster-40555.210546 | 6.22 | 13.08 | 48.93 | 43.87 | 21.27 | 18.28 | 32.55 | 39.85 | 2.38 | 5.28E-14 | 0.95 | 4.01E-02 | 1131 |
| Cluster-40555.189329 | 289.76 | 422.68 | 1884.82 | 1530.93 | 16.85 | 3.53 | 158.12 | 292.34 | 2.39 | 9.35E-21 | 4.57 | 7.19E-04 | 669 |
| Cluster-40555.136574 | 0.71 | 1.24 | 5.31 | 4.13 | 0 | 0 | 7.05 | 6.59 | 2.39 | 2.95E-04 | Inf | 2.17E-25 | 1724 |
| Cluster-40555.191033 | 1.66 | 1.12 | 7.18 | 6.07 | 0 | 0.29 | 2.9 | 3.89 | 2.39 | 1.02E-09 | 4.57 | 7.83E-12 | 3296 |
| Cluster-40555.208892 | 2.12 | 0.79 | 8.19 | 5.57 | 1.88 | 1.58 | 6.71 | 4.7 | 2.40 | 1.43E-03 | 1.80 | 2.67E-02 | 1046 |
| Cluster-40555.185110 | 22.73 | 32.46 | 122.03 | 143.11 | 78.89 | 72.21 | 159.21 | 160.69 | 2.40 | 2.26E-15 | 1.15 | 7.14E-04 | 1243 |
| Cluster-40555.190196 | 0.69 | 1.31 | 5.33 | 4.25 | 0.49 | 0.35 | 4.82 | 4.28 | 2.40 | 1.69E-03 | 3.49 | 2.12E-06 | 1383 |
| Cluster-40555.186677 | 3.29 | 0.8 | 9.11 | 9.99 | 2.04 | 0.82 | 7.17 | 9.42 | 2.40 | 4.34E-06 | 2.62 | 4.63E-06 | 1272 |
| Cluster-40555.187858 | 141.93 | 106.82 | 524.48 | 656.72 | 266.31 | 247.94 | 887.4 | 634.88 | 2.40 | 1.77E-10 | 1.63 | 2.78E-05 | 877 |
| Cluster-40555.186941 | 116.46 | 118.91 | 560.18 | 573.16 | 432.7 | 416.26 | 786.96 | 788.35 | 2.41 | 5.37E-27 | 0.96 | 5.68E-03 | 1307 |
| Cluster-40555.169432 | 5.53 | 10.75 | 36.8 | 42.54 | 9.79 | 16.55 | 53.49 | 42.58 | 2.41 | 1.20E-13 | 1.92 | 1.07E-08 | 1332 |
| Cluster-40555.162559 | 1.66 | 1.71 | 7.97 | 8.45 | 2.16 | 2.94 | 13.12 | 11.97 | 2.41 | 5.35E-04 | 2.35 | 1.37E-05 | 981 |
| Cluster-40555.170890 | 1.35 | 2.38 | 9.17 | 9.2 | 1.25 | 2.11 | 6.23 | 4.32 | 2.41 | 3.08E-05 | 1.71 | 2.56E-02 | 1180 |
| Cluster-40555.221171 | 1.89 | 1.66 | 8.39 | 8.81 | 2.15 | 0.99 | 5.82 | 6.25 | 2.41 | 7.86E-07 | 2.03 | 5.07E-04 | 1607 |
| Cluster-40555.183176 | 1.12 | 0.64 | 4.77 | 3.93 | 1.55 | 1.71 | 9.11 | 6.3 | 2.42 | 3.23E-02 | 2.31 | 1.19E-03 | 932 |
| Cluster-40555.178753 | 3.63 | 4.31 | 18.34 | 20.1 | 2.24 | 1.64 | 6.86 | 11 | 2.42 | 1.05E-07 | 2.26 | 1.57E-02 | 970 |
| Cluster-40555.189827 | 38.79 | 27.53 | 164.88 | 156.15 | 85.34 | 84.73 | 240.73 | 195.26 | 2.42 | 1.40E-26 | 1.42 | 3.42E-06 | 2796 |
| Cluster-40555.195075 | 5.47 | 7.09 | 31.26 | 30.19 | 27.23 | 18.82 | 43.75 | 43.96 | 2.42 | 5.19E-08 | 1.00 | 4.74E-02 | 740 |
| Cluster-40555.209363 | 7.04 | 6.75 | 34.9 | 32.11 | 4.65 | 3.9 | 33.85 | 32.4 | 2.42 | 6.04E-07 | 3.02 | 1.54E-09 | 621 |

| | | | | | | | | | | | | | |
|---|---|---|---|---|---|---|---|---|---|---|---|---|---|
| Cluster-40555.156064 | 1.12 | 1.37 | 5.62 | 6.53 | 2.55 | 3.18 | 10.46 | 9.77 | 2.43 | 2.77E-12 | 1.88 | 4.68E-08 | 4766 |
| Cluster-40555.198509 | 8.91 | 6.93 | 38.14 | 38.89 | 4.48 | 4.12 | 11.94 | 12.67 | 2.43 | 3.55E-13 | 1.59 | 3.80E-03 | 1071 |
| Cluster-40555.246212 | 0.94 | 2.24 | 9.37 | 6.54 | 0.55 | 0.39 | 3.65 | 4.28 | 2.43 | 5.36E-05 | 3.21 | 7.81E-05 | 1318 |
| Cluster-40555.188081 | 301.41 | 330.22 | 1528.54 | 1566.54 | 911.65 | 838.95 | 1724.8 | 1705 | 2.43 | 1.81E-28 | 1.04 | 1.89E-03 | 1221 |
| Cluster-40555.192571 | 2.41 | 5.5 | 21.92 | 17.48 | 6.16 | 7.28 | 22.85 | 20.65 | 2.43 | 1.48E-08 | 1.75 | 3.42E-05 | 1122 |
| Cluster-40555.110400 | 0.52 | 1.04 | 3.81 | 3.96 | 0.71 | 1.01 | 5.38 | 3.5 | 2.43 | 1.20E-02 | 2.41 | 2.72E-03 | 1184 |
| Cluster-40555.178108 | 1.42 | 2.05 | 9.4 | 7.84 | 2.77 | 3.06 | 5.51 | 6.09 | 2.44 | 8.84E-13 | 1.06 | 2.47E-02 | 3892 |
| Cluster-40555.203221 | 18.86 | 22.94 | 106.92 | 101.29 | 0.64 | 2.17 | 52.76 | 55.85 | 2.45 | 3.50E-13 | 5.31 | 1.57E-24 | 564 |
| Cluster-40555.193055 | 32.09 | 51.5 | 203.74 | 214.62 | 10.65 | 13.98 | 216.84 | 177.66 | 2.45 | 1.92E-16 | 4.05 | 2.64E-28 | 494 |
| Cluster-40555.191068 | 21.34 | 24.81 | 107.79 | 122.53 | 34 | 28.97 | 54.03 | 68.99 | 2.46 | 1.49E-23 | 1.04 | 1.28E-02 | 1468 |
| Cluster-40555.187546 | 26.18 | 41.02 | 190.67 | 148.18 | 13.3 | 10.01 | 73.82 | 95.08 | 2.46 | 1.64E-14 | 2.93 | 1.14E-09 | 2112 |
| Cluster-40555.202265 | 0.79 | 3.39 | 11.54 | 9.98 | 0.85 | 0.52 | 3.23 | 3.83 | 2.46 | 1.57E-07 | 2.44 | 1.57E-03 | 1545 |
| Cluster-40555.141153 | 0.56 | 0.69 | 2.8 | 3.4 | 0.07 | 0.36 | 1.45 | 1.49 | 2.46 | 1.05E-03 | 2.77 | 1.24E-02 | 1914 |
| Cluster-40555.191510 | 3.2 | 2.57 | 15.79 | 13.14 | 8.02 | 6.51 | 15.61 | 15.87 | 2.47 | 3.26E-11 | 1.19 | 6.00E-03 | 1898 |
| Cluster-40555.202608 | 5.36 | 2.7 | 21.09 | 18.81 | 0.44 | 0.45 | 5.9 | 5.68 | 2.47 | 8.54E-08 | 3.81 | 1.14E-05 | 930 |
| Cluster-40555.172395 | 1.77 | 0.76 | 7.31 | 5.37 | 1.71 | 1.1 | 4.37 | 5.45 | 2.47 | 5.12E-04 | 1.87 | 2.11E-02 | 1189 |
| Cluster-40555.229946 | 0.38 | 0.63 | 2.81 | 2.33 | 1.72 | 2.47 | 18.13 | 14.48 | 2.47 | 1.84E-02 | 3.01 | 2.48E-13 | 1533 |
| Cluster-40555.198790 | 4.42 | 5.74 | 25.91 | 25.69 | 8.57 | 9.73 | 21.21 | 27.37 | 2.48 | 8.63E-15 | 1.48 | 7.87E-04 | 1725 |
| Cluster-40555.196339 | 0.37 | 0.58 | 2.68 | 2.12 | 1.99 | 1.14 | 3.7 | 4.47 | 2.48 | 7.68E-05 | 1.47 | 4.21E-03 | 3410 |
| Cluster-40555.135315 | 0.75 | 0.35 | 2.97 | 2.53 | 0 | 0.29 | 4.97 | 5.34 | 2.48 | 5.64E-04 | 5.19 | 2.16E-18 | 2319 |
| Cluster-40555.219444 | 1.95 | 2.02 | 9.52 | 10.71 | 1.81 | 3.27 | 8 | 10.59 | 2.48 | 2.83E-07 | 1.94 | 5.91E-04 | 1435 |
| Cluster-40555.201169 | 3.39 | 2.77 | 16.54 | 14.71 | 5.51 | 3.92 | 12.78 | 10.09 | 2.49 | 1.41E-12 | 1.35 | 2.18E-03 | 2039 |
| Cluster-40555.180564 | 7.9 | 20.8 | 69.56 | 79.3 | 14.29 | 19.94 | 68.4 | 74.29 | 2.49 | 1.40E-12 | 2.12 | 5.64E-09 | 727 |
| Cluster-40555.189072 | 0.79 | 1.6 | 5.8 | 6.58 | 0.56 | 0.56 | 3.5 | 2.32 | 2.49 | 2.37E-04 | 2.47 | 1.36E-02 | 1271 |
| Cluster-40555.170546 | 14.9 | 12 | 67.44 | 68.47 | 31.62 | 25.46 | 64.93 | 75.66 | 2.49 | 1.31E-10 | 1.37 | 1.89E-03 | 560 |
| Cluster-40555.88659 | 0.77 | 1.77 | 6.64 | 6.64 | 3.32 | 3.07 | 9.18 | 9.15 | 2.49 | 7.00E-05 | 1.58 | 3.52E-03 | 1369 |
| Cluster-40555.184803 | 16.46 | 33.87 | 140.98 | 120.16 | 19.08 | 20.35 | 68.25 | 89.23 | 2.49 | 5.91E-21 | 2.06 | 7.61E-06 | 997 |
| Cluster-40555.207438 | 1.73 | 1.62 | 8.72 | 8.32 | 2.14 | 1.16 | 4.06 | 5.15 | 2.50 | 9.16E-07 | 1.56 | 3.50E-02 | 1540 |
| Cluster-40555.175045 | 7.81 | 4.92 | 35.18 | 29.54 | 1.2 | 1.64 | 13.11 | 13.43 | 2.50 | 5.17E-10 | 3.29 | 3.23E-08 | 848 |
| Cluster-40555.215324 | 0.34 | 0.51 | 2.41 | 2.01 | 0.15 | 0.31 | 1.49 | 1.84 | 2.50 | 1.33E-02 | 2.89 | 6.98E-03 | 1816 |
| Cluster-40555.231932 | 2.47 | 3.42 | 14.08 | 16.26 | 6.91 | 7.79 | 20.79 | 19.68 | 2.50 | 3.52E-07 | 1.52 | 9.88E-04 | 1023 |
| Cluster-40555.179056 | 0.69 | 3.55 | 13.39 | 9.01 | 0.95 | 1.72 | 6.08 | 7.85 | 2.50 | 8.71E-04 | 2.45 | 5.05E-03 | 778 |

| | | | | | | | | | | | | | |
|---|---|---|---|---|---|---|---|---|---|---|---|---|---|
| Cluster-40555.170654 | 4.37 | 3.67 | 20.33 | 20.95 | 5.13 | 4.68 | 24.47 | 27.38 | 2.50 | 3.74E-09 | 2.47 | 1.96E-09 | 1045 |
| Cluster-40555.142194 | 0.31 | 0.33 | 1.42 | 1.88 | 0.31 | 0.23 | 2.57 | 2.02 | 2.51 | 3.14E-03 | 3.14 | 3.62E-06 | 2779 |
| Cluster-40555.234717 | 0.56 | 0.51 | 3.11 | 2.32 | 0.21 | 0 | 1.22 | 1.39 | 2.51 | 2.27E-02 | 3.81 | 2.21E-02 | 1400 |
| Cluster-40555.190546 | 30.36 | 29.71 | 157.13 | 153.11 | 111 | 108.8 | 206.92 | 203.04 | 2.51 | 1.75E-26 | 0.97 | 5.96E-03 | 1794 |
| Cluster-40555.210611 | 3.22 | 3.21 | 15.47 | 17.7 | 6.08 | 7.37 | 14.61 | 15.86 | 2.51 | 1.26E-10 | 1.24 | 7.17E-03 | 1453 |
| Cluster-40555.179928 | 1.73 | 1.68 | 8.63 | 9.04 | 4 | 3.35 | 16.87 | 14.81 | 2.51 | 2.06E-13 | 2.17 | 1.14E-10 | 3583 |
| Cluster-40555.189707 | 10.55 | 13.24 | 62.53 | 61.17 | 36.62 | 40.92 | 117.15 | 101.94 | 2.51 | 3.59E-17 | 1.56 | 1.74E-06 | 1058 |
| Cluster-40555.235552 | 0.37 | 0.3 | 1.65 | 1.76 | 1.53 | 1.02 | 5.34 | 5.45 | 2.51 | 1.20E-02 | 2.16 | 2.93E-05 | 2199 |
| Cluster-40555.167198 | 2.02 | 1.01 | 7.7 | 7.91 | 1.27 | 1.43 | 6.2 | 5.9 | 2.52 | 1.78E-09 | 2.22 | 2.71E-06 | 2370 |
| Cluster-40555.196659 | 4.18 | 2.29 | 17.07 | 16.39 | 5.29 | 4.66 | 12.84 | 12.27 | 2.52 | 3.47E-09 | 1.40 | 7.42E-03 | 1216 |
| Cluster-40555.203224 | 2.73 | 2.02 | 12.71 | 11.86 | 5.59 | 4.91 | 23.74 | 22.65 | 2.52 | 1.87E-10 | 2.21 | 5.93E-10 | 1853 |
| Cluster-40555.181908 | 7.6 | 26.06 | 83.93 | 95.04 | 14.57 | 25.71 | 111.71 | 89.22 | 2.52 | 3.02E-11 | 2.37 | 3.89E-13 | 873 |
| Cluster-40555.206817 | 0 | 0.45 | 1.16 | 1.27 | 0 | 0.2 | 0.93 | 1.64 | 2.53 | 3.49E-02 | 3.73 | 5.81E-03 | 2504 |
| Cluster-40555.208689 | 1.9 | 1.51 | 8.9 | 8.99 | 2.04 | 1.82 | 6.91 | 7.48 | 2.53 | 2.48E-09 | 1.98 | 3.17E-05 | 2074 |
| Cluster-40555.234623 | 0.73 | 1.79 | 8.29 | 5.3 | 0.27 | 0.19 | 2.68 | 2.62 | 2.53 | 3.98E-04 | 3.55 | 2.12E-04 | 1462 |
| Cluster-40555.210779 | 2.02 | 1.34 | 7.79 | 9.48 | 0.2 | 0 | 3.01 | 4.41 | 2.53 | 1.35E-03 | 5.52 | 2.39E-04 | 798 |
| Cluster-40555.202869 | 1.12 | 0.97 | 4.78 | 6.12 | 2.3 | 1.23 | 9.32 | 6.92 | 2.54 | 1.68E-06 | 2.28 | 7.29E-07 | 2022 |
| Cluster-40555.184891 | 3.97 | 4.94 | 24.52 | 22.64 | 1.1 | 1.1 | 13.02 | 13.88 | 2.54 | 1.08E-10 | 3.70 | 9.22E-13 | 1127 |
| Cluster-40555.190017 | 95.91 | 112.64 | 683.67 | 423.79 | 395.17 | 494.81 | 873.26 | 808.97 | 2.54 | 2.86E-04 | 0.98 | 3.70E-03 | 1340 |
| Cluster-40555.180013 | 24.36 | 21.21 | 111.9 | 123.34 | 54.08 | 74.4 | 230.51 | 167.38 | 2.54 | 9.88E-08 | 1.66 | 9.78E-04 | 364 |
| Cluster-40555.185800 | 3.2 | 5.61 | 23.13 | 23.76 | 6.95 | 5.73 | 13.74 | 13.14 | 2.54 | 6.95E-17 | 1.15 | 7.71E-03 | 2222 |
| Cluster-40555.182990 | 84.29 | 123.14 | 546.23 | 553.9 | 128.91 | 95.3 | 206.57 | 216.3 | 2.54 | 1.46E-24 | 0.98 | 1.61E-02 | 520 |
| Cluster-40555.157875 | 0.87 | 1.21 | 6 | 5.36 | 0.58 | 1.26 | 4.4 | 4.42 | 2.54 | 1.32E-02 | 2.33 | 3.35E-02 | 850 |
| Cluster-40555.205132 | 3.9 | 6.09 | 26.35 | 26.68 | 9.05 | 6.13 | 24.25 | 20.82 | 2.54 | 2.08E-05 | 1.62 | 1.59E-02 | 562 |
| Cluster-40555.205139 | 0.17 | 0.4 | 1.21 | 1.91 | 0.03 | 0 | 1.07 | 0.95 | 2.54 | 1.51E-02 | 6.53 | 6.70E-05 | 2215 |
| Cluster-40555.187720 | 1.48 | 1.53 | 7.87 | 8.08 | 19.67 | 16.18 | 32.25 | 28.2 | 2.54 | 9.27E-12 | 0.82 | 4.87E-02 | 3133 |
| Cluster-40555.194245 | 2.48 | 4.16 | 20.14 | 15.66 | 10.39 | 10.73 | 29.25 | 27.73 | 2.55 | 7.24E-05 | 1.49 | 4.25E-03 | 666 |
| Cluster-40555.161856 | 0.73 | 0.61 | 3.47 | 3.61 | 1.58 | 2.03 | 6.46 | 4.71 | 2.55 | 1.02E-03 | 1.68 | 4.75E-03 | 1646 |
| Cluster-40555.171184 | 7.65 | 13.09 | 50.7 | 60.47 | 40.28 | 30.31 | 86.37 | 101.76 | 2.55 | 1.93E-13 | 1.49 | 1.82E-05 | 931 |
| Cluster-40555.189536 | 39.18 | 21.12 | 168.04 | 149.57 | 101.36 | 76.29 | 187.41 | 188.5 | 2.55 | 4.34E-26 | 1.15 | 6.38E-04 | 1358 |
| Cluster-40555.108604 | 0.83 | 0 | 2 | 2.26 | 0 | 0.41 | 1.63 | 1.73 | 2.55 | 1.44E-02 | 3.00 | 7.78E-03 | 1650 |
| Cluster-40555.187061 | 18.48 | 17.28 | 107.28 | 83.16 | 39.48 | 41.72 | 172.03 | 128.56 | 2.55 | 5.49E-16 | 1.94 | 1.10E-08 | 700 |

| | | | | | | | | | | | | | |
|---|---|---|---|---|---|---|---|---|---|---|---|---|---|
| Cluster-40555.214724 | 6.41 | 5.01 | 28.56 | 32.12 | 12.43 | 8.8 | 33.01 | 36.64 | 2.56 | 6.36E-10 | 1.79 | 2.69E-05 | 820 |
| Cluster-40555.185198 | 13.82 | 17.79 | 88.66 | 81.69 | 17.82 | 16.6 | 109.25 | 108.33 | 2.56 | 1.67E-24 | 2.73 | 2.17E-19 | 1855 |
| Cluster-40555.183213 | 4.18 | 4.39 | 23.03 | 22.71 | 2.02 | 2.14 | 13.45 | 11.27 | 2.56 | 8.49E-07 | 2.63 | 1.17E-04 | 717 |
| Cluster-40555.199508 | 1.96 | 2.3 | 12.38 | 10.59 | 7.04 | 6.67 | 14.1 | 13.05 | 2.56 | 7.15E-18 | 1.05 | 5.46E-03 | 4780 |
| Cluster-40555.199142 | 0.67 | 0.37 | 2.59 | 2.96 | 0.81 | 0.53 | 2.77 | 4.16 | 2.56 | 1.72E-03 | 2.45 | 2.80E-03 | 1849 |
| Cluster-40555.180899 | 2.05 | 1.51 | 10.26 | 8.92 | 1.81 | 0.95 | 5.43 | 4.51 | 2.57 | 3.02E-06 | 1.92 | 1.29E-02 | 1241 |
| Cluster-40555.181383 | 1.83 | 3.87 | 12.61 | 18.36 | 4.48 | 9.15 | 21.49 | 21.03 | 2.57 | 2.01E-04 | 1.69 | 1.67E-04 | 975 |
| Cluster-40555.188694 | 251.56 | 571.11 | 2279.7 | 2215.46 | 75.46 | 56.78 | 346.96 | 425.24 | 2.57 | 4.66E-31 | 2.62 | 4.63E-12 | 1425 |
| Cluster-40555.187652 | 23.95 | 58.87 | 238.67 | 215.17 | 105.92 | 124.99 | 218.07 | 190.03 | 2.57 | 1.73E-26 | 0.88 | 1.64E-02 | 1232 |
| Cluster-40555.212439 | 2.04 | 2.5 | 12.01 | 12.44 | 1.28 | 0.29 | 11.4 | 10.6 | 2.57 | 3.83E-05 | 3.95 | 6.45E-09 | 854 |
| Cluster-40555.189382 | 1.97 | 2.86 | 12.36 | 13.93 | 0.48 | 0.39 | 4.04 | 3.83 | 2.57 | 9.34E-10 | 3.26 | 9.35E-06 | 1533 |
| Cluster-40555.215293 | 0.35 | 0.51 | 2.56 | 2.26 | 0.99 | 0.59 | 4.77 | 3.25 | 2.58 | 8.84E-03 | 2.40 | 3.29E-04 | 1724 |
| Cluster-40555.193657 | 1.25 | 1.1 | 6.5 | 6.16 | 2.13 | 2.1 | 7.09 | 5.71 | 2.58 | 3.00E-07 | 1.66 | 1.11E-03 | 2031 |
| Cluster-40555.184510 | 0.29 | 0.37 | 1.68 | 1.93 | 0.49 | 0.32 | 1.54 | 1.34 | 2.58 | 6.89E-04 | 1.90 | 2.72E-02 | 3072 |
| Cluster-40555.169952 | 1.48 | 1.68 | 8.37 | 8.81 | 4.34 | 4.37 | 12.49 | 12 | 2.58 | 1.73E-03 | 1.55 | 2.45E-02 | 784 |
| Cluster-40555.163622 | 1.68 | 5.46 | 21.21 | 18.45 | 0 | 0.85 | 3.69 | 7.77 | 2.58 | 4.94E-12 | 3.82 | 3.22E-02 | 1502 |
| Cluster-40555.192148 | 12.23 | 10.16 | 54.6 | 66.38 | 3.24 | 3.58 | 34.01 | 38.48 | 2.58 | 2.26E-14 | 3.49 | 1.46E-19 | 1094 |
| Cluster-40555.200574 | 10.28 | 14.56 | 70.38 | 65.77 | 16.98 | 14.68 | 47.44 | 47.14 | 2.58 | 5.03E-22 | 1.65 | 1.27E-06 | 1530 |
| Cluster-40555.225032 | 1.06 | 0.61 | 4.04 | 4.83 | 2.99 | 4.03 | 7.13 | 8.43 | 2.58 | 6.59E-05 | 1.21 | 2.97E-02 | 1729 |
| Cluster-40555.214698 | 0.62 | 0.27 | 2.27 | 2.53 | 1.32 | 1.05 | 3.89 | 3.06 | 2.58 | 2.86E-03 | 1.62 | 2.36E-02 | 1933 |
| Cluster-40555.180184 | 5.55 | 6.68 | 33.5 | 33.34 | 15.56 | 14.78 | 29.69 | 26.31 | 2.58 | 5.38E-16 | 0.95 | 3.43E-02 | 1405 |
| Cluster-40555.171562 | 2.61 | 4.36 | 19.71 | 18.7 | 4.48 | 5.59 | 18.33 | 22.75 | 2.59 | 1.26E-17 | 2.09 | 1.09E-07 | 2765 |
| Cluster-40555.239714 | 0.89 | 0.69 | 4.29 | 4.37 | 0.62 | 0.77 | 4.96 | 3.74 | 2.59 | 3.48E-07 | 2.72 | 6.29E-08 | 2764 |
| Cluster-40555.155476 | 3.04 | 5.36 | 23.36 | 23.05 | 0.88 | 0.37 | 10.21 | 8.52 | 2.59 | 1.34E-04 | 3.98 | 5.57E-04 | 535 |
| Cluster-40555.194392 | 7.33 | 10.61 | 54.62 | 44.53 | 9.33 | 11.93 | 21.13 | 24.36 | 2.59 | 3.68E-13 | 1.16 | 3.42E-02 | 823 |
| Cluster-40555.166961 | 1.97 | 1.29 | 8.51 | 9.07 | 3.56 | 1.61 | 9.26 | 11.34 | 2.60 | 1.76E-03 | 2.09 | 5.06E-03 | 758 |
| Cluster-40555.242134 | 1.49 | 4.37 | 15.06 | 17.2 | 1.1 | 1.1 | 5.73 | 6.95 | 2.60 | 3.96E-04 | 2.62 | 4.11E-02 | 602 |
| Cluster-40555.179449 | 1 | 0.79 | 5.5 | 4.4 | 1.28 | 0 | 7.61 | 4.93 | 2.60 | 3.26E-09 | 3.41 | 6.04E-06 | 3412 |
| Cluster-40555.243044 | 3.09 | 0.91 | 10.97 | 10.76 | 1.75 | 2.55 | 12.09 | 9.41 | 2.60 | 1.19E-04 | 2.36 | 3.11E-04 | 822 |
| Cluster-40555.91878 | 0.54 | 0.52 | 2.99 | 2.88 | 0.61 | 1.19 | 3.77 | 3.77 | 2.61 | 1.28E-02 | 2.11 | 9.88E-03 | 1335 |
| Cluster-40555.182385 | 0.19 | 0.71 | 2.01 | 3.24 | 0 | 0 | 2.6 | 1.82 | 2.61 | 4.04E-02 | Inf | 1.54E-05 | 1161 |
| Cluster-40555.190152 | 10.11 | 24.64 | 105.43 | 90.52 | 2.19 | 2.02 | 43.86 | 59.57 | 2.61 | 1.37E-21 | 4.69 | 3.92E-13 | 1105 |

| | | | | | | | | | | | | | |
|---|---|---|---|---|---|---|---|---|---|---|---|---|---|
| Cluster-40555.204221 | 2.09 | 0.95 | 9.37 | 7.2 | 1.14 | 1.43 | 28.33 | 25.29 | 2.61 | 8.02E-03 | 4.44 | 1.35E-15 | 679 |
| Cluster-40555.201251 | 2.3 | 2.53 | 13.61 | 13.18 | 0.41 | 0.87 | 9.97 | 15.16 | 2.61 | 1.28E-02 | 4.34 | 5.35E-05 | 496 |
| Cluster-40555.167368 | 1.5 | 1.35 | 8.19 | 7.62 | 3.44 | 3.05 | 6.81 | 6.59 | 2.61 | 2.39E-11 | 1.12 | 2.45E-02 | 2859 |
| Cluster-40555.159469 | 5.37 | 7.17 | 34.3 | 35.82 | 8.54 | 9.55 | 17.8 | 25.12 | 2.62 | 2.39E-16 | 1.32 | 3.87E-02 | 1350 |
| Cluster-40555.249277 | 2.88 | 1.56 | 13.62 | 11.03 | 1.04 | 0.96 | 8.8 | 7.34 | 2.63 | 1.18E-06 | 3.08 | 1.97E-06 | 1054 |
| Cluster-40555.208256 | 0.61 | 0.2 | 1.95 | 2.55 | 0.48 | 0.42 | 2.8 | 2.64 | 2.63 | 6.78E-03 | 2.65 | 1.22E-03 | 1735 |
| Cluster-40555.169657 | 1.29 | 1.32 | 6.88 | 7.8 | 0.23 | 0.42 | 4.43 | 3.6 | 2.63 | 2.21E-05 | 3.70 | 1.33E-05 | 1247 |
| Cluster-40555.186567 | 3.11 | 1.98 | 13.52 | 14.86 | 1.41 | 1.87 | 10.12 | 13.67 | 2.63 | 6.92E-11 | 2.92 | 4.66E-07 | 1544 |
| Cluster-40555.196138 | 0.2 | 0.3 | 1.37 | 1.6 | 0.09 | 0 | 0.84 | 0.83 | 2.63 | 2.37E-02 | 4.13 | 1.37E-02 | 2079 |
| Cluster-40555.166606 | 0.68 | 1.02 | 4.45 | 5.31 | 0.6 | 0.38 | 2.24 | 2.48 | 2.64 | 1.91E-04 | 2.35 | 2.80E-02 | 1435 |
| Cluster-40555.193345 | 8.1 | 20.01 | 88.61 | 73.09 | 13.92 | 16.62 | 39.97 | 43.27 | 2.64 | 1.50E-17 | 1.51 | 2.04E-04 | 841 |
| Cluster-40555.204167 | 1.96 | 2.05 | 10.64 | 12.09 | 5.28 | 5.23 | 11.21 | 10.48 | 2.64 | 4.26E-10 | 1.11 | 3.09E-02 | 1706 |
| Cluster-40555.189158 | 43.9 | 32.22 | 199.55 | 228 | 77.01 | 126.93 | 475.72 | 514.08 | 2.64 | 1.21E-25 | 2.33 | 1.56E-15 | 922 |
| Cluster-40555.232291 | 2.01 | 0.62 | 6.23 | 8.4 | 1.42 | 1.23 | 5.89 | 4.95 | 2.64 | 1.10E-03 | 2.08 | 3.41E-02 | 854 |
| Cluster-40555.214111 | 0.54 | 1.98 | 7.85 | 6.85 | 1.97 | 0.37 | 7.26 | 6.43 | 2.64 | 1.55E-05 | 2.65 | 1.29E-05 | 1347 |
| Cluster-40555.204791 | 4.82 | 2.85 | 24.3 | 19.07 | 12.66 | 9.27 | 19.32 | 22.11 | 2.65 | 7.49E-18 | 0.99 | 1.82E-02 | 2346 |
| Cluster-40555.157016 | 0.71 | 0.43 | 3.22 | 3.25 | 0.71 | 0.13 | 7.99 | 9.24 | 2.65 | 2.18E-05 | 4.46 | 8.53E-22 | 2486 |
| Cluster-40555.183814 | 0 | 3.85 | 12.83 | 10.06 | 0 | 0.35 | 15.19 | 15.04 | 2.65 | 4.00E-05 | 6.34 | 1.80E-20 | 917 |
| Cluster-40555.186186 | 38.06 | 40.92 | 221.51 | 227.18 | 47.92 | 43.31 | 114.2 | 123.24 | 2.65 | 6.10E-20 | 1.44 | 2.75E-04 | 510 |
| Cluster-40555.213660 | 1.93 | 1.81 | 10.38 | 11 | 0 | 0.23 | 6.4 | 7.75 | 2.65 | 4.47E-11 | 6.00 | 2.22E-23 | 2016 |
| Cluster-40555.90291 | 0.49 | 0.2 | 2.24 | 1.75 | 0 | 0.1 | 1.19 | 1.18 | 2.65 | 4.66E-02 | 4.80 | 8.72E-03 | 1418 |
| Cluster-40555.189432 | 4.32 | 5.31 | 25.3 | 29.94 | 4.17 | 3.12 | 24.94 | 26.12 | 2.66 | 3.78E-15 | 2.88 | 5.07E-14 | 1373 |
| Cluster-40555.162181 | 0.4 | 0.05 | 1.36 | 1.11 | 0.17 | 0.16 | 1.5 | 1.13 | 2.66 | 1.85E-03 | 3.08 | 6.78E-05 | 3639 |
| Cluster-40555.204114 | 2.53 | 1.26 | 11.52 | 9.91 | 1.05 | 0.97 | 14.71 | 17.06 | 2.66 | 7.79E-09 | 4.05 | 4.46E-20 | 1539 |
| Cluster-40555.230151 | 0.58 | 1.18 | 5.24 | 5.12 | 1.72 | 1.25 | 5.84 | 4.83 | 2.67 | 2.18E-03 | 1.91 | 2.02E-02 | 1077 |
| Cluster-40555.194190 | 2.09 | 2.27 | 11.93 | 13.18 | 0.51 | 0.85 | 10.95 | 11.3 | 2.67 | 8.66E-10 | 4.08 | 2.63E-16 | 1494 |
| Cluster-40555.245532 | 0.48 | 0.46 | 2.49 | 2.91 | 0.19 | 0.16 | 2.62 | 2.05 | 2.67 | 7.65E-03 | 3.79 | 2.80E-04 | 1477 |
| Cluster-40555.191174 | 3.04 | 4.3 | 18.91 | 23.57 | 6.08 | 6.2 | 15.07 | 14.9 | 2.67 | 1.94E-11 | 1.35 | 8.07E-04 | 2065 |
| Cluster-40555.190820 | 1.01 | 0.57 | 5.61 | 3.49 | 3.94 | 3.67 | 7.91 | 6.42 | 2.67 | 1.50E-04 | 0.98 | 3.39E-02 | 3892 |
| Cluster-40555.186828 | 15.23 | 10.42 | 75.46 | 71.91 | 3.17 | 3.05 | 38.61 | 36.19 | 2.67 | 4.23E-17 | 3.65 | 5.88E-17 | 783 |
| Cluster-40555.195298 | 4.2 | 3.64 | 22.24 | 23.06 | 4.16 | 2.57 | 16.79 | 18 | 2.68 | 2.33E-11 | 2.44 | 1.03E-07 | 1102 |
| Cluster-40555.189086 | 22.45 | 24.73 | 136.38 | 138.23 | 0.96 | 1.42 | 28.07 | 33 | 2.68 | 3.79E-18 | 4.75 | 4.44E-15 | 578 |

| | | | | | | | | | | | | | |
|---|---|---|---|---|---|---|---|---|---|---|---|---|---|
| Cluster-40555.180848 | 4.2 | 3.9 | 24.35 | 22.62 | 0.42 | 1.37 | 17.08 | 14.55 | 2.68 | 3.93E-04 | 4.16 | 1.23E-05 | 484 |
| Cluster-40555.216698 | 1.3 | 4.1 | 17.57 | 14.59 | 2.35 | 2.54 | 10.77 | 10.11 | 2.68 | 1.71E-05 | 2.13 | 3.72E-03 | 741 |
| Cluster-40555.195506 | 4.75 | 2.91 | 18.73 | 25.41 | 16.12 | 11.25 | 25.01 | 28.59 | 2.69 | 3.59E-07 | 1.05 | 1.11E-02 | 1852 |
| Cluster-40555.201335 | 15.95 | 10.41 | 82.72 | 70.1 | 70.31 | 59.66 | 130.46 | 131.68 | 2.69 | 2.16E-17 | 1.08 | 3.87E-03 | 787 |
| Cluster-40555.195635 | 0.34 | 0.12 | 1.01 | 1.59 | 0.46 | 0 | 2.06 | 1.96 | 2.69 | 1.47E-03 | 3.24 | 4.70E-07 | 3684 |
| Cluster-40555.183323 | 0.56 | 0.8 | 4.18 | 3.96 | 0.29 | 0.62 | 5.37 | 3.29 | 2.69 | 1.35E-03 | 3.26 | 5.02E-05 | 1351 |
| Cluster-40555.193696 | 0.47 | 0 | 1.47 | 1.26 | 0.93 | 0.88 | 2.61 | 2.39 | 2.69 | 6.83E-03 | 1.52 | 3.25E-02 | 2678 |
| Cluster-40555.137688 | 4.67 | 3.93 | 27.55 | 22.68 | 2.67 | 2.64 | 13.21 | 9.46 | 2.69 | 6.50E-06 | 2.15 | 1.64E-02 | 585 |
| Cluster-40555.240280 | 0.2 | 0.27 | 1.42 | 1.44 | 0.17 | 0.13 | 1.01 | 1.38 | 2.69 | 2.23E-02 | 3.04 | 1.19E-02 | 2136 |
| Cluster-40555.191125 | 13.62 | 27.2 | 120.32 | 123.35 | 20.68 | 20.79 | 41.49 | 42.3 | 2.70 | 3.64E-18 | 1.08 | 4.43E-02 | 621 |
| Cluster-40555.191624 | 7.74 | 5.57 | 38.15 | 38.98 | 18.5 | 19.67 | 45.39 | 40.34 | 2.70 | 3.31E-09 | 1.23 | 1.33E-02 | 621 |
| Cluster-40555.179526 | 0.32 | 0.3 | 1.49 | 2.15 | 0.04 | 0.04 | 1.36 | 0.95 | 2.70 | 4.22E-02 | 4.90 | 7.78E-03 | 1464 |
| Cluster-40555.157317 | 0.54 | 0.42 | 2.72 | 2.74 | 0 | 0 | 3.59 | 3.84 | 2.70 | 1.21E-02 | Inf | 1.67E-11 | 1362 |
| Cluster-40555.190269 | 3.54 | 2.63 | 15.13 | 20.77 | 5.89 | 6.61 | 24.83 | 25.29 | 2.70 | 6.59E-07 | 2.06 | 9.93E-07 | 998 |
| Cluster-40555.198905 | 0.66 | 0.61 | 3.77 | 3.79 | 0.02 | 0 | 5.44 | 5.85 | 2.70 | 2.83E-05 | 8.90 | 1.01E-24 | 2077 |
| Cluster-40555.170459 | 3.41 | 0.15 | 9.94 | 10.42 | 1.13 | 0.81 | 7.02 | 8.14 | 2.70 | 9.42E-06 | 3.02 | 2.01E-05 | 969 |
| Cluster-40555.194943 | 32.32 | 24.01 | 171.4 | 136.11 | 0 | 2.03 | 144.07 | 150.7 | 2.71 | 7.30E-03 | 6.67 | 3.08E-06 | 260 |
| Cluster-40555.216712 | 0.86 | 1.17 | 5.92 | 6.02 | 0 | 0.47 | 3.77 | 2.62 | 2.71 | 2.05E-04 | 3.75 | 1.52E-04 | 1189 |
| Cluster-40555.231354 | 1.35 | 0.49 | 4.71 | 6 | 0.43 | 0.17 | 3.64 | 3.68 | 2.71 | 7.85E-08 | 3.69 | 5.93E-09 | 2228 |
| Cluster-40555.185037 | 4.39 | 3.21 | 21.05 | 23.79 | 6.15 | 4.71 | 20.29 | 22.09 | 2.71 | 7.77E-08 | 2.04 | 1.03E-04 | 747 |
| Cluster-40555.191156 | 13.81 | 14.53 | 82.95 | 84.86 | 5.49 | 4.19 | 56.71 | 39.3 | 2.71 | 4.88E-08 | 3.37 | 4.66E-07 | 398 |
| Cluster-40555.206010 | 2.7 | 0 | 7.92 | 7.69 | 4.57 | 2.76 | 8.28 | 8.2 | 2.72 | 1.22E-07 | 1.24 | 3.37E-02 | 1571 |
| Cluster-40555.206773 | 1.07 | 0.93 | 6.99 | 4.98 | 1.02 | 1 | 2.92 | 3.24 | 2.72 | 8.26E-10 | 1.68 | 2.44E-03 | 3306 |
| Cluster-40555.190282 | 330.61 | 189.59 | 1329.05 | 1393.3 | 621.29 | 636.32 | 2267.6 | 1478.26 | 2.72 | 5.07E-09 | 1.55 | 1.95E-02 | 231 |
| Cluster-40555.165403 | 4.06 | 5.11 | 32.21 | 23.22 | 2.07 | 1.41 | 21.85 | 13.5 | 2.73 | 5.81E-07 | 3.41 | 2.24E-05 | 625 |
| Cluster-40555.220238 | 1.22 | 0.62 | 6.12 | 4.84 | 0.28 | 0.57 | 4.67 | 7.6 | 2.73 | 1.65E-02 | 3.89 | 3.17E-04 | 776 |
| Cluster-40555.146461 | 1.13 | 2.51 | 12.92 | 9.44 | 1 | 0.35 | 6.21 | 10.28 | 2.73 | 9.06E-04 | 3.71 | 9.00E-04 | 690 |
| Cluster-40555.198638 | 1.12 | 0.7 | 6.19 | 4.82 | 0.31 | 0 | 3.1 | 3.58 | 2.73 | 2.49E-05 | 4.63 | 1.70E-07 | 1532 |
| Cluster-40555.185186 | 5.44 | 4.35 | 32.4 | 26.72 | 2.12 | 1.79 | 16.67 | 12.14 | 2.74 | 1.88E-04 | 2.91 | 7.28E-03 | 450 |
| Cluster-40555.187352 | 39.74 | 66.05 | 324.36 | 324.04 | 125.32 | 130.28 | 309.13 | 320 | 2.74 | 1.42E-33 | 1.37 | 1.05E-05 | 2099 |
| Cluster-40555.188037 | 64.89 | 128.28 | 608.85 | 581.32 | 28.89 | 20.69 | 144.07 | 211.83 | 2.74 | 1.41E-32 | 2.92 | 6.73E-05 | 995 |
| Cluster-40555.171117 | 0.41 | 0.33 | 2.19 | 2.43 | 0 | 0.17 | 1.59 | 1.88 | 2.75 | 1.77E-02 | 4.38 | 9.23E-04 | 1424 |

| | | | | | | | | | | | | | |
|---|---|---|---|---|---|---|---|---|---|---|---|---|---|
| Cluster-40555.196623 | 8.24 | 4.36 | 44.35 | 32.01 | 10.87 | 7.93 | 42.75 | 45.3 | 2.75 | 2.68E-10 | 2.30 | 1.70E-12 | 1881 |
| Cluster-40555.177591 | 110.91 | 59.24 | 552.59 | 360.57 | 9.65 | 14.9 | 520.57 | 289.6 | 2.75 | 1.96E-04 | 5.01 | 1.33E-04 | 239 |
| Cluster-40555.202807 | 1.11 | 3.73 | 13.61 | 16.56 | 8.43 | 4.34 | 22.91 | 18.48 | 2.75 | 4.15E-10 | 1.77 | 1.97E-07 | 3110 |
| Cluster-40555.188590 | 11.9 | 13.09 | 65.61 | 87.55 | 54.76 | 43.85 | 100.92 | 116.28 | 2.76 | 2.70E-08 | 1.21 | 5.09E-04 | 1162 |
| Cluster-40555.185608 | 1.29 | 3.05 | 12.87 | 14.22 | 3.78 | 4.47 | 13.17 | 13.25 | 2.76 | 1.02E-14 | 1.74 | 6.29E-06 | 2276 |
| Cluster-40555.180838 | 3.6 | 5.28 | 26.75 | 28.44 | 13.38 | 9.82 | 24.12 | 25.84 | 2.77 | 1.19E-19 | 1.18 | 2.52E-03 | 1942 |
| Cluster-40555.187592 | 1.34 | 1.52 | 9.71 | 7.98 | 0.03 | 1.2 | 3.93 | 3.7 | 2.77 | 2.17E-12 | 2.66 | 4.13E-07 | 2660 |
| Cluster-40555.207279 | 1.55 | 0.6 | 6.6 | 6.68 | 1.32 | 1.63 | 5.72 | 4.29 | 2.77 | 1.76E-04 | 1.84 | 3.24E-02 | 1059 |
| Cluster-40555.182224 | 6.77 | 9.48 | 49.11 | 52.29 | 37.13 | 35.49 | 73.26 | 88.46 | 2.77 | 1.09E-26 | 1.23 | 2.31E-04 | 2338 |
| Cluster-40555.202497 | 0.4 | 1 | 3.63 | 5.29 | 0.24 | 0.46 | 5.13 | 6.33 | 2.77 | 3.12E-03 | 4.16 | 2.76E-07 | 1061 |
| Cluster-40555.175287 | 26.63 | 69.36 | 333.81 | 275.68 | 7.82 | 14.35 | 60.35 | 74.06 | 2.77 | 2.43E-11 | 2.60 | 2.95E-03 | 317 |
| Cluster-40555.190367 | 331.31 | 440.06 | 2241.61 | 2561.53 | 1326.5 | 1211.13 | 1997.88 | 2232.95 | 2.77 | 4.51E-26 | 0.80 | 3.74E-02 | 629 |
| Cluster-40555.136737 | 0.12 | 1.46 | 5 | 5.4 | 1.36 | 0.86 | 6.27 | 6.71 | 2.78 | 4.73E-03 | 2.60 | 9.34E-04 | 940 |
| Cluster-40555.188709 | 327.24 | 535.6 | 2469.49 | 2938.68 | 895.99 | 742.64 | 2520.13 | 2649.16 | 2.78 | 1.66E-16 | 1.73 | 5.02E-09 | 1503 |
| Cluster-40555.228463 | 0.93 | 1.18 | 5.5 | 7.68 | 2.23 | 3.05 | 12.44 | 11.87 | 2.78 | 1.97E-04 | 2.26 | 2.04E-05 | 1040 |
| Cluster-40555.189949 | 0.87 | 1.17 | 6.98 | 5.84 | 2.84 | 2.47 | 7.84 | 5.99 | 2.78 | 1.39E-11 | 1.44 | 7.29E-04 | 3226 |
| Cluster-40555.203505 | 0.66 | 0.5 | 3.26 | 3.9 | 1.25 | 1.38 | 4.9 | 4.18 | 2.78 | 8.57E-08 | 1.85 | 9.38E-05 | 3129 |
| Cluster-40555.204061 | 5.1 | 4.05 | 28.37 | 28.54 | 2.25 | 2.92 | 14.2 | 18.78 | 2.78 | 8.08E-16 | 2.73 | 1.87E-07 | 1301 |
| Cluster-40555.203865 | 0 | 1.17 | 4.14 | 3.5 | 1.77 | 2.78 | 4.39 | 4.93 | 2.78 | 1.93E-08 | 1.10 | 1.96E-02 | 4397 |
| Cluster-40555.189861 | 1.11 | 1.42 | 8.16 | 7.67 | 1.67 | 1.43 | 4.09 | 4.3 | 2.78 | 6.83E-09 | 1.50 | 2.63E-02 | 1875 |
| Cluster-40555.201244 | 1.06 | 4.47 | 17.24 | 18.27 | 1.51 | 1.22 | 16.96 | 9.45 | 2.79 | 1.12E-05 | 3.34 | 1.24E-03 | 672 |
| Cluster-40555.192702 | 0.89 | 0.28 | 3.14 | 4.05 | 1.04 | 0.77 | 7.47 | 5.69 | 2.79 | 2.92E-02 | 2.90 | 3.59E-04 | 891 |
| Cluster-40555.204286 | 0.77 | 0.56 | 4.37 | 3.71 | 2.58 | 1.79 | 5.37 | 7.5 | 2.79 | 2.25E-03 | 1.64 | 1.96E-02 | 1224 |
| Cluster-40555.170507 | 0 | 1.33 | 3.75 | 4.83 | 0 | 0 | 2.57 | 1.75 | 2.79 | 3.45E-05 | Inf | 1.60E-11 | 2146 |
| Cluster-40555.160204 | 2.56 | 3.77 | 19.66 | 20.49 | 13.45 | 11.52 | 23.53 | 24.05 | 2.79 | 7.98E-19 | 1.00 | 1.34E-02 | 2317 |
| Cluster-40555.224290 | 1.15 | 0.31 | 4.68 | 4.47 | 0.61 | 0.6 | 3.54 | 4.48 | 2.80 | 4.98E-06 | 2.80 | 9.65E-06 | 1887 |
| Cluster-40555.199775 | 0.9 | 1.32 | 8.09 | 6.28 | 6 | 7.12 | 12.55 | 10.88 | 2.81 | 2.73E-12 | 0.90 | 4.63E-02 | 3119 |
| Cluster-40555.192492 | 0.18 | 0.39 | 1.36 | 2.26 | 0.01 | 0.01 | 0.71 | 0.75 | 2.81 | 3.46E-03 | 5.82 | 6.27E-06 | 3801 |
| Cluster-40555.187393 | 51.89 | 101.93 | 526.35 | 469.49 | 99.09 | 117.3 | 184.7 | 233.43 | 2.81 | 4.41E-33 | 1.01 | 1.64E-02 | 953 |
| Cluster-40555.190066 | 12.96 | 15.57 | 87.13 | 95.04 | 0.3 | 0.16 | 10.46 | 6.51 | 2.82 | 4.97E-16 | 5.29 | 1.27E-05 | 583 |
| Cluster-40555.187498 | 0.5 | 0.74 | 4.01 | 4 | 0.54 | 0.26 | 3.76 | 3.46 | 2.83 | 1.50E-05 | 3.24 | 1.15E-06 | 1969 |
| Cluster-40555.191600 | 3.16 | 2.36 | 18.71 | 16.63 | 7.34 | 5.93 | 16.61 | 16.97 | 2.83 | 2.40E-11 | 1.41 | 2.60E-03 | 1235 |

| | | | | | | | | | | | | | |
|---|---|---|---|---|---|---|---|---|---|---|---|---|---|
| Cluster-40555.195461 | 0.52 | 0.49 | 3.06 | 3.48 | 2.19 | 0.87 | 15.8 | 12.86 | 2.83 | 3.07E-04 | 3.31 | 3.58E-15 | 1733 |
| Cluster-40555.172367 | 1.17 | 2.86 | 14.47 | 12.11 | 3.45 | 3 | 8.61 | 11.35 | 2.84 | 1.08E-09 | 1.70 | 1.25E-03 | 1340 |
| Cluster-40555.140680 | 1.3 | 0.69 | 6.62 | 6.05 | 0.97 | 1.7 | 3.53 | 4.19 | 2.84 | 4.68E-07 | 1.58 | 3.17E-02 | 1679 |
| Cluster-40555.206030 | 0.25 | 0.26 | 1.77 | 1.58 | 0.71 | 0.78 | 3.04 | 3.24 | 2.84 | 2.05E-03 | 2.13 | 4.16E-04 | 2556 |
| Cluster-40555.254341 | 4.19 | 1.49 | 17.34 | 18.4 | 3.69 | 4.42 | 23.68 | 22.91 | 2.84 | 1.65E-03 | 2.59 | 5.12E-04 | 473 |
| Cluster-40555.198527 | 1.68 | 1.8 | 11.29 | 11.46 | 1.25 | 0.99 | 6.44 | 4.39 | 2.85 | 1.89E-10 | 2.33 | 1.10E-04 | 1577 |
| Cluster-40555.193624 | 25.11 | 22.76 | 149.44 | 162.77 | 25.43 | 28.17 | 111.27 | 142.85 | 2.85 | 2.30E-31 | 2.31 | 1.76E-07 | 1304 |
| Cluster-40555.176390 | 0.93 | 0.53 | 4.86 | 4.59 | 0.03 | 0 | 9.95 | 9.41 | 2.85 | 1.11E-05 | 9.34 | 1.57E-31 | 1707 |
| Cluster-40555.216653 | 0.67 | 0 | 2.33 | 1.95 | 0 | 0.33 | 3.22 | 1.72 | 2.85 | 6.59E-06 | 3.91 | 5.17E-04 | 3523 |
| Cluster-40555.225556 | 2.53 | 2.86 | 17.07 | 18.41 | 1.81 | 2.34 | 13.85 | 9.99 | 2.86 | 6.87E-08 | 2.58 | 2.83E-05 | 835 |
| Cluster-40555.189421 | 20.54 | 29.28 | 175.24 | 156.91 | 11.1 | 7.33 | 43.75 | 49.24 | 2.87 | 1.70E-32 | 2.41 | 3.84E-13 | 1601 |
| Cluster-40555.184769 | 1.29 | 0.66 | 6.66 | 6.17 | 0.62 | 1.68 | 5.72 | 6.73 | 2.87 | 1.24E-04 | 2.48 | 4.06E-04 | 1089 |
| Cluster-40555.188258 | 10.78 | 9.18 | 70.1 | 62.24 | 47.07 | 46.97 | 108.69 | 83.5 | 2.87 | 3.09E-31 | 1.09 | 1.07E-03 | 2757 |
| Cluster-40555.142738 | 0.26 | 0.13 | 0.89 | 1.59 | 0.22 | 0 | 1.38 | 1.28 | 2.87 | 4.48E-02 | 3.76 | 4.36E-03 | 1809 |
| Cluster-40555.169424 | 0.63 | 1.66 | 7.95 | 7.49 | 0.17 | 0.48 | 7.43 | 8.77 | 2.87 | 7.00E-07 | 4.71 | 7.31E-15 | 1404 |
| Cluster-40555.193126 | 2.93 | 3.52 | 22.5 | 20.62 | 4.92 | 5.3 | 11.8 | 14.68 | 2.88 | 1.15E-17 | 1.45 | 7.21E-04 | 1838 |
| Cluster-40555.187300 | 56.66 | 68.42 | 412.75 | 425.16 | 154.31 | 144.45 | 290.79 | 297.49 | 2.88 | 8.90E-34 | 1.04 | 2.88E-03 | 837 |
| Cluster-40555.204836 | 2.01 | 1.31 | 11.64 | 10.48 | 3.77 | 3.09 | 10.84 | 10.96 | 2.88 | 8.14E-06 | 1.74 | 7.02E-03 | 890 |
| Cluster-40555.211983 | 0.63 | 0.13 | 2.08 | 3.01 | 0.18 | 0.63 | 2.51 | 3.98 | 2.88 | 2.99E-02 | 3.02 | 3.78E-03 | 1090 |
| Cluster-40555.267343 | 0.2 | 0.2 | 1.46 | 1.31 | 1.14 | 0.91 | 3.18 | 2.91 | 2.88 | 9.03E-03 | 1.63 | 1.69E-02 | 2362 |
| Cluster-40555.178837 | 4.95 | 6.08 | 33.01 | 41.5 | 17.37 | 14.6 | 37.49 | 41.7 | 2.89 | 2.67E-12 | 1.38 | 3.57E-04 | 1125 |
| Cluster-40555.161541 | 1.11 | 1.56 | 9.62 | 8.65 | 1.73 | 1.92 | 4.76 | 4.49 | 2.90 | 1.75E-11 | 1.41 | 1.96E-02 | 2112 |
| Cluster-40555.160379 | 5.16 | 6.72 | 38.1 | 42.47 | 7.8 | 7.71 | 27.65 | 22.12 | 2.90 | 2.51E-17 | 1.74 | 3.69E-05 | 1031 |
| Cluster-40555.175138 | 0.52 | 0.56 | 4.2 | 3.15 | 0.68 | 1.37 | 4.73 | 3.8 | 2.90 | 2.13E-05 | 2.10 | 2.89E-04 | 2040 |
| Cluster-40555.210374 | 0.23 | 0.28 | 1.85 | 1.65 | 0.21 | 0 | 1.61 | 3.05 | 2.90 | 1.69E-03 | 4.59 | 2.78E-03 | 2466 |
| Cluster-40555.165480 | 1.43 | 0 | 4.48 | 4.96 | 0 | 0 | 4.6 | 5.5 | 2.90 | 1.35E-05 | Inf | 7.45E-18 | 1563 |
| Cluster-40555.200270 | 0.84 | 0.74 | 5.78 | 4.96 | 0.85 | 0.99 | 9.55 | 9.63 | 2.91 | 3.70E-12 | 3.45 | 6.62E-20 | 3638 |
| Cluster-40555.221253 | 0.64 | 0.22 | 2.28 | 3.47 | 0.12 | 0.17 | 2.38 | 3.09 | 2.91 | 2.08E-04 | 4.24 | 3.83E-07 | 1848 |
| Cluster-40555.229428 | 4.17 | 5.01 | 25.25 | 37.4 | 14.5 | 7.96 | 48.55 | 33.99 | 2.91 | 2.70E-05 | 1.95 | 8.12E-05 | 1013 |
| Cluster-40555.231459 | 0.6 | 1.62 | 8.95 | 6.44 | 3.76 | 4.37 | 22.67 | 19.41 | 2.92 | 8.89E-05 | 2.42 | 4.34E-08 | 1005 |
| Cluster-40555.170851 | 2.65 | 6.5 | 27.52 | 36.37 | 1 | 0.71 | 27.23 | 18.84 | 2.92 | 1.74E-06 | 4.86 | 1.07E-09 | 499 |
| Cluster-40555.238180 | 0.88 | 0 | 3.1 | 2.82 | 0.07 | 0.02 | 1.72 | 2.61 | 2.92 | 1.09E-03 | 5.38 | 5.37E-06 | 1553 |

| | | | | | | | | | | | | | |
|---|---|---|---|---|---|---|---|---|---|---|---|---|---|
| Cluster-40555.163251 | 0.63 | 1.63 | 8.78 | 7.14 | 0.17 | 0.45 | 4.61 | 3.34 | 2.92 | 8.70E-07 | 3.69 | 5.09E-06 | 1339 |
| Cluster-40555.151627 | 1.84 | 1.26 | 10.6 | 10.75 | 0.97 | 0.82 | 9.55 | 8.15 | 2.94 | 6.01E-13 | 3.37 | 2.02E-13 | 2019 |
| Cluster-40555.191126 | 5.92 | 7.77 | 46.7 | 49.05 | 17.99 | 16.16 | 53.46 | 44.32 | 2.94 | 2.27E-28 | 1.58 | 1.06E-06 | 2201 |
| Cluster-40555.142375 | 1.04 | 0.26 | 3.83 | 4.93 | 0.1 | 0.53 | 6.46 | 3.4 | 2.94 | 3.25E-02 | 3.98 | 1.96E-03 | 728 |
| Cluster-40555.113107 | 0.09 | 0.39 | 1.47 | 1.96 | 0 | 0 | 1.02 | 1.57 | 2.95 | 4.96E-02 | Inf | 5.31E-04 | 1375 |
| Cluster-40555.185670 | 5.47 | 1.26 | 21.89 | 24.22 | 17.86 | 13.76 | 40.94 | 42.63 | 2.95 | 4.39E-15 | 1.47 | 6.66E-05 | 1234 |
| Cluster-40555.234029 | 2 | 0 | 7.14 | 6.41 | 1.11 | 1.31 | 6.09 | 4.95 | 2.95 | 3.88E-05 | 2.25 | 3.42E-03 | 1079 |
| Cluster-40555.188516 | 110.27 | 124 | 970.18 | 659.59 | 2.36 | 2.43 | 503 | 585.17 | 2.96 | 7.33E-08 | 7.85 | 4.07E-49 | 324 |
| Cluster-40555.211347 | 0.77 | 0.3 | 3.72 | 3.66 | 0.66 | 0.82 | 2.7 | 3.9 | 2.96 | 5.82E-07 | 2.24 | 1.27E-03 | 2447 |
| Cluster-40555.211643 | 0.48 | 1.03 | 5.31 | 5.59 | 1.54 | 2.14 | 6.04 | 5.02 | 2.96 | 2.86E-07 | 1.65 | 3.14E-03 | 1874 |
| Cluster-40555.198975 | 0 | 4.1 | 15.2 | 14.93 | 0 | 0 | 13.66 | 9.71 | 2.96 | 1.33E-13 | Inf | 3.10E-32 | 1777 |
| Cluster-40555.218574 | 1.21 | 1.59 | 11.6 | 8.28 | 1.97 | 3.45 | 9.25 | 12.8 | 2.97 | 1.73E-05 | 2.08 | 1.05E-03 | 915 |
| Cluster-40555.198935 | 0.04 | 0.87 | 3.3 | 3.23 | 1.03 | 0.88 | 3.05 | 2.3 | 2.97 | 8.36E-08 | 1.55 | 9.97E-03 | 3301 |
| Cluster-40555.163280 | 0.19 | 0.96 | 4.15 | 4.5 | 1.95 | 1.83 | 10.6 | 9.45 | 2.97 | 1.17E-03 | 2.49 | 5.07E-06 | 1161 |
| Cluster-40555.143418 | 1.14 | 0.35 | 3.49 | 6.79 | 0 | 0 | 3.57 | 1.36 | 2.97 | 2.79E-02 | Inf | 1.19E-02 | 881 |
| Cluster-40555.229806 | 1.32 | 1.1 | 8.38 | 8.66 | 3.81 | 1.6 | 8.17 | 8.92 | 2.97 | 3.93E-04 | 1.77 | 4.09E-02 | 774 |
| Cluster-40555.142376 | 0.68 | 1.25 | 6.67 | 7.3 | 0.14 | 0 | 5.52 | 3.81 | 2.97 | 3.71E-04 | 6.08 | 7.98E-07 | 896 |
| Cluster-40555.192168 | 4.4 | 0 | 19.65 | 10.86 | 0 | 0 | 6.18 | 10.1 | 2.98 | 5.92E-03 | Inf | 2.63E-11 | 2264 |
| Cluster-40555.207733 | 3.46 | 7.07 | 40.41 | 36.31 | 9.98 | 9.38 | 25.84 | 24.78 | 2.98 | 2.06E-18 | 1.45 | 5.37E-04 | 1140 |
| Cluster-40555.171798 | 1.76 | 1.19 | 10.36 | 10.58 | 6.55 | 6.2 | 30.95 | 20.78 | 2.99 | 3.63E-03 | 2.09 | 3.66E-04 | 564 |
| Cluster-40555.179142 | 0.82 | 1.12 | 8.17 | 5.72 | 0.25 | 0.18 | 5.55 | 5.03 | 2.99 | 2.20E-05 | 4.72 | 1.40E-08 | 1165 |
| Cluster-40555.195195 | 2.24 | 2.6 | 14.08 | 20.69 | 1.57 | 2.47 | 24.47 | 16.57 | 2.99 | 4.66E-05 | 3.39 | 3.48E-07 | 567 |
| Cluster-40555.202995 | 1.28 | 2.91 | 17.28 | 13.6 | 2.54 | 2.98 | 15.24 | 16 | 2.99 | 1.13E-13 | 2.57 | 2.04E-10 | 1629 |
| Cluster-40555.209566 | 1.38 | 1.11 | 9.13 | 8.81 | 6.38 | 6.83 | 12.73 | 15.31 | 2.99 | 7.34E-10 | 1.15 | 1.42E-02 | 1662 |
| Cluster-40555.207896 | 1.96 | 2.22 | 14.45 | 15.41 | 1.37 | 1.85 | 16.29 | 18.68 | 2.99 | 2.85E-03 | 3.51 | 5.93E-05 | 482 |
| Cluster-40555.186849 | 0.49 | 0.08 | 1.97 | 2.1 | 0 | 0.11 | 0.97 | 0.99 | 3.00 | 6.22E-04 | 4.28 | 1.83E-03 | 2201 |
| Cluster-40555.185502 | 3.38 | 3.21 | 24.37 | 23.37 | 3.26 | 2.04 | 16.76 | 14.45 | 3.00 | 3.76E-20 | 2.63 | 2.29E-11 | 1842 |
| Cluster-40555.193407 | 168.22 | 240.83 | 1470.66 | 1509.66 | 178.27 | 176.67 | 1055.53 | 1000.42 | 3.00 | 1.99E-33 | 2.57 | 2.49E-16 | 364 |
| Cluster-40555.206734 | 2.88 | 1.28 | 11.7 | 18.14 | 0.64 | 1.22 | 9.03 | 6.54 | 3.00 | 1.82E-04 | 3.11 | 7.61E-05 | 815 |
| Cluster-40555.152625 | 1.41 | 0.84 | 8.67 | 7.71 | 5.66 | 3.22 | 11.03 | 8.32 | 3.01 | 1.49E-07 | 1.19 | 4.79E-02 | 1350 |
| Cluster-40555.256605 | 0.27 | 0.07 | 1.17 | 1.27 | 1.06 | 0.36 | 2.28 | 2.68 | 3.01 | 9.09E-03 | 1.90 | 1.03E-02 | 2419 |
| Cluster-40555.234244 | 2.3 | 0 | 8 | 7.89 | 1.74 | 3.47 | 19.63 | 30.73 | 3.01 | 2.16E-02 | 3.32 | 1.69E-04 | 538 |

| | | | | | | | | | | | | | |
|---|---|---|---|---|---|---|---|---|---|---|---|---|---|
| Cluster-40555.171853 | 2.46 | 1.28 | 14.22 | 12.64 | 0.51 | 0.51 | 9.71 | 9.76 | 3.01 | 2.92E-12 | 4.34 | 3.74E-16 | 1503 |
| Cluster-40555.156063 | 0.45 | 0.35 | 3.25 | 2.6 | 0.8 | 1.14 | 4.37 | 4.58 | 3.01 | 7.73E-10 | 2.27 | 3.45E-08 | 4588 |
| Cluster-40555.216790 | 1.94 | 6.34 | 32.86 | 29.84 | 2.06 | 4.48 | 37.27 | 46.22 | 3.02 | 8.20E-11 | 3.72 | 4.37E-17 | 726 |
| Cluster-40555.199826 | 0 | 0.45 | 1.67 | 1.7 | 0.14 | 0 | 1.69 | 1.44 | 3.02 | 1.34E-02 | 4.49 | 2.20E-04 | 1799 |
| Cluster-40555.157780 | 4.3 | 1.77 | 21.58 | 22.21 | 2.1 | 1.79 | 14.99 | 16.75 | 3.02 | 8.73E-09 | 3.10 | 3.05E-07 | 735 |
| Cluster-40555.188497 | 0.53 | 0.3 | 2.51 | 3.72 | 0 | 0 | 1.81 | 0.9 | 3.02 | 5.34E-04 | Inf | 9.95E-05 | 1525 |
| Cluster-40555.210277 | 1.75 | 1.56 | 12.94 | 11.55 | 2.62 | 2.95 | 13.38 | 10.72 | 3.02 | 1.29E-13 | 2.17 | 1.52E-07 | 1852 |
| Cluster-40555.188630 | 126.21 | 263.02 | 1435.81 | 1478.72 | 732.41 | 723.2 | 1403.62 | 1288.69 | 3.03 | 7.14E-42 | 0.95 | 5.53E-03 | 1826 |
| Cluster-40555.95689 | 0.6 | 0.37 | 3.74 | 3.16 | 0.51 | 1.24 | 4.69 | 2.81 | 3.03 | 5.78E-03 | 2.17 | 2.17E-02 | 1111 |
| Cluster-40555.208911 | 1.06 | 0.87 | 7.54 | 6.66 | 1.19 | 1.47 | 5.46 | 6.99 | 3.03 | 3.04E-06 | 2.28 | 4.66E-04 | 1248 |
| Cluster-40555.174951 | 0.12 | 0.13 | 0.9 | 0.98 | 0.25 | 0.22 | 1.28 | 1.77 | 3.03 | 4.16E-02 | 2.80 | 3.34E-03 | 2322 |
| Cluster-40555.216039 | 0.21 | 0.56 | 2.88 | 2.89 | 0.12 | 0 | 2.32 | 2.47 | 3.04 | 2.56E-02 | 5.42 | 5.27E-04 | 1036 |
| Cluster-40555.229386 | 0.06 | 0.62 | 2.39 | 2.65 | 0.11 | 0.17 | 2.41 | 3.71 | 3.04 | 2.02E-02 | 4.61 | 2.66E-05 | 1186 |
| Cluster-40555.219532 | 0.81 | 0.64 | 5.03 | 5.6 | 1.08 | 0.95 | 3.34 | 5.14 | 3.04 | 1.19E-07 | 2.13 | 1.09E-02 | 1896 |
| Cluster-40555.234990 | 0.7 | 2.9 | 12.89 | 14.64 | 1.08 | 0.41 | 6.88 | 8.32 | 3.04 | 5.47E-06 | 3.44 | 1.23E-04 | 744 |
| Cluster-40555.198933 | 0 | 1.1 | 4.71 | 3.81 | 0 | 0 | 4.56 | 4.62 | 3.04 | 7.80E-05 | Inf | 2.85E-16 | 1549 |
| Cluster-40555.163384 | 3.25 | 1.17 | 13.62 | 19.55 | 0.99 | 0 | 10.17 | 9.03 | 3.04 | 7.96E-04 | 4.55 | 9.75E-04 | 482 |
| Cluster-40555.220103 | 0.98 | 0.39 | 4.98 | 5.21 | 3.69 | 1.32 | 6.39 | 7.88 | 3.05 | 2.77E-05 | 1.60 | 1.35E-02 | 1347 |
| Cluster-40555.200481 | 0.91 | 0 | 2.75 | 3.86 | 0 | 0 | 1.09 | 2.38 | 3.05 | 3.21E-05 | Inf | 4.63E-04 | 2573 |
| Cluster-40555.180476 | 0.73 | 1.29 | 7.04 | 8.38 | 0 | 0.3 | 5.37 | 5.97 | 3.05 | 1.23E-11 | 5.24 | 5.17E-19 | 2199 |
| Cluster-40555.188305 | 512.74 | 837.54 | 4988.57 | 5276.77 | 2947.23 | 2853.96 | 5317.77 | 6364.34 | 3.05 | 2.45E-43 | 1.08 | 1.04E-03 | 1433 |
| Cluster-40555.166444 | 1.72 | 1.27 | 10.03 | 12.27 | 0 | 0 | 8.22 | 7.77 | 3.06 | 1.31E-04 | Inf | 2.65E-09 | 678 |
| Cluster-40555.188884 | 3.13 | 3.53 | 26.4 | 24.05 | 3.91 | 3.28 | 18.37 | 15.38 | 3.06 | 4.37E-23 | 2.30 | 4.20E-10 | 2143 |
| Cluster-40555.169926 | 0.32 | 0.56 | 3.87 | 3.01 | 0.22 | 0.42 | 3.99 | 4.45 | 3.06 | 1.65E-02 | 3.83 | 1.30E-04 | 979 |
| Cluster-40555.165296 | 3.11 | 2.13 | 22.84 | 16.98 | 9.88 | 9.83 | 23.65 | 27.68 | 3.07 | 3.94E-15 | 1.45 | 2.06E-04 | 1438 |
| Cluster-40555.188558 | 203.02 | 332.72 | 2062.59 | 2065.26 | 1061.44 | 1007.25 | 1636.25 | 1876.04 | 3.07 | 5.54E-43 | 0.83 | 2.58E-02 | 1239 |
| Cluster-40555.181573 | 0.5 | 0.39 | 2.72 | 4.01 | 0.4 | 0.35 | 1.94 | 1.78 | 3.07 | 1.73E-05 | 2.39 | 4.22E-03 | 2359 |
| Cluster-40555.213834 | 0.57 | 1.35 | 6.88 | 8.03 | 0.2 | 0.11 | 1.2 | 2.23 | 3.07 | 8.99E-08 | 3.50 | 2.07E-02 | 1448 |
| Cluster-40555.180413 | 3.77 | 2.52 | 23.8 | 24.02 | 9.7 | 7.25 | 22.32 | 20.75 | 3.08 | 9.71E-11 | 1.41 | 7.62E-03 | 813 |
| Cluster-40555.195288 | 0.62 | 0.06 | 2.49 | 2.62 | 0.21 | 0.45 | 2.4 | 2.77 | 3.08 | 2.27E-02 | 2.92 | 1.76E-02 | 1087 |
| Cluster-40555.182358 | 0.39 | 0 | 1.49 | 1.46 | 1.58 | 0.43 | 2.77 | 3.29 | 3.08 | 7.20E-04 | 1.70 | 1.23E-02 | 2802 |
| Cluster-40555.149987 | 0.53 | 0.38 | 3.19 | 3.64 | 0.47 | 0.1 | 3.15 | 2.68 | 3.08 | 3.35E-03 | 3.42 | 1.82E-03 | 1157 |

| | | | | | | | | | | | | | |
|---|---|---|---|---|---|---|---|---|---|---|---|---|---|
| Cluster-40555.185912 | 0.52 | 0.5 | 4.44 | 3.51 | 0.74 | 0.62 | 2.38 | 3.3 | 3.08 | 4.42E-10 | 2.14 | 3.20E-04 | 3445 |
| Cluster-40555.185721 | 0.93 | 1.89 | 12.56 | 9.15 | 6.85 | 11.51 | 20.63 | 19.58 | 3.08 | 8.06E-07 | 1.18 | 1.83E-02 | 994 |
| Cluster-40555.212215 | 0.21 | 0.51 | 2.96 | 2.67 | 0.5 | 0.63 | 3.46 | 2.22 | 3.08 | 1.03E-02 | 2.38 | 2.71E-02 | 1197 |
| Cluster-40555.136963 | 0.42 | 0.35 | 2.93 | 3.1 | 0.09 | 0.1 | 1.74 | 1.43 | 3.09 | 3.54E-03 | 4.13 | 8.17E-03 | 1280 |
| Cluster-40555.146048 | 0.12 | 0.28 | 1.13 | 1.88 | 0.07 | 0.22 | 1.37 | 1.37 | 3.09 | 4.36E-02 | 3.34 | 2.40E-02 | 1471 |
| Cluster-40555.126198 | 1.11 | 1.29 | 10.18 | 8.46 | 2.77 | 2.62 | 8.59 | 9.21 | 3.09 | 7.38E-09 | 1.78 | 8.02E-04 | 1400 |
| Cluster-40555.190175 | 5.21 | 3.04 | 32.19 | 30.96 | 24.61 | 24.36 | 56.64 | 50.71 | 3.09 | 1.60E-23 | 1.20 | 6.58E-04 | 1717 |
| Cluster-40555.212921 | 2.64 | 2.48 | 19.96 | 19.64 | 7.4 | 9.87 | 16.52 | 21.03 | 3.09 | 8.20E-19 | 1.18 | 1.29E-02 | 1802 |
| Cluster-40555.199869 | 2.11 | 2.32 | 18.48 | 16.01 | 6.52 | 6.68 | 14.13 | 15.97 | 3.09 | 2.01E-25 | 1.26 | 6.27E-04 | 3574 |
| Cluster-40555.177212 | 88.69 | 116.28 | 812.97 | 732.02 | 465.87 | 526.87 | 979.78 | 1034.29 | 3.09 | 3.89E-17 | 1.02 | 2.18E-02 | 281 |
| Cluster-40555.232689 | 0.21 | 0.08 | 1.41 | 0.9 | 0.33 | 0.63 | 1.89 | 1.98 | 3.10 | 2.59E-03 | 2.08 | 2.21E-03 | 3146 |
| Cluster-40555.240402 | 0 | 0.37 | 1.28 | 1.73 | 0.09 | 0.15 | 0.86 | 0.89 | 3.10 | 9.44E-04 | 2.85 | 1.92E-02 | 2743 |
| Cluster-40555.177510 | 0.13 | 0.49 | 1.77 | 3.09 | 0.13 | 0.04 | 3.51 | 3.36 | 3.11 | 7.15E-03 | 5.48 | 2.21E-08 | 1416 |
| Cluster-40555.170935 | 0.34 | 0.13 | 2.03 | 1.81 | 0 | 0 | 1.63 | 0.69 | 3.11 | 2.58E-02 | Inf | 3.86E-03 | 1364 |
| Cluster-40555.186343 | 2.28 | 1.27 | 14.31 | 13.26 | 0.33 | 0.09 | 7.46 | 6.2 | 3.11 | 3.57E-07 | 5.11 | 1.25E-07 | 830 |
| Cluster-40555.238366 | 0.86 | 0.79 | 5.39 | 7.66 | 0.67 | 0.92 | 5.46 | 4.33 | 3.11 | 9.62E-05 | 2.70 | 2.27E-03 | 973 |
| Cluster-40555.175697 | 2.24 | 3.27 | 21.1 | 22.44 | 0.36 | 1.04 | 9.74 | 10.83 | 3.11 | 4.13E-10 | 3.90 | 4.09E-08 | 816 |
| Cluster-40555.202405 | 1.22 | 5.54 | 24.07 | 30.65 | 6.7 | 6.35 | 36.95 | 35.98 | 3.11 | 1.90E-04 | 2.54 | 2.01E-04 | 424 |
| Cluster-40555.166885 | 1.08 | 1.47 | 11.81 | 8.39 | 0.7 | 0.86 | 5.22 | 5.16 | 3.11 | 5.71E-04 | 2.77 | 3.11E-02 | 668 |
| Cluster-40555.202182 | 1.49 | 1.03 | 11.14 | 8.69 | 2.32 | 1.84 | 5.97 | 7.2 | 3.12 | 3.07E-13 | 1.74 | 5.40E-04 | 2058 |
| Cluster-40555.165379 | 4.03 | 2.06 | 19.79 | 27.15 | 0.8 | 0.51 | 7.56 | 8.21 | 3.12 | 5.30E-05 | 3.79 | 1.72E-02 | 468 |
| Cluster-40555.116540 | 0.26 | 0.06 | 0.91 | 1.57 | 0.09 | 0.25 | 1.56 | 1.26 | 3.12 | 3.73E-02 | 3.12 | 1.34E-02 | 1704 |
| Cluster-40555.198802 | 1.86 | 0.98 | 9.99 | 12.1 | 4.41 | 2.99 | 9.39 | 10.74 | 3.12 | 5.48E-08 | 1.52 | 1.61E-02 | 1039 |
| Cluster-40555.190349 | 8.13 | 11.39 | 76.34 | 78.76 | 1.96 | 2.13 | 16.19 | 27.35 | 3.12 | 3.59E-27 | 3.48 | 1.45E-03 | 1074 |
| Cluster-40555.158279 | 1.97 | 1.44 | 12.81 | 13.96 | 0.47 | 1.5 | 8.97 | 9.32 | 3.12 | 8.93E-06 | 3.31 | 5.42E-05 | 705 |
| Cluster-40555.211792 | 0.43 | 0.07 | 2.15 | 1.64 | 0.92 | 0.59 | 3.2 | 2.72 | 3.13 | 2.18E-03 | 2.05 | 5.50E-03 | 1973 |
| Cluster-40555.179894 | 0.51 | 0.56 | 4.05 | 4.47 | 2.26 | 0 | 6.42 | 7.82 | 3.13 | 9.30E-04 | 2.78 | 4.31E-05 | 1114 |
| Cluster-40555.198851 | 3.65 | 3.01 | 20.09 | 32.55 | 0.33 | 0.19 | 16.1 | 15.34 | 3.13 | 3.67E-04 | 6.04 | 2.76E-24 | 1115 |
| Cluster-40555.187754 | 53.77 | 32.46 | 389.97 | 256.59 | 2.17 | 0 | 180.12 | 258.78 | 3.13 | 3.45E-07 | 7.74 | 6.62E-20 | 303 |
| Cluster-40555.197628 | 0.42 | 1.16 | 5.81 | 7.1 | 0 | 0.35 | 1.89 | 1.52 | 3.14 | 8.15E-11 | 3.33 | 1.75E-04 | 2263 |
| Cluster-40555.184288 | 2.97 | 1.85 | 13.91 | 23.79 | 0.42 | 0.29 | 8.8 | 9 | 3.14 | 1.80E-03 | 4.72 | 1.09E-05 | 606 |
| Cluster-40555.211332 | 0.6 | 1.15 | 8.58 | 6.3 | 0.43 | 0 | 9.09 | 7.58 | 3.14 | 2.22E-02 | 5.23 | 4.57E-05 | 563 |

| | | | | | | | | | | | | | |
|---|---|---|---|---|---|---|---|---|---|---|---|---|---|
| Cluster-40555.303211 | 0 | 0.56 | 3.01 | 1.65 | 0 | 0 | 1.73 | 1.09 | 3.15 | 1.64E-02 | Inf | 2.91E-04 | 1347 |
| Cluster-40555.254780 | 1.75 | 0.99 | 11.4 | 10.32 | 2.64 | 4.57 | 14.2 | 14.04 | 3.15 | 5.04E-04 | 2.02 | 5.66E-03 | 623 |
| Cluster-40555.188284 | 65.48 | 112.51 | 696.47 | 747.9 | 195.43 | 157.32 | 320.84 | 316.78 | 3.15 | 4.48E-42 | 0.92 | 1.25E-02 | 889 |
| Cluster-40555.201847 | 0.27 | 0.06 | 1.45 | 1.21 | 0.54 | 0.07 | 1.49 | 1.19 | 3.15 | 4.06E-04 | 2.22 | 9.04E-03 | 3296 |
| Cluster-40555.239689 | 0 | 0.75 | 2.69 | 3.63 | 0 | 0 | 1.35 | 0.55 | 3.15 | 8.74E-04 | Inf | 1.26E-02 | 1423 |
| Cluster-40555.251598 | 0.32 | 0.3 | 3.17 | 1.87 | 4.39 | 5.46 | 11.3 | 9.04 | 3.15 | 2.22E-04 | 1.10 | 9.35E-03 | 2936 |
| Cluster-40555.190366 | 22.84 | 41.4 | 246.58 | 264.51 | 106.79 | 90.58 | 377.23 | 288.01 | 3.15 | 3.42E-04 | 1.74 | 4.72E-02 | 248 |
| Cluster-40555.188043 | 0.34 | 0.33 | 3.21 | 2.02 | 0 | 0 | 0.79 | 2.01 | 3.15 | 4.05E-02 | Inf | 2.90E-02 | 1014 |
| Cluster-40555.189334 | 63.06 | 71.14 | 548.99 | 540.16 | 12.24 | 5.48 | 49.52 | 47.09 | 3.16 | 1.03E-43 | 2.53 | 2.55E-14 | 1589 |
| Cluster-40555.69786 | 1.56 | 0.08 | 5.93 | 6.98 | 0.11 | 0 | 2.28 | 2.36 | 3.16 | 1.58E-03 | 5.71 | 1.47E-02 | 754 |
| Cluster-40555.176033 | 1.38 | 0.33 | 6.29 | 7.2 | 0.43 | 0.82 | 2.32 | 4.05 | 3.16 | 2.37E-09 | 2.40 | 4.84E-02 | 1759 |
| Cluster-40555.173247 | 0.95 | 1.4 | 9.5 | 9.64 | 3.17 | 1.84 | 10.85 | 10.39 | 3.17 | 4.86E-12 | 2.17 | 8.58E-07 | 1811 |
| Cluster-40555.135888 | 0.94 | 1.75 | 11.2 | 11.07 | 1.93 | 1.65 | 10.9 | 6.18 | 3.17 | 4.16E-04 | 2.29 | 2.09E-02 | 624 |
| Cluster-40555.183181 | 1.91 | 1.19 | 12.57 | 12.6 | 4.58 | 2.76 | 19.84 | 20.04 | 3.17 | 7.83E-12 | 2.52 | 3.96E-10 | 1385 |
| Cluster-40555.204892 | 0 | 0.57 | 2.19 | 2.73 | 0 | 0 | 1.95 | 1.52 | 3.17 | 3.70E-04 | Inf | 3.34E-08 | 1917 |
| Cluster-40555.220775 | 0.49 | 0.24 | 3.76 | 2.35 | 0 | 0 | 2.82 | 2.07 | 3.18 | 1.47E-02 | Inf | 2.17E-05 | 1046 |
| Cluster-40555.194120 | 1.07 | 1.64 | 12.48 | 10.08 | 1.64 | 2.97 | 11.52 | 10 | 3.18 | 1.25E-06 | 2.27 | 1.99E-04 | 895 |
| Cluster-40555.225208 | 0.3 | 0.28 | 2.66 | 2.08 | 2.88 | 3.18 | 6.89 | 7.62 | 3.18 | 3.19E-03 | 1.33 | 2.53E-02 | 1559 |
| Cluster-40555.115562 | 0.93 | 0.88 | 6.44 | 8.46 | 0.26 | 0.27 | 3.41 | 3.89 | 3.19 | 1.47E-05 | 3.85 | 4.29E-04 | 988 |
| Cluster-40555.195558 | 0.35 | 0.12 | 1.38 | 2.31 | 0 | 0 | 1.88 | 1.17 | 3.19 | 1.15E-03 | Inf | 1.80E-10 | 2664 |
| Cluster-40555.68861 | 0.49 | 0.46 | 3.13 | 4.66 | 0.1 | 0.36 | 4.49 | 3.61 | 3.20 | 2.35E-02 | 4.10 | 1.56E-03 | 773 |
| Cluster-98882.0 | 0.28 | 0.43 | 3.34 | 2.86 | 0 | 0.02 | 0.88 | 0.92 | 3.20 | 3.04E-03 | Inf | 2.01E-02 | 1249 |
| Cluster-40555.197039 | 1.46 | 1.53 | 11.64 | 13.45 | 7.57 | 4.44 | 15.43 | 14.28 | 3.21 | 4.65E-13 | 1.39 | 2.26E-03 | 1516 |
| Cluster-40555.169774 | 0.77 | 2.19 | 12.8 | 12.6 | 2.64 | 3.76 | 15.64 | 21.69 | 3.21 | 3.40E-15 | 2.61 | 2.27E-05 | 1871 |
| Cluster-40555.188933 | 1.85 | 1.47 | 15.78 | 12.04 | 1.87 | 1.79 | 9.32 | 7.46 | 3.21 | 7.53E-18 | 2.26 | 2.61E-07 | 2155 |
| Cluster-40555.229698 | 0.58 | 0.11 | 3.36 | 2.33 | 0.09 | 0.14 | 2.11 | 2.16 | 3.21 | 2.76E-03 | 4.31 | 4.14E-04 | 1336 |
| Cluster-40555.187894 | 5.01 | 6.12 | 42.64 | 50.91 | 19.71 | 19.08 | 35.82 | 44.62 | 3.21 | 5.48E-16 | 1.12 | 2.37E-02 | 710 |
| Cluster-40555.191555 | 0.4 | 0.31 | 3.69 | 2.29 | 0.07 | 0.06 | 1.18 | 1.72 | 3.21 | 2.37E-05 | 4.63 | 3.09E-05 | 2191 |
| Cluster-40555.159823 | 0.73 | 0.15 | 4.29 | 2.9 | 0 | 0 | 1.36 | 1.79 | 3.22 | 2.72E-03 | Inf | 1.08E-03 | 1116 |
| Cluster-40555.175752 | 0 | 0.52 | 2.06 | 2.45 | 0 | 0.41 | 1.43 | 0.94 | 3.22 | 2.70E-06 | 2.56 | 3.14E-03 | 3150 |
| Cluster-40555.199798 | 1.52 | 2.13 | 14.45 | 16.62 | 2.7 | 3.01 | 18.74 | 20.31 | 3.22 | 2.52E-08 | 2.85 | 1.76E-08 | 828 |
| Cluster-40555.181128 | 0.24 | 0 | 0.95 | 1.06 | 0 | 0 | 0.57 | 0.57 | 3.22 | 3.64E-03 | Inf | 1.95E-04 | 2995 |

| | | | | | | | | | | | | | |
|---|---|---|---|---|---|---|---|---|---|---|---|---|---|
| Cluster-40555.220597 | 0.12 | 0.03 | 0.62 | 0.66 | 0.22 | 0.51 | 1.05 | 1.19 | 3.22 | 7.32E-03 | 1.69 | 3.97E-02 | 4154 |
| Cluster-40555.186473 | 13.94 | 14.26 | 128.92 | 112.32 | 55.29 | 63.26 | 101.18 | 99.27 | 3.23 | 3.11E-37 | 0.82 | 3.85E-02 | 1511 |
| Cluster-40555.168030 | 1.21 | 0.84 | 10.47 | 7.07 | 1.06 | 0.88 | 8.12 | 6.35 | 3.23 | 1.15E-08 | 2.97 | 2.06E-11 | 2510 |
| Cluster-40555.186014 | 1.74 | 2.38 | 20.61 | 14.93 | 10.46 | 10.71 | 24.21 | 19.61 | 3.23 | 3.29E-13 | 1.11 | 6.55E-03 | 1780 |
| Cluster-40555.228476 | 0.57 | 0.85 | 6.36 | 6.03 | 3 | 0.9 | 7.81 | 9.69 | 3.24 | 2.90E-04 | 2.26 | 1.15E-03 | 920 |
| Cluster-40555.205129 | 5.03 | 4.41 | 43.18 | 37.73 | 14.27 | 14.2 | 37.99 | 35.91 | 3.24 | 5.92E-27 | 1.44 | 4.70E-05 | 1616 |
| Cluster-40555.184876 | 23.69 | 24.88 | 215.34 | 199.78 | 32 | 26.94 | 69.94 | 54.74 | 3.24 | 1.26E-29 | 1.14 | 1.42E-02 | 591 |
| Cluster-40555.187013 | 0.15 | 0.99 | 6.34 | 3.74 | 1.25 | 0.91 | 10.47 | 7.19 | 3.24 | 4.89E-04 | 3.10 | 4.61E-08 | 1235 |
| Cluster-40555.158020 | 0.13 | 0.64 | 3.68 | 3.43 | 0.28 | 1.85 | 3.43 | 3.91 | 3.24 | 1.24E-03 | 1.83 | 4.31E-02 | 1233 |
| Cluster-40555.232630 | 0.55 | 0.32 | 4.38 | 3.09 | 1.38 | 1.08 | 4.03 | 3.17 | 3.24 | 8.13E-06 | 1.61 | 2.62E-02 | 1849 |
| Cluster-40555.187117 | 15.46 | 15.71 | 136.67 | 130.9 | 22.04 | 24.92 | 86.4 | 80.72 | 3.24 | 8.87E-27 | 1.89 | 2.63E-07 | 657 |
| Cluster-40555.191304 | 4.38 | 0 | 17.52 | 18.82 | 0.4 | 0 | 4.54 | 3.84 | 3.25 | 7.97E-19 | 4.54 | 6.82E-10 | 1698 |
| Cluster-40555.219125 | 0.54 | 2.08 | 9.47 | 13.53 | 0.92 | 1.24 | 14.4 | 8.08 | 3.25 | 1.72E-04 | 3.43 | 9.58E-04 | 625 |
| Cluster-40555.173542 | 0.26 | 0.94 | 4.88 | 5.74 | 0.56 | 0.64 | 4.93 | 3.39 | 3.25 | 2.73E-07 | 2.86 | 1.67E-05 | 1670 |
| Cluster-40555.176065 | 1.06 | 1.22 | 11.33 | 8.32 | 1.22 | 1.19 | 8.24 | 10.95 | 3.25 | 8.22E-07 | 3.05 | 8.84E-07 | 992 |
| Cluster-40555.211048 | 0.95 | 0.78 | 7.03 | 7.89 | 0.48 | 0.46 | 6.38 | 6.73 | 3.25 | 1.70E-07 | 3.85 | 5.49E-09 | 1276 |
| Cluster-40555.256097 | 0.66 | 0.62 | 4.9 | 6.04 | 2.24 | 2.37 | 8.18 | 6.15 | 3.25 | 3.14E-06 | 1.69 | 4.35E-03 | 1363 |
| Cluster-40555.201884 | 0.34 | 0.12 | 2.37 | 1.62 | 0.67 | 0.96 | 4.02 | 3.3 | 3.25 | 2.82E-04 | 2.23 | 1.32E-04 | 2329 |
| Cluster-40555.190829 | 4.87 | 3.63 | 42.03 | 30.7 | 22.28 | 20.07 | 54.94 | 52.46 | 3.25 | 2.03E-14 | 1.41 | 3.14E-04 | 840 |
| Cluster-40555.185429 | 5.48 | 2.35 | 31.7 | 35.12 | 0.26 | 1.79 | 24.06 | 17.46 | 3.26 | 6.70E-21 | 4.37 | 1.22E-18 | 1177 |
| Cluster-40555.191689 | 10.5 | 6.31 | 78.94 | 65.71 | 51.83 | 44.38 | 83.03 | 78.41 | 3.26 | 1.16E-34 | 0.82 | 4.59E-02 | 1728 |
| Cluster-40555.189865 | 8.09 | 10.35 | 87.16 | 74.58 | 19.57 | 19.88 | 83.66 | 74.86 | 3.26 | 7.34E-32 | 2.07 | 9.66E-11 | 1292 |
| Cluster-40555.173160 | 0.74 | 1.43 | 9.43 | 9.63 | 1.1 | 0.66 | 3.42 | 5.56 | 3.27 | 3.52E-10 | 2.43 | 1.74E-02 | 1443 |
| Cluster-40555.216454 | 0.45 | 0.5 | 4.04 | 4.05 | 1.75 | 2.32 | 6.5 | 5.48 | 3.27 | 2.72E-04 | 1.60 | 2.22E-02 | 1252 |
| Cluster-40555.203990 | 0.27 | 0.25 | 2.25 | 2.35 | 0.11 | 0.28 | 1.26 | 1.65 | 3.27 | 2.34E-04 | 2.97 | 5.74E-03 | 2018 |
| Cluster-40555.219514 | 0.11 | 0.34 | 2.27 | 1.62 | 0.47 | 0.35 | 3.85 | 2.42 | 3.28 | 7.09E-04 | 2.96 | 3.10E-05 | 2187 |
| Cluster-40555.218548 | 1.23 | 0.92 | 10.05 | 9.03 | 0.32 | 0.14 | 2.65 | 2.03 | 3.28 | 2.93E-07 | 3.53 | 1.26E-02 | 1036 |
| Cluster-40555.151985 | 0.33 | 0.4 | 4.27 | 2.24 | 0.18 | 0.1 | 1.68 | 1.73 | 3.28 | 4.59E-03 | 3.76 | 4.39E-04 | 1861 |
| Cluster-40555.185898 | 0.96 | 0.38 | 5.72 | 5.86 | 0.56 | 0.32 | 9.1 | 8.84 | 3.28 | 2.57E-09 | 4.43 | 6.23E-19 | 1933 |
| Cluster-40555.151350 | 0.21 | 2.61 | 12.52 | 13.31 | 1.87 | 0.88 | 6.76 | 8.02 | 3.29 | 1.04E-10 | 2.51 | 5.46E-05 | 1199 |
| Cluster-40555.204037 | 0.17 | 0.82 | 4.32 | 4.47 | 0 | 0.09 | 2.29 | 1.88 | 3.29 | 3.35E-04 | 5.28 | 3.95E-04 | 1162 |
| Cluster-40555.192218 | 9.46 | 14.06 | 103.01 | 108.15 | 46.13 | 45.27 | 109.67 | 112.14 | 3.30 | 7.82E-30 | 1.34 | 1.10E-04 | 868 |

| | | | | | | | | | | | | | |
|---|---|---|---|---|---|---|---|---|---|---|---|---|---|
| Cluster-40555.172256 | 4.82 | 1.43 | 26.61 | 27.81 | 16 | 10.6 | 28.99 | 22.46 | 3.30 | 1.35E-20 | 1.02 | 2.46E-02 | 1325 |
| Cluster-40555.186791 | 10.43 | 7.47 | 65.77 | 92.21 | 64.84 | 62.93 | 119.55 | 112.4 | 3.30 | 5.47E-08 | 0.93 | 1.30E-02 | 1394 |
| Cluster-40555.166886 | 1.08 | 0.9 | 8.62 | 8.97 | 0.53 | 0.19 | 10.09 | 6.1 | 3.30 | 4.76E-05 | 4.58 | 1.35E-06 | 797 |
| Cluster-40555.215724 | 2.24 | 1 | 16.27 | 12.23 | 1.16 | 1.4 | 7.24 | 5.45 | 3.30 | 9.62E-06 | 2.32 | 4.60E-02 | 654 |
| Cluster-40555.178275 | 1.27 | 1.48 | 12.93 | 12.21 | 0.23 | 0.26 | 20.82 | 16.03 | 3.30 | 4.78E-04 | 6.23 | 4.41E-12 | 557 |
| Cluster-40555.144710 | 0.1 | 0.59 | 3.12 | 3.22 | 0.27 | 0.19 | 2.76 | 3.17 | 3.31 | 5.78E-06 | 3.75 | 2.10E-07 | 2109 |
| Cluster-40555.204370 | 0.25 | 0.43 | 2.98 | 3.27 | 0.83 | 0.16 | 3.07 | 3.28 | 3.31 | 1.35E-04 | 2.76 | 5.74E-04 | 1621 |
| Cluster-40555.170693 | 1.47 | 1.31 | 11.14 | 13.75 | 0.82 | 0.36 | 7.14 | 4.35 | 3.31 | 1.77E-16 | 3.35 | 3.27E-05 | 1894 |
| Cluster-40555.261382 | 0.45 | 1.11 | 7.11 | 7.18 | 0 | 0 | 2.99 | 2.92 | 3.31 | 1.14E-02 | Inf | 1.45E-02 | 585 |
| Cluster-40555.210797 | 0.48 | 0.45 | 4.23 | 3.79 | 0.64 | 1.21 | 6.56 | 4.7 | 3.31 | 6.35E-03 | 2.60 | 2.80E-03 | 899 |
| Cluster-40555.176267 | 0.44 | 0.31 | 4.06 | 3.03 | 0.38 | 0.08 | 3.71 | 4.15 | 3.31 | 1.60E-02 | 4.19 | 4.55E-04 | 878 |
| Cluster-40555.181429 | 0 | 0.23 | 1.27 | 0.88 | 0.1 | 0.12 | 2.05 | 2.37 | 3.31 | 3.95E-02 | 4.38 | 1.42E-06 | 1991 |
| Cluster-40555.185810 | 3.98 | 1.64 | 23.69 | 25.93 | 5.78 | 2.32 | 23.27 | 22.14 | 3.31 | 3.48E-14 | 2.57 | 2.04E-08 | 920 |
| Cluster-40555.210326 | 0.62 | 0.74 | 7.39 | 5.1 | 1 | 1.27 | 4.46 | 4.13 | 3.32 | 8.36E-09 | 1.98 | 1.80E-03 | 1785 |
| Cluster-40555.251433 | 0.25 | 0.1 | 1.94 | 1.2 | 0.16 | 0.27 | 1.55 | 1.83 | 3.32 | 4.78E-05 | 3.03 | 1.60E-05 | 3377 |
| Cluster-40555.155596 | 1.07 | 0.76 | 9 | 7.77 | 0.58 | 1.18 | 5.27 | 2.9 | 3.32 | 1.81E-09 | 2.24 | 3.10E-02 | 1457 |
| Cluster-40555.205435 | 3.5 | 4.07 | 34.17 | 34.55 | 7.21 | 5.02 | 29.68 | 25.35 | 3.32 | 1.76E-26 | 2.24 | 3.13E-10 | 1646 |
| Cluster-40555.189734 | 0.78 | 0.43 | 5.54 | 5.28 | 1.46 | 1.15 | 3.46 | 3.91 | 3.32 | 4.06E-16 | 1.58 | 1.16E-03 | 3960 |
| Cluster-40555.168040 | 0.48 | 0.34 | 4.1 | 3.18 | 1.68 | 1.82 | 5.75 | 7.19 | 3.32 | 2.53E-06 | 1.94 | 1.16E-04 | 1973 |
| Cluster-40555.171248 | 2.14 | 2.31 | 19.77 | 20.69 | 1.18 | 1.43 | 13.03 | 7.97 | 3.32 | 1.41E-08 | 3.07 | 1.18E-04 | 692 |
| Cluster-40555.201361 | 2.19 | 1.94 | 17.76 | 19.42 | 0.87 | 0 | 10.66 | 6.35 | 3.33 | 1.31E-04 | 4.40 | 2.58E-03 | 486 |
| Cluster-40555.187960 | 72.45 | 84.53 | 767.16 | 668.9 | 81.99 | 77.83 | 268.6 | 252.41 | 3.33 | 9.68E-48 | 1.77 | 3.90E-09 | 1475 |
| Cluster-40555.165673 | 0.73 | 0.43 | 4.67 | 5.83 | 4.06 | 5.72 | 11.33 | 12.96 | 3.33 | 1.40E-07 | 1.37 | 2.68E-03 | 1646 |
| Cluster-40555.171849 | 3.23 | 2.68 | 28.05 | 25.93 | 1.9 | 1.16 | 33.43 | 30.36 | 3.33 | 8.91E-19 | 4.46 | 1.59E-27 | 1202 |
| Cluster-40555.184444 | 3.18 | 2.73 | 27.83 | 25.85 | 4.41 | 12.51 | 25.15 | 22.46 | 3.33 | 1.93E-06 | 1.52 | 4.05E-02 | 493 |
| Cluster-40555.187225 | 2.23 | 4.12 | 27.08 | 31.61 | 4.2 | 5.71 | 34.54 | 27.06 | 3.33 | 1.10E-15 | 2.69 | 6.28E-11 | 912 |
| Cluster-40555.197612 | 1 | 0.56 | 6.76 | 7.24 | 3.09 | 3.27 | 7.11 | 7.12 | 3.34 | 1.09E-10 | 1.22 | 2.93E-02 | 1861 |
| Cluster-40555.202674 | 1.56 | 1.36 | 14.94 | 12.05 | 2.08 | 1.74 | 16.32 | 15.67 | 3.34 | 1.89E-11 | 3.14 | 7.93E-12 | 1206 |
| Cluster-40555.146559 | 0.68 | 0.28 | 4.96 | 3.85 | 0.04 | 0.08 | 3.66 | 2.21 | 3.34 | 7.48E-06 | 5.72 | 5.38E-08 | 1530 |
| Cluster-40555.208842 | 0.06 | 0.17 | 1.22 | 1.06 | 0.9 | 1.31 | 6.15 | 4.63 | 3.35 | 1.28E-04 | 2.34 | 6.78E-09 | 4073 |
| Cluster-40555.204768 | 1.71 | 1.1 | 12.3 | 13.36 | 0.18 | 0.15 | 9.41 | 8.24 | 3.35 | 1.10E-14 | 5.83 | 1.59E-21 | 1584 |
| Cluster-40555.193130 | 1.5 | 1.31 | 12.72 | 13.06 | 1.82 | 2.2 | 7.42 | 7.23 | 3.35 | 1.05E-08 | 1.94 | 9.38E-03 | 939 |

| | | | | | | | | | | | | | |
|---|---|---|---|---|---|---|---|---|---|---|---|---|---|
| Cluster-40555.149725 | 0.69 | 0.86 | 8.39 | 6.12 | 0.51 | 0.27 | 5.21 | 5 | 3.35 | 2.47E-04 | 3.80 | 3.10E-04 | 819 |
| Cluster-40555.185448 | 1.26 | 3.19 | 19.1 | 22.88 | 3.13 | 3.78 | 14.81 | 16.17 | 3.35 | 4.38E-10 | 2.22 | 1.41E-04 | 761 |
| Cluster-40555.189636 | 0.82 | 0.47 | 6.97 | 5.23 | 2.12 | 1.7 | 7.3 | 8.33 | 3.35 | 4.29E-07 | 2.11 | 1.29E-04 | 1413 |
| Cluster-40555.223188 | 0.35 | 0.22 | 2.84 | 2.47 | 1.1 | 0.93 | 3.53 | 3.16 | 3.35 | 1.06E-07 | 1.80 | 8.92E-04 | 3138 |
| Cluster-40555.217218 | 3.19 | 2.66 | 25.02 | 29.15 | 7.33 | 4.63 | 31.61 | 27.4 | 3.36 | 3.09E-15 | 2.38 | 1.33E-08 | 917 |
| Cluster-40555.178030 | 0.82 | 2.49 | 15.47 | 16.17 | 1.82 | 2.43 | 19.34 | 12.76 | 3.36 | 6.65E-13 | 2.96 | 8.13E-07 | 1189 |
| Cluster-40555.147483 | 0.51 | 0.08 | 2.52 | 2.69 | 0.05 | 0.05 | 1.01 | 1.5 | 3.36 | 9.54E-04 | 5.08 | 3.06E-03 | 1485 |
| Cluster-40555.216767 | 1.45 | 1.65 | 14.57 | 14.58 | 3.88 | 1.6 | 6.51 | 8.29 | 3.36 | 1.12E-14 | 1.52 | 1.91E-02 | 1446 |
| Cluster-40555.151630 | 0.66 | 0.63 | 5.91 | 6.19 | 1.15 | 1.64 | 9.12 | 7.45 | 3.36 | 1.00E-05 | 2.61 | 1.18E-05 | 1138 |
| Cluster-40555.141158 | 0.2 | 0.16 | 1.85 | 1.57 | 0.32 | 0.3 | 2.51 | 3.08 | 3.37 | 1.06E-02 | 3.28 | 1.36E-04 | 1606 |
| Cluster-40555.164465 | 0.58 | 0.83 | 7.01 | 6.42 | 1.53 | 2.7 | 9.96 | 10.95 | 3.37 | 3.87E-09 | 2.36 | 1.45E-07 | 1660 |
| Cluster-40555.186961 | 9.62 | 8.69 | 86.83 | 84.24 | 9.13 | 11.47 | 36.99 | 29.75 | 3.37 | 8.28E-18 | 1.75 | 2.03E-03 | 526 |
| Cluster-40555.194615 | 1.81 | 1.19 | 13.9 | 14.1 | 9.3 | 7.19 | 23 | 19.53 | 3.37 | 6.25E-24 | 1.44 | 4.46E-05 | 2864 |
| Cluster-40555.165052 | 1.86 | 2.58 | 19.22 | 22.56 | 5.61 | 2.24 | 22.31 | 13.53 | 3.37 | 1.35E-18 | 2.27 | 5.32E-03 | 1402 |
| Cluster-40555.182932 | 0.98 | 2.99 | 19.32 | 19 | 1.07 | 2.03 | 7.91 | 10.55 | 3.37 | 6.66E-13 | 2.65 | 1.21E-05 | 1026 |
| Cluster-40555.250663 | 0.11 | 0 | 0.65 | 0.42 | 0 | 0.03 | 0.4 | 0.7 | 3.38 | 7.83E-03 | 5.09 | 6.29E-04 | 4763 |
| Cluster-40555.183984 | 0.33 | 0.53 | 3.66 | 3.92 | 0.14 | 0 | 3.92 | 2.49 | 3.38 | 6.83E-03 | 5.57 | 1.42E-04 | 906 |
| Cluster-40555.213280 | 2.91 | 2.81 | 26.99 | 26.86 | 3.17 | 1.71 | 14.71 | 17.41 | 3.38 | 9.73E-16 | 2.80 | 3.62E-08 | 959 |
| Cluster-40555.235688 | 0.25 | 0.36 | 3.24 | 2.73 | 0.54 | 0.42 | 2.95 | 2.03 | 3.38 | 2.27E-06 | 2.46 | 5.51E-04 | 2323 |
| Cluster-40555.179062 | 0.29 | 0.38 | 2.79 | 3.58 | 0 | 0 | 2.08 | 1.8 | 3.38 | 1.97E-04 | Inf | 1.15E-06 | 1476 |
| Cluster-40555.189152 | 11.08 | 11.89 | 107.39 | 109.78 | 11.54 | 9.6 | 19.55 | 23.43 | 3.38 | 5.53E-34 | 1.09 | 3.72E-02 | 1006 |
| Cluster-40555.179825 | 0.79 | 0.44 | 5.76 | 5.89 | 1.03 | 0.97 | 3.85 | 3.07 | 3.39 | 5.50E-07 | 1.86 | 3.45E-02 | 1390 |
| Cluster-40555.180265 | 3.06 | 6.91 | 40.6 | 55.35 | 19.15 | 13.71 | 37.47 | 48.05 | 3.39 | 1.17E-08 | 1.45 | 1.11E-02 | 502 |
| Cluster-40555.151099 | 0.84 | 0.93 | 10.05 | 7.04 | 0.19 | 0.08 | 2.68 | 4.46 | 3.39 | 2.89E-10 | 4.89 | 4.08E-05 | 1556 |
| Cluster-40555.133899 | 0.12 | 0.16 | 1.24 | 1.44 | 0 | 0.03 | 0.72 | 0.64 | 3.39 | 1.55E-02 | 5.43 | 2.31E-02 | 1804 |
| Cluster-40555.187382 | 30.35 | 43.67 | 361.57 | 348.81 | 199.93 | 191.25 | 391.48 | 443.67 | 3.39 | 1.14E-45 | 1.16 | 3.97E-04 | 1144 |
| Cluster-40555.219692 | 0.6 | 0.71 | 6.43 | 6.1 | 1.87 | 1.09 | 7.8 | 8.97 | 3.39 | 5.17E-05 | 2.58 | 9.08E-05 | 990 |
| Cluster-40555.204699 | 0.28 | 0.21 | 2.14 | 2.31 | 0.18 | 0.07 | 1.91 | 2.06 | 3.39 | 4.07E-02 | 4.09 | 1.36E-02 | 1022 |
| Cluster-40555.180193 | 0.19 | 0.21 | 2.05 | 1.84 | 0.88 | 0.53 | 2.44 | 2.78 | 3.39 | 1.43E-06 | 1.97 | 5.76E-04 | 3488 |
| Cluster-40555.153355 | 1.24 | 0 | 6.4 | 5.02 | 0 | 0 | 3.3 | 2.36 | 3.39 | 1.09E-07 | Inf | 2.85E-10 | 1542 |
| Cluster-40555.116415 | 0.58 | 0.54 | 5.55 | 5.13 | 0 | 0.08 | 1.56 | 1.61 | 3.40 | 1.30E-03 | 5.25 | 4.86E-02 | 850 |
| Cluster-40555.197865 | 0.36 | 0.21 | 3.13 | 2.28 | 0.53 | 0.37 | 3.21 | 2.58 | 3.40 | 4.82E-04 | 2.77 | 9.34E-04 | 1603 |

| | | | | | | | | | | | | | |
|---|---|---|---|---|---|---|---|---|---|---|---|---|---|
| Cluster-40555.214089 | 0.28 | 0.18 | 2.17 | 2.24 | 0.57 | 0.55 | 3.31 | 2.42 | 3.40 | 5.11E-07 | 2.43 | 1.16E-05 | 3235 |
| Cluster-40555.189266 | 3.53 | 2.6 | 29.53 | 29 | 5.8 | 4.17 | 17.59 | 22.84 | 3.40 | 2.10E-27 | 2.09 | 9.59E-06 | 1856 |
| Cluster-40555.186162 | 4.73 | 4.63 | 43.61 | 46.48 | 0.53 | 0.94 | 25.57 | 15.45 | 3.40 | 4.64E-23 | 4.87 | 3.65E-08 | 1003 |
| Cluster-40555.154735 | 0.8 | 0.22 | 5.44 | 4.18 | 0.13 | 0 | 3.1 | 3.41 | 3.40 | 3.68E-02 | 5.74 | 1.25E-02 | 627 |
| Cluster-40555.162743 | 0.51 | 0.18 | 3.79 | 2.71 | 0 | 0.8 | 2.5 | 3.01 | 3.40 | 3.56E-05 | 2.80 | 3.96E-04 | 1725 |
| Cluster-40555.193485 | 0.36 | 0.38 | 3.51 | 3.71 | 0.43 | 0.35 | 2.48 | 2.83 | 3.41 | 6.89E-04 | 2.86 | 1.04E-02 | 1196 |
| Cluster-40555.185487 | 0.85 | 1.25 | 8.22 | 11.67 | 4.41 | 3.52 | 10.96 | 11.09 | 3.41 | 1.70E-07 | 1.54 | 8.17E-03 | 1075 |
| Cluster-40555.172688 | 0.41 | 0 | 1.67 | 2.03 | 0.13 | 0.11 | 1.13 | 1.27 | 3.41 | 2.67E-04 | 3.52 | 2.46E-03 | 2195 |
| Cluster-40555.179276 | 0.8 | 0.99 | 8.41 | 8.8 | 0.41 | 0.09 | 2.69 | 2.54 | 3.41 | 3.72E-06 | 3.48 | 1.97E-02 | 920 |
| Cluster-40555.189208 | 0.61 | 1.03 | 8.54 | 7.65 | 0.06 | 0.06 | 2.17 | 1.96 | 3.41 | 2.81E-14 | 5.53 | 4.73E-09 | 2301 |
| Cluster-40555.183138 | 3.53 | 5.23 | 40.86 | 44.25 | 9.78 | 8.85 | 32.52 | 37.98 | 3.41 | 1.72E-28 | 1.99 | 1.56E-08 | 1450 |
| Cluster-40555.177275 | 5.43 | 4.27 | 47.77 | 45.22 | 38.44 | 27.79 | 95.95 | 72.24 | 3.41 | 1.76E-20 | 1.41 | 8.43E-05 | 852 |
| Cluster-40555.192552 | 5.99 | 10.78 | 85.79 | 78.92 | 1.95 | 0 | 10.22 | 16.25 | 3.41 | 3.32E-27 | 3.88 | 2.15E-04 | 839 |
| Cluster-40555.197338 | 1.36 | 0.76 | 11.12 | 9.13 | 2.32 | 1.62 | 9.21 | 6.63 | 3.42 | 2.64E-11 | 2.07 | 1.27E-04 | 1436 |
| Cluster-40555.162231 | 0.49 | 0.2 | 2.84 | 3.56 | 0.28 | 0.24 | 2.37 | 2.57 | 3.42 | 1.64E-05 | 3.34 | 1.31E-04 | 1753 |
| Cluster-40555.174375 | 8.95 | 7.14 | 73.62 | 81.53 | 25.79 | 21.38 | 64.02 | 48.35 | 3.42 | 1.47E-18 | 1.31 | 5.81E-03 | 556 |
| Cluster-40555.275490 | 0.37 | 0 | 1.41 | 2.02 | 0 | 0 | 1.83 | 1.73 | 3.42 | 1.21E-02 | Inf | 6.27E-06 | 1439 |
| Cluster-40555.192208 | 0.86 | 1.14 | 9.39 | 10.19 | 0.78 | 0.54 | 3.84 | 4.95 | 3.42 | 2.38E-19 | 2.80 | 3.14E-08 | 2791 |
| Cluster-40555.121652 | 0.36 | 0.88 | 6.38 | 6.39 | 1.19 | 0.96 | 7.4 | 7.52 | 3.43 | 2.77E-05 | 2.85 | 3.33E-05 | 1015 |
| Cluster-40555.220087 | 0.93 | 0.61 | 6.99 | 8.15 | 0.21 | 0.13 | 4.93 | 5.39 | 3.43 | 1.97E-13 | 4.95 | 4.50E-16 | 2144 |
| Cluster-40555.182947 | 0.85 | 1.3 | 10.14 | 10.89 | 1.38 | 1.09 | 20.86 | 25.7 | 3.43 | 1.42E-15 | 4.32 | 1.87E-21 | 1970 |
| Cluster-40555.190893 | 0.05 | 0.47 | 2.41 | 2.82 | 0.56 | 0.29 | 2.27 | 1.9 | 3.43 | 1.28E-05 | 2.33 | 4.67E-03 | 2221 |
| Cluster-40555.224378 | 0.19 | 0.06 | 1.41 | 1.01 | 0.73 | 0.65 | 2.12 | 1.86 | 3.43 | 3.38E-06 | 1.60 | 3.90E-03 | 5034 |
| Cluster-40555.190048 | 11.77 | 5.44 | 86.66 | 79.36 | 37.15 | 30.54 | 104.11 | 87.97 | 3.43 | 2.05E-25 | 1.57 | 1.10E-05 | 731 |
| Cluster-40555.144029 | 1.3 | 1.02 | 11.61 | 11.14 | 0.75 | 0.89 | 5.3 | 5.17 | 3.43 | 2.77E-12 | 2.74 | 3.12E-05 | 1403 |
| Cluster-40555.199773 | 5.78 | 5.35 | 57 | 52.36 | 5.91 | 4.3 | 50.11 | 42.92 | 3.44 | 9.77E-38 | 3.26 | 6.04E-24 | 2185 |
| Cluster-40555.135149 | 0.19 | 0.45 | 2.99 | 3.23 | 0.23 | 0.09 | 1.71 | 2.52 | 3.44 | 9.11E-05 | 3.69 | 3.75E-04 | 1616 |
| Cluster-40555.145862 | 0.26 | 0.3 | 1.77 | 3.7 | 0.35 | 0.26 | 2.12 | 2.16 | 3.44 | 2.60E-02 | 2.87 | 2.92E-02 | 1200 |
| Cluster-40555.181297 | 0.82 | 0.61 | 7.4 | 6.56 | 0.22 | 0.09 | 4.72 | 3.22 | 3.44 | 4.14E-15 | 4.78 | 7.65E-14 | 2731 |
| Cluster-40555.189483 | 14.22 | 22.55 | 198.73 | 167.88 | 30.43 | 40.17 | 90.45 | 135.48 | 3.44 | 3.48E-33 | 1.74 | 1.97E-02 | 668 |
| Cluster-40555.159538 | 2.37 | 5.25 | 36.01 | 39.85 | 14.71 | 17.45 | 57.29 | 51.75 | 3.44 | 8.50E-05 | 1.79 | 1.14E-02 | 371 |
| Cluster-40555.191312 | 1.33 | 2.56 | 20.5 | 18.38 | 3.58 | 2.23 | 8.53 | 12.2 | 3.44 | 8.19E-17 | 1.92 | 5.34E-03 | 1314 |

| | | | | | | | | | | | | | |
|---|---|---|---|---|---|---|---|---|---|---|---|---|---|
| Cluster-40555.180765 | 2.16 | 3.24 | 25.33 | 28.59 | 17.83 | 16.54 | 32.15 | 34.63 | 3.45 | 1.51E-19 | 1.03 | 1.92E-02 | 1177 |
| Cluster-40555.205126 | 0.38 | 0.36 | 4.16 | 3.19 | 0.42 | 0.33 | 4.44 | 4.68 | 3.45 | 9.12E-04 | 3.67 | 1.32E-05 | 1151 |
| Cluster-40555.188392 | 0 | 1.08 | 4.1 | 7.02 | 0 | 0 | 4.91 | 4.68 | 3.46 | 2.43E-03 | Inf | 4.84E-20 | 1819 |
| Cluster-40555.198871 | 0.57 | 0.42 | 4.11 | 5.68 | 0.6 | 0.54 | 4.54 | 3.95 | 3.46 | 8.33E-09 | 2.96 | 2.69E-08 | 2557 |
| Cluster-40555.161018 | 0.58 | 0.25 | 3.72 | 4.27 | 0.36 | 0.43 | 2.64 | 3.32 | 3.46 | 2.63E-04 | 2.95 | 5.22E-03 | 1158 |
| Cluster-40555.191000 | 22.4 | 26.44 | 222.32 | 269.19 | 169.94 | 130.13 | 350.76 | 456.5 | 3.47 | 2.42E-21 | 1.50 | 4.73E-04 | 597 |
| Cluster-40555.194149 | 0.71 | 0.49 | 5.78 | 6.85 | 0.22 | 0 | 4.66 | 5.37 | 3.48 | 1.52E-03 | 5.73 | 3.91E-05 | 722 |
| Cluster-40555.148828 | 1.09 | 1.26 | 12.25 | 10.75 | 1.79 | 3.3 | 10.71 | 12.64 | 3.48 | 3.61E-03 | 2.26 | 4.30E-02 | 490 |
| Cluster-40555.172542 | 1.65 | 0.79 | 14.04 | 9.98 | 1.57 | 3.06 | 17.41 | 12.75 | 3.48 | 4.36E-05 | 2.76 | 4.31E-05 | 638 |
| Cluster-40555.202482 | 2.14 | 1.32 | 16.08 | 18.51 | 7.3 | 7.68 | 28.22 | 26.39 | 3.48 | 4.40E-27 | 1.93 | 4.31E-09 | 2646 |
| Cluster-40555.200114 | 1.1 | 2.71 | 21.43 | 18.06 | 10.05 | 5.94 | 29.23 | 27.2 | 3.49 | 1.40E-09 | 1.89 | 6.64E-05 | 747 |
| Cluster-40555.188218 | 177.53 | 168.94 | 1624.14 | 1893.04 | 3.15 | 3.68 | 554.28 | 800.73 | 3.49 | 6.19E-31 | 7.69 | 4.00E-19 | 876 |
| Cluster-40555.193301 | 8 | 11.09 | 97.2 | 98.56 | 12.36 | 15.91 | 39.71 | 36.04 | 3.49 | 1.18E-23 | 1.47 | 3.04E-03 | 602 |
| Cluster-40555.223410 | 0.44 | 0 | 2.91 | 1.51 | 0.58 | 0.54 | 1.69 | 2.23 | 3.49 | 4.32E-03 | 1.88 | 1.40E-02 | 2740 |
| Cluster-40555.214132 | 1.65 | 3.28 | 26.25 | 24.58 | 5.37 | 11.31 | 18.02 | 20.62 | 3.49 | 3.42E-22 | 1.26 | 2.17E-02 | 1463 |
| Cluster-40555.188190 | 188.72 | 373.94 | 2861.06 | 2961.18 | 920.01 | 901.82 | 2373.57 | 2212.44 | 3.49 | 1.00E-53 | 1.40 | 4.69E-06 | 1052 |
| Cluster-40555.220069 | 0.48 | 0.19 | 3.5 | 3.19 | 0.19 | 1.28 | 6.68 | 7.35 | 3.49 | 8.22E-05 | 3.29 | 3.95E-09 | 1497 |
| Cluster-40555.164967 | 0.3 | 0.49 | 3.88 | 4.22 | 0.06 | 0.17 | 3.02 | 1.58 | 3.50 | 2.51E-05 | 4.45 | 4.85E-04 | 1408 |
| Cluster-40555.189611 | 6.16 | 10.54 | 92.01 | 81.24 | 5.2 | 4.39 | 33.79 | 32.53 | 3.50 | 7.97E-35 | 2.86 | 2.68E-14 | 1174 |
| Cluster-40555.190632 | 4.47 | 5.16 | 48.48 | 50.34 | 11.33 | 11.73 | 47.75 | 39.39 | 3.50 | 1.76E-31 | 1.98 | 4.92E-09 | 1437 |
| Cluster-40555.205381 | 0 | 0.46 | 2.43 | 2.47 | 0.15 | 0.92 | 3.7 | 3.27 | 3.50 | 4.96E-05 | 2.74 | 1.12E-05 | 2089 |
| Cluster-40555.186113 | 12.22 | 11.64 | 114.06 | 129.98 | 85.16 | 88.41 | 139.16 | 151.94 | 3.50 | 1.95E-38 | 0.81 | 4.35E-02 | 1190 |
| Cluster-40555.201470 | 15.56 | 9.35 | 126.22 | 126.83 | 75.37 | 61.1 | 156.49 | 132.39 | 3.50 | 9.52E-29 | 1.15 | 2.36E-03 | 630 |
| Cluster-40555.217681 | 1.82 | 0.41 | 9.15 | 12.91 | 1.98 | 1.02 | 20.91 | 20.39 | 3.50 | 2.90E-07 | 3.84 | 2.76E-13 | 829 |
| Cluster-40555.247597 | 0 | 0.46 | 2.3 | 2.47 | 0 | 0 | 0.97 | 0.86 | 3.50 | 1.53E-03 | Inf | 3.71E-03 | 1510 |
| Cluster-40555.201863 | 0.5 | 0.21 | 4.36 | 2.95 | 0.19 | 0.24 | 2.98 | 3.39 | 3.50 | 4.95E-08 | 3.93 | 2.94E-09 | 2348 |
| Cluster-40555.232727 | 0.1 | 0.54 | 3.55 | 3.26 | 1.69 | 2.39 | 5.18 | 4.91 | 3.50 | 3.61E-10 | 1.37 | 3.63E-03 | 3274 |
| Cluster-40555.186910 | 5.14 | 4.16 | 49.62 | 46.26 | 11.22 | 11.52 | 74.81 | 64.36 | 3.51 | 8.27E-35 | 2.68 | 8.46E-18 | 1812 |
| Cluster-40555.204610 | 0.49 | 0.35 | 5.08 | 3.42 | 0.81 | 0.69 | 5.73 | 4.67 | 3.51 | 3.87E-05 | 2.85 | 3.14E-05 | 1342 |
| Cluster-40555.161145 | 5.11 | 7.65 | 67.08 | 65.81 | 17.11 | 14.23 | 50.74 | 54.61 | 3.51 | 1.28E-21 | 1.82 | 8.01E-06 | 689 |
| Cluster-40555.155809 | 2.46 | 1.13 | 19.44 | 16.09 | 0 | 0 | 21.54 | 25.8 | 3.51 | 2.36E-02 | Inf | 3.05E-06 | 363 |
| Cluster-40555.186010 | 16.99 | 14.93 | 179.59 | 150.62 | 62.61 | 53.7 | 213.37 | 146.48 | 3.52 | 1.10E-30 | 1.69 | 5.17E-04 | 597 |

| | | | | | | | | | | | | | |
|---|---|---|---|---|---|---|---|---|---|---|---|---|---|
| Cluster-40555.195942 | 2.21 | 0.62 | 17.76 | 11.33 | 13.76 | 17.7 | 31.94 | 26.53 | 3.52 | 5.03E-07 | 0.95 | 2.10E-02 | 1689 |
| Cluster-40555.188349 | 12.94 | 12.08 | 122.73 | 136.89 | 38.46 | 37.05 | 107.52 | 104.75 | 3.52 | 1.76E-43 | 1.56 | 1.13E-06 | 1403 |
| Cluster-40555.267818 | 0.22 | 0.37 | 3.19 | 3 | 0.09 | 0.32 | 1.47 | 1.36 | 3.52 | 2.93E-06 | 2.86 | 7.05E-03 | 2073 |
| Cluster-40555.194528 | 3.81 | 2.98 | 36.62 | 33.62 | 22.34 | 23.44 | 55.35 | 48.51 | 3.52 | 7.47E-24 | 1.24 | 7.20E-04 | 1203 |
| Cluster-40555.149460 | 0 | 0.47 | 2.13 | 2.99 | 0.31 | 0 | 2.07 | 4.79 | 3.52 | 6.57E-04 | 4.67 | 3.71E-02 | 1525 |
| Cluster-40555.182668 | 2.01 | 2.25 | 21.3 | 23.08 | 0.17 | 0.39 | 9.52 | 4.83 | 3.53 | 8.56E-08 | 4.99 | 7.77E-04 | 579 |
| Cluster-40555.164482 | 0.37 | 0.35 | 3.96 | 3.69 | 2.59 | 2.32 | 8.21 | 7.84 | 3.53 | 2.09E-08 | 1.77 | 6.57E-05 | 2301 |
| Cluster-40555.134332 | 0.19 | 0 | 0.79 | 1.18 | 0 | 0 | 0.63 | 1.02 | 3.53 | 6.40E-03 | Inf | 3.27E-05 | 2488 |
| Cluster-40555.204380 | 0.38 | 0.15 | 2.13 | 3.24 | 3.19 | 0.75 | 6.23 | 8.43 | 3.53 | 1.34E-05 | 2.00 | 1.42E-02 | 2088 |
| Cluster-40555.189706 | 122.63 | 155.03 | 1451.6 | 1471.17 | 746.29 | 699.35 | 1374.12 | 1396.58 | 3.53 | 1.01E-54 | 1.01 | 2.91E-03 | 1510 |
| Cluster-40555.225026 | 0.38 | 0.55 | 5.09 | 4.76 | 0.19 | 0 | 3.79 | 2.42 | 3.53 | 4.40E-03 | 5.16 | 2.85E-03 | 768 |
| Cluster-40555.196695 | 0.73 | 1.09 | 9.18 | 10.12 | 1.02 | 1.59 | 7.64 | 5.29 | 3.53 | 2.63E-17 | 2.35 | 1.54E-06 | 2290 |
| Cluster-40555.173634 | 2.04 | 2.8 | 22.3 | 28.72 | 4.16 | 5.97 | 31.92 | 24.17 | 3.54 | 9.00E-09 | 2.52 | 5.32E-06 | 573 |
| Cluster-40555.157232 | 0.47 | 0.63 | 6.09 | 5.49 | 0.43 | 0.59 | 3.49 | 2.95 | 3.54 | 9.12E-09 | 2.72 | 3.12E-04 | 1684 |
| Cluster-40555.218805 | 1.07 | 2.08 | 14.61 | 19.01 | 4.95 | 2.45 | 10.81 | 10.21 | 3.54 | 2.57E-09 | 1.60 | 4.13E-02 | 772 |
| Cluster-40555.232122 | 0.11 | 0.15 | 1.29 | 1.53 | 0.57 | 0.39 | 2.57 | 2.05 | 3.54 | 3.67E-03 | 2.34 | 4.03E-03 | 2049 |
| Cluster-40555.189770 | 49.23 | 53.34 | 513.07 | 569.41 | 301.91 | 282.28 | 567.08 | 602.65 | 3.54 | 3.70E-51 | 1.07 | 1.48E-03 | 995 |
| Cluster-40555.229601 | 0.26 | 0.08 | 2.04 | 1.77 | 0.28 | 0.62 | 2.04 | 3.5 | 3.54 | 5.48E-03 | 2.70 | 2.21E-02 | 1515 |
| Cluster-40555.152717 | 1.77 | 2.8 | 23.4 | 25.19 | 1.15 | 0.48 | 14.1 | 13.39 | 3.54 | 1.90E-10 | 4.15 | 1.79E-08 | 674 |
| Cluster-40555.213847 | 0.27 | 0.14 | 1.9 | 2.36 | 0 | 0.11 | 1.67 | 1.13 | 3.54 | 1.49E-03 | 4.67 | 1.10E-03 | 1580 |
| Cluster-40555.143194 | 0.1 | 0.21 | 1.57 | 1.82 | 0.1 | 0.1 | 1.94 | 1.44 | 3.55 | 4.11E-02 | 4.10 | 9.37E-03 | 1202 |
| Cluster-40555.158102 | 0.77 | 0.75 | 7.93 | 7.72 | 0.5 | 0.82 | 5.15 | 4.72 | 3.55 | 6.89E-06 | 2.93 | 1.66E-03 | 915 |
| Cluster-40555.190948 | 31.63 | 31.07 | 319.52 | 346.11 | 189.93 | 166.03 | 362.56 | 371.35 | 3.55 | 2.51E-51 | 1.11 | 8.00E-04 | 1442 |
| Cluster-40555.213174 | 0.49 | 0 | 2.42 | 2.29 | 0 | 0 | 0.82 | 2.27 | 3.55 | 2.61E-02 | Inf | 4.89E-02 | 991 |
| Cluster-40555.222387 | 0.79 | 0.52 | 7.06 | 6.79 | 1 | 1.2 | 6.14 | 7.07 | 3.55 | 3.17E-10 | 2.65 | 8.55E-07 | 1659 |
| Cluster-40555.178818 | 1.09 | 0.15 | 6.63 | 6.05 | 0 | 0.56 | 3.66 | 6.26 | 3.56 | 2.86E-02 | 4.15 | 2.45E-02 | 530 |
| Cluster-40555.203314 | 0 | 9.57 | 71.51 | 40.69 | 10.16 | 5.2 | 83.49 | 53.03 | 3.56 | 7.12E-03 | 3.19 | 4.50E-03 | 294 |
| Cluster-40555.172768 | 0.41 | 0.42 | 4.24 | 4.5 | 1.6 | 1.74 | 6.58 | 8.2 | 3.56 | 1.60E-04 | 2.22 | 5.94E-04 | 1115 |
| Cluster-40555.188809 | 0.98 | 0.82 | 10.08 | 9.48 | 0.05 | 0.05 | 4.74 | 6.51 | 3.56 | 5.09E-10 | 6.89 | 3.83E-13 | 1230 |
| Cluster-40555.237836 | 0.64 | 0 | 3.47 | 3.07 | 0 | 0.29 | 2.57 | 2.39 | 3.57 | 6.05E-06 | 4.12 | 2.71E-06 | 1790 |
| Cluster-40555.196765 | 0 | 0.21 | 1.07 | 1.15 | 0.41 | 0.38 | 1.86 | 1.55 | 3.57 | 2.60E-02 | 2.17 | 4.30E-02 | 1878 |
| Cluster-40555.173950 | 2.27 | 0 | 12.77 | 10.45 | 0.44 | 0.67 | 8.92 | 5.34 | 3.57 | 2.57E-04 | 3.68 | 3.50E-03 | 563 |

| | | | | | | | | | | | | | |
|---|---|---|---|---|---|---|---|---|---|---|---|---|---|
| Cluster-40555.211005 | 0.57 | 0.66 | 5.53 | 7.6 | 3.3 | 1.57 | 11.37 | 11.49 | 3.57 | 1.19E-06 | 2.32 | 1.36E-05 | 1118 |
| Cluster-40555.204914 | 4.71 | 6.47 | 62.25 | 59.09 | 2.55 | 2.47 | 43.36 | 33.21 | 3.57 | 1.45E-30 | 3.99 | 1.25E-24 | 1113 |
| Cluster-40555.206326 | 3.63 | 1.88 | 28.66 | 30.28 | 8.74 | 8.27 | 24.19 | 25.02 | 3.57 | 5.66E-21 | 1.60 | 1.36E-04 | 1110 |
| Cluster-40555.190411 | 0.09 | 0.13 | 1.26 | 1.09 | 0.07 | 0.07 | 2.11 | 1.69 | 3.58 | 5.93E-04 | 4.86 | 7.94E-10 | 3047 |
| Cluster-40555.213565 | 0.03 | 0.19 | 1.34 | 1.12 | 0.58 | 0.61 | 2.35 | 2.37 | 3.58 | 4.60E-04 | 2.05 | 1.13E-03 | 3059 |
| Cluster-40555.238132 | 0.8 | 0.17 | 6.02 | 4.28 | 0.65 | 1.09 | 4.07 | 4.48 | 3.58 | 1.21E-10 | 2.35 | 2.18E-05 | 2240 |
| Cluster-40555.208241 | 0.09 | 0.43 | 3.08 | 2.67 | 0 | 0 | 1.91 | 2.25 | 3.59 | 4.22E-02 | Inf | 3.64E-03 | 821 |
| Cluster-40555.184295 | 6.51 | 5.66 | 61.81 | 68.99 | 34.82 | 16.99 | 65.75 | 97.12 | 3.59 | 6.32E-10 | 1.72 | 1.75E-02 | 399 |
| Cluster-40555.202249 | 0.21 | 0.52 | 3.56 | 4.53 | 0 | 0.22 | 3.6 | 3.78 | 3.59 | 1.54E-03 | 5.12 | 2.43E-05 | 953 |
| Cluster-40555.190057 | 15.92 | 21.19 | 209.2 | 199.36 | 11.71 | 7.51 | 47.34 | 57.31 | 3.59 | 9.57E-39 | 2.52 | 2.52E-10 | 715 |
| Cluster-40555.161254 | 2.03 | 0.25 | 12.19 | 12.07 | 0.87 | 0.52 | 8.73 | 10.33 | 3.59 | 2.14E-13 | 3.87 | 2.60E-12 | 1332 |
| Cluster-40555.235014 | 0.17 | 0.55 | 4.85 | 3.87 | 0 | 0 | 2.15 | 1.86 | 3.59 | 2.14E-02 | Inf | 2.86E-02 | 689 |
| Cluster-40555.197276 | 1.17 | 2.21 | 21.23 | 17.04 | 1.01 | 0.73 | 11.25 | 8.55 | 3.59 | 2.80E-10 | 3.55 | 1.77E-06 | 780 |
| Cluster-40555.200568 | 3.25 | 8.41 | 57.95 | 72.15 | 0 | 0 | 83.55 | 54.11 | 3.60 | 4.21E-16 | Inf | 1.07E-24 | 886 |
| Cluster-40555.189848 | 2.47 | 1.87 | 22.15 | 25.86 | 14.14 | 11.98 | 33.84 | 36.52 | 3.61 | 4.57E-14 | 1.50 | 5.22E-04 | 839 |
| Cluster-40555.226142 | 1.19 | 0.87 | 10.84 | 11.86 | 3.55 | 5.77 | 13.3 | 12.25 | 3.61 | 8.16E-11 | 1.50 | 3.52E-03 | 1143 |
| Cluster-40555.183818 | 0.27 | 0.75 | 6.94 | 4.86 | 0.63 | 0.22 | 5.21 | 4.31 | 3.61 | 2.47E-08 | 3.57 | 1.01E-07 | 1590 |
| Cluster-40555.194613 | 0.7 | 1.32 | 11.79 | 11.06 | 0.94 | 0.25 | 4.79 | 5.5 | 3.62 | 3.12E-11 | 3.20 | 2.28E-05 | 1207 |
| Cluster-40555.159539 | 0 | 0.43 | 2.61 | 2.3 | 1.15 | 1.7 | 3.54 | 3.91 | 3.62 | 1.69E-06 | 1.44 | 1.41E-02 | 2624 |
| Cluster-40555.185071 | 3.08 | 1.99 | 28.44 | 27.32 | 0.75 | 0.79 | 10.65 | 8.88 | 3.62 | 1.08E-12 | 3.72 | 5.66E-06 | 703 |
| Cluster-40555.172184 | 0.64 | 1.46 | 13.55 | 10.23 | 0.38 | 0.37 | 6.35 | 8.88 | 3.62 | 6.43E-15 | 4.43 | 1.50E-09 | 1613 |
| Cluster-40555.184795 | 16.48 | 20.09 | 202.66 | 207.18 | 55.03 | 48.96 | 148 | 170.69 | 3.62 | 3.38E-49 | 1.69 | 5.62E-08 | 1404 |
| Cluster-40555.193950 | 1.79 | 2.12 | 22.16 | 21.62 | 3.73 | 2.87 | 19.25 | 16.71 | 3.62 | 8.78E-16 | 2.51 | 7.37E-08 | 1018 |
| Cluster-40555.189111 | 0.37 | 0.85 | 6.97 | 6.93 | 0.14 | 0 | 3.6 | 3.07 | 3.63 | 7.19E-13 | 5.83 | 7.41E-13 | 2094 |
| Cluster-40555.173253 | 0.21 | 0.2 | 2.16 | 2.12 | 0.92 | 0.52 | 2.33 | 2.63 | 3.63 | 5.51E-04 | 1.84 | 4.90E-02 | 1763 |
| Cluster-40555.104250 | 0.4 | 0.13 | 3.82 | 2.03 | 0 | 0 | 0.71 | 1.41 | 3.63 | 7.92E-03 | Inf | 3.24E-02 | 1037 |
| Cluster-40555.192812 | 2.5 | 2.17 | 28.64 | 23.93 | 20.7 | 21.59 | 47.55 | 37.33 | 3.63 | 4.51E-31 | 1.07 | 3.07E-03 | 2217 |
| Cluster-40555.214564 | 0.38 | 0.24 | 2.89 | 3.88 | 1.51 | 1.44 | 5.06 | 4.85 | 3.63 | 2.31E-10 | 1.81 | 1.25E-04 | 2965 |
| Cluster-40555.200745 | 0.83 | 1.29 | 11.98 | 11.61 | 0.16 | 0.08 | 4.03 | 5.56 | 3.63 | 5.76E-08 | 5.39 | 4.70E-06 | 859 |
| Cluster-40555.158343 | 0.57 | 0.06 | 3.6 | 3.27 | 1.75 | 2.88 | 9.34 | 7.69 | 3.63 | 1.32E-09 | 1.93 | 1.47E-06 | 2739 |
| Cluster-40555.202157 | 4.05 | 3.11 | 38.65 | 41.68 | 2.74 | 2.48 | 17.66 | 14.03 | 3.64 | 1.19E-22 | 2.66 | 1.73E-07 | 942 |
| Cluster-40555.192020 | 3.75 | 3.44 | 45.88 | 35.91 | 5.01 | 9.63 | 42.73 | 33.54 | 3.64 | 6.11E-30 | 2.43 | 1.12E-12 | 1426 |

| | | | | | | | | | | | | | |
|---|---|---|---|---|---|---|---|---|---|---|---|---|---|
| Cluster-40555.137241 | 0.84 | 0.95 | 12.27 | 8.17 | 0 | 0 | 22.14 | 16.86 | 3.64 | 1.77E-05 | Inf | 4.56E-23 | 723 |
| Cluster-40555.212438 | 5.82 | 10.41 | 100.66 | 86.81 | 1.16 | 0.81 | 88.17 | 86.18 | 3.65 | 1.29E-32 | 6.54 | 1.49E-53 | 872 |
| Cluster-40555.195237 | 14.29 | 19.96 | 196.41 | 196.49 | 0 | 0 | 165.8 | 140.94 | 3.65 | 4.52E-36 | Inf | 6.06E-73 | 618 |
| Cluster-40555.191024 | 0 | 0.8 | 3.96 | 5.25 | 1.07 | 0.71 | 8.19 | 4.6 | 3.65 | 5.97E-05 | 2.89 | 3.45E-03 | 1139 |
| Cluster-40555.224920 | 0.39 | 0 | 1.75 | 2.41 | 0.18 | 0.54 | 1.88 | 2.56 | 3.65 | 9.02E-06 | 2.67 | 2.45E-04 | 2413 |
| Cluster-40555.188243 | 0.35 | 0.5 | 4.95 | 4.89 | 0.82 | 1.33 | 4.68 | 4.18 | 3.65 | 1.03E-07 | 2.09 | 1.38E-03 | 1626 |
| Cluster-40555.295652 | 0.16 | 0.25 | 2.14 | 2.37 | 0.71 | 0.62 | 2.24 | 2.18 | 3.66 | 3.58E-05 | 1.83 | 3.20E-02 | 2127 |
| Cluster-40555.212569 | 0.5 | 0.77 | 8.33 | 6.28 | 0.5 | 0.95 | 6.53 | 5.9 | 3.66 | 1.20E-09 | 3.14 | 1.02E-07 | 1489 |
| Cluster-40555.199414 | 0.53 | 0.68 | 6.48 | 7.82 | 1.86 | 1.66 | 8.47 | 7.04 | 3.66 | 1.51E-10 | 2.20 | 1.95E-05 | 1589 |
| Cluster-40555.192639 | 2.12 | 1.53 | 18.21 | 21.95 | 0.04 | 0 | 56.62 | 41.3 | 3.66 | 1.70E-04 | Inf | 2.25E-21 | 436 |
| Cluster-40555.277905 | 0.08 | 0.07 | 0.86 | 0.77 | 0.03 | 0.12 | 0.98 | 0.51 | 3.66 | 2.38E-02 | 3.50 | 1.90E-02 | 2433 |
| Cluster-40555.190039 | 7.22 | 8.04 | 94.76 | 81.51 | 15.54 | 14.31 | 62.03 | 56.06 | 3.67 | 4.41E-35 | 2.05 | 3.80E-09 | 1007 |
| Cluster-40555.184477 | 4.13 | 3.05 | 41.72 | 41.02 | 19.91 | 9.93 | 42.61 | 46.97 | 3.67 | 2.27E-26 | 1.67 | 6.73E-06 | 1110 |
| Cluster-40555.196874 | 0.78 | 0.76 | 11.12 | 6.95 | 0.87 | 2.19 | 9.76 | 8.98 | 3.67 | 1.03E-06 | 2.65 | 6.56E-06 | 1051 |
| Cluster-40555.170374 | 0 | 1.97 | 11.38 | 12.55 | 3.61 | 3.26 | 10.63 | 10.97 | 3.67 | 1.66E-09 | 1.72 | 4.32E-03 | 984 |
| Cluster-40555.195878 | 0.47 | 0.44 | 5.69 | 4.79 | 0.78 | 0.24 | 3.5 | 3.53 | 3.67 | 1.83E-10 | 2.87 | 8.60E-06 | 2103 |
| Cluster-40555.151231 | 0.29 | 0 | 2.05 | 1.1 | 0.13 | 0 | 1.2 | 1.19 | 3.67 | 1.80E-02 | 4.44 | 1.32E-02 | 1460 |
| Cluster-40555.174080 | 3.48 | 3.13 | 36.94 | 38.7 | 3.61 | 2.89 | 20.01 | 23.48 | 3.67 | 9.79E-10 | 2.80 | 1.86E-04 | 489 |
| Cluster-40555.185807 | 27.32 | 25.13 | 293.96 | 307.72 | 73.83 | 72.73 | 153.9 | 146.68 | 3.67 | 4.44E-32 | 1.09 | 1.55E-02 | 430 |
| Cluster-40555.201575 | 0.41 | 1.07 | 8.92 | 8.67 | 1.76 | 2.98 | 7.92 | 11.62 | 3.67 | 1.74E-03 | 2.14 | 3.70E-02 | 572 |
| Cluster-40555.188950 | 81.5 | 136.81 | 1266.36 | 1288.66 | 613.07 | 604.24 | 1034.3 | 1194.33 | 3.68 | 3.59E-55 | 0.94 | 8.25E-03 | 687 |
| Cluster-40555.183554 | 3.32 | 2.52 | 34.91 | 32.37 | 5.83 | 5.19 | 31.67 | 31.22 | 3.68 | 2.81E-15 | 2.57 | 2.22E-08 | 718 |
| Cluster-40555.160914 | 0.43 | 1.7 | 13.48 | 11.82 | 0.62 | 0.49 | 5.93 | 6.24 | 3.68 | 1.35E-06 | 3.57 | 8.59E-04 | 711 |
| Cluster-40555.218089 | 0.5 | 1.36 | 10.46 | 11.21 | 0.15 | 0.32 | 8.98 | 7.51 | 3.68 | 1.96E-05 | 5.13 | 7.66E-07 | 672 |
| Cluster-40555.180759 | 2.02 | 3.64 | 31.28 | 35.13 | 11.6 | 20.18 | 50.26 | 51.12 | 3.68 | 2.57E-35 | 1.73 | 4.53E-08 | 2178 |
| Cluster-40555.285872 | 0.48 | 0 | 2.99 | 2.4 | 0.09 | 0 | 0.98 | 1.42 | 3.68 | 2.14E-05 | 4.90 | 7.82E-04 | 1872 |
| Cluster-40555.144505 | 0.6 | 0.22 | 5.21 | 4.28 | 1.34 | 0.85 | 3.77 | 5.04 | 3.69 | 2.50E-06 | 2.09 | 5.55E-03 | 1369 |
| Cluster-40555.198152 | 0.35 | 0.49 | 5.41 | 4.51 | 1.14 | 0.78 | 9.27 | 6.27 | 3.69 | 5.94E-05 | 3.08 | 2.03E-06 | 1084 |
| Cluster-40555.180186 | 2.95 | 5.79 | 51.85 | 51.84 | 8.02 | 9.02 | 49.23 | 44.56 | 3.69 | 3.80E-31 | 2.52 | 2.83E-13 | 1203 |
| Cluster-40555.175235 | 0.3 | 0.58 | 6.28 | 4.11 | 0.52 | 0.85 | 5.48 | 8.16 | 3.69 | 5.93E-08 | 3.38 | 2.67E-05 | 1770 |
| Cluster-40555.213628 | 1.24 | 0.56 | 9.19 | 11.41 | 4.45 | 2.97 | 10.72 | 14.33 | 3.69 | 9.70E-05 | 1.83 | 2.96E-02 | 616 |
| Cluster-40555.197309 | 1.83 | 0 | 8.73 | 12.03 | 0.14 | 0.73 | 5.27 | 5.49 | 3.69 | 8.01E-08 | 3.61 | 1.33E-04 | 863 |

| | | | | | | | | | | | | | |
|---|---|---|---|---|---|---|---|---|---|---|---|---|---|
| Cluster-40555.189920 | 4.04 | 4.32 | 44.26 | 53.08 | 3.92 | 0.58 | 60.58 | 59.05 | 3.69 | 2.52E-17 | 4.83 | 2.16E-25 | 624 |
| Cluster-40555.170766 | 0.21 | 0.4 | 3.12 | 4.06 | 0.43 | 0.98 | 5.95 | 2.99 | 3.69 | 1.99E-02 | 2.71 | 4.18E-02 | 751 |
| Cluster-40555.208140 | 0.12 | 0 | 0.59 | 0.83 | 0.15 | 0 | 1.04 | 0.7 | 3.70 | 2.33E-02 | 3.73 | 5.04E-03 | 2567 |
| Cluster-40555.192710 | 181.63 | 156.1 | 1988.04 | 1977.53 | 355.28 | 524.12 | 1789.69 | 2113.35 | 3.70 | 9.75E-59 | 2.21 | 5.51E-12 | 866 |
| Cluster-40555.165902 | 0.07 | 0.26 | 1.88 | 2 | 0.07 | 0 | 1.5 | 0.75 | 3.70 | 1.51E-03 | 5.21 | 2.15E-03 | 1691 |
| Cluster-40555.216633 | 0.1 | 0.78 | 5.19 | 5.2 | 1.07 | 0.31 | 4.19 | 3.48 | 3.71 | 5.07E-07 | 2.60 | 9.38E-04 | 1418 |
| Cluster-40555.194927 | 0.92 | 2.36 | 21.46 | 18.47 | 0.79 | 0.95 | 14.33 | 9.82 | 3.71 | 2.20E-09 | 3.85 | 3.59E-07 | 683 |
| Cluster-40555.194931 | 0.68 | 0.55 | 8.34 | 6.34 | 0.35 | 0.25 | 3.08 | 2.84 | 3.71 | 2.47E-15 | 3.36 | 3.66E-07 | 2378 |
| Cluster-40555.228553 | 0 | 0.2 | 1.09 | 1.21 | 0.27 | 0 | 4.39 | 3.88 | 3.72 | 3.17E-03 | 4.99 | 5.86E-15 | 2409 |
| Cluster-40555.189247 | 8.27 | 6.37 | 98.82 | 75.99 | 4.68 | 3.85 | 28.94 | 29.22 | 3.72 | 2.99E-27 | 2.84 | 3.14E-16 | 1835 |
| Cluster-40555.248174 | 0.07 | 0.3 | 2.76 | 1.98 | 0 | 0.07 | 1.35 | 0.89 | 3.72 | 3.17E-03 | 5.65 | 8.05E-03 | 1340 |
| Cluster-40555.127313 | 0.36 | 0 | 1.66 | 2.53 | 0 | 0 | 1.47 | 1.92 | 3.72 | 3.65E-04 | Inf | 5.43E-07 | 1711 |
| Cluster-40555.270958 | 0.13 | 0.06 | 1.09 | 1.09 | 0.1 | 0.05 | 1.27 | 1.09 | 3.72 | 3.10E-03 | 4.07 | 2.63E-04 | 2493 |
| Cluster-40555.211776 | 0.48 | 0.17 | 4.02 | 3.72 | 0.6 | 0.43 | 3.17 | 2.34 | 3.73 | 1.30E-13 | 2.50 | 4.28E-06 | 3551 |
| Cluster-40555.232594 | 0.47 | 0.34 | 4.12 | 5.68 | 1.6 | 0.87 | 10.15 | 10.02 | 3.73 | 6.95E-08 | 3.10 | 1.27E-10 | 1564 |
| Cluster-40555.167126 | 0.86 | 0 | 4.43 | 5.55 | 0 | 0 | 9.23 | 8.04 | 3.74 | 3.85E-03 | Inf | 6.34E-11 | 713 |
| Cluster-40555.186617 | 1.72 | 2.78 | 28.12 | 26.98 | 2.85 | 1.33 | 19.7 | 24.05 | 3.74 | 1.35E-14 | 3.46 | 4.10E-11 | 775 |
| Cluster-40555.131078 | 1.02 | 1.38 | 16.99 | 12.83 | 2.16 | 2.46 | 9.81 | 9.33 | 3.74 | 1.71E-09 | 2.11 | 2.58E-03 | 829 |
| Cluster-40555.174803 | 1.69 | 2.97 | 23.4 | 33.27 | 0.08 | 0.92 | 14.96 | 12.79 | 3.74 | 1.61E-03 | 4.53 | 3.19E-02 | 350 |
| Cluster-40555.222407 | 0.31 | 0.21 | 2.6 | 3.76 | 0.3 | 0.52 | 3.04 | 3.96 | 3.75 | 5.38E-04 | 3.13 | 7.25E-04 | 1193 |
| Cluster-40555.234762 | 0.13 | 0 | 0.96 | 0.69 | 0.16 | 0 | 0.76 | 0.71 | 3.75 | 2.33E-02 | 3.35 | 4.29E-02 | 2340 |
| Cluster-40555.126500 | 2.24 | 2.37 | 27.77 | 28.8 | 1.79 | 1.3 | 14.46 | 12.71 | 3.75 | 2.66E-25 | 3.20 | 4.63E-12 | 1356 |
| Cluster-40555.230186 | 0.49 | 0.79 | 7.14 | 8.72 | 0.16 | 0.13 | 1.79 | 2.75 | 3.75 | 1.38E-12 | 4.00 | 3.80E-05 | 1693 |
| Cluster-40555.184240 | 0.38 | 0.28 | 4.05 | 4.01 | 0.87 | 0.51 | 7.4 | 7.21 | 3.75 | 2.86E-07 | 3.48 | 4.54E-11 | 1736 |
| Cluster-40555.182405 | 0.71 | 0.19 | 5.78 | 5.13 | 5.82 | 5.1 | 11.98 | 9.42 | 3.76 | 7.98E-11 | 1.04 | 3.85E-02 | 1993 |
| Cluster-40555.210923 | 0.96 | 2.85 | 22.91 | 24.49 | 7.37 | 10.89 | 18.23 | 21.99 | 3.76 | 1.12E-24 | 1.20 | 4.70E-03 | 1533 |
| Cluster-40555.239070 | 0.27 | 0 | 2.27 | 1.13 | 0.12 | 0.06 | 1.34 | 1.1 | 3.76 | 8.21E-03 | 3.62 | 1.67E-02 | 1613 |
| Cluster-40555.243447 | 0.08 | 0.17 | 1.65 | 1.41 | 0.74 | 0.4 | 1.79 | 2.14 | 3.76 | 4.91E-04 | 1.87 | 3.21E-02 | 2335 |
| Cluster-40555.206420 | 1.42 | 0.79 | 13.98 | 13.23 | 0 | 0.11 | 3.44 | 2.73 | 3.76 | 2.00E-10 | 5.39 | 1.59E-04 | 926 |
| Cluster-40555.213544 | 0.67 | 0.51 | 7.34 | 7.12 | 0.1 | 0.51 | 4.89 | 5.41 | 3.76 | 4.17E-12 | 4.10 | 3.37E-11 | 1766 |
| Cluster-40555.187524 | 0.24 | 0 | 1.77 | 1.14 | 0.21 | 0.17 | 1.35 | 1.21 | 3.76 | 2.06E-03 | 2.84 | 1.73E-02 | 2032 |
| Cluster-40555.189530 | 5.26 | 5.8 | 70.3 | 66.38 | 19.84 | 18.53 | 64.98 | 57.71 | 3.76 | 2.52E-42 | 1.74 | 7.02E-08 | 1674 |

| | | | | | | | | | | | | | |
|---|---|---|---|---|---|---|---|---|---|---|---|---|---|
| Cluster-40555.176102 | 2.3 | 1.69 | 26.28 | 22.85 | 3.35 | 2.79 | 16.88 | 17.01 | 3.77 | 1.91E-25 | 2.53 | 4.00E-10 | 1541 |
| Cluster-40555.129821 | 0.25 | 0.11 | 2.87 | 1.63 | 0 | 0.22 | 1.98 | 1.7 | 3.77 | 7.33E-04 | 4.10 | 2.70E-04 | 1622 |
| Cluster-40555.202359 | 0.53 | 0.7 | 7.25 | 8.22 | 0 | 0 | 5.48 | 5.85 | 3.77 | 1.44E-07 | Inf | 1.14E-12 | 1060 |
| Cluster-40555.195967 | 0.5 | 0.3 | 5.26 | 4.46 | 1.79 | 1.19 | 5.22 | 4.85 | 3.77 | 2.48E-09 | 1.83 | 1.67E-03 | 1904 |
| Cluster-40555.175562 | 0.81 | 0.7 | 8.9 | 9.6 | 6.23 | 8.24 | 16.48 | 14.14 | 3.77 | 1.99E-13 | 1.14 | 1.23E-02 | 1583 |
| Cluster-40555.211904 | 0.13 | 0 | 1.09 | 0.54 | 0 | 0.16 | 1.25 | 1.1 | 3.77 | 5.30E-03 | 3.95 | 2.18E-05 | 3114 |
| Cluster-40555.169874 | 1.11 | 0.49 | 10.46 | 8.72 | 0.27 | 0 | 3.03 | 5.05 | 3.78 | 2.36E-04 | 5.02 | 7.40E-03 | 611 |
| Cluster-40555.187370 | 2.47 | 1.36 | 25.24 | 22.02 | 0.28 | 0 | 49.74 | 36.29 | 3.78 | 7.36E-37 | 8.32 | 3.26E-47 | 2986 |
| Cluster-40555.178574 | 0.62 | 0.37 | 5.47 | 7 | 0.12 | 0.05 | 6.8 | 7.18 | 3.78 | 1.33E-11 | 6.66 | 5.70E-23 | 1855 |
| Cluster-40555.206159 | 0.73 | 1.28 | 11.35 | 13.97 | 1.27 | 0 | 4.33 | 3.52 | 3.78 | 1.25E-10 | 2.76 | 9.74E-03 | 981 |
| Cluster-40555.170508 | 0.16 | 0.54 | 4.51 | 4.49 | 0.2 | 0.13 | 6.34 | 7.89 | 3.78 | 1.68E-03 | 5.20 | 2.21E-08 | 851 |
| Cluster-40555.203750 | 0 | 0.5 | 3.18 | 3.25 | 0 | 0 | 2.66 | 2.98 | 3.78 | 2.06E-03 | Inf | 2.17E-06 | 1070 |
| Cluster-40555.161214 | 0.9 | 0.74 | 9.65 | 10.62 | 1.21 | 1.27 | 20.53 | 21.04 | 3.79 | 1.17E-05 | 4.13 | 3.17E-12 | 695 |
| Cluster-40555.181208 | 0.4 | 0.07 | 2.77 | 3.01 | 0.8 | 0.59 | 2.9 | 2.13 | 3.79 | 1.36E-06 | 1.92 | 1.71E-02 | 2036 |
| Cluster-40555.215434 | 0.61 | 0.31 | 5.16 | 6.17 | 0.48 | 0.2 | 4.39 | 3.3 | 3.79 | 8.37E-06 | 3.61 | 3.13E-04 | 1048 |
| Cluster-40555.107060 | 0.14 | 0.4 | 3.63 | 3.58 | 0.14 | 0.31 | 3.79 | 5.39 | 3.79 | 1.74E-04 | 4.40 | 1.35E-07 | 1231 |
| Cluster-40555.206075 | 1 | 0.6 | 9.87 | 9.99 | 2.01 | 1.69 | 9.11 | 10.51 | 3.79 | 5.77E-19 | 2.47 | 3.50E-09 | 2200 |
| Cluster-40555.236178 | 0.19 | 0.52 | 6.16 | 3.26 | 0.96 | 1.3 | 4.09 | 4.79 | 3.79 | 1.07E-03 | 2.05 | 1.33E-02 | 1186 |
| Cluster-40555.247080 | 0.73 | 0.41 | 6.59 | 7.62 | 1.55 | 1.64 | 5.45 | 7.38 | 3.80 | 3.88E-05 | 2.08 | 2.42E-02 | 811 |
| Cluster-40555.189752 | 3.91 | 4.42 | 59.35 | 46.35 | 0.67 | 0.7 | 8.78 | 9.83 | 3.80 | 7.94E-32 | 3.83 | 9.10E-16 | 1871 |
| Cluster-40555.180812 | 0.25 | 0 | 2.06 | 1.33 | 0 | 0 | 0.7 | 1.22 | 3.80 | 4.39E-02 | Inf | 3.26E-02 | 1128 |
| Cluster-40555.188667 | 2.59 | 4.31 | 46.25 | 42.17 | 1.25 | 1.05 | 19.47 | 18.62 | 3.80 | 2.21E-42 | 4.12 | 5.11E-28 | 2408 |
| Cluster-40555.172414 | 0.62 | 0.15 | 6.09 | 3.75 | 0 | 0.06 | 7.66 | 5.65 | 3.81 | 3.13E-06 | 7.40 | 1.82E-19 | 1495 |
| Cluster-40555.183979 | 0.84 | 1.77 | 18.18 | 15.48 | 1.86 | 2.35 | 15.41 | 28.14 | 3.81 | 8.50E-12 | 3.44 | 7.91E-03 | 895 |
| Cluster-40555.208896 | 2 | 1.63 | 24.85 | 21.16 | 4.98 | 4.67 | 16.03 | 15.9 | 3.81 | 2.09E-11 | 1.79 | 5.00E-03 | 699 |
| Cluster-40555.162876 | 0.77 | 0.82 | 10.08 | 10.14 | 1.51 | 1.76 | 4.69 | 6.33 | 3.81 | 7.68E-17 | 1.83 | 9.74E-04 | 1883 |
| Cluster-40555.165091 | 0.27 | 0.51 | 5.53 | 4.58 | 0 | 0 | 2.27 | 1.38 | 3.81 | 3.90E-06 | Inf | 5.18E-05 | 1242 |
| Cluster-40555.186837 | 14.2 | 14.33 | 178.94 | 183.89 | 55.79 | 40.49 | 226.65 | 217.79 | 3.81 | 1.14E-44 | 2.28 | 1.89E-13 | 843 |
| Cluster-40555.220383 | 0.29 | 0.16 | 3.16 | 2.54 | 0.4 | 0.6 | 1.82 | 2.33 | 3.81 | 2.11E-06 | 2.09 | 1.65E-02 | 2063 |
| Cluster-40555.164037 | 0.1 | 0.52 | 3.71 | 4.44 | 0 | 0 | 11.55 | 7.06 | 3.82 | 1.19E-02 | Inf | 1.08E-10 | 726 |
| Cluster-40555.205895 | 0.54 | 0.31 | 5.5 | 5.31 | 1.28 | 3.35 | 12.02 | 10.68 | 3.82 | 1.04E-08 | 2.33 | 1.22E-07 | 1578 |
| Cluster-40555.181690 | 0.72 | 0.89 | 10.06 | 10.68 | 0.71 | 0.88 | 6.38 | 6.92 | 3.82 | 3.21E-10 | 3.10 | 7.33E-06 | 1096 |

| | | | | | | | | | | | | | |
|---|---|---|---|---|---|---|---|---|---|---|---|---|---|
| Cluster-40555.196792 | 0.32 | 0.3 | 3.88 | 4.11 | 0.9 | 0.68 | 4.9 | 5.26 | 3.83 | 6.73E-21 | 2.76 | 5.69E-13 | 5718 |
| Cluster-40555.190562 | 0.87 | 1.47 | 16.45 | 14.19 | 3.04 | 4.61 | 8.87 | 10.88 | 3.83 | 4.87E-15 | 1.42 | 1.38E-02 | 1194 |
| Cluster-40555.239726 | 0 | 0.53 | 4.11 | 3.1 | 0.61 | 0.23 | 4.32 | 4.12 | 3.83 | 4.82E-05 | 3.41 | 9.44E-06 | 1384 |
| Cluster-40555.204203 | 1.52 | 1.91 | 25.66 | 19.04 | 14.67 | 6.25 | 15.92 | 17.72 | 3.84 | 3.90E-20 | 1.49 | 9.93E-05 | 2174 |
| Cluster-40555.201638 | 0.67 | 4.94 | 37.16 | 37.99 | 10.14 | 9.96 | 31.47 | 34.46 | 3.84 | 1.08E-16 | 1.78 | 1.37E-04 | 697 |
| Cluster-40555.189650 | 0.26 | 0.54 | 5.85 | 4.71 | 1.04 | 0.84 | 7.17 | 5.04 | 3.84 | 2.58E-13 | 2.77 | 3.67E-09 | 2598 |
| Cluster-40555.181853 | 1.17 | 0.65 | 12.53 | 10.87 | 0.65 | 0.58 | 6.13 | 6.93 | 3.84 | 1.40E-16 | 3.48 | 9.60E-10 | 1620 |
| Cluster-40555.198240 | 0.72 | 0.76 | 11.14 | 8.31 | 9.71 | 10.58 | 29.69 | 25.3 | 3.84 | 3.90E-21 | 1.50 | 7.53E-06 | 2839 |
| Cluster-40555.189387 | 3.83 | 3.75 | 45.94 | 52.29 | 4.88 | 5.36 | 43.58 | 46.67 | 3.84 | 3.80E-43 | 3.20 | 2.18E-23 | 2222 |
| Cluster-40555.129207 | 0.69 | 0.06 | 5.21 | 4.54 | 0.89 | 0.58 | 4.68 | 4.36 | 3.84 | 1.99E-04 | 2.67 | 6.81E-03 | 935 |
| Cluster-40555.176023 | 0.9 | 3.38 | 28.07 | 28.33 | 5.78 | 3.93 | 16.35 | 13.84 | 3.84 | 1.07E-25 | 1.70 | 1.37E-04 | 1359 |
| Cluster-40555.184760 | 1.8 | 1.64 | 21.1 | 23.54 | 2.62 | 3.41 | 16.72 | 10.43 | 3.84 | 3.70E-26 | 2.22 | 9.26E-04 | 1630 |
| Cluster-40555.150134 | 0.47 | 0.38 | 5.74 | 5.37 | 0.26 | 0.47 | 6.62 | 4.94 | 3.84 | 7.23E-04 | 3.97 | 1.03E-04 | 779 |
| Cluster-40555.152014 | 0.41 | 0.63 | 7.7 | 6.2 | 0.13 | 0.23 | 4.17 | 4.35 | 3.84 | 7.14E-08 | 4.62 | 3.25E-07 | 1194 |
| Cluster-40555.167669 | 0.11 | 0.08 | 1.04 | 1.38 | 0.15 | 0.5 | 3.59 | 2.41 | 3.85 | 3.59E-02 | 3.25 | 1.66E-04 | 1466 |
| Cluster-40555.225155 | 0.34 | 0.44 | 5.51 | 4.62 | 0.96 | 1.38 | 6.25 | 6.9 | 3.85 | 5.12E-08 | 2.56 | 3.98E-06 | 1560 |
| Cluster-40555.187775 | 133.29 | 50.88 | 1192.32 | 1138.62 | 1.65 | 2.75 | 505.3 | 630.69 | 3.86 | 3.05E-52 | 8.05 | 1.57E-54 | 412 |
| Cluster-40555.43433 | 0.43 | 0.44 | 5.58 | 5.88 | 1.15 | 1.43 | 5.42 | 8.07 | 3.86 | 1.43E-03 | 2.45 | 1.35E-02 | 714 |
| Cluster-40555.178031 | 0.99 | 3.32 | 26.26 | 32.16 | 2.54 | 2.44 | 46.19 | 37.89 | 3.86 | 1.87E-16 | 4.15 | 1.06E-21 | 790 |
| Cluster-40555.163978 | 0.97 | 1.06 | 13.09 | 13.73 | 4.6 | 4.46 | 21.61 | 26.02 | 3.86 | 2.17E-10 | 2.46 | 3.93E-08 | 906 |
| Cluster-40555.228577 | 0.42 | 0.51 | 6.2 | 6.29 | 0 | 0 | 2.99 | 4.74 | 3.87 | 4.11E-02 | Inf | 2.76E-02 | 494 |
| Cluster-40555.187999 | 0.44 | 0.8 | 9.84 | 6.85 | 0.6 | 0.63 | 5 | 4.88 | 3.87 | 9.19E-06 | 3.06 | 3.60E-03 | 812 |
| Cluster-40555.277436 | 0 | 0.52 | 3.25 | 3.69 | 0.1 | 0.19 | 1.48 | 2.11 | 3.87 | 2.20E-05 | 3.54 | 3.97E-03 | 1457 |
| Cluster-40555.167396 | 0.6 | 0.49 | 7.09 | 7.34 | 0.58 | 0.1 | 9.55 | 7.82 | 3.87 | 9.96E-05 | 4.76 | 1.24E-07 | 748 |
| Cluster-40555.188021 | 0.48 | 0.07 | 4.07 | 2.8 | 0 | 0 | 7.76 | 6.93 | 3.88 | 2.20E-03 | Inf | 1.62E-14 | 978 |
| Cluster-40555.158776 | 0 | 2.25 | 14.19 | 18.14 | 1.49 | 3.09 | 33.79 | 19.01 | 3.89 | 2.09E-02 | 3.54 | 3.06E-03 | 363 |
| Cluster-40555.170083 | 5.5 | 6.77 | 79.27 | 86.66 | 3.95 | 4.62 | 66.08 | 77.77 | 3.89 | 2.15E-35 | 4.13 | 3.41E-29 | 894 |
| Cluster-40555.188666 | 7.6 | 4.67 | 78.07 | 85.73 | 0 | 0.59 | 64.14 | 55.03 | 3.90 | 1.35E-24 | 7.60 | 6.17E-36 | 586 |
| Cluster-40555.211845 | 0.29 | 0 | 1.89 | 1.93 | 0 | 0 | 2.32 | 2.35 | 3.90 | 1.41E-03 | Inf | 9.83E-09 | 1579 |
| Cluster-40555.178998 | 0 | 0.23 | 1.43 | 1.7 | 0.25 | 0.27 | 2.42 | 3.96 | 3.90 | 3.91E-03 | 3.66 | 6.13E-04 | 1696 |
| Cluster-40555.153104 | 0.47 | 0.71 | 8.72 | 7.05 | 0 | 0 | 2.11 | 3.44 | 3.90 | 7.08E-16 | Inf | 2.69E-08 | 2160 |
| Cluster-40555.174748 | 1.35 | 0.69 | 15.02 | 13.15 | 0.81 | 0.58 | 15.36 | 14.17 | 3.90 | 1.18E-09 | 4.50 | 1.11E-12 | 821 |

| | | | | | | | | | | | | | |
|---|---|---|---|---|---|---|---|---|---|---|---|---|---|
| Cluster-40555.200989 | 1.28 | 1.43 | 16.08 | 21.02 | 4.81 | 2.85 | 13.15 | 14.27 | 3.90 | 3.15E-11 | 1.92 | 2.95E-03 | 754 |
| Cluster-40555.164020 | 1.49 | 4.73 | 41.65 | 44.7 | 3.06 | 2.91 | 24.21 | 28.85 | 3.90 | 2.76E-31 | 3.22 | 3.98E-16 | 1227 |
| Cluster-40555.176533 | 1.96 | 8.34 | 72.9 | 71.34 | 0.19 | 0.11 | 17.5 | 12.05 | 3.91 | 6.91E-30 | 6.86 | 1.10E-17 | 801 |
| Cluster-40555.191766 | 10.58 | 30.23 | 268.79 | 296.41 | 111.95 | 112.52 | 212.93 | 205.61 | 3.91 | 4.10E-54 | 0.96 | 6.53E-03 | 1366 |
| Cluster-40555.250593 | 0.24 | 0 | 1.63 | 1.5 | 0.3 | 0.88 | 1.99 | 2.01 | 3.91 | 3.29E-05 | 1.81 | 1.47E-02 | 2686 |
| Cluster-40555.162685 | 0 | 0.84 | 6.65 | 5.47 | 1.07 | 2.57 | 7.66 | 6.06 | 3.91 | 8.80E-06 | 1.96 | 5.83E-03 | 1014 |
| Cluster-40555.208903 | 0.42 | 0.3 | 6.21 | 3.6 | 0.53 | 1.1 | 3.92 | 3.01 | 3.91 | 2.00E-05 | 2.15 | 7.00E-04 | 2058 |
| Cluster-40555.186419 | 114.03 | 98.75 | 1345.49 | 1546.33 | 493.87 | 436.08 | 1244.26 | 1160.44 | 3.92 | 1.91E-42 | 1.43 | 5.07E-06 | 483 |
| Cluster-40555.190326 | 3.76 | 5.42 | 72.01 | 55.33 | 12.08 | 10.88 | 58.43 | 53.77 | 3.92 | 5.97E-28 | 2.36 | 4.50E-13 | 1491 |
| Cluster-40555.205816 | 0.25 | 0.19 | 3.06 | 3.29 | 0.44 | 0.44 | 2.55 | 2.18 | 3.93 | 2.27E-05 | 2.47 | 1.46E-02 | 1501 |
| Cluster-40555.177902 | 0.27 | 0.57 | 6.34 | 5.88 | 0.2 | 0.46 | 3.24 | 2.95 | 3.93 | 2.12E-04 | 3.31 | 2.71E-02 | 796 |
| Cluster-40555.219797 | 0.1 | 0.09 | 1.37 | 1.1 | 1.15 | 0.49 | 2.42 | 1.93 | 3.93 | 6.23E-06 | 1.49 | 1.84E-02 | 3958 |
| Cluster-40555.181524 | 1.01 | 0.92 | 7.59 | 17.3 | 0 | 1.39 | 16.04 | 12.86 | 3.93 | 3.03E-02 | 4.54 | 2.43E-04 | 428 |
| Cluster-40555.134567 | 0.16 | 0.12 | 1.95 | 2.04 | 0.42 | 0.22 | 2.04 | 2.3 | 3.94 | 6.97E-04 | 2.89 | 3.53E-03 | 1645 |
| Cluster-40555.130264 | 0 | 0.26 | 1.64 | 2.24 | 0 | 0.11 | 1.94 | 1.38 | 3.94 | 1.58E-02 | 4.90 | 5.00E-03 | 1148 |
| Cluster-40555.200834 | 1.68 | 1.03 | 16.96 | 19.74 | 2.46 | 2.11 | 13.97 | 11.24 | 3.94 | 5.56E-13 | 2.51 | 2.60E-05 | 848 |
| Cluster-40555.275999 | 0.16 | 0.22 | 2.82 | 2.58 | 0.96 | 0.61 | 3.18 | 3.47 | 3.94 | 2.37E-06 | 2.14 | 1.37E-03 | 2061 |
| Cluster-40555.224376 | 0.17 | 0 | 0.81 | 1.57 | 0 | 0.3 | 3.94 | 4.58 | 3.94 | 4.45E-02 | 4.76 | 1.23E-08 | 1346 |
| Cluster-40555.199652 | 0.03 | 0.4 | 3.04 | 3.15 | 0.77 | 0.12 | 1.8 | 1.79 | 3.94 | 8.58E-07 | 2.13 | 4.21E-02 | 1967 |
| Cluster-40555.188019 | 16.45 | 11.12 | 186.41 | 194.82 | 84.63 | 75.69 | 199.99 | 176.68 | 3.95 | 5.34E-38 | 1.29 | 3.11E-04 | 565 |
| Cluster-40555.130061 | 0.19 | 0.89 | 6.85 | 8.21 | 0.6 | 0.39 | 5.32 | 7.24 | 3.95 | 2.72E-06 | 3.74 | 1.41E-05 | 891 |
| Cluster-40555.176345 | 0.33 | 0 | 3.11 | 1.51 | 2.2 | 0.77 | 4.06 | 3.95 | 3.96 | 3.82E-03 | 1.52 | 3.25E-03 | 3399 |
| Cluster-40555.247902 | 0.34 | 0 | 2.26 | 2.07 | 0 | 0.02 | 0.96 | 1.71 | 3.96 | 2.50E-03 | Inf | 6.25E-04 | 1315 |
| Cluster-40555.178948 | 4.38 | 5.07 | 69.5 | 65.77 | 23.38 | 22.42 | 63.47 | 57.13 | 3.97 | 3.65E-16 | 1.46 | 4.15E-03 | 476 |
| Cluster-40555.144177 | 0.13 | 0.57 | 5.52 | 4.56 | 0.11 | 0.29 | 2.55 | 2.68 | 3.97 | 4.08E-13 | 3.83 | 2.34E-08 | 2542 |
| Cluster-40555.148191 | 1.29 | 1.72 | 22.12 | 21.17 | 5 | 5.34 | 13.96 | 11.38 | 3.97 | 6.48E-16 | 1.35 | 2.99E-02 | 920 |
| Cluster-40555.177845 | 1.19 | 0.76 | 14.79 | 12.92 | 2.81 | 2.25 | 14.29 | 13.17 | 3.98 | 3.83E-27 | 2.51 | 1.55E-11 | 2480 |
| Cluster-40555.203659 | 0 | 0.34 | 1.92 | 3.17 | 0.48 | 0.38 | 1.92 | 1.29 | 3.98 | 1.67E-04 | 1.95 | 1.70E-02 | 2945 |
| Cluster-40555.193303 | 4.45 | 2.92 | 51.66 | 52.09 | 4.43 | 4.89 | 28.89 | 27.11 | 3.98 | 5.03E-12 | 2.64 | 2.48E-04 | 450 |
| Cluster-40555.231739 | 0.11 | 0.3 | 2.73 | 3.81 | 0.59 | 0.34 | 3.3 | 3.85 | 3.98 | 1.51E-03 | 3.08 | 3.95E-03 | 1012 |
| Cluster-40555.150601 | 0.27 | 0 | 1.88 | 1.93 | 0.95 | 1.01 | 4.27 | 3.94 | 3.99 | 5.53E-03 | 2.15 | 7.35E-03 | 1301 |
| Cluster-40555.191557 | 0.33 | 0.5 | 5.48 | 6.53 | 1.21 | 0.62 | 3.22 | 3.04 | 3.99 | 1.27E-17 | 1.84 | 1.60E-03 | 2902 |

| | | | | | | | | | | | | | |
|---|---|---|---|---|---|---|---|---|---|---|---|---|---|
| Cluster-40555.123347 | 0.06 | 0.05 | 0.81 | 0.74 | 0.06 | 0.02 | 1.07 | 1.04 | 3.99 | 1.36E-03 | 4.97 | 9.45E-07 | 3521 |
| Cluster-40555.199917 | 0.44 | 0.12 | 4.04 | 3.7 | 2.84 | 1.87 | 8.78 | 7.04 | 3.99 | 8.05E-06 | 1.81 | 1.50E-03 | 1356 |
| Cluster-40555.187670 | 85.74 | 126.61 | 1480.79 | 1623.87 | 536.04 | 516.29 | 1019.02 | 1019.19 | 4.00 | 3.12E-64 | 1.02 | 2.97E-03 | 648 |
| Cluster-40555.166357 | 1.05 | 0.87 | 15.98 | 11.73 | 3.23 | 1.17 | 11.09 | 10.54 | 4.00 | 3.13E-19 | 2.39 | 8.87E-08 | 1746 |
| Cluster-40555.179468 | 1.26 | 1.3 | 18.99 | 18.03 | 1.57 | 2.89 | 16.78 | 11.67 | 4.01 | 7.80E-20 | 2.71 | 1.05E-07 | 1278 |
| Cluster-40555.181125 | 1.38 | 1.12 | 17.96 | 18.83 | 2.67 | 1.18 | 14.58 | 12.91 | 4.01 | 3.77E-20 | 2.91 | 5.44E-10 | 1290 |
| Cluster-40555.202764 | 0.11 | 0.38 | 2.91 | 3.74 | 0 | 0 | 2.52 | 4.97 | 4.01 | 2.39E-02 | Inf | 4.55E-04 | 724 |
| Cluster-40555.192153 | 19.87 | 44.15 | 513.44 | 442.06 | 52.7 | 67.89 | 225.8 | 164.13 | 4.01 | 3.58E-52 | 1.74 | 2.19E-05 | 582 |
| Cluster-40555.186946 | 5.12 | 5.65 | 80.46 | 78.69 | 18.55 | 13.34 | 88.69 | 67.32 | 4.02 | 4.67E-41 | 2.36 | 6.09E-13 | 1109 |
| Cluster-40555.185160 | 0.35 | 1.1 | 10.65 | 10.98 | 1.12 | 0.87 | 10.06 | 14.69 | 4.02 | 2.49E-19 | 3.70 | 1.56E-06 | 1945 |
| Cluster-40555.216451 | 2.91 | 2.74 | 42.65 | 40.35 | 9.48 | 7.57 | 39.92 | 34.4 | 4.02 | 2.49E-15 | 2.19 | 9.56E-06 | 580 |
| Cluster-40555.208108 | 0 | 0.13 | 1.03 | 1.1 | 0.12 | 0.06 | 2.57 | 1.99 | 4.02 | 2.79E-02 | 4.62 | 4.24E-06 | 1708 |
| Cluster-40555.211498 | 0.79 | 0.99 | 12.45 | 13.87 | 1.29 | 1.31 | 9.68 | 8.92 | 4.02 | 2.31E-17 | 2.91 | 1.09E-08 | 1430 |
| Cluster-40555.155197 | 1.93 | 1.97 | 27.49 | 29.61 | 8.22 | 4.01 | 35.04 | 22.91 | 4.02 | 5.56E-07 | 2.32 | 1.66E-03 | 441 |
| Cluster-40555.209562 | 0.08 | 0.1 | 1.39 | 1 | 0.1 | 0.09 | 1.14 | 0.84 | 4.03 | 1.34E-03 | 3.38 | 6.24E-03 | 2416 |
| Cluster-40555.87180 | 0.31 | 0 | 2.09 | 2.09 | 0.29 | 0 | 2.8 | 3.97 | 4.03 | 1.28E-02 | 4.68 | 4.09E-05 | 1082 |
| Cluster-40555.170566 | 0 | 0.13 | 1.29 | 0.8 | 1.19 | 0.3 | 2.36 | 2.33 | 4.04 | 6.60E-05 | 1.75 | 3.52E-03 | 3845 |
| Cluster-40555.237291 | 0.25 | 0.14 | 2.64 | 3.01 | 0 | 0.18 | 2.19 | 1.39 | 4.04 | 2.05E-03 | 4.31 | 1.02E-02 | 1081 |
| Cluster-40555.189154 | 2.61 | 3.4 | 45.63 | 44.45 | 0.1 | 0.36 | 42.94 | 40.59 | 4.04 | 4.47E-47 | 7.50 | 1.02E-72 | 2408 |
| Cluster-40555.42768 | 0.24 | 0.35 | 3.7 | 5.16 | 0 | 0.06 | 2.09 | 3.46 | 4.04 | 4.38E-05 | 6.51 | 1.70E-05 | 1061 |
| Cluster-40555.232104 | 0.23 | 0.32 | 3.18 | 4.91 | 0.38 | 0.22 | 2.48 | 3.53 | 4.04 | 7.67E-04 | 3.40 | 1.26E-02 | 899 |
| Cluster-40555.146778 | 0.4 | 0.2 | 4.19 | 5.08 | 0.75 | 0.78 | 4.92 | 3.7 | 4.04 | 1.90E-03 | 2.53 | 4.74E-02 | 763 |
| Cluster-40555.202660 | 0.09 | 0.03 | 0.71 | 1.05 | 0.33 | 0.53 | 2.83 | 2.8 | 4.05 | 3.73E-02 | 2.78 | 3.75E-04 | 1784 |
| Cluster-40555.170658 | 1.72 | 1.26 | 22.59 | 22.42 | 0.18 | 0.46 | 11.54 | 8.71 | 4.05 | 4.27E-22 | 4.96 | 4.84E-16 | 1205 |
| Cluster-40555.155933 | 0.1 | 0.13 | 1.94 | 1.5 | 0.13 | 0 | 1.7 | 1.8 | 4.05 | 2.65E-03 | 4.77 | 1.58E-04 | 1605 |
| Cluster-40555.176117 | 1.02 | 1.34 | 15.61 | 19.21 | 0.94 | 0 | 19.78 | 20.15 | 4.05 | 2.87E-15 | 5.49 | 3.02E-23 | 993 |
| Cluster-40555.183412 | 0.35 | 0.17 | 3.15 | 4.08 | 0 | 0 | 4.38 | 3.28 | 4.05 | 5.66E-04 | Inf | 5.63E-08 | 997 |
| Cluster-40555.185513 | 20.63 | 37.91 | 455.7 | 439.63 | 189.16 | 196.38 | 352.43 | 398.33 | 4.06 | 2.93E-65 | 1.03 | 2.37E-03 | 1694 |
| Cluster-40555.191429 | 3.21 | 1.62 | 36.43 | 35.54 | 2.01 | 1.49 | 21.17 | 21.82 | 4.06 | 7.17E-31 | 3.69 | 4.97E-18 | 1255 |
| Cluster-40555.234281 | 0.11 | 0.12 | 1.56 | 1.77 | 0 | 0.3 | 1.41 | 1.79 | 4.06 | 1.67E-02 | 3.45 | 2.27E-02 | 1252 |
| Cluster-40555.189646 | 1.1 | 0 | 9.05 | 7.27 | 3.25 | 5.93 | 11.42 | 16.59 | 4.07 | 7.75E-07 | 1.66 | 3.54E-02 | 865 |
| Cluster-40555.157957 | 1.59 | 2.16 | 28.5 | 28.81 | 2.25 | 0.41 | 24.94 | 11.44 | 4.07 | 3.90E-05 | 3.78 | 4.56E-02 | 387 |

| | | | | | | | | | | | | | |
|---|---|---|---|---|---|---|---|---|---|---|---|---|---|
| Cluster-40555.160153 | 0.09 | 0.21 | 3.11 | 1.86 | 0.72 | 0 | 3.29 | 2.7 | 4.07 | 7.00E-05 | 3.20 | 7.43E-05 | 1718 |
| Cluster-40555.188171 | 9.63 | 40.8 | 402.56 | 387.77 | 26.93 | 8.12 | 230.41 | 290.4 | 4.08 | 3.92E-62 | 3.99 | 2.93E-16 | 1213 |
| Cluster-40555.216576 | 0.03 | 0.17 | 1.63 | 1.4 | 0.7 | 0.38 | 2 | 2.14 | 4.08 | 2.64E-05 | 2.02 | 5.31E-03 | 2817 |
| Cluster-40555.188845 | 21.2 | 19.27 | 383.01 | 240.87 | 0.3 | 0.31 | 41.64 | 46.66 | 4.08 | 1.94E-08 | 7.24 | 4.24E-53 | 1208 |
| Cluster-40555.219937 | 0.8 | 0 | 4.42 | 7.43 | 3.33 | 2.41 | 6.52 | 5.47 | 4.09 | 6.21E-05 | 1.13 | 1.62E-02 | 3618 |
| Cluster-40555.244642 | 0.17 | 0.07 | 2.03 | 1.73 | 0.15 | 0 | 2.28 | 1.16 | 4.09 | 1.37E-02 | 4.57 | 6.10E-03 | 1170 |
| Cluster-40555.185931 | 0.29 | 0.41 | 5.64 | 5.33 | 0.79 | 0.26 | 5.88 | 5.83 | 4.09 | 2.28E-16 | 3.58 | 3.92E-14 | 2830 |
| Cluster-40555.138443 | 0 | 0.15 | 1 | 1.23 | 0 | 0 | 1.67 | 1.82 | 4.09 | 2.64E-03 | Inf | 4.93E-10 | 2271 |
| Cluster-40555.140562 | 0.19 | 0.25 | 3.74 | 3.18 | 0.14 | 0.24 | 1.88 | 2.08 | 4.09 | 4.58E-08 | 3.42 | 1.40E-04 | 2017 |
| Cluster-40555.193603 | 0.42 | 0 | 3.54 | 2.58 | 0 | 1.22 | 2.93 | 2.43 | 4.10 | 5.84E-10 | 2.14 | 5.65E-03 | 2740 |
| Cluster-40555.195496 | 0.59 | 0.56 | 9.5 | 8.42 | 1.99 | 1.4 | 7.2 | 6.44 | 4.10 | 5.50E-07 | 2.09 | 1.61E-02 | 834 |
| Cluster-40555.189899 | 0.39 | 0.34 | 6.66 | 4.61 | 0.09 | 0 | 2.45 | 3.31 | 4.10 | 2.95E-09 | 6.43 | 8.46E-09 | 1541 |
| Cluster-40555.190799 | 1.81 | 0.79 | 22.11 | 17.93 | 0.01 | 0.72 | 6.08 | 8.56 | 4.10 | 4.99E-31 | 4.39 | 6.67E-09 | 2057 |
| Cluster-40555.203156 | 0 | 0.22 | 1.45 | 1.93 | 1.04 | 0.83 | 3.88 | 3.18 | 4.10 | 6.28E-03 | 1.96 | 2.01E-02 | 1418 |
| Cluster-40555.221885 | 0.08 | 0.26 | 2.35 | 2.85 | 0.73 | 0.92 | 4.31 | 2.7 | 4.11 | 2.60E-07 | 2.14 | 1.08E-03 | 2297 |
| Cluster-40555.189257 | 78.35 | 92.31 | 1298.37 | 1373.7 | 697.6 | 737.06 | 1090.35 | 1293.72 | 4.11 | 6.68E-68 | 0.80 | 3.83E-02 | 809 |
| Cluster-40555.170098 | 0.16 | 0.48 | 6.11 | 4.14 | 0.61 | 1.53 | 6.11 | 6.44 | 4.11 | 4.36E-11 | 2.60 | 1.50E-09 | 2885 |
| Cluster-40555.177732 | 2.09 | 1.2 | 25.56 | 25.49 | 4 | 2.94 | 45.97 | 43.31 | 4.11 | 3.97E-22 | 3.75 | 2.73E-23 | 1068 |
| Cluster-40555.164443 | 1.66 | 0.85 | 20.9 | 17.99 | 0.25 | 1.41 | 22.11 | 26.91 | 4.12 | 3.16E-16 | 4.91 | 1.42E-23 | 965 |
| Cluster-40555.210208 | 0.29 | 0.42 | 5.42 | 5.94 | 0.22 | 0.08 | 2.58 | 2.42 | 4.12 | 1.42E-07 | 3.94 | 7.94E-04 | 1225 |
| Cluster-40555.179110 | 2.51 | 5.34 | 60.4 | 65.8 | 7.74 | 8.11 | 34.64 | 50.75 | 4.12 | 1.09E-08 | 2.51 | 2.71E-03 | 361 |
| Cluster-40555.193531 | 0.27 | 0 | 1.9 | 2.37 | 0 | 0 | 1.2 | 0.76 | 4.13 | 5.60E-06 | Inf | 3.10E-05 | 2149 |
| Cluster-40555.194614 | 0.41 | 0.55 | 7.41 | 7.95 | 3.81 | 3.11 | 22.37 | 24.46 | 4.13 | 2.02E-21 | 2.83 | 2.33E-17 | 2823 |
| Cluster-40555.235822 | 0.46 | 0.37 | 7.22 | 7.29 | 1.24 | 1.17 | 22.63 | 25.83 | 4.13 | 4.06E-04 | 4.40 | 1.83E-13 | 647 |
| Cluster-40555.161524 | 0.77 | 1.99 | 25.18 | 19.52 | 4.93 | 4.77 | 25.53 | 21.86 | 4.13 | 3.48E-30 | 2.35 | 7.19E-12 | 2187 |
| Cluster-40555.188524 | 1.76 | 5.21 | 68.91 | 45.12 | 0 | 0 | 52.68 | 49.59 | 4.13 | 3.06E-09 | Inf | 1.52E-24 | 458 |
| Cluster-40555.138411 | 0.1 | 0.09 | 1.26 | 1.75 | 0.3 | 0.05 | 4.51 | 1.95 | 4.14 | 3.02E-02 | 4.27 | 2.20E-02 | 1193 |
| Cluster-40555.56629 | 0.08 | 0.27 | 2.58 | 3.1 | 0 | 0.08 | 2.09 | 2.38 | 4.14 | 1.25E-02 | 5.78 | 4.15E-03 | 863 |
| Cluster-40555.233365 | 0.48 | 0.09 | 3.74 | 5.1 | 0 | 0 | 2.48 | 2.02 | 4.14 | 5.19E-11 | Inf | 1.53E-11 | 2068 |
| Cluster-40555.195759 | 2.55 | 3.88 | 53.47 | 50.33 | 7.78 | 7.93 | 58.26 | 44.02 | 4.14 | 6.04E-23 | 2.76 | 1.12E-11 | 683 |
| Cluster-40555.206143 | 0.67 | 0.36 | 7.74 | 8.65 | 1.82 | 1.61 | 10.45 | 12.43 | 4.15 | 1.63E-15 | 2.81 | 8.27E-11 | 1784 |
| Cluster-40555.189114 | 0.54 | 0.31 | 7.13 | 6.57 | 3.55 | 3.72 | 7.3 | 7.18 | 4.15 | 7.16E-19 | 1.06 | 3.64E-02 | 2659 |

| | | | | | | | | | | | | | |
|---|---|---|---|---|---|---|---|---|---|---|---|---|---|
| Cluster-40555.185410 | 0.61 | 3.19 | 32.44 | 31.29 | 1.85 | 0.64 | 19.54 | 15.78 | 4.15 | 6.08E-04 | 3.89 | 3.36E-02 | 343 |
| Cluster-40555.87987 | 0.21 | 0 | 1.45 | 1.49 | 0.36 | 0.65 | 1.85 | 2.15 | 4.15 | 1.89E-03 | 2.02 | 4.67E-02 | 1777 |
| Cluster-40555.128587 | 0.13 | 0.25 | 3.09 | 3.72 | 0.46 | 0.3 | 6.44 | 3 | 4.15 | 3.52E-03 | 3.65 | 2.25E-02 | 875 |
| Cluster-40555.195276 | 0.16 | 0.49 | 4.93 | 5.78 | 0.38 | 0.07 | 7.66 | 4.81 | 4.15 | 1.64E-10 | 4.90 | 3.58E-09 | 1737 |
| Cluster-40555.180294 | 0.2 | 0.27 | 2.48 | 5.17 | 0 | 0 | 3.23 | 3.45 | 4.15 | 7.88E-03 | Inf | 8.37E-13 | 1625 |
| Cluster-40555.181975 | 6.17 | 7.13 | 113.06 | 102.85 | 65.57 | 50.68 | 140.68 | 118.32 | 4.16 | 1.10E-29 | 1.22 | 1.74E-03 | 562 |
| Cluster-40555.167595 | 1.94 | 0.98 | 22.94 | 23.51 | 5.25 | 5.68 | 21.26 | 26.2 | 4.16 | 2.50E-22 | 2.19 | 8.49E-08 | 1134 |
| Cluster-40555.195074 | 3.19 | 1.98 | 34.43 | 47.82 | 0.45 | 0 | 3.57 | 3.05 | 4.16 | 8.46E-11 | 3.97 | 2.96E-02 | 680 |
| Cluster-40555.209194 | 0.9 | 1.01 | 15.72 | 15.4 | 18.54 | 21.14 | 36.48 | 47.48 | 4.16 | 1.47E-14 | 1.15 | 1.26E-02 | 1014 |
| Cluster-40555.240207 | 0.08 | 0.07 | 1.29 | 1.21 | 0.97 | 1.54 | 4.79 | 5.54 | 4.17 | 3.00E-02 | 2.10 | 1.51E-03 | 1412 |
| Cluster-40555.175696 | 0.71 | 1.88 | 20.81 | 22.18 | 0 | 0.08 | 9.09 | 4.59 | 4.17 | 5.18E-16 | 7.41 | 1.26E-05 | 877 |
| Cluster-40555.112156 | 0.36 | 0.5 | 9.32 | 5.04 | 0.06 | 0 | 6.48 | 7.88 | 4.17 | 1.72E-04 | 8.05 | 1.33E-14 | 1058 |
| Cluster-40555.191286 | 0.5 | 0.34 | 5.66 | 7.85 | 0 | 0.51 | 12.47 | 6.38 | 4.17 | 4.77E-10 | 5.22 | 5.67E-05 | 1328 |
| Cluster-40555.189914 | 2.15 | 1.7 | 33.24 | 29.93 | 2.03 | 3.4 | 32.45 | 33.48 | 4.17 | 5.84E-11 | 3.63 | 5.61E-10 | 524 |
| Cluster-40555.173367 | 0.38 | 0.41 | 6.17 | 6.58 | 0.1 | 0.25 | 4.83 | 6.47 | 4.17 | 2.40E-13 | 5.16 | 1.19E-13 | 1891 |
| Cluster-40555.230426 | 0.35 | 0 | 2.78 | 2.89 | 0 | 0 | 1.92 | 2.35 | 4.18 | 1.63E-10 | Inf | 6.36E-16 | 2941 |
| Cluster-40555.132498 | 1.08 | 1.02 | 10.78 | 23.14 | 13.11 | 10.36 | 24.67 | 30.02 | 4.18 | 1.04E-02 | 1.29 | 2.38E-02 | 662 |
| Cluster-40555.98837 | 0.06 | 0.12 | 1.7 | 1.29 | 0.19 | 0.19 | 2.88 | 1.74 | 4.18 | 3.52E-02 | 3.55 | 3.80E-03 | 1205 |
| Cluster-40555.224758 | 0.07 | 0.07 | 1.33 | 0.96 | 0.28 | 0.15 | 0.76 | 1.05 | 4.18 | 4.76E-06 | 2.13 | 2.42E-02 | 4039 |
| Cluster-40555.242130 | 0.1 | 0 | 0.83 | 0.89 | 0.17 | 0 | 1.06 | 1 | 4.19 | 1.40E-03 | 3.69 | 5.61E-04 | 2961 |
| Cluster-40555.164320 | 0.21 | 0.18 | 2.89 | 3.98 | 0.35 | 0 | 1.8 | 1.38 | 4.20 | 1.89E-07 | 3.30 | 5.43E-03 | 1746 |
| Cluster-40555.227474 | 0 | 0.14 | 1.38 | 0.86 | 0.44 | 0.32 | 2.37 | 2.05 | 4.20 | 5.98E-04 | 2.60 | 1.57E-04 | 2752 |
| Cluster-40555.194209 | 3.61 | 3.36 | 57.7 | 57.57 | 14.55 | 12.59 | 43.05 | 45.02 | 4.20 | 3.15E-29 | 1.77 | 1.22E-05 | 789 |
| Cluster-40555.201530 | 0.17 | 0.19 | 2.34 | 3.64 | 0 | 0.2 | 2.78 | 2.6 | 4.20 | 1.79E-05 | 4.75 | 2.40E-06 | 1475 |
| Cluster-40555.187125 | 0.42 | 0 | 3.23 | 3.27 | 0.15 | 0.22 | 1.94 | 1.9 | 4.21 | 7.61E-07 | 3.45 | 9.22E-04 | 1693 |
| Cluster-40555.87431 | 0.45 | 0.38 | 8.16 | 5.64 | 0.16 | 0 | 5.64 | 6.45 | 4.21 | 1.40E-07 | 6.22 | 5.82E-11 | 1064 |
| Cluster-40555.205951 | 0.49 | 0.67 | 9.88 | 9.67 | 0.17 | 0.18 | 16.45 | 18.51 | 4.21 | 1.61E-13 | 6.69 | 5.88E-33 | 1341 |
| Cluster-40555.95234 | 0 | 0.26 | 1.94 | 2.79 | 0.11 | 0 | 0.95 | 1.48 | 4.21 | 3.10E-03 | 4.62 | 4.71E-02 | 1140 |
| Cluster-40555.189911 | 4.47 | 5.47 | 91.6 | 77.26 | 24.14 | 21.38 | 51.98 | 41.84 | 4.21 | 3.60E-59 | 1.11 | 1.58E-03 | 2483 |
| Cluster-98945.0 | 0.31 | 0.08 | 3.01 | 2.89 | 0 | 0 | 2.63 | 1.04 | 4.22 | 1.96E-02 | Inf | 2.42E-02 | 779 |
| Cluster-40555.145238 | 0.09 | 0.3 | 3.03 | 3.75 | 0.09 | 0.19 | 1.93 | 4.19 | 4.22 | 8.26E-03 | 4.52 | 3.22E-02 | 790 |
| Cluster-40555.203079 | 0.24 | 0.13 | 3.25 | 3.83 | 0.18 | 0 | 3.72 | 3.49 | 4.22 | 5.82E-03 | 5.45 | 3.96E-04 | 791 |

| | | | | | | | | | | | | | |
|---|---|---|---|---|---|---|---|---|---|---|---|---|---|
| Cluster-40555.165027 | 0.26 | 0 | 1.9 | 2.12 | 1.02 | 0.51 | 3.86 | 2.94 | 4.22 | 4.07E-03 | 2.24 | 1.93E-02 | 1233 |
| Cluster-40555.180844 | 0.61 | 0.59 | 9.78 | 10.78 | 3.68 | 2.31 | 13.15 | 8.05 | 4.22 | 2.19E-14 | 1.89 | 1.10E-02 | 1345 |
| Cluster-40555.198111 | 0.6 | 1.82 | 19.19 | 22.22 | 5.71 | 2.33 | 13.01 | 14.3 | 4.22 | 1.72E-16 | 1.86 | 1.24E-03 | 906 |
| Cluster-40555.185154 | 10.28 | 13.53 | 213.04 | 194.14 | 13.42 | 15.93 | 76.44 | 64.43 | 4.23 | 3.26E-64 | 2.32 | 9.59E-14 | 1677 |
| Cluster-40555.183212 | 4 | 4.64 | 79.48 | 64.93 | 16.97 | 18.29 | 96.45 | 75.18 | 4.23 | 8.53E-07 | 2.31 | 1.58E-03 | 322 |
| Cluster-40555.268199 | 0.22 | 0.11 | 2.72 | 2.56 | 0.18 | 0.16 | 1.53 | 1.59 | 4.23 | 1.45E-04 | 3.31 | 1.78E-02 | 1417 |
| Cluster-40555.214768 | 0.55 | 0.15 | 6.71 | 4.97 | 2.55 | 2.48 | 10.28 | 13.9 | 4.23 | 2.00E-02 | 2.33 | 1.72E-02 | 527 |
| Cluster-40555.225595 | 1.04 | 0.7 | 15.22 | 13.35 | 0 | 0.59 | 4.56 | 5.98 | 4.24 | 2.65E-10 | 4.24 | 7.94E-05 | 790 |
| Cluster-40555.214875 | 0.17 | 0.14 | 2.65 | 2.78 | 0.51 | 0.36 | 5.03 | 6.61 | 4.25 | 2.02E-02 | 3.83 | 8.24E-05 | 819 |
| Cluster-40555.185158 | 0.12 | 0 | 1.29 | 0.55 | 0.11 | 0 | 1.86 | 2.64 | 4.25 | 3.07E-02 | 5.53 | 2.06E-08 | 2023 |
| Cluster-40555.236004 | 0.12 | 0 | 1.11 | 1.2 | 0.03 | 0 | 1.21 | 2.03 | 4.26 | 1.30E-02 | 6.72 | 4.10E-05 | 1657 |
| Cluster-40555.161802 | 1.36 | 0.97 | 23.56 | 17.39 | 1.32 | 0.94 | 12.62 | 14.13 | 4.26 | 9.78E-14 | 3.62 | 1.62E-08 | 787 |
| Cluster-40555.188270 | 0.66 | 0.85 | 12.94 | 13.84 | 0.51 | 0.89 | 9.12 | 5.9 | 4.26 | 4.96E-15 | 3.43 | 4.20E-07 | 1135 |
| Cluster-40555.246749 | 0.04 | 0.24 | 2.36 | 2.33 | 0.07 | 0 | 1.14 | 1.22 | 4.27 | 2.22E-05 | 5.42 | 5.27E-04 | 1827 |
| Cluster-40555.198842 | 0.67 | 2.74 | 29.48 | 30.7 | 2.72 | 1.33 | 13.14 | 15.19 | 4.27 | 5.40E-15 | 2.93 | 3.01E-05 | 654 |
| Cluster-40555.126902 | 0.34 | 0.71 | 10.07 | 8.67 | 0 | 0 | 0.62 | 0.67 | 4.27 | 1.22E-19 | Inf | 3.66E-03 | 2070 |
| Cluster-40555.194865 | 0.75 | 0.5 | 10.37 | 11.35 | 5.46 | 4.93 | 16.65 | 17.28 | 4.27 | 7.18E-18 | 1.78 | 1.14E-05 | 1581 |
| Cluster-40555.167779 | 0.41 | 0.15 | 5.06 | 4.43 | 1 | 0.47 | 4.05 | 3.6 | 4.27 | 5.57E-14 | 2.45 | 1.21E-05 | 2506 |
| Cluster-40555.45148 | 0.12 | 0.4 | 4.49 | 4.84 | 0 | 0 | 6.8 | 2.84 | 4.27 | 6.35E-03 | Inf | 3.11E-03 | 660 |
| Cluster-40555.135541 | 0.27 | 0.2 | 4.74 | 3.93 | 0.28 | 0.7 | 4.17 | 5.27 | 4.27 | 3.35E-09 | 3.31 | 5.66E-08 | 1789 |
| Cluster-40555.185292 | 0 | 0.18 | 1.74 | 1.55 | 0.17 | 0.13 | 1.48 | 1.28 | 4.28 | 2.50E-04 | 3.30 | 3.10E-03 | 2050 |
| Cluster-40555.166265 | 0.93 | 3.07 | 33.76 | 37.98 | 4.01 | 5.98 | 41.84 | 42.88 | 4.28 | 5.27E-23 | 3.13 | 3.62E-15 | 834 |
| Cluster-40555.185321 | 0.31 | 0.25 | 3.66 | 5.96 | 0 | 0.31 | 3.71 | 3.36 | 4.28 | 1.50E-02 | 4.50 | 3.64E-02 | 581 |
| Cluster-40555.237917 | 0.09 | 0.19 | 2.69 | 2.62 | 2.59 | 1.02 | 5.77 | 5.37 | 4.28 | 3.96E-07 | 1.71 | 1.49E-03 | 2122 |
| Cluster-40555.170726 | 0.16 | 0.19 | 3.42 | 2.91 | 2.08 | 1.38 | 5.37 | 4.94 | 4.28 | 1.13E-06 | 1.64 | 8.37E-03 | 1730 |
| Cluster-40555.184953 | 0 | 0.34 | 2.09 | 4.25 | 0 | 1.63 | 6.18 | 4.86 | 4.28 | 6.54E-03 | 2.78 | 7.38E-06 | 1411 |
| Cluster-40555.167204 | 0.24 | 0.2 | 3.23 | 4.51 | 0.12 | 0.06 | 1.64 | 3.38 | 4.28 | 7.91E-05 | 4.91 | 1.16E-02 | 1063 |
| Cluster-40555.188403 | 1.57 | 2.21 | 35.3 | 32.21 | 6.88 | 7.54 | 22.31 | 22.15 | 4.29 | 1.48E-46 | 1.69 | 1.02E-06 | 2474 |
| Cluster-40555.226007 | 0.53 | 0.38 | 6.51 | 9.51 | 0.74 | 0.94 | 4.49 | 3.45 | 4.29 | 1.22E-07 | 2.30 | 4.32E-02 | 895 |
| Cluster-40555.185596 | 1.72 | 3.05 | 40.88 | 45.22 | 5.53 | 5.18 | 28.83 | 16.71 | 4.29 | 1.58E-35 | 2.15 | 1.01E-02 | 1205 |
| Cluster-40555.194148 | 0.08 | 0.06 | 1.28 | 1.27 | 0.04 | 0.03 | 1.13 | 1.44 | 4.29 | 7.52E-06 | 5.14 | 5.80E-08 | 3327 |
| Cluster-40555.209856 | 0 | 0.43 | 3.77 | 4.16 | 0.29 | 0.46 | 5.93 | 4.2 | 4.30 | 2.30E-02 | 3.98 | 4.44E-03 | 631 |

| | | | | | | | | | | | | | |
|---|---|---|---|---|---|---|---|---|---|---|---|---|---|
| Cluster-78539.0 | 0 | 0.14 | 1.11 | 1.45 | 0.06 | 0.09 | 1.22 | 1.18 | 4.30 | 6.16E-03 | 3.91 | 7.20E-03 | 1711 |
| Cluster-40555.204173 | 0.87 | 2.12 | 25.45 | 28.48 | 2.83 | 3.39 | 19.44 | 22.5 | 4.30 | 1.04E-13 | 2.80 | 1.43E-06 | 644 |
| Cluster-40555.202895 | 14.17 | 2.11 | 124.81 | 123.1 | 9.43 | 1.61 | 50.17 | 56.51 | 4.30 | 1.05E-06 | 3.32 | 4.26E-02 | 283 |
| Cluster-40555.252694 | 0.07 | 0.14 | 1.83 | 1.89 | 0.48 | 0.37 | 2.78 | 2.24 | 4.30 | 3.97E-04 | 2.63 | 2.51E-03 | 1713 |
| Cluster-40555.227623 | 0 | 0.79 | 8.32 | 6.08 | 0 | 0 | 3.73 | 3.22 | 4.30 | 2.67E-07 | Inf | 3.50E-07 | 990 |
| Cluster-40555.201336 | 5.4 | 1.35 | 62 | 56.3 | 18.64 | 18.37 | 100.66 | 88.15 | 4.31 | 1.17E-40 | 2.41 | 3.61E-14 | 1134 |
| Cluster-40555.184962 | 2.86 | 12.41 | 142.24 | 139.18 | 7.84 | 7.61 | 76.33 | 75.47 | 4.31 | 2.81E-50 | 3.36 | 6.15E-22 | 916 |
| Cluster-40555.217462 | 0.14 | 0.04 | 1.54 | 1.46 | 0.22 | 0.16 | 1.26 | 1.54 | 4.31 | 7.58E-04 | 2.86 | 1.55E-02 | 1903 |
| Cluster-40555.191861 | 10.24 | 16.72 | 228.35 | 259.39 | 25.92 | 20.72 | 189.64 | 170.95 | 4.31 | 3.50E-37 | 3.01 | 1.40E-15 | 440 |
| Cluster-40555.171756 | 0.19 | 0 | 1.11 | 2.16 | 0.36 | 0.4 | 2.54 | 2.27 | 4.31 | 2.45E-03 | 2.73 | 5.29E-04 | 2025 |
| Cluster-40555.189527 | 6.35 | 7.18 | 113.76 | 129.75 | 4.57 | 6.23 | 24.11 | 34.2 | 4.31 | 1.29E-49 | 2.50 | 1.41E-04 | 2864 |
| Cluster-40555.190861 | 1.37 | 1.07 | 19.87 | 23.38 | 6.42 | 4.21 | 18.19 | 18.27 | 4.31 | 2.41E-14 | 1.85 | 1.04E-03 | 755 |
| Cluster-40555.183410 | 0.75 | 0.5 | 11.54 | 11.03 | 3.78 | 4.17 | 13.36 | 10.24 | 4.31 | 4.73E-11 | 1.63 | 5.52E-03 | 966 |
| Cluster-40555.187631 | 10.12 | 20.21 | 294.85 | 258.74 | 96.57 | 126.66 | 240.95 | 225.68 | 4.31 | 8.80E-64 | 1.12 | 7.24E-04 | 1124 |
| Cluster-40555.237568 | 0.53 | 0.29 | 6.94 | 7.67 | 0.68 | 0.23 | 4.19 | 4.16 | 4.31 | 1.14E-12 | 3.30 | 5.17E-06 | 1522 |
| Cluster-40555.186133 | 6.41 | 7.83 | 138.39 | 118.68 | 42.88 | 46.49 | 123.06 | 138.35 | 4.31 | 9.78E-29 | 1.61 | 3.51E-05 | 484 |
| Cluster-40555.188288 | 824.86 | 1121.88 | 16668.18 | 18646.8 | 3266.08 | 2976.66 | 16332.2 | 16252.29 | 4.31 | 1.03E-62 | 2.45 | 1.24E-17 | 1442 |
| Cluster-40555.201456 | 6.05 | 6.25 | 110.36 | 113.15 | 17.58 | 24.19 | 74.26 | 85.27 | 4.32 | 1.32E-40 | 1.99 | 2.54E-08 | 725 |
| Cluster-40555.161789 | 0.13 | 0.05 | 1.76 | 1.45 | 0.4 | 0.35 | 4.72 | 4.29 | 4.32 | 2.90E-08 | 3.66 | 2.12E-15 | 3825 |
| Cluster-40555.176008 | 0.53 | 0.61 | 9.75 | 11.09 | 1.25 | 2.3 | 8.21 | 6.64 | 4.32 | 4.64E-07 | 2.11 | 2.39E-02 | 716 |
| Cluster-40555.189214 | 1 | 1.36 | 21.76 | 21.49 | 0 | 0.68 | 27.48 | 31.59 | 4.32 | 1.75E-40 | 6.48 | 8.62E-60 | 2669 |
| Cluster-40555.204712 | 0.08 | 0.06 | 1.4 | 1.2 | 0.47 | 0.38 | 1.45 | 1.91 | 4.33 | 7.18E-05 | 2.07 | 1.26E-02 | 2738 |
| Cluster-40555.173005 | 0.16 | 0.28 | 4.18 | 4.16 | 0.97 | 0.86 | 6.9 | 5.41 | 4.33 | 1.60E-08 | 2.80 | 4.10E-07 | 1685 |
| Cluster-40555.203763 | 0.29 | 0.63 | 8.21 | 8.85 | 1.4 | 1.54 | 9.24 | 8.82 | 4.33 | 2.00E-06 | 2.66 | 5.03E-04 | 767 |
| Cluster-40555.231210 | 0.21 | 0.05 | 3.21 | 1.55 | 0.21 | 0.17 | 1.43 | 2.17 | 4.34 | 1.51E-03 | 3.38 | 1.05E-04 | 3239 |
| Cluster-40555.231105 | 0.1 | 0.18 | 2.46 | 2.88 | 0.34 | 0.21 | 2.55 | 2.59 | 4.34 | 4.62E-12 | 3.30 | 1.22E-08 | 3518 |
| Cluster-40555.178944 | 2.89 | 4.19 | 58.67 | 72.1 | 20.79 | 16.64 | 75.03 | 58.42 | 4.34 | 2.07E-24 | 1.90 | 2.38E-08 | 1045 |
| Cluster-40555.142761 | 0.23 | 0.19 | 4.06 | 3.54 | 0 | 0 | 1.79 | 2.41 | 4.34 | 2.88E-09 | Inf | 3.40E-10 | 1970 |
| Cluster-40555.202085 | 0.12 | 0.57 | 7.08 | 6.38 | 1.22 | 0.84 | 2.93 | 4.29 | 4.35 | 5.24E-13 | 1.89 | 1.29E-02 | 1715 |
| Cluster-40555.202337 | 0.38 | 0.48 | 7.97 | 8.08 | 0.47 | 0.49 | 5.05 | 4.82 | 4.35 | 2.90E-11 | 3.43 | 4.18E-06 | 1266 |
| Cluster-40555.186022 | 3.6 | 1.89 | 47.89 | 53.53 | 5.12 | 5.91 | 27.95 | 18.64 | 4.36 | 8.21E-24 | 2.13 | 1.41E-04 | 661 |
| Cluster-40555.148757 | 0 | 0.56 | 6.31 | 3.69 | 1.68 | 2.62 | 10.06 | 9.84 | 4.36 | 2.10E-02 | 2.27 | 2.19E-02 | 573 |

| | | | | | | | | | | | | | |
|---|---|---|---|---|---|---|---|---|---|---|---|---|---|
| Cluster-40555.85829 | 0.53 | 0.51 | 8.5 | 10.74 | 1.8 | 0.81 | 11.48 | 8.8 | 4.36 | 5.71E-08 | 3.03 | 1.41E-05 | 812 |
| Cluster-40555.112066 | 0.09 | 0.07 | 2.23 | 1.59 | 0.15 | 0.07 | 2.2 | 1.2 | 4.36 | 1.28E-02 | 4.04 | 1.61E-02 | 1132 |
| Cluster-40555.200352 | 0.72 | 0.53 | 11.34 | 11.68 | 0.11 | 0.21 | 12.35 | 9.33 | 4.37 | 1.39E-19 | 6.13 | 1.58E-26 | 1624 |
| Cluster-40555.202548 | 0 | 0.23 | 2.21 | 2.22 | 0.21 | 0.43 | 2.11 | 2.12 | 4.37 | 1.24E-03 | 2.77 | 2.06E-02 | 1324 |
| Cluster-40555.119978 | 0.6 | 0.74 | 13.5 | 11.56 | 4.16 | 2.76 | 12.67 | 14.18 | 4.37 | 2.51E-11 | 2.03 | 3.23E-04 | 913 |
| Cluster-40555.248985 | 0.32 | 2.96 | 31.17 | 33.12 | 0 | 0 | 5.74 | 4.43 | 4.37 | 4.78E-17 | Inf | 6.46E-06 | 678 |
| Cluster-40555.244389 | 0.14 | 0.04 | 1.04 | 2.13 | 0.35 | 0.37 | 2.32 | 3.46 | 4.37 | 7.09E-03 | 3.08 | 1.45E-03 | 1327 |
| Cluster-40555.154630 | 0.29 | 0.24 | 5.08 | 5.91 | 1.02 | 2.18 | 5.19 | 5.13 | 4.37 | 2.37E-06 | 1.75 | 4.75E-02 | 1014 |
| Cluster-40555.192164 | 0.99 | 0.07 | 10.55 | 8.6 | 1.55 | 0.76 | 15.38 | 15.17 | 4.38 | 1.43E-19 | 3.82 | 2.17E-20 | 1895 |
| Cluster-40555.187289 | 0.68 | 0.14 | 6.84 | 8 | 0.5 | 0.18 | 4.31 | 6.01 | 4.38 | 1.72E-03 | 4.02 | 1.96E-02 | 549 |
| Cluster-40555.172055 | 0.34 | 0.11 | 4.15 | 4.57 | 0 | 0.41 | 1.61 | 2.11 | 4.38 | 4.43E-11 | 3.18 | 4.57E-04 | 2020 |
| Cluster-40555.190945 | 4.59 | 2.86 | 67.52 | 71.91 | 11.64 | 9.67 | 51.2 | 49.91 | 4.38 | 8.74E-44 | 2.32 | 3.97E-11 | 1084 |
| Cluster-40555.195561 | 1.28 | 2.97 | 43.31 | 38 | 13.59 | 6.53 | 32.46 | 48.71 | 4.38 | 2.65E-19 | 2.09 | 9.31E-03 | 650 |
| Cluster-40555.157454 | 0.13 | 0 | 1.26 | 1.35 | 0.13 | 0.17 | 1.75 | 1.9 | 4.38 | 6.73E-05 | 3.73 | 4.36E-06 | 2611 |
| Cluster-40555.182015 | 0.45 | 0.72 | 9.82 | 12.51 | 2.67 | 1.63 | 11.2 | 12.07 | 4.38 | 6.44E-04 | 2.50 | 3.13E-02 | 480 |
| Cluster-40555.125082 | 0.22 | 0 | 1.95 | 1.72 | 0.03 | 0 | 1.99 | 1.69 | 4.39 | 3.20E-02 | Inf | 1.33E-03 | 973 |
| Cluster-40555.206755 | 0 | 0.2 | 1.96 | 2.44 | 0.51 | 1.14 | 9.02 | 9.13 | 4.39 | 1.39E-02 | 3.51 | 7.87E-08 | 971 |
| Cluster-40555.258564 | 0.13 | 0.42 | 7.12 | 3.68 | 10.12 | 6.38 | 27.11 | 29.73 | 4.40 | 4.18E-03 | 1.86 | 4.09E-04 | 638 |
| Cluster-40555.189538 | 1.46 | 2.32 | 33.88 | 40.51 | 7.93 | 10.46 | 23.22 | 31.44 | 4.40 | 2.20E-17 | 1.63 | 3.06E-03 | 617 |
| Cluster-40555.194575 | 0.52 | 0.34 | 8.06 | 8.43 | 0.67 | 1.27 | 8.7 | 6.56 | 4.40 | 5.76E-16 | 3.02 | 2.16E-09 | 1703 |
| Cluster-40555.196262 | 0 | 0.14 | 1.24 | 1.5 | 0.41 | 0.38 | 1.46 | 1.7 | 4.40 | 2.27E-04 | 2.06 | 3.62E-02 | 2305 |
| Cluster-40555.218125 | 0.08 | 0.11 | 2.12 | 1.38 | 0 | 0.41 | 1.78 | 3.06 | 4.40 | 7.95E-04 | 3.59 | 3.59E-03 | 1695 |
| Cluster-40555.189223 | 1.09 | 0.59 | 13.25 | 18.6 | 2.79 | 2.9 | 9.66 | 12.09 | 4.41 | 2.41E-10 | 1.99 | 1.34E-03 | 905 |
| Cluster-40555.230042 | 0.01 | 0.17 | 1.93 | 1.61 | 0.47 | 0 | 1.28 | 1.44 | 4.41 | 2.06E-05 | 2.63 | 1.48E-02 | 2309 |
| Cluster-40555.159610 | 6.96 | 16.1 | 260.12 | 191.18 | 6.97 | 0 | 50.37 | 61.72 | 4.41 | 2.78E-12 | 4.08 | 4.96E-03 | 289 |
| Cluster-40555.269621 | 0.18 | 0 | 1.88 | 1.4 | 0.06 | 0 | 1.18 | 1.38 | 4.41 | 3.06E-02 | 5.61 | 2.29E-02 | 1081 |
| Cluster-40555.137873 | 0 | 0.15 | 1.74 | 1.32 | 0.33 | 0.12 | 1.38 | 1.55 | 4.42 | 7.44E-05 | 2.81 | 3.75E-03 | 2378 |
| Cluster-40555.219135 | 0 | 0.33 | 3.37 | 3.83 | 0.46 | 0.11 | 3.15 | 2.25 | 4.43 | 3.01E-03 | 3.50 | 3.39E-02 | 821 |
| Cluster-40555.235762 | 0 | 0.3 | 3.53 | 2.53 | 0 | 0.08 | 2.26 | 1.81 | 4.43 | 2.58E-04 | 5.30 | 3.40E-04 | 1203 |
| Cluster-40555.191547 | 5.29 | 15.38 | 197.57 | 214.91 | 120.93 | 73.09 | 243.72 | 177.86 | 4.43 | 3.50E-25 | 1.18 | 4.12E-02 | 366 |
| Cluster-40555.227451 | 0.08 | 0.43 | 4.39 | 5.68 | 0.26 | 0.26 | 2.47 | 2.17 | 4.43 | 2.28E-15 | 3.22 | 3.63E-06 | 2640 |
| Cluster-40555.227499 | 1.09 | 0.81 | 23.68 | 14.22 | 1.01 | 0.49 | 10.91 | 9.93 | 4.43 | 3.37E-07 | 3.86 | 4.17E-08 | 833 |

| | | | | | | | | | | | | | |
|---|---|---|---|---|---|---|---|---|---|---|---|---|---|
| Cluster-40555.133489 | 0.21 | 0.91 | 9.08 | 11.87 | 0 | 0 | 8.79 | 9.01 | 4.43 | 5.27E-04 | Inf | 1.04E-05 | 498 |
| Cluster-40555.182617 | 1.32 | 0.87 | 24.17 | 18.39 | 10.13 | 4.78 | 36.55 | 29.02 | 4.44 | 8.60E-24 | 2.22 | 2.67E-09 | 1225 |
| Cluster-40555.194977 | 0.47 | 0.5 | 9.58 | 9.37 | 1.82 | 1.17 | 5.91 | 5.94 | 4.44 | 5.14E-20 | 2.07 | 9.25E-05 | 1939 |
| Cluster-40555.218296 | 0.32 | 0 | 3.75 | 2.4 | 0 | 0 | 2.66 | 1.63 | 4.44 | 9.43E-09 | Inf | 1.77E-11 | 2345 |
| Cluster-40555.155625 | 0.61 | 0.94 | 14.15 | 16.67 | 3.25 | 0.81 | 17.7 | 24.78 | 4.44 | 3.23E-12 | 3.22 | 4.84E-09 | 814 |
| Cluster-40555.205613 | 0.19 | 0.31 | 4.81 | 5.19 | 0.84 | 0.89 | 5.38 | 3.49 | 4.44 | 5.98E-06 | 2.38 | 9.18E-03 | 1026 |
| Cluster-40555.195024 | 1.32 | 1.01 | 24.1 | 21.64 | 8.17 | 7.06 | 19.07 | 19.51 | 4.44 | 2.41E-36 | 1.41 | 2.55E-04 | 1978 |
| Cluster-40555.190972 | 0.8 | 1.95 | 31.46 | 23.71 | 9 | 5.8 | 42.45 | 35.92 | 4.45 | 1.97E-24 | 2.48 | 3.11E-11 | 1047 |
| Cluster-40555.208469 | 3.01 | 0.65 | 28.62 | 40.69 | 4.36 | 4.74 | 35.65 | 18.51 | 4.45 | 6.04E-10 | 2.62 | 3.17E-02 | 506 |
| Cluster-40555.284069 | 0.18 | 0.38 | 5.06 | 6.26 | 0 | 0 | 4.36 | 2.2 | 4.45 | 9.49E-05 | Inf | 9.95E-05 | 787 |
| Cluster-40555.191546 | 3.19 | 5.24 | 78.82 | 90.82 | 1.43 | 1.43 | 61.75 | 44.91 | 4.45 | 5.32E-41 | 5.31 | 1.27E-24 | 832 |
| Cluster-40555.118889 | 0.18 | 0 | 1.66 | 1.61 | 0 | 0.25 | 0.92 | 1.45 | 4.45 | 8.88E-04 | 3.32 | 2.08E-02 | 1675 |
| Cluster-40555.173578 | 0 | 0.25 | 2.24 | 2.94 | 0.71 | 0.43 | 4.37 | 2.88 | 4.45 | 2.31E-03 | 2.74 | 6.77E-03 | 1059 |
| Cluster-40555.173214 | 0.65 | 1.73 | 22.45 | 25.08 | 3.81 | 1.79 | 10.98 | 12.85 | 4.46 | 4.16E-19 | 2.17 | 3.42E-04 | 886 |
| Cluster-40555.191236 | 2.95 | 7.05 | 90.03 | 111.96 | 27.06 | 20.27 | 87.97 | 98.35 | 4.46 | 7.69E-23 | 2.05 | 1.24E-09 | 868 |
| Cluster-40555.197128 | 0.38 | 0.88 | 13.31 | 12.18 | 0 | 0.78 | 14.82 | 6.47 | 4.46 | 8.88E-03 | 4.75 | 2.94E-02 | 394 |
| Cluster-40555.188102 | 1.97 | 2.24 | 43.62 | 40.81 | 4.73 | 5.84 | 33.73 | 38.72 | 4.46 | 8.71E-35 | 2.85 | 2.14E-14 | 1118 |
| Cluster-40555.178366 | 0.36 | 0.07 | 5.12 | 3.3 | 0.79 | 0.18 | 3.58 | 3.5 | 4.46 | 1.29E-03 | 2.96 | 3.34E-02 | 803 |
| Cluster-40555.195462 | 0 | 0.58 | 3.99 | 8.13 | 1.45 | 1.71 | 13.75 | 10.47 | 4.46 | 7.42E-03 | 2.99 | 2.44E-04 | 603 |
| Cluster-40555.106252 | 0.28 | 0.21 | 5.23 | 4.64 | 0.44 | 0.59 | 3.01 | 3.29 | 4.46 | 1.72E-05 | 2.59 | 3.29E-02 | 976 |
| Cluster-40555.163598 | 0.42 | 0.15 | 5.97 | 5.25 | 0.06 | 0 | 2.76 | 3.63 | 4.46 | 6.13E-07 | 6.90 | 1.84E-06 | 1066 |
| Cluster-40555.184023 | 0.11 | 0.69 | 7.64 | 8.82 | 1.71 | 0.74 | 5.63 | 6.31 | 4.47 | 1.44E-11 | 2.35 | 5.90E-04 | 1236 |
| Cluster-40555.180661 | 2.21 | 1.48 | 39.95 | 34.23 | 2.5 | 3.06 | 24.6 | 24.53 | 4.47 | 7.45E-48 | 3.20 | 6.33E-20 | 2102 |
| Cluster-40555.199373 | 1.01 | 2.04 | 30.94 | 24.29 | 4.82 | 1.17 | 35.1 | 39.24 | 4.48 | 2.04E-03 | 3.65 | 1.26E-03 | 335 |
| Cluster-40555.131910 | 0.11 | 0.4 | 5.64 | 4.92 | 0.05 | 0.06 | 1.36 | 2.09 | 4.48 | 4.07E-07 | 5.08 | 3.04E-03 | 1157 |
| Cluster-40555.193770 | 1.01 | 0.55 | 17.38 | 12.8 | 0.2 | 0 | 2.49 | 3.76 | 4.48 | 1.13E-12 | 4.84 | 1.19E-03 | 859 |
| Cluster-40555.216489 | 0.35 | 0 | 3.64 | 3.14 | 0.14 | 0.21 | 2.08 | 2.25 | 4.48 | 2.47E-07 | 3.69 | 2.13E-04 | 1653 |
| Cluster-40555.194228 | 0.1 | 0.13 | 2.37 | 2.21 | 0.1 | 0.1 | 0.99 | 2.07 | 4.48 | 1.40E-03 | 4.04 | 4.44E-02 | 1231 |
| Cluster-40555.185791 | 1.01 | 3.09 | 41.83 | 43.66 | 1.7 | 1.04 | 40.19 | 28.86 | 4.49 | 1.11E-22 | 4.71 | 3.92E-18 | 697 |
| Cluster-40555.209356 | 0.26 | 0 | 2.4 | 2.6 | 0.7 | 0.35 | 2.7 | 2.43 | 4.49 | 9.00E-07 | 2.36 | 2.98E-03 | 1965 |
| Cluster-40555.202939 | 2.56 | 2.58 | 52.84 | 52.8 | 34.03 | 32.34 | 73.3 | 77.74 | 4.49 | 2.72E-26 | 1.25 | 1.80E-03 | 691 |
| Cluster-40555.188867 | 29.12 | 42.95 | 731.45 | 747.09 | 77.04 | 55.84 | 339.84 | 390.12 | 4.49 | 1.62E-76 | 2.53 | 1.85E-17 | 1056 |

| | | | | | | | | | | | | | |
|---|---|---|---|---|---|---|---|---|---|---|---|---|---|
| Cluster-40555.165592 | 0.25 | 0.25 | 4.82 | 4.99 | 0.15 | 0.15 | 3.65 | 2.84 | 4.49 | 8.26E-13 | 4.56 | 5.30E-10 | 2086 |
| Cluster-40555.170395 | 0.09 | 2.17 | 27.48 | 20.58 | 28.33 | 12.91 | 49.42 | 47.54 | 4.50 | 4.71E-17 | 1.31 | 1.87E-03 | 815 |
| Cluster-40555.209833 | 0 | 0.14 | 1.11 | 1.96 | 0.62 | 0.48 | 3.12 | 3.39 | 4.50 | 3.17E-02 | 2.64 | 1.33E-02 | 1091 |
| Cluster-40555.196895 | 2.3 | 2.65 | 50.56 | 51.71 | 4.79 | 2.76 | 55.13 | 54.18 | 4.50 | 1.89E-33 | 3.93 | 1.69E-24 | 902 |
| Cluster-40555.208438 | 0.12 | 0.17 | 3.13 | 3.09 | 1 | 0.54 | 3.26 | 3.21 | 4.50 | 1.55E-07 | 2.16 | 3.20E-03 | 1846 |
| Cluster-40555.180616 | 1.66 | 4.53 | 68.92 | 60.56 | 9.05 | 6.05 | 29.41 | 33.67 | 4.50 | 5.58E-34 | 2.14 | 1.05E-06 | 798 |
| Cluster-40555.189171 | 1.48 | 4.25 | 65.92 | 54.83 | 0 | 0.37 | 90.4 | 72.61 | 4.50 | 1.01E-40 | 8.81 | 1.43E-72 | 1071 |
| Cluster-40555.181635 | 0.55 | 0.38 | 9.4 | 9.6 | 1.18 | 0.63 | 9.45 | 9.49 | 4.51 | 1.48E-12 | 3.45 | 2.49E-09 | 1173 |
| Cluster-40555.171424 | 0.46 | 0.21 | 6.66 | 6.84 | 0.14 | 0.14 | 1.88 | 1.55 | 4.51 | 2.26E-13 | 3.68 | 1.64E-03 | 1632 |
| Cluster-40555.167670 | 0 | 0.32 | 3.58 | 2.87 | 0.48 | 0.49 | 6.24 | 8.2 | 4.52 | 4.51E-04 | 3.99 | 2.11E-08 | 1069 |
| Cluster-40555.220427 | 0.36 | 0.25 | 7 | 5.58 | 0.25 | 0.58 | 7.49 | 5.9 | 4.52 | 4.03E-14 | 4.04 | 7.73E-14 | 1870 |
| Cluster-40555.233808 | 0.14 | 0.12 | 2.63 | 2.63 | 1.93 | 2.21 | 7.3 | 11.32 | 4.52 | 4.62E-03 | 2.25 | 7.26E-03 | 955 |
| Cluster-40555.201054 | 0.84 | 0.53 | 13.85 | 13.95 | 1.31 | 1.11 | 5.78 | 4.43 | 4.52 | 8.06E-13 | 2.13 | 2.96E-02 | 895 |
| Cluster-40555.193269 | 0.67 | 0.18 | 9.47 | 8.02 | 3.79 | 1.7 | 11.35 | 12.84 | 4.53 | 1.58E-25 | 2.23 | 4.37E-09 | 2704 |
| Cluster-40555.159567 | 0.06 | 0 | 0.67 | 0.43 | 0 | 0 | 0.77 | 0.65 | 4.54 | 1.84E-02 | Inf | 1.79E-05 | 2977 |
| Cluster-40555.173070 | 0 | 0.26 | 2.48 | 3.04 | 0.43 | 0.92 | 2.4 | 2.85 | 4.54 | 7.72E-07 | 2.01 | 1.33E-02 | 1843 |
| Cluster-40555.194527 | 0 | 0.6 | 6.64 | 6.21 | 2.1 | 2.18 | 8.67 | 12.25 | 4.54 | 3.89E-09 | 2.37 | 2.20E-04 | 1221 |
| Cluster-40555.163649 | 0.38 | 0 | 3.88 | 4.01 | 0.22 | 0 | 1.9 | 1.1 | 4.54 | 4.45E-07 | 3.96 | 9.74E-03 | 1377 |
| Cluster-40555.216031 | 0.45 | 0.21 | 5.59 | 6.44 | 0.4 | 1.37 | 6.01 | 4.74 | 4.54 | 1.12E-04 | 2.62 | 1.71E-02 | 731 |
| Cluster-40555.186695 | 0 | 0.15 | 2.03 | 1.48 | 0 | 0 | 1.73 | 1.07 | 4.54 | 3.44E-02 | Inf | 7.58E-03 | 1016 |
| Cluster-40555.117261 | 0.33 | 0.53 | 10.1 | 7.29 | 0 | 0.16 | 6.59 | 7.45 | 4.54 | 2.36E-04 | 6.47 | 2.47E-05 | 576 |
| Cluster-40555.207877 | 0.9 | 1.09 | 23.42 | 18.95 | 0.97 | 0.97 | 10.17 | 12.36 | 4.54 | 2.91E-17 | 3.58 | 2.54E-08 | 871 |
| Cluster-40555.151171 | 8.09 | 0.84 | 75.48 | 105.18 | 0.43 | 18.63 | 60.06 | 44 | 4.55 | 7.12E-12 | 2.46 | 6.06E-04 | 593 |
| Cluster-40555.184334 | 0.65 | 2.6 | 35.87 | 35.19 | 8.33 | 9.16 | 37.04 | 39.01 | 4.55 | 8.77E-12 | 2.17 | 1.15E-04 | 489 |
| Cluster-40555.207038 | 0.29 | 1.14 | 14.26 | 17.03 | 0 | 0 | 4.69 | 7.18 | 4.55 | 2.05E-04 | Inf | 1.41E-02 | 433 |
| Cluster-40555.195219 | 1.87 | 2.24 | 43.59 | 44.14 | 5.61 | 4.66 | 22.34 | 26.69 | 4.55 | 1.08E-59 | 2.33 | 1.75E-12 | 2929 |
| Cluster-40555.187657 | 6.85 | 8.16 | 151.68 | 169.29 | 50.32 | 34.8 | 140.75 | 158.41 | 4.55 | 7.11E-66 | 1.89 | 1.18E-09 | 1299 |
| Cluster-40555.188039 | 14.32 | 20.97 | 386.56 | 372.32 | 97.88 | 108.54 | 272.38 | 254.75 | 4.56 | 4.10E-73 | 1.41 | 6.63E-06 | 1091 |
| Cluster-40555.169190 | 0 | 0.4 | 5.25 | 3.88 | 0 | 0 | 4.4 | 2.35 | 4.56 | 3.97E-06 | Inf | 2.57E-06 | 1138 |
| Cluster-40555.179687 | 0.15 | 0.28 | 5.48 | 4.23 | 3.73 | 6.28 | 19.82 | 15.7 | 4.56 | 1.88E-06 | 1.87 | 2.52E-05 | 1122 |
| Cluster-40555.178476 | 0.63 | 0.26 | 9.62 | 9.11 | 0.77 | 0.16 | 11.34 | 9.14 | 4.56 | 6.59E-09 | 4.56 | 2.77E-10 | 876 |
| Cluster-40555.166884 | 0.09 | 0.14 | 3.13 | 2.32 | 0.39 | 0.34 | 3.12 | 1.77 | 4.56 | 1.92E-04 | 2.77 | 1.27E-02 | 1286 |

| | | | | | | | | | | | | | |
|---|---|---|---|---|---|---|---|---|---|---|---|---|---|
| Cluster-40555.149946 | 0.64 | 0.42 | 8.94 | 12.36 | 2.94 | 3.89 | 15.12 | 17.69 | 4.57 | 5.93E-07 | 2.33 | 2.21E-04 | 664 |
| Cluster-40555.188839 | 0.61 | 1.18 | 18.56 | 20.5 | 0.79 | 0.49 | 11.79 | 11.85 | 4.57 | 1.03E-18 | 4.28 | 3.98E-12 | 972 |
| Cluster-40555.147543 | 0.15 | 0 | 1.26 | 1.8 | 0 | 0 | 1.12 | 2.2 | 4.57 | 1.20E-02 | Inf | 1.17E-03 | 1227 |
| Cluster-40555.189251 | 3.06 | 2.64 | 56.89 | 65.63 | 2.64 | 2.56 | 15.01 | 19.03 | 4.57 | 4.14E-47 | 2.78 | 5.88E-10 | 3318 |
| Cluster-40555.200651 | 0.19 | 1.99 | 22.11 | 25.73 | 4.4 | 4.6 | 20.36 | 21.75 | 4.57 | 5.71E-12 | 2.29 | 1.75E-04 | 601 |
| Cluster-40555.191677 | 4.61 | 0 | 60.75 | 29.81 | 0 | 0.54 | 22.43 | 37.06 | 4.57 | 1.10E-03 | 6.77 | 1.10E-06 | 359 |
| Cluster-40555.184701 | 5.17 | 4.81 | 102.84 | 108.07 | 24.22 | 13.51 | 121.7 | 115.29 | 4.57 | 1.44E-12 | 2.70 | 2.01E-06 | 338 |
| Cluster-40555.176960 | 0.23 | 0.19 | 4.36 | 4.47 | 0 | 0.11 | 2.1 | 2.24 | 4.58 | 4.05E-06 | 5.27 | 4.48E-04 | 1123 |
| Cluster-40555.177792 | 1.24 | 2.87 | 47.71 | 42.6 | 28.1 | 23.55 | 59.18 | 62.24 | 4.58 | 3.79E-22 | 1.30 | 2.46E-03 | 652 |
| Cluster-40555.179634 | 0.83 | 1.23 | 23.49 | 22.08 | 0.42 | 0.24 | 8.06 | 8.85 | 4.58 | 2.22E-27 | 4.79 | 3.09E-14 | 1293 |
| Cluster-40555.177351 | 0 | 0.36 | 3.85 | 3.91 | 0.3 | 0.29 | 2.33 | 1.74 | 4.58 | 1.17E-07 | 2.82 | 8.60E-03 | 1537 |
| Cluster-40555.167125 | 0.36 | 0.49 | 10.65 | 8.04 | 4.59 | 4.94 | 17.09 | 12.49 | 4.58 | 6.23E-06 | 1.69 | 1.50E-02 | 666 |
| Cluster-40555.145921 | 0 | 0.38 | 4.78 | 3.79 | 0.31 | 0 | 2.4 | 1.56 | 4.58 | 2.70E-06 | 3.86 | 5.83E-03 | 1213 |
| Cluster-40555.203930 | 0.71 | 0.48 | 7.61 | 18.19 | 1.66 | 0.92 | 11.52 | 14.46 | 4.58 | 1.76E-02 | 3.41 | 6.77E-10 | 1001 |
| Cluster-40555.270503 | 0.35 | 0.19 | 5.92 | 5.73 | 0.23 | 1.8 | 11.09 | 10.61 | 4.59 | 5.05E-04 | 3.44 | 9.49E-06 | 677 |
| Cluster-40555.293196 | 0 | 0.11 | 0.97 | 1.24 | 0.31 | 0.1 | 1.89 | 1.21 | 4.59 | 4.61E-03 | 3.02 | 6.81E-03 | 1901 |
| Cluster-40555.200445 | 0.11 | 0.11 | 1.39 | 3.07 | 0.12 | 0.24 | 2.84 | 2.02 | 4.59 | 1.01E-02 | 3.88 | 7.49E-05 | 1547 |
| Cluster-40555.141334 | 0.22 | 0.44 | 7.14 | 7.52 | 0.95 | 1.56 | 6.72 | 6.85 | 4.59 | 2.48E-05 | 2.49 | 1.12E-02 | 710 |
| Cluster-40555.189301 | 2.16 | 1.08 | 32.98 | 37.13 | 0.96 | 0.43 | 8.02 | 11.9 | 4.59 | 9.68E-49 | 3.91 | 2.28E-06 | 2041 |
| Cluster-40555.192542 | 7.62 | 8.9 | 168.66 | 193.2 | 66.94 | 69.89 | 144.72 | 143.88 | 4.59 | 1.09E-50 | 1.14 | 1.27E-03 | 859 |
| Cluster-40555.189141 | 5.18 | 5.04 | 101.87 | 121.26 | 4.03 | 1.63 | 78.9 | 69.24 | 4.59 | 1.18E-34 | 4.78 | 1.57E-35 | 888 |
| Cluster-40555.174898 | 0.19 | 0.22 | 4.18 | 4.79 | 0.16 | 0.11 | 1.21 | 1.76 | 4.60 | 9.13E-13 | 3.66 | 4.19E-04 | 2172 |
| Cluster-40555.189125 | 10.02 | 11.12 | 240.16 | 223.83 | 57.1 | 58.58 | 143 | 163.27 | 4.60 | 6.24E-60 | 1.47 | 1.33E-05 | 757 |
| Cluster-40555.175557 | 0.28 | 0.49 | 8.06 | 9.06 | 0.43 | 1.12 | 3.69 | 3.11 | 4.60 | 7.23E-11 | 2.16 | 3.68E-02 | 1095 |
| Cluster-40555.153381 | 0 | 0.07 | 0.81 | 0.8 | 0.18 | 0.14 | 1.07 | 0.99 | 4.60 | 4.67E-03 | 2.71 | 1.95E-02 | 2572 |
| Cluster-40555.195471 | 0.08 | 0.18 | 2.52 | 3.13 | 0.48 | 0.59 | 4.8 | 3.6 | 4.60 | 3.27E-03 | 3.01 | 3.32E-03 | 929 |
| Cluster-40555.145053 | 0.3 | 0.22 | 5.76 | 5.58 | 0.18 | 0.19 | 3.9 | 3.24 | 4.61 | 3.84E-11 | 4.33 | 1.11E-07 | 1556 |
| Cluster-40555.211001 | 0.73 | 1.2 | 20.71 | 22.43 | 1.44 | 1.51 | 13.46 | 17.09 | 4.61 | 6.21E-11 | 3.44 | 4.03E-06 | 594 |
| Cluster-40555.193454 | 0.08 | 0.05 | 1.27 | 1.42 | 0.06 | 0.04 | 0.87 | 0.6 | 4.61 | 1.15E-05 | 4.05 | 3.47E-03 | 2817 |
| Cluster-40555.220707 | 0.24 | 0.36 | 7.01 | 6.12 | 0 | 0 | 8.43 | 10.41 | 4.62 | 1.07E-04 | Inf | 1.38E-11 | 702 |
| Cluster-40555.100049 | 0 | 0.44 | 6.49 | 3.75 | 0.42 | 0.22 | 3.37 | 3.84 | 4.62 | 1.11E-03 | 3.58 | 2.07E-02 | 717 |
| Cluster-40555.195481 | 0.26 | 0.21 | 4.27 | 6.14 | 0.13 | 0 | 4.93 | 7.97 | 4.62 | 2.18E-03 | 6.78 | 6.51E-06 | 636 |

| | | | | | | | | | | | | | |
|---|---|---|---|---|---|---|---|---|---|---|---|---|---|
| Cluster-40555.172713 | 0.34 | 0.13 | 6.24 | 5.31 | 0.65 | 0.97 | 2.9 | 4.14 | 4.62 | 3.70E-07 | 2.20 | 4.15E-02 | 1041 |
| Cluster-40555.191381 | 30.95 | 23.75 | 666.88 | 555.28 | 33.59 | 42.7 | 934.86 | 793.75 | 4.63 | 1.59E-76 | 4.56 | 1.88E-49 | 878 |
| Cluster-40555.220746 | 0.12 | 0.22 | 3.51 | 4 | 0.48 | 1.96 | 20.7 | 15.17 | 4.63 | 8.01E-05 | 3.90 | 1.07E-15 | 1038 |
| Cluster-40555.161982 | 0.99 | 0.54 | 15.55 | 18.42 | 2.91 | 3.64 | 18.01 | 19.96 | 4.63 | 8.96E-15 | 2.59 | 2.33E-07 | 847 |
| Cluster-40555.235817 | 0.09 | 0.1 | 2.03 | 2.36 | 0.11 | 0.81 | 2.87 | 2.73 | 4.63 | 2.92E-09 | 2.62 | 5.19E-06 | 2933 |
| Cluster-40555.179537 | 1.66 | 2.14 | 42.91 | 43.1 | 7.05 | 5.54 | 56.25 | 54.15 | 4.63 | 2.36E-20 | 3.20 | 8.54E-14 | 614 |
| Cluster-40555.193805 | 1.52 | 2.27 | 41.6 | 43.56 | 24.3 | 24.46 | 58.7 | 43.72 | 4.64 | 8.52E-19 | 1.12 | 2.49E-02 | 582 |
| Cluster-40555.161504 | 0 | 0.09 | 0.91 | 1.4 | 0.15 | 0.33 | 1.27 | 1.94 | 4.64 | 3.17E-03 | 2.81 | 8.16E-03 | 1892 |
| Cluster-40555.235075 | 0.19 | 0 | 1.48 | 2.26 | 0 | 0.13 | 1.07 | 1.24 | 4.64 | 1.11E-03 | 4.11 | 2.86E-02 | 1370 |
| Cluster-40555.187746 | 33.97 | 51.25 | 830.67 | 1108.64 | 177.49 | 97.5 | 881.46 | 906.32 | 4.64 | 5.86E-16 | 2.78 | 1.39E-20 | 611 |
| Cluster-40555.164219 | 1.25 | 0 | 11.94 | 15.04 | 1.63 | 1.49 | 12.33 | 8.56 | 4.65 | 2.07E-09 | 2.77 | 3.56E-04 | 689 |
| Cluster-40555.191694 | 1.33 | 1.19 | 30.06 | 27.6 | 2.78 | 0.82 | 23.69 | 22.51 | 4.65 | 1.41E-38 | 3.76 | 5.71E-22 | 1630 |
| Cluster-40555.133090 | 0.45 | 0.62 | 11.24 | 13.15 | 0.45 | 0 | 9.44 | 9.46 | 4.65 | 1.86E-06 | 5.52 | 2.06E-06 | 583 |
| Cluster-40555.190389 | 3.57 | 2.34 | 69 | 65.03 | 2.5 | 2.05 | 63.37 | 57.01 | 4.65 | 4.33E-55 | 4.79 | 1.26E-41 | 1511 |
| Cluster-40555.170361 | 0 | 0.2 | 2.9 | 2.37 | 0.07 | 0.12 | 5.25 | 7.19 | 4.65 | 3.83E-03 | 6.00 | 4.06E-10 | 983 |
| Cluster-40555.150927 | 0.17 | 0.18 | 3.49 | 4.61 | 0.44 | 0.23 | 2.62 | 3.56 | 4.66 | 7.37E-09 | 3.30 | 5.50E-05 | 1614 |
| Cluster-40555.196019 | 3.27 | 1.89 | 58.83 | 57.91 | 0.96 | 1.59 | 67.54 | 61.27 | 4.66 | 3.52E-36 | 5.71 | 3.06E-41 | 853 |
| Cluster-40555.181608 | 0.27 | 0 | 3.46 | 2.65 | 0.32 | 0.36 | 2.54 | 1.91 | 4.66 | 3.64E-12 | 2.78 | 2.95E-05 | 2881 |
| Cluster-40555.197521 | 0.37 | 0.23 | 6.47 | 7.13 | 3.22 | 4.09 | 15.89 | 12.34 | 4.66 | 4.22E-11 | 2.01 | 7.83E-06 | 1309 |
| Cluster-40555.188508 | 3.09 | 2.23 | 59.24 | 62.49 | 3.25 | 3.29 | 20.16 | 29.64 | 4.66 | 4.96E-48 | 3.01 | 4.97E-05 | 1228 |
| Cluster-40555.273317 | 0.08 | 0.14 | 1.95 | 3 | 0.34 | 0.92 | 5.11 | 5.89 | 4.66 | 2.02E-03 | 3.14 | 5.41E-05 | 1057 |
| Cluster-40555.189597 | 1.34 | 2.3 | 40.72 | 43.01 | 1.01 | 0.22 | 30.8 | 28.43 | 4.67 | 1.48E-35 | 5.68 | 1.97E-32 | 1068 |
| Cluster-40555.191692 | 1.27 | 0.53 | 24.3 | 17.15 | 4.27 | 4.28 | 21.65 | 16.9 | 4.67 | 1.02E-17 | 2.23 | 4.58E-08 | 1315 |
| Cluster-40555.233057 | 0.56 | 0.78 | 15.78 | 16.06 | 1.1 | 1.46 | 17.74 | 16.6 | 4.68 | 1.40E-13 | 3.80 | 8.66E-12 | 832 |
| Cluster-40555.190212 | 0.59 | 0 | 7.74 | 5.41 | 0 | 0.11 | 1.17 | 2.9 | 4.68 | 5.30E-15 | 5.47 | 3.25E-02 | 1826 |
| Cluster-40555.195157 | 1.03 | 0.71 | 17.68 | 21.26 | 7.43 | 7.26 | 23.66 | 26.75 | 4.68 | 7.92E-18 | 1.84 | 3.80E-05 | 894 |
| Cluster-40555.197034 | 0.26 | 0.26 | 6.29 | 5.93 | 0.5 | 0.15 | 5.89 | 5.28 | 4.69 | 5.44E-15 | 4.20 | 8.13E-13 | 1936 |
| Cluster-40555.162912 | 0.11 | 0.29 | 4.53 | 4.83 | 0 | 0.61 | 4.38 | 3.96 | 4.70 | 1.53E-13 | 3.79 | 1.48E-10 | 2193 |
| Cluster-40555.184651 | 0.29 | 0 | 5.04 | 2.04 | 0.32 | 0.43 | 6.42 | 4.79 | 4.70 | 1.34E-02 | 3.95 | 2.22E-12 | 1982 |
| Cluster-40555.231740 | 0 | 0.58 | 7.23 | 7.18 | 0.24 | 0 | 10.25 | 10.61 | 4.70 | 1.52E-02 | 6.55 | 5.61E-05 | 465 |
| Cluster-40555.203027 | 1.4 | 1.71 | 35.98 | 38.5 | 13.85 | 8.63 | 44.97 | 41.71 | 4.70 | 3.49E-37 | 2.02 | 1.14E-08 | 1215 |
| Cluster-40555.221129 | 0.13 | 0.06 | 2.11 | 2.18 | 0.19 | 0.16 | 1.86 | 2.03 | 4.71 | 1.22E-08 | 3.57 | 2.51E-06 | 2739 |

| | | | | | | | | | | | | | |
|---|---|---|---|---|---|---|---|---|---|---|---|---|---|
| Cluster-40555.207937 | 0.09 | 0.21 | 3.78 | 3.67 | 0 | 0 | 1.45 | 1.93 | 4.71 | 7.28E-09 | Inf | 3.24E-07 | 1765 |
| Cluster-40555.168289 | 0.11 | 0 | 1.38 | 1.24 | 0 | 0.08 | 1.08 | 1.97 | 4.71 | 2.90E-07 | 5.42 | 2.66E-04 | 3625 |
| Cluster-40555.202284 | 0.1 | 0.23 | 5.34 | 2.44 | 0 | 0.14 | 3.57 | 2.42 | 4.71 | 2.46E-03 | 5.47 | 7.93E-15 | 2767 |
| Cluster-40555.189620 | 1 | 1.87 | 35.13 | 33.68 | 20.7 | 19.98 | 38.71 | 41.29 | 4.71 | 7.29E-50 | 1.04 | 5.06E-03 | 2135 |
| Cluster-40555.167323 | 1.09 | 2.27 | 36.18 | 44.83 | 1.94 | 0.68 | 10.32 | 14.93 | 4.71 | 6.61E-14 | 3.35 | 1.64E-03 | 487 |
| Cluster-40555.196150 | 0.3 | 0.89 | 14.86 | 13.86 | 1.21 | 1.36 | 7.53 | 7.05 | 4.71 | 1.02E-33 | 2.56 | 6.23E-09 | 2419 |
| Cluster-40555.190915 | 1.29 | 1.08 | 24.16 | 32.07 | 0 | 0.17 | 7.62 | 12.05 | 4.71 | 3.33E-15 | 6.59 | 7.68E-08 | 744 |
| Cluster-40555.191215 | 0.79 | 2.33 | 37.99 | 37.8 | 0.13 | 0 | 36.39 | 30.2 | 4.71 | 3.96E-56 | 9.20 | 1.33E-77 | 2544 |
| Cluster-40555.216613 | 0 | 0.34 | 5.05 | 4.25 | 0 | 0 | 2.41 | 1.31 | 4.72 | 2.45E-03 | Inf | 4.08E-02 | 691 |
| Cluster-40555.185374 | 5.56 | 4.69 | 119.44 | 123.27 | 9.08 | 7.37 | 41.42 | 38.91 | 4.72 | 8.28E-41 | 2.35 | 5.01E-07 | 603 |
| Cluster-40555.197613 | 0.43 | 0.69 | 12.62 | 14.27 | 1.51 | 2.52 | 10.69 | 15.41 | 4.72 | 1.77E-18 | 2.76 | 9.63E-05 | 1224 |
| Cluster-40555.127650 | 0.17 | 0.02 | 2.29 | 2.36 | 0.03 | 0 | 0.94 | 0.76 | 4.72 | 1.94E-06 | 6.02 | 2.69E-03 | 1916 |
| Cluster-40555.163268 | 0.24 | 0.26 | 6.13 | 5.82 | 0.08 | 0 | 4.38 | 4.94 | 4.72 | 4.61E-06 | 7.04 | 3.22E-07 | 877 |
| Cluster-40555.177031 | 0.31 | 0 | 4.02 | 3.3 | 2.22 | 0.92 | 5.6 | 4.12 | 4.73 | 6.85E-13 | 1.71 | 1.04E-03 | 2550 |
| Cluster-40555.171519 | 0.37 | 0.17 | 5.65 | 6.59 | 1.83 | 1.24 | 7.14 | 6.99 | 4.73 | 1.63E-05 | 2.30 | 8.88E-03 | 789 |
| Cluster-40555.231092 | 0.36 | 0.27 | 7.8 | 7.55 | 2.13 | 2.28 | 7.15 | 5.44 | 4.74 | 8.37E-14 | 1.57 | 1.17E-02 | 1447 |
| Cluster-40555.165211 | 0.6 | 0.47 | 13.18 | 12.95 | 2.65 | 2.76 | 20.31 | 18.17 | 4.74 | 9.47E-18 | 2.89 | 1.36E-11 | 1203 |
| Cluster-40555.158492 | 0.25 | 0.31 | 6.49 | 7.34 | 0.75 | 0.28 | 7.63 | 8.61 | 4.74 | 1.55E-04 | 4.10 | 6.06E-05 | 643 |
| Cluster-40555.178959 | 2.12 | 2.22 | 57.78 | 47.94 | 22.45 | 13.61 | 48.5 | 48.93 | 4.75 | 1.69E-17 | 1.50 | 5.56E-03 | 493 |
| Cluster-40555.183823 | 0.34 | 0.64 | 9.93 | 13.99 | 0.17 | 0.17 | 7.53 | 8.45 | 4.75 | 1.02E-10 | 5.62 | 1.11E-09 | 832 |
| Cluster-40555.159369 | 0.94 | 0.61 | 19.37 | 18.64 | 6.28 | 2.35 | 17.14 | 17.73 | 4.75 | 2.12E-10 | 2.11 | 2.15E-03 | 605 |
| Cluster-40555.192124 | 1 | 1.21 | 28.17 | 25.84 | 3.84 | 3.24 | 10.82 | 14.56 | 4.76 | 6.48E-33 | 1.92 | 2.33E-04 | 1332 |
| Cluster-83299.2 | 0 | 0.32 | 2.93 | 5.11 | 0.13 | 0 | 2.47 | 2.89 | 4.76 | 9.30E-03 | 5.50 | 3.62E-02 | 640 |
| Cluster-40555.191426 | 4.72 | 3.6 | 111.02 | 92.09 | 10.37 | 2.3 | 106.97 | 139.56 | 4.77 | 7.51E-44 | 4.38 | 3.41E-15 | 729 |
| Cluster-40555.170104 | 0.35 | 0.71 | 12.01 | 14.67 | 0.74 | 0.52 | 9.76 | 10.79 | 4.77 | 1.64E-18 | 4.11 | 5.69E-13 | 1216 |
| Cluster-40555.188211 | 0.95 | 0.83 | 20.45 | 23.03 | 10.69 | 9.94 | 24.58 | 24.65 | 4.77 | 3.04E-25 | 1.32 | 2.35E-03 | 1137 |
| Cluster-40555.171757 | 0.06 | 0.1 | 1.77 | 2.23 | 0.53 | 0.37 | 2.24 | 3.19 | 4.77 | 7.34E-03 | 2.67 | 3.41E-02 | 1062 |
| Cluster-40555.255105 | 0.14 | 0.06 | 2.63 | 2.28 | 0.07 | 0.29 | 3.74 | 1.69 | 4.77 | 6.30E-03 | 3.93 | 2.31E-02 | 941 |
| Cluster-40555.154454 | 0.32 | 0.14 | 7.06 | 4.72 | 0.39 | 0 | 5.25 | 4.39 | 4.78 | 1.51E-05 | 4.66 | 4.37E-05 | 832 |
| Cluster-40555.142424 | 0.4 | 1.05 | 20.77 | 16.04 | 5.78 | 6.47 | 19.82 | 22.41 | 4.78 | 3.85E-16 | 1.85 | 1.17E-04 | 870 |
| Cluster-40555.177657 | 0.23 | 0 | 2.61 | 3.2 | 0.53 | 0.29 | 3.52 | 4.95 | 4.78 | 4.41E-05 | 3.45 | 2.64E-05 | 1251 |
| Cluster-40555.202564 | 0.04 | 0.22 | 3.29 | 3.51 | 0.43 | 0.23 | 5.08 | 2.82 | 4.78 | 3.63E-06 | 3.66 | 5.24E-04 | 1346 |

| | | | | | | | | | | | | | |
|---|---|---|---|---|---|---|---|---|---|---|---|---|---|
| Cluster-40555.225029 | 0.31 | 0.32 | 8.1 | 7.93 | 0.36 | 0.69 | 4.89 | 4.66 | 4.78 | 7.70E-19 | 3.23 | 3.81E-08 | 1901 |
| Cluster-40555.202477 | 0.09 | 0.24 | 3.85 | 4.47 | 2.22 | 1.64 | 6.22 | 6.08 | 4.78 | 6.41E-14 | 1.74 | 2.68E-04 | 2439 |
| Cluster-40555.190185 | 1.06 | 0.58 | 21.29 | 19.6 | 12.97 | 6.4 | 22.57 | 26.82 | 4.78 | 7.28E-41 | 1.44 | 2.02E-03 | 2247 |
| Cluster-40555.187468 | 3.91 | 4.11 | 100.21 | 99.83 | 26.52 | 25.97 | 76.98 | 80.14 | 4.78 | 3.77E-39 | 1.65 | 1.78E-05 | 638 |
| Cluster-40555.205694 | 0.97 | 0.81 | 21.19 | 23.05 | 8.17 | 3.54 | 18.21 | 15.73 | 4.79 | 4.56E-27 | 1.62 | 4.82E-04 | 1207 |
| Cluster-40555.234245 | 0.28 | 0 | 2.67 | 4.02 | 0.14 | 0.15 | 14.99 | 16.57 | 4.79 | 3.48E-02 | 6.86 | 7.21E-13 | 607 |
| Cluster-40555.184708 | 0 | 0.14 | 1.65 | 1.98 | 0.99 | 0.72 | 4.69 | 2.87 | 4.79 | 1.27E-03 | 2.20 | 5.67E-03 | 1408 |
| Cluster-40555.187878 | 8.88 | 9.46 | 236.9 | 221.43 | 98.83 | 93.29 | 263.15 | 245.77 | 4.79 | 1.41E-50 | 1.46 | 1.70E-05 | 534 |
| Cluster-40555.213102 | 0.12 | 0.96 | 14.21 | 14.09 | 0 | 0.04 | 0.88 | 1.39 | 4.79 | 2.92E-23 | 5.84 | 2.94E-03 | 1496 |
| Cluster-40555.196261 | 0.51 | 0.78 | 16.54 | 16.52 | 6.75 | 8.23 | 32.38 | 39.61 | 4.79 | 9.95E-40 | 2.33 | 4.51E-10 | 2605 |
| Cluster-40555.189758 | 0 | 1.38 | 16.45 | 19.97 | 0 | 1.52 | 9.34 | 7.08 | 4.80 | 5.76E-17 | 3.47 | 1.26E-06 | 894 |
| Cluster-40555.173293 | 0.72 | 0 | 9.27 | 8.27 | 2.68 | 2.47 | 6.83 | 6.43 | 4.80 | 2.12E-32 | 1.43 | 9.14E-04 | 3328 |
| Cluster-40555.199200 | 0.22 | 0 | 2.41 | 2.94 | 0.14 | 0.33 | 1.98 | 1.91 | 4.80 | 3.05E-19 | 3.09 | 8.92E-09 | 5067 |
| Cluster-40555.208976 | 3.77 | 0 | 47.93 | 42.53 | 2.12 | 3.66 | 21.9 | 28.57 | 4.80 | 6.74E-33 | 3.19 | 7.31E-10 | 870 |
| Cluster-40555.181660 | 0.1 | 0 | 1.34 | 0.97 | 0 | 0 | 0.96 | 1.28 | 4.80 | 4.05E-02 | Inf | 3.61E-03 | 1287 |
| Cluster-40555.182003 | 0.89 | 0.96 | 23.26 | 22.61 | 7.36 | 8.29 | 32.98 | 45.09 | 4.80 | 2.88E-20 | 2.38 | 1.93E-05 | 887 |
| Cluster-40555.189085 | 2.2 | 3.47 | 72.06 | 72.82 | 7.63 | 6.23 | 19.3 | 23.79 | 4.80 | 7.34E-59 | 1.71 | 1.27E-05 | 1509 |
| Cluster-40555.206681 | 0.41 | 1.35 | 23.11 | 22.15 | 1.29 | 0.25 | 4.44 | 6.72 | 4.80 | 9.34E-41 | 2.98 | 1.09E-03 | 2075 |
| Cluster-40555.196957 | 1.31 | 2.22 | 41.08 | 50.08 | 3.62 | 3.55 | 54.87 | 57.91 | 4.81 | 1.97E-28 | 4.04 | 3.92E-26 | 909 |
| Cluster-40555.177062 | 0.24 | 0 | 2.6 | 3.15 | 0.21 | 0 | 2.15 | 1.76 | 4.81 | 3.46E-05 | 4.21 | 2.06E-03 | 1285 |
| Cluster-40555.143068 | 0.16 | 0.26 | 5.3 | 5.42 | 0 | 0.08 | 4.79 | 4.77 | 4.81 | 1.31E-05 | 6.91 | 2.02E-07 | 877 |
| Cluster-40555.189571 | 17.44 | 31.35 | 603.75 | 655.57 | 226.01 | 178.99 | 665.33 | 659.03 | 4.82 | 2.03E-86 | 1.78 | 2.15E-09 | 1369 |
| Cluster-40555.178265 | 0.18 | 0.26 | 5.98 | 5.82 | 0.25 | 0.24 | 3.95 | 3.32 | 4.82 | 7.32E-10 | 3.90 | 1.11E-05 | 1295 |
| Cluster-40555.244723 | 0 | 0.12 | 1.4 | 1.51 | 0.04 | 0 | 0.76 | 0.66 | 4.82 | 1.09E-03 | 5.60 | 2.33E-02 | 1728 |
| Cluster-40555.214708 | 0.13 | 0.28 | 5.86 | 4.94 | 0.66 | 1.24 | 6.1 | 5.26 | 4.82 | 2.08E-06 | 2.63 | 1.11E-03 | 977 |
| Cluster-40555.197785 | 0.25 | 0.74 | 11.94 | 13.82 | 0 | 0.62 | 7.01 | 8.43 | 4.82 | 8.79E-18 | 4.62 | 4.11E-12 | 1194 |
| Cluster-40555.228584 | 0.42 | 0.14 | 6.69 | 7.47 | 0.08 | 0 | 6.52 | 5.35 | 4.82 | 6.56E-07 | 7.27 | 1.41E-08 | 832 |
| Cluster-40555.218967 | 2.4 | 0 | 31.83 | 27.84 | 2.87 | 0 | 23.27 | 20.53 | 4.83 | 1.97E-40 | 4.04 | 7.73E-23 | 1542 |
| Cluster-40555.236114 | 0.05 | 0.1 | 1.41 | 2.12 | 0.17 | 0.08 | 2.06 | 1.27 | 4.83 | 6.38E-04 | 3.73 | 3.52E-03 | 1510 |
| Cluster-40555.218856 | 0.06 | 0.16 | 2.89 | 2.74 | 0.14 | 0 | 1.27 | 2.13 | 4.83 | 2.74E-14 | 4.73 | 1.32E-04 | 3590 |
| Cluster-40555.177580 | 0 | 0.25 | 3.46 | 3.57 | 0.85 | 0.17 | 3.43 | 2.65 | 4.83 | 1.31E-04 | 2.63 | 2.97E-02 | 1032 |
| Cluster-40555.248813 | 0.04 | 0 | 0.35 | 0.56 | 0.04 | 0 | 1.09 | 1.31 | 4.84 | 2.81E-02 | 6.21 | 1.83E-07 | 2900 |

| | | | | | | | | | | | | | |
|---|---|---|---|---|---|---|---|---|---|---|---|---|---|
| Cluster-40555.196917 | 1.37 | 0 | 18.97 | 15.11 | 10.02 | 10.02 | 28.35 | 22.1 | 4.84 | 1.80E-16 | 1.39 | 2.87E-03 | 899 |
| Cluster-40555.272094 | 0 | 0.16 | 2.15 | 2.34 | 0 | 0 | 1.32 | 1.36 | 4.84 | 7.03E-03 | Inf | 1.22E-02 | 991 |
| Cluster-40555.215438 | 0.54 | 0.25 | 9.64 | 10.8 | 2.41 | 1.39 | 7.78 | 6.99 | 4.84 | 2.50E-22 | 2.04 | 5.01E-05 | 1795 |
| Cluster-40555.180749 | 2.22 | 1.38 | 50.34 | 42.45 | 0.67 | 1.76 | 35.87 | 41.29 | 4.85 | 2.88E-19 | 5.03 | 1.08E-17 | 548 |
| Cluster-40555.190447 | 0.42 | 0.58 | 13.7 | 12.19 | 1 | 1.23 | 9.55 | 7.9 | 4.85 | 1.36E-38 | 3.04 | 1.33E-14 | 3069 |
| Cluster-40555.276826 | 0.1 | 0.04 | 2.03 | 1.6 | 0 | 0 | 1.63 | 1.51 | 4.85 | 4.22E-03 | Inf | 3.33E-04 | 1232 |
| Cluster-40555.218892 | 0.27 | 0.22 | 7.27 | 5.69 | 2.15 | 2.54 | 15.46 | 8.87 | 4.85 | 5.59E-04 | 2.43 | 8.70E-03 | 617 |
| Cluster-40555.230979 | 0.05 | 0.03 | 1.03 | 1.06 | 0.27 | 0.37 | 1.98 | 2.23 | 4.85 | 3.74E-03 | 2.75 | 1.70E-03 | 1949 |
| Cluster-40555.175597 | 0.75 | 0.11 | 13.92 | 7.93 | 1.43 | 0.48 | 8.75 | 11.76 | 4.85 | 6.35E-07 | 3.52 | 1.09E-08 | 2903 |
| Cluster-40555.220928 | 0.13 | 0 | 1.73 | 2.21 | 0.12 | 0 | 2.3 | 2.38 | 4.85 | 2.52E-03 | 5.64 | 8.49E-05 | 1176 |
| Cluster-40555.204576 | 0.31 | 0.18 | 6.32 | 6.47 | 0.56 | 0.43 | 8.17 | 9.03 | 4.85 | 5.90E-21 | 4.20 | 9.56E-21 | 2538 |
| Cluster-40555.156856 | 0.05 | 0.04 | 0.96 | 1.29 | 0 | 0 | 1.68 | 2.19 | 4.85 | 3.80E-02 | Inf | 1.41E-05 | 1288 |
| Cluster-40555.187798 | 2.86 | 2.7 | 64.17 | 78.58 | 14.07 | 6.49 | 67.53 | 75.64 | 4.85 | 1.57E-28 | 2.88 | 1.74E-14 | 711 |
| Cluster-40555.159912 | 0.29 | 0.94 | 18.13 | 14.88 | 0.37 | 0.68 | 3.5 | 3.62 | 4.86 | 5.84E-13 | 2.81 | 4.83E-02 | 771 |
| Cluster-40555.210053 | 0.04 | 0 | 0.57 | 0.52 | 0 | 0 | 0.46 | 0.2 | 4.86 | 2.77E-03 | Inf | 5.04E-03 | 3637 |
| Cluster-40555.144729 | 0.29 | 0.16 | 7.28 | 4.3 | 2.8 | 1.47 | 9.25 | 6.45 | 4.86 | 6.21E-05 | 1.95 | 2.59E-02 | 769 |
| Cluster-40555.246984 | 0.4 | 0 | 3.57 | 6.5 | 1.56 | 1.01 | 7.65 | 11.82 | 4.86 | 2.05E-03 | 3.01 | 9.22E-04 | 623 |
| Cluster-40555.144362 | 0 | 0.2 | 2.82 | 2.78 | 0.06 | 0.06 | 1.87 | 1.39 | 4.86 | 8.13E-04 | 4.79 | 1.38E-02 | 1048 |
| Cluster-40555.126225 | 0.04 | 0.03 | 0.78 | 1.16 | 0.04 | 0 | 0.68 | 0.9 | 4.86 | 3.50E-02 | 5.46 | 4.16E-02 | 1453 |
| Cluster-40555.205961 | 0.13 | 0.23 | 5.44 | 4.16 | 1.11 | 0.79 | 4.68 | 3.02 | 4.87 | 3.17E-09 | 2.11 | 6.21E-03 | 1456 |
| Cluster-40555.194341 | 0.89 | 0.59 | 18.97 | 19.84 | 27.73 | 34.27 | 67.17 | 62 | 4.87 | 7.50E-20 | 1.11 | 3.63E-03 | 958 |
| Cluster-40555.207867 | 0 | 0.86 | 12.91 | 11.2 | 0 | 0 | 6.91 | 9.19 | 4.87 | 1.04E-04 | Inf | 4.72E-05 | 492 |
| Cluster-40555.184247 | 0.14 | 0.04 | 2.43 | 2.16 | 0.77 | 0.81 | 3.9 | 3.84 | 4.88 | 4.05E-08 | 2.34 | 3.72E-05 | 2359 |
| Cluster-40555.198499 | 0 | 0.06 | 0.96 | 0.83 | 0.34 | 1.45 | 2.88 | 2.73 | 4.88 | 6.37E-03 | 1.67 | 1.96E-02 | 2144 |
| Cluster-40555.177879 | 0 | 0.43 | 4.72 | 7.41 | 1.08 | 1.41 | 18.49 | 11.97 | 4.88 | 4.48E-02 | 3.65 | 7.28E-04 | 443 |
| Cluster-40555.128223 | 0.24 | 0.13 | 4.52 | 5.36 | 0 | 0 | 8.15 | 7.01 | 4.88 | 3.06E-05 | Inf | 1.21E-12 | 861 |
| Cluster-40555.137142 | 0.08 | 0.04 | 1.02 | 1.97 | 0.15 | 0.15 | 3.73 | 3.96 | 4.88 | 1.11E-03 | 4.75 | 7.65E-15 | 2651 |
| Cluster-40555.175642 | 0 | 0.46 | 5.45 | 7.25 | 0.13 | 0.66 | 5.99 | 5.74 | 4.89 | 9.52E-12 | 3.96 | 1.59E-09 | 1399 |
| Cluster-40555.185738 | 0.61 | 0.29 | 13.46 | 10.4 | 0.77 | 0.72 | 6.63 | 7.61 | 4.89 | 1.11E-10 | 3.33 | 8.23E-05 | 815 |
| Cluster-40555.139777 | 0.17 | 0 | 3.51 | 1.63 | 0 | 0.13 | 3.4 | 4.34 | 4.89 | 3.01E-03 | 5.71 | 1.77E-08 | 1247 |
| Cluster-40555.197278 | 0.06 | 0.13 | 1.91 | 3.03 | 0.08 | 0.07 | 2.03 | 2.51 | 4.90 | 3.60E-04 | 5.52 | 1.12E-04 | 1191 |
| Cluster-40555.157187 | 0 | 0.15 | 2.29 | 1.86 | 0 | 0 | 1.49 | 3.6 | 4.91 | 6.45E-04 | Inf | 4.29E-03 | 1356 |

| | | | | | | | | | | | | | |
|---|---|---|---|---|---|---|---|---|---|---|---|---|---|
| Cluster-40555.218329 | 0.14 | 0.04 | 2.46 | 1.98 | 0.09 | 0.3 | 2.04 | 1.9 | 4.91 | 2.43E-04 | 3.37 | 4.84E-03 | 1376 |
| Cluster-40555.180622 | 3.07 | 4.48 | 104.33 | 92.25 | 4.67 | 1.14 | 67.99 | 43.99 | 4.91 | 8.21E-09 | 4.26 | 2.23E-04 | 307 |
| Cluster-40555.193307 | 2.69 | 1.8 | 63.24 | 59.62 | 1.03 | 3.35 | 25.3 | 29.65 | 4.91 | 1.43E-25 | 3.67 | 5.19E-10 | 566 |
| Cluster-40555.192873 | 0 | 0.04 | 0.84 | 0.4 | 0.27 | 0.18 | 1.23 | 0.71 | 4.92 | 6.39E-03 | 2.19 | 4.67E-02 | 3048 |
| Cluster-40555.184849 | 1.69 | 1.67 | 44.13 | 47.57 | 8.62 | 8.7 | 25.68 | 30.17 | 4.92 | 1.87E-53 | 1.76 | 9.83E-07 | 1663 |
| Cluster-40555.173919 | 0.09 | 0 | 1.29 | 1.14 | 0 | 0 | 1.17 | 2.31 | 4.92 | 2.35E-02 | Inf | 3.88E-04 | 1324 |
| Cluster-40555.177833 | 0.5 | 0.31 | 12.38 | 10.48 | 3.96 | 3.1 | 11.88 | 13.17 | 4.92 | 9.74E-24 | 1.90 | 7.92E-06 | 1755 |
| Cluster-40555.181316 | 0.36 | 1.16 | 21.7 | 20.79 | 0.7 | 0 | 16.01 | 13.81 | 4.92 | 1.23E-09 | 5.52 | 2.28E-08 | 536 |
| Cluster-40555.217567 | 0 | 0.23 | 4.07 | 2.91 | 0.05 | 0.09 | 2.12 | 1.51 | 4.92 | 2.07E-08 | 4.52 | 5.72E-05 | 1744 |
| Cluster-40555.175615 | 1.54 | 1.5 | 38.01 | 44.66 | 11.59 | 2.41 | 44.29 | 43.34 | 4.92 | 3.33E-09 | 2.73 | 1.44E-04 | 391 |
| Cluster-40555.222611 | 0.18 | 0.36 | 7.42 | 6.33 | 0.52 | 0.56 | 16.97 | 13.75 | 4.92 | 5.14E-07 | 4.83 | 4.65E-15 | 865 |
| Cluster-40555.213043 | 0 | 0.09 | 1.68 | 0.81 | 0.12 | 0 | 1.63 | 1.88 | 4.93 | 1.05E-03 | 5.09 | 4.60E-12 | 3994 |
| Cluster-40555.217091 | 0.2 | 0 | 2.22 | 3.19 | 0.78 | 0 | 8.3 | 8.08 | 4.93 | 2.16E-10 | 4.50 | 4.03E-26 | 3573 |
| Cluster-40555.188883 | 4.31 | 6.07 | 149.46 | 140.41 | 33.53 | 18.8 | 76.29 | 112.44 | 4.93 | 1.35E-45 | 1.93 | 8.70E-03 | 585 |
| Cluster-40555.179895 | 0.5 | 0.21 | 9.89 | 9.55 | 4.85 | 4.37 | 15 | 16.82 | 4.94 | 5.22E-21 | 1.85 | 3.45E-06 | 1730 |
| Cluster-40555.242421 | 0.19 | 0 | 1.79 | 2.65 | 0 | 0 | 3.29 | 3.05 | 4.94 | 1.33E-03 | Inf | 7.59E-08 | 1133 |
| Cluster-40555.144298 | 0.13 | 0 | 1.57 | 1.41 | 0.2 | 0 | 2.15 | 1.16 | 4.94 | 2.10E-02 | 4.04 | 2.09E-02 | 1125 |
| Cluster-40555.169195 | 0 | 0.17 | 3.12 | 1.88 | 0.18 | 0.22 | 3.54 | 3.1 | 4.95 | 4.35E-02 | 4.09 | 1.06E-02 | 737 |
| Cluster-40555.223636 | 0.26 | 0 | 4.01 | 2.96 | 0.04 | 0.13 | 2.07 | 1.34 | 4.96 | 7.33E-08 | 4.56 | 2.86E-04 | 1576 |
| Cluster-40555.136769 | 0.08 | 0 | 1.53 | 0.77 | 0 | 0 | 0.99 | 0.63 | 4.96 | 1.84E-03 | Inf | 6.90E-04 | 1990 |
| Cluster-40555.223669 | 0 | 0.35 | 4.51 | 5.87 | 0 | 0 | 1.29 | 1.6 | 4.96 | 6.76E-10 | Inf | 1.35E-04 | 1393 |
| Cluster-40555.195174 | 0.14 | 0.27 | 5.96 | 5.45 | 3.02 | 2.48 | 7 | 7.21 | 4.96 | 1.28E-17 | 1.44 | 3.01E-03 | 2295 |
| Cluster-40555.163501 | 0.46 | 0.71 | 17.93 | 15.61 | 6.09 | 2.43 | 54.56 | 59.36 | 4.96 | 2.73E-03 | 3.81 | 1.16E-07 | 369 |
| Cluster-40555.201587 | 0.06 | 0.33 | 5.87 | 4.85 | 0 | 0 | 3.81 | 4.23 | 4.97 | 8.24E-10 | Inf | 1.32E-12 | 1379 |
| Cluster-40555.165936 | 0.42 | 0.35 | 8.61 | 13.18 | 1.66 | 1.74 | 6.7 | 7.06 | 4.97 | 8.22E-08 | 2.08 | 3.85E-02 | 719 |
| Cluster-40555.180268 | 0.39 | 0.23 | 7.92 | 7.91 | 0.97 | 0.88 | 7.01 | 7.19 | 4.98 | 1.10E-10 | 3.00 | 1.17E-05 | 1062 |
| Cluster-40555.176360 | 0.11 | 0 | 0.86 | 1.97 | 0 | 0 | 1.15 | 0.81 | 4.98 | 9.47E-03 | Inf | 4.29E-05 | 2114 |
| Cluster-40555.125735 | 0.07 | 0.06 | 2.28 | 1.47 | 0.49 | 0.66 | 3.39 | 3.34 | 4.98 | 2.73E-02 | 2.61 | 3.02E-02 | 936 |
| Cluster-40555.187958 | 20.05 | 8.98 | 390.64 | 429.19 | 82.18 | 51.61 | 741.37 | 740.54 | 4.98 | 6.51E-85 | 3.55 | 1.77E-32 | 1015 |
| Cluster-40555.218253 | 0.2 | 0.44 | 7.54 | 11.02 | 4.91 | 3.94 | 18.21 | 13.51 | 4.98 | 1.60E-09 | 1.91 | 1.33E-04 | 989 |
| Cluster-40555.180572 | 2.64 | 1.69 | 56.73 | 64.09 | 85.42 | 86.63 | 176.83 | 161.36 | 4.99 | 9.89E-12 | 1.02 | 4.55E-02 | 375 |
| Cluster-40555.190542 | 1.04 | 1.01 | 31.15 | 27.35 | 5.9 | 4.26 | 24.74 | 22.9 | 4.99 | 4.38E-45 | 2.30 | 1.43E-10 | 1796 |

| | | | | | | | | | | | | | |
|---|---|---|---|---|---|---|---|---|---|---|---|---|---|
| Cluster-40555.193114 | 0.24 | 0 | 1.96 | 3.91 | 0.37 | 0.59 | 5.18 | 6.7 | 4.99 | 1.33E-02 | 3.67 | 6.83E-04 | 715 |
| Cluster-40555.231736 | 0.18 | 0.05 | 2.99 | 3.32 | 0.24 | 0.18 | 3.02 | 2.67 | 4.99 | 9.87E-05 | 3.87 | 1.15E-03 | 1083 |
| Cluster-40555.144289 | 0 | 0.16 | 3.17 | 1.67 | 0.11 | 0 | 1.16 | 1.47 | 4.99 | 9.02E-05 | 4.62 | 7.12E-05 | 2273 |
| Cluster-40555.188391 | 0.25 | 0.47 | 10.9 | 10.67 | 4.22 | 4.26 | 9.15 | 9.29 | 4.99 | 1.47E-24 | 1.19 | 1.89E-02 | 1878 |
| Cluster-40555.131687 | 0 | 0.22 | 3.96 | 2.67 | 0 | 0.23 | 2.53 | 1.86 | 4.99 | 3.55E-03 | 4.59 | 2.41E-02 | 805 |
| Cluster-40555.193653 | 0 | 0.16 | 2.42 | 2.61 | 1.17 | 1.25 | 2.84 | 3.54 | 4.99 | 3.30E-08 | 1.46 | 4.29E-02 | 2176 |
| Cluster-40555.188648 | 43.68 | 62.8 | 1486.55 | 1610.18 | 892.99 | 809.46 | 1600.04 | 1417.79 | 4.99 | 1.61E-89 | 0.89 | 1.40E-02 | 606 |
| Cluster-40555.277482 | 0.07 | 0 | 1.68 | 1.03 | 0.31 | 0.12 | 1.69 | 2.16 | 5.00 | 1.85E-02 | 3.25 | 1.81E-02 | 1251 |
| Cluster-40555.236934 | 0.34 | 0.28 | 7.35 | 10.29 | 0.55 | 0.88 | 10.58 | 14.38 | 5.00 | 6.28E-12 | 4.18 | 2.15E-10 | 1268 |
| Cluster-40555.190133 | 16.71 | 13.95 | 445.3 | 442.42 | 234.51 | 223.69 | 435.81 | 336.57 | 5.00 | 5.52E-90 | 0.82 | 2.98E-02 | 1490 |
| Cluster-40555.190486 | 0.18 | 0.95 | 15.01 | 18.57 | 5.75 | 2.36 | 23.53 | 23.35 | 5.00 | 1.01E-21 | 2.62 | 2.87E-10 | 1137 |
| Cluster-40555.165900 | 1.14 | 0 | 20.34 | 11.86 | 0 | 0 | 4.99 | 3.89 | 5.00 | 1.06E-07 | Inf | 2.32E-17 | 1692 |
| Cluster-40555.190830 | 0.48 | 0.08 | 7.74 | 7.91 | 6.49 | 6.27 | 14.49 | 13.09 | 5.00 | 1.71E-19 | 1.17 | 8.37E-03 | 1874 |
| Cluster-40555.206288 | 0 | 0.27 | 4.13 | 4.35 | 0.28 | 0 | 1.25 | 2.03 | 5.00 | 3.36E-09 | 3.62 | 5.09E-03 | 1543 |
| Cluster-40555.182299 | 1.13 | 1.21 | 36.25 | 32.49 | 19.85 | 25.46 | 45.19 | 44.76 | 5.00 | 3.48E-61 | 1.05 | 2.78E-03 | 2801 |
| Cluster-40555.257338 | 0 | 0.06 | 0.63 | 1.18 | 0.03 | 0 | 1.53 | 2.15 | 5.00 | 2.61E-02 | 6.83 | 4.06E-06 | 1584 |
| Cluster-40555.204535 | 0 | 0.21 | 3.08 | 3.29 | 0.97 | 1.02 | 4.57 | 3.92 | 5.01 | 2.61E-08 | 2.14 | 7.24E-04 | 1787 |
| Cluster-40555.149376 | 0.23 | 0 | 2.55 | 3.89 | 0.08 | 0.08 | 6.22 | 5.77 | 5.02 | 7.37E-04 | 6.35 | 6.73E-09 | 889 |
| Cluster-40555.154499 | 0.37 | 0 | 5.76 | 4.43 | 1.09 | 0.38 | 10.29 | 10.38 | 5.02 | 1.56E-02 | 3.90 | 3.89E-04 | 528 |
| Cluster-40555.132076 | 0.19 | 0 | 1.98 | 3.46 | 0.14 | 0.1 | 2.33 | 2.34 | 5.02 | 7.68E-05 | 4.36 | 3.65E-04 | 1260 |
| Cluster-40555.156323 | 0.05 | 0 | 0.77 | 0.87 | 0.23 | 0.06 | 0.81 | 1.26 | 5.02 | 8.09E-04 | 2.97 | 7.03E-03 | 2750 |
| Cluster-40555.160565 | 0.94 | 0.68 | 21.74 | 24.1 | 1.21 | 0.32 | 10.69 | 13.61 | 5.03 | 4.70E-11 | 4.05 | 3.10E-05 | 544 |
| Cluster-40555.135001 | 0 | 0.12 | 2.12 | 1.43 | 0.24 | 0 | 0.86 | 1.1 | 5.03 | 2.62E-05 | 3.15 | 3.81E-02 | 1999 |
| Cluster-40555.167531 | 0 | 0.6 | 8.23 | 9.18 | 0 | 0.12 | 5.08 | 6.25 | 5.03 | 1.71E-06 | 6.60 | 6.70E-06 | 684 |
| Cluster-40555.193355 | 1.06 | 1.16 | 30.88 | 35.38 | 1.97 | 4.01 | 64.48 | 44.01 | 5.03 | 9.47E-21 | 4.22 | 3.43E-12 | 672 |
| Cluster-40555.182911 | 0.7 | 1.37 | 31.08 | 31.5 | 9.09 | 5.75 | 66.73 | 72.49 | 5.04 | 1.07E-24 | 3.31 | 1.62E-19 | 814 |
| Cluster-40555.190437 | 0.08 | 0 | 1.76 | 0.97 | 0.27 | 0.25 | 1.6 | 1.95 | 5.04 | 1.02E-03 | 2.81 | 7.45E-03 | 1790 |
| Cluster-40555.201040 | 0 | 0.25 | 4.51 | 3.27 | 0 | 0 | 4.11 | 4.32 | 5.04 | 2.29E-04 | Inf | 1.23E-07 | 904 |
| Cluster-40555.124415 | 0 | 0.1 | 1.31 | 2.09 | 0 | 0 | 1.24 | 2.13 | 5.04 | 1.78E-03 | Inf | 3.25E-05 | 1364 |
| Cluster-40555.204949 | 0.33 | 0 | 5.72 | 3.92 | 0.1 | 1.52 | 2.96 | 4.11 | 5.05 | 1.15E-13 | 2.15 | 3.24E-02 | 2002 |
| Cluster-40555.138083 | 0 | 0.11 | 2.04 | 1.58 | 0 | 0 | 1.96 | 3.63 | 5.05 | 2.10E-04 | Inf | 1.32E-05 | 1658 |
| Cluster-40555.185668 | 1.48 | 3.29 | 67.69 | 77.7 | 2.11 | 1.52 | 62.59 | 59.52 | 5.05 | 1.34E-33 | 5.14 | 6.12E-28 | 624 |

| | | | | | | | | | | | | | |
|---|---|---|---|---|---|---|---|---|---|---|---|---|---|
| Cluster-40555.211634 | 0.33 | 0 | 4.25 | 5.49 | 3.81 | 3.65 | 7.73 | 8.33 | 5.05 | 3.34E-15 | 1.17 | 2.25E-02 | 2115 |
| Cluster-40555.100515 | 0.1 | 0 | 1.58 | 1.4 | 0 | 0 | 2.6 | 2.61 | 5.05 | 1.28E-02 | Inf | 6.73E-07 | 1198 |
| Cluster-40555.198931 | 0.17 | 0.93 | 18.99 | 15.39 | 1.37 | 1.19 | 4.32 | 5.95 | 5.05 | 7.66E-25 | 2.07 | 3.79E-03 | 1301 |
| Cluster-40555.184242 | 1.43 | 3.11 | 69.15 | 68.74 | 2.59 | 1.25 | 57.43 | 55.2 | 5.05 | 2.42E-49 | 4.96 | 3.63E-36 | 1012 |
| Cluster-40555.197401 | 0.81 | 0.46 | 19.42 | 18.69 | 0.18 | 0.55 | 15.98 | 13.61 | 5.06 | 3.45E-35 | 5.34 | 1.15E-29 | 1743 |
| Cluster-40555.185761 | 2.33 | 8.35 | 151.13 | 182.72 | 18.74 | 18.47 | 128.43 | 125.07 | 5.06 | 6.41E-32 | 2.82 | 3.85E-11 | 414 |
| Cluster-40555.200534 | 0.09 | 0.38 | 5.94 | 8.64 | 0.18 | 1.6 | 17.36 | 10.37 | 5.07 | 5.96E-07 | 3.98 | 1.01E-05 | 790 |
| Cluster-40555.162321 | 0 | 0.23 | 3.92 | 3.96 | 0.29 | 0 | 2.68 | 3.67 | 5.07 | 1.53E-04 | 4.56 | 9.34E-04 | 905 |
| Cluster-40555.189395 | 0.54 | 0.62 | 19 | 16.22 | 0.63 | 0.78 | 14.62 | 15.95 | 5.07 | 4.72E-20 | 4.50 | 2.80E-16 | 1015 |
| Cluster-40555.173963 | 0.31 | 0 | 2.93 | 6.07 | 1.57 | 1.4 | 4.56 | 7.24 | 5.07 | 2.01E-03 | 2.07 | 2.42E-02 | 1040 |
| Cluster-40555.215860 | 0.51 | 0 | 6.54 | 7.48 | 0 | 0 | 1.89 | 1.31 | 5.07 | 1.12E-07 | Inf | 1.48E-02 | 865 |
| Cluster-40555.180440 | 0.4 | 0.43 | 10.66 | 11.73 | 4.96 | 3.22 | 18.12 | 20.41 | 5.07 | 3.19E-08 | 2.31 | 7.63E-05 | 676 |
| Cluster-40555.193633 | 3.48 | 4.06 | 117.2 | 114.55 | 8.15 | 10.49 | 73.12 | 85.33 | 5.08 | 3.21E-78 | 3.15 | 4.79E-23 | 1869 |
| Cluster-40555.192543 | 0.48 | 0.67 | 15.38 | 20.06 | 9.09 | 8.52 | 17.67 | 16.82 | 5.08 | 1.23E-19 | 1.04 | 1.02E-02 | 2771 |
| Cluster-40555.150776 | 0 | 0.05 | 0.79 | 0.76 | 0.18 | 0.08 | 1.45 | 1.5 | 5.08 | 1.76E-03 | 3.65 | 4.42E-05 | 2722 |
| Cluster-40555.175882 | 0.16 | 0.04 | 3.64 | 2.88 | 0.29 | 0 | 1.09 | 0.94 | 5.08 | 1.09E-12 | 2.99 | 1.10E-02 | 2603 |
| Cluster-40555.258556 | 0.07 | 0.06 | 1.95 | 1.94 | 0.07 | 0 | 3.86 | 3.05 | 5.08 | 1.51E-02 | 6.80 | 5.20E-06 | 958 |
| Cluster-40555.189075 | 1.9 | 1.71 | 57.78 | 53.11 | 2.98 | 2.64 | 17.09 | 20.93 | 5.08 | 2.15E-74 | 2.83 | 3.72E-13 | 2971 |
| Cluster-40555.189720 | 18.44 | 10.49 | 394.45 | 486.25 | 106.19 | 61.1 | 624.82 | 695.56 | 5.09 | 1.34E-30 | 3.06 | 2.29E-25 | 1428 |
| Cluster-40555.219293 | 0 | 0.78 | 14.19 | 11.28 | 0 | 0 | 6.32 | 9.02 | 5.09 | 1.82E-03 | Inf | 6.02E-03 | 412 |
| Cluster-40555.214182 | 0.01 | 0.02 | 0.41 | 0.61 | 0.02 | 0.13 | 0.64 | 0.84 | 5.09 | 9.35E-04 | 3.24 | 9.82E-04 | 4176 |
| Cluster-40555.167257 | 0.73 | 3.1 | 59.9 | 61.93 | 8 | 3.39 | 38.44 | 42.72 | 5.09 | 8.82E-32 | 2.92 | 1.08E-10 | 660 |
| Cluster-40555.215558 | 0.49 | 0.37 | 11.13 | 14.8 | 0 | 0 | 16.13 | 14.75 | 5.09 | 1.31E-02 | Inf | 4.75E-04 | 364 |
| Cluster-40555.185702 | 1.44 | 1.12 | 37.05 | 40.39 | 4.7 | 4.84 | 49.65 | 46.35 | 5.10 | 6.35E-22 | 3.38 | 2.24E-14 | 632 |
| Cluster-40555.159575 | 0 | 0.06 | 0.82 | 0.91 | 0 | 0.21 | 0.85 | 1.16 | 5.10 | 2.00E-02 | 3.36 | 3.60E-02 | 1744 |
| Cluster-40555.241751 | 0.38 | 0.14 | 7.9 | 5.36 | 0.37 | 0 | 5.53 | 5.79 | 5.10 | 4.79E-05 | 5.19 | 6.86E-05 | 686 |
| Cluster-40555.187327 | 0.23 | 1.05 | 19.03 | 21.81 | 1.88 | 1.86 | 13.52 | 16.57 | 5.10 | 2.79E-47 | 3.08 | 1.34E-14 | 2525 |
| Cluster-40555.189213 | 0.16 | 0.09 | 4.47 | 3.71 | 0.17 | 0.14 | 15.76 | 13.63 | 5.10 | 1.00E-11 | 6.64 | 1.08E-38 | 1961 |
| Cluster-40555.192039 | 0.52 | 0.31 | 12.7 | 13.03 | 3.01 | 1.95 | 11.07 | 16.21 | 5.11 | 1.91E-25 | 2.54 | 5.55E-04 | 1613 |
| Cluster-40555.189194 | 3.56 | 3.39 | 101.14 | 116.42 | 44.6 | 50.92 | 182.46 | 126.04 | 5.11 | 1.69E-55 | 1.75 | 9.45E-05 | 2054 |
| Cluster-40555.195274 | 0.09 | 0.32 | 6.59 | 6.37 | 1.33 | 0.67 | 3.67 | 4.13 | 5.11 | 1.07E-25 | 2.04 | 6.94E-05 | 3045 |
| Cluster-40555.151902 | 0.13 | 0.14 | 3.82 | 4.04 | 0.64 | 0.38 | 4.29 | 3.71 | 5.12 | 1.68E-08 | 3.06 | 3.88E-05 | 1483 |

| | | | | | | | | | | | | | |
|---|---|---|---|---|---|---|---|---|---|---|---|---|---|
| Cluster-40555.189507 | 30.52 | 28.55 | 920.18 | 931.15 | 272.95 | 245.83 | 712.68 | 757.05 | 5.12 | 1.20E-81 | 1.56 | 7.09E-07 | 491 |
| Cluster-40555.169014 | 0.45 | 0.79 | 22.34 | 21.75 | 0 | 0 | 15.31 | 9.97 | 5.12 | 1.55E-10 | Inf | 1.25E-09 | 538 |
| Cluster-40555.170043 | 0.67 | 0 | 10.26 | 10.08 | 0.63 | 0.5 | 6.05 | 7.34 | 5.12 | 1.77E-11 | 3.67 | 6.89E-06 | 908 |
| Cluster-40555.158778 | 0.17 | 0 | 2.19 | 2.74 | 0.17 | 0.27 | 3.49 | 3.62 | 5.12 | 7.86E-03 | 4.16 | 1.34E-03 | 851 |
| Cluster-40555.164908 | 0.58 | 0.24 | 10.42 | 13.96 | 1.36 | 0 | 9.74 | 8.52 | 5.12 | 5.39E-09 | 3.81 | 3.28E-05 | 667 |
| Cluster-40555.187626 | 64.83 | 59.7 | 1596.73 | 2013.01 | 172.04 | 180.03 | 1088.2 | 1198.9 | 5.12 | 1.74E-15 | 2.68 | 2.11E-04 | 218 |
| Cluster-40555.192508 | 0.36 | 0.46 | 13.48 | 12.1 | 2.44 | 1.5 | 7.23 | 10.39 | 5.13 | 1.79E-23 | 2.24 | 1.23E-03 | 1503 |
| Cluster-40555.158188 | 0.34 | 0.7 | 16.69 | 16.9 | 4.25 | 2.14 | 14.58 | 20.97 | 5.13 | 2.05E-08 | 2.56 | 5.91E-04 | 545 |
| Cluster-40555.204956 | 0 | 1.7 | 29.46 | 26.91 | 7.75 | 5.64 | 30.8 | 33.37 | 5.13 | 2.04E-27 | 2.33 | 6.02E-09 | 967 |
| Cluster-40555.229507 | 0 | 0.28 | 4.25 | 4.91 | 0.56 | 0.15 | 5.34 | 3.8 | 5.13 | 4.29E-07 | 3.76 | 1.38E-05 | 1125 |
| Cluster-40555.192496 | 0.87 | 0.74 | 28.36 | 23.54 | 16.42 | 8.5 | 31.86 | 38.72 | 5.13 | 6.71E-16 | 1.59 | 1.51E-03 | 640 |
| Cluster-40555.236070 | 1.03 | 0.25 | 20.57 | 18.88 | 1.37 | 1.46 | 14.68 | 18.06 | 5.13 | 6.98E-09 | 3.64 | 1.61E-05 | 516 |
| Cluster-40555.158727 | 0.06 | 0.05 | 1.63 | 1.75 | 0.08 | 0.44 | 3.37 | 2.71 | 5.14 | 3.64E-05 | 3.56 | 1.12E-06 | 1922 |
| Cluster-40555.203601 | 0.41 | 0.95 | 21.29 | 22.76 | 0 | 1.28 | 11.29 | 11.74 | 5.14 | 5.26E-05 | 4.16 | 2.22E-02 | 384 |
| Cluster-40555.189393 | 119.75 | 219.99 | 5358.75 | 5635.77 | 2693.49 | 3299.1 | 9076.54 | 10600.03 | 5.14 | 4.45E-102 | 1.78 | 1.04E-09 | 3159 |
| Cluster-40555.197074 | 0.13 | 0 | 1.93 | 1.79 | 0 | 0 | 0.82 | 1.37 | 5.14 | 7.91E-03 | Inf | 2.32E-02 | 1056 |
| Cluster-40555.124890 | 0.19 | 0 | 2.73 | 3.01 | 0.09 | 0.1 | 4.38 | 3.22 | 5.14 | 7.44E-03 | 5.38 | 3.46E-04 | 772 |
| Cluster-40555.187456 | 0.2 | 0.48 | 12.15 | 10.08 | 1.58 | 1.39 | 12.39 | 11.63 | 5.15 | 1.13E-25 | 3.08 | 3.25E-13 | 1881 |
| Cluster-40555.150746 | 0 | 0.07 | 1.38 | 0.94 | 0 | 0 | 1.93 | 2.14 | 5.15 | 9.27E-05 | Inf | 2.80E-13 | 2576 |
| Cluster-40555.269367 | 0 | 0.11 | 1.89 | 1.69 | 0.06 | 0 | 2.43 | 1.39 | 5.15 | 1.66E-02 | 6.04 | 2.35E-03 | 1013 |
| Cluster-40555.157409 | 0.07 | 0 | 0.97 | 1.16 | 0.18 | 0 | 1.49 | 1.99 | 5.15 | 6.83E-03 | 4.50 | 2.10E-04 | 1656 |
| Cluster-40555.191544 | 0 | 0.63 | 8.42 | 13.14 | 0.41 | 0.43 | 18.67 | 12.68 | 5.15 | 1.39E-02 | 5.28 | 7.44E-04 | 384 |
| Cluster-40555.230298 | 0 | 0.24 | 3.74 | 4.22 | 0.59 | 0.4 | 3.63 | 3.96 | 5.16 | 1.96E-09 | 3.02 | 3.05E-05 | 1614 |
| Cluster-40555.189539 | 7.86 | 11.12 | 268.03 | 348.89 | 66.15 | 68.46 | 221.82 | 267.59 | 5.16 | 4.21E-21 | 1.93 | 8.40E-10 | 726 |
| Cluster-40555.147806 | 0 | 0.23 | 5.14 | 3.38 | 0.25 | 0.16 | 2.28 | 3.99 | 5.17 | 8.07E-05 | 4.15 | 1.98E-03 | 902 |
| Cluster-40555.189499 | 0.58 | 0.03 | 9.86 | 9.12 | 0 | 0.15 | 8.87 | 7.46 | 5.17 | 2.68E-21 | 6.90 | 1.45E-24 | 1688 |
| Cluster-40555.165377 | 0.13 | 0.08 | 2.48 | 3.56 | 0 | 0 | 2.27 | 3.71 | 5.18 | 2.60E-09 | Inf | 2.05E-08 | 1945 |
| Cluster-40555.182604 | 0.24 | 0.72 | 16.72 | 15.63 | 2.45 | 2.61 | 13.47 | 12.73 | 5.18 | 3.20E-36 | 2.44 | 5.91E-10 | 2049 |
| Cluster-40555.145096 | 0.85 | 0 | 10.16 | 16.33 | 0 | 0.51 | 20.75 | 24.84 | 5.18 | 2.41E-07 | 6.44 | 3.24E-30 | 1007 |
| Cluster-40555.214093 | 0.03 | 0.02 | 0.82 | 0.93 | 0.53 | 0.36 | 1.66 | 2.08 | 5.19 | 2.07E-05 | 2.16 | 9.84E-04 | 3584 |
| Cluster-40555.203259 | 1.24 | 0.91 | 38.36 | 32.44 | 0.2 | 0.56 | 39.2 | 33.53 | 5.19 | 4.95E-33 | 6.61 | 4.32E-39 | 965 |
| Cluster-40555.137818 | 0.18 | 0.15 | 5.18 | 5.72 | 0.35 | 0.46 | 4.45 | 6.27 | 5.19 | 2.02E-05 | 3.78 | 2.63E-04 | 796 |

| | | | | | | | | | | | | | |
|---|---|---|---|---|---|---|---|---|---|---|---|---|---|
| Cluster-40555.165699 | 0 | 0.25 | 3.19 | 5.73 | 5.15 | 0.61 | 23.64 | 41.43 | 5.19 | 9.87E-05 | 3.60 | 4.16E-03 | 1033 |
| Cluster-40555.189224 | 10.14 | 13.91 | 404.19 | 401.4 | 14.82 | 23.47 | 307.56 | 377.92 | 5.20 | 7.59E-91 | 4.22 | 1.01E-22 | 1183 |
| Cluster-101106.0 | 0 | 0.19 | 3.26 | 4.27 | 0 | 0 | 2.89 | 2.08 | 5.21 | 6.18E-04 | Inf | 8.09E-04 | 818 |
| Cluster-40555.188821 | 0.15 | 0 | 2.55 | 1.51 | 0.39 | 0.57 | 3.22 | 3.23 | 5.21 | 6.59E-03 | 2.83 | 1.04E-02 | 1032 |
| Cluster-40555.201722 | 1.74 | 0.77 | 36.44 | 46.65 | 1.47 | 1.52 | 13.55 | 14.56 | 5.21 | 4.25E-22 | 3.30 | 7.68E-08 | 783 |
| Cluster-40555.174275 | 0.05 | 0.18 | 3.84 | 4.27 | 0.05 | 0.06 | 1.66 | 2.42 | 5.21 | 1.47E-06 | 5.30 | 6.88E-04 | 1145 |
| Cluster-40555.163604 | 0.12 | 0 | 1.99 | 1.79 | 2.3 | 2.35 | 5.15 | 4.7 | 5.22 | 4.30E-11 | 1.14 | 2.13E-02 | 3565 |
| Cluster-40555.255484 | 0 | 0.13 | 2.24 | 2.15 | 0.15 | 0.36 | 2.16 | 1.84 | 5.22 | 6.61E-04 | 3.03 | 2.24E-02 | 1223 |
| Cluster-40555.189996 | 6.98 | 9.95 | 296.74 | 280.98 | 58.07 | 42.26 | 121.61 | 119.26 | 5.22 | 1.31E-96 | 1.34 | 3.00E-05 | 2534 |
| Cluster-40555.188681 | 27.32 | 42.6 | 1228.6 | 1161.79 | 23.19 | 25.1 | 163.68 | 312.96 | 5.22 | 6.38E-99 | 3.38 | 1.75E-02 | 1068 |
| Cluster-40555.127815 | 0.14 | 0 | 2.4 | 2.16 | 0 | 0.24 | 3.16 | 3.65 | 5.23 | 6.35E-10 | 4.83 | 3.59E-14 | 2682 |
| Cluster-40555.207566 | 0.14 | 0 | 3.2 | 2.08 | 0 | 0 | 6.01 | 5.38 | 5.23 | 5.38E-03 | Inf | 1.17E-09 | 864 |
| Cluster-40555.148368 | 0.29 | 0.42 | 12.27 | 12.33 | 0.82 | 0.99 | 4.91 | 5.84 | 5.24 | 6.15E-14 | 2.62 | 2.97E-03 | 924 |
| Cluster-40555.221356 | 0.07 | 0 | 1.32 | 1.42 | 0.67 | 0.61 | 2.83 | 2.78 | 5.24 | 4.47E-03 | 2.20 | 2.19E-02 | 1420 |
| Cluster-40555.112154 | 0 | 0.31 | 6.45 | 4.46 | 0.28 | 0.19 | 5.31 | 6.64 | 5.24 | 3.89E-05 | 4.77 | 7.01E-06 | 782 |
| Cluster-40555.177181 | 0.59 | 0.7 | 20.8 | 23.54 | 3.88 | 2.34 | 17.11 | 16.01 | 5.24 | 2.07E-20 | 2.49 | 4.11E-06 | 833 |
| Cluster-40555.164859 | 0.06 | 0 | 0.61 | 1.23 | 0 | 0 | 0.58 | 0.79 | 5.24 | 3.52E-03 | Inf | 3.03E-03 | 1959 |
| Cluster-40555.178258 | 0.08 | 0.03 | 1.44 | 2.14 | 0.55 | 0.12 | 5.55 | 4.21 | 5.24 | 1.89E-04 | 3.98 | 9.17E-09 | 1540 |
| Cluster-40555.185673 | 3.53 | 0.34 | 54.62 | 72.98 | 0.2 | 7.15 | 35.91 | 23.47 | 5.24 | 1.42E-17 | 3.02 | 6.06E-05 | 728 |
| Cluster-40555.171665 | 0.48 | 0.36 | 16.25 | 13.44 | 0.68 | 0.51 | 7.84 | 6.03 | 5.25 | 6.86E-26 | 3.63 | 1.03E-09 | 1440 |
| Cluster-40555.256721 | 0.16 | 0 | 1.99 | 2.65 | 0 | 0.29 | 3.17 | 1.86 | 5.25 | 1.23E-04 | 4.13 | 1.91E-04 | 1288 |
| Cluster-40555.109371 | 0.07 | 0 | 0.68 | 1.55 | 0.15 | 0 | 2.78 | 2.58 | 5.25 | 7.93E-03 | 5.45 | 6.07E-08 | 1669 |
| Cluster-40555.164787 | 0 | 1.61 | 36.78 | 23.92 | 0 | 0 | 18.03 | 15.01 | 5.25 | 1.14E-11 | Inf | 7.61E-14 | 571 |
| Cluster-40555.166750 | 0 | 0.38 | 6.57 | 6.67 | 0.53 | 0 | 2.65 | 2.35 | 5.26 | 3.41E-10 | 3.33 | 8.05E-03 | 1136 |
| Cluster-40555.188230 | 2.4 | 3.01 | 86.05 | 103.08 | 20.9 | 23.3 | 111.49 | 88.85 | 5.27 | 1.01E-38 | 2.24 | 9.39E-13 | 1207 |
| Cluster-40555.188273 | 17.19 | 19.22 | 643.73 | 635.87 | 381.98 | 326.55 | 743.72 | 710.97 | 5.27 | 4.20E-101 | 1.11 | 7.38E-04 | 1839 |
| Cluster-40555.180396 | 0.28 | 0.13 | 7.51 | 7.82 | 0.45 | 0.48 | 4 | 4.04 | 5.27 | 3.45E-11 | 3.15 | 7.46E-04 | 1076 |
| Cluster-40555.185366 | 0.64 | 0.29 | 17.88 | 14.62 | 15.14 | 9.63 | 27.61 | 32.15 | 5.28 | 3.51E-21 | 1.35 | 1.54E-03 | 1085 |
| Cluster-40555.188763 | 21.36 | 52.64 | 1284.79 | 1369.57 | 171.73 | 178.18 | 502.23 | 644.53 | 5.28 | 3.78E-99 | 1.78 | 1.46E-05 | 821 |
| Cluster-40555.205017 | 0.28 | 0.32 | 12.03 | 9.62 | 2.89 | 2.17 | 8.64 | 9.1 | 5.28 | 4.63E-31 | 1.88 | 1.14E-05 | 2331 |
| Cluster-40555.200516 | 0.27 | 0 | 5.27 | 4.41 | 1.02 | 0.02 | 2.6 | 2.49 | 5.28 | 1.17E-16 | 2.38 | 1.25E-03 | 2301 |
| Cluster-40555.188135 | 35.3 | 25.54 | 872.14 | 1092.95 | 260.82 | 233.96 | 889.34 | 682.68 | 5.29 | 2.56E-23 | 1.66 | 5.05E-04 | 260 |

| | | | | | | | | | | | | | |
|---|---|---|---|---|---|---|---|---|---|---|---|---|---|
| Cluster-40555.153848 | 0 | 0.02 | 0.27 | 0.53 | 0.55 | 0 | 1.15 | 1.12 | 5.29 | 6.48E-03 | 2.17 | 1.14E-02 | 3917 |
| Cluster-40555.209515 | 1.71 | 2.16 | 69.56 | 68.6 | 0.82 | 0.59 | 7.74 | 9.46 | 5.29 | 4.42E-62 | 3.69 | 7.09E-11 | 1346 |
| Cluster-40555.198545 | 1.06 | 5.29 | 110.89 | 121.69 | 11.44 | 12.44 | 170.55 | 117.26 | 5.29 | 7.09E-63 | 3.64 | 1.66E-11 | 932 |
| Cluster-40555.239487 | 0.1 | 0.08 | 3.72 | 3.64 | 1.15 | 0.77 | 6.33 | 7.3 | 5.29 | 1.41E-08 | 2.91 | 2.34E-07 | 1534 |
| Cluster-40555.188153 | 519.01 | 1069.78 | 29411.4 | 28186.87 | 9426.91 | 9109.86 | 18716.4 | 19198.65 | 5.30 | 2.24E-106 | 1.10 | 7.38E-04 | 918 |
| Cluster-40555.188651 | 0 | 0.14 | 1.91 | 3.28 | 0 | 0 | 3.71 | 1.39 | 5.31 | 3.26E-05 | Inf | 1.84E-03 | 1699 |
| Cluster-40555.178219 | 0.02 | 0.02 | 0.7 | 0.72 | 0 | 0 | 1.13 | 0.65 | 5.31 | 4.49E-03 | Inf | 1.13E-05 | 2505 |
| Cluster-40555.201309 | 0.15 | 0.08 | 4.14 | 3.51 | 0.37 | 0.44 | 3.21 | 3.82 | 5.31 | 2.01E-11 | 3.16 | 2.40E-06 | 1950 |
| Cluster-40555.221833 | 0.13 | 0 | 2.38 | 2.18 | 0.04 | 0.17 | 1.24 | 1.31 | 5.31 | 3.00E-09 | 3.57 | 4.77E-04 | 2463 |
| Cluster-40555.224017 | 0 | 1.07 | 22.63 | 18.44 | 1.71 | 0 | 16.72 | 15.21 | 5.31 | 1.62E-05 | 4.33 | 8.29E-04 | 407 |
| Cluster-40555.204676 | 0 | 0.05 | 1.13 | 1.02 | 0 | 0 | 0.78 | 0.7 | 5.32 | 2.14E-09 | Inf | 2.29E-10 | 5173 |
| Cluster-40555.181561 | 0.5 | 0.17 | 11.36 | 11.98 | 2.38 | 2.23 | 6.41 | 9.11 | 5.32 | 6.89E-35 | 1.82 | 4.53E-03 | 2425 |
| Cluster-40555.210194 | 0.46 | 0 | 8.73 | 6.68 | 0.78 | 0.8 | 7.16 | 9.94 | 5.32 | 3.42E-09 | 3.46 | 9.04E-07 | 915 |
| Cluster-40555.179885 | 1.2 | 1.23 | 44.68 | 41.09 | 8.01 | 13.52 | 54.32 | 65.22 | 5.32 | 1.18E-30 | 2.52 | 1.16E-11 | 761 |
| Cluster-40555.200953 | 0 | 0.24 | 4.92 | 4 | 1.34 | 0.74 | 3.36 | 5.01 | 5.33 | 7.97E-12 | 2.10 | 1.04E-02 | 1796 |
| Cluster-40555.177988 | 0.12 | 0.11 | 3.47 | 4.62 | 0.77 | 0.32 | 3.04 | 4.71 | 5.33 | 5.39E-09 | 2.89 | 1.51E-03 | 1452 |
| Cluster-40555.183404 | 0.29 | 1.09 | 26.26 | 25.69 | 15.96 | 8.01 | 41.31 | 31.32 | 5.34 | 5.23E-26 | 1.68 | 5.38E-05 | 923 |
| Cluster-40555.165086 | 0.15 | 0.17 | 6.62 | 5.45 | 0.07 | 0.03 | 6.09 | 7.81 | 5.34 | 1.42E-18 | 7.20 | 5.91E-23 | 2135 |
| Cluster-40555.143078 | 0.27 | 0.21 | 10.95 | 6.65 | 3.2 | 3.34 | 16.48 | 21.71 | 5.35 | 4.57E-03 | 2.60 | 4.63E-03 | 446 |
| Cluster-40555.203510 | 0.66 | 0.5 | 21.9 | 20.41 | 0.07 | 0.04 | 6.32 | 6.46 | 5.35 | 3.79E-42 | 7.26 | 5.66E-23 | 1880 |
| Cluster-40555.204486 | 0.06 | 0.14 | 4.22 | 3.72 | 0.48 | 0.44 | 3.5 | 2.86 | 5.35 | 3.07E-07 | 2.86 | 2.44E-03 | 1256 |
| Cluster-40555.137140 | 0 | 0.03 | 0.66 | 0.57 | 0.05 | 0.13 | 1.53 | 1.57 | 5.35 | 6.05E-03 | 4.19 | 2.40E-06 | 2734 |
| Cluster-40555.196586 | 0.08 | 0.48 | 10.44 | 11.51 | 2.18 | 2.51 | 12.39 | 11.09 | 5.35 | 2.58E-26 | 2.38 | 1.41E-08 | 1835 |
| Cluster-40555.201455 | 0.21 | 0.89 | 24.34 | 18.23 | 0.91 | 0.78 | 6.63 | 9.49 | 5.36 | 5.48E-26 | 3.32 | 4.55E-06 | 1083 |
| Cluster-40555.194912 | 0.76 | 0.55 | 27.06 | 22.06 | 0.3 | 0 | 9.25 | 7.86 | 5.36 | 1.02E-45 | 6.00 | 1.68E-24 | 1874 |
| Cluster-40555.151229 | 0 | 0.29 | 5.16 | 5.96 | 1.42 | 0.99 | 6.37 | 6.76 | 5.36 | 6.21E-16 | 2.52 | 6.09E-07 | 1937 |
| Cluster-40555.186317 | 0.93 | 0.72 | 31.88 | 30.92 | 6.98 | 5.11 | 36.16 | 31.01 | 5.36 | 4.56E-35 | 2.54 | 2.99E-11 | 1074 |
| Cluster-40555.238941 | 0.08 | 0 | 1.33 | 1.6 | 0.34 | 0.41 | 2.67 | 2.42 | 5.36 | 8.97E-10 | 2.81 | 2.35E-07 | 3810 |
| Cluster-40555.161542 | 0.28 | 0.03 | 6 | 5.81 | 0 | 0.04 | 2.45 | 2.21 | 5.36 | 5.59E-20 | 7.61 | 2.87E-12 | 2287 |
| Cluster-40555.183511 | 4.93 | 4.93 | 189.83 | 178.35 | 68.59 | 89.93 | 148.17 | 170.37 | 5.36 | 3.25E-63 | 1.06 | 4.23E-03 | 670 |
| Cluster-40555.155912 | 0.16 | 0.65 | 16.55 | 14.72 | 0.32 | 0.5 | 8.97 | 11.15 | 5.37 | 1.13E-08 | 4.68 | 1.21E-05 | 566 |
| Cluster-40555.176809 | 0 | 0.09 | 1.43 | 1.75 | 0.53 | 0.59 | 2.89 | 3.03 | 5.37 | 1.72E-04 | 2.45 | 1.56E-03 | 1746 |

| | | | | | | | | | | | | | |
|---|---|---|---|---|---|---|---|---|---|---|---|---|---|
| Cluster-40555.222380 | 1.74 | 0.22 | 25.92 | 44.08 | 0.74 | 0.69 | 4.79 | 9.09 | 5.37 | 1.46E-06 | 3.31 | 2.48E-02 | 626 |
| Cluster-40555.84995 | 0.07 | 0.03 | 2.19 | 1.36 | 2.15 | 0.62 | 4.75 | 3.9 | 5.38 | 9.59E-05 | 1.74 | 1.29E-02 | 1695 |
| Cluster-40555.202651 | 0.59 | 0.51 | 19.26 | 21.4 | 17.05 | 19.79 | 33.48 | 40.01 | 5.38 | 1.58E-38 | 1.06 | 5.90E-03 | 1686 |
| Cluster-40555.220324 | 0 | 0.06 | 1.83 | 1.27 | 0.09 | 0.05 | 1.47 | 1.93 | 5.38 | 5.58E-03 | 4.70 | 2.12E-03 | 1287 |
| Cluster-40555.138252 | 0.11 | 0 | 2.05 | 2.1 | 0.76 | 0.3 | 3.04 | 5.69 | 5.38 | 1.83E-03 | 3.13 | 3.07E-02 | 1105 |
| Cluster-40555.223117 | 0.13 | 0.04 | 3.38 | 2.56 | 0.12 | 0.16 | 3.74 | 5.3 | 5.39 | 6.59E-21 | 5.10 | 7.04E-11 | 4596 |
| Cluster-40555.185558 | 21.14 | 24.5 | 823.22 | 900.62 | 166.76 | 162.47 | 678.53 | 715.69 | 5.39 | 1.75E-75 | 2.13 | 1.93E-11 | 398 |
| Cluster-40555.183523 | 0.15 | 0.26 | 7.64 | 8.04 | 0.44 | 0.42 | 6 | 7.28 | 5.39 | 3.49E-13 | 4.01 | 5.38E-09 | 1204 |
| Cluster-40555.191723 | 0.43 | 0.75 | 24.45 | 21.1 | 2.7 | 2.38 | 20.71 | 20.08 | 5.39 | 5.88E-43 | 3.08 | 1.93E-16 | 1818 |
| Cluster-40555.133724 | 0.11 | 0.09 | 3.54 | 3.87 | 0 | 0.22 | 3.96 | 3.99 | 5.39 | 2.76E-03 | 5.18 | 8.21E-04 | 713 |
| Cluster-40555.189548 | 1.57 | 1.37 | 58.52 | 51.88 | 11.47 | 8.67 | 43.38 | 39.8 | 5.39 | 3.33E-41 | 2.11 | 7.29E-08 | 854 |
| Cluster-40555.188194 | 0.7 | 1.34 | 38.02 | 40.76 | 11.88 | 9.36 | 32.23 | 35.4 | 5.40 | 9.52E-63 | 1.74 | 2.36E-07 | 2099 |
| Cluster-40555.224163 | 0.2 | 0.12 | 6.21 | 5.27 | 3.33 | 2.67 | 6.68 | 9.34 | 5.40 | 2.47E-17 | 1.50 | 1.41E-02 | 2043 |
| Cluster-40555.204767 | 0.1 | 0 | 1.47 | 2.53 | 0.12 | 0.12 | 2.11 | 1.82 | 5.40 | 1.73E-05 | 4.07 | 1.21E-05 | 2034 |
| Cluster-40555.235445 | 0.04 | 0 | 0.87 | 0.66 | 0.21 | 0.14 | 2.18 | 1.1 | 5.41 | 1.80E-03 | 3.25 | 8.56E-03 | 2622 |
| Cluster-40555.242434 | 0 | 0.43 | 7.26 | 9.88 | 0.13 | 0 | 6.42 | 7.28 | 5.41 | 3.81E-06 | 6.86 | 2.60E-06 | 635 |
| Cluster-40555.192300 | 0.12 | 0 | 2.62 | 1.8 | 0.23 | 0.17 | 4.81 | 6.13 | 5.41 | 4.09E-05 | 4.88 | 8.32E-12 | 1476 |
| Cluster-40555.189900 | 0 | 0.18 | 3.46 | 3.93 | 0.26 | 0.21 | 3.56 | 2.97 | 5.41 | 3.78E-14 | 3.89 | 1.42E-09 | 2434 |
| Cluster-40555.208425 | 0 | 0.21 | 4.44 | 3.69 | 0 | 0.13 | 3.17 | 3.79 | 5.41 | 1.04E-12 | 5.68 | 4.42E-13 | 2047 |
| Cluster-40555.128671 | 0.18 | 0.12 | 6.78 | 4.18 | 0.49 | 0.15 | 2.88 | 2.84 | 5.42 | 6.77E-08 | 3.35 | 5.74E-03 | 1051 |
| Cluster-40555.171962 | 0.23 | 0.04 | 5.07 | 5.07 | 1.33 | 1.29 | 7.24 | 6.83 | 5.42 | 6.38E-13 | 2.49 | 2.07E-06 | 1662 |
| Cluster-40555.190290 | 0.17 | 0.38 | 10.05 | 11.04 | 0 | 0 | 2.86 | 3.32 | 5.42 | 1.00E-20 | Inf | 2.16E-10 | 1445 |
| Cluster-40555.163171 | 0.24 | 0 | 4.95 | 4.23 | 0.05 | 0.15 | 6.37 | 5.12 | 5.42 | 1.68E-08 | 5.90 | 2.54E-12 | 1246 |
| Cluster-40555.194294 | 0 | 0.16 | 4.02 | 2.44 | 3.12 | 1.94 | 4.86 | 5.84 | 5.42 | 6.59E-09 | 1.16 | 4.41E-02 | 2538 |
| Cluster-40555.167869 | 0 | 1.12 | 19.67 | 26.21 | 2.16 | 0.21 | 17.26 | 10.83 | 5.43 | 1.68E-10 | 3.67 | 1.16E-04 | 508 |
| Cluster-40555.192486 | 0.33 | 0.15 | 10.56 | 8.18 | 0.33 | 0.24 | 8.82 | 9.19 | 5.43 | 1.36E-32 | 5.02 | 1.81E-27 | 2699 |
| Cluster-40555.164634 | 0.15 | 0.24 | 8.28 | 7.07 | 4.76 | 2.84 | 12.2 | 16.21 | 5.43 | 2.48E-09 | 1.98 | 3.07E-04 | 924 |
| Cluster-40555.223527 | 0.94 | 0 | 18.09 | 15.31 | 2.03 | 3.08 | 20.03 | 23.4 | 5.43 | 1.46E-03 | 3.20 | 9.58E-03 | 368 |
| Cluster-40555.164109 | 0.05 | 0 | 0.81 | 1.04 | 0.08 | 0.08 | 1.93 | 3.2 | 5.43 | 1.21E-03 | 5.19 | 2.97E-05 | 2202 |
| Cluster-40555.192066 | 0.58 | 0 | 11.55 | 11.14 | 0.31 | 0 | 11.54 | 14.29 | 5.44 | 2.46E-05 | 6.11 | 6.21E-07 | 502 |
| Cluster-40555.189168 | 3.85 | 4.75 | 197.08 | 145.3 | 37.4 | 16.86 | 148.63 | 139.76 | 5.44 | 2.22E-31 | 2.49 | 2.85E-16 | 1544 |
| Cluster-40555.229615 | 0.18 | 0 | 2.92 | 3.99 | 0 | 0 | 1.74 | 1.3 | 5.44 | 1.11E-03 | Inf | 4.02E-02 | 789 |

| | | | | | | | | | | | | | |
|---|---|---|---|---|---|---|---|---|---|---|---|---|---|
| Cluster-40555.212863 | 0.05 | 0.02 | 1.52 | 1.39 | 0.93 | 0.89 | 2.49 | 2.3 | 5.44 | 2.98E-08 | 1.45 | 2.67E-02 | 3284 |
| Cluster-40555.191585 | 0.07 | 0 | 1.1 | 1.33 | 0.07 | 0.11 | 1.1 | 0.72 | 5.45 | 3.59E-07 | 3.42 | 9.50E-04 | 3293 |
| Cluster-40555.243735 | 0 | 0.12 | 2.45 | 2.61 | 0 | 0 | 3.94 | 4.17 | 5.45 | 2.74E-06 | Inf | 1.13E-14 | 1561 |
| Cluster-40555.199082 | 0.18 | 0 | 4.81 | 2.61 | 0.43 | 0.28 | 3.77 | 2.7 | 5.46 | 1.52E-03 | 3.31 | 3.46E-02 | 759 |
| Cluster-40555.201023 | 0 | 0.06 | 1.53 | 0.7 | 0.33 | 0.14 | 1.24 | 2.13 | 5.46 | 1.78E-03 | 2.98 | 1.47E-02 | 2526 |
| Cluster-40555.179630 | 0.09 | 0.38 | 10.11 | 9.51 | 0.35 | 0.65 | 10.41 | 9.35 | 5.46 | 4.66E-21 | 4.35 | 5.18E-17 | 1564 |
| Cluster-40555.204207 | 0.36 | 0 | 7.03 | 6.81 | 1.41 | 0.85 | 6.37 | 7.38 | 5.46 | 1.58E-25 | 2.69 | 1.90E-09 | 2539 |
| Cluster-40555.185719 | 0 | 0.21 | 4.98 | 3.98 | 1.42 | 1.27 | 7.32 | 8.77 | 5.47 | 3.13E-03 | 2.63 | 7.93E-03 | 637 |
| Cluster-40555.191377 | 0.58 | 2.73 | 66.02 | 71.78 | 2.76 | 7.11 | 41.74 | 45.44 | 5.48 | 8.88E-53 | 3.18 | 2.11E-17 | 970 |
| Cluster-40555.214323 | 0 | 0.07 | 1.99 | 1.42 | 0.1 | 0.56 | 1.73 | 1.97 | 5.48 | 9.23E-08 | 2.51 | 6.74E-04 | 2747 |
| Cluster-40555.165040 | 0.31 | 0.06 | 6.26 | 8.8 | 0.05 | 0.09 | 6.85 | 5.79 | 5.48 | 1.43E-12 | 6.62 | 7.54E-16 | 1358 |
| Cluster-40555.125019 | 0.12 | 0.05 | 3.15 | 3.8 | 0.3 | 0.19 | 3.24 | 3.13 | 5.48 | 2.25E-05 | 3.78 | 9.01E-04 | 1044 |
| Cluster-40555.173009 | 0.33 | 0.18 | 10.49 | 9.62 | 2.03 | 0.82 | 10.61 | 10.47 | 5.49 | 4.04E-28 | 2.97 | 3.92E-12 | 2064 |
| Cluster-40555.216438 | 0.13 | 0.23 | 8.17 | 7 | 0.21 | 0.19 | 5.3 | 4.2 | 5.49 | 1.01E-21 | 4.60 | 3.41E-13 | 2006 |
| Cluster-40555.205568 | 1.53 | 0.79 | 50.35 | 42.89 | 0.55 | 0.72 | 16.71 | 18.01 | 5.49 | 6.74E-26 | 4.84 | 1.04E-10 | 612 |
| Cluster-40555.204239 | 0.28 | 0.78 | 22.81 | 23.19 | 3.08 | 2.46 | 17.65 | 12.49 | 5.50 | 1.88E-19 | 2.50 | 2.78E-05 | 757 |
| Cluster-40555.202906 | 0.15 | 0.12 | 4.76 | 6.03 | 19.15 | 18.08 | 48.42 | 32.98 | 5.51 | 1.22E-03 | 1.19 | 2.43E-02 | 597 |
| Cluster-97046.0 | 0.09 | 0.08 | 2.84 | 4.12 | 0.18 | 0.38 | 3.43 | 2.13 | 5.51 | 1.15E-03 | 3.34 | 4.73E-02 | 782 |
| Cluster-40555.203326 | 0 | 0.38 | 8.49 | 7.94 | 0.23 | 0 | 8.71 | 6.05 | 5.51 | 2.27E-03 | 6.09 | 1.69E-03 | 471 |
| Cluster-40555.188159 | 3.57 | 3.33 | 146.49 | 139.72 | 308.92 | 289 | 499.74 | 516.96 | 5.51 | 3.46E-81 | 0.83 | 2.70E-02 | 1234 |
| Cluster-40555.195227 | 0.07 | 0.06 | 2.2 | 2.38 | 0.34 | 0.36 | 3.01 | 3.18 | 5.52 | 2.39E-10 | 3.21 | 1.04E-07 | 2655 |
| Cluster-40555.186288 | 0.14 | 0.1 | 5.52 | 6.1 | 0.62 | 0.74 | 4.98 | 4.65 | 5.52 | 4.33E-07 | 2.95 | 2.44E-03 | 890 |
| Cluster-40555.169168 | 0.06 | 0.07 | 3.42 | 2.51 | 0.78 | 0.58 | 2.15 | 2.99 | 5.52 | 1.84E-08 | 1.99 | 2.22E-02 | 1794 |
| Cluster-40555.217206 | 0.18 | 0 | 5.15 | 2.06 | 0.82 | 0.65 | 10.45 | 6.21 | 5.52 | 6.10E-03 | 3.55 | 2.44E-05 | 1610 |
| Cluster-40555.166460 | 0.71 | 0 | 10.26 | 17.34 | 0.48 | 0.32 | 9.39 | 16.79 | 5.52 | 7.11E-06 | 5.26 | 2.93E-04 | 469 |
| Cluster-40555.170343 | 0.39 | 0.67 | 23.26 | 21.6 | 3.33 | 1.16 | 25.72 | 21.5 | 5.52 | 1.91E-45 | 3.48 | 3.62E-21 | 1911 |
| Cluster-40555.142847 | 0.11 | 0 | 2.29 | 2.21 | 1.72 | 2.07 | 5.95 | 5.65 | 5.53 | 7.19E-04 | 1.68 | 2.88E-02 | 1130 |
| Cluster-40555.214598 | 0 | 0.17 | 3.61 | 3.94 | 0.1 | 0 | 2.13 | 1.9 | 5.53 | 1.90E-03 | 5.41 | 4.93E-02 | 728 |
| Cluster-40555.224560 | 0.37 | 0.15 | 12.35 | 9.18 | 8.43 | 11.51 | 34.18 | 32.34 | 5.54 | 1.77E-05 | 1.79 | 1.59E-03 | 525 |
| Cluster-40555.187886 | 57.03 | 26.68 | 1382.86 | 1969.04 | 458.51 | 244.57 | 2698.51 | 2744.73 | 5.54 | 1.40E-14 | 3.01 | 3.82E-23 | 347 |
| Cluster-40555.167785 | 0.16 | 0 | 3.13 | 3.49 | 0.16 | 0 | 2.16 | 2.21 | 5.54 | 6.00E-04 | 4.89 | 1.36E-02 | 851 |
| Cluster-96786.0 | 0.08 | 0 | 2.59 | 0.94 | 0 | 0 | 1.45 | 0.57 | 5.55 | 2.36E-02 | Inf | 1.60E-02 | 1381 |

| | | | | | | | | | | | | | |
|---|---|---|---|---|---|---|---|---|---|---|---|---|---|
| Cluster-40555.205745 | 0.13 | 0 | 3.16 | 2.05 | 0.46 | 0.67 | 2.12 | 2.22 | 5.55 | 3.03E-09 | 1.99 | 1.63E-02 | 2153 |
| Cluster-40555.159876 | 0.22 | 0 | 5.53 | 3.18 | 0.92 | 1.56 | 7.7 | 4.95 | 5.55 | 6.94E-08 | 2.40 | 1.60E-04 | 2303 |
| Cluster-40555.170314 | 0 | 0.19 | 4.23 | 4.25 | 0 | 0.48 | 3.89 | 3.1 | 5.55 | 3.75E-05 | 3.89 | 1.43E-03 | 887 |
| Cluster-40555.273653 | 0 | 0.04 | 0.76 | 1.23 | 0 | 0 | 1.55 | 1.65 | 5.55 | 2.77E-05 | Inf | 4.67E-12 | 2912 |
| Cluster-40555.223423 | 0.03 | 0 | 0.59 | 0.76 | 0 | 0 | 0.55 | 0.87 | 5.56 | 4.58E-02 | Inf | 7.08E-03 | 1739 |
| Cluster-40555.194420 | 0.46 | 0 | 8.19 | 12.65 | 0.14 | 0 | 3.4 | 5.21 | 5.56 | 7.96E-08 | 6.21 | 8.35E-04 | 640 |
| Cluster-40555.133345 | 0 | 0.05 | 0.81 | 1.08 | 0 | 0 | 0.48 | 0.61 | 5.56 | 1.42E-03 | Inf | 8.54E-03 | 2140 |
| Cluster-40555.220544 | 0.14 | 0 | 3.01 | 2.74 | 0.28 | 0.43 | 5.59 | 4.14 | 5.57 | 4.73E-02 | 3.83 | 1.07E-02 | 609 |
| Cluster-40555.165674 | 0.93 | 0.54 | 35.07 | 28.15 | 7.95 | 7.09 | 35.99 | 37.83 | 5.58 | 5.67E-60 | 2.36 | 3.32E-13 | 2186 |
| Cluster-40555.137283 | 0.37 | 0 | 7.55 | 8.04 | 0.36 | 0.15 | 3.51 | 4.69 | 5.58 | 8.87E-10 | 4.08 | 2.51E-04 | 908 |
| Cluster-40555.122688 | 0.06 | 0 | 1 | 1.04 | 0 | 0.14 | 1.35 | 1.13 | 5.59 | 4.33E-02 | 4.10 | 2.91E-02 | 1265 |
| Cluster-40555.242432 | 0.13 | 0 | 1.97 | 3.38 | 0 | 0.27 | 3.69 | 1.99 | 5.59 | 3.85E-02 | 4.42 | 4.91E-02 | 637 |
| Cluster-40555.177703 | 0.42 | 4.24 | 106.51 | 103.35 | 8.71 | 8.56 | 143.78 | 146.11 | 5.59 | 4.03E-69 | 4.13 | 8.60E-37 | 1075 |
| Cluster-40555.194186 | 5.73 | 1.58 | 155.99 | 138.82 | 21.64 | 14.02 | 71.92 | 94.09 | 5.59 | 5.42E-19 | 2.25 | 1.69E-03 | 330 |
| Cluster-40555.162986 | 0.16 | 0 | 3.22 | 4.08 | 0 | 0 | 2.2 | 2.1 | 5.59 | 3.85E-04 | Inf | 3.12E-03 | 817 |
| Cluster-40555.177604 | 2.43 | 3.03 | 111.38 | 128.87 | 16.66 | 13.67 | 222.24 | 206.33 | 5.60 | 8.25E-53 | 3.89 | 5.02E-31 | 642 |
| Cluster-79400.0 | 0.1 | 0 | 2.33 | 1.74 | 0 | 0 | 2.23 | 0.95 | 5.60 | 4.48E-02 | Inf | 4.13E-02 | 764 |
| Cluster-40555.154682 | 0.4 | 0.33 | 16.75 | 15.41 | 0 | 0.1 | 5.09 | 4.39 | 5.60 | 3.50E-14 | 6.53 | 1.34E-05 | 743 |
| Cluster-40555.178345 | 0.13 | 0 | 2.73 | 2.59 | 0 | 0 | 2.95 | 3.9 | 5.60 | 4.21E-02 | Inf | 1.52E-03 | 644 |
| Cluster-40555.240476 | 0 | 0.1 | 2.58 | 1.47 | 0 | 0 | 1.56 | 1.23 | 5.60 | 1.59E-03 | Inf | 1.59E-03 | 1182 |
| Cluster-40555.180737 | 6.58 | 5.83 | 291.94 | 255.38 | 0.34 | 0.36 | 15.1 | 22.99 | 5.60 | 3.43E-67 | 5.83 | 7.21E-09 | 544 |
| Cluster-40555.230462 | 0.09 | 0.23 | 6.81 | 7.99 | 0.1 | 0.12 | 4.33 | 4.68 | 5.61 | 4.47E-09 | 5.34 | 4.17E-06 | 896 |
| Cluster-40555.136066 | 0.19 | 0.46 | 19.09 | 10.28 | 3.99 | 4.66 | 19.66 | 21.85 | 5.61 | 1.71E-05 | 2.33 | 1.01E-03 | 521 |
| Cluster-40555.190462 | 0.52 | 0.29 | 17.06 | 18.35 | 13.3 | 14.45 | 24.91 | 29.24 | 5.61 | 6.67E-41 | 1.03 | 1.01E-02 | 1932 |
| Cluster-40555.194928 | 0.68 | 2.35 | 70.74 | 66.96 | 0.22 | 0 | 39.83 | 45.39 | 5.61 | 4.03E-43 | 8.87 | 9.25E-40 | 742 |
| Cluster-40555.30952 | 0.06 | 0.03 | 2.21 | 1.63 | 0.25 | 0.31 | 2.65 | 1.26 | 5.61 | 8.03E-06 | 2.87 | 4.90E-02 | 1824 |
| Cluster-40555.206443 | 0.09 | 0 | 1.62 | 2.22 | 0 | 0 | 2.36 | 2.41 | 5.62 | 3.12E-04 | Inf | 2.86E-07 | 1339 |
| Cluster-40555.216207 | 0 | 0.21 | 4.75 | 5.3 | 0.45 | 0.24 | 3.02 | 1.72 | 5.62 | 1.57E-10 | 2.86 | 8.03E-03 | 1371 |
| Cluster-40555.187227 | 0.78 | 0.7 | 33.93 | 31.84 | 0.77 | 0.95 | 22.35 | 20.39 | 5.62 | 8.50E-66 | 4.68 | 2.24E-35 | 2467 |
| Cluster-40555.220849 | 0.7 | 0.78 | 33.13 | 38.58 | 22.58 | 16.18 | 74.09 | 70.03 | 5.62 | 2.08E-09 | 1.95 | 4.42E-04 | 395 |
| Cluster-40555.192511 | 0.26 | 0 | 6.38 | 5.42 | 0.15 | 0.41 | 3.72 | 5.76 | 5.62 | 4.26E-08 | 4.14 | 1.65E-05 | 964 |
| Cluster-40555.164437 | 0.4 | 0.25 | 12.43 | 15.6 | 3.04 | 0.45 | 16.12 | 8.78 | 5.63 | 7.72E-24 | 2.93 | 1.03E-02 | 1252 |

| | | | | | | | | | | | | | |
|---|---|---|---|---|---|---|---|---|---|---|---|---|---|
| Cluster-40555.187096 | 0.15 | 0.13 | 8.26 | 4.43 | 0 | 0 | 2.44 | 1.76 | 5.63 | 1.06E-05 | Inf | 1.35E-03 | 888 |
| Cluster-40555.206352 | 0.27 | 0 | 6.92 | 4.57 | 0.52 | 0.82 | 5.49 | 4.29 | 5.63 | 3.92E-04 | 2.90 | 4.59E-02 | 626 |
| Cluster-40555.185582 | 0.22 | 0 | 5.29 | 4.5 | 0 | 0 | 4.21 | 4.15 | 5.63 | 1.01E-21 | Inf | 5.50E-27 | 2827 |
| Cluster-40555.206690 | 0 | 0.3 | 7.02 | 7.05 | 0.49 | 0.79 | 4.37 | 3.79 | 5.63 | 2.06E-31 | 2.73 | 1.55E-08 | 3202 |
| Cluster-40555.235001 | 0.25 | 0 | 5.66 | 5.19 | 0 | 0 | 1.29 | 2.3 | 5.64 | 2.99E-06 | Inf | 8.35E-03 | 843 |
| Cluster-40555.184623 | 2.02 | 2 | 94.99 | 86.59 | 50.57 | 46.19 | 153.26 | 148.52 | 5.64 | 1.14E-57 | 1.71 | 1.35E-07 | 861 |
| Cluster-40555.138622 | 0 | 0.17 | 3.95 | 4.38 | 0.1 | 0.1 | 2.96 | 1.65 | 5.64 | 4.77E-09 | 4.81 | 4.15E-05 | 1389 |
| Cluster-40555.222576 | 0 | 0.07 | 1.78 | 1.57 | 0 | 0 | 2.64 | 2.97 | 5.64 | 1.58E-05 | Inf | 2.04E-13 | 1961 |
| Cluster-40555.147481 | 0 | 0.19 | 4.61 | 4.44 | 0 | 0 | 2.66 | 3.43 | 5.64 | 1.48E-05 | Inf | 2.39E-05 | 888 |
| Cluster-40555.209572 | 0.05 | 0.04 | 1.89 | 2.28 | 0.17 | 0.1 | 0.94 | 1.23 | 5.65 | 6.30E-08 | 3.09 | 1.47E-02 | 2185 |
| Cluster-40555.193813 | 0.12 | 0.03 | 2.92 | 3.07 | 0 | 0.04 | 2.6 | 2.18 | 5.66 | 4.18E-10 | 7.41 | 1.11E-10 | 1993 |
| Cluster-40555.109355 | 0.2 | 0 | 4.98 | 3.54 | 0.09 | 0.42 | 3.69 | 4.55 | 5.66 | 3.06E-08 | 4.12 | 1.13E-06 | 1262 |
| Cluster-40555.220354 | 0 | 0.29 | 5.97 | 6.74 | 1.02 | 0.65 | 8.73 | 6.57 | 5.66 | 3.31E-09 | 3.27 | 2.17E-06 | 1011 |
| Cluster-40555.190390 | 1.36 | 1.22 | 64.21 | 54.11 | 53.21 | 41.94 | 81.14 | 91.82 | 5.66 | 1.99E-80 | 0.94 | 1.18E-02 | 2345 |
| Cluster-40555.216086 | 0.23 | 0.29 | 12.43 | 13.13 | 0.13 | 0 | 3.63 | 3.82 | 5.66 | 5.66E-18 | 6.07 | 1.01E-06 | 1043 |
| Cluster-40555.192918 | 0.66 | 0.64 | 28.67 | 30.77 | 3.21 | 1.4 | 28.12 | 33.18 | 5.66 | 1.40E-22 | 3.82 | 2.47E-14 | 701 |
| Cluster-40555.187889 | 17.46 | 22.6 | 841.77 | 992.83 | 136.8 | 154.02 | 887.83 | 981.11 | 5.67 | 4.80E-50 | 2.71 | 1.27E-16 | 338 |
| Cluster-40555.171808 | 0.51 | 0.14 | 14.9 | 14.62 | 0.9 | 1 | 15.27 | 13.07 | 5.68 | 4.12E-42 | 3.96 | 2.30E-23 | 2322 |
| Cluster-40555.160361 | 0.3 | 0 | 7.3 | 5.96 | 1 | 0.25 | 14.68 | 12.9 | 5.68 | 2.55E-12 | 4.55 | 4.11E-18 | 1241 |
| Cluster-40555.219543 | 0 | 0.24 | 5.28 | 6.13 | 0.26 | 0 | 4.07 | 4.38 | 5.69 | 5.24E-16 | 5.07 | 7.58E-12 | 1795 |
| Cluster-40555.190481 | 0.05 | 0 | 0.57 | 1.22 | 0 | 0 | 1.14 | 0.55 | 5.69 | 2.50E-02 | Inf | 9.10E-03 | 1461 |
| Cluster-40555.160635 | 0.12 | 0 | 2.15 | 2.41 | 0.17 | 0.1 | 4.84 | 4.42 | 5.69 | 1.88E-04 | 5.28 | 6.01E-09 | 1211 |
| Cluster-40555.212365 | 1.3 | 0.78 | 51.61 | 44.83 | 0 | 0 | 12.78 | 13.97 | 5.69 | 1.08E-36 | Inf | 1.41E-18 | 781 |
| Cluster-40555.240866 | 0.01 | 0.08 | 2.29 | 2.28 | 0.51 | 0.69 | 5.69 | 6.43 | 5.70 | 1.18E-14 | 3.41 | 3.44E-16 | 3746 |
| Cluster-40555.185357 | 0.85 | 2.02 | 77.19 | 61.2 | 9.29 | 6.91 | 98.83 | 87.83 | 5.70 | 3.98E-42 | 3.59 | 4.58E-23 | 716 |
| Cluster-40555.157983 | 0.1 | 0.31 | 10.05 | 9.76 | 1.3 | 1.76 | 11.84 | 11.52 | 5.70 | 1.02E-09 | 2.99 | 7.78E-06 | 770 |
| Cluster-40555.163202 | 0.54 | 0 | 11.43 | 13.86 | 0.5 | 0.26 | 10.78 | 7.49 | 5.71 | 1.06E-06 | 4.68 | 2.18E-04 | 521 |
| Cluster-40555.145106 | 0 | 0.07 | 1.9 | 1.76 | 0.16 | 0.3 | 1.74 | 1.26 | 5.71 | 7.58E-06 | 2.71 | 1.49E-02 | 1892 |
| Cluster-40555.220488 | 0.03 | 0 | 0.48 | 0.86 | 0.05 | 0 | 2.31 | 2.48 | 5.71 | 2.35E-02 | 6.50 | 2.66E-09 | 1891 |
| Cluster-40555.186522 | 0 | 0.36 | 8.63 | 9.36 | 0.48 | 0.23 | 8.54 | 8.47 | 5.72 | 2.03E-33 | 4.69 | 1.80E-24 | 2698 |
| Cluster-40555.181916 | 0.21 | 0 | 5.09 | 4.13 | 0 | 0.42 | 10.56 | 6.37 | 5.72 | 2.73E-02 | 5.33 | 2.60E-04 | 503 |
| Cluster-40555.97572 | 0.04 | 0.03 | 1.42 | 1.06 | 0.11 | 0.04 | 0.88 | 0.98 | 5.72 | 3.27E-04 | 3.85 | 1.53E-02 | 1970 |

| | | | | | | | | | | | | | |
|---|---|---|---|---|---|---|---|---|---|---|---|---|---|
| Cluster-40555.34707 | 0 | 0.01 | 0.2 | 0.18 | 0.02 | 0.04 | 0.68 | 0.62 | 5.72 | 4.76E-02 | 4.60 | 2.74E-06 | 5441 |
| Cluster-40555.147392 | 0.05 | 0 | 0.87 | 1.4 | 0.08 | 0 | 1.46 | 1.27 | 5.73 | 2.28E-02 | 4.91 | 1.20E-02 | 1219 |
| Cluster-40555.190333 | 0.71 | 0.46 | 28.02 | 27.4 | 0.56 | 0.29 | 49.08 | 50.65 | 5.73 | 1.77E-17 | 6.96 | 2.30E-31 | 606 |
| Cluster-40555.152949 | 0.29 | 0.32 | 14.67 | 14.7 | 1.61 | 0.6 | 12.56 | 16.17 | 5.73 | 7.76E-14 | 3.79 | 4.17E-09 | 761 |
| Cluster-40555.154837 | 0.1 | 0.11 | 4.56 | 5.16 | 0.14 | 0.56 | 5.94 | 5.48 | 5.73 | 5.99E-14 | 4.05 | 6.76E-12 | 1772 |
| Cluster-40555.223836 | 0.1 | 0 | 2.15 | 2.83 | 0 | 0 | 2.57 | 3.72 | 5.73 | 7.62E-07 | Inf | 1.23E-11 | 1598 |
| Cluster-40555.175146 | 2.44 | 0.37 | 70.3 | 55.41 | 1.97 | 2.55 | 84.29 | 62.98 | 5.73 | 1.66E-12 | 5.06 | 8.74E-13 | 364 |
| Cluster-40555.210408 | 0 | 0.07 | 1.42 | 1.48 | 0.1 | 0.13 | 0.8 | 1.67 | 5.73 | 5.63E-06 | 3.64 | 4.79E-02 | 2341 |
| Cluster-40555.110463 | 0.03 | 0.04 | 1.63 | 1.47 | 0 | 0.21 | 2.57 | 1.35 | 5.73 | 2.80E-04 | 4.20 | 8.64E-04 | 1642 |
| Cluster-40555.202876 | 0.46 | 0 | 10.88 | 9.29 | 1.99 | 1.06 | 13.94 | 14.73 | 5.73 | 9.83E-15 | 3.31 | 1.81E-10 | 1040 |
| Cluster-40555.239799 | 0 | 0.1 | 2.78 | 3.13 | 0 | 0 | 1.56 | 1.74 | 5.73 | 4.67E-04 | Inf | 5.15E-03 | 939 |
| Cluster-40555.168177 | 0.06 | 0 | 1.51 | 1.72 | 0 | 0 | 0.68 | 1.42 | 5.74 | 1.27E-04 | Inf | 2.09E-03 | 1652 |
| Cluster-40555.219128 | 0.29 | 0.24 | 11.66 | 13.52 | 0.14 | 0.45 | 7.78 | 5.08 | 5.74 | 1.73E-08 | 4.48 | 5.69E-04 | 599 |
| Cluster-40555.236973 | 0.06 | 0 | 1.6 | 1.41 | 0 | 0.16 | 1.89 | 1.42 | 5.74 | 1.40E-04 | 4.34 | 1.65E-04 | 1751 |
| Cluster-40555.158613 | 0.07 | 0.14 | 5.49 | 5.08 | 0 | 0 | 1.91 | 2.05 | 5.75 | 2.55E-20 | Inf | 2.91E-12 | 2443 |
| Cluster-40555.170967 | 0.09 | 0 | 2.23 | 1.86 | 0.13 | 0.04 | 2.12 | 2.11 | 5.75 | 1.36E-04 | 4.71 | 1.82E-04 | 1363 |
| Cluster-40555.186388 | 1.77 | 1.85 | 87.19 | 89.36 | 41.55 | 47.93 | 101.45 | 103.41 | 5.75 | 7.13E-86 | 1.26 | 1.18E-04 | 1846 |
| Cluster-40555.216420 | 0 | 0.14 | 3.09 | 3.63 | 0 | 0 | 0.85 | 0.85 | 5.75 | 9.59E-12 | Inf | 2.33E-04 | 2081 |
| Cluster-40555.154380 | 0.73 | 0 | 14.23 | 19.17 | 0.48 | 1.01 | 22.44 | 31.92 | 5.75 | 5.34E-07 | 5.23 | 2.11E-10 | 464 |
| Cluster-40555.249054 | 0.07 | 0 | 1.71 | 1.37 | 0 | 0 | 0.73 | 0.37 | 5.75 | 1.32E-04 | Inf | 3.46E-02 | 1735 |
| Cluster-40555.208426 | 0 | 0.18 | 4.37 | 5.18 | 0.26 | 0.09 | 2.93 | 3.14 | 5.76 | 2.98E-15 | 4.17 | 5.29E-08 | 1976 |
| Cluster-40555.194386 | 0.17 | 0 | 3.85 | 5.4 | 0 | 0 | 1.42 | 4.03 | 5.76 | 4.88E-07 | Inf | 2.36E-02 | 999 |
| Cluster-40555.214039 | 0.34 | 0 | 8.51 | 7.34 | 0.92 | 0.91 | 6.21 | 6.29 | 5.76 | 9.30E-19 | 2.85 | 6.57E-07 | 1559 |
| Cluster-40555.169355 | 0 | 0.13 | 3.42 | 3.05 | 0 | 0 | 5.94 | 6.1 | 5.76 | 7.21E-10 | Inf | 1.45E-24 | 1848 |
| Cluster-40555.158578 | 0.03 | 0 | 0.7 | 0.84 | 1.18 | 0.54 | 2.69 | 2.81 | 5.76 | 2.09E-02 | 1.77 | 4.97E-02 | 1725 |
| Cluster-40555.154449 | 0.11 | 0 | 3 | 2.47 | 0.08 | 0.33 | 1.4 | 1.98 | 5.76 | 4.05E-11 | 3.13 | 3.27E-04 | 2353 |
| Cluster-40555.203723 | 0.14 | 0.06 | 4.51 | 4.88 | 0.43 | 1.58 | 5.81 | 6.35 | 5.77 | 7.73E-15 | 2.63 | 2.62E-07 | 1921 |
| Cluster-40555.188938 | 17.66 | 20.01 | 911.09 | 959.37 | 527.18 | 492.2 | 1255.64 | 918.83 | 5.77 | 1.79E-112 | 1.15 | 5.98E-04 | 991 |
| Cluster-40555.202905 | 0.37 | 0 | 9.98 | 7.75 | 4.58 | 4.51 | 9.73 | 10.13 | 5.77 | 2.66E-31 | 1.20 | 6.99E-03 | 2472 |
| Cluster-40555.149943 | 0.06 | 0.03 | 1.94 | 1.94 | 0.24 | 0.26 | 2.46 | 2.82 | 5.77 | 7.53E-13 | 3.47 | 5.99E-10 | 3703 |
| Cluster-40555.131448 | 0 | 0.02 | 0.93 | 0.32 | 0.13 | 0.12 | 1.2 | 0.92 | 5.78 | 4.83E-02 | 3.12 | 2.81E-02 | 1926 |
| Cluster-40555.176767 | 0 | 0.48 | 13.5 | 10.92 | 0.92 | 0.67 | 5.68 | 7.51 | 5.78 | 4.74E-32 | 3.13 | 1.74E-08 | 1969 |

| | | | | | | | | | | | | | |
|---|---|---|---|---|---|---|---|---|---|---|---|---|---|
| Cluster-40555.198843 | 0 | 0.1 | 2.38 | 2.96 | 0.12 | 0 | 2.77 | 2.47 | 5.78 | 3.55E-02 | 5.53 | 3.16E-02 | 650 |
| Cluster-40555.227609 | 0.15 | 0 | 3.56 | 3.5 | 0 | 0.61 | 6.58 | 5.72 | 5.79 | 1.97E-02 | 4.34 | 1.24E-03 | 590 |
| Cluster-40555.229382 | 0.09 | 0 | 1.7 | 2.85 | 0 | 0.33 | 2.01 | 2.96 | 5.79 | 3.14E-06 | 3.98 | 8.85E-06 | 1789 |
| Cluster-40555.201846 | 0.05 | 0.02 | 1.74 | 2.01 | 0.03 | 0.06 | 2.49 | 2.52 | 5.79 | 4.69E-11 | 5.74 | 2.63E-15 | 3247 |
| Cluster-40555.134313 | 0.1 | 0 | 3.18 | 2.37 | 0.15 | 0.21 | 4.45 | 2.89 | 5.79 | 5.16E-07 | 4.48 | 4.56E-08 | 1528 |
| Cluster-40555.182940 | 0 | 0.33 | 9.16 | 8.83 | 0.94 | 2.29 | 13.32 | 9.89 | 5.80 | 2.79E-06 | 2.88 | 3.85E-04 | 612 |
| Cluster-40555.171510 | 0.12 | 0.14 | 7.34 | 4.77 | 7.63 | 6.44 | 15.27 | 13.77 | 5.80 | 5.62E-13 | 1.11 | 2.21E-02 | 1541 |
| Cluster-40555.188572 | 0 | 22.49 | 634.11 | 548.97 | 0 | 8.78 | 272.36 | 258.8 | 5.80 | 1.03E-111 | 5.93 | 6.04E-72 | 1581 |
| Cluster-40555.220079 | 0 | 0.31 | 8.3 | 8.29 | 0.14 | 0.15 | 7.53 | 6.81 | 5.80 | 2.28E-37 | 5.65 | 2.79E-31 | 3318 |
| Cluster-40555.156915 | 0.19 | 0 | 5.47 | 3.82 | 0 | 0 | 1.82 | 4.07 | 5.80 | 9.27E-05 | Inf | 5.81E-03 | 770 |
| Cluster-40555.179296 | 2.47 | 1.33 | 104.74 | 87.96 | 36.21 | 35.11 | 82.07 | 72.59 | 5.81 | 1.16E-45 | 1.18 | 5.38E-03 | 614 |
| Cluster-40555.172869 | 0 | 0.17 | 4.44 | 4.57 | 0.67 | 0.42 | 3.2 | 3.96 | 5.81 | 2.51E-10 | 2.79 | 5.27E-04 | 1438 |
| Cluster-40555.172750 | 0.12 | 0 | 3.5 | 3.97 | 0.48 | 0.37 | 2.93 | 3.97 | 5.81 | 6.56E-05 | 3.11 | 1.19E-02 | 890 |
| Cluster-40555.149428 | 1.1 | 0.42 | 38.94 | 35.72 | 1.67 | 2.75 | 82.44 | 86.55 | 5.82 | 1.74E-35 | 5.31 | 8.81E-43 | 877 |
| Cluster-40555.191251 | 0.54 | 0.05 | 15.98 | 13.37 | 1.63 | 1.21 | 16.64 | 15.95 | 5.82 | 7.41E-51 | 3.59 | 1.75E-23 | 2992 |
| Cluster-40555.114126 | 0.05 | 0.04 | 2.42 | 2.26 | 0.68 | 0.87 | 3.98 | 3.49 | 5.82 | 1.29E-04 | 2.33 | 7.79E-03 | 1229 |
| Cluster-40555.182036 | 0 | 0.54 | 16.31 | 13.27 | 2.51 | 2.19 | 18.04 | 17.13 | 5.82 | 1.89E-17 | 2.96 | 3.78E-09 | 914 |
| Cluster-40555.227514 | 0.1 | 0.44 | 14.29 | 13.53 | 6.57 | 7 | 25.8 | 22.9 | 5.83 | 7.00E-18 | 1.90 | 1.11E-05 | 965 |
| Cluster-40555.170019 | 0.1 | 0.13 | 6.57 | 5.39 | 0.35 | 0.34 | 8.89 | 9.61 | 5.83 | 6.82E-11 | 4.76 | 3.42E-14 | 1204 |
| Cluster-40555.115115 | 0 | 0.02 | 0.51 | 0.43 | 0 | 0 | 0.47 | 0.36 | 5.84 | 3.18E-02 | Inf | 1.47E-02 | 2543 |
| Cluster-40555.90992 | 0.07 | 0 | 1.54 | 1.6 | 0.97 | 1.21 | 3.97 | 4.44 | 5.84 | 1.55E-02 | 1.99 | 4.42E-02 | 1024 |
| Cluster-40555.173511 | 0 | 0.23 | 5.99 | 6.38 | 0 | 0 | 7.87 | 9.02 | 5.84 | 2.99E-02 | Inf | 7.76E-04 | 435 |
| Cluster-40555.152875 | 0 | 0.07 | 1.7 | 2.23 | 0 | 0 | 1.27 | 2.46 | 5.84 | 2.82E-02 | Inf | 8.29E-03 | 814 |
| Cluster-40555.198335 | 0.34 | 0.42 | 20.78 | 18.56 | 0 | 0 | 6.2 | 6.29 | 5.84 | 1.77E-48 | Inf | 3.51E-29 | 2160 |
| Cluster-40555.274447 | 0.11 | 0 | 2.37 | 2.92 | 0.1 | 0.16 | 1.5 | 1.72 | 5.84 | 4.69E-05 | 3.66 | 2.76E-02 | 1171 |
| Cluster-40555.166389 | 1.78 | 1.34 | 81.8 | 79.12 | 3.73 | 3.11 | 56.76 | 87.37 | 5.85 | 2.93E-39 | 4.47 | 4.43E-07 | 590 |
| Cluster-40555.177066 | 1.02 | 1.44 | 54.36 | 72.5 | 1.03 | 0 | 118.91 | 168.76 | 5.85 | 6.08E-07 | 8.20 | 3.73E-16 | 306 |
| Cluster-40555.180917 | 0 | 0.29 | 8.95 | 6.82 | 0 | 0 | 5.08 | 4.66 | 5.85 | 5.58E-16 | Inf | 6.52E-15 | 1368 |
| Cluster-40555.270157 | 0 | 0.09 | 2.14 | 2.83 | 0 | 0.11 | 1.23 | 1.66 | 5.85 | 1.63E-04 | 4.70 | 1.52E-02 | 1138 |
| Cluster-40555.174419 | 0.18 | 0 | 4.28 | 4.66 | 0.88 | 0.74 | 8.73 | 5.96 | 5.86 | 1.38E-02 | 3.23 | 1.50E-02 | 534 |
| Cluster-40555.189581 | 5.58 | 4.12 | 240.64 | 266.9 | 16.73 | 6.15 | 195.93 | 251.06 | 5.86 | 1.80E-89 | 4.38 | 1.84E-17 | 834 |
| Cluster-40555.213775 | 0.07 | 0 | 1.44 | 1.99 | 0.3 | 0.31 | 1.71 | 1.72 | 5.86 | 5.01E-10 | 2.54 | 5.13E-04 | 3110 |

| | | | | | | | | | | | | | |
|---|---|---|---|---|---|---|---|---|---|---|---|---|---|
| Cluster-40555.189498 | 1.32 | 0.58 | 47.81 | 50.89 | 76.79 | 79.02 | 122.18 | 144.36 | 5.86 | 4.95E-68 | 0.84 | 2.94E-02 | 1648 |
| Cluster-40555.256805 | 0 | 0.09 | 2.32 | 2.55 | 0.11 | 0.22 | 4.41 | 3.87 | 5.86 | 2.66E-02 | 4.71 | 1.24E-03 | 713 |
| Cluster-40555.190553 | 2.41 | 5.21 | 213.58 | 202 | 78.53 | 83.89 | 212.44 | 167.83 | 5.87 | 2.60E-69 | 1.28 | 2.28E-04 | 617 |
| Cluster-40555.219908 | 0.12 | 0 | 3.64 | 2.51 | 0.68 | 0.8 | 4.55 | 4.16 | 5.87 | 9.09E-10 | 2.62 | 1.56E-05 | 1879 |
| Cluster-40555.186618 | 1.93 | 1.22 | 92.03 | 75.02 | 71.15 | 67.6 | 199.11 | 185.03 | 5.87 | 4.46E-60 | 1.53 | 1.64E-06 | 912 |
| Cluster-40555.259794 | 0 | 0.1 | 3.02 | 2.34 | 0 | 0.15 | 3.8 | 4.23 | 5.88 | 2.75E-02 | 6.08 | 6.23E-04 | 678 |
| Cluster-40555.185029 | 0.63 | 0.17 | 21.19 | 19.89 | 0.21 | 0.22 | 17.99 | 15.95 | 5.88 | 1.06E-09 | 6.39 | 3.21E-09 | 497 |
| Cluster-40555.202917 | 0.71 | 0.37 | 36.48 | 22.33 | 6.75 | 2.11 | 40.77 | 37.95 | 5.88 | 7.85E-10 | 3.25 | 5.30E-09 | 507 |
| Cluster-40555.143737 | 0 | 0.03 | 0.81 | 0.89 | 0.1 | 0 | 1.29 | 1.52 | 5.89 | 2.37E-02 | 4.86 | 1.06E-03 | 1576 |
| Cluster-40555.202126 | 0.13 | 0 | 3.03 | 3.58 | 0.19 | 0.33 | 2.09 | 4.09 | 5.89 | 3.08E-05 | 3.65 | 2.51E-02 | 1015 |
| Cluster-40555.73977 | 0 | 0.31 | 10.68 | 6.61 | 0 | 0 | 5.48 | 9.46 | 5.89 | 1.25E-06 | Inf | 1.73E-06 | 652 |
| Cluster-40555.184994 | 1.41 | 1.5 | 85.22 | 72.79 | 81.59 | 110.6 | 189.9 | 153.46 | 5.90 | 5.78E-96 | 0.89 | 1.09E-02 | 2751 |
| Cluster-40555.165288 | 0 | 0.2 | 6.3 | 5.29 | 1.26 | 0.26 | 11.56 | 11.4 | 5.90 | 2.45E-02 | 4.01 | 1.85E-03 | 456 |
| Cluster-40555.172486 | 0.03 | 0 | 0.97 | 0.41 | 0.27 | 0.67 | 1.76 | 2.79 | 5.90 | 3.39E-03 | 2.32 | 1.74E-02 | 3941 |
| Cluster-40555.188835 | 0.87 | 0.42 | 33.88 | 34.77 | 16.07 | 19.45 | 56.72 | 52.27 | 5.91 | 3.61E-56 | 1.68 | 3.10E-07 | 1606 |
| Cluster-40555.135789 | 0.12 | 0 | 2.68 | 3.76 | 0.73 | 1.15 | 6.56 | 6.68 | 5.91 | 1.02E-02 | 2.86 | 8.57E-03 | 652 |
| Cluster-40555.185097 | 2.25 | 2.15 | 119.49 | 120.31 | 14.96 | 13.07 | 86.82 | 75.6 | 5.91 | 2.57E-66 | 2.60 | 8.19E-14 | 800 |
| Cluster-40555.203931 | 0 | 0.36 | 7.02 | 13.6 | 0.89 | 1.86 | 11.2 | 14.84 | 5.91 | 3.96E-04 | 3.28 | 1.62E-03 | 480 |
| Cluster-40555.185477 | 2.68 | 4.41 | 183.63 | 209.5 | 56.33 | 51.29 | 182.64 | 210.35 | 5.92 | 7.77E-73 | 1.94 | 1.58E-10 | 1368 |
| Cluster-40555.162024 | 0.34 | 0 | 11.05 | 7.12 | 0.74 | 5.28 | 14.53 | 11.91 | 5.92 | 3.68E-12 | 2.16 | 1.51E-04 | 964 |
| Cluster-40555.170712 | 0.56 | 0 | 16.56 | 13.61 | 0 | 0.13 | 27.57 | 25.2 | 5.92 | 3.69E-33 | 8.52 | 1.54E-54 | 1616 |
| Cluster-40555.190062 | 6.26 | 2 | 241.51 | 203.05 | 73.61 | 62.81 | 172.76 | 148.81 | 5.92 | 8.00E-80 | 1.30 | 1.80E-04 | 714 |
| Cluster-40555.197539 | 0.1 | 0.26 | 9.53 | 10.24 | 2.01 | 2.06 | 11.67 | 12.01 | 5.93 | 8.84E-37 | 2.60 | 5.29E-12 | 2656 |
| Cluster-40555.249331 | 0 | 0.1 | 2.76 | 3.34 | 0 | 0 | 2.39 | 2.66 | 5.93 | 8.98E-05 | Inf | 2.89E-05 | 1010 |
| Cluster-40555.199459 | 0.03 | 0 | 0.73 | 0.73 | 0.05 | 0 | 0.35 | 0.41 | 5.93 | 2.36E-05 | 3.95 | 3.30E-02 | 3783 |
| Cluster-40555.235277 | 0.13 | 0.22 | 9.71 | 10.07 | 0.08 | 0 | 2.26 | 1.6 | 5.93 | 2.60E-25 | 6.02 | 1.69E-06 | 1716 |
| Cluster-40555.136533 | 0.12 | 0 | 2.71 | 3.82 | 0.03 | 0.06 | 4.44 | 3.72 | 5.93 | 1.29E-10 | 6.51 | 1.06E-14 | 1830 |
| Cluster-40555.210585 | 0.03 | 0 | 0.7 | 0.52 | 0.04 | 0.14 | 1.34 | 0.82 | 5.94 | 1.06E-02 | 3.61 | 1.86E-03 | 2401 |
| Cluster-40555.163040 | 0.08 | 0 | 1.96 | 2.15 | 0.22 | 0.39 | 2.67 | 3.25 | 5.94 | 9.34E-03 | 3.31 | 1.57E-02 | 890 |
| Cluster-40555.195655 | 0.22 | 0 | 4.07 | 5.15 | 0.27 | 0.5 | 5.19 | 5.58 | 5.95 | 8.54E-03 | 3.65 | 2.77E-02 | 542 |
| Cluster-40555.101453 | 0 | 0.06 | 1.22 | 2.13 | 0.19 | 0.31 | 1.55 | 2.82 | 5.96 | 1.61E-02 | 3.24 | 4.57E-02 | 957 |
| Cluster-40555.239504 | 0 | 0.34 | 8.8 | 10.6 | 0.73 | 0.67 | 6.98 | 5.31 | 5.96 | 1.77E-21 | 3.21 | 1.10E-07 | 1470 |

| | | | | | | | | | | | | | |
|---|---|---|---|---|---|---|---|---|---|---|---|---|---|
| Cluster-40555.179120 | 0.51 | 0.22 | 20.13 | 21.83 | 0.16 | 0 | 16.3 | 12.12 | 5.96 | 7.39E-25 | 7.75 | 9.43E-22 | 921 |
| Cluster-40555.217978 | 0.08 | 0 | 2.37 | 1.75 | 0.29 | 0.2 | 1.2 | 1.34 | 5.96 | 7.39E-13 | 2.45 | 2.88E-03 | 3448 |
| Cluster-40555.188543 | 0.57 | 0.46 | 32.13 | 25.94 | 4.19 | 4.83 | 68.44 | 62.84 | 5.96 | 1.64E-18 | 3.92 | 4.07E-20 | 604 |
| Cluster-40555.182355 | 2.79 | 2.04 | 132.82 | 139.39 | 16.81 | 10.69 | 234.04 | 192.92 | 5.96 | 1.81E-87 | 4.03 | 1.15E-37 | 1196 |
| Cluster-40555.151949 | 0.13 | 0 | 4.06 | 2.63 | 0 | 0 | 2.33 | 1.94 | 5.96 | 2.25E-05 | Inf | 1.19E-04 | 1045 |
| Cluster-40555.149023 | 0.05 | 0 | 1.25 | 1.41 | 0 | 0 | 2.26 | 1.32 | 5.96 | 8.28E-03 | Inf | 6.18E-05 | 1248 |
| Cluster-40555.212120 | 0 | 0.43 | 13.66 | 11.53 | 0.25 | 0.36 | 2.92 | 4.59 | 5.97 | 1.21E-15 | 3.73 | 9.62E-04 | 919 |
| Cluster-40555.174936 | 0 | 0.08 | 2.1 | 2.71 | 0 | 0 | 1.23 | 1.27 | 5.97 | 2.44E-07 | Inf | 3.46E-05 | 1742 |
| Cluster-40555.172342 | 0.42 | 0.33 | 21.75 | 22.29 | 0.52 | 0 | 7.83 | 8.23 | 5.98 | 4.85E-17 | 5.19 | 2.02E-06 | 663 |
| Cluster-40555.176302 | 0.59 | 0 | 16.91 | 14.98 | 0.41 | 0.41 | 9.56 | 10.12 | 5.98 | 3.35E-08 | 4.68 | 1.57E-04 | 511 |
| Cluster-40555.229605 | 0.08 | 0.06 | 3.91 | 4.1 | 1.05 | 0.79 | 4.36 | 5.75 | 5.98 | 2.84E-05 | 2.54 | 8.35E-03 | 891 |
| Cluster-40555.200456 | 0 | 1.13 | 31.5 | 35.78 | 0 | 0 | 19.34 | 28.02 | 5.98 | 2.01E-44 | Inf | 1.96E-18 | 1184 |
| Cluster-40555.217821 | 0 | 0.24 | 9.07 | 4.82 | 0.46 | 0 | 4.95 | 6.76 | 5.99 | 2.18E-06 | 4.77 | 8.42E-11 | 1657 |
| Cluster-40555.86906 | 0.09 | 0.22 | 8.51 | 9.66 | 2.09 | 0.37 | 14.18 | 10.19 | 5.99 | 5.01E-10 | 3.40 | 2.26E-07 | 804 |
| Cluster-40555.192128 | 0 | 0.12 | 3.76 | 4.17 | 1.94 | 1.37 | 6.23 | 4.67 | 5.99 | 1.04E-09 | 1.79 | 5.88E-03 | 1475 |
| Cluster-40555.207616 | 0.4 | 0 | 10.3 | 10.9 | 0.18 | 1.37 | 7.96 | 9.13 | 5.99 | 1.85E-26 | 3.50 | 2.54E-12 | 1651 |
| Cluster-40555.295436 | 0 | 0.17 | 7.43 | 3.57 | 0.84 | 0 | 2.93 | 4.05 | 6.00 | 1.92E-04 | 3.15 | 9.85E-03 | 927 |
| Cluster-40555.188589 | 2.59 | 4.32 | 183.73 | 219.75 | 45.6 | 42.27 | 239.22 | 276.72 | 6.00 | 3.47E-46 | 2.62 | 1.16E-17 | 826 |
| Cluster-40555.188065 | 73.31 | 57.89 | 3333.83 | 3695.65 | 518.97 | 399.92 | 2246.3 | 1826.9 | 6.00 | 1.64E-73 | 2.15 | 3.00E-09 | 260 |
| Cluster-40555.184150 | 0.05 | 0 | 1.68 | 1.49 | 0.1 | 0.09 | 1.27 | 1 | 6.00 | 5.98E-11 | 3.60 | 2.64E-05 | 3662 |
| Cluster-40555.167849 | 0.11 | 0 | 2.76 | 3.3 | 0 | 0.22 | 3.56 | 1.9 | 6.01 | 6.50E-03 | 4.61 | 2.14E-02 | 707 |
| Cluster-40555.188167 | 66.51 | 47.17 | 3311.26 | 3306.86 | 2890.61 | 2695.35 | 6064.37 | 5262.56 | 6.02 | 1.07E-125 | 1.08 | 9.01E-04 | 792 |
| Cluster-40555.182280 | 0 | 0.18 | 5.45 | 5.7 | 0.33 | 0.74 | 4.13 | 6.58 | 6.02 | 6.57E-10 | 3.41 | 4.71E-04 | 1142 |
| Cluster-40555.201490 | 0.11 | 0 | 2.83 | 3.04 | 0.33 | 0.15 | 5.98 | 5.21 | 6.02 | 8.64E-06 | 4.53 | 7.84E-09 | 1189 |
| Cluster-40555.90617 | 0 | 0.05 | 1.42 | 1.42 | 0.38 | 0.17 | 2.1 | 2.28 | 6.03 | 1.35E-02 | 3.08 | 2.48E-02 | 1130 |
| Cluster-40555.163788 | 0.06 | 0.16 | 6.73 | 6.4 | 0.12 | 0.26 | 5.51 | 5.26 | 6.03 | 3.10E-10 | 4.88 | 5.86E-08 | 1035 |
| Cluster-40555.122398 | 0 | 0.06 | 1.51 | 1.84 | 0.27 | 0.29 | 2.18 | 2.48 | 6.03 | 1.27E-02 | 3.23 | 2.74E-02 | 998 |
| Cluster-40555.180173 | 0.09 | 0 | 2.41 | 2.97 | 1.49 | 0.15 | 7.13 | 4.99 | 6.03 | 7.42E-06 | 3.01 | 7.09E-06 | 1278 |
| Cluster-40555.138888 | 0.04 | 0.01 | 1.7 | 1.24 | 0.03 | 0.54 | 1.92 | 1.64 | 6.03 | 4.07E-08 | 2.66 | 1.81E-04 | 2986 |
| Cluster-40555.182579 | 3.77 | 0 | 92.39 | 100.04 | 0.95 | 0.98 | 90.56 | 84.43 | 6.03 | 2.57E-11 | 6.52 | 4.00E-10 | 311 |
| Cluster-40555.190118 | 13.17 | 12.83 | 762.08 | 787.72 | 305.84 | 312.91 | 819.86 | 852.29 | 6.04 | 3.38E-115 | 1.50 | 9.16E-07 | 856 |
| Cluster-40555.119464 | 0.13 | 0 | 3.48 | 3.95 | 0 | 0 | 6.08 | 4.02 | 6.04 | 5.60E-03 | Inf | 3.66E-05 | 633 |

| | | | | | | | | | | | | | |
|---|---|---|---|---|---|---|---|---|---|---|---|---|---|
| Cluster-40555.160389 | 0 | 0.36 | 11.68 | 11.06 | 4.77 | 5.01 | 20.34 | 13.94 | 6.04 | 5.09E-10 | 1.86 | 1.98E-03 | 699 |
| Cluster-40555.210734 | 0.08 | 0.25 | 10.26 | 9.58 | 0 | 0 | 5.86 | 4.98 | 6.04 | 1.25E-12 | Inf | 8.49E-10 | 904 |
| Cluster-40555.229626 | 0 | 0.71 | 21.66 | 23.39 | 2.78 | 2.89 | 31.94 | 40.08 | 6.04 | 3.32E-05 | 3.71 | 3.84E-05 | 370 |
| Cluster-40555.188041 | 0 | 0.35 | 11.01 | 11.29 | 0 | 0 | 6.88 | 7.37 | 6.05 | 1.45E-24 | Inf | 6.49E-23 | 1493 |
| Cluster-40555.167564 | 0.63 | 2.12 | 85.72 | 81.71 | 0.38 | 0 | 51.61 | 33.69 | 6.05 | 6.62E-50 | 7.74 | 2.79E-17 | 704 |
| Cluster-40555.141822 | 0.28 | 0 | 8.19 | 7.77 | 0 | 0 | 1.52 | 0.96 | 6.05 | 5.27E-14 | Inf | 6.38E-03 | 1138 |
| Cluster-40555.186501 | 2.31 | 1.45 | 123.49 | 101.59 | 0.42 | 0.25 | 5.21 | 4.26 | 6.06 | 6.32E-99 | 3.91 | 1.20E-10 | 1977 |
| Cluster-40555.186665 | 0.25 | 0.07 | 9.43 | 9.37 | 1.46 | 0.77 | 19.61 | 18.71 | 6.06 | 3.76E-11 | 4.18 | 2.26E-14 | 843 |
| Cluster-40555.195633 | 11.07 | 8.53 | 669.37 | 436.56 | 19.68 | 4.47 | 96.09 | 105.24 | 6.06 | 2.90E-15 | 3.09 | 1.66E-02 | 268 |
| Cluster-40555.173466 | 0.96 | 0.81 | 49.23 | 56.93 | 10.05 | 9.39 | 78.72 | 77.95 | 6.06 | 6.58E-61 | 3.08 | 1.75E-22 | 1617 |
| Cluster-40555.188945 | 17.39 | 36.43 | 1705.33 | 1628.37 | 74.62 | 47.18 | 836.75 | 1090.73 | 6.07 | 5.97E-123 | 4.06 | 1.66E-14 | 876 |
| Cluster-40555.194285 | 1.09 | 0.33 | 39.35 | 43 | 0.17 | 0 | 22.69 | 25.93 | 6.07 | 3.08E-36 | 8.22 | 1.94E-28 | 797 |
| Cluster-40555.102684 | 0.07 | 0.24 | 9.27 | 10.38 | 1.02 | 0.92 | 10.29 | 7.63 | 6.08 | 5.74E-24 | 3.27 | 6.22E-11 | 1592 |
| Cluster-40555.136428 | 0.04 | 0.09 | 3.93 | 4.26 | 0.04 | 0.15 | 1.77 | 2.17 | 6.08 | 9.21E-11 | 4.42 | 1.20E-04 | 1549 |
| Cluster-40555.206569 | 0.15 | 1.24 | 39.25 | 49.53 | 2.4 | 0.47 | 37.55 | 42.03 | 6.08 | 9.43E-26 | 4.89 | 1.41E-18 | 581 |
| Cluster-40555.190050 | 3.06 | 1.97 | 114.79 | 195.86 | 0 | 0 | 59.17 | 98.15 | 6.08 | 1.05E-07 | Inf | 6.09E-12 | 479 |
| Cluster-40555.165583 | 0 | 0.03 | 0.94 | 1.12 | 0.47 | 0.65 | 2.63 | 2.36 | 6.08 | 9.69E-03 | 2.20 | 2.63E-02 | 1494 |
| Cluster-40555.185730 | 1.53 | 0.5 | 43.15 | 76.62 | 6.99 | 10.35 | 61.17 | 67.04 | 6.09 | 1.03E-06 | 2.94 | 1.36E-12 | 583 |
| Cluster-40555.199609 | 0.13 | 0.14 | 8.41 | 8.77 | 0.2 | 0.19 | 4.59 | 5.58 | 6.09 | 6.43E-40 | 4.74 | 8.11E-21 | 3242 |
| Cluster-40555.166030 | 0.09 | 0 | 3.13 | 1.92 | 0 | 0 | 3.24 | 2.78 | 6.09 | 6.30E-06 | Inf | 1.17E-09 | 1394 |
| Cluster-40555.122553 | 0.25 | 0 | 8.41 | 6.67 | 0 | 0 | 4.86 | 4.14 | 6.09 | 5.19E-06 | Inf | 9.93E-05 | 643 |
| Cluster-40555.136569 | 0 | 0.07 | 1.59 | 1.87 | 0 | 0.06 | 1.21 | 1.84 | 6.10 | 1.81E-05 | 5.56 | 3.72E-05 | 1760 |
| Cluster-40555.160526 | 0 | 0.76 | 24.44 | 24.79 | 0.45 | 0.08 | 17.69 | 17.24 | 6.10 | 2.24E-27 | 6.15 | 2.51E-21 | 894 |
| Cluster-40555.247071 | 0 | 0.07 | 3.2 | 1.48 | 0.24 | 0 | 4.21 | 4.34 | 6.10 | 1.17E-02 | 5.19 | 6.86E-05 | 821 |
| Cluster-40555.185508 | 0 | 0.23 | 8.18 | 7.31 | 2.24 | 1.92 | 8.18 | 8.22 | 6.11 | 1.35E-21 | 2.05 | 2.09E-05 | 1778 |
| Cluster-40555.206506 | 0.04 | 0 | 1.52 | 2.44 | 0 | 0 | 2.5 | 2.25 | 6.11 | 3.34E-03 | Inf | 6.96E-05 | 998 |
| Cluster-40555.188988 | 3.98 | 1.19 | 199.2 | 116.27 | 7.95 | 7.46 | 161.6 | 114.4 | 6.11 | 3.02E-10 | 4.23 | 6.16E-16 | 704 |
| Cluster-40555.118757 | 0 | 0.12 | 3.64 | 4.11 | 0.14 | 0.15 | 3.96 | 3.65 | 6.12 | 8.33E-03 | 4.77 | 1.57E-02 | 599 |
| Cluster-40555.175210 | 0.06 | 0.06 | 4.17 | 4.09 | 0.2 | 0.16 | 2.14 | 3.12 | 6.12 | 2.27E-11 | 3.97 | 1.76E-05 | 1611 |
| Cluster-40555.185129 | 1.47 | 3.44 | 153.61 | 162.81 | 22.48 | 24.92 | 145.59 | 102.34 | 6.12 | 1.51E-35 | 2.43 | 5.72E-06 | 397 |
| Cluster-40555.233960 | 0.3 | 0.1 | 13.04 | 11.25 | 0.32 | 0.67 | 13.61 | 14.06 | 6.13 | 1.11E-21 | 4.81 | 2.27E-19 | 1211 |
| Cluster-40555.166768 | 0.21 | 0 | 6.75 | 4.86 | 0 | 0.06 | 9.44 | 11 | 6.14 | 5.68E-12 | 8.75 | 4.81E-25 | 1285 |

| | | | | | | | | | | | | | |
|---|---|---|---|---|---|---|---|---|---|---|---|---|---|
| Cluster-40555.171221 | 0.24 | 0 | 8.13 | 7.84 | 0.99 | 0.5 | 6.05 | 5.97 | 6.14 | 3.71E-12 | 3.10 | 8.05E-05 | 1003 |
| Cluster-40555.228585 | 0 | 0.36 | 10.2 | 14.28 | 0 | 0 | 10.83 | 9.9 | 6.14 | 1.08E-05 | Inf | 4.47E-06 | 478 |
| Cluster-40555.176539 | 0.74 | 0 | 19.71 | 21.64 | 0.74 | 0 | 20.43 | 13.96 | 6.14 | 3.23E-03 | 5.63 | 2.05E-02 | 328 |
| Cluster-40555.189717 | 10.89 | 9.24 | 683.78 | 606.21 | 220.48 | 217.02 | 687.37 | 722.43 | 6.15 | 9.32E-116 | 1.75 | 4.45E-09 | 904 |
| Cluster-40555.191080 | 0.46 | 0 | 12.05 | 16.24 | 0.46 | 0.39 | 4.04 | 6.86 | 6.15 | 1.20E-19 | 3.75 | 1.10E-03 | 2151 |
| Cluster-40555.201861 | 0.31 | 0.38 | 25.18 | 19.3 | 14.64 | 14.58 | 29.37 | 24 | 6.15 | 3.78E-45 | 0.93 | 2.34E-02 | 2104 |
| Cluster-40555.188444 | 62.09 | 87.26 | 4620.34 | 5103.01 | 1032.7 | 1012.73 | 3198.54 | 3132.04 | 6.16 | 7.57E-120 | 1.70 | 8.73E-09 | 1262 |
| Cluster-40555.165359 | 1.81 | 0 | 59.92 | 45.29 | 12.18 | 14.48 | 121.67 | 96.39 | 6.16 | 2.26E-09 | 3.06 | 7.91E-08 | 344 |
| Cluster-40555.182179 | 0.31 | 0.39 | 25.52 | 20.08 | 2.05 | 1.59 | 15.2 | 16.94 | 6.16 | 1.58E-25 | 3.22 | 2.20E-09 | 884 |
| Cluster-40555.187607 | 5.05 | 1.53 | 243.23 | 173.8 | 155.09 | 90.32 | 238.94 | 253.81 | 6.17 | 3.34E-27 | 1.08 | 6.08E-03 | 494 |
| Cluster-40555.161986 | 0.2 | 0.1 | 9.91 | 8.21 | 3.88 | 4.64 | 11.32 | 11.84 | 6.17 | 7.18E-22 | 1.50 | 1.36E-03 | 1548 |
| Cluster-40555.179580 | 0 | 0.22 | 6.33 | 7.49 | 0.05 | 0.47 | 2.43 | 3.04 | 6.17 | 1.00E-13 | 3.48 | 9.26E-04 | 1241 |
| Cluster-40555.163709 | 0.18 | 0.15 | 11.25 | 10.23 | 0 | 0.19 | 6.19 | 4.87 | 6.17 | 4.46E-06 | 5.87 | 2.28E-03 | 529 |
| Cluster-40555.178104 | 0 | 0.27 | 9.21 | 8.69 | 0.91 | 0.79 | 9.93 | 9.81 | 6.18 | 7.69E-23 | 3.61 | 1.27E-13 | 1640 |
| Cluster-40555.182928 | 0.41 | 0.31 | 20.77 | 24.05 | 4.41 | 4.78 | 37.18 | 44.64 | 6.18 | 9.30E-33 | 3.22 | 3.71E-18 | 1103 |
| Cluster-40555.89704 | 0 | 0.04 | 1.24 | 1.27 | 0 | 0 | 1.52 | 1.71 | 6.18 | 6.17E-03 | Inf | 5.71E-05 | 1356 |
| Cluster-40555.217743 | 0.05 | 0.04 | 2.9 | 3.16 | 0.5 | 0.59 | 2.61 | 2.18 | 6.19 | 2.74E-17 | 2.19 | 3.47E-04 | 3141 |
| Cluster-40555.178143 | 0.36 | 0 | 12.72 | 9.23 | 0 | 0 | 8.07 | 6.16 | 6.19 | 1.46E-09 | Inf | 2.40E-08 | 682 |
| Cluster-40555.184212 | 7.14 | 4.81 | 358.06 | 425.37 | 64.9 | 55.4 | 383.34 | 322 | 6.20 | 7.58E-52 | 2.61 | 1.11E-16 | 542 |
| Cluster-40555.132249 | 1.28 | 0.32 | 48.56 | 54.6 | 18.41 | 14.16 | 59.46 | 70.64 | 6.20 | 5.52E-54 | 2.07 | 1.60E-09 | 1020 |
| Cluster-40555.197430 | 0.14 | 0.03 | 5.87 | 5.52 | 2.16 | 2.08 | 9.17 | 6.35 | 6.20 | 3.40E-25 | 1.94 | 1.20E-05 | 2651 |
| Cluster-40555.218410 | 0.04 | 0 | 1 | 1 | 0.23 | 0.16 | 1.52 | 1.28 | 6.20 | 2.38E-03 | 2.90 | 1.78E-02 | 1825 |
| Cluster-40555.154254 | 0.14 | 0 | 3.09 | 5.98 | 0.14 | 0 | 4.25 | 2.85 | 6.20 | 1.87E-03 | 5.76 | 1.11E-02 | 600 |
| Cluster-40555.80141 | 0.03 | 0 | 0.88 | 0.6 | 0.31 | 0.12 | 1.38 | 1.14 | 6.21 | 2.62E-03 | 2.66 | 1.36E-02 | 2376 |
| Cluster-40555.209060 | 0.07 | 0 | 1.79 | 2.33 | 0.13 | 0 | 2.38 | 2.88 | 6.21 | 6.11E-16 | 5.54 | 4.37E-18 | 4036 |
| Cluster-40555.231002 | 0.04 | 0 | 1.4 | 1.32 | 0 | 0 | 4.02 | 2.9 | 6.21 | 2.31E-03 | Inf | 3.49E-11 | 1405 |
| Cluster-40555.169507 | 0 | 0.12 | 3.86 | 2.28 | 0.15 | 0.16 | 3.59 | 4.57 | 6.21 | 6.40E-03 | 4.72 | 1.20E-03 | 721 |
| Cluster-40555.140547 | 0.03 | 0 | 1.18 | 0.71 | 0 | 0.12 | 1.83 | 2.18 | 6.23 | 2.47E-03 | 5.12 | 5.40E-07 | 1940 |
| Cluster-40555.139222 | 0.14 | 0.12 | 9.35 | 8.57 | 0.05 | 0.15 | 3.29 | 3.98 | 6.23 | 1.17E-17 | 5.27 | 1.32E-07 | 1261 |
| Cluster-40555.83482 | 0 | 0.24 | 9.17 | 7.72 | 0 | 0.1 | 7.4 | 7.96 | 6.23 | 8.71E-09 | 7.30 | 7.77E-10 | 765 |
| Cluster-40555.199014 | 0.31 | 0 | 6.13 | 13.38 | 1.24 | 1.62 | 17.19 | 25.77 | 6.23 | 3.11E-03 | 3.95 | 4.83E-05 | 421 |
| Cluster-83369.0 | 0.06 | 0 | 2.19 | 1.46 | 0 | 0 | 2.09 | 2.15 | 6.23 | 2.25E-03 | Inf | 3.46E-05 | 1134 |

| | | | | | | | | | | | | | |
|---|---|---|---|---|---|---|---|---|---|---|---|---|---|
| Cluster-40555.184690 | 0.08 | 0 | 2.31 | 3.61 | 0 | 0 | 1.13 | 0.88 | 6.24 | 7.95E-09 | Inf | 1.69E-04 | 1860 |
| Cluster-40555.123043 | 0 | 0.06 | 1.8 | 2.55 | 0.29 | 0.44 | 3.81 | 3.32 | 6.24 | 4.16E-03 | 3.32 | 4.09E-03 | 918 |
| Cluster-40555.180152 | 0.98 | 0.11 | 29.13 | 39.48 | 5.93 | 3.76 | 38.34 | 60.62 | 6.24 | 2.16E-18 | 3.43 | 1.58E-04 | 671 |
| Cluster-40555.109445 | 0.22 | 0 | 6.86 | 7.27 | 0.65 | 0.68 | 6.75 | 7.12 | 6.24 | 1.86E-03 | 3.43 | 3.70E-02 | 485 |
| Cluster-40555.163846 | 0.15 | 0.61 | 26.15 | 27.84 | 1.98 | 2 | 24.51 | 16.8 | 6.24 | 4.73E-22 | 3.43 | 1.73E-09 | 690 |
| Cluster-40555.228538 | 0.64 | 0 | 27.12 | 12.2 | 8.41 | 4 | 35.52 | 32.42 | 6.24 | 2.49E-03 | 2.51 | 3.17E-02 | 339 |
| Cluster-40555.155595 | 0 | 0.07 | 1.75 | 1.65 | 0 | 0.23 | 3.61 | 3.58 | 6.25 | 4.56E-03 | 4.93 | 5.32E-06 | 1094 |
| Cluster-99237.0 | 0.06 | 0 | 1.99 | 1.97 | 0 | 0 | 1.7 | 1.42 | 6.25 | 1.81E-03 | Inf | 1.87E-03 | 1068 |
| Cluster-40555.183656 | 34.25 | 28.49 | 2204.08 | 2024.97 | 1448.87 | 1164.42 | 2928.47 | 2733.92 | 6.25 | 3.50E-108 | 1.17 | 4.05E-04 | 374 |
| Cluster-40555.193877 | 6.03 | 6.4 | 391.9 | 441.08 | 54.92 | 57.19 | 312.25 | 343.51 | 6.25 | 1.92E-37 | 2.56 | 1.41E-08 | 307 |
| Cluster-40555.184455 | 0.43 | 0.61 | 36.96 | 37.48 | 1.84 | 2.23 | 28.24 | 30.17 | 6.25 | 5.56E-47 | 3.92 | 2.74E-21 | 1110 |
| Cluster-40555.190531 | 0.16 | 0.03 | 6.17 | 6.07 | 0.35 | 0.16 | 4.03 | 3.61 | 6.26 | 5.66E-16 | 3.98 | 2.90E-07 | 1548 |
| Cluster-40555.181746 | 0.82 | 1.39 | 66.85 | 91.06 | 2.23 | 2.49 | 63.53 | 65.26 | 6.26 | 4.38E-20 | 4.83 | 8.52E-41 | 1289 |
| Cluster-40555.166517 | 0.26 | 0.78 | 37.84 | 40.97 | 6.1 | 7.13 | 24.05 | 21.8 | 6.27 | 1.62E-33 | 1.85 | 2.37E-04 | 754 |
| Cluster-40555.55511 | 0.02 | 0 | 1.03 | 0.62 | 0 | 0.03 | 0.68 | 0.45 | 6.27 | 1.87E-03 | 5.49 | 1.74E-02 | 2209 |
| Cluster-40555.179966 | 0.06 | 0.16 | 7.8 | 7.79 | 0.52 | 0.39 | 3.86 | 2.45 | 6.28 | 2.11E-35 | 2.85 | 2.49E-05 | 2958 |
| Cluster-40555.193059 | 0.11 | 0.25 | 11.43 | 13.19 | 9.03 | 5.64 | 19.11 | 20.73 | 6.28 | 2.67E-50 | 1.52 | 1.51E-05 | 3086 |
| Cluster-40555.196908 | 0.18 | 0.18 | 13.06 | 13.07 | 1.98 | 2.09 | 13.11 | 7.39 | 6.28 | 2.63E-36 | 2.40 | 7.68E-03 | 1915 |
| Cluster-40555.154641 | 0.06 | 0 | 2 | 2.85 | 0 | 0 | 0.73 | 1.59 | 6.28 | 1.25E-03 | Inf | 4.52E-02 | 929 |
| Cluster-40555.184498 | 0 | 0.2 | 6.77 | 7.53 | 0.38 | 0.65 | 4.82 | 5.81 | 6.29 | 4.99E-15 | 3.43 | 1.01E-06 | 1296 |
| Cluster-40555.200665 | 0.59 | 0.43 | 35.48 | 37.9 | 30.12 | 27.18 | 51.54 | 61.18 | 6.30 | 7.04E-56 | 1.05 | 6.27E-03 | 1379 |
| Cluster-40555.107433 | 0.05 | 0.04 | 3.56 | 3.06 | 0 | 0.2 | 2.59 | 2.98 | 6.30 | 1.08E-09 | 4.75 | 1.84E-07 | 1666 |
| Cluster-40555.192068 | 0.1 | 0 | 4.15 | 1.64 | 0 | 0 | 1.94 | 1.19 | 6.30 | 8.93E-03 | Inf | 2.08E-02 | 844 |
| Cluster-40555.186835 | 1.56 | 2.15 | 115.89 | 153.07 | 39.97 | 55.08 | 205.82 | 215.05 | 6.31 | 1.24E-23 | 2.20 | 6.40E-12 | 631 |
| Cluster-40555.214634 | 1.51 | 0 | 43.92 | 51.18 | 3.82 | 8.66 | 71.86 | 61.89 | 6.31 | 2.90E-07 | 3.42 | 3.13E-05 | 326 |
| Cluster-40555.185966 | 0.23 | 0 | 8.65 | 7.28 | 1.81 | 0 | 22.57 | 30.38 | 6.31 | 3.61E-07 | 4.99 | 2.56E-13 | 681 |
| Cluster-40555.201838 | 0.19 | 0 | 6.78 | 7.92 | 0.9 | 0.11 | 5.68 | 4.92 | 6.31 | 5.11E-14 | 3.46 | 7.17E-06 | 1171 |
| Cluster-40555.193151 | 0 | 0.67 | 25.94 | 24.6 | 0.17 | 0.09 | 6.44 | 9.37 | 6.32 | 8.78E-79 | 6.05 | 6.39E-12 | 3602 |
| Cluster-40555.251683 | 0 | 0.03 | 0.84 | 1.32 | 0 | 0.19 | 1.58 | 1.66 | 6.32 | 2.55E-03 | 4.20 | 6.08E-04 | 1634 |
| Cluster-40555.189561 | 0.25 | 0.2 | 17.34 | 16.03 | 1.42 | 0.91 | 13.83 | 10.24 | 6.32 | 3.07E-33 | 3.44 | 8.42E-13 | 1408 |
| Cluster-40555.112739 | 0 | 0.02 | 1 | 0.76 | 0.23 | 0.41 | 3.09 | 2.57 | 6.32 | 3.16E-03 | 3.22 | 1.42E-05 | 1959 |
| Cluster-40555.189531 | 0.61 | 1.07 | 60.46 | 58.32 | 6.18 | 6.17 | 37.01 | 39.37 | 6.33 | 1.64E-45 | 2.70 | 1.34E-10 | 763 |

| | | | | | | | | | | | | | |
|---|---|---|---|---|---|---|---|---|---|---|---|---|---|
| Cluster-40555.187614 | 0 | 0.09 | 3.91 | 3.23 | 0.07 | 0.04 | 3.6 | 3.29 | 6.33 | 1.76E-09 | 6.03 | 5.92E-10 | 1538 |
| Cluster-40555.191292 | 0.93 | 1.15 | 74.05 | 76.59 | 6.17 | 6.21 | 36.04 | 27.67 | 6.33 | 1.75E-74 | 2.42 | 6.22E-11 | 1209 |
| Cluster-40555.198751 | 0 | 0.11 | 4.77 | 3.5 | 0 | 0.01 | 2.5 | 3.15 | 6.33 | 2.19E-12 | Inf | 8.46E-12 | 1741 |
| Cluster-40555.173259 | 0.11 | 0 | 4.2 | 3.87 | 0 | 0 | 2.21 | 3.16 | 6.34 | 1.00E-03 | Inf | 5.05E-03 | 686 |
| Cluster-40555.87182 | 0 | 0.04 | 0.98 | 1.24 | 0.05 | 0.11 | 1.41 | 1.23 | 6.34 | 2.32E-03 | 4.28 | 2.45E-03 | 1649 |
| Cluster-40555.129320 | 0 | 0.03 | 0.86 | 1.25 | 0 | 0 | 1.6 | 1.53 | 6.35 | 2.19E-03 | Inf | 1.59E-06 | 1732 |
| Cluster-40555.264339 | 0.04 | 0 | 1.51 | 1.07 | 0.09 | 0.09 | 2.05 | 2.16 | 6.35 | 2.67E-07 | 4.62 | 9.74E-10 | 2921 |
| Cluster-40555.272373 | 0.15 | 0 | 6.81 | 5.67 | 1.53 | 1.32 | 6.01 | 3.91 | 6.35 | 7.52E-11 | 1.86 | 2.90E-02 | 1089 |
| Cluster-40555.183684 | 0.02 | 0.14 | 4.23 | 6.63 | 0.7 | 0 | 4.83 | 3.88 | 6.35 | 9.68E-07 | 3.81 | 1.18E-03 | 824 |
| Cluster-40555.214180 | 0.06 | 0 | 2.32 | 2.8 | 0 | 0.07 | 2.05 | 0.95 | 6.35 | 1.56E-07 | 5.38 | 2.45E-03 | 1605 |
| Cluster-40555.177095 | 0 | 0.07 | 3.89 | 2.07 | 0.16 | 0 | 2.03 | 1.51 | 6.36 | 2.93E-06 | 4.69 | 3.40E-05 | 1831 |
| Cluster-40555.216127 | 0.19 | 0 | 8.15 | 5.99 | 0.36 | 0.14 | 21.72 | 17.32 | 6.36 | 7.15E-11 | 6.32 | 2.60E-26 | 997 |
| Cluster-40555.213735 | 0.2 | 0.96 | 43.65 | 44.04 | 1.73 | 0 | 22.61 | 23.88 | 6.36 | 1.79E-42 | 4.89 | 4.46E-20 | 872 |
| Cluster-40555.181619 | 0.49 | 0.52 | 36.37 | 39.92 | 3.46 | 1.32 | 27.73 | 32.76 | 6.37 | 1.62E-53 | 3.76 | 2.30E-21 | 1247 |
| Cluster-40555.188374 | 0 | 0.1 | 2.42 | 3.86 | 0 | 0 | 2.84 | 3.69 | 6.37 | 7.59E-07 | Inf | 4.06E-09 | 1249 |
| Cluster-40555.165888 | 0.11 | 0.1 | 8.45 | 7.77 | 0.09 | 0.19 | 3.1 | 4.73 | 6.37 | 2.84E-10 | 5.06 | 7.49E-05 | 862 |
| Cluster-40555.146490 | 0.1 | 0 | 2.34 | 4.58 | 0.09 | 0.3 | 4.33 | 3.88 | 6.37 | 1.17E-04 | 4.44 | 3.22E-07 | 1262 |
| Cluster-40555.148227 | 0.13 | 0 | 5.8 | 4.34 | 0.11 | 0 | 5.23 | 3.29 | 6.38 | 4.88E-11 | 6.08 | 5.62E-10 | 1299 |
| Cluster-40555.188161 | 8.39 | 6.27 | 542.38 | 557.57 | 42.1 | 47.59 | 418.42 | 588.59 | 6.38 | 2.50E-118 | 3.56 | 5.86E-08 | 867 |
| Cluster-40555.191064 | 0.93 | 0.54 | 50.96 | 60.88 | 1.64 | 1.58 | 12.57 | 20.49 | 6.38 | 6.12E-49 | 3.45 | 5.99E-04 | 1643 |
| Cluster-40555.161919 | 0.09 | 0.02 | 5.02 | 3.32 | 2.99 | 2.91 | 10.75 | 12.09 | 6.38 | 4.97E-15 | 2.02 | 2.99E-07 | 2333 |
| Cluster-40555.191791 | 0.7 | 0.23 | 31.82 | 36.32 | 26.48 | 28.65 | 55.63 | 70.5 | 6.39 | 1.16E-59 | 1.26 | 1.55E-03 | 1564 |
| Cluster-40555.203449 | 0.34 | 0 | 11.5 | 13.13 | 0 | 0 | 12.99 | 9.66 | 6.39 | 4.44E-32 | Inf | 1.87E-36 | 1686 |
| Cluster-40555.179897 | 1.03 | 0.46 | 49.95 | 60.59 | 31.02 | 31.89 | 87.72 | 95.72 | 6.39 | 7.24E-43 | 1.61 | 4.53E-07 | 1526 |
| Cluster-40555.165908 | 0.03 | 0 | 1.06 | 0.98 | 0.32 | 0.63 | 3.79 | 3.45 | 6.40 | 6.70E-04 | 2.96 | 3.37E-06 | 2015 |
| Cluster-40555.226160 | 0.34 | 0.09 | 18.48 | 13.86 | 0.44 | 0.46 | 10.96 | 13.22 | 6.40 | 2.96E-14 | 4.80 | 2.57E-09 | 691 |
| Cluster-40555.170045 | 0.05 | 0 | 2.56 | 1.47 | 0 | 0.06 | 1.21 | 0.97 | 6.41 | 7.88E-04 | 5.28 | 4.25E-02 | 1144 |
| Cluster-40555.217285 | 0.19 | 0 | 4.97 | 7.09 | 0 | 0.52 | 2.82 | 2.13 | 6.41 | 6.24E-08 | 3.31 | 4.14E-02 | 852 |
| Cluster-40555.178598 | 0.31 | 0.17 | 16 | 20.89 | 0.31 | 0.21 | 20.52 | 15 | 6.42 | 8.82E-18 | 6.16 | 3.96E-17 | 727 |
| Cluster-40555.184860 | 0 | 0.18 | 5.7 | 5.84 | 0 | 0 | 4.56 | 4.21 | 6.42 | 1.61E-03 | Inf | 2.64E-03 | 539 |
| Cluster-40555.153632 | 0.03 | 0 | 1.39 | 0.91 | 0 | 0.02 | 1.28 | 2.29 | 6.42 | 1.07E-07 | 7.88 | 3.40E-06 | 3421 |
| Cluster-40555.176859 | 0.6 | 0.24 | 29.59 | 33.36 | 0.6 | 0.56 | 30.37 | 30.28 | 6.42 | 7.18E-45 | 5.80 | 1.29E-35 | 1149 |

| | | | | | | | | | | | | | |
|---|---|---|---|---|---|---|---|---|---|---|---|---|---|
| Cluster-40555.203861 | 0.03 | 0 | 1.05 | 0.91 | 0 | 0 | 2.35 | 1.41 | 6.43 | 5.74E-04 | Inf | 6.58E-10 | 2116 |
| Cluster-40555.225233 | 0.27 | 0 | 8.69 | 11.24 | 0 | 0.07 | 17.4 | 13.51 | 6.43 | 2.58E-15 | 8.83 | 2.62E-26 | 985 |
| Cluster-40555.155151 | 0 | 0.43 | 15.84 | 15.64 | 0 | 0 | 6.72 | 8.51 | 6.43 | 2.39E-10 | Inf | 1.80E-06 | 572 |
| Cluster-40555.195799 | 0.03 | 0.05 | 3.33 | 2.19 | 0.67 | 1.17 | 3.91 | 4.78 | 6.43 | 1.08E-13 | 2.31 | 4.44E-06 | 2703 |
| Cluster-40555.172202 | 0 | 0.51 | 19.58 | 20.8 | 1.25 | 0 | 18.46 | 14.9 | 6.43 | 1.75E-50 | 4.84 | 2.83E-29 | 1961 |
| Cluster-40555.179932 | 0.58 | 0.38 | 34.73 | 39.02 | 3.04 | 4.3 | 19.68 | 20.22 | 6.44 | 1.27E-38 | 2.51 | 1.90E-07 | 874 |
| Cluster-40555.166217 | 0.45 | 0 | 17.67 | 16.31 | 0.74 | 0.31 | 17.18 | 6.64 | 6.45 | 9.55E-12 | 4.57 | 4.53E-02 | 585 |
| Cluster-40555.195368 | 0.32 | 0 | 12.63 | 13.46 | 0.32 | 0.36 | 22.02 | 17.02 | 6.45 | 8.61E-19 | 5.89 | 3.23E-24 | 965 |
| Cluster-40555.125267 | 0 | 0.04 | 1.19 | 0.84 | 0 | 0 | 0.64 | 0.78 | 6.45 | 1.52E-03 | Inf | 3.05E-03 | 1892 |
| Cluster-40555.182572 | 1.83 | 0.72 | 94.15 | 90.26 | 0 | 0 | 35.88 | 34.99 | 6.45 | 5.62E-15 | Inf | 8.12E-07 | 335 |
| Cluster-40555.67255 | 0.11 | 0 | 5.33 | 4.23 | 0 | 0 | 4.32 | 3.44 | 6.45 | 5.74E-08 | Inf | 1.70E-08 | 1032 |
| Cluster-40555.217671 | 0 | 0.35 | 15.08 | 13.55 | 0.15 | 0 | 6.21 | 6.78 | 6.45 | 1.46E-27 | 6.71 | 1.76E-15 | 1305 |
| Cluster-40555.230210 | 0.63 | 0 | 18.62 | 25.93 | 0 | 0 | 16.84 | 9.68 | 6.46 | 3.70E-04 | Inf | 1.27E-02 | 340 |
| Cluster-40555.186331 | 0 | 0.06 | 3.69 | 2.76 | 0 | 0 | 2.65 | 1.12 | 6.46 | 1.38E-03 | Inf | 1.29E-02 | 779 |
| Cluster-40555.188751 | 2.28 | 1.46 | 120.29 | 174.73 | 0.82 | 2.55 | 100.81 | 131.8 | 6.47 | 1.79E-15 | 6.15 | 9.16E-24 | 898 |
| Cluster-40555.159618 | 0.22 | 0 | 6.73 | 7.18 | 0 | 0.18 | 5.03 | 6.68 | 6.48 | 3.10E-08 | 6.00 | 2.81E-07 | 799 |
| Cluster-40555.119653 | 0.14 | 0 | 5.86 | 4.68 | 0 | 0 | 4.37 | 4.83 | 6.48 | 4.07E-04 | Inf | 1.94E-04 | 620 |
| Cluster-40555.207752 | 0.32 | 0 | 12.53 | 12.08 | 0 | 0.13 | 4.36 | 4.98 | 6.48 | 1.01E-15 | 5.86 | 1.55E-06 | 873 |
| Cluster-40555.205286 | 0.2 | 0 | 4.43 | 10.49 | 1.55 | 1.22 | 9.35 | 16.15 | 6.48 | 5.60E-03 | 3.27 | 4.49E-03 | 511 |
| Cluster-40555.186378 | 0.27 | 0 | 9.37 | 11.79 | 0.2 | 0 | 8.93 | 8.39 | 6.49 | 5.66E-16 | 6.56 | 5.22E-14 | 972 |
| Cluster-40555.211130 | 0 | 0.48 | 22.09 | 19.87 | 0 | 0.41 | 15.44 | 13.86 | 6.49 | 1.20E-25 | 6.27 | 1.27E-19 | 908 |
| Cluster-40555.132934 | 0.16 | 0 | 6.94 | 5.25 | 0 | 0 | 11.98 | 10.21 | 6.49 | 3.95E-04 | Inf | 2.30E-09 | 569 |
| Cluster-40555.172341 | 0.11 | 0.25 | 15.01 | 14.38 | 6.39 | 5.42 | 19.77 | 25.83 | 6.49 | 1.60E-26 | 2.02 | 1.64E-05 | 1215 |
| Cluster-40555.211404 | 0 | 0.03 | 2.04 | 2.03 | 0.06 | 0.02 | 1.18 | 1.78 | 6.50 | 9.22E-04 | 5.87 | 6.56E-03 | 1112 |
| Cluster-40555.156495 | 0.75 | 0.1 | 31.91 | 36.24 | 0.86 | 0.39 | 5.95 | 6.18 | 6.51 | 6.75E-26 | 3.36 | 4.57E-03 | 649 |
| Cluster-40555.187377 | 1.69 | 3.27 | 215.39 | 202.3 | 8.74 | 4.05 | 51.37 | 57.27 | 6.51 | 1.00E-81 | 3.16 | 3.51E-14 | 663 |
| Cluster-40555.222841 | 0.07 | 0 | 2.67 | 1.75 | 0 | 0.11 | 1.39 | 1.94 | 6.53 | 2.38E-08 | 4.93 | 5.85E-06 | 2022 |
| Cluster-40555.193206 | 1.01 | 1.16 | 96.44 | 86.45 | 27.25 | 7.87 | 149.81 | 120.53 | 6.53 | 1.54E-103 | 3.03 | 4.38E-22 | 2063 |
| Cluster-40555.186166 | 1.57 | 0.55 | 90.33 | 84.84 | 45.46 | 37.85 | 98.79 | 98.61 | 6.53 | 5.05E-79 | 1.31 | 1.27E-04 | 1124 |
| Cluster-40555.180455 | 0.35 | 0.27 | 23.09 | 28.23 | 0.35 | 0 | 8.17 | 10.77 | 6.53 | 3.62E-08 | 5.87 | 6.64E-03 | 404 |
| Cluster-40555.237877 | 0 | 0.02 | 0.92 | 0.71 | 0.24 | 0.11 | 0.86 | 1.17 | 6.54 | 8.14E-04 | 2.63 | 3.60E-02 | 2425 |
| Cluster-40555.183520 | 0.49 | 0.72 | 51.6 | 51.51 | 6.45 | 3.61 | 105.58 | 138.01 | 6.54 | 2.46E-42 | 4.67 | 6.14E-17 | 758 |

| | | | | | | | | | | | | | |
|---|---|---|---|---|---|---|---|---|---|---|---|---|---|
| Cluster-40555.203542 | 0.13 | 0 | 4.76 | 5.35 | 0.88 | 1.07 | 5.19 | 6.53 | 6.54 | 2.16E-04 | 2.68 | 2.85E-02 | 648 |
| Cluster-40555.194695 | 0 | 0.1 | 4.02 | 5.14 | 0.11 | 0 | 2.4 | 1.97 | 6.54 | 2.07E-11 | 5.29 | 2.56E-05 | 1411 |
| Cluster-40555.161991 | 0.05 | 0 | 1.76 | 2.56 | 0 | 0 | 1.59 | 2.31 | 6.54 | 1.91E-04 | Inf | 6.58E-05 | 1167 |
| Cluster-40555.203376 | 0.62 | 0 | 22.22 | 28.42 | 1.94 | 0.11 | 22.71 | 21.78 | 6.56 | 1.51E-27 | 4.53 | 4.62E-22 | 1123 |
| Cluster-40555.196377 | 1.39 | 3.86 | 204.9 | 256.97 | 62.28 | 52.32 | 132.48 | 143.43 | 6.57 | 1.30E-35 | 1.34 | 1.63E-04 | 716 |
| Cluster-40555.158078 | 0.12 | 0 | 3.3 | 4.67 | 0 | 0.11 | 3.2 | 2.62 | 6.58 | 1.43E-04 | 5.90 | 1.94E-03 | 764 |
| Cluster-40555.154527 | 0 | 0.33 | 18.1 | 17.4 | 1.57 | 1.32 | 11.14 | 18.86 | 6.58 | 1.34E-11 | 3.45 | 1.74E-03 | 567 |
| Cluster-40555.178545 | 0 | 0.13 | 6.57 | 5.56 | 0.64 | 1.18 | 6.54 | 6.84 | 6.59 | 5.60E-04 | 2.92 | 2.68E-02 | 561 |
| Cluster-40555.180182 | 0.19 | 0 | 8.15 | 7.98 | 0 | 0 | 13.78 | 14.85 | 6.59 | 4.16E-13 | Inf | 6.73E-27 | 1015 |
| Cluster-40555.200698 | 0 | 0.32 | 11.77 | 16.51 | 2.84 | 3.47 | 22.95 | 20.14 | 6.60 | 1.35E-15 | 2.83 | 1.02E-10 | 1034 |
| Cluster-40555.188070 | 0 | 0.59 | 29.34 | 25.29 | 0.24 | 0 | 16.94 | 16.54 | 6.60 | 1.13E-11 | 7.21 | 3.56E-08 | 462 |
| Cluster-40555.190270 | 0.26 | 0 | 11.28 | 10.82 | 0.49 | 0.91 | 12.4 | 9.77 | 6.61 | 2.24E-39 | 4.02 | 2.26E-21 | 2304 |
| Cluster-40555.165083 | 0.09 | 0.19 | 13.77 | 11.29 | 0.06 | 0.06 | 9.19 | 10.83 | 6.61 | 1.52E-36 | 7.52 | 5.43E-33 | 1935 |
| Cluster-40555.193724 | 0.12 | 0 | 6.57 | 4.93 | 0 | 0 | 5.86 | 7.16 | 6.61 | 4.44E-17 | Inf | 4.56E-24 | 1693 |
| Cluster-40555.260394 | 0.06 | 0 | 2.29 | 2.86 | 0.05 | 0.09 | 1.45 | 1.58 | 6.61 | 3.38E-09 | 4.39 | 2.10E-04 | 1857 |
| Cluster-40555.196872 | 0 | 0.46 | 29.23 | 14.47 | 0.87 | 0.61 | 33.2 | 27.76 | 6.61 | 4.71E-05 | 5.43 | 4.73E-10 | 431 |
| Cluster-40555.141866 | 0.04 | 0 | 1.4 | 0.97 | 0.83 | 0.54 | 4.42 | 4.5 | 6.63 | 1.39E-04 | 2.79 | 1.99E-06 | 2011 |
| Cluster-40555.201821 | 0.38 | 0.11 | 23.17 | 20.08 | 3.6 | 4.4 | 13.64 | 16.46 | 6.63 | 3.86E-48 | 1.98 | 1.09E-06 | 1670 |
| Cluster-40555.180249 | 0.17 | 0.07 | 10.76 | 10.58 | 4.67 | 4.2 | 13.39 | 14.02 | 6.63 | 4.84E-13 | 1.69 | 5.40E-03 | 828 |
| Cluster-40555.184436 | 0.28 | 0.34 | 28.03 | 26.74 | 4.81 | 7.4 | 33.12 | 28.01 | 6.64 | 1.53E-56 | 2.38 | 4.05E-12 | 1697 |
| Cluster-40555.172103 | 0.06 | 0.05 | 5.12 | 5.21 | 0.31 | 0 | 3.07 | 2.21 | 6.64 | 8.83E-09 | 4.24 | 1.61E-03 | 1034 |
| Cluster-40555.184664 | 0.25 | 0.21 | 21.73 | 19.95 | 1.48 | 1.12 | 41.06 | 32.57 | 6.65 | 8.92E-50 | 4.89 | 1.62E-39 | 1798 |
| Cluster-40555.115948 | 0.03 | 0 | 0.59 | 1.42 | 0 | 0.07 | 0.79 | 0.58 | 6.65 | 4.90E-03 | 4.29 | 1.16E-02 | 2335 |
| Cluster-40555.274223 | 0.02 | 0.08 | 3.92 | 3.69 | 1.18 | 0.59 | 3.41 | 3.58 | 6.65 | 2.78E-08 | 2.07 | 2.84E-02 | 1257 |
| Cluster-40555.141183 | 0 | 0.07 | 2.54 | 3.95 | 0.07 | 0.16 | 3.14 | 4.07 | 6.65 | 2.63E-04 | 4.89 | 3.29E-04 | 844 |
| Cluster-40555.167983 | 0.07 | 0 | 2.37 | 3.16 | 1.16 | 1.17 | 5.73 | 5.71 | 6.65 | 6.37E-14 | 2.37 | 4.59E-07 | 2546 |
| Cluster-40555.109797 | 0.02 | 0 | 1.15 | 0.95 | 0.27 | 0.19 | 1.19 | 1.13 | 6.66 | 1.01E-04 | 2.45 | 4.02E-02 | 2276 |
| Cluster-40555.190337 | 0 | 0.77 | 38.51 | 35.65 | 5.97 | 9.56 | 33.78 | 35.42 | 6.66 | 8.00E-74 | 2.21 | 1.46E-11 | 2016 |
| Cluster-40555.185836 | 0.42 | 0.17 | 28.26 | 25.34 | 0.27 | 0.5 | 20.23 | 23.1 | 6.67 | 4.36E-34 | 5.87 | 9.82E-26 | 957 |
| Cluster-40555.207320 | 0 | 0.05 | 1.88 | 2.3 | 0.07 | 0.61 | 2.79 | 2.48 | 6.67 | 1.45E-08 | 2.97 | 3.71E-05 | 2111 |
| Cluster-40555.194058 | 0.17 | 0 | 6.34 | 8.36 | 0 | 0 | 9.52 | 8.2 | 6.68 | 3.36E-14 | Inf | 1.33E-20 | 1138 |
| Cluster-40555.190074 | 0.83 | 1.5 | 106.64 | 113.29 | 41.03 | 36.06 | 79.51 | 83.18 | 6.68 | 9.94E-44 | 1.14 | 1.83E-02 | 498 |

| | | | | | | | | | | | | | |
|---|---|---|---|---|---|---|---|---|---|---|---|---|---|
| Cluster-40555.240591 | 0.15 | 0 | 7.12 | 6.54 | 0 | 0 | 15.22 | 12.49 | 6.69 | 7.15E-05 | Inf | 5.90E-12 | 577 |
| Cluster-40555.180373 | 0.54 | 0 | 23.52 | 24.42 | 9.66 | 11.65 | 34.48 | 30.27 | 6.69 | 1.10E-55 | 1.66 | 1.34E-06 | 1803 |
| Cluster-40555.160971 | 0 | 0.32 | 17.5 | 15.63 | 0.58 | 0.66 | 12.86 | 11.76 | 6.69 | 1.82E-26 | 4.39 | 1.48E-14 | 1090 |
| Cluster-40555.134822 | 0.51 | 0 | 22.24 | 19.99 | 0 | 0 | 57.28 | 49.93 | 6.70 | 4.07E-14 | Inf | 1.91E-36 | 571 |
| Cluster-40555.189913 | 0.34 | 0.07 | 18.33 | 19.48 | 0.33 | 0 | 17.86 | 25.16 | 6.70 | 5.83E-22 | 7.13 | 5.29E-15 | 826 |
| Cluster-40555.192042 | 0.05 | 0.04 | 4.03 | 4.87 | 0.51 | 0.14 | 3.55 | 4.35 | 6.70 | 1.28E-31 | 3.69 | 1.58E-14 | 3987 |
| Cluster-40555.211241 | 5.04 | 5.58 | 472.96 | 489.94 | 48.5 | 70.06 | 306.47 | 210.68 | 6.70 | 4.62E-31 | 2.12 | 2.02E-03 | 280 |
| Cluster-40555.169005 | 0 | 0.13 | 5.49 | 7.15 | 0.3 | 0.34 | 4.62 | 3.04 | 6.71 | 7.85E-09 | 3.69 | 1.49E-03 | 885 |
| Cluster-40555.183459 | 1.76 | 0.26 | 98.61 | 87.41 | 2.92 | 2.65 | 59.79 | 44.75 | 6.71 | 3.10E-73 | 4.29 | 1.27E-24 | 917 |
| Cluster-40555.184060 | 0.37 | 0 | 14.24 | 19.36 | 13.71 | 9.78 | 31.49 | 33.13 | 6.72 | 7.90E-19 | 1.53 | 1.54E-04 | 1049 |
| Cluster-40555.181278 | 1.34 | 0 | 50.21 | 56.14 | 0 | 0 | 34.2 | 60.96 | 6.72 | 4.72E-05 | Inf | 1.86E-04 | 291 |
| Cluster-40555.58895 | 0.04 | 0 | 3.52 | 2.43 | 0 | 0 | 2.63 | 1.7 | 6.73 | 5.92E-05 | Inf | 1.45E-04 | 1019 |
| Cluster-40555.174281 | 0.09 | 0 | 4.3 | 3.69 | 0 | 0.04 | 4.88 | 2.74 | 6.73 | 7.17E-10 | 7.44 | 1.35E-07 | 1380 |
| Cluster-40555.254519 | 0.33 | 0 | 15.16 | 12.85 | 1.58 | 1.24 | 14.11 | 12.79 | 6.74 | 1.72E-14 | 3.32 | 3.99E-07 | 742 |
| Cluster-40555.178649 | 0.22 | 0 | 11.89 | 11.27 | 0 | 0.25 | 19.69 | 15.94 | 6.74 | 5.42E-10 | 7.12 | 2.93E-16 | 650 |
| Cluster-40555.191307 | 1.9 | 0.68 | 118.08 | 126.75 | 4.19 | 3.3 | 203.06 | 187.09 | 6.74 | 1.27E-66 | 5.77 | 3.01E-53 | 670 |
| Cluster-40555.228656 | 0.09 | 0.03 | 4.85 | 3.82 | 0.67 | 0.91 | 11.68 | 16.83 | 6.74 | 4.87E-05 | 4.30 | 5.02E-08 | 777 |
| Cluster-40555.135546 | 0.05 | 0 | 2.06 | 2.5 | 0 | 0 | 2.47 | 1.68 | 6.74 | 3.79E-05 | Inf | 9.23E-06 | 1253 |
| Cluster-40555.194290 | 0.37 | 0 | 14.82 | 18.03 | 0 | 0.38 | 11.35 | 22.74 | 6.74 | 3.79E-05 | 6.47 | 7.67E-04 | 397 |
| Cluster-40555.151197 | 0.08 | 0 | 3.25 | 4.39 | 0.5 | 0.81 | 4.38 | 3.97 | 6.74 | 3.41E-10 | 2.72 | 1.70E-04 | 1434 |
| Cluster-40555.203987 | 0 | 0.1 | 6.01 | 7.07 | 0.46 | 0.22 | 8.99 | 9.49 | 6.75 | 4.66E-09 | 4.81 | 3.54E-10 | 882 |
| Cluster-40555.205449 | 0.15 | 0 | 9.28 | 6.5 | 0 | 0.68 | 4.27 | 5.02 | 6.75 | 6.21E-10 | 3.77 | 4.18E-04 | 837 |
| Cluster-40555.194605 | 0 | 0.26 | 13.62 | 13.03 | 0 | 0.6 | 22.21 | 17.12 | 6.77 | 5.60E-34 | 6.04 | 6.15E-38 | 1651 |
| Cluster-40555.197425 | 0 | 0.05 | 2.43 | 2.02 | 0.08 | 0.04 | 0.95 | 0.83 | 6.77 | 2.85E-13 | 4.00 | 4.89E-04 | 3050 |
| Cluster-40555.187622 | 0.5 | 0.2 | 35.49 | 31.81 | 9.22 | 10.1 | 24.55 | 31.14 | 6.77 | 2.18E-14 | 1.59 | 3.95E-02 | 459 |
| Cluster-40555.215556 | 0.13 | 0 | 5.34 | 5.36 | 0.36 | 0.32 | 7.31 | 6.92 | 6.78 | 2.30E-10 | 4.38 | 6.46E-10 | 1132 |
| Cluster-40555.180045 | 0.61 | 0 | 23.61 | 25.19 | 0 | 0 | 126.02 | 86.3 | 6.78 | 2.16E-10 | Inf | 5.04E-23 | 450 |
| Cluster-40555.187541 | 2.78 | 1.83 | 233.39 | 217.67 | 5.66 | 3.08 | 264.51 | 222.27 | 6.79 | 9.51E-50 | 5.90 | 9.53E-39 | 398 |
| Cluster-40555.203063 | 0.03 | 0.02 | 2.91 | 2.19 | 0.02 | 0.31 | 1.45 | 2.76 | 6.79 | 1.04E-09 | 3.68 | 1.57E-02 | 2006 |
| Cluster-40555.209958 | 0.27 | 0 | 11.94 | 14.17 | 0 | 0.56 | 8.52 | 9.62 | 6.79 | 1.69E-10 | 5.02 | 1.95E-06 | 617 |
| Cluster-40555.179382 | 0.71 | 0 | 33.23 | 33.63 | 11.47 | 4.89 | 36.53 | 24.59 | 6.79 | 3.12E-15 | 1.98 | 3.83E-03 | 470 |
| Cluster-40555.218704 | 0.18 | 0 | 8.09 | 9.39 | 3.63 | 4.23 | 9.19 | 12.85 | 6.79 | 2.47E-15 | 1.55 | 1.02E-02 | 1062 |

| | | | | | | | | | | | | | |
|---|---|---|---|---|---|---|---|---|---|---|---|---|---|
| Cluster-40555.141067 | 0.07 | 0 | 3.32 | 3.22 | 9.55 | 3.56 | 18.2 | 14.52 | 6.80 | 5.44E-24 | 1.40 | 1.30E-02 | 3849 |
| Cluster-40555.180962 | 0.22 | 0 | 9.92 | 11.44 | 0 | 0 | 11.14 | 9.14 | 6.81 | 1.25E-10 | Inf | 2.29E-12 | 702 |
| Cluster-40555.172682 | 0.07 | 0 | 3.45 | 3.01 | 0 | 0 | 4.37 | 2.65 | 6.81 | 1.65E-10 | Inf | 2.40E-11 | 1727 |
| Cluster-40555.216525 | 0 | 0.04 | 2.08 | 1.55 | 0 | 0 | 1.93 | 1.77 | 6.82 | 1.23E-13 | Inf | 8.15E-18 | 3785 |
| Cluster-40555.174888 | 0.05 | 0.01 | 3.02 | 3.53 | 0.32 | 0.32 | 1.29 | 1.41 | 6.83 | 8.67E-20 | 2.14 | 1.36E-02 | 3090 |
| Cluster-40555.188469 | 2.48 | 7.7 | 547.31 | 538.83 | 68.57 | 93.19 | 372.78 | 382.14 | 6.84 | 4.84E-42 | 2.22 | 1.29E-06 | 295 |
| Cluster-40555.181289 | 0 | 0.22 | 16.86 | 14.5 | 0.26 | 0 | 6.97 | 2.68 | 6.85 | 1.26E-09 | 5.92 | 3.53E-02 | 540 |
| Cluster-89857.0 | 0.1 | 0 | 4.59 | 4.98 | 0 | 0.1 | 3.6 | 3.42 | 6.86 | 1.33E-05 | 6.18 | 2.83E-04 | 771 |
| Cluster-40555.208123 | 0.16 | 0 | 9.82 | 6.33 | 2.09 | 2.52 | 8.36 | 10.18 | 6.86 | 9.43E-11 | 2.07 | 2.70E-03 | 870 |
| Cluster-40555.122727 | 0 | 0.06 | 3.36 | 2.85 | 0.27 | 0 | 2.87 | 2.88 | 6.87 | 5.95E-05 | 4.55 | 9.61E-04 | 970 |
| Cluster-40555.213836 | 0.17 | 0.14 | 18.46 | 13.83 | 0 | 0 | 5.88 | 8.95 | 6.87 | 2.64E-10 | Inf | 6.97E-06 | 551 |
| Cluster-40555.128717 | 0.09 | 0 | 4.25 | 5.17 | 0 | 0.1 | 4.17 | 4.83 | 6.87 | 1.10E-05 | 6.59 | 7.92E-06 | 783 |
| Cluster-40555.145042 | 0.09 | 0.07 | 8.76 | 8.76 | 0.18 | 0.46 | 6.2 | 4.34 | 6.87 | 1.76E-10 | 4.07 | 1.24E-04 | 796 |
| Cluster-40555.175113 | 0.68 | 0 | 32.76 | 35.25 | 0 | 0 | 14.07 | 14.29 | 6.88 | 3.24E-16 | Inf | 1.93E-08 | 479 |
| Cluster-40555.107365 | 0 | 0.02 | 1.33 | 1.34 | 0.25 | 0.32 | 2.3 | 1.43 | 6.88 | 5.06E-05 | 2.75 | 3.83E-03 | 1926 |
| Cluster-40555.220249 | 0.4 | 0 | 23.09 | 16.54 | 0 | 0 | 15.07 | 10.24 | 6.88 | 1.47E-05 | Inf | 4.88E-04 | 385 |
| Cluster-40555.203400 | 0.25 | 0.2 | 20.55 | 24.96 | 1.2 | 0.4 | 15.01 | 16.97 | 6.88 | 7.99E-41 | 4.41 | 5.86E-22 | 1501 |
| Cluster-40555.102549 | 0 | 0.1 | 4.51 | 6.77 | 0.49 | 0.73 | 7.15 | 6.06 | 6.89 | 3.83E-05 | 3.46 | 1.52E-03 | 653 |
| Cluster-40555.216630 | 0 | 0.08 | 3.99 | 5.95 | 0.41 | 0.07 | 5.55 | 6.24 | 6.89 | 1.60E-11 | 4.69 | 7.50E-13 | 1605 |
| Cluster-40555.223394 | 0.06 | 0.02 | 4.1 | 4.35 | 0 | 0 | 3.59 | 3.16 | 6.89 | 8.60E-16 | Inf | 5.29E-16 | 1970 |
| Cluster-40555.178626 | 0 | 0.15 | 8.76 | 7.37 | 0.09 | 0.18 | 3.76 | 4.24 | 6.89 | 6.24E-10 | 4.97 | 1.61E-04 | 819 |
| Cluster-40555.212414 | 0.11 | 0 | 3.67 | 6.6 | 0 | 0 | 7.3 | 6.68 | 6.89 | 1.14E-05 | Inf | 2.67E-09 | 740 |
| Cluster-40555.168625 | 0 | 0.69 | 25.72 | 51.81 | 10.32 | 14.84 | 34.67 | 32.92 | 6.89 | 4.24E-05 | 1.47 | 2.02E-02 | 491 |
| Cluster-40555.191476 | 0 | 0.1 | 5.86 | 5.9 | 0 | 0 | 1.65 | 2.79 | 6.90 | 4.57E-10 | Inf | 9.20E-05 | 1029 |
| Cluster-40555.190314 | 0.35 | 0 | 15.39 | 19.83 | 2.64 | 2.78 | 18.56 | 16.64 | 6.90 | 3.62E-26 | 2.77 | 1.15E-10 | 1260 |
| Cluster-40555.200987 | 0.04 | 0.12 | 7.89 | 8.37 | 1 | 1.71 | 6.78 | 7.26 | 6.91 | 8.86E-20 | 2.42 | 1.93E-05 | 1411 |
| Cluster-40555.190425 | 0.06 | 0.71 | 42.34 | 44.89 | 0 | 0.16 | 31.22 | 45.01 | 6.92 | 3.62E-79 | 8.96 | 8.19E-20 | 1842 |
| Cluster-40555.178770 | 1.33 | 0 | 66.24 | 62.59 | 14.09 | 11.77 | 58.82 | 77.55 | 6.92 | 2.17E-11 | 2.43 | 1.30E-03 | 336 |
| Cluster-40555.183866 | 0 | 0.09 | 5.05 | 5.06 | 2.48 | 1.95 | 7.01 | 5.09 | 6.92 | 2.11E-23 | 1.51 | 1.85E-03 | 2521 |
| Cluster-40555.192271 | 0 | 0.09 | 4.66 | 5.61 | 0.64 | 0.52 | 3.66 | 5.1 | 6.92 | 2.63E-10 | 3.03 | 3.33E-04 | 1153 |
| Cluster-40555.195495 | 0 | 0.04 | 2.04 | 2.06 | 0.08 | 0.22 | 2.35 | 2.51 | 6.92 | 3.36E-05 | 4.04 | 1.72E-04 | 1379 |
| Cluster-40555.188382 | 1.71 | 3.94 | 307.42 | 325.81 | 6.31 | 2.33 | 113 | 130.04 | 6.92 | 3.21E-118 | 4.90 | 3.26E-43 | 933 |

| | | | | | | | | | | | | | |
|---|---|---|---|---|---|---|---|---|---|---|---|---|---|
| Cluster-40555.186516 | 0 | 0.02 | 1.08 | 0.85 | 0 | 0.22 | 0.92 | 0.64 | 6.93 | 3.70E-05 | 2.81 | 3.29E-02 | 2662 |
| Cluster-40555.189263 | 0.17 | 0.55 | 39.34 | 38.42 | 0.37 | 0.34 | 5.53 | 5.55 | 6.93 | 1.57E-44 | 4.01 | 9.01E-06 | 929 |
| Cluster-40555.209185 | 0.9 | 0.21 | 54.32 | 58.3 | 2.12 | 12.98 | 80.47 | 66.21 | 6.96 | 1.45E-30 | 3.30 | 4.03E-15 | 534 |
| Cluster-40555.42815 | 0 | 0.16 | 9.97 | 9.52 | 0.07 | 0 | 1.83 | 1.22 | 6.96 | 2.54E-15 | 5.66 | 1.75E-02 | 985 |
| Cluster-40555.180736 | 0.07 | 0.05 | 7.54 | 3.06 | 0 | 0 | 1.8 | 1.15 | 6.96 | 1.13E-03 | Inf | 6.90E-04 | 1209 |
| Cluster-40555.164966 | 0 | 0.11 | 5.58 | 5.12 | 0.67 | 0.56 | 12.18 | 9.87 | 6.97 | 1.57E-10 | 4.21 | 2.70E-13 | 1145 |
| Cluster-40555.314862 | 0.05 | 0 | 3.08 | 1.95 | 0 | 0 | 2.5 | 2.02 | 6.98 | 5.64E-06 | Inf | 9.82E-07 | 1320 |
| Cluster-40555.161757 | 0 | 0.14 | 9.83 | 6.61 | 0 | 0 | 1.4 | 1.8 | 6.98 | 1.74E-10 | Inf | 1.68E-02 | 844 |
| Cluster-40555.143461 | 0.05 | 0.02 | 4.22 | 4.51 | 0.18 | 0 | 2.89 | 4.41 | 6.98 | 7.12E-17 | 5.42 | 1.36E-07 | 2024 |
| Cluster-40555.178717 | 0.05 | 0 | 3.77 | 3.68 | 0.11 | 0.56 | 4.46 | 3.41 | 6.98 | 5.41E-12 | 3.63 | 1.84E-07 | 1692 |
| Cluster-40555.191809 | 0.28 | 0 | 16.85 | 14.94 | 2.87 | 1.27 | 19.08 | 17.43 | 6.99 | 1.93E-22 | 3.23 | 9.87E-11 | 934 |
| Cluster-40555.192669 | 0.2 | 0 | 11.65 | 10.55 | 0.39 | 0.1 | 8.25 | 9.27 | 6.99 | 5.42E-12 | 5.24 | 1.99E-08 | 744 |
| Cluster-40555.189450 | 2.41 | 1.23 | 220.91 | 197.85 | 9.85 | 14.42 | 119.32 | 134.07 | 6.99 | 1.77E-108 | 3.44 | 1.87E-26 | 974 |
| Cluster-40555.147018 | 0 | 0.07 | 3.7 | 4.27 | 0.86 | 1.07 | 9.4 | 11.59 | 6.99 | 1.62E-05 | 3.50 | 1.36E-07 | 863 |
| Cluster-40555.186418 | 0.46 | 0 | 22.78 | 25.55 | 0.51 | 0 | 18.56 | 15.5 | 6.99 | 3.37E-06 | 6.31 | 4.01E-04 | 370 |
| Cluster-40555.198890 | 0.13 | 0.04 | 9.2 | 9.94 | 3.87 | 5.44 | 10.81 | 10.8 | 7.00 | 5.95E-57 | 1.27 | 6.06E-04 | 4076 |
| Cluster-40555.226866 | 0.05 | 0 | 3.19 | 2.83 | 0.06 | 0.23 | 6.69 | 5.58 | 7.00 | 3.71E-06 | 5.53 | 1.91E-11 | 1160 |
| Cluster-40555.193224 | 0.46 | 2.21 | 155.02 | 170.22 | 0.56 | 1.86 | 74.31 | 60.38 | 7.01 | 3.15E-104 | 5.84 | 8.47E-49 | 1069 |
| Cluster-40555.242304 | 0.09 | 0 | 5.17 | 4.95 | 0.09 | 0 | 2.01 | 2.19 | 7.01 | 2.96E-06 | 5.68 | 1.70E-02 | 802 |
| Cluster-40555.179211 | 0.07 | 0.06 | 7.92 | 8.13 | 0.22 | 0.28 | 6.94 | 5.61 | 7.02 | 1.12E-11 | 4.63 | 2.63E-07 | 906 |
| Cluster-40555.195443 | 0.03 | 0 | 1.49 | 2.36 | 0 | 0 | 2.87 | 2.58 | 7.02 | 1.97E-06 | Inf | 6.26E-11 | 1676 |
| Cluster-40555.175095 | 0 | 0.04 | 2.98 | 2.9 | 0 | 0.18 | 1.89 | 1.19 | 7.04 | 3.05E-16 | 4.16 | 1.56E-06 | 2797 |
| Cluster-40555.190023 | 0.26 | 0.22 | 29.47 | 27.06 | 0.26 | 0 | 14.61 | 12.7 | 7.04 | 3.87E-22 | 6.85 | 4.96E-12 | 634 |
| Cluster-40555.190511 | 0.15 | 0 | 8.57 | 7.76 | 0.61 | 3.5 | 7.91 | 9.62 | 7.05 | 1.62E-12 | 2.12 | 1.03E-03 | 941 |
| Cluster-40555.193530 | 0.85 | 0 | 50.69 | 46.48 | 1.31 | 2.65 | 21.4 | 26.76 | 7.05 | 2.30E-44 | 3.68 | 2.88E-13 | 777 |
| Cluster-40555.231805 | 0.25 | 0 | 13.58 | 14.37 | 0.49 | 1.03 | 6.85 | 9.76 | 7.05 | 1.84E-06 | 3.48 | 2.56E-02 | 460 |
| Cluster-40555.222912 | 0.1 | 0 | 6.79 | 5.18 | 0.11 | 0.53 | 4.68 | 5.19 | 7.05 | 1.84E-12 | 4.02 | 3.51E-07 | 1200 |
| Cluster-40555.154239 | 0.06 | 0.03 | 5.05 | 5.82 | 0.28 | 0.46 | 7.59 | 7.03 | 7.05 | 3.90E-32 | 4.35 | 3.34E-23 | 3235 |
| Cluster-40555.194144 | 0.22 | 0 | 14.4 | 10.91 | 4.39 | 2.99 | 17.39 | 24.53 | 7.06 | 2.09E-06 | 2.58 | 9.65E-04 | 483 |
| Cluster-40555.176429 | 0.25 | 0 | 16.8 | 13.82 | 0.18 | 0.07 | 10.54 | 9.76 | 7.06 | 4.61E-37 | 6.39 | 5.26E-25 | 1551 |
| Cluster-40555.169118 | 0.19 | 0 | 11.48 | 9.96 | 2.88 | 1.19 | 25.57 | 23.55 | 7.07 | 1.78E-06 | 3.67 | 6.43E-08 | 524 |
| Cluster-40555.181497 | 0.11 | 0 | 6.56 | 6.53 | 0.99 | 1.85 | 5.93 | 7.87 | 7.07 | 1.51E-06 | 2.33 | 2.06E-02 | 693 |

| | | | | | | | | | | | | | |
|---|---|---|---|---|---|---|---|---|---|---|---|---|---|
| Cluster-40555.204237 | 0.24 | 0.1 | 17.34 | 23.05 | 0.35 | 0 | 20.39 | 22.48 | 7.07 | 3.64E-18 | 7.04 | 3.07E-19 | 666 |
| Cluster-40555.178729 | 0 | 0.1 | 6.3 | 6.64 | 0 | 0 | 3.4 | 5.64 | 7.08 | 8.07E-17 | Inf | 2.93E-08 | 1448 |
| Cluster-40555.200664 | 0.07 | 0 | 3.95 | 4.18 | 0.68 | 0.16 | 3.5 | 3.31 | 7.09 | 1.21E-06 | 3.15 | 7.36E-03 | 963 |
| Cluster-40555.183231 | 0.5 | 0 | 34.25 | 31.18 | 2.7 | 0.29 | 23.41 | 28.26 | 7.10 | 7.52E-19 | 4.15 | 1.26E-09 | 526 |
| Cluster-40555.241837 | 0 | 0.06 | 2.45 | 3.8 | 0.98 | 0.6 | 6.22 | 6.23 | 7.10 | 4.41E-06 | 3.06 | 2.52E-05 | 1085 |
| Cluster-40555.183112 | 0.29 | 0.4 | 41.94 | 44.14 | 3.1 | 5.43 | 30.09 | 31.23 | 7.11 | 4.37E-40 | 2.90 | 1.10E-10 | 765 |
| Cluster-40555.230314 | 0.07 | 0 | 5.04 | 3.92 | 0 | 0 | 2.98 | 2.19 | 7.11 | 1.21E-06 | Inf | 1.21E-04 | 909 |
| Cluster-40555.123771 | 0 | 0.23 | 15.6 | 14.45 | 0.57 | 1.19 | 9.44 | 12.97 | 7.11 | 5.84E-06 | 3.70 | 7.82E-03 | 434 |
| Cluster-40555.194913 | 0.13 | 0.04 | 9.44 | 10.07 | 4.75 | 5.57 | 11.13 | 9.81 | 7.11 | 4.85E-29 | 1.08 | 3.73E-02 | 1718 |
| Cluster-40555.197946 | 0.39 | 0.93 | 59.53 | 81.53 | 7.99 | 1.21 | 71.84 | 70.23 | 7.11 | 5.77E-19 | 4.05 | 5.64E-15 | 450 |
| Cluster-40555.164513 | 0.23 | 0.1 | 23.06 | 24.13 | 0 | 0 | 7.86 | 7.85 | 7.12 | 1.07E-28 | Inf | 5.36E-13 | 857 |
| Cluster-40555.247585 | 0 | 0.03 | 1.91 | 1.52 | 0.06 | 0.07 | 3.56 | 2.05 | 7.13 | 5.15E-06 | 5.55 | 8.58E-07 | 1810 |
| Cluster-40555.169629 | 0 | 0.2 | 17.48 | 13.17 | 1.03 | 1.39 | 11.79 | 9.71 | 7.13 | 4.15E-22 | 3.19 | 1.17E-07 | 955 |
| Cluster-40555.195795 | 0.21 | 0.6 | 52.99 | 51.59 | 42.89 | 50.28 | 96.19 | 93.81 | 7.14 | 3.12E-89 | 1.09 | 1.41E-03 | 1879 |
| Cluster-40555.231193 | 0 | 0.13 | 8.28 | 9 | 0.2 | 0 | 10.72 | 8.28 | 7.15 | 1.18E-17 | 6.67 | 3.91E-19 | 1192 |
| Cluster-40555.157954 | 0.52 | 0 | 31.35 | 31.55 | 0.26 | 0 | 27.35 | 16.41 | 7.15 | 1.38E-13 | 7.51 | 2.97E-08 | 451 |
| Cluster-40555.181659 | 0.09 | 0 | 3.98 | 7.32 | 0 | 0 | 1.46 | 3.11 | 7.15 | 1.55E-05 | Inf | 5.41E-03 | 787 |
| Cluster-40555.129190 | 0.04 | 0 | 2.86 | 2.24 | 0.04 | 0 | 1.11 | 1.66 | 7.16 | 6.00E-07 | 6.26 | 6.09E-04 | 1448 |
| Cluster-40555.178793 | 0.08 | 0 | 5.49 | 4.31 | 0.76 | 0.48 | 6.26 | 5.1 | 7.16 | 6.00E-07 | 3.26 | 3.09E-04 | 879 |
| Cluster-40555.223110 | 0.18 | 0 | 9.43 | 12.61 | 0 | 0 | 8.89 | 2.97 | 7.17 | 3.72E-07 | Inf | 3.03E-02 | 535 |
| Cluster-40555.169854 | 0.11 | 0.07 | 8.28 | 12.07 | 5.92 | 6.54 | 13.33 | 16.35 | 7.17 | 9.51E-13 | 1.32 | 3.87E-02 | 825 |
| Cluster-40555.189567 | 0.75 | 0 | 55.59 | 34.81 | 3.37 | 1.95 | 49.28 | 62.48 | 7.17 | 5.16E-12 | 4.46 | 5.46E-11 | 395 |
| Cluster-40555.197712 | 0.6 | 0 | 37.14 | 40.12 | 0.18 | 0.35 | 47.89 | 42.47 | 7.17 | 1.13E-51 | 7.39 | 4.75E-50 | 1047 |
| Cluster-40555.189762 | 0 | 0.05 | 2.66 | 2.38 | 0.63 | 0.78 | 3.87 | 3.9 | 7.18 | 2.80E-06 | 2.52 | 1.48E-03 | 1352 |
| Cluster-40555.189412 | 0.62 | 0.27 | 49.21 | 63.03 | 8.12 | 0.18 | 50.95 | 67.32 | 7.18 | 5.57E-34 | 3.95 | 2.16E-10 | 1933 |
| Cluster-40555.188259 | 0.09 | 0 | 5.9 | 3.87 | 0.58 | 0.85 | 3.17 | 4.36 | 7.18 | 5.83E-07 | 2.41 | 4.27E-02 | 892 |
| Cluster-40555.167101 | 4.04 | 0 | 197.68 | 276.31 | 10.6 | 5.31 | 151.69 | 104.34 | 7.19 | 6.79E-20 | 4.07 | 1.48E-09 | 389 |
| Cluster-40555.201220 | 0.83 | 0.2 | 64.51 | 70.06 | 4.03 | 1.74 | 134.47 | 112.59 | 7.19 | 1.02E-47 | 5.50 | 3.05E-43 | 663 |
| Cluster-40555.187904 | 0 | 3.14 | 203.25 | 266.46 | 0 | 0 | 170.22 | 234.36 | 7.19 | 1.74E-06 | Inf | 6.68E-05 | 237 |
| Cluster-40555.246002 | 0.03 | 0 | 2.21 | 2.41 | 0 | 0.04 | 2.53 | 2.05 | 7.20 | 3.07E-07 | 6.98 | 8.08E-08 | 1598 |
| Cluster-40555.207734 | 0.06 | 0.04 | 8.37 | 7.39 | 0.2 | 0.2 | 5.77 | 3.95 | 7.21 | 1.42E-19 | 4.59 | 1.52E-09 | 1405 |
| Cluster-40555.187890 | 0.18 | 0 | 10.72 | 11.65 | 0 | 0.09 | 12.54 | 11.88 | 7.21 | 6.11E-41 | 8.35 | 2.46E-43 | 2204 |

| | | | | | | | | | | | | | |
|---|---|---|---|---|---|---|---|---|---|---|---|---|---|
| Cluster-40555.173520 | 0.17 | 0 | 12.24 | 14.52 | 0.46 | 0 | 35.19 | 18.28 | 7.21 | 3.00E-14 | 6.82 | 1.27E-06 | 726 |
| Cluster-40555.164835 | 0.26 | 0.21 | 34.67 | 29.29 | 4.2 | 4.03 | 18.88 | 18.58 | 7.23 | 7.31E-35 | 2.25 | 1.19E-05 | 824 |
| Cluster-40555.179690 | 1.46 | 0.57 | 106.58 | 158.19 | 6.22 | 3.04 | 79.62 | 76.88 | 7.23 | 1.81E-14 | 4.15 | 3.19E-13 | 398 |
| Cluster-40555.166379 | 0.76 | 0 | 52.16 | 49.06 | 0.38 | 1.25 | 49.35 | 44.36 | 7.23 | 1.83E-83 | 5.89 | 3.73E-52 | 1637 |
| Cluster-40555.134440 | 0 | 0.02 | 1.68 | 1.02 | 0.31 | 0.16 | 1.57 | 1.05 | 7.24 | 1.74E-06 | 2.58 | 1.27E-02 | 2400 |
| Cluster-40555.194368 | 0.26 | 0 | 13.81 | 19.07 | 2.52 | 2.82 | 28.92 | 24.34 | 7.24 | 2.83E-18 | 3.37 | 1.29E-15 | 1034 |
| Cluster-40555.187146 | 8.35 | 0 | 544 | 562.25 | 45.96 | 51.38 | 778.38 | 960.77 | 7.25 | 4.26E-144 | 4.22 | 1.81E-22 | 1027 |
| Cluster-40555.199476 | 0 | 0.36 | 31.72 | 21.78 | 0 | 0 | 3.13 | 7.22 | 7.26 | 6.96E-13 | Inf | 1.09E-02 | 478 |
| Cluster-40555.193651 | 0.08 | 0 | 4.74 | 4.92 | 1.16 | 1.01 | 5.9 | 5.26 | 7.26 | 3.38E-21 | 2.43 | 1.11E-06 | 2264 |
| Cluster-40555.188116 | 0.21 | 0 | 12.88 | 14.83 | 0.07 | 0 | 17.45 | 27.86 | 7.26 | 2.41E-21 | 9.52 | 1.81E-11 | 960 |
| Cluster-40555.203541 | 0.28 | 0 | 19.43 | 19.74 | 6.77 | 3.88 | 21.94 | 21.47 | 7.27 | 6.80E-77 | 2.11 | 2.19E-10 | 3240 |
| Cluster-40555.251060 | 0 | 0.02 | 2.08 | 1.17 | 0 | 0 | 1.34 | 1.18 | 7.27 | 1.40E-06 | Inf | 1.60E-06 | 2075 |
| Cluster-40555.195371 | 0.33 | 0.26 | 31.41 | 50.44 | 0 | 0 | 39.59 | 61.34 | 7.28 | 2.47E-09 | Inf | 1.65E-12 | 411 |
| Cluster-40555.150024 | 0.05 | 0 | 3.08 | 3.14 | 0 | 0.05 | 3.91 | 4.45 | 7.30 | 9.43E-08 | 7.51 | 1.92E-11 | 1323 |
| Cluster-40555.189156 | 5.21 | 3.23 | 598.95 | 603.5 | 137.3 | 124.71 | 366.45 | 512.27 | 7.30 | 2.04E-144 | 1.82 | 1.77E-03 | 991 |
| Cluster-40555.174815 | 0.13 | 0.11 | 16.78 | 17.04 | 4.93 | 0.55 | 39.62 | 51.08 | 7.32 | 1.53E-14 | 4.16 | 3.58E-14 | 639 |
| Cluster-40555.172292 | 0 | 0.01 | 1.14 | 0.99 | 0.13 | 0 | 1.68 | 1.44 | 7.33 | 4.99E-07 | 4.69 | 5.79E-08 | 3114 |
| Cluster-40555.190851 | 1.24 | 0 | 96.63 | 76.35 | 0 | 0.16 | 58.55 | 53.24 | 7.33 | 8.09E-52 | 9.63 | 1.13E-39 | 614 |
| Cluster-40555.175714 | 0.51 | 0.39 | 68.8 | 65.15 | 0 | 6.44 | 84.18 | 84.1 | 7.33 | 3.47E-74 | 4.72 | 6.55E-41 | 1081 |
| Cluster-40555.87057 | 0.11 | 0 | 8.3 | 7.5 | 0.78 | 0 | 16.79 | 13.13 | 7.33 | 6.21E-08 | 5.38 | 2.89E-12 | 690 |
| Cluster-40555.217162 | 0.14 | 0 | 10.8 | 8.94 | 3.11 | 0 | 16.52 | 21.19 | 7.35 | 5.64E-08 | 3.68 | 6.44E-08 | 608 |
| Cluster-40555.187744 | 0.11 | 0 | 6.79 | 7.74 | 0.32 | 0.11 | 12.37 | 9.75 | 7.35 | 3.98E-08 | 5.85 | 3.90E-11 | 734 |
| Cluster-40555.200894 | 1.37 | 0 | 78.67 | 91.15 | 12.39 | 14.07 | 97.61 | 90.14 | 7.35 | 6.00E-29 | 2.87 | 4.73E-10 | 422 |
| Cluster-40555.186721 | 3.95 | 2.6 | 488.02 | 483.33 | 275.14 | 253.07 | 431.83 | 564.38 | 7.36 | 8.78E-139 | 0.99 | 2.98E-02 | 941 |
| Cluster-40555.220745 | 0.06 | 0 | 4.24 | 5.06 | 0.98 | 1.05 | 18.67 | 13.99 | 7.36 | 3.19E-08 | 4.04 | 8.48E-15 | 1007 |
| Cluster-40555.205018 | 0.09 | 0 | 6.33 | 6.08 | 0.39 | 0.33 | 3.94 | 4.39 | 7.37 | 1.10E-22 | 3.63 | 1.22E-08 | 1938 |
| Cluster-40555.176187 | 0 | 0.21 | 20.44 | 15.04 | 0.19 | 0.66 | 7.17 | 4.72 | 7.38 | 2.30E-31 | 3.80 | 1.79E-07 | 1152 |
| Cluster-40555.116184 | 0.06 | 0 | 4.46 | 4.75 | 0 | 0.07 | 2.27 | 2.9 | 7.38 | 2.80E-08 | 6.33 | 8.79E-05 | 1021 |
| Cluster-40555.180402 | 0.1 | 0 | 9.65 | 8.42 | 0.31 | 0.24 | 8.32 | 5.95 | 7.39 | 5.22E-23 | 4.71 | 1.23E-13 | 1435 |
| Cluster-40555.148617 | 0.03 | 0 | 2.36 | 1.59 | 0.12 | 0.41 | 1.53 | 1.31 | 7.40 | 3.40E-08 | 2.49 | 2.21E-02 | 2056 |
| Cluster-40555.157418 | 0 | 0.04 | 3.18 | 3.67 | 0.1 | 0 | 4.23 | 4.17 | 7.40 | 1.63E-07 | 6.50 | 2.33E-09 | 1193 |
| Cluster-40555.187090 | 0.44 | 0.29 | 56.31 | 53.66 | 0.77 | 0.71 | 52.73 | 46.13 | 7.40 | 2.38E-52 | 6.12 | 1.02E-37 | 817 |

| | | | | | | | | | | | | | |
|---|---|---|---|---|---|---|---|---|---|---|---|---|---|
| Cluster-40555.215906 | 0.19 | 0.16 | 27.77 | 24.51 | 0.93 | 0.73 | 17.22 | 14.58 | 7.40 | 9.20E-52 | 4.34 | 6.96E-21 | 1420 |
| Cluster-40555.141246 | 0.04 | 0 | 2.37 | 2.98 | 0 | 0 | 4.57 | 3.17 | 7.41 | 1.68E-08 | Inf | 2.74E-14 | 1592 |
| Cluster-40555.186001 | 0.69 | 0.34 | 75.8 | 81.03 | 10.66 | 8.67 | 34.15 | 38.11 | 7.41 | 2.37E-75 | 1.97 | 4.59E-07 | 963 |
| Cluster-40555.183316 | 0.44 | 3.06 | 292.69 | 252.95 | 0.43 | 0.45 | 35.39 | 22.74 | 7.41 | 1.97E-98 | 6.25 | 2.19E-12 | 631 |
| Cluster-40555.196840 | 0.08 | 0.22 | 28.18 | 21.88 | 1 | 1.95 | 29.06 | 29.49 | 7.42 | 6.44E-33 | 4.36 | 9.65E-22 | 923 |
| Cluster-40555.237695 | 0.15 | 0.04 | 13.38 | 15.21 | 0.14 | 0.1 | 25.22 | 25.85 | 7.42 | 1.58E-29 | 7.79 | 6.97E-43 | 1243 |
| Cluster-40555.174611 | 0 | 0.01 | 1.98 | 1.75 | 0.34 | 0.15 | 1.6 | 1.47 | 7.43 | 1.43E-07 | 2.74 | 1.07E-02 | 2006 |
| Cluster-40555.112583 | 0.06 | 0 | 4.51 | 3.46 | 0.49 | 0.67 | 5.3 | 5.92 | 7.43 | 2.67E-16 | 3.34 | 2.96E-10 | 2083 |
| Cluster-40555.184065 | 0 | 0.16 | 15.25 | 11.3 | 8.48 | 4.13 | 23.03 | 24.65 | 7.43 | 1.58E-07 | 2.00 | 4.65E-03 | 507 |
| Cluster-40555.188952 | 0.17 | 0.55 | 57.24 | 58.26 | 0.34 | 0.54 | 86.65 | 97.78 | 7.43 | 1.28E-33 | 7.80 | 9.59E-44 | 550 |
| Cluster-40555.237671 | 0.05 | 0 | 3.95 | 3.14 | 0 | 0 | 1.27 | 1.92 | 7.44 | 1.53E-08 | Inf | 1.32E-04 | 1296 |
| Cluster-40555.213069 | 0.06 | 0 | 4.53 | 3.85 | 0.13 | 0.1 | 4.59 | 5.09 | 7.44 | 1.43E-08 | 5.52 | 6.00E-09 | 1135 |
| Cluster-40555.168944 | 0.07 | 0 | 5.5 | 5.44 | 0 | 0 | 4.54 | 2.18 | 7.45 | 1.11E-08 | Inf | 1.18E-04 | 933 |
| Cluster-40555.158430 | 0.1 | 0 | 8.46 | 7.62 | 0.23 | 0.62 | 7.63 | 8.14 | 7.45 | 1.64E-30 | 4.27 | 5.54E-18 | 2094 |
| Cluster-40555.112821 | 0 | 0.03 | 2.48 | 2.32 | 0 | 0 | 1.03 | 1.37 | 7.46 | 8.63E-08 | Inf | 9.47E-05 | 1656 |
| Cluster-40555.193860 | 0.15 | 0.19 | 29.78 | 27.2 | 0 | 6.82 | 77.53 | 102.62 | 7.48 | 1.32E-57 | 4.75 | 2.97E-15 | 1497 |
| Cluster-40555.198319 | 0 | 0.22 | 16.75 | 20.11 | 0 | 0.46 | 6.54 | 9.26 | 7.49 | 1.99E-22 | 5.15 | 9.31E-09 | 813 |
| Cluster-40555.228459 | 0.14 | 0 | 10.4 | 11.93 | 1.18 | 0 | 6.02 | 6.9 | 7.49 | 2.44E-17 | 3.56 | 1.49E-05 | 937 |
| Cluster-40555.144953 | 0.09 | 0.06 | 11.84 | 15.94 | 0.32 | 0.48 | 15.25 | 25.26 | 7.49 | 2.66E-21 | 5.73 | 6.14E-07 | 1292 |
| Cluster-40555.141064 | 0 | 0.04 | 3.62 | 2.66 | 0.05 | 0 | 1.47 | 1.49 | 7.50 | 6.67E-08 | 6.22 | 7.43E-04 | 1356 |
| Cluster-40555.183291 | 0.04 | 0 | 2.87 | 3.86 | 0.02 | 0 | 2.82 | 3.14 | 7.51 | 9.63E-18 | 8.31 | 2.84E-17 | 2532 |
| Cluster-40555.139464 | 0.15 | 0 | 9.4 | 13.71 | 0.52 | 0.15 | 12.89 | 17.86 | 7.52 | 1.07E-14 | 5.63 | 1.84E-13 | 1257 |
| Cluster-40555.193884 | 0.05 | 0 | 5.29 | 3.5 | 0.05 | 0.17 | 2.57 | 1.76 | 7.53 | 5.18E-09 | 4.33 | 1.67E-03 | 1147 |
| Cluster-40555.174123 | 0.39 | 0.48 | 72.34 | 73.09 | 2.13 | 1.22 | 75.03 | 53.92 | 7.53 | 2.80E-36 | 5.33 | 3.66E-19 | 511 |
| Cluster-40555.188448 | 0.29 | 0 | 28.76 | 20.75 | 1.41 | 1.43 | 9.13 | 15.15 | 7.53 | 5.00E-30 | 3.17 | 2.43E-03 | 890 |
| Cluster-40555.149017 | 0.09 | 0 | 7.39 | 6.96 | 1.05 | 1.34 | 5.42 | 9.09 | 7.53 | 3.40E-09 | 2.65 | 1.08E-02 | 803 |
| Cluster-40555.189766 | 0.71 | 0 | 60.98 | 52.14 | 22.39 | 27.28 | 70.89 | 73.22 | 7.54 | 8.86E-54 | 1.59 | 9.65E-06 | 814 |
| Cluster-40555.133065 | 0.15 | 0 | 12.4 | 11.68 | 0 | 0 | 3.98 | 4.47 | 7.55 | 2.56E-09 | Inf | 1.10E-03 | 588 |
| Cluster-40555.145962 | 0.13 | 0 | 9.89 | 11.17 | 0.43 | 1.04 | 11.01 | 13.38 | 7.55 | 4.08E-18 | 4.08 | 2.22E-12 | 1011 |
| Cluster-40555.185094 | 0.06 | 0 | 4.15 | 5.28 | 0.12 | 0.15 | 8.73 | 7.41 | 7.55 | 5.01E-26 | 5.97 | 1.55E-31 | 2770 |
| Cluster-40555.188093 | 0.53 | 0.17 | 47.81 | 68.72 | 31.57 | 13.63 | 53.77 | 75.33 | 7.56 | 2.82E-19 | 1.61 | 4.50E-02 | 1712 |
| Cluster-40555.174184 | 0.78 | 0 | 56.75 | 60.07 | 0 | 1.62 | 39.01 | 52.61 | 7.57 | 1.70E-09 | 5.78 | 3.81E-06 | 324 |

| | | | | | | | | | | | | | |
|---|---|---|---|---|---|---|---|---|---|---|---|---|---|
| Cluster-40555.187884 | 1.39 | 0.73 | 172.14 | 189.66 | 2.22 | 1.18 | 75.36 | 70.74 | 7.57 | 5.23E-152 | 5.51 | 1.61E-60 | 3295 |
| Cluster-40555.204181 | 0.61 | 0.13 | 69.99 | 54.47 | 7.17 | 11.53 | 26.51 | 26.8 | 7.58 | 1.93E-61 | 1.57 | 1.73E-04 | 1017 |
| Cluster-40555.141741 | 0.08 | 0 | 6.48 | 7.58 | 0.58 | 0.17 | 5.8 | 8.76 | 7.58 | 1.28E-09 | 4.37 | 3.27E-06 | 832 |
| Cluster-40555.188750 | 0 | 0.27 | 22.46 | 24.02 | 0.37 | 1.47 | 10.13 | 9.67 | 7.58 | 1.53E-69 | 3.46 | 1.95E-16 | 2288 |
| Cluster-40555.241276 | 0.13 | 0 | 8.07 | 6.33 | 1.08 | 0.75 | 5.14 | 5.77 | 7.59 | 1.55E-09 | 2.68 | 6.17E-03 | 823 |
| Cluster-40555.188289 | 0.25 | 0 | 21.27 | 21.7 | 0.74 | 3.56 | 16.17 | 18.16 | 7.61 | 8.94E-69 | 3.04 | 3.07E-17 | 2364 |
| Cluster-40555.179311 | 0.17 | 0.28 | 38.76 | 39.8 | 0.65 | 1.19 | 38.42 | 39.89 | 7.61 | 1.42E-43 | 5.47 | 8.42E-31 | 846 |
| Cluster-40555.179744 | 11.79 | 0 | 867.87 | 804.78 | 695.45 | 743.8 | 1351.38 | 1401.01 | 7.61 | 4.17E-46 | 0.93 | 4.02E-02 | 273 |
| Cluster-40555.184638 | 4.81 | 2.93 | 690.94 | 679.66 | 154.66 | 156.33 | 320.51 | 283.32 | 7.63 | 5.09E-133 | 1.02 | 5.07E-03 | 597 |
| Cluster-40555.188496 | 5.26 | 3.53 | 805.93 | 766.87 | 154.2 | 166.24 | 646.23 | 689.98 | 7.63 | 1.33E-166 | 2.13 | 2.50E-13 | 1632 |
| Cluster-40555.149339 | 0.29 | 0 | 25.5 | 30.28 | 2.06 | 2.76 | 27.5 | 24.02 | 7.63 | 1.49E-34 | 3.48 | 6.20E-14 | 857 |
| Cluster-40555.187529 | 3.31 | 0.28 | 275.82 | 354.8 | 9.31 | 7.85 | 401.1 | 487.87 | 7.64 | 6.84E-38 | 5.77 | 1.68E-36 | 1767 |
| Cluster-40555.182937 | 0.18 | 0.04 | 17.34 | 19.62 | 0.21 | 0.09 | 18.69 | 13.85 | 7.64 | 1.30E-46 | 6.93 | 1.07E-35 | 1602 |
| Cluster-40555.189292 | 0.18 | 0 | 18.17 | 23.31 | 11.25 | 5.97 | 24.09 | 19.13 | 7.65 | 2.12E-19 | 1.40 | 2.38E-02 | 666 |
| Cluster-40555.201814 | 0.17 | 0 | 17.42 | 11.63 | 0.16 | 0.17 | 7.5 | 6.93 | 7.65 | 1.11E-17 | 5.50 | 7.18E-09 | 845 |
| Cluster-40555.200540 | 0.12 | 0 | 12.76 | 11.26 | 0.15 | 0 | 11.19 | 15.35 | 7.67 | 1.36E-47 | 7.57 | 1.57E-19 | 2409 |
| Cluster-40555.176467 | 0 | 0.02 | 3.8 | 4.29 | 2.29 | 2.18 | 7.54 | 7.47 | 7.68 | 3.22E-09 | 1.82 | 3.55E-03 | 1212 |
| Cluster-40555.193611 | 0.31 | 0 | 21.16 | 30.27 | 12.71 | 13.97 | 79.04 | 81.11 | 7.68 | 2.94E-15 | 2.64 | 6.55E-12 | 596 |
| Cluster-40555.164104 | 0.07 | 0 | 7.54 | 5.9 | 0 | 0.77 | 12.36 | 10.8 | 7.69 | 3.27E-10 | 4.93 | 2.78E-13 | 907 |
| Cluster-40555.219386 | 0.21 | 0 | 15.65 | 17.82 | 0 | 0 | 6.78 | 6.38 | 7.69 | 2.63E-28 | Inf | 3.40E-14 | 1038 |
| Cluster-40555.191321 | 0.05 | 0.05 | 6.93 | 7.89 | 0 | 0 | 6.25 | 4.97 | 7.69 | 6.36E-19 | Inf | 4.08E-17 | 1374 |
| Cluster-40555.193380 | 0.3 | 0.06 | 32.64 | 33.62 | 0.37 | 0.15 | 22.12 | 20.77 | 7.69 | 1.03E-41 | 6.45 | 5.69E-26 | 905 |
| Cluster-40555.189872 | 0.5 | 0.5 | 84.64 | 107.81 | 0.74 | 0.63 | 287.79 | 316.32 | 7.70 | 6.22E-38 | 8.85 | 1.54E-109 | 1372 |
| Cluster-40555.195285 | 0 | 0.17 | 16.32 | 18.65 | 0.1 | 0.07 | 8.2 | 9.18 | 7.70 | 9.47E-45 | 6.70 | 5.52E-24 | 1609 |
| Cluster-40555.156998 | 0.17 | 0.09 | 25.28 | 23.76 | 3.98 | 2.41 | 34.74 | 34.45 | 7.71 | 3.82E-41 | 3.51 | 1.70E-19 | 1122 |
| Cluster-40555.156714 | 0.07 | 0 | 3.79 | 7.07 | 0 | 0.09 | 1.82 | 1.23 | 7.72 | 4.48E-06 | 5.65 | 8.05E-03 | 1072 |
| Cluster-40555.185964 | 0.06 | 0 | 6.87 | 6.35 | 0.05 | 0.21 | 4.14 | 4.22 | 7.72 | 3.59E-20 | 4.89 | 2.17E-10 | 1606 |
| Cluster-40555.174134 | 0.11 | 0 | 7.45 | 9.55 | 0.33 | 0.54 | 7.2 | 9.86 | 7.74 | 1.24E-20 | 4.43 | 1.41E-10 | 1315 |
| Cluster-40555.203890 | 0.09 | 0.38 | 43.28 | 50.56 | 0 | 0 | 27.12 | 25.62 | 7.74 | 1.41E-64 | Inf | 9.39E-52 | 1316 |
| Cluster-40555.190102 | 2.44 | 0.95 | 342.55 | 295.86 | 14.74 | 8.08 | 268.34 | 136.62 | 7.76 | 1.47E-63 | 4.21 | 2.30E-03 | 384 |
| Cluster-40555.190484 | 0.1 | 0 | 9.41 | 10.78 | 0.14 | 1.07 | 5.75 | 5.9 | 7.76 | 6.56E-21 | 3.25 | 3.79E-06 | 1164 |
| Cluster-40555.143770 | 0.39 | 0.13 | 46.76 | 55.27 | 0.18 | 0.32 | 60.38 | 89.54 | 7.78 | 3.75E-55 | 8.35 | 1.47E-16 | 874 |

| | | | | | | | | | | | | | |
|---|---|---|---|---|---|---|---|---|---|---|---|---|---|
| Cluster-40555.182998 | 0 | 0.06 | 9.19 | 3.4 | 1.21 | 0.96 | 5.63 | 6.36 | 7.79 | 3.09E-03 | 2.54 | 2.17E-03 | 929 |
| Cluster-40555.160249 | 0.49 | 0 | 43.27 | 50.83 | 0.19 | 0 | 63.1 | 64.86 | 7.80 | 2.38E-45 | 9.51 | 1.32E-52 | 758 |
| Cluster-40555.214336 | 0.07 | 0.06 | 12.15 | 12.49 | 0 | 0 | 2.73 | 3.82 | 7.81 | 2.22E-20 | Inf | 1.07E-06 | 986 |
| Cluster-40555.189917 | 0.14 | 0 | 13.34 | 12.56 | 0.16 | 0 | 7.76 | 7.39 | 7.81 | 3.01E-11 | 6.96 | 8.73E-07 | 625 |
| Cluster-40555.185874 | 0.74 | 1.08 | 199.64 | 173.66 | 2.98 | 1.53 | 227.91 | 141.29 | 7.83 | 1.12E-28 | 6.41 | 9.31E-09 | 328 |
| Cluster-40555.206168 | 0.04 | 0 | 2.75 | 2.87 | 0.09 | 0.39 | 2.86 | 1.64 | 7.83 | 1.77E-11 | 3.24 | 1.01E-03 | 1967 |
| Cluster-40555.188754 | 3.12 | 0.06 | 338.34 | 299.47 | 11.74 | 0.89 | 262.74 | 313.62 | 7.84 | 5.81E-134 | 5.62 | 6.56E-41 | 927 |
| Cluster-40555.206578 | 0.24 | 0.1 | 33.68 | 36.54 | 2.11 | 2.1 | 44.72 | 39.15 | 7.85 | 1.65E-51 | 4.38 | 2.79E-28 | 1061 |
| Cluster-40555.138326 | 0.09 | 0 | 11.51 | 10.86 | 0 | 0 | 10.5 | 9.03 | 7.86 | 3.95E-22 | Inf | 2.56E-22 | 1136 |
| Cluster-40555.172905 | 0.37 | 0 | 37.99 | 36.11 | 0 | 0.19 | 15.13 | 10.92 | 7.86 | 3.53E-22 | 7.09 | 1.69E-08 | 525 |
| Cluster-40555.205148 | 0.07 | 0 | 6.5 | 7.85 | 0.07 | 0 | 8.71 | 10.2 | 7.87 | 7.38E-12 | 8.22 | 2.56E-16 | 944 |
| Cluster-40555.91178 | 0.05 | 0 | 5.35 | 5.77 | 0 | 0 | 6.5 | 6.42 | 7.88 | 7.14E-12 | Inf | 7.91E-16 | 1150 |
| Cluster-40555.193425 | 0.53 | 0 | 51.09 | 57.16 | 25.84 | 16.29 | 37.43 | 43.9 | 7.88 | 8.29E-70 | 1.03 | 2.00E-02 | 1082 |
| Cluster-40555.234163 | 0.05 | 0 | 5.36 | 5.21 | 0.11 | 0.06 | 4.59 | 4.7 | 7.88 | 1.81E-22 | 6.02 | 8.96E-18 | 2135 |
| Cluster-40555.187852 | 0.42 | 0.1 | 57.51 | 50.67 | 3.63 | 4.68 | 29.8 | 33.29 | 7.88 | 3.82E-98 | 2.99 | 2.09E-18 | 1878 |
| Cluster-40555.168836 | 1.27 | 0 | 104.06 | 154.18 | 1.53 | 0.29 | 90.56 | 111.09 | 7.89 | 4.62E-17 | 6.89 | 2.47E-39 | 606 |
| Cluster-40555.183313 | 0.07 | 0.06 | 13.96 | 12.9 | 0 | 0 | 9.21 | 9.06 | 7.91 | 1.00E-21 | Inf | 1.05E-17 | 976 |
| Cluster-40555.205612 | 0.09 | 0 | 8.79 | 9.86 | 0.38 | 0.56 | 8.92 | 7.41 | 7.91 | 3.38E-12 | 4.23 | 3.66E-07 | 803 |
| Cluster-40555.37615 | 0 | 0.04 | 5.22 | 4.57 | 0.05 | 0 | 1.42 | 0.85 | 7.91 | 8.24E-11 | 5.59 | 2.38E-02 | 1192 |
| Cluster-40555.198188 | 0.05 | 0 | 5.52 | 4.57 | 0.3 | 0.24 | 1.48 | 2.45 | 7.91 | 4.77E-12 | 2.97 | 2.64E-02 | 1268 |
| Cluster-40555.188940 | 2.5 | 0.02 | 229.21 | 305.19 | 1.26 | 0.88 | 302.16 | 346.13 | 7.91 | 1.35E-31 | 8.32 | 6.57E-96 | 1224 |
| Cluster-40555.184207 | 0.05 | 0.04 | 11.97 | 10.54 | 0.29 | 0.14 | 8.91 | 8.25 | 7.91 | 8.73E-39 | 5.36 | 3.28E-23 | 1975 |
| Cluster-40555.153237 | 0.11 | 0 | 12.2 | 11.18 | 0.02 | 0.06 | 9.69 | 4.58 | 7.92 | 4.56E-23 | 7.99 | 1.92E-05 | 1135 |
| Cluster-40555.234261 | 0.03 | 0 | 3.31 | 3.31 | 0.15 | 0.22 | 2.87 | 1.7 | 7.93 | 2.80E-12 | 3.66 | 4.62E-05 | 1810 |
| Cluster-40555.186815 | 9.39 | 0.37 | 926.77 | 1139.71 | 3.2 | 3.45 | 615.41 | 1368.26 | 7.93 | 4.18E-55 | 8.29 | 2.58E-05 | 808 |
| Cluster-40555.196931 | 1.15 | 0.15 | 134.42 | 148.32 | 34.07 | 25.27 | 71.89 | 85.8 | 7.94 | 7.36E-104 | 1.48 | 2.92E-05 | 943 |
| Cluster-40555.204183 | 0 | 0.13 | 11.61 | 12.99 | 1.49 | 2.28 | 7.58 | 11.75 | 7.95 | 2.36E-21 | 2.42 | 5.56E-03 | 1020 |
| Cluster-40555.185381 | 0.07 | 0 | 5.82 | 8.34 | 0.06 | 0 | 9.27 | 15.67 | 7.95 | 1.07E-15 | 8.74 | 2.91E-09 | 1727 |
| Cluster-40555.200809 | 0.46 | 0 | 43.52 | 33.72 | 0.61 | 2.18 | 21.29 | 18.87 | 7.96 | 2.13E-12 | 3.73 | 7.95E-04 | 396 |
| Cluster-40555.190522 | 0.17 | 0 | 21.43 | 16.16 | 4.25 | 1.69 | 16.5 | 19.48 | 7.96 | 2.29E-41 | 2.70 | 1.28E-10 | 1378 |
| Cluster-40555.172361 | 0.27 | 0.21 | 46.14 | 50.08 | 0.16 | 0.27 | 68.68 | 52.55 | 7.97 | 3.43E-54 | 8.33 | 9.22E-53 | 884 |
| Cluster-40555.204210 | 0.08 | 0 | 10.67 | 7.09 | 2.7 | 3.05 | 10.02 | 9.35 | 7.97 | 3.77E-21 | 1.82 | 4.85E-06 | 2649 |

| | | | | | | | | | | | | | |
|---|---|---|---|---|---|---|---|---|---|---|---|---|---|
| Cluster-40555.234556 | 0.13 | 0 | 12.81 | 14.58 | 0 | 0.13 | 11.28 | 17.62 | 7.97 | 1.01E-12 | 7.81 | 3.66E-09 | 645 |
| Cluster-40555.159793 | 0.14 | 0 | 16.55 | 14.32 | 0 | 0 | 22.13 | 16.12 | 7.97 | 1.29E-12 | Inf | 2.07E-17 | 604 |
| Cluster-40555.191572 | 0.76 | 0 | 94.08 | 73.61 | 0.22 | 0.7 | 123.88 | 177.34 | 7.98 | 1.86E-67 | 8.39 | 8.94E-21 | 894 |
| Cluster-40555.234011 | 0.06 | 0 | 5.83 | 5.93 | 0.16 | 0 | 18.23 | 15.58 | 7.98 | 9.75E-13 | 7.87 | 2.36E-31 | 1162 |
| Cluster-40555.188576 | 21.62 | 9.3 | 3312.38 | 3664 | 1196.91 | 1101.56 | 4845.53 | 3675.66 | 7.98 | 4.67E-164 | 1.95 | 2.27E-11 | 2538 |
| Cluster-40555.214962 | 0.14 | 0 | 16.91 | 14.22 | 0.79 | 0.28 | 8.54 | 11.99 | 7.98 | 1.46E-33 | 4.36 | 3.32E-09 | 1286 |
| Cluster-40555.187863 | 0.41 | 0.11 | 59.05 | 57.99 | 0.66 | 0.42 | 39.63 | 46.51 | 7.98 | 6.80E-67 | 6.39 | 2.69E-41 | 970 |
| Cluster-40555.191958 | 0.08 | 0.04 | 13.42 | 10.51 | 0.07 | 0.23 | 10.37 | 10.83 | 7.99 | 5.98E-48 | 6.21 | 4.96E-35 | 2406 |
| Cluster-40555.195205 | 1.91 | 0.25 | 220.63 | 256.91 | 153.7 | 141.66 | 338.89 | 339.77 | 7.99 | 3.53E-78 | 1.26 | 1.21E-04 | 746 |
| Cluster-40555.282590 | 0 | 0.18 | 22.99 | 19.32 | 0.42 | 0.22 | 9.81 | 4.91 | 8.01 | 1.47E-11 | 4.58 | 4.00E-03 | 491 |
| Cluster-40555.161426 | 0.05 | 0 | 6.71 | 5.48 | 0 | 0 | 6.77 | 6.43 | 8.01 | 6.73E-13 | Inf | 3.87E-16 | 1152 |
| Cluster-40555.168766 | 0.03 | 0 | 3.99 | 4.25 | 1.41 | 1.33 | 5.32 | 5.8 | 8.02 | 3.51E-13 | 2.08 | 5.33E-04 | 1583 |
| Cluster-40555.188511 | 0.13 | 0 | 16.13 | 14.98 | 1.35 | 1.11 | 13.51 | 12.68 | 8.03 | 5.12E-51 | 3.48 | 2.63E-17 | 2023 |
| Cluster-40555.195859 | 0.18 | 0 | 19.74 | 21.2 | 0.54 | 0.51 | 27.27 | 15.61 | 8.03 | 3.10E-13 | 5.33 | 2.82E-06 | 529 |
| Cluster-40555.187503 | 0.23 | 0 | 23.23 | 29.16 | 0 | 0.12 | 45.64 | 48.67 | 8.03 | 5.74E-35 | 9.66 | 7.45E-60 | 1100 |
| Cluster-40555.211689 | 0.18 | 0 | 21.68 | 19.46 | 0 | 0 | 7.56 | 7.38 | 8.05 | 7.24E-44 | Inf | 2.26E-21 | 1345 |
| Cluster-40555.181566 | 0.53 | 0 | 63.43 | 57.86 | 0.13 | 0.41 | 86.72 | 91.06 | 8.05 | 5.85E-44 | 8.40 | 4.25E-51 | 629 |
| Cluster-40555.228579 | 0 | 0.15 | 20.33 | 17.04 | 0.09 | 0.27 | 14.72 | 11.53 | 8.06 | 8.55E-23 | 6.22 | 1.88E-15 | 805 |
| Cluster-40555.181741 | 0.05 | 0.04 | 8.78 | 12.24 | 0.05 | 0.1 | 14.31 | 11.07 | 8.07 | 6.47E-18 | 7.52 | 1.43E-27 | 1280 |
| Cluster-40555.205596 | 0 | 0.12 | 15.72 | 14.54 | 0 | 0 | 16.45 | 18 | 8.08 | 6.94E-41 | Inf | 1.49E-46 | 1614 |
| Cluster-40555.194907 | 0.89 | 0.42 | 160.88 | 153.42 | 0.75 | 0.39 | 73.67 | 64.19 | 8.08 | 2.58E-83 | 7.00 | 7.07E-41 | 645 |
| Cluster-40555.186638 | 5.52 | 0.66 | 657.83 | 762.61 | 46.6 | 57.72 | 595.43 | 515.5 | 8.09 | 3.36E-82 | 3.45 | 2.13E-24 | 379 |
| Cluster-40555.210540 | 0.07 | 0 | 7.67 | 9.17 | 0.14 | 0.19 | 6.18 | 6.62 | 8.09 | 1.61E-36 | 5.34 | 1.35E-21 | 2306 |
| Cluster-40555.185923 | 0.24 | 0 | 29.65 | 27.83 | 2.99 | 2.35 | 16.45 | 24.24 | 8.11 | 2.07E-45 | 3.02 | 6.05E-05 | 1067 |
| Cluster-40555.182435 | 0.08 | 0.03 | 13.45 | 15.02 | 1.31 | 0.75 | 7.89 | 7.35 | 8.11 | 1.30E-35 | 2.96 | 9.55E-08 | 1440 |
| Cluster-40555.165099 | 0 | 0.08 | 11.42 | 8.87 | 0.43 | 0.1 | 5.37 | 6.83 | 8.11 | 2.14E-12 | 4.56 | 1.31E-05 | 778 |
| Cluster-40555.185633 | 0.09 | 0 | 14.51 | 10.94 | 4.85 | 8.39 | 30.53 | 24.07 | 8.13 | 4.05E-26 | 2.09 | 4.38E-08 | 1186 |
| Cluster-40555.183513 | 1.11 | 0 | 140.8 | 128.36 | 0.38 | 0.68 | 141.62 | 158.26 | 8.13 | 1.64E-106 | 8.22 | 2.04E-82 | 1004 |
| Cluster-40555.139177 | 0 | 0.16 | 18.86 | 17.28 | 3.69 | 2.83 | 12.58 | 11.76 | 8.14 | 4.14E-24 | 1.97 | 1.35E-03 | 854 |
| Cluster-40555.185758 | 0.96 | 0 | 122.71 | 110.37 | 0 | 0.16 | 215.77 | 221.64 | 8.15 | 1.70E-61 | 11.39 | 5.68E-78 | 568 |
| Cluster-40555.188295 | 0.72 | 0.38 | 131.77 | 142.51 | 15.35 | 15.15 | 147.08 | 152.16 | 8.15 | 1.12E-131 | 3.36 | 4.46E-28 | 1562 |
| Cluster-40555.191647 | 0.76 | 0 | 94.44 | 94.43 | 0.28 | 0.68 | 131.3 | 120.86 | 8.16 | 3.44E-74 | 8.07 | 5.10E-68 | 770 |

| | | | | | | | | | | | | | |
|---|---|---|---|---|---|---|---|---|---|---|---|---|---|
| Cluster-40555.198964 | 0 | 0.03 | 5.11 | 5.52 | 0 | 0 | 5.44 | 5.16 | 8.17 | 9.15E-35 | Inf | 6.71E-37 | 3345 |
| Cluster-40555.187597 | 0.05 | 0 | 6.73 | 6.01 | 0.1 | 0 | 7.96 | 2.34 | 8.18 | 1.52E-14 | 6.81 | 4.41E-02 | 1217 |
| Cluster-40555.182366 | 0.37 | 0 | 43.75 | 48.83 | 0.72 | 0 | 38.77 | 33.01 | 8.19 | 1.51E-38 | 6.75 | 4.74E-27 | 658 |
| Cluster-40555.190970 | 1.42 | 0.75 | 288.81 | 279.65 | 10.59 | 9.26 | 280.84 | 282.99 | 8.19 | 2.00E-120 | 4.89 | 3.99E-48 | 740 |
| Cluster-40555.183356 | 0.04 | 0 | 4.13 | 4.87 | 0 | 0.11 | 11.04 | 13.65 | 8.20 | 5.72E-15 | 7.87 | 3.10E-32 | 1634 |
| Cluster-40555.244231 | 0 | 0.03 | 3.17 | 4.18 | 0.03 | 0.29 | 10.8 | 12.01 | 8.21 | 1.49E-13 | 6.17 | 1.53E-29 | 1769 |
| Cluster-40555.230613 | 0 | 0.05 | 5.49 | 7.49 | 0.66 | 1.2 | 5.5 | 9.01 | 8.21 | 1.40E-13 | 3.02 | 3.11E-03 | 1118 |
| Cluster-40555.176086 | 0.1 | 0 | 10.73 | 10.05 | 0 | 0 | 10.24 | 12.99 | 8.22 | 4.93E-15 | Inf | 7.68E-19 | 866 |
| Cluster-40555.163983 | 0.15 | 0 | 21.55 | 17.75 | 0.15 | 0.79 | 21.92 | 19.17 | 8.22 | 5.73E-15 | 5.48 | 6.44E-13 | 581 |
| Cluster-40555.135109 | 0.06 | 0 | 7.52 | 7.55 | 0.22 | 0.41 | 15.41 | 10.98 | 8.23 | 3.75E-15 | 5.41 | 2.28E-19 | 1097 |
| Cluster-40555.187711 | 1.38 | 0.28 | 232.46 | 204.37 | 53.35 | 35.18 | 143.19 | 141.62 | 8.23 | 3.91E-85 | 1.76 | 1.27E-06 | 544 |
| Cluster-40555.190654 | 0.15 | 0 | 19.15 | 21.42 | 0.41 | 0.79 | 38.93 | 42.46 | 8.24 | 2.39E-71 | 6.15 | 1.05E-59 | 2444 |
| Cluster-40555.182996 | 0.36 | 0.17 | 69.2 | 77.8 | 27.51 | 17.23 | 62.45 | 73.24 | 8.24 | 1.21E-100 | 1.68 | 4.60E-07 | 1423 |
| Cluster-40555.193765 | 0.08 | 0 | 12.04 | 12.03 | 0.08 | 0 | 5.09 | 4.76 | 8.24 | 3.07E-49 | 6.92 | 2.03E-22 | 2381 |
| Cluster-40555.210908 | 0.11 | 0 | 14.72 | 16.69 | 0 | 0 | 10.42 | 9.85 | 8.26 | 3.84E-40 | Inf | 4.18E-30 | 1487 |
| Cluster-40555.175550 | 0 | 0.07 | 7.93 | 12.16 | 0.16 | 0.35 | 4.08 | 4.03 | 8.26 | 3.83E-11 | 4.04 | 7.54E-04 | 836 |
| Cluster-40555.191051 | 0 | 0.15 | 22.05 | 19.85 | 0 | 0.16 | 10.1 | 9.58 | 8.28 | 5.69E-54 | 6.84 | 1.17E-27 | 1670 |
| Cluster-40555.150666 | 0.09 | 0 | 15.67 | 11.92 | 0 | 0 | 11.5 | 5.99 | 8.29 | 1.27E-15 | Inf | 1.47E-06 | 739 |
| Cluster-40555.229638 | 0.08 | 0 | 10.38 | 11.09 | 0.79 | 1.12 | 12.78 | 10.36 | 8.29 | 1.63E-29 | 3.64 | 2.64E-14 | 1489 |
| Cluster-40555.147541 | 0 | 0.03 | 3.31 | 3.27 | 2.96 | 3.1 | 6.91 | 8.22 | 8.30 | 2.42E-14 | 1.39 | 5.06E-03 | 2077 |
| Cluster-40555.188478 | 13.33 | 2.34 | 2157.92 | 2219.91 | 185.69 | 235.81 | 1649.38 | 1194.34 | 8.31 | 1.94E-192 | 2.81 | 8.92E-12 | 1166 |
| Cluster-40555.195924 | 0.46 | 0 | 56.06 | 66.92 | 0.68 | 0.95 | 62.63 | 54.1 | 8.31 | 6.69E-30 | 6.21 | 3.06E-23 | 477 |
| Cluster-40555.188514 | 0.51 | 0 | 70.47 | 74.52 | 0 | 0 | 43.14 | 40.64 | 8.31 | 7.10E-126 | Inf | 6.57E-89 | 2288 |
| Cluster-40555.170828 | 0.06 | 0.06 | 15.97 | 15.98 | 0.26 | 0.63 | 11.39 | 10.32 | 8.32 | 2.64E-49 | 4.62 | 3.85E-22 | 1861 |
| Cluster-40555.218541 | 0.11 | 0.08 | 24.12 | 20.21 | 3.59 | 1.01 | 22.02 | 18.15 | 8.33 | 1.46E-28 | 3.22 | 2.92E-10 | 843 |
| Cluster-40555.204927 | 0.34 | 0 | 48.95 | 44.72 | 0.76 | 0.36 | 29.38 | 32.71 | 8.35 | 1.88E-16 | 5.97 | 1.57E-09 | 407 |
| Cluster-40555.172440 | 0 | 0.22 | 32.77 | 37.65 | 2.53 | 5.27 | 57.75 | 47.11 | 8.36 | 4.82E-15 | 3.78 | 6.46E-11 | 437 |
| Cluster-40555.202967 | 0.26 | 0 | 34.01 | 42.26 | 0.1 | 0.37 | 35.59 | 35.36 | 8.37 | 1.57E-44 | 7.28 | 9.35E-59 | 1718 |
| Cluster-40555.182269 | 0.37 | 0 | 42.4 | 59.69 | 0.76 | 0 | 103.88 | 57.99 | 8.38 | 4.15E-16 | 7.88 | 5.03E-08 | 397 |
| Cluster-40555.187847 | 2.78 | 0 | 422.9 | 382.16 | 0 | 1.96 | 279.23 | 310.04 | 8.38 | 1.15E-160 | 8.25 | 1.41E-100 | 1169 |
| Cluster-40555.185809 | 0.47 | 0.38 | 126.89 | 131.86 | 2.64 | 1.46 | 156.01 | 148.98 | 8.39 | 3.15E-67 | 6.29 | 5.81E-49 | 571 |
| Cluster-40555.138937 | 0 | 0.08 | 9.56 | 9.28 | 0 | 0 | 11.6 | 11.12 | 8.40 | 2.71E-15 | Inf | 2.83E-20 | 935 |

| | | | | | | | | | | | | | |
|---|---|---|---|---|---|---|---|---|---|---|---|---|---|
| Cluster-40555.189580 | 0.08 | 0.03 | 16.53 | 16.18 | 0.47 | 0.26 | 12.47 | 15.34 | 8.40 | 1.90E-60 | 5.34 | 1.29E-26 | 2346 |
| Cluster-40555.194958 | 0.65 | 0 | 97.92 | 102.46 | 0.44 | 0 | 146.98 | 151.94 | 8.42 | 9.94E-110 | 9.44 | 1.05E-96 | 1266 |
| Cluster-40555.161696 | 0 | 0.04 | 5.64 | 7.75 | 0.43 | 0.65 | 16.1 | 13.27 | 8.45 | 4.83E-16 | 4.81 | 1.59E-20 | 1241 |
| Cluster-40555.169388 | 0.05 | 0 | 4.49 | 8.98 | 1.1 | 0.39 | 9.65 | 5.09 | 8.45 | 6.76E-06 | 3.38 | 3.77E-03 | 1345 |
| Cluster-40555.222255 | 0.05 | 0 | 7.72 | 6.42 | 0.13 | 0 | 7.06 | 4.32 | 8.45 | 1.43E-17 | 6.51 | 5.29E-10 | 1307 |
| Cluster-40555.186134 | 0.42 | 0 | 62.42 | 62.04 | 5.99 | 9.32 | 79.75 | 46.64 | 8.46 | 8.32E-18 | 3.08 | 1.96E-03 | 379 |
| Cluster-40555.187169 | 0 | 0.13 | 21.78 | 19.35 | 5.15 | 6.24 | 14.34 | 10.52 | 8.47 | 2.67E-79 | 1.18 | 2.87E-03 | 2866 |
| Cluster-40555.170438 | 0.06 | 0 | 9.86 | 6.96 | 0.22 | 0.16 | 5.55 | 3.75 | 8.48 | 1.01E-17 | 4.70 | 1.26E-07 | 1158 |
| Cluster-40555.188642 | 0.05 | 0 | 7.61 | 5.47 | 0.44 | 0.64 | 7.31 | 6.06 | 8.48 | 2.21E-30 | 3.68 | 2.72E-15 | 2568 |
| Cluster-40555.215555 | 0.16 | 0 | 24.74 | 24.41 | 0.47 | 0.16 | 22.95 | 22.03 | 8.48 | 4.88E-18 | 6.23 | 1.25E-14 | 567 |
| Cluster-40555.187259 | 0.97 | 0.52 | 257.63 | 234.3 | 15.98 | 3.99 | 144.85 | 127.71 | 8.48 | 3.90E-102 | 3.86 | 3.40E-26 | 613 |
| Cluster-40555.160169 | 0 | 0.02 | 4.66 | 3.01 | 0.67 | 0.29 | 1.71 | 2.64 | 8.50 | 5.19E-15 | 2.25 | 1.67E-02 | 2063 |
| Cluster-40555.186866 | 0.06 | 0.05 | 19.77 | 19.71 | 0 | 0.55 | 22.38 | 22.47 | 8.53 | 1.90E-32 | 6.41 | 8.71E-30 | 1006 |
| Cluster-40555.201207 | 0.03 | 0 | 5 | 3.25 | 0.15 | 0 | 6.29 | 5.51 | 8.53 | 5.17E-16 | 6.43 | 2.26E-22 | 2156 |
| Cluster-40555.190261 | 1.52 | 0.07 | 232.23 | 278.35 | 26.63 | 22.9 | 274.94 | 284.14 | 8.53 | 7.32E-67 | 3.56 | 2.19E-30 | 867 |
| Cluster-40555.203342 | 0 | 0.01 | 1.06 | 2.82 | 0 | 0 | 0.41 | 1 | 8.53 | 1.38E-03 | Inf | 6.01E-03 | 3830 |
| Cluster-40555.184996 | 0.08 | 0 | 13.3 | 10.53 | 0.65 | 0.33 | 13.55 | 17.7 | 8.54 | 1.67E-46 | 5.08 | 5.18E-17 | 2235 |
| Cluster-40555.146939 | 0.06 | 0 | 7.52 | 12.28 | 0.06 | 0.13 | 5.33 | 12.76 | 8.54 | 7.19E-10 | 6.68 | 4.44E-03 | 1049 |
| Cluster-40555.189995 | 4.27 | 0.66 | 767.36 | 864.72 | 1.32 | 3.17 | 538.72 | 753.36 | 8.55 | 1.42E-128 | 8.22 | 7.47E-25 | 668 |
| Cluster-40555.169889 | 0.06 | 0 | 8.68 | 12.46 | 1.89 | 1.65 | 20.31 | 18.05 | 8.56 | 2.40E-15 | 3.50 | 1.89E-13 | 1008 |
| Cluster-40555.187660 | 4.86 | 0.59 | 752.89 | 907.37 | 294.24 | 266.45 | 1348.29 | 1342.71 | 8.56 | 1.52E-57 | 2.29 | 1.20E-12 | 321 |
| Cluster-40555.183593 | 0.31 | 0 | 47.3 | 46.85 | 10.93 | 18.06 | 47.83 | 58.61 | 8.57 | 7.63E-35 | 1.93 | 5.91E-06 | 600 |
| Cluster-40555.178565 | 0 | 0.06 | 12.81 | 9.91 | 0.29 | 0 | 17.8 | 15.51 | 8.59 | 2.39E-17 | 6.94 | 1.20E-22 | 905 |
| Cluster-40555.194694 | 0.18 | 0 | 31.45 | 29.11 | 0.09 | 0.65 | 20.35 | 27.38 | 8.60 | 2.06E-35 | 6.05 | 6.69E-17 | 800 |
| Cluster-40555.194457 | 0.25 | 0.07 | 58.92 | 56.09 | 1.27 | 7.95 | 37.22 | 40.34 | 8.60 | 2.11E-82 | 3.11 | 2.75E-18 | 1228 |
| Cluster-40555.165563 | 0.06 | 0 | 9.26 | 9.5 | 0.67 | 0.87 | 7.64 | 8.03 | 8.61 | 1.27E-19 | 3.46 | 5.24E-08 | 1135 |
| Cluster-40555.189948 | 15.13 | 1.83 | 2801.5 | 3086.71 | 1220.6 | 941.07 | 2908.75 | 3156.57 | 8.63 | 1.18E-187 | 1.56 | 2.46E-07 | 798 |
| Cluster-40555.176005 | 0.28 | 0.14 | 65.73 | 77.46 | 1.01 | 0.45 | 67.41 | 72.82 | 8.66 | 1.70E-64 | 6.67 | 2.34E-52 | 947 |
| Cluster-40555.184056 | 0.27 | 0 | 47.83 | 42.51 | 27.86 | 17.58 | 163.89 | 203.52 | 8.67 | 2.36E-61 | 3.10 | 1.26E-13 | 1019 |
| Cluster-40555.196533 | 0.12 | 0 | 19.4 | 22.81 | 0 | 1.75 | 29.64 | 23.56 | 8.68 | 1.48E-37 | 4.93 | 2.63E-27 | 1078 |
| Cluster-40555.181596 | 0.31 | 0 | 57.02 | 54.11 | 3.08 | 2.1 | 30.6 | 31.77 | 8.69 | 2.26E-37 | 3.66 | 7.26E-11 | 573 |
| Cluster-40555.181103 | 1.22 | 0 | 199.5 | 217.46 | 2.16 | 0.95 | 181.09 | 200.69 | 8.71 | 4.07E-63 | 7.07 | 1.41E-44 | 429 |

| | | | | | | | | | | | | | |
|---|---|---|---|---|---|---|---|---|---|---|---|---|---|
| Cluster-40555.180427 | 0.04 | 0 | 4.41 | 11.39 | 0.62 | 0 | 5.4 | 4.31 | 8.75 | 6.70E-04 | 4.08 | 4.60E-08 | 1394 |
| Cluster-40555.171994 | 0.07 | 0 | 11.47 | 13.83 | 0 | 0 | 5.47 | 2.42 | 8.75 | 9.33E-22 | Inf | 3.47E-04 | 976 |
| Cluster-40555.155578 | 0.05 | 0 | 9.24 | 9.61 | 0.05 | 0.05 | 1.9 | 2.08 | 8.75 | 1.21E-21 | 5.37 | 3.86E-04 | 1223 |
| Cluster-40555.182581 | 0.07 | 0 | 11.93 | 15.09 | 0.7 | 0 | 7.86 | 5.65 | 8.77 | 4.95E-22 | 4.39 | 1.68E-07 | 938 |
| Cluster-40555.196354 | 0 | 0.05 | 9.71 | 8.23 | 0.66 | 0.45 | 10.62 | 6.46 | 8.77 | 1.23E-35 | 4.01 | 6.57E-07 | 2106 |
| Cluster-40555.175319 | 0 | 0.04 | 10.73 | 9.35 | 2.18 | 3.73 | 12.56 | 10.84 | 8.77 | 1.12E-35 | 2.04 | 6.86E-07 | 1908 |
| Cluster-40555.187331 | 0.53 | 0 | 91.17 | 101.2 | 1.08 | 1.67 | 114.02 | 93.01 | 8.79 | 3.53E-22 | 6.26 | 1.10E-18 | 355 |
| Cluster-40555.183190 | 0.07 | 0 | 13.73 | 11.36 | 0 | 0 | 7.28 | 11.44 | 8.79 | 5.17E-22 | Inf | 1.65E-10 | 1006 |
| Cluster-40555.167565 | 0 | 0.25 | 45.69 | 31.53 | 0 | 0 | 21.65 | 24.54 | 8.79 | 9.46E-20 | Inf | 2.05E-13 | 479 |
| Cluster-40555.208057 | 0 | 0.04 | 6.04 | 7.38 | 0 | 0 | 2.66 | 3.83 | 8.81 | 2.22E-20 | Inf | 1.56E-11 | 1522 |
| Cluster-40555.204201 | 0.1 | 0 | 20.07 | 18.88 | 0 | 0.21 | 12.86 | 15.05 | 8.81 | 2.31E-22 | 7.10 | 5.04E-16 | 746 |
| Cluster-40555.192535 | 0 | 0.07 | 14.54 | 15.65 | 2.3 | 2.91 | 7.12 | 8.78 | 8.82 | 2.10E-20 | 1.68 | 4.36E-02 | 824 |
| Cluster-40555.201662 | 0 | 0.07 | 12.26 | 13.48 | 0.62 | 0.33 | 3.89 | 5.12 | 8.82 | 4.88E-37 | 3.30 | 1.15E-06 | 1583 |
| Cluster-40555.188493 | 0.14 | 0 | 29.16 | 24.94 | 0 | 0.07 | 25.66 | 19.15 | 8.82 | 2.50E-40 | 9.33 | 2.47E-34 | 967 |
| Cluster-40555.181905 | 0.2 | 0 | 42.21 | 40.8 | 0 | 0 | 34.37 | 28.85 | 8.82 | 7.68E-55 | Inf | 2.16E-44 | 950 |
| Cluster-40555.189401 | 4.96 | 0 | 864.37 | 842.5 | 50.05 | 67.28 | 712.29 | 758.98 | 8.82 | 1.29E-66 | 3.64 | 5.47E-20 | 295 |
| Cluster-40555.192852 | 0.21 | 0 | 37.83 | 42.54 | 9.17 | 9.6 | 75.27 | 69.73 | 8.83 | 2.69E-116 | 3.01 | 1.05E-23 | 3364 |
| Cluster-40555.138945 | 0 | 0.05 | 10.24 | 12.56 | 0.06 | 0 | 5.69 | 6.06 | 8.84 | 7.19E-21 | 7.68 | 1.97E-11 | 1020 |
| Cluster-40555.187258 | 3.45 | 0.21 | 690.9 | 782.93 | 213.27 | 192.01 | 770.07 | 710.44 | 8.87 | 4.51E-125 | 1.93 | 8.38E-11 | 724 |
| Cluster-40555.190309 | 0.06 | 0 | 11.3 | 12.2 | 0.07 | 0.32 | 7.85 | 10.25 | 8.88 | 6.07E-42 | 5.52 | 8.81E-18 | 1940 |
| Cluster-40555.188539 | 0.84 | 0 | 177.77 | 170.1 | 0.1 | 0 | 3.33 | 5.17 | 8.90 | 1.70E-101 | 6.49 | 1.02E-04 | 722 |
| Cluster-40555.144059 | 0 | 0.04 | 9.22 | 7.53 | 0 | 0 | 1.32 | 1.12 | 8.91 | 2.06E-21 | Inf | 1.33E-03 | 1348 |
| Cluster-40555.161916 | 0.21 | 0 | 39.36 | 48.83 | 0 | 0 | 39.81 | 32.24 | 8.94 | 4.26E-39 | Inf | 4.66E-37 | 727 |
| Cluster-40555.212856 | 0 | 0.05 | 9.27 | 12.79 | 1.56 | 2.27 | 14.63 | 17.65 | 8.94 | 8.62E-19 | 3.12 | 4.41E-11 | 1099 |
| Cluster-40555.189687 | 0.05 | 0.06 | 24.48 | 21.45 | 0.74 | 0.86 | 15.46 | 11.29 | 8.97 | 3.12E-94 | 4.13 | 3.30E-21 | 3294 |
| Cluster-40555.188517 | 2.11 | 0 | 476.77 | 409.58 | 36.31 | 33.92 | 459.28 | 514.63 | 8.98 | 1.02E-97 | 3.85 | 2.83E-30 | 428 |
| Cluster-40555.204238 | 0.1 | 0 | 20.07 | 23.67 | 8.6 | 10.06 | 33.5 | 25.19 | 8.98 | 3.04E-25 | 1.71 | 2.37E-04 | 746 |
| Cluster-40555.248373 | 0.03 | 0 | 8.56 | 6.97 | 0.07 | 0.11 | 1.35 | 2.07 | 9.01 | 2.52E-25 | 4.37 | 2.66E-04 | 1662 |
| Cluster-40555.198317 | 0.25 | 0 | 57.8 | 58.79 | 8.07 | 6.72 | 25.26 | 30.93 | 9.04 | 1.59E-61 | 2.00 | 7.46E-06 | 832 |
| Cluster-40555.197271 | 0.61 | 0 | 139.14 | 132.56 | 1.22 | 0.95 | 31.77 | 45.32 | 9.04 | 6.28E-46 | 5.21 | 2.32E-11 | 424 |
| Cluster-40555.200265 | 0.07 | 0 | 17.14 | 17.89 | 0 | 0 | 4.98 | 7.89 | 9.06 | 2.00E-26 | Inf | 1.03E-08 | 903 |
| Cluster-40555.186002 | 0 | 0.31 | 77.22 | 77.11 | 0.14 | 0 | 13.12 | 10.7 | 9.06 | 2.02E-57 | 7.73 | 7.33E-12 | 654 |

| | | | | | | | | | | | | | |
|---|---|---|---|---|---|---|---|---|---|---|---|---|---|
| Cluster-40555.186858 | 0.1 | 0.11 | 49.47 | 47.13 | 4.6 | 4.48 | 60.36 | 67.68 | 9.06 | 4.51E-72 | 3.89 | 1.53E-28 | 1147 |
| Cluster-40555.195690 | 0.92 | 0 | 220.22 | 214.94 | 48.28 | 49.71 | 132.07 | 166.66 | 9.10 | 8.95E-103 | 1.67 | 5.41E-06 | 631 |
| Cluster-40555.186463 | 0.18 | 0 | 42.88 | 45.99 | 5.04 | 0.19 | 113.41 | 140.6 | 9.12 | 4.24E-48 | 5.72 | 3.78E-30 | 787 |
| Cluster-40555.217346 | 0.12 | 0 | 32.6 | 28.3 | 4 | 2.29 | 26.64 | 18.58 | 9.15 | 9.42E-28 | 2.91 | 2.02E-07 | 654 |
| Cluster-40555.214559 | 0.13 | 0 | 31.77 | 35.05 | 0.37 | 0.78 | 25.29 | 26.03 | 9.23 | 3.07E-51 | 5.52 | 1.84E-28 | 1018 |
| Cluster-40555.182737 | 0.16 | 0.1 | 74.59 | 75.56 | 0.62 | 0.77 | 62.45 | 56.82 | 9.25 | 3.98E-119 | 6.50 | 5.88E-63 | 1731 |
| Cluster-40555.193732 | 0.26 | 0.04 | 84 | 82.48 | 0.15 | 0.11 | 87.27 | 67.63 | 9.26 | 1.32E-100 | 9.28 | 3.27E-71 | 1175 |
| Cluster-40555.195523 | 0 | 0.05 | 18.03 | 23.92 | 0.39 | 0 | 17.09 | 15.59 | 9.27 | 8.25E-24 | 6.37 | 9.36E-19 | 816 |
| Cluster-40555.189722 | 0.73 | 0.16 | 247.81 | 257.78 | 19.8 | 12.08 | 38.39 | 30.64 | 9.32 | 8.65E-145 | 1.19 | 6.68E-03 | 990 |
| Cluster-40555.187874 | 0.33 | 0.28 | 167.17 | 174.99 | 7.61 | 3.28 | 33.78 | 45.33 | 9.32 | 8.99E-136 | 2.95 | 4.67E-07 | 1157 |
| Cluster-40555.192802 | 0 | 0.17 | 45.02 | 50.05 | 0.75 | 0.9 | 10.12 | 9.42 | 9.33 | 5.99E-79 | 3.62 | 3.10E-11 | 1283 |
| Cluster-40555.180283 | 0 | 0.31 | 85.98 | 92.13 | 3.55 | 2.04 | 41.33 | 45.83 | 9.35 | 1.75E-66 | 4.04 | 1.73E-18 | 676 |
| Cluster-40555.191822 | 0.03 | 0 | 10.58 | 9.96 | 0.18 | 0.2 | 4.43 | 1.91 | 9.35 | 2.64E-54 | 4.13 | 1.76E-02 | 2941 |
| Cluster-40555.188785 | 3.77 | 0 | 1088.04 | 1023.18 | 4.63 | 5.05 | 1586.71 | 1198.99 | 9.36 | 1.43E-184 | 8.22 | 2.59E-62 | 718 |
| Cluster-40555.191109 | 0 | 0.05 | 14.02 | 20.19 | 0.13 | 0.14 | 17.33 | 16.84 | 9.37 | 3.15E-17 | 7.08 | 7.38E-26 | 991 |
| Cluster-40555.220251 | 0.04 | 0 | 11.31 | 12.26 | 0 | 0 | 4.74 | 4.1 | 9.38 | 2.76E-32 | Inf | 1.40E-14 | 1450 |
| Cluster-40555.178562 | 0.03 | 0 | 7.31 | 6.63 | 0.26 | 0.16 | 11.2 | 10.79 | 9.39 | 2.62E-32 | 5.87 | 1.36E-32 | 2290 |
| Cluster-40555.207739 | 0.07 | 0 | 19.32 | 21.16 | 0.06 | 0 | 6.78 | 10.47 | 9.47 | 5.38E-34 | 8.20 | 7.43E-10 | 995 |
| Cluster-40555.184055 | 1.74 | 0.12 | 572.47 | 589.61 | 139.48 | 102.61 | 721.83 | 789.58 | 9.49 | 5.31E-149 | 2.71 | 1.35E-19 | 597 |
| Cluster-40555.187979 | 0.46 | 0 | 138.29 | 142.05 | 45.55 | 67.82 | 164.2 | 171.36 | 9.50 | 3.68E-137 | 1.62 | 1.26E-07 | 1349 |
| Cluster-40555.171925 | 0.09 | 0 | 31.91 | 26.44 | 0.27 | 0 | 16.21 | 17.98 | 9.52 | 1.04E-34 | 7.11 | 3.94E-20 | 791 |
| Cluster-40555.198318 | 0.16 | 0 | 41.01 | 64.01 | 1.31 | 1.88 | 9.87 | 19.18 | 9.60 | 4.78E-14 | 3.24 | 3.00E-02 | 571 |
| Cluster-40555.204330 | 0.05 | 0 | 12.14 | 17.66 | 0 | 0 | 4.21 | 11.15 | 9.67 | 7.27E-18 | Inf | 1.20E-03 | 1410 |
| Cluster-40555.191551 | 2.16 | 0 | 707.01 | 645.04 | 78.76 | 68.96 | 666.78 | 533.51 | 9.71 | 4.07E-98 | 3.07 | 3.81E-18 | 363 |
| Cluster-40555.134294 | 0.1 | 0 | 30.07 | 31.59 | 0.08 | 0 | 54.24 | 38.28 | 9.90 | 1.28E-43 | 10.43 | 1.23E-32 | 911 |
| Cluster-40555.190381 | 0.59 | 0.09 | 334.74 | 332.76 | 0.27 | 0 | 251.39 | 237.08 | 10.05 | 1.27E-166 | 11.11 | 2.95E-110 | 1047 |
| Cluster-40555.197282 | 0 | 0.09 | 50.01 | 54.88 | 0 | 0.07 | 23.05 | 20.21 | 10.27 | 3.38E-49 | 8.60 | 5.24E-23 | 704 |
| Cluster-40555.195888 | 0.1 | 0 | 62.28 | 58.09 | 0 | 0.33 | 70.14 | 79.68 | 10.30 | 1.11E-121 | 8.83 | 4.93E-91 | 2020 |
| Cluster-40555.184335 | 0.12 | 0 | 69.08 | 61.6 | 4.31 | 5.94 | 93.69 | 88.11 | 10.34 | 8.65E-145 | 4.21 | 2.69E-41 | 2770 |
| Cluster-40555.194563 | 0.32 | 0 | 185.32 | 193.65 | 0.24 | 0 | 29.37 | 23.31 | 10.40 | 2.25E-126 | 7.91 | 4.55E-32 | 861 |
| Cluster-40555.185003 | 0.02 | 0 | 15.05 | 10.58 | 1.94 | 1.74 | 11.84 | 19.76 | 10.72 | 2.84E-35 | 3.18 | 2.60E-03 | 3037 |
| Cluster-40555.187304 | 0.13 | 0 | 98.29 | 100.43 | 0.02 | 0.08 | 20.89 | 19.02 | 10.82 | 1.58E-155 | 8.67 | 7.91E-57 | 2178 |

| | | | | | | | | | | | | | |
|---|---|---|---|---|---|---|---|---|---|---|---|---|---|
| Cluster-40555.190279 | 0.06 | 0 | 59.26 | 57.1 | 0 | 0.12 | 31.23 | 29.16 | 11.15 | 1.22E-81 | 8.99 | 1.62E-45 | 1085 |
| Cluster-100005.0 | 0 | 0 | 3.49 | 3.44 | 0.16 | 0 | 3.96 | 2.52 | Inf | 1.36E-02 | 5.44 | 4.33E-02 | 561 |
| Cluster-40555.100090 | 0 | 0 | 1.42 | 1.01 | 0 | 0 | 0.61 | 0.99 | Inf | 5.76E-03 | Inf | 3.86E-02 | 1262 |
| Cluster-40555.100514 | 0 | 0 | 0.69 | 0.94 | 0.04 | 0 | 2.02 | 2.24 | Inf | 3.51E-02 | 6.71 | 1.30E-05 | 1328 |
| Cluster-40555.100648 | 0 | 0 | 6.44 | 4.93 | 0 | 0 | 13.82 | 20.55 | Inf | 1.14E-06 | Inf | 3.28E-13 | 720 |
| Cluster-40555.101003 | 0 | 0 | 3.55 | 3.72 | 0.07 | 0 | 3.88 | 3.47 | Inf | 1.51E-06 | 6.87 | 2.28E-06 | 956 |
| Cluster-40555.101117 | 0 | 0 | 0.81 | 0.77 | 0.41 | 0 | 1.5 | 1.05 | Inf | 3.38E-06 | 2.74 | 1.43E-03 | 3317 |
| Cluster-40555.101915 | 0 | 0 | 1.02 | 1.06 | 0.36 | 0.82 | 4.86 | 3.58 | Inf | 4.98E-02 | 2.88 | 1.52E-03 | 1045 |
| Cluster-40555.102028 | 0 | 0 | 0.74 | 0.81 | 0 | 0 | 1.21 | 0.87 | Inf | 9.18E-03 | Inf | 4.06E-04 | 1685 |
| Cluster-40555.102184 | 0 | 0 | 0.81 | 2.35 | 0 | 0 | 1.5 | 2.15 | Inf | 3.34E-02 | Inf | 5.05E-03 | 870 |
| Cluster-40555.103343 | 0 | 0 | 0.27 | 0.42 | 0 | 0 | 0.83 | 0.4 | Inf | 2.55E-03 | Inf | 4.08E-05 | 4026 |
| Cluster-40555.104040 | 0 | 0 | 0.71 | 0.62 | 0.1 | 0 | 0.77 | 1.03 | Inf | 5.96E-04 | 4.31 | 1.04E-03 | 2618 |
| Cluster-40555.104233 | 0 | 0 | 1 | 1.49 | 0.4 | 0.08 | 2.5 | 1.87 | Inf | 1.37E-02 | 3.37 | 1.84E-02 | 1086 |
| Cluster-40555.104411 | 0 | 0 | 0.7 | 0.75 | 0.26 | 0 | 1.44 | 1.56 | Inf | 2.15E-02 | 3.57 | 6.59E-03 | 1612 |
| Cluster-40555.105074 | 0 | 0 | 6.06 | 8.78 | 0 | 0 | 3.11 | 3.69 | Inf | 1.94E-08 | Inf | 5.46E-04 | 689 |
| Cluster-40555.105085 | 0 | 0 | 1.14 | 1.46 | 0 | 0 | 1.37 | 1.34 | Inf | 2.63E-02 | Inf | 1.45E-02 | 966 |
| Cluster-40555.105086 | 0 | 0 | 5.73 | 5.23 | 0 | 0 | 5.04 | 9.57 | Inf | 5.29E-11 | Inf | 5.53E-06 | 1036 |
| Cluster-40555.105901 | 0 | 0 | 1.01 | 2.22 | 0 | 0.11 | 1.67 | 0.8 | Inf | 4.14E-04 | 4.62 | 2.85E-03 | 1723 |
| Cluster-40555.106271 | 0 | 0 | 5.25 | 7.87 | 0 | 0.15 | 3.14 | 3.62 | Inf | 7.17E-12 | 5.55 | 3.85E-05 | 942 |
| Cluster-40555.106633 | 0 | 0 | 7.08 | 5.35 | 0 | 0 | 8.04 | 5.65 | Inf | 4.60E-12 | Inf | 2.35E-14 | 1019 |
| Cluster-40555.106779 | 0 | 0 | 0.27 | 0.51 | 0.02 | 0.02 | 1.36 | 0.87 | Inf | 3.31E-02 | 5.75 | 1.24E-05 | 2466 |
| Cluster-40555.107126 | 0 | 0 | 1.98 | 2.06 | 0 | 0 | 2.85 | 2.81 | Inf | 2.45E-02 | Inf | 1.31E-03 | 727 |
| Cluster-40555.107353 | 0 | 0 | 1.56 | 2.29 | 0 | 0 | 0.51 | 0.5 | Inf | 1.00E-10 | Inf | 7.32E-03 | 2358 |
| Cluster-40555.107413 | 0 | 0 | 1.64 | 1.12 | 0 | 0 | 1 | 0.7 | Inf | 4.53E-05 | Inf | 1.90E-03 | 1722 |
| Cluster-40555.107414 | 0 | 0 | 0.81 | 0.69 | 0 | 0 | 0.5 | 0.79 | Inf | 1.50E-02 | Inf | 2.32E-02 | 1626 |
| Cluster-40555.107655 | 0 | 0 | 2.55 | 2.29 | 0 | 0 | 2.43 | 1.93 | Inf | 1.07E-05 | Inf | 1.27E-05 | 1179 |
| Cluster-40555.109315 | 0 | 0 | 2.94 | 3.1 | 0 | 0 | 2.16 | 2.75 | Inf | 4.28E-04 | Inf | 1.29E-03 | 795 |
| Cluster-40555.109373 | 0 | 0 | 2.18 | 2.88 | 0.09 | 0.04 | 2.26 | 3.71 | Inf | 1.53E-08 | 5.44 | 2.01E-05 | 1539 |
| Cluster-40555.109499 | 0 | 0 | 0.59 | 0.65 | 0 | 0.21 | 1.47 | 1.99 | Inf | 4.39E-02 | 4.01 | 6.37E-04 | 1612 |
| Cluster-40555.109845 | 0 | 0 | 2.68 | 2.92 | 0 | 0 | 2.21 | 1.41 | Inf | 8.42E-05 | Inf | 2.71E-03 | 928 |
| Cluster-40555.109955 | 0 | 0 | 17.64 | 16.92 | 1.2 | 0 | 13.36 | 21.71 | Inf | 8.34E-04 | 4.96 | 9.96E-03 | 345 |
| Cluster-40555.110185 | 0 | 0 | 2.33 | 2.19 | 0 | 0 | 1.51 | 1.24 | Inf | 1.53E-12 | Inf | 2.37E-08 | 2398 |

| | | | | | | | | | | | | | |
|---|---|---|---|---|---|---|---|---|---|---|---|---|---|
| Cluster-40555.110394 | 0 | 0 | 0.37 | 0.18 | 0 | 0 | 1.64 | 0.67 | Inf | 4.66E-02 | Inf | 6.14E-04 | 3414 |
| Cluster-40555.110686 | 0 | 0 | 0.77 | 0.4 | 0 | 0 | 0.91 | 0.41 | Inf | 2.29E-02 | Inf | 3.90E-03 | 1971 |
| Cluster-40555.110797 | 0 | 0 | 0.46 | 0.83 | 0 | 0.37 | 1.26 | 1.12 | Inf | 3.01E-03 | 2.77 | 1.40E-02 | 2204 |
| Cluster-40555.110869 | 0 | 0 | 2.19 | 3.68 | 0 | 0 | 3.67 | 2.43 | Inf | 1.09E-05 | Inf | 2.79E-06 | 994 |
| Cluster-40555.111463 | 0 | 0 | 7.05 | 8.5 | 0.86 | 0.49 | 12.33 | 7.84 | Inf | 2.60E-12 | 3.97 | 2.10E-07 | 864 |
| Cluster-40555.112279 | 0 | 0 | 1.3 | 1.23 | 0 | 0 | 1.11 | 0.44 | Inf | 3.62E-03 | Inf | 4.13E-02 | 1269 |
| Cluster-40555.112556 | 0 | 0 | 1.86 | 1.18 | 0 | 0 | 1.13 | 1.63 | Inf | 7.49E-03 | Inf | 7.13E-03 | 1024 |
| Cluster-40555.112732 | 0 | 0 | 0.82 | 0.81 | 0 | 0 | 1.13 | 0.59 | Inf | 1.83E-02 | Inf | 7.64E-03 | 1471 |
| Cluster-40555.113329 | 0 | 0 | 0.73 | 0.62 | 0 | 0 | 0.77 | 0.79 | Inf | 4.10E-02 | Inf | 8.65E-03 | 1559 |
| Cluster-40555.113439 | 0 | 0 | 2.06 | 2.03 | 0 | 0 | 0.95 | 1.1 | Inf | 3.49E-04 | Inf | 3.32E-02 | 1055 |
| Cluster-40555.113580 | 0 | 0 | 1.79 | 1.84 | 0.19 | 0 | 2.5 | 1.94 | Inf | 5.23E-05 | 4.78 | 1.42E-04 | 1330 |
| Cluster-40555.114370 | 0 | 0 | 1.55 | 0.53 | 0 | 0 | 1.38 | 1.25 | Inf | 1.87E-02 | Inf | 1.40E-04 | 1518 |
| Cluster-40555.114447 | 0 | 0 | 4.08 | 5.25 | 2.14 | 1.95 | 10.43 | 10.96 | Inf | 1.74E-10 | 2.45 | 6.59E-06 | 1115 |
| Cluster-40555.114551 | 0 | 0 | 0.44 | 0.51 | 0 | 0 | 1.65 | 1.72 | Inf | 2.21E-03 | Inf | 3.32E-13 | 3049 |
| Cluster-40555.114816 | 0 | 0 | 1.18 | 1.52 | 0.34 | 0.41 | 2.64 | 1.77 | Inf | 2.00E-03 | 2.66 | 2.93E-02 | 1267 |
| Cluster-40555.114908 | 0 | 0 | 0.61 | 1.48 | 0 | 0 | 0.73 | 1.19 | Inf | 1.58E-02 | Inf | 2.32E-02 | 1190 |
| Cluster-40555.115114 | 0 | 0 | 0.87 | 0.41 | 0 | 0 | 0.8 | 0.4 | Inf | 3.48E-02 | Inf | 2.49E-02 | 1694 |
| Cluster-40555.115354 | 0 | 0 | 1.42 | 2.06 | 0.24 | 0.13 | 1.9 | 2.05 | Inf | 1.59E-03 | 3.51 | 2.94E-02 | 1044 |
| Cluster-40555.115584 | 0 | 0 | 3.23 | 3.74 | 0.63 | 0.89 | 7.36 | 11.98 | Inf | 4.62E-06 | 3.73 | 2.82E-04 | 930 |
| Cluster-40555.116376 | 0 | 0 | 1.12 | 1.26 | 0 | 0 | 1.36 | 0.88 | Inf | 3.89E-03 | Inf | 3.21E-03 | 1307 |
| Cluster-40555.117009 | 0 | 0 | 0.48 | 0.54 | 0.64 | 0.93 | 3.81 | 3.35 | Inf | 2.89E-02 | 2.25 | 3.42E-04 | 2067 |
| Cluster-40555.117357 | 0 | 0 | 0.68 | 2.2 | 0 | 0 | 1.5 | 2.18 | Inf | 3.17E-02 | Inf | 4.66E-05 | 1231 |
| Cluster-40555.119476 | 0 | 0 | 0.74 | 0.55 | 0.12 | 0.05 | 1.47 | 1.02 | Inf | 1.65E-02 | 3.86 | 3.98E-03 | 1831 |
| Cluster-40555.119733 | 0 | 0 | 1.25 | 0.78 | 0 | 0 | 1.24 | 0.48 | Inf | 2.45E-02 | Inf | 3.51E-02 | 1216 |
| Cluster-40555.119977 | 0 | 0 | 7.3 | 6.97 | 0 | 0 | 7.89 | 10.11 | Inf | 1.50E-05 | Inf | 1.66E-07 | 565 |
| Cluster-40555.120555 | 0 | 0 | 0.94 | 0.5 | 0 | 0 | 2.34 | 2 | Inf | 3.59E-02 | Inf | 1.08E-07 | 1506 |
| Cluster-40555.120724 | 0 | 0 | 1.03 | 0.96 | 0.05 | 0 | 1.41 | 1.12 | Inf | 3.60E-02 | 5.70 | 1.49E-02 | 1147 |
| Cluster-40555.120921 | 0 | 0 | 17.14 | 11.7 | 0 | 0 | 22.91 | 18.63 | Inf | 1.41E-13 | Inf | 4.52E-20 | 631 |
| Cluster-40555.121939 | 0 | 0 | 0.94 | 1.05 | 0 | 0 | 1.51 | 1.34 | Inf | 2.52E-03 | Inf | 2.11E-05 | 1598 |
| Cluster-40555.122125 | 0 | 0 | 7.66 | 7.32 | 0.12 | 0.12 | 7.72 | 6.74 | Inf | 3.99E-08 | 6.01 | 6.54E-07 | 679 |
| Cluster-40555.122238 | 0 | 0 | 18.88 | 20.34 | 0 | 0.27 | 10.35 | 14.64 | Inf | 1.50E-24 | 6.40 | 2.46E-12 | 759 |
| Cluster-40555.122460 | 0 | 0 | 0.37 | 0.39 | 0 | 0 | 0.42 | 0.24 | Inf | 3.15E-04 | Inf | 4.95E-04 | 4590 |

| | | | | | | | | | | | | | |
|---|---|---|---|---|---|---|---|---|---|---|---|---|---|
| Cluster-40555.122581 | 0 | 0 | 5.78 | 3.15 | 0 | 0 | 2.1 | 3.08 | Inf | 2.93E-08 | Inf | 4.51E-07 | 1227 |
| Cluster-40555.122994 | 0 | 0 | 1.23 | 1.15 | 1.06 | 0.76 | 3.97 | 4.61 | Inf | 4.82E-02 | 2.36 | 2.27E-02 | 938 |
| Cluster-40555.124439 | 0 | 0 | 1.31 | 1.37 | 0.42 | 0 | 2.16 | 2.14 | Inf | 1.83E-02 | 3.37 | 3.85E-02 | 1000 |
| Cluster-40555.124734 | 0 | 0 | 19.35 | 12.21 | 0 | 0 | 14.83 | 7.52 | Inf | 8.58E-08 | Inf | 1.15E-04 | 455 |
| Cluster-40555.124801 | 0 | 0 | 1.78 | 2.46 | 1.66 | 2.05 | 6.53 | 7.63 | Inf | 1.32E-02 | 1.99 | 3.64E-02 | 746 |
| Cluster-40555.124926 | 0 | 0 | 0.57 | 0.45 | 0.08 | 0.11 | 0.9 | 0.78 | Inf | 3.14E-05 | 3.21 | 3.72E-04 | 4295 |
| Cluster-40555.125098 | 0 | 0 | 0.82 | 1.54 | 0 | 0 | 1.57 | 1.91 | Inf | 1.15E-03 | Inf | 7.16E-06 | 1449 |
| Cluster-40555.125157 | 0 | 0 | 1.51 | 2.38 | 0.2 | 0.06 | 2.47 | 1.75 | Inf | 4.47E-04 | 4.24 | 5.60E-03 | 1065 |
| Cluster-40555.125158 | 0 | 0 | 2.99 | 2.99 | 0.12 | 0 | 2.22 | 1.53 | Inf | 2.13E-07 | 4.78 | 1.70E-03 | 1215 |
| Cluster-40555.125357 | 0 | 0 | 0.68 | 0.24 | 0 | 0 | 0.67 | 0.46 | Inf | 1.22E-02 | Inf | 2.89E-04 | 2975 |
| Cluster-40555.125382 | 0 | 0 | 2.15 | 1.94 | 0.03 | 0.17 | 2.15 | 1.31 | Inf | 1.92E-07 | 4.09 | 3.65E-04 | 1670 |
| Cluster-40555.125403 | 0 | 0 | 8.77 | 6.35 | 0 | 0 | 11.14 | 16.72 | Inf | 1.45E-19 | Inf | 1.28E-14 | 1294 |
| Cluster-40555.125522 | 0 | 0 | 0.75 | 0.63 | 0 | 0 | 0.49 | 1.12 | Inf | 2.97E-03 | Inf | 1.11E-02 | 2171 |
| Cluster-40555.126344 | 0 | 0 | 8.19 | 4.79 | 0.32 | 0 | 2.13 | 4.68 | Inf | 2.06E-10 | 4.55 | 3.25E-02 | 999 |
| Cluster-40555.126553 | 0 | 0 | 1.38 | 0.55 | 0 | 0 | 1.6 | 1.8 | Inf | 5.38E-03 | Inf | 3.05E-06 | 1559 |
| Cluster-40555.126574 | 0 | 0 | 3.15 | 3.85 | 0.08 | 0 | 3.15 | 4.45 | Inf | 1.21E-10 | 6.67 | 1.47E-10 | 1416 |
| Cluster-40555.126662 | 0 | 0 | 0.46 | 0.67 | 0 | 0 | 0.78 | 0.62 | Inf | 2.72E-02 | Inf | 4.45E-03 | 1838 |
| Cluster-40555.126665 | 0 | 0 | 2.95 | 4.03 | 0 | 0.1 | 4.69 | 3.23 | Inf | 9.71E-05 | 6.39 | 4.97E-05 | 783 |
| Cluster-40555.126771 | 0 | 0 | 3.43 | 3.68 | 0 | 0 | 2.08 | 1.66 | Inf | 6.21E-05 | Inf | 1.05E-02 | 800 |
| Cluster-40555.126897 | 0 | 0 | 2.62 | 1.51 | 0 | 0 | 3.36 | 2.82 | Inf | 2.50E-03 | Inf | 1.07E-05 | 920 |
| Cluster-40555.127197 | 0 | 0 | 2.05 | 1.17 | 0 | 0 | 0.98 | 1.15 | Inf | 2.54E-04 | Inf | 3.64E-03 | 1346 |
| Cluster-40555.127217 | 0 | 0 | 2.27 | 0.9 | 0.12 | 0 | 1.19 | 1.62 | Inf | 3.03E-03 | 4.69 | 3.24E-03 | 1436 |
| Cluster-40555.128521 | 0 | 0 | 0.81 | 0.92 | 0 | 0 | 1.11 | 0.54 | Inf | 4.39E-02 | Inf | 4.10E-02 | 1227 |
| Cluster-40555.128559 | 0 | 0 | 1.64 | 1.55 | 0.45 | 0.42 | 2.24 | 2.41 | Inf | 4.26E-05 | 2.46 | 1.56E-02 | 1507 |
| Cluster-40555.128799 | 0 | 0 | 0.69 | 0.37 | 0.01 | 0 | 2.02 | 1.95 | Inf | 4.53E-04 | 8.18 | 5.40E-16 | 3395 |
| Cluster-40555.129191 | 0 | 0 | 1.02 | 1.31 | 0 | 0 | 0.82 | 1.95 | Inf | 3.53E-03 | Inf | 1.32E-02 | 1325 |
| Cluster-40555.129357 | 0 | 0 | 5.21 | 4.26 | 0 | 0 | 3.92 | 4.74 | Inf | 2.92E-12 | Inf | 2.83E-12 | 1268 |
| Cluster-40555.129664 | 0 | 0 | 1.67 | 1.66 | 0 | 0 | 0.44 | 1.1 | Inf | 2.24E-04 | Inf | 4.91E-02 | 1291 |
| Cluster-40555.129832 | 0 | 0 | 4.4 | 5.67 | 0 | 0 | 6.05 | 5.33 | Inf | 8.45E-11 | Inf | 6.47E-13 | 1074 |
| Cluster-40555.130108 | 0 | 0 | 1.02 | 1.24 | 0 | 0 | 1.5 | 1.2 | Inf | 2.62E-03 | Inf | 2.01E-04 | 1423 |
| Cluster-40555.130279 | 0 | 0 | 1.39 | 1.97 | 0 | 0.15 | 1.45 | 0.96 | Inf | 2.77E-04 | 4.00 | 4.36E-02 | 1252 |
| Cluster-40555.130318 | 0.01 | 0 | 1.45 | 1.61 | 0 | 0.05 | 1.9 | 2.09 | Inf | 1.23E-02 | 5.78 | 4.15E-03 | 939 |

| | | | | | | | | | | | | | |
|---|---|---|---|---|---|---|---|---|---|---|---|---|---|
| Cluster-40555.130384 | 0 | 0 | 1.38 | 1.48 | 0.06 | 0.04 | 2.5 | 2.27 | Inf | 3.76E-06 | 5.43 | 2.22E-08 | 1923 |
| Cluster-40555.130460 | 0 | 0 | 0.73 | 0.49 | 0 | 0 | 0.66 | 0.49 | Inf | 5.56E-03 | Inf | 3.77E-03 | 2238 |
| Cluster-40555.130678 | 0 | 0 | 0.9 | 1.1 | 0.12 | 0.25 | 1.26 | 1.56 | Inf | 1.90E-04 | 3.00 | 7.07E-03 | 2000 |
| Cluster-40555.130769 | 0 | 0 | 1.68 | 1.72 | 0 | 0 | 0.46 | 1.21 | Inf | 3.46E-11 | Inf | 1.45E-02 | 2776 |
| Cluster-40555.131246 | 0 | 0 | 2.28 | 1.58 | 0 | 0 | 1.72 | 1.28 | Inf | 2.82E-04 | Inf | 1.13E-03 | 1147 |
| Cluster-40555.131269 | 0 | 0 | 1.28 | 0.9 | 0 | 0 | 0.5 | 0.91 | Inf | 5.04E-03 | Inf | 3.84E-02 | 1392 |
| Cluster-40555.131462 | 0 | 0 | 4.16 | 3.15 | 0.28 | 0.44 | 4.34 | 4.34 | Inf | 4.41E-03 | 3.65 | 2.78E-02 | 607 |
| Cluster-40555.131676 | 0 | 0 | 1.44 | 0.78 | 0 | 0 | 0.59 | 0.31 | Inf | 4.53E-05 | Inf | 3.46E-02 | 2102 |
| Cluster-40555.131909 | 0 | 0 | 4.61 | 2.33 | 0 | 0 | 1.96 | 2.19 | Inf | 1.74E-06 | Inf | 1.21E-05 | 1227 |
| Cluster-40555.132333 | 0 | 0 | 0.88 | 0.7 | 0 | 0.04 | 0.81 | 1.73 | Inf | 5.97E-05 | 6.05 | 4.10E-03 | 2752 |
| Cluster-40555.133066 | 0 | 0 | 7.31 | 10.48 | 0 | 0 | 5.13 | 5.21 | Inf | 3.87E-12 | Inf | 1.73E-07 | 776 |
| Cluster-40555.133232 | 0 | 0 | 0.96 | 1.04 | 1.24 | 1.21 | 4.59 | 3.51 | Inf | 3.39E-08 | 1.79 | 1.94E-04 | 3395 |
| Cluster-40555.133585 | 0 | 0 | 0.76 | 0.52 | 0 | 0 | 0.42 | 0.45 | Inf | 3.16E-03 | Inf | 2.01E-02 | 2314 |
| Cluster-40555.133611 | 0 | 0 | 3.85 | 3.25 | 0.05 | 0 | 2.92 | 2.55 | Inf | 1.57E-09 | 7.04 | 3.36E-07 | 1301 |
| Cluster-40555.134080 | 0 | 0 | 4.2 | 6.3 | 0 | 0 | 3.12 | 2.9 | Inf | 8.53E-10 | Inf | 6.27E-06 | 962 |
| Cluster-40555.134084 | 0 | 0 | 4.43 | 3.75 | 0.49 | 0.25 | 2.84 | 1.9 | Inf | 1.59E-19 | 2.72 | 4.53E-04 | 2149 |
| Cluster-40555.134216 | 0 | 0 | 1.68 | 1.29 | 0 | 0 | 1.36 | 1.69 | Inf | 3.14E-05 | Inf | 4.25E-06 | 1661 |
| Cluster-40555.134269 | 0 | 0 | 1.8 | 1.81 | 0 | 0.08 | 3.02 | 2.74 | Inf | 1.34E-03 | 6.56 | 9.66E-06 | 1059 |
| Cluster-40555.134389 | 0 | 0 | 2.88 | 4.02 | 0 | 0 | 1.74 | 2.37 | Inf | 8.26E-06 | Inf | 1.28E-03 | 904 |
| Cluster-40555.134437 | 0 | 0 | 0.47 | 0.73 | 0.07 | 0.05 | 0.66 | 1.18 | Inf | 6.72E-05 | 3.99 | 5.10E-03 | 3361 |
| Cluster-40555.134560 | 0 | 0 | 1.25 | 1.66 | 0 | 0 | 2.57 | 5.76 | Inf | 4.17E-03 | Inf | 8.44E-04 | 1086 |
| Cluster-40555.134573 | 0 | 0 | 1.69 | 1.75 | 0 | 0 | 1.14 | 1.27 | Inf | 1.61E-05 | Inf | 3.89E-04 | 1504 |
| Cluster-40555.134605 | 0 | 0 | 17.12 | 20.41 | 0 | 0 | 16.6 | 14.77 | Inf | 2.98E-03 | Inf | 1.73E-02 | 324 |
| Cluster-40555.134606 | 0 | 0 | 3.93 | 3.66 | 0 | 0 | 4.41 | 2.22 | Inf | 7.58E-05 | Inf | 1.49E-04 | 760 |
| Cluster-40555.134647 | 0 | 0 | 3 | 2.42 | 0.58 | 0.61 | 8.61 | 7.77 | Inf | 9.45E-03 | 3.84 | 5.35E-05 | 672 |
| Cluster-40555.134723 | 0 | 0 | 0.7 | 1.07 | 0 | 0 | 1.74 | 0.84 | Inf | 3.78E-03 | Inf | 3.62E-04 | 1645 |
| Cluster-40555.135035 | 0 | 0 | 5.54 | 6.17 | 0 | 0.24 | 1.75 | 2.57 | Inf | 2.19E-09 | 4.17 | 2.17E-02 | 873 |
| Cluster-40555.135098 | 0 | 0 | 3.62 | 1.68 | 0 | 0 | 2.67 | 2.87 | Inf | 9.99E-05 | Inf | 4.34E-06 | 1037 |
| Cluster-40555.135213 | 0 | 0 | 18.83 | 22.45 | 7.11 | 1.33 | 64.89 | 53.53 | Inf | 2.49E-04 | 3.89 | 3.60E-06 | 339 |
| Cluster-40555.135234 | 0 | 0 | 1.21 | 0.99 | 0 | 0.15 | 1.16 | 2.26 | Inf | 2.61E-02 | 4.34 | 9.96E-03 | 1116 |
| Cluster-40555.135295 | 0 | 0 | 53.85 | 46 | 0 | 0 | 8.95 | 16.52 | Inf | 1.02E-09 | Inf | 3.26E-02 | 332 |
| Cluster-40555.135773 | 0 | 0 | 5.66 | 5.62 | 0 | 0 | 5.06 | 4.41 | Inf | 7.94E-07 | Inf | 3.76E-06 | 727 |

| | | | | | | | | | | | | | |
|---|---|---|---|---|---|---|---|---|---|---|---|---|---|
| Cluster-40555.135858 | 0 | 0 | 5.73 | 3.94 | 0.53 | 0.56 | 7.2 | 10.27 | Inf | 2.08E-04 | 4.07 | 5.16E-05 | 622 |
| Cluster-40555.136163 | 0 | 0 | 2.13 | 2.04 | 0 | 0 | 1.36 | 1.15 | Inf | 1.41E-06 | Inf | 2.83E-04 | 1486 |
| Cluster-40555.136232 | 0 | 0 | 4.15 | 3.78 | 0.59 | 1.02 | 5.28 | 3.92 | Inf | 1.56E-08 | 2.60 | 1.74E-03 | 1093 |
| Cluster-40555.136313 | 0 | 0 | 1.19 | 1.05 | 0 | 0 | 3.65 | 4.16 | Inf | 9.78E-03 | Inf | 9.61E-11 | 1239 |
| Cluster-40555.136347 | 0 | 0 | 2.31 | 1.7 | 0.21 | 0 | 2.07 | 2.41 | Inf | 2.92E-04 | 4.44 | 2.13E-03 | 1104 |
| Cluster-40555.136540 | 0 | 0 | 2.51 | 1.93 | 0 | 0.19 | 1.95 | 2.66 | Inf | 8.01E-03 | 4.61 | 2.33E-02 | 776 |
| Cluster-40555.137194 | 0 | 0 | 1.69 | 1.14 | 0.19 | 0.14 | 1.27 | 1.76 | Inf | 9.17E-08 | 3.28 | 4.71E-04 | 2416 |
| Cluster-40555.137299 | 0 | 0 | 1.38 | 2.08 | 1.11 | 0.81 | 4.81 | 3.96 | Inf | 1.36E-04 | 2.24 | 4.24E-03 | 1270 |
| Cluster-40555.137599 | 0 | 0 | 3.99 | 2.73 | 0 | 0 | 2.45 | 2.01 | Inf | 4.72E-06 | Inf | 2.39E-04 | 971 |
| Cluster-40555.137758 | 0 | 0 | 1.04 | 0.83 | 0.12 | 0.12 | 2.42 | 1.65 | Inf | 9.45E-03 | 4.13 | 4.15E-04 | 1452 |
| Cluster-40555.137779 | 0 | 0 | 3.29 | 1.51 | 0 | 0 | 0.89 | 1.68 | Inf | 7.91E-06 | Inf | 6.10E-05 | 2522 |
| Cluster-40555.137798 | 0 | 0 | 0.52 | 0.52 | 0.13 | 0 | 0.83 | 0.71 | Inf | 1.19E-02 | 3.85 | 1.76E-02 | 2283 |
| Cluster-40555.138176 | 0 | 0 | 1.17 | 1.2 | 0.11 | 0 | 1.87 | 1.81 | Inf | 1.19E-02 | 5.25 | 1.76E-03 | 1154 |
| Cluster-40555.138290 | 0 | 0 | 3.9 | 5.68 | 0 | 0.09 | 1.58 | 2.16 | Inf | 1.29E-06 | 5.33 | 3.55E-02 | 789 |
| Cluster-40555.138450 | 0 | 0 | 1.52 | 2.11 | 0.18 | 0.09 | 3.1 | 3.3 | Inf | 2.03E-02 | 4.63 | 3.28E-03 | 788 |
| Cluster-40555.138736 | 0 | 0 | 0.37 | 0.48 | 0 | 0 | 0.38 | 0.75 | Inf | 1.12E-02 | Inf | 9.93E-04 | 2735 |
| Cluster-40555.139144 | 0 | 0 | 4.39 | 5.15 | 0.35 | 0 | 2.39 | 3.4 | Inf | 4.90E-05 | 4.16 | 4.87E-02 | 668 |
| Cluster-40555.139217 | 0 | 0 | 0.75 | 0.95 | 0 | 0.06 | 0.79 | 0.57 | Inf | 2.00E-03 | 4.49 | 3.67E-02 | 1859 |
| Cluster-40555.139472 | 0 | 0 | 3.53 | 3.09 | 0 | 0 | 2.53 | 3.07 | Inf | 9.01E-06 | Inf | 2.41E-05 | 943 |
| Cluster-40555.139677 | 0 | 0 | 5.05 | 2.64 | 0 | 0 | 4.66 | 3.19 | Inf | 2.29E-02 | Inf | 1.25E-02 | 517 |
| Cluster-40555.139851 | 0 | 0 | 2.5 | 2.95 | 0 | 0 | 1.4 | 1.22 | Inf | 2.15E-07 | Inf | 7.92E-04 | 1305 |
| Cluster-40555.140136 | 0 | 0 | 2.15 | 0.98 | 0 | 0 | 1.21 | 1.25 | Inf | 1.42E-03 | Inf | 3.10E-03 | 1208 |
| Cluster-40555.140138 | 0 | 0 | 1.34 | 1.42 | 0 | 0 | 1.92 | 2.36 | Inf | 2.89E-02 | Inf | 6.48E-04 | 927 |
| Cluster-40555.140247 | 0 | 0 | 15.24 | 16.05 | 0 | 0.06 | 2.63 | 3.07 | Inf | 1.13E-32 | 6.66 | 3.52E-06 | 1127 |
| Cluster-40555.140528 | 0 | 0 | 1.93 | 2.66 | 0 | 0 | 1.89 | 1.61 | Inf | 2.33E-04 | Inf | 1.58E-03 | 995 |
| Cluster-40555.140637 | 0 | 0 | 3.46 | 3.11 | 0.16 | 0.13 | 2.78 | 2.18 | Inf | 9.53E-10 | 4.15 | 9.59E-05 | 1406 |
| Cluster-40555.140658 | 0 | 0 | 7.35 | 7.44 | 0 | 0 | 4.76 | 2.49 | Inf | 3.43E-10 | Inf | 3.61E-05 | 792 |
| Cluster-40555.140684 | 0 | 0 | 2.87 | 6.23 | 0 | 0 | 3.53 | 6.03 | Inf | 8.72E-03 | Inf | 5.94E-03 | 497 |
| Cluster-40555.140685 | 0 | 0 | 5.22 | 4.91 | 0 | 0.11 | 5.39 | 6.19 | Inf | 1.11E-05 | 6.68 | 3.01E-06 | 698 |
| Cluster-40555.140690 | 0 | 0 | 2.1 | 2.27 | 0 | 0 | 2.92 | 2.78 | Inf | 1.90E-05 | Inf | 1.05E-07 | 1222 |
| Cluster-40555.141026 | 0 | 0 | 3.47 | 2.4 | 0.26 | 0.1 | 1.78 | 1.44 | Inf | 5.91E-08 | 3.24 | 3.13E-02 | 1323 |
| Cluster-40555.141193 | 0 | 0 | 7.36 | 9.82 | 0 | 0 | 3.67 | 3.81 | Inf | 7.76E-07 | Inf | 6.15E-03 | 563 |

| | | | | | | | | | | | | | |
|---|---|---|---|---|---|---|---|---|---|---|---|---|---|
| Cluster-40555.141244 | 0 | 0 | 1.02 | 1.42 | 0 | 0 | 1.31 | 1.38 | Inf | 1.32E-04 | Inf | 1.45E-05 | 1712 |
| Cluster-40555.141282 | 0 | 0 | 0.86 | 2.15 | 0 | 0 | 1.42 | 1.95 | Inf | 1.58E-02 | Inf | 6.02E-03 | 908 |
| Cluster-40555.141325 | 0 | 0 | 7.6 | 13.58 | 0.23 | 0.27 | 10.5 | 10.73 | Inf | 1.09E-07 | 5.37 | 7.60E-10 | 721 |
| Cluster-40555.141476 | 0 | 0 | 8.38 | 9.02 | 0 | 0 | 8.18 | 8.29 | Inf | 3.67E-28 | Inf | 4.85E-27 | 1573 |
| Cluster-40555.141648 | 0 | 0 | 1.38 | 1.05 | 0 | 0 | 1.61 | 1.24 | Inf | 3.97E-02 | Inf | 8.84E-03 | 977 |
| Cluster-40555.141855 | 0 | 0 | 1.55 | 3.58 | 0 | 0 | 2.49 | 1.7 | Inf | 3.33E-03 | Inf | 5.34E-03 | 784 |
| Cluster-40555.142116 | 0 | 0 | 0.74 | 0.55 | 0 | 0 | 0.35 | 0.41 | Inf | 4.85E-05 | Inf | 2.58E-03 | 3360 |
| Cluster-40555.142199 | 0 | 0 | 1.3 | 1.28 | 0.52 | 0.26 | 2.43 | 1.36 | Inf | 4.32E-07 | 2.35 | 1.79E-02 | 2379 |
| Cluster-40555.142216 | 0 | 0 | 4.5 | 3.99 | 1.94 | 0.58 | 3.61 | 4.49 | Inf | 3.38E-13 | 1.78 | 2.70E-02 | 1465 |
| Cluster-40555.142411 | 0 | 0 | 1.7 | 1.61 | 0.52 | 0.64 | 4.07 | 4.31 | Inf | 2.35E-03 | 2.87 | 1.49E-03 | 1074 |
| Cluster-40555.142578 | 0 | 0 | 2.03 | 2.92 | 0 | 0 | 2.34 | 2.02 | Inf | 1.78E-02 | Inf | 2.86E-02 | 656 |
| Cluster-40555.142655 | 0 | 0 | 1.12 | 1.16 | 0 | 0.07 | 2.34 | 3.21 | Inf | 4.98E-02 | 6.35 | 7.41E-05 | 979 |
| Cluster-40555.142769 | 0 | 0 | 3.4 | 2.51 | 0 | 0 | 2.13 | 0.9 | Inf | 7.06E-11 | Inf | 1.11E-03 | 1695 |
| Cluster-40555.143362 | 0 | 0 | 8.43 | 7.29 | 0.11 | 0.11 | 7.28 | 5.31 | Inf | 1.15E-10 | 6.15 | 1.09E-07 | 785 |
| Cluster-40555.143438 | 0 | 0 | 0.98 | 1.19 | 0 | 0 | 1.06 | 1.47 | Inf | 3.05E-12 | Inf | 6.57E-15 | 4566 |
| Cluster-40555.143668 | 0 | 0 | 1.02 | 1.2 | 0.11 | 0.17 | 1.58 | 1.48 | Inf | 7.06E-03 | 3.47 | 2.62E-02 | 1284 |
| Cluster-40555.143904 | 0 | 0 | 2.73 | 5.75 | 0.24 | 0 | 1.5 | 1.77 | Inf | 3.65E-05 | 3.88 | 1.53E-02 | 1226 |
| Cluster-40555.143917 | 0 | 0 | 0.51 | 1.01 | 0 | 0 | 1.4 | 1.39 | Inf | 3.57E-05 | Inf | 9.95E-15 | 4036 |
| Cluster-40555.143987 | 0 | 0 | 0.65 | 0.54 | 0.16 | 0 | 0.69 | 0.58 | Inf | 2.84E-05 | 3.12 | 1.12E-02 | 3742 |
| Cluster-40555.144173 | 0 | 0 | 3.82 | 4.63 | 0.49 | 2.2 | 8.13 | 8.63 | Inf | 2.62E-03 | 2.65 | 1.40E-02 | 572 |
| Cluster-40555.144302 | 0 | 0 | 2.36 | 1.44 | 0 | 0 | 3.46 | 2.23 | Inf | 1.90E-09 | Inf | 2.08E-14 | 2211 |
| Cluster-40555.144578 | 0 | 0 | 1.01 | 2.43 | 0 | 0 | 1.77 | 1.73 | Inf | 4.05E-03 | Inf | 9.33E-04 | 1040 |
| Cluster-40555.144838 | 0 | 0 | 16.05 | 19.41 | 0.58 | 0.3 | 26.37 | 20.94 | Inf | 2.19E-15 | 5.82 | 1.58E-15 | 593 |
| Cluster-40555.145145 | 0 | 0 | 1.4 | 2.7 | 0 | 0 | 2.46 | 1.88 | Inf | 4.42E-02 | Inf | 2.88E-02 | 658 |
| Cluster-40555.145150 | 0 | 0 | 1.2 | 0.95 | 0 | 0 | 1.14 | 0.97 | Inf | 8.51E-09 | Inf | 1.39E-09 | 3452 |
| Cluster-40555.145276 | 0 | 0 | 12.99 | 14.66 | 0 | 0.14 | 21.8 | 17.2 | Inf | 5.33E-24 | 8.09 | 3.32E-29 | 949 |
| Cluster-40555.145431 | 0 | 0 | 5.8 | 6.14 | 0 | 0.13 | 9.27 | 8.8 | Inf | 4.42E-23 | 7.16 | 1.86E-28 | 1798 |
| Cluster-40555.145677 | 0 | 0 | 1.54 | 1.33 | 0 | 0 | 1.63 | 1.76 | Inf | 1.53E-11 | Inf | 9.73E-15 | 3368 |
| Cluster-40555.145747 | 0 | 0 | 3.16 | 3.65 | 0.12 | 0 | 2.65 | 2.11 | Inf | 1.28E-10 | 5.44 | 5.14E-06 | 1446 |
| Cluster-40555.145808 | 0 | 0 | 9.38 | 10.43 | 0.05 | 0 | 1.52 | 2.1 | Inf | 7.41E-22 | 6.19 | 9.90E-04 | 1129 |
| Cluster-40555.146015 | 0 | 0 | 4.1 | 3.73 | 0 | 0.11 | 2.81 | 2.8 | Inf | 3.07E-13 | 5.66 | 3.33E-08 | 1570 |
| Cluster-40555.146150 | 0 | 0 | 5.41 | 9.17 | 0 | 0.19 | 4.93 | 8.95 | Inf | 3.00E-05 | 6.24 | 2.66E-04 | 534 |

| | | | | | | | | | | | | | |
|---|---|---|---|---|---|---|---|---|---|---|---|---|---|
| Cluster-40555.146185 | 0 | 0 | 3.11 | 3.29 | 0.21 | 0.69 | 3.75 | 4.21 | Inf | 7.56E-04 | 3.13 | 1.98E-02 | 735 |
| Cluster-40555.146207 | 0 | 0 | 5.07 | 6.19 | 0 | 0.11 | 10.63 | 8.26 | Inf | 8.27E-07 | 7.46 | 4.54E-11 | 723 |
| Cluster-40555.146208 | 0 | 0 | 9.25 | 10.05 | 0 | 0 | 15.12 | 10.84 | Inf | 2.18E-14 | Inf | 1.05E-19 | 834 |
| Cluster-40555.146387 | 0 | 0 | 4.56 | 6.52 | 0 | 0 | 2.1 | 4.75 | Inf | 7.62E-04 | Inf | 2.30E-02 | 529 |
| Cluster-40555.146417 | 0 | 0 | 7.16 | 13.58 | 1.57 | 1.24 | 10.3 | 6.82 | Inf | 1.23E-07 | 2.66 | 6.98E-06 | 1141 |
| Cluster-40555.146588 | 0 | 0 | 9.39 | 7.54 | 0.14 | 0 | 2.25 | 3.96 | Inf | 1.28E-09 | 5.96 | 4.03E-03 | 702 |
| Cluster-40555.147032 | 0 | 0 | 9.3 | 8.32 | 3.13 | 3.27 | 12.03 | 13.13 | Inf | 6.02E-13 | 2.05 | 8.16E-04 | 831 |
| Cluster-40555.147036 | 0 | 0 | 4.28 | 2.68 | 1.34 | 2.39 | 10.1 | 6.12 | Inf | 6.35E-03 | 2.17 | 4.28E-02 | 606 |
| Cluster-40555.147047 | 0 | 0 | 0.24 | 0.32 | 0 | 0 | 1.07 | 0.59 | Inf | 7.57E-03 | Inf | 7.90E-08 | 4292 |
| Cluster-40555.147223 | 0 | 0 | 12.98 | 13.17 | 0 | 0 | 2.07 | 2.06 | Inf | 4.01E-24 | Inf | 3.30E-04 | 997 |
| Cluster-40555.147416 | 0 | 0 | 0.46 | 0.81 | 0 | 0 | 1 | 0.54 | Inf | 2.64E-03 | Inf | 2.49E-04 | 2282 |
| Cluster-40555.147658 | 0 | 0 | 15.33 | 12.9 | 0 | 0 | 15.76 | 12.47 | Inf | 2.67E-06 | Inf | 1.39E-06 | 435 |
| Cluster-40555.147663 | 0 | 0 | 0.59 | 0.71 | 0 | 0.08 | 1.13 | 1.01 | Inf | 6.19E-03 | 4.72 | 8.01E-04 | 2043 |
| Cluster-40555.147897 | 0 | 0 | 0.68 | 0.73 | 0 | 0 | 0.43 | 1.14 | Inf | 5.42E-05 | Inf | 1.26E-02 | 3017 |
| Cluster-40555.147900 | 0 | 0 | 4.65 | 3.94 | 0 | 0 | 4.22 | 7.54 | Inf | 9.69E-30 | Inf | 5.90E-09 | 3121 |
| Cluster-40555.148077 | 0 | 0 | 0.22 | 0.29 | 0 | 0 | 0.25 | 0.83 | Inf | 2.71E-03 | Inf | 4.59E-02 | 5327 |
| Cluster-40555.148147 | 0 | 0 | 2.67 | 2.18 | 0.41 | 0 | 1.62 | 2.14 | Inf | 4.46E-12 | 3.32 | 2.32E-04 | 2199 |
| Cluster-40555.148237 | 0 | 0 | 4.85 | 5.28 | 0 | 0 | 1.48 | 1.5 | Inf | 1.92E-08 | Inf | 1.71E-02 | 897 |
| Cluster-40555.148254 | 0 | 0 | 3.37 | 3.98 | 0.15 | 0 | 3.63 | 4.09 | Inf | 1.04E-04 | 5.44 | 4.53E-04 | 756 |
| Cluster-40555.148268 | 0 | 0 | 4.91 | 4.03 | 0.14 | 0.08 | 1.53 | 2.95 | Inf | 1.52E-07 | 4.45 | 9.57E-03 | 918 |
| Cluster-40555.148574 | 0 | 0 | 0.95 | 1.23 | 0 | 0 | 1.7 | 2.58 | Inf | 5.27E-05 | Inf | 2.35E-09 | 2019 |
| Cluster-40555.148581 | 0 | 0 | 1.71 | 1.9 | 0 | 0.08 | 2.19 | 2.67 | Inf | 5.99E-03 | 6.01 | 9.93E-04 | 910 |
| Cluster-40555.148616 | 0 | 0 | 0.77 | 2.27 | 0 | 0 | 1.36 | 0.83 | Inf | 7.23E-03 | Inf | 5.60E-06 | 2178 |
| Cluster-40555.148709 | 0 | 0 | 1.06 | 2.36 | 0 | 0.1 | 1.67 | 3.75 | Inf | 2.65E-02 | 5.85 | 1.90E-02 | 782 |
| Cluster-40555.148738 | 0 | 0 | 6.14 | 5.03 | 0 | 0.15 | 3.76 | 5.38 | Inf | 1.52E-09 | 5.95 | 5.11E-07 | 924 |
| Cluster-40555.148767 | 0 | 0 | 3.29 | 3.24 | 0.51 | 0.18 | 1.74 | 2.16 | Inf | 2.18E-09 | 2.60 | 4.31E-02 | 1366 |
| Cluster-40555.148807 | 0 | 0 | 2.23 | 1.73 | 0 | 0 | 1.56 | 2.39 | Inf | 5.76E-05 | Inf | 1.96E-05 | 1245 |
| Cluster-40555.148827 | 0 | 0 | 1.36 | 1.89 | 0 | 0 | 1.28 | 1.84 | Inf | 1.35E-03 | Inf | 1.08E-03 | 1123 |
| Cluster-40555.149525 | 0 | 0 | 4.66 | 5.47 | 0.19 | 0 | 3.18 | 2.14 | Inf | 1.68E-06 | 4.91 | 1.22E-02 | 753 |
| Cluster-40555.149889 | 0 | 0 | 1.5 | 1.72 | 0 | 0 | 1.7 | 1.17 | Inf | 2.81E-07 | Inf | 5.09E-07 | 1980 |
| Cluster-40555.150032 | 0 | 0 | 0.72 | 0.61 | 0 | 0 | 1.42 | 0.8 | Inf | 2.19E-03 | Inf | 2.41E-06 | 2288 |
| Cluster-40555.150180 | 0 | 0 | 1.32 | 1.22 | 0 | 0 | 1.65 | 1.33 | Inf | 1.77E-02 | Inf | 3.16E-03 | 1055 |

| | | | | | | | | | | | | | |
|---|---|---|---|---|---|---|---|---|---|---|---|---|---|
| Cluster-40555.150186 | 0 | 0 | 3.78 | 6.08 | 0.15 | 0 | 3.5 | 3.48 | Inf | 4.36E-08 | 5.62 | 9.93E-05 | 873 |
| Cluster-40555.150292 | 0 | 0 | 5.52 | 6.38 | 0.57 | 0.72 | 8.04 | 7.61 | Inf | 1.15E-11 | 3.65 | 1.40E-07 | 1009 |
| Cluster-40555.150499 | 0 | 0 | 2.07 | 1.52 | 0.46 | 0.36 | 4.59 | 4.38 | Inf | 1.23E-02 | 3.52 | 1.21E-03 | 855 |
| Cluster-40555.150832 | 0 | 0 | 1.18 | 0.41 | 0 | 0 | 0.95 | 0.94 | Inf | 2.92E-02 | Inf | 1.31E-03 | 1644 |
| Cluster-40555.150911 | 0 | 0 | 4.39 | 5.8 | 0.52 | 1.12 | 9.72 | 12.42 | Inf | 7.16E-05 | 3.84 | 6.14E-06 | 631 |
| Cluster-40555.151026 | 0 | 0 | 2 | 2.04 | 0 | 0 | 1.24 | 2.85 | Inf | 1.34E-03 | Inf | 8.71E-03 | 957 |
| Cluster-40555.151158 | 0 | 0 | 44.3 | 25.69 | 1.34 | 0 | 37.05 | 30.48 | Inf | 1.37E-03 | 5.73 | 1.28E-02 | 291 |
| Cluster-40555.151266 | 0 | 0 | 1.97 | 2.3 | 0.16 | 0 | 2.03 | 2.22 | Inf | 3.18E-08 | 4.86 | 5.50E-06 | 1731 |
| Cluster-40555.151491 | 0 | 0 | 1.42 | 0.92 | 0 | 0 | 1.79 | 1.28 | Inf | 4.37E-02 | Inf | 4.48E-03 | 998 |
| Cluster-40555.151922 | 0 | 0 | 8.93 | 11.92 | 0.42 | 0.43 | 10.73 | 17.85 | Inf | 1.59E-17 | 5.06 | 1.06E-05 | 906 |
| Cluster-40555.151999 | 0 | 0 | 1.01 | 0.85 | 0.11 | 0.28 | 1.16 | 1.21 | Inf | 6.91E-07 | 2.67 | 2.63E-03 | 3151 |
| Cluster-40555.152196 | 0 | 0 | 4.38 | 3.7 | 0.33 | 0.17 | 8.95 | 4.21 | Inf | 6.16E-03 | 4.79 | 6.38E-03 | 556 |
| Cluster-40555.152323 | 0 | 0 | 2.02 | 1.81 | 0.48 | 0.28 | 1.52 | 1.33 | Inf | 7.98E-12 | 2.00 | 3.72E-02 | 2648 |
| Cluster-40555.152716 | 0 | 0 | 3.67 | 4.15 | 0 | 0 | 3.83 | 4.28 | Inf | 3.09E-14 | Inf | 1.19E-15 | 1656 |
| Cluster-40555.152848 | 0 | 0 | 1.69 | 1.93 | 0.64 | 0.12 | 2.64 | 3.05 | Inf | 6.74E-07 | 2.99 | 2.46E-04 | 1727 |
| Cluster-40555.153106 | 0 | 0 | 2.64 | 3.45 | 0 | 0 | 1.59 | 2.89 | Inf | 8.37E-09 | Inf | 2.36E-05 | 1361 |
| Cluster-40555.153161 | 0 | 0 | 1.72 | 3.64 | 0.04 | 0.04 | 0.81 | 1.47 | Inf | 1.08E-04 | 4.98 | 5.42E-03 | 1533 |
| Cluster-40555.153906 | 0 | 0 | 1.73 | 3 | 0 | 0.23 | 1.59 | 1.59 | Inf | 3.69E-08 | 3.73 | 2.66E-05 | 2489 |
| Cluster-40555.153971 | 0 | 0 | 3.59 | 8.09 | 0.18 | 0 | 13.76 | 16.33 | Inf | 1.45E-04 | 7.22 | 1.74E-21 | 905 |
| Cluster-40555.153977 | 0 | 0 | 6.02 | 4.89 | 0 | 0.12 | 1.56 | 2.52 | Inf | 3.03E-16 | 5.04 | 5.49E-05 | 1435 |
| Cluster-40555.154190 | 0 | 0 | 10.04 | 9.33 | 0 | 0 | 10.25 | 6.53 | Inf | 1.99E-03 | Inf | 5.38E-03 | 401 |
| Cluster-40555.154240 | 0 | 0 | 1.83 | 1.95 | 0.11 | 0 | 3.45 | 2.66 | Inf | 5.04E-09 | 6.03 | 2.62E-12 | 2097 |
| Cluster-40555.154242 | 0 | 0 | 5.53 | 6.68 | 0.2 | 0.31 | 7.29 | 5.75 | Inf | 1.20E-25 | 4.70 | 1.11E-16 | 1934 |
| Cluster-40555.154346 | 0 | 0 | 5.04 | 4.23 | 0 | 0 | 4.48 | 5.38 | Inf | 8.28E-03 | Inf | 3.64E-03 | 505 |
| Cluster-40555.154548 | 0 | 0 | 2.72 | 4.35 | 0.4 | 0.1 | 1.81 | 3.54 | Inf | 3.34E-06 | 3.54 | 3.62E-02 | 929 |
| Cluster-40555.154627 | 0 | 0 | 2.53 | 2.94 | 0 | 0 | 4.79 | 3.08 | Inf | 1.66E-02 | Inf | 6.90E-04 | 626 |
| Cluster-40555.154688 | 0 | 0 | 0.79 | 0.9 | 0 | 0.05 | 3.89 | 4.32 | Inf | 3.39E-02 | 7.48 | 3.54E-11 | 1316 |
| Cluster-40555.154833 | 0 | 0 | 6.44 | 5.79 | 0.34 | 0.86 | 7.52 | 5.87 | Inf | 1.48E-20 | 3.52 | 4.01E-10 | 1593 |
| Cluster-40555.154958 | 0 | 0 | 5.26 | 5.25 | 0 | 0 | 4.9 | 3.35 | Inf | 2.46E-14 | Inf | 3.27E-12 | 1316 |
| Cluster-40555.154959 | 0 | 0 | 2.8 | 2.8 | 0.35 | 0.56 | 2.05 | 3.28 | Inf | 1.02E-07 | 2.61 | 1.04E-02 | 1318 |
| Cluster-40555.154961 | 0 | 0 | 1.92 | 3.09 | 0 | 0 | 2.62 | 1.62 | Inf | 1.98E-10 | Inf | 1.77E-12 | 2534 |
| Cluster-40555.155123 | 0 | 0 | 0.54 | 0.47 | 0 | 0.19 | 1.66 | 1.51 | Inf | 1.14E-04 | 4.09 | 6.29E-09 | 3909 |

| | | | | | | | | | | | | | |
|---|---|---|---|---|---|---|---|---|---|---|---|---|---|
| Cluster-40555.155403 | 0 | 0 | 16.27 | 16.6 | 0.13 | 0 | 4.43 | 7.49 | Inf | 9.32E-16 | 6.65 | 2.37E-05 | 632 |
| Cluster-40555.155531 | 0 | 0 | 3.77 | 2.88 | 0.16 | 0.25 | 4.04 | 3.04 | Inf | 4.24E-05 | 4.19 | 1.08E-03 | 866 |
| Cluster-40555.155684 | 0 | 0 | 1.33 | 2.04 | 0 | 0 | 2.11 | 2.27 | Inf | 5.72E-06 | Inf | 2.24E-08 | 1609 |
| Cluster-40555.156321 | 0 | 0 | 7.89 | 9.99 | 0 | 0.08 | 7.75 | 9.88 | Inf | 1.74E-26 | 7.75 | 6.14E-24 | 1442 |
| Cluster-40555.156539 | 0 | 0 | 3.45 | 2.81 | 0 | 0 | 3.13 | 4.15 | Inf | 3.39E-03 | Inf | 4.55E-04 | 671 |
| Cluster-40555.156617 | 0 | 0 | 2.7 | 1.97 | 0 | 0 | 1.35 | 2.04 | Inf | 3.15E-06 | Inf | 5.51E-05 | 1307 |
| Cluster-40555.156701 | 0 | 0 | 2.04 | 1.96 | 0.15 | 0 | 1.99 | 1.1 | Inf | 5.79E-05 | 4.53 | 7.95E-03 | 1239 |
| Cluster-40555.156979 | 0 | 0 | 1.68 | 1.41 | 0.34 | 0.36 | 2.52 | 1.38 | Inf | 5.90E-09 | 2.52 | 1.17E-02 | 2502 |
| Cluster-40555.157141 | 0 | 0 | 1.62 | 1.97 | 0.06 | 0 | 1.66 | 1.96 | Inf | 8.11E-04 | 6.11 | 1.61E-03 | 1087 |
| Cluster-40555.157243 | 0 | 0 | 0.38 | 0.51 | 0 | 0 | 0.55 | 0.2 | Inf | 4.76E-04 | Inf | 2.56E-02 | 3737 |
| Cluster-40555.157256 | 0 | 0 | 1.16 | 0.56 | 0.11 | 0.08 | 1.18 | 1.35 | Inf | 3.48E-02 | 3.89 | 2.88E-02 | 1329 |
| Cluster-40555.157545 | 0 | 0 | 1.03 | 0.37 | 0 | 0 | 0.88 | 0.84 | Inf | 4.37E-02 | Inf | 6.19E-03 | 1519 |
| Cluster-40555.157554 | 0 | 0.02 | 4.58 | 5.53 | 0 | 0 | 1.6 | 4.16 | Inf | 3.76E-17 | Inf | 5.31E-03 | 1568 |
| Cluster-40555.157562 | 0 | 0 | 3.08 | 3.26 | 0 | 0.12 | 2.95 | 2.5 | Inf | 4.57E-05 | 6.11 | 4.64E-04 | 888 |
| Cluster-40555.157930 | 0 | 0 | 2.02 | 0.97 | 0.47 | 1.18 | 3.96 | 3.14 | Inf | 5.74E-04 | 2.14 | 1.09E-02 | 1345 |
| Cluster-40555.158107 | 0 | 0 | 10.86 | 9.67 | 0.48 | 0.11 | 11.78 | 15.44 | Inf | 2.88E-15 | 5.67 | 2.42E-15 | 840 |
| Cluster-40555.158390 | 0 | 0 | 0.67 | 0.54 | 0 | 0 | 1.04 | 0.79 | Inf | 8.73E-07 | Inf | 2.13E-11 | 4655 |
| Cluster-40555.158400 | 0 | 0 | 0.43 | 0.42 | 0.03 | 0.02 | 0.87 | 0.47 | Inf | 1.78E-05 | 4.64 | 1.21E-04 | 5281 |
| Cluster-40555.158401 | 0 | 0 | 0.39 | 0.38 | 0 | 0 | 0.25 | 0.73 | Inf | 2.56E-04 | Inf | 2.94E-02 | 4656 |
| Cluster-40555.158484 | 0 | 0 | 1.16 | 1.14 | 0.23 | 0.18 | 3.69 | 3.68 | Inf | 4.89E-03 | 4.21 | 3.10E-06 | 1297 |
| Cluster-40555.158486 | 0 | 0 | 1.95 | 2.68 | 0.12 | 0.13 | 4.3 | 2.29 | Inf | 9.71E-05 | 4.80 | 4.06E-04 | 1048 |
| Cluster-40555.158488 | 0 | 0 | 1.43 | 1.36 | 0 | 0.22 | 2.69 | 2.19 | Inf | 2.08E-02 | 4.57 | 2.17E-03 | 971 |
| Cluster-40555.158530 | 0 | 0 | 1.19 | 1.42 | 0.12 | 0.07 | 1.82 | 1.82 | Inf | 1.46E-02 | 4.38 | 1.31E-02 | 1036 |
| Cluster-40555.158582 | 0 | 0 | 1.6 | 1.42 | 0.42 | 0.45 | 3.17 | 3.17 | Inf | 1.35E-04 | 2.92 | 5.45E-04 | 1464 |
| Cluster-40555.158602 | 0 | 0 | 1.73 | 3.38 | 0 | 0 | 4.55 | 3.31 | Inf | 1.38E-04 | Inf | 1.55E-07 | 941 |
| Cluster-40555.158689 | 0 | 0 | 3.67 | 6.58 | 0.42 | 0 | 2.57 | 3.97 | Inf | 1.30E-05 | 3.96 | 3.26E-02 | 672 |
| Cluster-40555.158717 | 0 | 0 | 4.77 | 4.83 | 0.44 | 0.05 | 2.83 | 2.69 | Inf | 1.53E-17 | 3.59 | 3.25E-05 | 1685 |
| Cluster-40555.158831 | 0 | 0 | 1.27 | 1.23 | 0.2 | 0.19 | 1.52 | 1.11 | Inf | 2.96E-06 | 2.82 | 9.49E-03 | 2218 |
| Cluster-40555.158883 | 0 | 0 | 1.03 | 0.7 | 0 | 0.07 | 1.42 | 1.46 | Inf | 6.41E-06 | 5.33 | 2.82E-08 | 2952 |
| Cluster-40555.159027 | 0 | 0 | 2.99 | 3.32 | 0 | 0 | 2.51 | 3.19 | Inf | 5.06E-04 | Inf | 6.48E-04 | 766 |
| Cluster-40555.159031 | 0 | 0 | 2.41 | 2.55 | 0 | 0 | 2.29 | 0.62 | Inf | 2.40E-14 | Inf | 3.24E-02 | 2489 |
| Cluster-40555.159051 | 0 | 0 | 1.39 | 1.55 | 0.09 | 0.19 | 2.48 | 1.34 | Inf | 2.15E-02 | 4.08 | 3.90E-02 | 905 |

| | | | | | | | | | | | | | |
|---|---|---|---|---|---|---|---|---|---|---|---|---|---|
| Cluster-40555.159101 | 0 | 0 | 68.27 | 69.7 | 2.53 | 0 | 41 | 46.7 | Inf | 1.69E-11 | 5.20 | 5.92E-05 | 319 |
| Cluster-40555.159179 | 0 | 0 | 2.58 | 2.24 | 0 | 0 | 1.64 | 2.28 | Inf | 9.23E-04 | Inf | 2.54E-03 | 882 |
| Cluster-40555.159256 | 0 | 0.01 | 4.67 | 4.08 | 0 | 0.27 | 6.1 | 5.8 | Inf | 5.35E-09 | 5.59 | 4.32E-10 | 1055 |
| Cluster-40555.159382 | 0 | 0 | 18.72 | 16 | 0.37 | 1.33 | 6.39 | 7.2 | Inf | 3.12E-18 | 3.05 | 2.87E-03 | 674 |
| Cluster-40555.159442 | 0 | 0 | 1.73 | 1.74 | 0 | 0.36 | 2.01 | 1.51 | Inf | 1.46E-06 | 3.27 | 1.97E-03 | 1723 |
| Cluster-40555.159445 | 0 | 0 | 6.56 | 7.01 | 0.36 | 0.37 | 7.05 | 9.38 | Inf | 9.65E-05 | 4.56 | 4.66E-04 | 533 |
| Cluster-40555.159460 | 0 | 0 | 1.59 | 2.22 | 0 | 0.46 | 2.08 | 3.6 | Inf | 3.35E-07 | 3.69 | 2.88E-03 | 1685 |
| Cluster-40555.159541 | 0 | 0 | 1.41 | 0.82 | 0 | 0.26 | 1.99 | 1.42 | Inf | 1.46E-07 | 3.76 | 1.08E-06 | 2957 |
| Cluster-40555.159614 | 0 | 0 | 2.57 | 2.42 | 0 | 0 | 2.7 | 2.06 | Inf | 6.48E-07 | Inf | 3.57E-07 | 1332 |
| Cluster-40555.159642 | 0 | 0 | 4.29 | 4.07 | 0 | 0 | 4.41 | 5.82 | Inf | 5.39E-08 | Inf | 1.28E-10 | 1000 |
| Cluster-40555.159792 | 0 | 0 | 7.83 | 10.65 | 0.13 | 0 | 14.65 | 10.27 | Inf | 4.94E-08 | 7.53 | 2.54E-10 | 590 |
| Cluster-40555.159806 | 0 | 0 | 2.92 | 4.14 | 0.88 | 0.86 | 3.24 | 2.44 | Inf | 3.99E-18 | 1.77 | 2.31E-03 | 3119 |
| Cluster-40555.159941 | 0 | 0 | 1.05 | 1.32 | 0 | 0 | 0.71 | 0.91 | Inf | 1.85E-05 | Inf | 5.42E-04 | 2039 |
| Cluster-40555.159997 | 0 | 0 | 7.02 | 7.26 | 1.49 | 0.62 | 10.4 | 14.24 | Inf | 5.78E-03 | 3.62 | 7.62E-03 | 427 |
| Cluster-40555.160120 | 0 | 0 | 15.11 | 21.29 | 1.82 | 3.65 | 13.66 | 15.02 | Inf | 5.59E-19 | 2.44 | 1.50E-05 | 832 |
| Cluster-40555.160140 | 0 | 0 | 0.28 | 0.24 | 0.37 | 0.51 | 1.36 | 2.01 | Inf | 8.58E-03 | 2.00 | 8.58E-03 | 4562 |
| Cluster-40555.160232 | 0 | 0 | 2.61 | 0.8 | 0.28 | 0 | 2.38 | 2.36 | Inf | 2.50E-02 | 4.11 | 2.96E-04 | 1371 |
| Cluster-40555.160369 | 0 | 0 | 3.54 | 4.82 | 0 | 0 | 9.09 | 7.13 | Inf | 1.54E-03 | Inf | 2.14E-07 | 589 |
| Cluster-40555.160574 | 0 | 0 | 6.34 | 8.04 | 0 | 0 | 3.37 | 4.25 | Inf | 6.45E-12 | Inf | 9.16E-07 | 890 |
| Cluster-40555.160650 | 0 | 0 | 2.15 | 2.01 | 0.25 | 0 | 4.36 | 7.07 | Inf | 5.59E-03 | 5.65 | 7.73E-06 | 834 |
| Cluster-40555.160654 | 0 | 0 | 1.74 | 1.41 | 0.96 | 0.8 | 4.32 | 5.26 | Inf | 2.84E-05 | 2.51 | 9.59E-05 | 1585 |
| Cluster-40555.160727 | 0 | 0 | 0.86 | 0.71 | 0.04 | 0 | 0.48 | 0.89 | Inf | 1.27E-02 | 5.43 | 4.76E-02 | 1589 |
| Cluster-40555.160743 | 0 | 0 | 5.78 | 4.86 | 0.29 | 0 | 4.4 | 6.06 | Inf | 1.19E-17 | 5.32 | 2.17E-11 | 1566 |
| Cluster-40555.160744 | 0 | 0 | 2.72 | 3.55 | 0.07 | 0.05 | 3.37 | 4.21 | Inf | 1.83E-11 | 6.30 | 4.47E-12 | 1647 |
| Cluster-40555.160746 | 0 | 0 | 0.62 | 0.55 | 0 | 0 | 0.29 | 0.47 | Inf | 4.73E-03 | Inf | 3.86E-02 | 2362 |
| Cluster-40555.160804 | 0 | 0 | 4.14 | 4.72 | 0 | 0 | 2.07 | 4.19 | Inf | 2.95E-16 | Inf | 6.75E-05 | 1673 |
| Cluster-40555.160888 | 0 | 0 | 2.66 | 2.16 | 0.12 | 0 | 3.39 | 2.16 | Inf | 2.61E-02 | 5.63 | 2.05E-02 | 655 |
| Cluster-40555.161057 | 0 | 0 | 9.39 | 8.12 | 0 | 0 | 8.51 | 8.37 | Inf | 2.67E-06 | Inf | 1.88E-06 | 541 |
| Cluster-40555.161251 | 0 | 0 | 0.3 | 0.36 | 0 | 0 | 0.22 | 0.34 | Inf | 9.50E-03 | Inf | 1.41E-02 | 3572 |
| Cluster-40555.161307 | 0 | 0 | 2.86 | 3.51 | 0.53 | 0.59 | 4 | 4.18 | Inf | 7.10E-14 | 2.92 | 2.72E-06 | 1920 |
| Cluster-40555.161341 | 0 | 0 | 1.23 | 1.84 | 0 | 0 | 0.99 | 1.32 | Inf | 3.78E-03 | Inf | 1.68E-02 | 1065 |
| Cluster-40555.161653 | 0 | 0 | 2.39 | 3.41 | 0 | 0 | 4.28 | 6.08 | Inf | 4.31E-04 | Inf | 5.92E-08 | 808 |

| | | | | | | | | | | | | | |
|---|---|---|---|---|---|---|---|---|---|---|---|---|---|
| Cluster-40555.161792 | 0 | 0 | 3.54 | 3.6 | 0 | 0 | 2.41 | 6.75 | Inf | 6.45E-06 | Inf | 1.44E-02 | 897 |
| Cluster-40555.161879 | 0 | 0 | 3.63 | 2.86 | 0 | 0 | 2.15 | 4.47 | Inf | 1.67E-07 | Inf | 4.06E-04 | 1161 |
| Cluster-40555.161917 | 0 | 0 | 2.12 | 3.35 | 0 | 0 | 3.41 | 2.7 | Inf | 4.47E-04 | Inf | 8.44E-05 | 832 |
| Cluster-40555.162041 | 0 | 0 | 16.91 | 18.69 | 0 | 0 | 8.66 | 5.1 | Inf | 7.43E-15 | Inf | 4.76E-06 | 582 |
| Cluster-40555.162076 | 0 | 0 | 6.23 | 6.58 | 0 | 0.18 | 2.63 | 1.77 | Inf | 2.61E-23 | 4.70 | 4.35E-06 | 1704 |
| Cluster-40555.162095 | 0 | 0 | 8.52 | 5.9 | 0 | 0 | 6.21 | 6.16 | Inf | 2.08E-10 | Inf | 8.23E-10 | 825 |
| Cluster-40555.162120 | 0 | 0 | 1.35 | 1.41 | 0 | 0 | 1.34 | 1.21 | Inf | 1.17E-03 | Inf | 9.39E-04 | 1311 |
| Cluster-40555.162200 | 0 | 0 | 0.57 | 0.43 | 0 | 0 | 0.25 | 0.33 | Inf | 2.31E-05 | Inf | 2.15E-03 | 4478 |
| Cluster-40555.162392 | 0 | 0 | 16.95 | 16.06 | 0 | 0 | 11.47 | 6.53 | Inf | 2.50E-06 | Inf | 1.94E-03 | 409 |
| Cluster-40555.162408 | 0 | 0 | 23.09 | 21.23 | 1.63 | 0 | 25.54 | 22.15 | Inf | 5.79E-60 | 4.99 | 5.22E-32 | 1589 |
| Cluster-40555.162826 | 0 | 0 | 4.29 | 5.44 | 0.21 | 0.41 | 2.98 | 3.23 | Inf | 7.88E-22 | 3.35 | 1.63E-06 | 2013 |
| Cluster-40555.163012 | 0 | 0 | 1.57 | 1.77 | 0.26 | 0.15 | 1.35 | 1.62 | Inf | 3.67E-07 | 2.97 | 8.70E-03 | 1904 |
| Cluster-40555.163112 | 0 | 0 | 9.8 | 7.76 | 0 | 0 | 4.12 | 5.28 | Inf | 5.57E-04 | Inf | 3.92E-02 | 440 |
| Cluster-40555.163131 | 0 | 0 | 0.63 | 0.81 | 0.1 | 0 | 0.8 | 0.67 | Inf | 5.27E-05 | 3.94 | 3.52E-03 | 2942 |
| Cluster-40555.163633 | 0 | 0 | 1.19 | 0.84 | 0 | 0 | 0.89 | 1.32 | Inf | 1.04E-02 | Inf | 3.01E-03 | 1340 |
| Cluster-40555.163727 | 0 | 0 | 37.32 | 43.84 | 1.58 | 0 | 86 | 59.17 | Inf | 3.65E-18 | 6.51 | 6.82E-16 | 434 |
| Cluster-40555.163766 | 0 | 0 | 19.03 | 7.68 | 0 | 0 | 4.54 | 2.64 | Inf | 4.17E-05 | Inf | 2.03E-06 | 902 |
| Cluster-40555.163873 | 0 | 0 | 1.21 | 2.34 | 0.19 | 0.14 | 1.59 | 1.88 | Inf | 3.53E-05 | 3.47 | 8.09E-03 | 1415 |
| Cluster-40555.163913 | 0 | 0 | 2.68 | 2.75 | 0.19 | 0 | 3.43 | 2.5 | Inf | 1.81E-03 | 5.10 | 4.37E-03 | 767 |
| Cluster-40555.163968 | 0 | 0 | 0.79 | 0.58 | 0 | 0 | 1.07 | 0.81 | Inf | 5.04E-03 | Inf | 1.01E-04 | 2052 |
| Cluster-40555.164075 | 0 | 0 | 17.29 | 11.71 | 0 | 0 | 8.92 | 12.13 | Inf | 8.56E-18 | Inf | 4.13E-14 | 749 |
| Cluster-40555.164101 | 0 | 0 | 0.98 | 1.31 | 0 | 0 | 0.69 | 1.56 | Inf | 2.31E-02 | Inf | 1.93E-02 | 1070 |
| Cluster-40555.164447 | 0 | 0 | 1.29 | 1.35 | 0 | 0 | 2.75 | 2.1 | Inf | 4.98E-02 | Inf | 4.03E-04 | 878 |
| Cluster-40555.164631 | 0 | 0 | 11.45 | 14.67 | 0.05 | 0 | 17.15 | 18.08 | Inf | 1.96E-29 | 9.53 | 8.95E-35 | 1171 |
| Cluster-40555.165074 | 0 | 0 | 1.49 | 1.49 | 0 | 0 | 2.89 | 6.34 | Inf | 5.37E-09 | Inf | 3.70E-05 | 2578 |
| Cluster-40555.165134 | 0 | 0 | 5.28 | 4.32 | 0.2 | 0.35 | 3.32 | 5.11 | Inf | 1.04E-08 | 3.99 | 9.45E-05 | 966 |
| Cluster-40555.165262 | 0 | 0 | 1.08 | 1.67 | 0.11 | 0 | 1.75 | 1.71 | Inf | 8.93E-03 | 4.97 | 8.86E-03 | 1056 |
| Cluster-40555.165411 | 0 | 0 | 1.12 | 1.1 | 0 | 0 | 0.61 | 1.39 | Inf | 2.79E-02 | Inf | 3.21E-02 | 1089 |
| Cluster-40555.165532 | 0 | 0 | 0.57 | 0.47 | 0 | 0.18 | 1.31 | 1.17 | Inf | 3.07E-02 | 3.88 | 1.60E-03 | 1989 |
| Cluster-40555.165734 | 0 | 0 | 5.31 | 6.39 | 0 | 0 | 1.35 | 2.12 | Inf | 8.74E-15 | Inf | 1.11E-04 | 1231 |
| Cluster-40555.165800 | 0 | 0 | 5.51 | 3.79 | 0 | 0 | 4.05 | 2.45 | Inf | 6.66E-07 | Inf | 3.12E-05 | 844 |
| Cluster-40555.165810 | 0 | 0 | 0.49 | 0.27 | 0 | 0 | 0.91 | 0.74 | Inf | 5.21E-03 | Inf | 1.52E-07 | 3467 |

| | | | | | | | | | | | | | |
|---|---|---|---|---|---|---|---|---|---|---|---|---|---|
| Cluster-40555.165983 | 0 | 0 | 3.16 | 2.45 | 0.96 | 0.98 | 4.88 | 2.67 | Inf | 1.55E-10 | 2.02 | 4.61E-02 | 1721 |
| Cluster-40555.166373 | 0 | 0 | 6.52 | 6.12 | 0 | 0 | 0.91 | 1.74 | Inf | 8.75E-31 | Inf | 6.87E-05 | 2274 |
| Cluster-40555.166459 | 0 | 0 | 3.55 | 2.35 | 0 | 0 | 3.99 | 3.96 | Inf | 3.27E-02 | Inf | 3.12E-03 | 566 |
| Cluster-40555.166480 | 0 | 0 | 3.22 | 2.1 | 0 | 0 | 1.79 | 1.29 | Inf | 2.83E-05 | Inf | 2.26E-03 | 1054 |
| Cluster-40555.166505 | 0 | 0 | 2.51 | 2.25 | 0 | 0 | 2.61 | 1.88 | Inf | 2.19E-12 | Inf | 1.09E-12 | 2272 |
| Cluster-40555.166523 | 0 | 0 | 13.92 | 11.19 | 0.14 | 0.15 | 12.87 | 11.33 | Inf | 6.94E-43 | 6.47 | 9.17E-32 | 1782 |
| Cluster-40555.166683 | 0 | 0 | 4.83 | 4.27 | 0 | 0 | 7.69 | 8.54 | Inf | 2.04E-06 | Inf | 2.12E-12 | 810 |
| Cluster-40555.166732 | 0 | 0 | 9.59 | 8.66 | 0 | 0 | 7.49 | 10.72 | Inf | 8.47E-07 | Inf | 3.22E-07 | 550 |
| Cluster-40555.166783 | 0 | 0 | 18.28 | 19.74 | 2.3 | 0 | 17.64 | 22.04 | Inf | 2.03E-04 | 4.21 | 9.20E-03 | 349 |
| Cluster-40555.166785 | 0 | 0 | 9.91 | 12.63 | 0 | 0.1 | 10.13 | 14.65 | Inf | 1.04E-05 | Inf | 1.78E-06 | 456 |
| Cluster-40555.166989 | 0 | 0 | 0.4 | 0.54 | 0 | 0 | 0.41 | 0.19 | Inf | 3.65E-03 | Inf | 4.10E-02 | 2894 |
| Cluster-40555.167075 | 0 | 0 | 2.34 | 2.33 | 0 | 0 | 1.76 | 1.29 | Inf | 3.50E-06 | Inf | 2.42E-04 | 1280 |
| Cluster-40555.167220 | 0 | 0 | 4.12 | 3.79 | 0.08 | 0 | 1.57 | 1.47 | Inf | 1.00E-06 | 5.49 | 3.66E-02 | 921 |
| Cluster-40555.167360 | 0 | 0 | 5.08 | 6.46 | 0.04 | 0.17 | 3.36 | 4.13 | Inf | 5.47E-17 | 5.16 | 1.45E-08 | 1391 |
| Cluster-40555.167604 | 0 | 0 | 2.8 | 1.76 | 0 | 0 | 2.11 | 1.19 | Inf | 2.59E-03 | Inf | 1.27E-02 | 854 |
| Cluster-40555.167644 | 0 | 0 | 1.51 | 1.6 | 0 | 0 | 2.92 | 1.97 | Inf | 2.89E-02 | Inf | 6.85E-04 | 846 |
| Cluster-40555.167826 | 0 | 0 | 25.4 | 21.32 | 0 | 0 | 22.96 | 16.29 | Inf | 8.28E-03 | Inf | 4.05E-02 | 300 |
| Cluster-40555.167877 | 0 | 0 | 4.02 | 3.99 | 0 | 0 | 3.6 | 1.1 | Inf | 1.80E-07 | Inf | 4.50E-02 | 977 |
| Cluster-40555.167994 | 0 | 0 | 45.62 | 38.62 | 0 | 0 | 23.22 | 35.87 | Inf | 1.61E-16 | Inf | 1.13E-10 | 414 |
| Cluster-40555.168102 | 0 | 0 | 5.52 | 7.78 | 0.21 | 0 | 7.22 | 7.23 | Inf | 4.41E-15 | 6.17 | 1.31E-13 | 1126 |
| Cluster-40555.168157 | 0 | 0 | 0.95 | 1.75 | 0 | 0 | 0.74 | 1.67 | Inf | 8.90E-06 | Inf | 2.72E-03 | 1935 |
| Cluster-40555.168426 | 0 | 0 | 0.67 | 0.87 | 0 | 0 | 0.41 | 0.56 | Inf | 8.40E-04 | Inf | 1.42E-02 | 2190 |
| Cluster-40555.168612 | 0 | 0 | 24.68 | 24.92 | 0 | 0.1 | 20.55 | 16.67 | Inf | 1.41E-30 | 8.58 | 9.98E-23 | 768 |
| Cluster-40555.168661 | 0 | 0 | 0.47 | 0.65 | 0 | 0 | 0.32 | 0.65 | Inf | 1.82E-03 | Inf | 3.78E-03 | 2673 |
| Cluster-40555.168694 | 0 | 0 | 2.47 | 2.86 | 0 | 0 | 1.65 | 2.18 | Inf | 3.63E-05 | Inf | 6.43E-04 | 1003 |
| Cluster-40555.168736 | 0 | 0 | 2.43 | 2.16 | 0 | 0 | 1.46 | 1.74 | Inf | 2.24E-05 | Inf | 3.86E-04 | 1187 |
| Cluster-40555.169004 | 0 | 0 | 0.74 | 2.06 | 0 | 0 | 1.43 | 1.32 | Inf | 1.18E-02 | Inf | 1.11E-03 | 1234 |
| Cluster-40555.169137 | 0 | 0 | 2.93 | 3.83 | 0 | 0 | 3.44 | 1.77 | Inf | 1.74E-10 | Inf | 5.32E-06 | 1436 |
| Cluster-40555.169158 | 0 | 0 | 3.88 | 7.83 | 0 | 0.98 | 8.26 | 3.98 | Inf | 2.54E-05 | 3.68 | 1.29E-02 | 884 |
| Cluster-40555.169519 | 0 | 0 | 6.67 | 7.05 | 0.27 | 0.1 | 4.69 | 4.36 | Inf | 4.94E-12 | 4.77 | 9.68E-06 | 934 |
| Cluster-40555.169559 | 0 | 0 | 0.99 | 0.72 | 0 | 0 | 0.75 | 0.83 | Inf | 5.09E-07 | Inf | 2.83E-07 | 3469 |
| Cluster-40555.170129 | 0 | 0 | 5.87 | 5.74 | 1.16 | 1.15 | 5.15 | 7.84 | Inf | 1.97E-21 | 2.58 | 1.74E-03 | 1723 |

| | | | | | | | | | | | | | |
|---|---|---|---|---|---|---|---|---|---|---|---|---|---|
| Cluster-40555.170194 | 0 | 0 | 7.93 | 7.28 | 0.34 | 0.37 | 6.77 | 6.14 | Inf | 1.82E-33 | 4.22 | 1.66E-15 | 2107 |
| Cluster-40555.170295 | 0 | 0 | 4.39 | 3.89 | 0 | 0 | 3.26 | 3.1 | Inf | 1.39E-11 | Inf | 8.28E-10 | 1346 |
| Cluster-40555.170344 | 0 | 0 | 8.87 | 8.84 | 0 | 0 | 6.14 | 8.91 | Inf | 2.64E-35 | Inf | 5.67E-16 | 1941 |
| Cluster-40555.170504 | 0 | 0 | 2.93 | 2.62 | 0 | 0 | 2.08 | 2.42 | Inf | 4.61E-07 | Inf | 3.03E-06 | 1244 |
| Cluster-40555.170509 | 0 | 0 | 1.18 | 1.1 | 0 | 0 | 2.11 | 2.14 | Inf | 1.84E-06 | Inf | 3.90E-13 | 2454 |
| Cluster-40555.170539 | 0 | 0 | 3.92 | 4.28 | 0.03 | 0.03 | 3.6 | 4.43 | Inf | 7.23E-21 | 7.46 | 4.19E-19 | 2258 |
| Cluster-40555.170554 | 0 | 0 | 2.59 | 3 | 0 | 0 | 2.26 | 1.37 | Inf | 3.44E-16 | Inf | 1.69E-10 | 2494 |
| Cluster-40555.170787 | 0 | 0 | 10.15 | 13.63 | 0 | 0 | 4.72 | 6.13 | Inf | 7.16E-05 | Inf | 3.92E-02 | 418 |
| Cluster-40555.170802 | 0 | 0 | 1.93 | 1.95 | 0 | 0.57 | 3.32 | 4.16 | Inf | 7.26E-09 | 3.76 | 6.90E-09 | 2014 |
| Cluster-40555.171065 | 0 | 0 | 14.07 | 15.06 | 0.26 | 0.46 | 54.46 | 33.39 | Inf | 3.72E-09 | 7.16 | 4.39E-11 | 500 |
| Cluster-40555.171120 | 0 | 0 | 3.13 | 3.86 | 0.12 | 0.07 | 5.05 | 3.26 | Inf | 3.41E-08 | 5.77 | 3.18E-08 | 1155 |
| Cluster-40555.171232 | 0 | 0 | 1.06 | 1.65 | 0 | 0.09 | 1.35 | 1.5 | Inf | 2.51E-04 | 5.14 | 1.10E-03 | 1479 |
| Cluster-40555.171252 | 0 | 0 | 2.68 | 2.99 | 0 | 0.27 | 3.38 | 2.51 | Inf | 6.05E-06 | 4.61 | 1.39E-04 | 1074 |
| Cluster-40555.171429 | 0 | 0 | 0.67 | 2.75 | 0 | 0 | 5.77 | 3.1 | Inf | 3.08E-02 | Inf | 1.10E-09 | 2654 |
| Cluster-40555.171615 | 0 | 0 | 10.51 | 12.06 | 0.54 | 0 | 11.04 | 8.03 | Inf | 2.68E-05 | 5.24 | 1.81E-03 | 442 |
| Cluster-40555.171800 | 0 | 0 | 2.02 | 3.63 | 0 | 0 | 3.45 | 3.94 | Inf | 1.15E-07 | Inf | 8.82E-20 | 2245 |
| Cluster-40555.171854 | 0 | 0 | 11.95 | 10.39 | 0 | 0 | 7.61 | 8.44 | Inf | 7.42E-38 | Inf | 8.18E-29 | 1721 |
| Cluster-40555.171911 | 0 | 0 | 4.89 | 4.71 | 0 | 0 | 2.8 | 4.02 | Inf | 1.35E-20 | Inf | 4.07E-13 | 1957 |
| Cluster-40555.171948 | 0 | 0 | 9.12 | 11.13 | 0 | 0.17 | 5.64 | 7.67 | Inf | 3.52E-14 | 6.18 | 2.41E-08 | 797 |
| Cluster-40555.172099 | 0 | 0 | 5.39 | 4.76 | 0 | 0 | 4.44 | 3.56 | Inf | 3.07E-14 | Inf | 2.69E-12 | 1351 |
| Cluster-40555.172238 | 0 | 0 | 8.68 | 14.32 | 0 | 0 | 10.73 | 10.45 | Inf | 1.29E-12 | Inf | 1.13E-36 | 1796 |
| Cluster-40555.172260 | 0 | 0 | 0.63 | 0.42 | 0 | 0 | 2.18 | 1.28 | Inf | 1.19E-02 | Inf | 1.73E-09 | 2293 |
| Cluster-40555.172308 | 0 | 0 | 3.45 | 1.75 | 0.05 | 0 | 1.4 | 2.87 | Inf | 3.84E-06 | 6.92 | 1.22E-03 | 1472 |
| Cluster-40555.172339 | 0 | 0 | 2.56 | 3.06 | 0.07 | 0.15 | 4.3 | 5.68 | Inf | 6.67E-05 | 5.57 | 2.49E-07 | 935 |
| Cluster-40555.172427 | 0 | 0 | 0.87 | 1.03 | 0 | 0 | 1.22 | 0.84 | Inf | 8.49E-06 | Inf | 7.16E-07 | 2617 |
| Cluster-40555.172504 | 0 | 0 | 3.57 | 2.66 | 0 | 0 | 2.49 | 1.13 | Inf | 1.96E-08 | Inf | 5.78E-04 | 1320 |
| Cluster-40555.172526 | 0 | 0 | 0.97 | 1.19 | 0.15 | 0.57 | 1.44 | 1.64 | Inf | 2.42E-05 | 2.14 | 3.40E-02 | 2158 |
| Cluster-40555.172632 | 0 | 0 | 9.93 | 10.75 | 1.98 | 0.78 | 10.77 | 12.29 | Inf | 2.58E-05 | 3.15 | 1.09E-02 | 459 |
| Cluster-40555.172764 | 0 | 0 | 1.68 | 1.51 | 0.16 | 0 | 1.56 | 2.21 | Inf | 4.73E-03 | 4.48 | 1.13E-02 | 1026 |
| Cluster-40555.172875 | 0 | 0 | 5.24 | 5.57 | 0.41 | 0 | 5.62 | 3.91 | Inf | 2.96E-05 | 4.76 | 1.95E-03 | 638 |
| Cluster-40555.172890 | 0 | 0 | 2.36 | 1.93 | 0 | 0 | 1.66 | 1.97 | Inf | 3.13E-04 | Inf | 5.46E-04 | 1045 |
| Cluster-40555.173027 | 0 | 0 | 1.75 | 5.42 | 0.24 | 0 | 3.7 | 3.5 | Inf | 8.64E-03 | 4.94 | 2.16E-06 | 1179 |

| | | | | | | | | | | | | | |
|---|---|---|---|---|---|---|---|---|---|---|---|---|---|
| Cluster-40555.173053 | 0 | 0 | 9.41 | 13.33 | 0 | 0 | 8.58 | 6.14 | Inf | 1.92E-10 | Inf | 4.29E-07 | 608 |
| Cluster-40555.173210 | 0 | 0 | 0.75 | 0.27 | 0 | 0 | 0.63 | 0.4 | Inf | 3.72E-02 | Inf | 1.49E-02 | 2081 |
| Cluster-40555.173239 | 0 | 0 | 3.64 | 2.27 | 0 | 0.15 | 2.33 | 2.76 | Inf | 1.37E-13 | 5.15 | 3.18E-10 | 2272 |
| Cluster-40555.173241 | 0 | 0 | 4.77 | 3.92 | 1.9 | 1.57 | 4.27 | 5.61 | Inf | 3.17E-26 | 1.58 | 1.79E-03 | 2726 |
| Cluster-40555.173272 | 0 | 0 | 32.12 | 30.99 | 0 | 0 | 5.74 | 7.68 | Inf | 1.02E-14 | Inf | 3.61E-03 | 441 |
| Cluster-40555.173312 | 0 | 0 | 2.21 | 1.56 | 0 | 0.61 | 2.81 | 2.18 | Inf | 8.31E-04 | 3.05 | 1.47E-02 | 1067 |
| Cluster-40555.173801 | 0 | 0 | 1.16 | 2.38 | 0 | 0 | 0.83 | 1.05 | Inf | 5.89E-04 | Inf | 3.92E-02 | 1109 |
| Cluster-40555.173812 | 0 | 0 | 3.57 | 4.85 | 0.67 | 1.19 | 4.59 | 6.34 | Inf | 2.47E-07 | 2.63 | 2.46E-03 | 921 |
| Cluster-40555.174130 | 0 | 0 | 8.72 | 9.05 | 0.6 | 0.61 | 9.49 | 6.65 | Inf | 1.43E-07 | 3.81 | 5.72E-04 | 589 |
| Cluster-40555.174140 | 0 | 0 | 26.38 | 29.83 | 0.79 | 1.49 | 21.85 | 34.92 | Inf | 1.92E-09 | 4.57 | 4.33E-06 | 388 |
| Cluster-40555.174374 | 0 | 0 | 5.77 | 5.04 | 0 | 0 | 5.65 | 6.26 | Inf | 9.93E-05 | Inf | 1.22E-05 | 604 |
| Cluster-40555.174385 | 0 | 0 | 13.54 | 36.87 | 0 | 0 | 38.75 | 35.3 | Inf | 3.94E-03 | Inf | 3.17E-09 | 357 |
| Cluster-40555.174498 | 0 | 0 | 25.18 | 23.48 | 0.04 | 0 | 27.95 | 27.19 | Inf | 2.30E-36 | 9.65 | 6.52E-37 | 897 |
| Cluster-40555.174549 | 0 | 0 | 5.4 | 3.72 | 0 | 0 | 5.54 | 4.67 | Inf | 5.93E-21 | Inf | 2.55E-24 | 2111 |
| Cluster-40555.174683 | 0 | 0 | 7.77 | 6.61 | 0.48 | 0 | 9.2 | 10.81 | Inf | 1.67E-26 | 5.54 | 4.76E-24 | 1765 |
| Cluster-40555.174842 | 0 | 0 | 7.56 | 9.82 | 0 | 0 | 4.46 | 4.58 | Inf | 2.57E-28 | Inf | 7.86E-18 | 1706 |
| Cluster-40555.175030 | 0 | 0 | 5.14 | 3.07 | 0 | 0 | 5.23 | 5.53 | Inf | 8.94E-05 | Inf | 4.75E-07 | 728 |
| Cluster-40555.175054 | 0 | 0 | 1.17 | 0.88 | 0.04 | 0 | 2.47 | 1.67 | Inf | 3.27E-03 | 6.92 | 1.26E-06 | 1530 |
| Cluster-40555.175057 | 0 | 0 | 6 | 5.99 | 0.21 | 0.5 | 5.13 | 5.51 | Inf | 1.23E-14 | 3.92 | 2.19E-07 | 1202 |
| Cluster-40555.175141 | 0 | 0 | 27.98 | 26.72 | 0 | 0 | 20.56 | 11.19 | Inf | 3.83E-30 | Inf | 8.60E-09 | 714 |
| Cluster-40555.175229 | 0 | 0 | 0.7 | 0.27 | 0 | 0 | 0.84 | 0.46 | Inf | 3.84E-02 | Inf | 2.30E-03 | 2139 |
| Cluster-40555.175466 | 0 | 0 | 6.14 | 6 | 0 | 0.11 | 5.4 | 5.71 | Inf | 9.66E-26 | 6.62 | 1.45E-20 | 1970 |
| Cluster-40555.175507 | 0 | 0 | 16.07 | 16.01 | 0 | 0 | 9.52 | 9.13 | Inf | 3.73E-42 | Inf | 7.04E-27 | 1411 |
| Cluster-40555.175616 | 0 | 0 | 29.62 | 22.83 | 3.04 | 0 | 36.48 | 31.26 | Inf | 1.57E-03 | 4.56 | 6.79E-03 | 307 |
| Cluster-40555.175670 | 0 | 0 | 2.09 | 2.16 | 0 | 0 | 2.13 | 3.43 | Inf | 9.73E-07 | Inf | 6.03E-08 | 1487 |
| Cluster-40555.175695 | 0 | 0 | 1.93 | 1.21 | 0.15 | 0.25 | 1.59 | 1.6 | Inf | 8.10E-06 | 3.07 | 7.95E-03 | 1732 |
| Cluster-40555.175823 | 0 | 0 | 3.45 | 4.66 | 1.84 | 1.41 | 7.03 | 5.52 | Inf | 1.18E-07 | 2.02 | 1.10E-02 | 974 |
| Cluster-40555.175957 | 0 | 0 | 4.15 | 3.6 | 0 | 0 | 3.57 | 4.72 | Inf | 8.90E-11 | Inf | 1.48E-12 | 1338 |
| Cluster-40555.176175 | 0 | 0 | 10.52 | 10.29 | 0 | 0 | 9.13 | 9.17 | Inf | 3.92E-33 | Inf | 1.32E-29 | 1590 |
| Cluster-40555.176356 | 0 | 0 | 2.03 | 2.72 | 0.11 | 0.61 | 5.49 | 5.19 | Inf | 1.76E-03 | 3.96 | 7.69E-05 | 830 |
| Cluster-40555.176440 | 0 | 0 | 8.76 | 18.23 | 0 | 0 | 13.4 | 12.22 | Inf | 1.84E-03 | Inf | 4.42E-03 | 357 |
| Cluster-40555.176665 | 0 | 0 | 2.02 | 1.61 | 0.28 | 1.08 | 3.13 | 3.06 | Inf | 1.60E-06 | 2.22 | 3.73E-03 | 1674 |

| | | | | | | | | | | | | | |
|---|---|---|---|---|---|---|---|---|---|---|---|---|---|
| Cluster-40555.176863 | 0 | 0 | 6.86 | 7.07 | 0.12 | 0.17 | 6.43 | 9.22 | Inf | 1.44E-15 | 5.87 | 6.54E-11 | 1127 |
| Cluster-40555.177008 | 0 | 0 | 4.05 | 2.48 | 0 | 0 | 4.53 | 3.88 | Inf | 4.71E-03 | Inf | 2.00E-04 | 646 |
| Cluster-40555.177041 | 0 | 0 | 6.6 | 4 | 0.47 | 0.65 | 12.07 | 10.68 | Inf | 4.85E-04 | 4.39 | 5.70E-06 | 569 |
| Cluster-40555.177205 | 0 | 0 | 7.25 | 7.65 | 0 | 0 | 1.99 | 2.12 | Inf | 4.22E-11 | Inf | 3.66E-03 | 830 |
| Cluster-40555.177278 | 0 | 0 | 3.66 | 3.71 | 0 | 0 | 2.84 | 4.85 | Inf | 6.35E-09 | Inf | 8.05E-07 | 1187 |
| Cluster-40555.177292 | 0 | 0 | 1.32 | 1.28 | 0 | 0.15 | 1.49 | 1.27 | Inf | 3.73E-05 | 4.12 | 1.05E-03 | 1797 |
| Cluster-40555.177299 | 0 | 0 | 0.56 | 0.96 | 0 | 0 | 1.13 | 0.89 | Inf | 1.25E-02 | Inf | 9.53E-04 | 1621 |
| Cluster-40555.177363 | 0 | 0 | 5.44 | 7.28 | 0 | 0 | 20.93 | 17.13 | Inf | 2.07E-14 | Inf | 9.48E-37 | 1123 |
| Cluster-40555.177412 | 0 | 0 | 6.52 | 2.09 | 0 | 0 | 2.65 | 2.7 | Inf | 3.21E-03 | Inf | 7.03E-09 | 1432 |
| Cluster-40555.177558 | 0 | 0 | 39.78 | 58.14 | 0 | 0 | 69.63 | 56.72 | Inf | 3.65E-05 | Inf | 7.68E-06 | 289 |
| Cluster-40555.177741 | 0 | 0 | 0.59 | 0.54 | 0 | 0 | 0.62 | 0.34 | Inf | 5.59E-03 | Inf | 9.03E-03 | 2385 |
| Cluster-40555.177861 | 0 | 0 | 2.78 | 2.35 | 0 | 0 | 1.05 | 1.34 | Inf | 3.94E-15 | Inf | 7.15E-08 | 2559 |
| Cluster-40555.177920 | 0 | 0 | 1.63 | 0.86 | 0.57 | 0 | 2.16 | 1.86 | Inf | 1.77E-06 | 2.96 | 3.35E-04 | 2342 |
| Cluster-40555.178178 | 0 | 0 | 7.29 | 7.39 | 0 | 0 | 1.42 | 2.29 | Inf | 5.30E-11 | Inf | 7.08E-03 | 836 |
| Cluster-40555.178231 | 0 | 0 | 6.02 | 3.07 | 0.25 | 0.26 | 5.41 | 10.31 | Inf | 4.09E-02 | 4.98 | 5.42E-03 | 455 |
| Cluster-40555.178255 | 0 | 0 | 3.66 | 4 | 0 | 0 | 3.9 | 2.48 | Inf | 3.50E-05 | Inf | 1.22E-04 | 790 |
| Cluster-40555.178483 | 0 | 0 | 3.42 | 2.98 | 0 | 0 | 1.46 | 2.51 | Inf | 9.50E-23 | Inf | 2.44E-07 | 3105 |
| Cluster-40555.178523 | 0 | 0 | 87.45 | 88.31 | 26.01 | 26.34 | 84.61 | 118.09 | Inf | 3.90E-24 | 1.99 | 1.52E-04 | 369 |
| Cluster-40555.178525 | 0 | 0 | 1.21 | 0.43 | 0.34 | 0.3 | 1.35 | 1.58 | Inf | 1.35E-02 | 2.24 | 4.75E-02 | 2028 |
| Cluster-40555.178551 | 0 | 0 | 53.87 | 54.63 | 0 | 0 | 24.45 | 19.15 | Inf | 5.84E-48 | Inf | 2.06E-22 | 661 |
| Cluster-40555.178662 | 0 | 0 | 11.94 | 10.2 | 0 | 0 | 2.06 | 1.22 | Inf | 1.32E-30 | Inf | 2.63E-05 | 1409 |
| Cluster-40555.178663 | 0 | 0 | 9.06 | 10.72 | 0 | 0 | 9.17 | 11.28 | Inf | 4.24E-18 | Inf | 2.56E-19 | 968 |
| Cluster-40555.178671 | 0 | 0 | 18.28 | 19.74 | 0 | 0 | 4.16 | 3.17 | Inf | 8.40E-39 | Inf | 4.56E-09 | 1135 |
| Cluster-40555.178705 | 0 | 0 | 8.97 | 11.68 | 5.11 | 3.41 | 26.33 | 20.24 | Inf | 3.91E-26 | 2.52 | 2.26E-10 | 1267 |
| Cluster-40555.178828 | 0 | 0 | 21.41 | 19.8 | 0 | 0.07 | 5.19 | 6.76 | Inf | 9.05E-33 | 7.35 | 3.45E-10 | 926 |
| Cluster-40555.178831 | 0 | 0 | 4.59 | 2.81 | 0 | 0 | 3.81 | 1.29 | Inf | 3.28E-07 | Inf | 1.82E-02 | 1032 |
| Cluster-40555.179201 | 0 | 0 | 9.33 | 9.4 | 0 | 0.18 | 5.65 | 5.72 | Inf | 2.42E-21 | 6.13 | 1.25E-11 | 1161 |
| Cluster-40555.179255 | 0 | 0 | 4.69 | 3.82 | 0 | 0 | 2.76 | 3.02 | Inf | 4.22E-18 | Inf | 1.30E-13 | 1939 |
| Cluster-40555.179322 | 0 | 0 | 11.99 | 9.89 | 0 | 0 | 4.08 | 4.78 | Inf | 2.07E-29 | Inf | 9.34E-14 | 1371 |
| Cluster-40555.179814 | 0 | 0 | 0.59 | 0.69 | 0 | 0.2 | 0.73 | 1.01 | Inf | 1.51E-05 | 3.12 | 1.23E-03 | 3636 |
| Cluster-40555.179849 | 0 | 0 | 1.51 | 1.85 | 0.08 | 0 | 1.17 | 2.12 | Inf | 6.19E-03 | 5.72 | 1.41E-02 | 956 |
| Cluster-40555.179881 | 0 | 0 | 1.46 | 0.95 | 0 | 0 | 0.7 | 1.13 | Inf | 3.24E-05 | Inf | 2.22E-04 | 1977 |

| | | | | | | | | | | | | | |
|---|---|---|---|---|---|---|---|---|---|---|---|---|---|
| Cluster-40555.179927 | 0 | 0 | 3.28 | 2.11 | 0 | 0 | 2.87 | 2.56 | Inf | 1.82E-02 | Inf | 1.04E-02 | 637 |
| Cluster-40555.179936 | 0 | 0 | 7.83 | 3.92 | 2.48 | 2.62 | 6.96 | 5.67 | Inf | 1.56E-07 | 1.36 | 4.01E-02 | 1427 |
| Cluster-40555.180185 | 0 | 0 | 22.51 | 22.5 | 0 | 2.8 | 23.53 | 22.32 | Inf | 3.05E-62 | 4.05 | 1.43E-25 | 1632 |
| Cluster-40555.180302 | 0 | 0 | 3.29 | 3.38 | 3.6 | 3.41 | 6.84 | 7.09 | Inf | 6.85E-23 | 1.06 | 3.13E-02 | 2982 |
| Cluster-40555.180399 | 0 | 0 | 0.89 | 0.46 | 0 | 0 | 0.63 | 0.36 | Inf | 4.41E-03 | Inf | 1.50E-02 | 2160 |
| Cluster-40555.180495 | 0 | 0 | 1.83 | 2.73 | 0 | 0 | 5.74 | 6.17 | Inf | 1.16E-05 | Inf | 1.61E-15 | 1205 |
| Cluster-40555.180503 | 0 | 0 | 2.1 | 2.15 | 0 | 0 | 2.5 | 2.63 | Inf | 2.79E-03 | Inf | 2.77E-04 | 865 |
| Cluster-40555.180670 | 0 | 0 | 6.21 | 4.69 | 0.09 | 0 | 2.48 | 2.61 | Inf | 1.72E-08 | 6.15 | 1.19E-03 | 869 |
| Cluster-40555.180791 | 0 | 0 | 1.49 | 1.6 | 0 | 0.2 | 0.79 | 1.07 | Inf | 1.62E-08 | 3.35 | 8.79E-03 | 2377 |
| Cluster-40555.180819 | 0 | 0 | 2.54 | 1.64 | 0.44 | 0.99 | 3.85 | 3.32 | Inf | 8.07E-05 | 2.36 | 1.10E-02 | 1172 |
| Cluster-40555.180885 | 0 | 0 | 47.83 | 48.17 | 0 | 0 | 19.03 | 16.8 | Inf | 1.15E-137 | Inf | 1.83E-68 | 2855 |
| Cluster-40555.180926 | 0 | 0 | 5.45 | 4.86 | 0.41 | 0.32 | 7.45 | 5.12 | Inf | 3.74E-06 | 4.17 | 7.55E-05 | 728 |
| Cluster-40555.181018 | 0 | 0 | 30.6 | 24.6 | 0 | 0 | 3.45 | 7.84 | Inf | 8.51E-65 | Inf | 2.19E-04 | 1461 |
| Cluster-40555.181020 | 0 | 0 | 4.78 | 1.91 | 0 | 0 | 1.59 | 1.23 | Inf | 7.28E-05 | Inf | 3.24E-09 | 2556 |
| Cluster-40555.181022 | 0 | 0 | 1.77 | 1.45 | 0 | 0 | 1.77 | 0.98 | Inf | 1.08E-10 | Inf | 7.49E-08 | 2829 |
| Cluster-40555.181034 | 0 | 0 | 6.21 | 5.06 | 0 | 0.28 | 4.96 | 6.64 | Inf | 5.73E-26 | 5.35 | 2.69E-14 | 2136 |
| Cluster-40555.181049 | 0 | 0 | 2.15 | 2.56 | 1.33 | 0 | 2.6 | 2.65 | Inf | 6.86E-12 | 2.10 | 1.72E-02 | 2179 |
| Cluster-40555.181198 | 0 | 0 | 2.98 | 2.34 | 0 | 0 | 3.87 | 2.9 | Inf | 3.87E-03 | Inf | 2.02E-04 | 736 |
| Cluster-40555.181203 | 0 | 0 | 0.43 | 0.47 | 0 | 0 | 0.31 | 0.28 | Inf | 2.13E-03 | Inf | 2.04E-02 | 3247 |
| Cluster-40555.181313 | 0 | 0 | 3.03 | 2.42 | 0.3 | 0.07 | 2.51 | 2.09 | Inf | 3.07E-07 | 3.76 | 1.48E-03 | 1294 |
| Cluster-40555.181449 | 0 | 0 | 7.34 | 10.4 | 0 | 0 | 10.41 | 13.37 | Inf | 4.93E-03 | Inf | 4.58E-04 | 394 |
| Cluster-40555.181500 | 0 | 0 | 32.82 | 46.15 | 20.84 | 14.77 | 54.74 | 67.81 | Inf | 3.30E-09 | 1.83 | 2.09E-02 | 346 |
| Cluster-40555.181557 | 0 | 0 | 9.83 | 9.98 | 1.46 | 2.24 | 4.32 | 4.38 | Inf | 2.60E-43 | 1.29 | 3.87E-02 | 2186 |
| Cluster-40555.181661 | 0 | 0 | 7.51 | 7.93 | 0 | 0 | 11.27 | 8.77 | Inf | 5.07E-03 | Inf | 4.81E-04 | 416 |
| Cluster-40555.181995 | 0 | 0 | 8.68 | 12.17 | 0 | 0 | 7.32 | 7.36 | Inf | 4.36E-20 | Inf | 2.40E-22 | 1425 |
| Cluster-40555.182244 | 0 | 0 | 2.4 | 1.52 | 0.66 | 0.85 | 4.39 | 3.11 | Inf | 5.69E-09 | 2.38 | 1.04E-04 | 2058 |
| Cluster-40555.182392 | 0 | 0 | 7.67 | 5.99 | 0 | 0 | 10.94 | 9.9 | Inf | 2.74E-05 | Inf | 8.55E-09 | 566 |
| Cluster-40555.182443 | 0 | 0 | 53.39 | 51.16 | 0 | 0 | 13.72 | 13.77 | Inf | 1.38E-127 | Inf | 8.10E-52 | 2277 |
| Cluster-40555.182460 | 0 | 0 | 15.36 | 14.88 | 0.13 | 0 | 7.96 | 6.14 | Inf | 2.22E-30 | 7.07 | 5.43E-14 | 1088 |
| Cluster-40555.182491 | 0 | 0 | 14.81 | 7.87 | 0 | 0 | 2.78 | 2.78 | Inf | 1.86E-10 | Inf | 1.14E-09 | 1486 |
| Cluster-40555.182560 | 0 | 0 | 0.42 | 0.23 | 0 | 0 | 0.79 | 0.9 | Inf | 1.57E-03 | Inf | 9.61E-11 | 4689 |
| Cluster-40555.182776 | 0 | 0 | 19.94 | 20.09 | 0.11 | 0.11 | 12.19 | 10.97 | Inf | 1.80E-22 | 6.78 | 3.17E-12 | 706 |

| | | | | | | | | | | | | | |
|---|---|---|---|---|---|---|---|---|---|---|---|---|---|
| Cluster-40555.182787 | 0 | 0 | 2.41 | 3.2 | 0 | 0 | 1.63 | 1.04 | Inf | 5.01E-07 | Inf | 1.62E-03 | 1212 |
| Cluster-40555.182851 | 0 | 0 | 2.43 | 2.63 | 0.06 | 0.05 | 2.06 | 1.55 | Inf | 3.39E-08 | 5.05 | 1.27E-04 | 1507 |
| Cluster-40555.182980 | 0 | 0 | 12.84 | 11.75 | 0.29 | 0 | 5.8 | 9.88 | Inf | 4.97E-23 | 5.76 | 1.57E-05 | 1008 |
| Cluster-40555.182999 | 0 | 0 | 41.14 | 34.68 | 11.79 | 14.97 | 53.13 | 50.11 | Inf | 5.94E-27 | 2.00 | 9.86E-06 | 551 |
| Cluster-40555.183099 | 0 | 0 | 24.32 | 23.66 | 1.67 | 0.62 | 51.8 | 61.59 | Inf | 3.07E-29 | 5.71 | 2.26E-35 | 759 |
| Cluster-40555.183253 | 0 | 0 | 1.75 | 3.84 | 0.1 | 0 | 3.5 | 4.06 | Inf | 9.70E-04 | 6.59 | 4.00E-05 | 820 |
| Cluster-40555.183328 | 0 | 0 | 5.14 | 4.98 | 0.31 | 0.53 | 4.71 | 3.39 | Inf | 6.71E-07 | 3.33 | 6.92E-03 | 791 |
| Cluster-40555.183338 | 0 | 0 | 0.52 | 0.61 | 0 | 0 | 1.06 | 1.03 | Inf | 5.25E-03 | Inf | 3.12E-06 | 2360 |
| Cluster-40555.183341 | 0 | 0 | 4.02 | 2.5 | 0.62 | 0.99 | 8.73 | 5.18 | Inf | 2.59E-03 | 3.20 | 1.61E-03 | 673 |
| Cluster-40555.183506 | 0 | 0 | 10.23 | 12.11 | 0 | 0 | 4.9 | 4.01 | Inf | 3.22E-20 | Inf | 7.36E-09 | 966 |
| Cluster-40555.183566 | 0 | 0 | 6.89 | 6.41 | 0 | 0 | 7.11 | 4.42 | Inf | 2.37E-02 | Inf | 4.80E-02 | 403 |
| Cluster-40555.183598 | 0 | 0 | 1.58 | 2.38 | 0 | 0 | 2.17 | 2.42 | Inf | 6.72E-05 | Inf | 3.64E-06 | 1208 |
| Cluster-40555.183645 | 0 | 0 | 1.07 | 0.91 | 0 | 0 | 0.93 | 0.92 | Inf | 1.18E-05 | Inf | 7.36E-06 | 2503 |
| Cluster-40555.183713 | 0 | 0 | 12.04 | 10.21 | 0 | 0 | 4.99 | 4.56 | Inf | 6.91E-07 | Inf | 5.22E-03 | 501 |
| Cluster-40555.183720 | 0 | 0 | 9.94 | 9.88 | 0 | 0.14 | 8.07 | 7.91 | Inf | 7.03E-20 | 7.00 | 4.71E-15 | 1049 |
| Cluster-40555.183749 | 0 | 0 | 0.72 | 0.5 | 0 | 0 | 1.69 | 1.88 | Inf | 2.53E-02 | Inf | 4.39E-08 | 1846 |
| Cluster-40555.183779 | 0 | 0 | 3.45 | 4.29 | 0 | 0 | 4.39 | 3.63 | Inf | 3.49E-11 | Inf | 2.28E-12 | 1359 |
| Cluster-40555.183789 | 0 | 0 | 2.46 | 2.81 | 0.12 | 0 | 4.75 | 2.76 | Inf | 3.89E-03 | 6.30 | 4.14E-04 | 725 |
| Cluster-40555.183927 | 0 | 0 | 1.58 | 0.78 | 0 | 0 | 1.53 | 2.74 | Inf | 1.35E-05 | Inf | 1.39E-06 | 2562 |
| Cluster-40555.184153 | 0 | 0 | 2.04 | 4.32 | 0 | 0 | 0.92 | 0.91 | Inf | 1.25E-05 | Inf | 1.46E-05 | 2390 |
| Cluster-40555.184336 | 0 | 0 | 2.27 | 2.74 | 0.97 | 0.72 | 3.75 | 3.3 | Inf | 1.55E-18 | 2.13 | 4.16E-05 | 3125 |
| Cluster-40555.184362 | 0 | 0 | 2.81 | 2.01 | 0.2 | 0 | 2.11 | 1.92 | Inf | 4.36E-23 | 4.42 | 7.55E-12 | 4142 |
| Cluster-40555.184530 | 0 | 0 | 23.38 | 21.93 | 0.19 | 0 | 6.05 | 5.8 | Inf | 7.56E-18 | 6.35 | 2.85E-04 | 568 |
| Cluster-40555.184610 | 0 | 0 | 5.02 | 4.23 | 0 | 0 | 0.97 | 1.01 | Inf | 2.36E-18 | Inf | 2.32E-04 | 1825 |
| Cluster-40555.184705 | 0 | 0 | 1.17 | 5.12 | 0.26 | 0.25 | 9.79 | 6.34 | Inf | 2.08E-02 | 5.03 | 3.47E-12 | 2674 |
| Cluster-40555.184766 | 0 | 0 | 3.04 | 4.67 | 0.09 | 0.12 | 3.94 | 6.5 | Inf | 3.65E-11 | 5.76 | 3.23E-06 | 1461 |
| Cluster-40555.184906 | 0 | 0 | 18.25 | 12.36 | 0.08 | 0.04 | 5.53 | 6.27 | Inf | 9.96E-26 | 6.74 | 2.16E-16 | 1476 |
| Cluster-40555.184939 | 0.02 | 0 | 12.82 | 9.33 | 0.19 | 0 | 3.51 | 8.69 | Inf | 5.31E-26 | 6.10 | 1.38E-02 | 1224 |
| Cluster-40555.185032 | 0 | 0 | 1.05 | 0.89 | 0 | 0 | 1.02 | 1.2 | Inf | 2.43E-07 | Inf | 2.97E-09 | 3182 |
| Cluster-40555.185033 | 0 | 0 | 2.13 | 1.95 | 0.19 | 0 | 1.09 | 0.8 | Inf | 4.62E-12 | 3.46 | 6.36E-03 | 2536 |
| Cluster-40555.185036 | 0 | 0 | 6.17 | 4.21 | 0.6 | 0.18 | 3.46 | 6.07 | Inf | 1.46E-19 | 3.70 | 3.61E-03 | 1770 |
| Cluster-40555.185108 | 0 | 0 | 6.48 | 3.2 | 0 | 0 | 8.17 | 5.55 | Inf | 4.12E-06 | Inf | 5.07E-14 | 998 |

| | | | | | | | | | | | | | |
|---|---|---|---|---|---|---|---|---|---|---|---|---|---|
| Cluster-40555.185143 | 0 | 0 | 0.54 | 0.59 | 0 | 0 | 0.81 | 0.44 | Inf | 3.85E-02 | Inf | 1.27E-02 | 1804 |
| Cluster-40555.185296 | 0 | 0 | 4.66 | 2.4 | 0.29 | 0.87 | 2.7 | 2.96 | Inf | 1.62E-06 | 2.32 | 3.34E-02 | 1178 |
| Cluster-40555.185364 | 0 | 0 | 8.73 | 5.58 | 1.03 | 0.63 | 16.41 | 22.48 | Inf | 1.42E-03 | 4.79 | 5.35E-07 | 462 |
| Cluster-40555.185725 | 0 | 0 | 2.24 | 1.59 | 0.04 | 0.19 | 2.34 | 1.57 | Inf | 5.82E-07 | 4.05 | 1.24E-04 | 1695 |
| Cluster-40555.185828 | 0 | 0 | 102.64 | 115.54 | 7.61 | 5.14 | 109.39 | 82.65 | Inf | 9.73E-35 | 3.97 | 3.96E-14 | 393 |
| Cluster-40555.185857 | 0 | 0 | 5.01 | 6.89 | 0 | 0 | 6.69 | 7.65 | Inf | 5.62E-03 | Inf | 1.10E-03 | 457 |
| Cluster-40555.185862 | 0 | 0 | 3.79 | 4.05 | 0.32 | 0 | 7.72 | 3.81 | Inf | 5.97E-08 | 5.35 | 5.34E-04 | 1036 |
| Cluster-40555.185918 | 0 | 0 | 2.27 | 0.69 | 0 | 0 | 1.36 | 0.91 | Inf | 2.87E-02 | Inf | 2.27E-03 | 1346 |
| Cluster-40555.185944 | 0 | 0 | 4.06 | 5.27 | 1.74 | 0.51 | 3.18 | 3.01 | Inf | 1.92E-28 | 1.55 | 9.97E-03 | 3104 |
| Cluster-40555.185998 | 0 | 0 | 39.08 | 37.08 | 0 | 0 | 3.81 | 2.85 | Inf | 8.48E-27 | Inf | 1.74E-02 | 546 |
| Cluster-40555.186080 | 0 | 0 | 154.15 | 197.64 | 0 | 1.41 | 164.04 | 111 | Inf | 8.60E-26 | 8.12 | 3.08E-12 | 312 |
| Cluster-40555.186099 | 0 | 0 | 1.34 | 1.79 | 0 | 0 | 0.87 | 1.07 | Inf | 8.77E-10 | Inf | 1.53E-06 | 2631 |
| Cluster-40555.186124 | 0 | 0 | 4.55 | 4.34 | 0 | 0 | 1.58 | 3 | Inf | 6.77E-09 | Inf | 2.79E-04 | 1031 |
| Cluster-40555.186158 | 0 | 0 | 1.06 | 0.46 | 0 | 0 | 1.95 | 1.18 | Inf | 6.79E-04 | Inf | 6.58E-10 | 2476 |
| Cluster-40555.186216 | 0 | 0 | 24.33 | 21.19 | 0.41 | 0 | 17.14 | 12.96 | Inf | 9.42E-09 | 6.60 | 3.63E-05 | 409 |
| Cluster-40555.186334 | 0 | 0 | 3.6 | 3.6 | 0 | 0.17 | 4.3 | 4.83 | Inf | 3.28E-14 | 5.85 | 9.67E-15 | 1789 |
| Cluster-40555.186374 | 0 | 0 | 45.1 | 40.41 | 1.24 | 2.39 | 44.93 | 34.31 | Inf | 2.89E-91 | 4.48 | 2.28E-32 | 1569 |
| Cluster-40555.186572 | 0 | 0 | 13.48 | 13.34 | 0 | 0 | 10.12 | 4.11 | Inf | 1.55E-24 | Inf | 2.67E-04 | 993 |
| Cluster-40555.186573 | 0 | 0 | 5.26 | 5.79 | 0.06 | 0 | 3.85 | 5.54 | Inf | 6.91E-11 | 7.36 | 4.88E-09 | 1015 |
| Cluster-40555.186814 | 0 | 0 | 33.96 | 31.2 | 0.75 | 0 | 12.42 | 16.39 | Inf | 2.58E-11 | 5.36 | 8.14E-04 | 394 |
| Cluster-40555.186851 | 0 | 0 | 3.33 | 6.62 | 1.68 | 1.86 | 4.84 | 4.73 | Inf | 3.53E-07 | 1.50 | 5.79E-03 | 2409 |
| Cluster-40555.187233 | 0 | 0 | 60.58 | 64.44 | 0 | 0 | 14.87 | 23.86 | Inf | 1.21E-92 | Inf | 2.12E-12 | 1168 |
| Cluster-40555.187290 | 0 | 0 | 74.42 | 77.52 | 1.9 | 1.96 | 21.23 | 27.87 | Inf | 9.01E-119 | 3.75 | 8.36E-12 | 1447 |
| Cluster-40555.187478 | 0 | 0 | 0.26 | 0.67 | 0 | 0 | 2.59 | 1.15 | Inf | 8.88E-03 | Inf | 5.20E-05 | 2810 |
| Cluster-40555.187507 | 0 | 0 | 3.25 | 2.39 | 0.19 | 0.39 | 2.2 | 2.45 | Inf | 6.00E-11 | 3.07 | 4.68E-04 | 1771 |
| Cluster-40555.187712 | 0 | 0 | 10.81 | 2.5 | 0.59 | 1.18 | 6.26 | 3.83 | Inf | 2.30E-02 | 2.56 | 9.25E-04 | 1221 |
| Cluster-40555.187730 | 0 | 0 | 0.7 | 0.23 | 0 | 0 | 0.77 | 0.69 | Inf | 1.76E-02 | Inf | 2.92E-07 | 3729 |
| Cluster-40555.187733 | 0 | 0 | 1.74 | 1.38 | 0 | 0 | 2.75 | 2.56 | Inf | 6.45E-07 | Inf | 6.36E-13 | 1994 |
| Cluster-40555.187849 | 0 | 0 | 9.04 | 7.34 | 0 | 1.04 | 5.3 | 6.41 | Inf | 1.06E-25 | 3.51 | 4.95E-09 | 1536 |
| Cluster-40555.187862 | 0 | 0 | 0.23 | 0.41 | 0.03 | 0.06 | 0.92 | 1 | Inf | 1.91E-02 | 4.38 | 3.30E-05 | 3275 |
| Cluster-40555.188024 | 0 | 0 | 0.76 | 0.22 | 0 | 0 | 1.45 | 0.88 | Inf | 4.81E-02 | Inf | 1.50E-09 | 3151 |
| Cluster-40555.188047 | 0 | 0 | 649.36 | 770.64 | 0 | 0 | 812.85 | 964.09 | Inf | 4.40E-112 | Inf | 2.84E-109 | 1123 |

| | | | | | | | | | | | | | |
|---|---|---|---|---|---|---|---|---|---|---|---|---|---|
| Cluster-40555.188048 | 0 | 0 | 686.91 | 677.81 | 4.11 | 4.2 | 58.12 | 153.76 | Inf | 5.24E-58 | 4.66 | 4.96E-02 | 290 |
| Cluster-40555.188051 | 0 | 0 | 111.96 | 96.91 | 1.24 | 2.58 | 34.09 | 61.33 | Inf | 3.63E-142 | 4.71 | 2.55E-04 | 1561 |
| Cluster-40555.188069 | 0 | 0 | 1.81 | 1.58 | 0 | 0 | 1.52 | 1.06 | Inf | 5.77E-12 | Inf | 5.44E-10 | 2989 |
| Cluster-40555.188118 | 0 | 0 | 3.14 | 6.03 | 0 | 0 | 2.56 | 1.52 | Inf | 6.84E-07 | Inf | 2.52E-08 | 1698 |
| Cluster-40555.188436 | 0 | 0 | 19.17 | 20.45 | 0 | 0 | 11.94 | 10.29 | Inf | 8.14E-57 | Inf | 5.88E-35 | 1629 |
| Cluster-40555.188580 | 0 | 0 | 0.27 | 0.61 | 0 | 0 | 0.38 | 0.57 | Inf | 4.03E-02 | Inf | 1.97E-02 | 2177 |
| Cluster-40555.188854 | 0 | 0 | 1.25 | 2.09 | 0 | 0 | 2.59 | 2.04 | Inf | 3.43E-03 | Inf | 1.00E-04 | 993 |
| Cluster-40555.188948 | 0 | 0 | 2.23 | 3.77 | 0 | 0 | 3.15 | 3.11 | Inf | 1.95E-06 | Inf | 4.05E-07 | 1072 |
| Cluster-40555.188958 | 0 | 0 | 6.3 | 6.17 | 0 | 0 | 4.04 | 4.54 | Inf | 2.18E-09 | Inf | 5.58E-07 | 843 |
| Cluster-40555.188981 | 0 | 0 | 29.79 | 31.88 | 0 | 0.3 | 12.8 | 12.32 | Inf | 6.41E-14 | 6.39 | 4.79E-05 | 434 |
| Cluster-40555.189024 | 0 | 0 | 83.03 | 70.74 | 2.07 | 0.6 | 47.1 | 40.82 | Inf | 5.10E-58 | 5.15 | 4.25E-23 | 628 |
| Cluster-40555.189174 | 0 | 0 | 1.96 | 3.09 | 0 | 0 | 20.87 | 13.22 | Inf | 4.47E-03 | Inf | 1.60E-14 | 738 |
| Cluster-40555.189176 | 0 | 0 | 4.14 | 5.33 | 0 | 0 | 3.6 | 2.3 | Inf | 1.46E-30 | Inf | 1.04E-16 | 3270 |
| Cluster-40555.189193 | 0 | 0 | 13.65 | 15.04 | 0 | 0 | 3.71 | 8.69 | Inf | 2.00E-07 | Inf | 1.19E-02 | 459 |
| Cluster-40555.189233 | 0 | 0 | 1.85 | 2.23 | 0.15 | 0.17 | 3.44 | 1.96 | Inf | 2.13E-08 | 4.05 | 7.99E-05 | 1826 |
| Cluster-40555.189234 | 0 | 0 | 0.94 | 1.07 | 0 | 0 | 1.08 | 0.67 | Inf | 1.36E-12 | Inf | 2.25E-11 | 5040 |
| Cluster-40555.189238 | 0 | 0 | 21.78 | 26.58 | 0 | 0 | 5.28 | 6.19 | Inf | 4.66E-12 | Inf | 8.59E-03 | 450 |
| Cluster-40555.189248 | 0 | 0 | 92.45 | 82.61 | 2.7 | 3.35 | 43.9 | 53.06 | Inf | 2.98E-21 | 4.03 | 1.83E-06 | 355 |
| Cluster-40555.189398 | 0 | 0 | 1.88 | 2.8 | 0 | 0 | 3.31 | 3.42 | Inf | 2.42E-04 | Inf | 9.41E-07 | 975 |
| Cluster-40555.189481 | 0 | 0 | 14.78 | 16.8 | 0 | 0 | 8.55 | 7.78 | Inf | 3.40E-06 | Inf | 3.71E-03 | 411 |
| Cluster-40555.189847 | 0 | 0 | 4.55 | 4.71 | 0 | 0 | 2.48 | 1.14 | Inf | 1.19E-32 | Inf | 1.46E-05 | 3190 |
| Cluster-40555.189940 | 0 | 0 | 6.46 | 7.64 | 0.38 | 0.56 | 3.97 | 4.71 | Inf | 1.88E-07 | 3.21 | 2.72E-02 | 666 |
| Cluster-40555.189985 | 0 | 0 | 13.29 | 15.28 | 1.63 | 1.67 | 11.82 | 12.25 | Inf | 7.55E-12 | 2.94 | 6.56E-04 | 577 |
| Cluster-40555.190005 | 0 | 0 | 12.28 | 16.83 | 0 | 0 | 4.28 | 2.89 | Inf | 3.64E-19 | Inf | 4.31E-05 | 768 |
| Cluster-40555.190054 | 0 | 0 | 1.02 | 0.73 | 0.93 | 1.06 | 4.3 | 4.57 | Inf | 2.05E-03 | 2.23 | 2.87E-04 | 1814 |
| Cluster-40555.190092 | 0 | 0 | 3.75 | 4.4 | 0 | 0 | 4.8 | 4.39 | Inf | 1.83E-25 | Inf | 2.38E-28 | 2748 |
| Cluster-40555.190256 | 0 | 0 | 42.21 | 12.76 | 21.17 | 11.65 | 56.76 | 45.09 | Inf | 6.21E-04 | 1.71 | 3.32E-06 | 993 |
| Cluster-40555.190316 | 0 | 0 | 6.64 | 6.1 | 0 | 0 | 5.38 | 6.01 | Inf | 4.51E-31 | Inf | 1.77E-28 | 2282 |
| Cluster-40555.190917 | 0 | 0 | 12.46 | 12.1 | 0.6 | 0 | 26.88 | 26.38 | Inf | 1.15E-34 | 6.61 | 4.69E-43 | 1449 |
| Cluster-40555.191010 | 0 | 0 | 191.04 | 204.23 | 70.52 | 35.59 | 385.17 | 401.33 | Inf | 1.86E-162 | 2.97 | 2.03E-23 | 1211 |
| Cluster-40555.191073 | 0 | 0 | 24.74 | 17.5 | 2.39 | 0.6 | 54.59 | 34.51 | Inf | 5.60E-06 | 5.10 | 2.72E-07 | 372 |
| Cluster-40555.191353 | 0 | 0 | 0.82 | 0.86 | 0 | 0 | 0.23 | 0.35 | Inf | 3.17E-06 | Inf | 3.28E-02 | 3102 |

| | | | | | | | | | | | | | |
|---|---|---|---|---|---|---|---|---|---|---|---|---|---|
| Cluster-40555.191402 | 0 | 0 | 16.08 | 12.75 | 0 | 0 | 2.5 | 4.47 | Inf | 1.58E-12 | Inf | 4.18E-03 | 599 |
| Cluster-40555.191531 | 0 | 0 | 4.89 | 5.02 | 0 | 0 | 1.59 | 1.7 | Inf | 3.11E-17 | Inf | 3.08E-06 | 1612 |
| Cluster-40555.191618 | 0 | 0 | 6.64 | 6.7 | 0 | 0.54 | 12.27 | 11.86 | Inf | 1.17E-13 | 5.43 | 1.33E-17 | 1049 |
| Cluster-40555.191770 | 0 | 0 | 50.76 | 33.53 | 11.19 | 3.63 | 50.87 | 61.32 | Inf | 2.08E-23 | 3.01 | 1.47E-13 | 739 |
| Cluster-40555.191785 | 0 | 0 | 12.01 | 12.49 | 0 | 0 | 7.72 | 10.35 | Inf | 8.69E-15 | Inf | 1.15E-11 | 726 |
| Cluster-40555.191840 | 0 | 0 | 0.71 | 1.21 | 0 | 0 | 0.55 | 0.63 | Inf | 3.00E-05 | Inf | 1.82E-03 | 2344 |
| Cluster-40555.192031 | 0 | 0 | 32.49 | 47.33 | 0 | 0 | 19.43 | 7.85 | Inf | 1.07E-21 | Inf | 4.93E-05 | 1064 |
| Cluster-40555.192072 | 0 | 0 | 3.08 | 3.05 | 0 | 0 | 4.32 | 5.14 | Inf | 3.04E-04 | Inf | 3.31E-07 | 807 |
| Cluster-40555.192123 | 0 | 0 | 8.23 | 6.84 | 0 | 0 | 3.5 | 6.35 | Inf | 5.76E-04 | Inf | 1.18E-02 | 470 |
| Cluster-40555.192191 | 0 | 0 | 3.89 | 4.82 | 0.27 | 0.33 | 8.71 | 6.89 | Inf | 5.48E-12 | 4.76 | 1.43E-13 | 1307 |
| Cluster-40555.192230 | 0 | 0 | 2.2 | 1.15 | 0 | 0 | 1.45 | 0.74 | Inf | 6.18E-07 | Inf | 5.85E-05 | 2041 |
| Cluster-40555.192260 | 0 | 0 | 1.15 | 0.71 | 0 | 0 | 0.44 | 0.86 | Inf | 3.50E-03 | Inf | 1.95E-02 | 1653 |
| Cluster-40555.192262 | 0 | 0 | 6.68 | 6.6 | 0 | 0 | 5.72 | 4.41 | Inf | 7.70E-30 | Inf | 4.31E-24 | 2104 |
| Cluster-40555.192268 | 0 | 0 | 5.85 | 8.35 | 0.07 | 0.21 | 3.95 | 4.59 | Inf | 1.86E-15 | 4.99 | 1.74E-07 | 1150 |
| Cluster-40555.192333 | 0 | 0 | 11.02 | 7.96 | 0 | 0 | 7.97 | 11.66 | Inf | 3.35E-04 | Inf | 1.33E-04 | 437 |
| Cluster-40555.192436 | 0 | 0 | 1.75 | 1.14 | 0 | 0 | 1.09 | 2.94 | Inf | 1.76E-05 | Inf | 1.07E-02 | 1760 |
| Cluster-40555.192575 | 0 | 0 | 1.26 | 1.41 | 0.36 | 0.19 | 1.65 | 1.06 | Inf | 6.06E-11 | 2.39 | 2.86E-03 | 3390 |
| Cluster-40555.192632 | 0 | 0 | 0.68 | 0.6 | 0 | 0 | 0.72 | 0.36 | Inf | 9.78E-03 | Inf | 1.51E-02 | 1990 |
| Cluster-40555.192692 | 0 | 0 | 1.24 | 0.98 | 1.02 | 0 | 2.86 | 3.21 | Inf | 1.95E-04 | 2.68 | 4.77E-04 | 1848 |
| Cluster-40555.192883 | 0 | 0 | 1.99 | 2.38 | 0 | 0 | 2.69 | 2.45 | Inf | 7.92E-21 | Inf | 1.35E-24 | 3978 |
| Cluster-40555.192927 | 0 | 0 | 4.5 | 5.22 | 0 | 0 | 2.55 | 1.88 | Inf | 6.07E-13 | Inf | 2.31E-06 | 1279 |
| Cluster-40555.193280 | 0 | 0 | 3.53 | 1.67 | 1.59 | 0.46 | 4 | 4.86 | Inf | 9.81E-06 | 2.23 | 7.68E-04 | 1681 |
| Cluster-40555.193342 | 0 | 0 | 2.93 | 1.8 | 0.61 | 0.67 | 2.47 | 2.32 | Inf | 1.92E-11 | 1.97 | 1.17E-02 | 2165 |
| Cluster-40555.193452 | 0 | 0 | 17.21 | 33.35 | 9.5 | 5.78 | 24.49 | 31.75 | Inf | 3.47E-08 | 1.96 | 4.23E-05 | 886 |
| Cluster-40555.193455 | 0 | 0 | 1.15 | 1.94 | 0.69 | 0.46 | 2.43 | 2.75 | Inf | 1.09E-05 | 2.28 | 1.10E-02 | 1648 |
| Cluster-40555.193614 | 0 | 0 | 10.15 | 12.49 | 0 | 0 | 31.12 | 28.78 | Inf | 5.67E-22 | Inf | 1.08E-45 | 1022 |
| Cluster-40555.193646 | 0 | 0 | 0.91 | 1.49 | 0 | 0 | 1.29 | 1.15 | Inf | 1.06E-02 | Inf | 7.38E-03 | 1135 |
| Cluster-40555.193654 | 0 | 0 | 22.47 | 18.33 | 0.12 | 0.49 | 25.09 | 19.69 | Inf | 8.90E-21 | 6.22 | 1.22E-18 | 666 |
| Cluster-40555.193655 | 0 | 0 | 9.59 | 12.62 | 0.38 | 0 | 9.03 | 9.52 | Inf | 3.74E-11 | 5.74 | 1.27E-07 | 642 |
| Cluster-40555.193656 | 0 | 0 | 5.15 | 3.88 | 0 | 0 | 4.73 | 4.54 | Inf | 1.16E-21 | Inf | 2.86E-23 | 2187 |
| Cluster-40555.193661 | 0 | 0 | 1.61 | 2.48 | 0.12 | 0 | 4.3 | 3.22 | Inf | 1.39E-10 | 6.08 | 1.95E-15 | 2205 |
| Cluster-40555.193812 | 0 | 0 | 17.34 | 18.52 | 0.12 | 0 | 10.61 | 9.88 | Inf | 4.19E-62 | 7.35 | 2.69E-33 | 1971 |

| | | | | | | | | | | | | | |
|---|---|---|---|---|---|---|---|---|---|---|---|---|---|
| Cluster-40555.193832 | 0 | 0 | 0.2 | 0.6 | 0 | 0 | 2.72 | 5.91 | Inf | 2.99E-02 | Inf | 2.39E-05 | 2966 |
| Cluster-40555.193846 | 0 | 0 | 1.23 | 0.84 | 0.08 | 0.14 | 1.06 | 1.24 | Inf | 3.61E-03 | 3.36 | 4.59E-02 | 1506 |
| Cluster-40555.193889 | 0 | 0 | 5.94 | 7.37 | 0 | 0.16 | 6.27 | 4.84 | Inf | 1.03E-39 | 6.13 | 1.99E-27 | 3014 |
| Cluster-40555.193965 | 0 | 0 | 1.81 | 1.37 | 0 | 0 | 1.76 | 1.77 | Inf | 1.57E-03 | Inf | 2.33E-04 | 1147 |
| Cluster-40555.193995 | 0 | 0 | 1.82 | 3.04 | 0 | 0 | 1.88 | 0.9 | Inf | 1.76E-07 | Inf | 4.48E-04 | 1495 |
| Cluster-40555.194130 | 0 | 0 | 0.98 | 1.28 | 0 | 0 | 0.78 | 0.87 | Inf | 4.17E-03 | Inf | 2.01E-02 | 1362 |
| Cluster-40555.194383 | 0 | 0 | 4.17 | 3.14 | 0 | 0 | 2.69 | 1.52 | Inf | 1.02E-07 | Inf | 7.38E-05 | 1086 |
| Cluster-40555.194531 | 0 | 0 | 3.99 | 3.77 | 0.31 | 0.85 | 2.52 | 2.69 | Inf | 1.77E-10 | 2.21 | 3.82E-02 | 1301 |
| Cluster-40555.194603 | 0 | 0 | 17.41 | 12.67 | 1.25 | 1.06 | 6.08 | 3.69 | Inf | 3.76E-40 | 2.13 | 3.11E-03 | 1922 |
| Cluster-40555.194629 | 0 | 0 | 0.71 | 0.91 | 0 | 0 | 0.46 | 0.51 | Inf | 1.85E-05 | Inf | 2.18E-03 | 2823 |
| Cluster-40555.194663 | 0 | 0 | 11.99 | 9.48 | 0.32 | 0 | 5.06 | 2.84 | Inf | 4.66E-38 | 4.67 | 2.62E-05 | 1798 |
| Cluster-40555.194677 | 0 | 0 | 4.99 | 5.11 | 0.68 | 0.45 | 6.07 | 4.19 | Inf | 9.08E-18 | 3.28 | 1.51E-07 | 1631 |
| Cluster-40555.194754 | 0 | 0 | 3.81 | 3.91 | 0.11 | 0 | 2.91 | 3.1 | Inf | 3.97E-14 | 6.03 | 1.58E-09 | 1669 |
| Cluster-40555.194794 | 0 | 0 | 5.51 | 6.91 | 0 | 0 | 3.23 | 2.31 | Inf | 4.51E-10 | Inf | 1.01E-04 | 876 |
| Cluster-40555.194833 | 0 | 0 | 10.63 | 11.92 | 0 | 0 | 9.76 | 9.3 | Inf | 1.41E-28 | Inf | 5.70E-25 | 1287 |
| Cluster-40555.194922 | 0 | 0 | 11.35 | 16.34 | 0.37 | 0 | 10.63 | 7.6 | Inf | 1.22E-18 | 5.82 | 7.09E-17 | 1205 |
| Cluster-40555.194976 | 0 | 0 | 16.37 | 20.96 | 0 | 0 | 12.98 | 14.56 | Inf | 6.15E-31 | Inf | 4.19E-24 | 945 |
| Cluster-40555.195095 | 0 | 0 | 4.48 | 4.38 | 0 | 0 | 4.42 | 7.38 | Inf | 2.79E-07 | Inf | 2.54E-07 | 894 |
| Cluster-40555.195120 | 0 | 0 | 3.72 | 2.43 | 0.27 | 0 | 1.25 | 1.22 | Inf | 4.54E-16 | 3.32 | 2.91E-03 | 2341 |
| Cluster-40555.195175 | 0 | 0 | 1.35 | 0.75 | 0.34 | 0.53 | 2.7 | 2.22 | Inf | 5.40E-06 | 2.51 | 2.01E-04 | 2518 |
| Cluster-40555.195261 | 0 | 0 | 0.65 | 1.1 | 0.08 | 0.2 | 1.52 | 1.07 | Inf | 3.43E-03 | 3.26 | 1.80E-02 | 1658 |
| Cluster-40555.195465 | 0 | 0 | 2.02 | 1.28 | 0.08 | 0 | 0.7 | 1.54 | Inf | 2.32E-09 | 4.88 | 2.59E-02 | 2493 |
| Cluster-40555.195515 | 0 | 0 | 2.98 | 3.23 | 0.3 | 0.15 | 2.55 | 3.32 | Inf | 8.17E-15 | 3.75 | 2.82E-07 | 2092 |
| Cluster-40555.195542 | 0 | 0 | 37.28 | 37.66 | 13.16 | 11.41 | 34.85 | 35.75 | Inf | 3.06E-52 | 1.59 | 1.07E-04 | 909 |
| Cluster-40555.195586 | 0 | 0 | 6.9 | 7.58 | 0.09 | 0 | 2.13 | 2.98 | Inf | 7.39E-33 | 5.81 | 2.80E-10 | 2140 |
| Cluster-40555.195598 | 0 | 0 | 1.16 | 1.81 | 0 | 1.32 | 2.52 | 2.57 | Inf | 1.82E-06 | 1.97 | 2.78E-02 | 1906 |
| Cluster-40555.195617 | 0 | 0 | 4.33 | 5.13 | 0 | 0 | 1.09 | 1.05 | Inf | 2.76E-17 | Inf | 3.30E-04 | 1669 |
| Cluster-40555.195644 | 0 | 0 | 3.98 | 3.53 | 0.13 | 0.98 | 3.73 | 2.36 | Inf | 1.23E-15 | 2.47 | 3.11E-03 | 1880 |
| Cluster-40555.195892 | 0 | 0 | 33.61 | 40.44 | 0 | 0 | 28.5 | 31.39 | Inf | 3.41E-14 | Inf | 1.67E-11 | 407 |
| Cluster-40555.195960 | 0 | 0 | 129.27 | 103.87 | 0 | 0.59 | 74.23 | 95.59 | Inf | 6.40E-135 | 8.21 | 1.08E-35 | 2110 |
| Cluster-40555.196226 | 0 | 0 | 7.32 | 4.87 | 0.39 | 0.2 | 6.13 | 3.7 | Inf | 2.68E-18 | 4.16 | 1.71E-06 | 1488 |
| Cluster-40555.196242 | 0 | 0 | 4.28 | 10.62 | 0.41 | 0.1 | 3.51 | 5.07 | Inf | 6.79E-05 | 4.13 | 4.20E-07 | 1783 |

| | | | | | | | | | | | | | |
|---|---|---|---|---|---|---|---|---|---|---|---|---|---|
| Cluster-40555.196245 | 0 | 0 | 2.69 | 2.88 | 0 | 0.68 | 3.97 | 2.29 | Inf | 5.75E-09 | 3.20 | 1.61E-03 | 1490 |
| Cluster-40555.196355 | 0 | 0 | 21.6 | 31.26 | 0.28 | 0 | 40.27 | 29.06 | Inf | 1.46E-12 | 8.08 | 5.00E-15 | 440 |
| Cluster-40555.196440 | 0 | 0 | 0.67 | 0.41 | 0 | 0 | 1.76 | 3.24 | Inf | 9.27E-06 | Inf | 2.09E-07 | 4488 |
| Cluster-40555.196528 | 0 | 0 | 4.97 | 5.22 | 0.35 | 0 | 2.8 | 4.36 | Inf | 2.49E-05 | 4.46 | 1.29E-02 | 664 |
| Cluster-40555.196587 | 0 | 0 | 1.85 | 2.01 | 0.85 | 0.94 | 3.08 | 2.54 | Inf | 9.58E-08 | 1.70 | 4.27E-02 | 1806 |
| Cluster-40555.196608 | 0 | 0 | 20.6 | 22.23 | 0 | 0.56 | 11.67 | 13.25 | Inf | 8.68E-38 | 5.41 | 2.72E-17 | 1012 |
| Cluster-40555.196687 | 0 | 0 | 5.17 | 7.72 | 0.11 | 0 | 4.42 | 4.97 | Inf | 4.11E-07 | 6.48 | 1.06E-04 | 678 |
| Cluster-40555.196693 | 0 | 0 | 0.82 | 0.61 | 0 | 0 | 0.82 | 0.9 | Inf | 8.96E-05 | Inf | 1.84E-06 | 2881 |
| Cluster-40555.196772 | 0 | 0 | 3.61 | 4.7 | 0 | 0.11 | 3.34 | 2.75 | Inf | 3.59E-10 | 5.85 | 1.63E-06 | 1191 |
| Cluster-40555.196836 | 0 | 0 | 1.06 | 0.49 | 0 | 0 | 1.57 | 0.96 | Inf | 7.74E-03 | Inf | 2.22E-05 | 1771 |
| Cluster-40555.196875 | 0 | 0 | 1.38 | 1.05 | 0.11 | 0 | 1.33 | 2.38 | Inf | 1.95E-02 | 5.17 | 3.04E-03 | 1085 |
| Cluster-40555.196964 | 0 | 0 | 1.72 | 2.09 | 0 | 0 | 1.35 | 2.31 | Inf | 1.10E-03 | Inf | 7.45E-04 | 1020 |
| Cluster-40555.196966 | 0 | 0 | 9.82 | 5.39 | 0 | 0 | 0.59 | 1.53 | Inf | 1.21E-10 | Inf | 2.58E-02 | 1657 |
| Cluster-40555.197320 | 0 | 0 | 8.91 | 8.59 | 0 | 0 | 6.4 | 4.62 | Inf | 3.82E-15 | Inf | 2.41E-10 | 930 |
| Cluster-40555.197331 | 0 | 0 | 12.94 | 10.89 | 0 | 0.2 | 10.61 | 10.82 | Inf | 9.84E-08 | 6.73 | 1.62E-06 | 513 |
| Cluster-40555.197344 | 0 | 0 | 5.11 | 5 | 0.41 | 0.27 | 8.68 | 8.46 | Inf | 6.81E-12 | 4.72 | 8.61E-13 | 1166 |
| Cluster-40555.197440 | 0 | 0 | 4.02 | 1.75 | 0 | 0 | 3.97 | 6.5 | Inf | 3.94E-05 | Inf | 1.40E-09 | 1891 |
| Cluster-40555.197487 | 0 | 0 | 2.19 | 0.87 | 0 | 0 | 1.72 | 1.78 | Inf | 3.84E-02 | Inf | 8.65E-03 | 853 |
| Cluster-40555.197526 | 0 | 0 | 13.25 | 16.32 | 0.8 | 0.25 | 9.73 | 12.29 | Inf | 2.22E-11 | 4.57 | 1.16E-05 | 554 |
| Cluster-40555.197644 | 0 | 0 | 10.32 | 12.15 | 0 | 0 | 7.75 | 7.23 | Inf | 1.60E-26 | Inf | 4.91E-19 | 1206 |
| Cluster-40555.197741 | 0 | 0 | 22.85 | 26.15 | 1.96 | 1.35 | 21.44 | 18.32 | Inf | 4.14E-05 | 3.63 | 3.60E-02 | 338 |
| Cluster-40555.197760 | 0 | 0 | 6.55 | 5.47 | 0.77 | 0 | 2.29 | 4.3 | Inf | 2.38E-14 | 3.25 | 4.84E-02 | 1192 |
| Cluster-40555.197906 | 0 | 0 | 5.6 | 6.2 | 1.13 | 0.36 | 6.95 | 6.19 | Inf | 6.46E-13 | 3.24 | 4.90E-06 | 1099 |
| Cluster-40555.197907 | 0 | 0 | 2.79 | 1.51 | 0 | 0 | 2.57 | 2.39 | Inf | 1.14E-05 | Inf | 1.75E-07 | 1317 |
| Cluster-40555.197918 | 0 | 0 | 0.74 | 0.5 | 0 | 0 | 0.89 | 0.94 | Inf | 2.87E-02 | Inf | 6.57E-04 | 1783 |
| Cluster-40555.198001 | 0 | 0 | 4.61 | 3.82 | 0.05 | 0.11 | 2.17 | 1.99 | Inf | 2.26E-11 | 4.95 | 1.91E-04 | 1312 |
| Cluster-40555.198093 | 0 | 0 | 1.8 | 2.34 | 0 | 0 | 0.82 | 1.23 | Inf | 7.84E-14 | Inf | 1.62E-07 | 2814 |
| Cluster-40555.198118 | 0 | 0 | 6.39 | 5.44 | 0.12 | 0.27 | 4.03 | 3.81 | Inf | 1.01E-23 | 4.38 | 3.28E-10 | 1877 |
| Cluster-40555.198175 | 0 | 0 | 5.39 | 2.63 | 0.86 | 0 | 2.29 | 3.01 | Inf | 5.03E-07 | 2.73 | 8.90E-04 | 1880 |
| Cluster-40555.198260 | 0 | 0 | 5.06 | 4.12 | 0 | 0.88 | 3.02 | 5.98 | Inf | 1.02E-24 | 3.39 | 3.60E-02 | 2439 |
| Cluster-40555.198291 | 0 | 0 | 7.39 | 4.31 | 0 | 1.77 | 4.42 | 4.64 | Inf | 1.32E-10 | 2.38 | 3.64E-03 | 1104 |
| Cluster-40555.198385 | 0 | 0 | 21.2 | 23.6 | 0.36 | 2.49 | 10.23 | 13.48 | Inf | 2.92E-45 | 3.09 | 4.45E-07 | 1156 |

| | | | | | | | | | | | | | |
|---|---|---|---|---|---|---|---|---|---|---|---|---|---|
| Cluster-40555.198450 | 0 | 0 | 8.97 | 8.1 | 0.06 | 0 | 10.24 | 9.55 | Inf | 6.33E-42 | 8.37 | 2.00E-40 | 2424 |
| Cluster-40555.198497 | 0 | 0 | 2.17 | 2.67 | 1.55 | 0.93 | 4.19 | 5.96 | Inf | 8.19E-19 | 2.12 | 1.92E-03 | 3270 |
| Cluster-40555.198602 | 0 | 0 | 1 | 0.73 | 0.44 | 0.12 | 1.57 | 1.72 | Inf | 4.10E-05 | 2.66 | 1.69E-03 | 2619 |
| Cluster-40555.198609 | 0 | 0 | 3.23 | 1.85 | 0.18 | 0.18 | 3.12 | 3.54 | Inf | 1.86E-03 | 4.31 | 2.77E-03 | 817 |
| Cluster-40555.198651 | 0 | 0 | 2.63 | 1.58 | 0.14 | 0 | 2.18 | 2.8 | Inf | 1.06E-04 | 5.11 | 1.39E-04 | 1160 |
| Cluster-40555.198700 | 0 | 0 | 0.24 | 0.37 | 0 | 0 | 0.47 | 0.48 | Inf | 1.04E-04 | Inf | 5.29E-08 | 6113 |
| Cluster-40555.198772 | 0 | 0 | 6.89 | 10.34 | 0.46 | 0.32 | 4.38 | 6.38 | Inf | 3.14E-07 | 3.89 | 1.03E-02 | 578 |
| Cluster-40555.198880 | 0 | 0 | 49.59 | 46.68 | 0.21 | 0 | 21.83 | 15.78 | Inf | 1.67E-40 | 7.28 | 4.00E-16 | 627 |
| Cluster-40555.198965 | 0 | 0 | 1.95 | 0.93 | 0 | 0 | 2.13 | 0.93 | Inf | 5.61E-05 | Inf | 4.25E-04 | 1854 |
| Cluster-40555.199338 | 0 | 0 | 28.98 | 33.56 | 0.55 | 0 | 31.63 | 27.16 | Inf | 8.48E-47 | 6.81 | 9.12E-34 | 929 |
| Cluster-40555.199401 | 0 | 0 | 0.77 | 1.55 | 0.14 | 0 | 1.16 | 2.36 | Inf | 7.69E-04 | 4.69 | 1.25E-02 | 1521 |
| Cluster-40555.199455 | 0 | 0 | 33.82 | 31.53 | 0 | 0 | 22.39 | 14.26 | Inf | 1.71E-86 | Inf | 6.93E-22 | 1822 |
| Cluster-40555.199461 | 0 | 0 | 4.83 | 2.55 | 0 | 0 | 2.17 | 2.36 | Inf | 2.01E-07 | Inf | 1.71E-07 | 1421 |
| Cluster-40555.199572 | 0 | 0 | 0.94 | 1.19 | 0 | 0 | 1.36 | 1.03 | Inf | 2.06E-06 | Inf | 7.86E-08 | 2575 |
| Cluster-40555.199644 | 0 | 0 | 2.18 | 1.38 | 0 | 0 | 3.39 | 2.51 | Inf | 2.02E-05 | Inf | 3.32E-10 | 1478 |
| Cluster-40555.199646 | 0 | 0 | 3.77 | 6.79 | 0 | 0.22 | 1.84 | 2.15 | Inf | 3.92E-08 | 4.11 | 2.63E-04 | 1515 |
| Cluster-40555.199688 | 0 | 0 | 7.38 | 5.8 | 0 | 0.86 | 7.1 | 8.55 | Inf | 1.59E-29 | 4.19 | 6.62E-18 | 2121 |
| Cluster-40555.199963 | 0 | 0 | 5.32 | 4.77 | 0 | 0 | 0.43 | 0.61 | Inf | 5.34E-19 | Inf | 3.89E-02 | 1746 |
| Cluster-40555.199986 | 0 | 0 | 2.75 | 2.1 | 0.08 | 0.12 | 3.64 | 3.15 | Inf | 6.37E-14 | 5.20 | 3.95E-14 | 2506 |
| Cluster-40555.200030 | 0 | 0 | 1.37 | 1.23 | 0 | 0 | 1.48 | 1.53 | Inf | 3.86E-04 | Inf | 2.91E-05 | 1510 |
| Cluster-40555.200158 | 0 | 0 | 26.9 | 20.33 | 0 | 1.84 | 16.49 | 18.32 | Inf | 4.18E-52 | 4.25 | 1.20E-22 | 1483 |
| Cluster-40555.200162 | 0 | 0 | 3.89 | 5.62 | 0 | 0.17 | 1.15 | 1.09 | Inf | 4.17E-13 | 4.01 | 4.29E-02 | 1300 |
| Cluster-40555.200330 | 0 | 0 | 19.37 | 18.8 | 0.13 | 0.13 | 13.92 | 16.98 | Inf | 4.99E-34 | 6.97 | 2.93E-24 | 1009 |
| Cluster-40555.200402 | 0 | 0 | 2.85 | 3.26 | 0.63 | 0.9 | 6.51 | 5.56 | Inf | 9.93E-08 | 3.03 | 5.92E-06 | 1230 |
| Cluster-40555.200535 | 0 | 0 | 2.1 | 1.13 | 0 | 0 | 2.62 | 3.98 | Inf | 4.07E-08 | Inf | 2.96E-12 | 2885 |
| Cluster-40555.200586 | 0 | 0 | 4.94 | 4.74 | 0 | 0.31 | 3.46 | 3.29 | Inf | 4.19E-19 | 4.43 | 5.83E-09 | 1811 |
| Cluster-40555.200598 | 0 | 0 | 3.57 | 2.22 | 0 | 0 | 2.76 | 3.07 | Inf | 2.31E-05 | Inf | 5.14E-06 | 1001 |
| Cluster-40555.200833 | 0 | 0 | 8 | 8.26 | 0 | 0 | 7.55 | 9.83 | Inf | 6.45E-06 | Inf | 1.08E-06 | 542 |
| Cluster-40555.200946 | 0 | 0 | 0.55 | 1.08 | 0 | 0 | 0.95 | 0.99 | Inf | 4.35E-04 | Inf | 3.46E-05 | 2156 |
| Cluster-40555.200954 | 0 | 0 | 2.82 | 2.2 | 0 | 0 | 3.27 | 3.71 | Inf | 1.16E-03 | Inf | 1.21E-05 | 844 |
| Cluster-40555.200992 | 0 | 0 | 13.56 | 12.72 | 0.27 | 0.28 | 12.5 | 12.66 | Inf | 3.87E-06 | 5.55 | 8.47E-05 | 442 |
| Cluster-40555.201037 | 0 | 0 | 3.72 | 2.22 | 0 | 0.16 | 6.56 | 5.83 | Inf | 3.16E-02 | 6.28 | 1.27E-04 | 574 |

| | | | | | | | | | | | | | |
|---|---|---|---|---|---|---|---|---|---|---|---|---|---|
| Cluster-40555.201039 | 0 | 0 | 3.04 | 2.85 | 0.13 | 0.07 | 2.86 | 4.05 | Inf | 2.32E-05 | 5.18 | 4.23E-05 | 971 |
| Cluster-40555.201457 | 0 | 0 | 42.13 | 61.1 | 0 | 0 | 5.67 | 3.59 | Inf | 3.02E-24 | Inf | 1.53E-12 | 1225 |
| Cluster-40555.201479 | 0 | 0 | 35.93 | 26.18 | 3.4 | 3.28 | 30.45 | 22.52 | Inf | 6.93E-15 | 3.04 | 4.99E-05 | 449 |
| Cluster-40555.201482 | 0 | 0 | 5.9 | 6.84 | 0 | 0 | 4.08 | 4.05 | Inf | 1.58E-07 | Inf | 4.11E-05 | 714 |
| Cluster-40555.201538 | 0 | 0 | 1.62 | 1.72 | 0 | 0 | 1.49 | 2.25 | Inf | 7.62E-10 | Inf | 5.12E-10 | 2521 |
| Cluster-40555.201562 | 0 | 0 | 6.2 | 5.71 | 0.41 | 0 | 4.79 | 4.33 | Inf | 6.58E-37 | 4.55 | 9.33E-17 | 2911 |
| Cluster-40555.201711 | 0 | 0 | 6.79 | 5.95 | 0.19 | 0 | 3.1 | 3.09 | Inf | 1.75E-29 | 5.10 | 5.12E-11 | 2168 |
| Cluster-40555.201766 | 0 | 0 | 16.9 | 19.46 | 0 | 0.22 | 7.73 | 8.81 | Inf | 1.75E-21 | 6.28 | 5.50E-09 | 724 |
| Cluster-40555.201941 | 0 | 0 | 19.62 | 20.61 | 9.88 | 1.92 | 18.49 | 24.86 | Inf | 2.48E-42 | 1.98 | 2.13E-02 | 1181 |
| Cluster-40555.201949 | 0 | 0 | 5.47 | 5.24 | 0 | 0 | 4.82 | 8.66 | Inf | 2.03E-05 | Inf | 1.53E-05 | 655 |
| Cluster-40555.201964 | 0 | 0 | 0.61 | 0.59 | 0.52 | 0 | 1.08 | 1 | Inf | 6.23E-06 | 2.12 | 1.58E-02 | 4106 |
| Cluster-40555.202011 | 0 | 0 | 1.35 | 0.8 | 0.15 | 0.17 | 3.44 | 3.53 | Inf | 6.58E-05 | 4.54 | 1.29E-10 | 2089 |
| Cluster-40555.202106 | 0 | 0 | 4.11 | 4.73 | 0 | 0 | 2.31 | 2.57 | Inf | 4.06E-49 | Inf | 5.32E-30 | 5295 |
| Cluster-40555.202188 | 0 | 0 | 0.6 | 0.44 | 0 | 0.15 | 1.04 | 0.64 | Inf | 1.80E-03 | 3.57 | 1.84E-03 | 3008 |
| Cluster-40555.202399 | 0 | 0 | 3.48 | 2.36 | 0 | 0 | 1.33 | 1.99 | Inf | 8.46E-16 | Inf | 2.25E-09 | 2406 |
| Cluster-40555.202499 | 0 | 0 | 6.74 | 5.42 | 0 | 0.21 | 7.5 | 6.93 | Inf | 1.73E-30 | 6.11 | 4.49E-27 | 2352 |
| Cluster-40555.202551 | 0 | 0 | 1.43 | 1.66 | 0.2 | 0.33 | 2.12 | 1.58 | Inf | 4.62E-06 | 2.82 | 5.78E-03 | 1770 |
| Cluster-40555.202643 | 0 | 0 | 19.72 | 15.52 | 0.54 | 0.19 | 8.7 | 8.4 | Inf | 7.24E-56 | 4.62 | 3.59E-18 | 1792 |
| Cluster-40555.202769 | 0 | 0 | 8.67 | 3.9 | 0 | 0 | 3.61 | 2.45 | Inf | 3.17E-05 | Inf | 2.18E-05 | 898 |
| Cluster-40555.202777 | 0 | 0 | 3.37 | 3.21 | 0.18 | 0.29 | 2.31 | 3.27 | Inf | 7.13E-06 | 3.56 | 8.44E-03 | 953 |
| Cluster-40555.202798 | 0 | 0 | 3.66 | 2.84 | 0.63 | 0.78 | 4.8 | 5.06 | Inf | 7.39E-07 | 2.87 | 4.14E-04 | 1086 |
| Cluster-40555.202838 | 0 | 0 | 3.86 | 3.16 | 0 | 0.53 | 5.46 | 5.87 | Inf | 3.85E-05 | 4.48 | 5.88E-06 | 840 |
| Cluster-40555.202844 | 0 | 0 | 4.02 | 5.36 | 0 | 0 | 4.39 | 3.84 | Inf | 2.37E-08 | Inf | 9.06E-08 | 932 |
| Cluster-40555.202968 | 0 | 0 | 2.7 | 3.98 | 0.03 | 0 | 3.65 | 3.37 | Inf | 1.10E-08 | 7.30 | 1.00E-08 | 1235 |
| Cluster-40555.203081 | 0 | 0 | 3.56 | 2.03 | 0 | 0 | 4.05 | 3.95 | Inf | 3.06E-07 | Inf | 1.46E-11 | 1289 |
| Cluster-40555.203336 | 0 | 0 | 2.14 | 2.05 | 0 | 0.53 | 2.62 | 3.46 | Inf | 1.22E-04 | 3.60 | 6.67E-04 | 1129 |
| Cluster-40555.203338 | 0 | 0 | 3.03 | 3.35 | 0.11 | 0.46 | 6.74 | 5.87 | Inf | 1.87E-03 | 4.50 | 5.54E-05 | 692 |
| Cluster-40555.203339 | 0 | 0 | 41.5 | 43.21 | 0.16 | 0.25 | 15.55 | 35.66 | Inf | 1.25E-58 | 7.20 | 7.58E-04 | 929 |
| Cluster-40555.203496 | 0 | 0 | 1.12 | 1.17 | 0.06 | 0 | 0.96 | 0.69 | Inf | 1.19E-05 | 5.22 | 2.09E-03 | 2179 |
| Cluster-40555.203522 | 0 | 0 | 15.12 | 14.36 | 4.6 | 8.34 | 23.48 | 29.58 | Inf | 2.19E-28 | 2.09 | 5.41E-06 | 1047 |
| Cluster-40555.203570 | 0 | 0 | 3.77 | 3.7 | 0.07 | 0 | 3.46 | 5.16 | Inf | 5.86E-07 | 7.17 | 7.30E-08 | 981 |
| Cluster-40555.204029 | 0 | 0 | 4.77 | 3.86 | 0 | 0 | 5.29 | 5.26 | Inf | 5.55E-15 | Inf | 2.75E-19 | 1619 |

| | | | | | | | | | | | | | |
|---|---|---|---|---|---|---|---|---|---|---|---|---|---|
| Cluster-40555.204155 | 0 | 0 | 3.62 | 4.96 | 0 | 0 | 2.53 | 1.41 | Inf | 1.18E-09 | Inf | 1.24E-04 | 1109 |
| Cluster-40555.204174 | 0 | 0 | 15.24 | 9.28 | 0.21 | 0.05 | 4.92 | 8.55 | Inf | 8.49E-09 | 6.43 | 1.64E-04 | 543 |
| Cluster-40555.204187 | 0 | 0 | 3.89 | 2.78 | 0.38 | 0.93 | 4.24 | 6.68 | Inf | 6.63E-06 | 3.08 | 9.75E-04 | 954 |
| Cluster-40555.204195 | 0 | 0 | 3.75 | 3.83 | 0.16 | 0.15 | 8.82 | 9.47 | Inf | 3.85E-20 | 6.01 | 1.62E-30 | 2347 |
| Cluster-40555.204261 | 0 | 0 | 3.24 | 2.27 | 0 | 0 | 1.94 | 4.4 | Inf | 2.34E-07 | Inf | 1.16E-03 | 1304 |
| Cluster-40555.204328 | 0 | 0 | 3.23 | 2.49 | 0 | 0 | 1.48 | 0.84 | Inf | 2.19E-07 | Inf | 3.85E-03 | 1266 |
| Cluster-40555.204358 | 0 | 0 | 5.43 | 4.67 | 0 | 0 | 2.95 | 3.4 | Inf | 1.92E-03 | Inf | 2.82E-02 | 536 |
| Cluster-40555.204427 | 0 | 0 | 1.34 | 1.48 | 0 | 0 | 2.11 | 1.8 | Inf | 4.80E-07 | Inf | 1.21E-10 | 2173 |
| Cluster-40555.204444 | 0 | 0 | 67.65 | 76.64 | 0.06 | 0 | 60.68 | 56.63 | Inf | 4.74E-125 | 11.35 | 2.89E-95 | 2174 |
| Cluster-40555.204643 | 0 | 0 | 4.05 | 2.93 | 0 | 0 | 2.79 | 2.52 | Inf | 1.26E-06 | Inf | 1.49E-05 | 1007 |
| Cluster-40555.204657 | 0 | 0 | 58.64 | 60.5 | 0 | 0 | 40.11 | 43.21 | Inf | 2.49E-05 | Inf | 3.69E-03 | 281 |
| Cluster-40555.204681 | 0 | 0 | 4.55 | 8.05 | 1.47 | 0.57 | 3.62 | 5.04 | Inf | 1.46E-07 | 2.19 | 2.53E-02 | 1013 |
| Cluster-40555.205009 | 0 | 0 | 0.46 | 1.1 | 0 | 0 | 2.33 | 2.29 | Inf | 2.33E-02 | Inf | 1.74E-07 | 1404 |
| Cluster-40555.205165 | 0 | 0 | 5.4 | 5.68 | 0 | 0.26 | 5.14 | 5.32 | Inf | 1.30E-28 | 5.36 | 1.97E-19 | 2359 |
| Cluster-40555.205280 | 0 | 0 | 57.21 | 56.26 | 3.21 | 0.72 | 39.7 | 42.48 | Inf | 9.14E-99 | 4.49 | 1.90E-32 | 1384 |
| Cluster-40555.205391 | 0 | 0 | 3.45 | 5.81 | 0 | 0 | 9.59 | 4.41 | Inf | 5.72E-09 | Inf | 3.27E-06 | 1466 |
| Cluster-40555.205439 | 0 | 0 | 3.92 | 2.93 | 0 | 0.03 | 2 | 4.8 | Inf | 2.22E-02 | Inf | 2.28E-02 | 548 |
| Cluster-40555.205450 | 0 | 0 | 1.31 | 1.28 | 0 | 0.11 | 0.78 | 0.96 | Inf | 2.79E-07 | 3.95 | 3.70E-03 | 2416 |
| Cluster-40555.205473 | 0 | 0 | 7.54 | 8.04 | 0 | 0 | 6.19 | 7.96 | Inf | 1.32E-10 | Inf | 2.92E-10 | 781 |
| Cluster-40555.205870 | 0 | 0 | 3.14 | 1.64 | 0 | 0.44 | 1.45 | 1.45 | Inf | 1.09E-06 | 2.76 | 4.33E-02 | 1511 |
| Cluster-40555.205910 | 0 | 0 | 2.47 | 1.85 | 0 | 0 | 1.92 | 2.49 | Inf | 1.50E-06 | Inf | 1.96E-07 | 1452 |
| Cluster-40555.206174 | 0 | 0 | 2.2 | 3.22 | 0.92 | 0.16 | 5.82 | 4.98 | Inf | 8.56E-06 | 3.38 | 2.67E-05 | 1074 |
| Cluster-40555.206178 | 0 | 0 | 1.73 | 1.42 | 0.17 | 0.71 | 2.2 | 3.84 | Inf | 2.62E-10 | 2.83 | 2.43E-02 | 2809 |
| Cluster-40555.206319 | 0 | 0 | 3.77 | 4.41 | 0.32 | 0.11 | 2.32 | 4.07 | Inf | 1.18E-04 | 3.97 | 2.49E-02 | 702 |
| Cluster-40555.206329 | 0 | 0 | 4.67 | 4.09 | 1.68 | 1.18 | 4.04 | 5.07 | Inf | 7.98E-14 | 1.76 | 1.52E-02 | 1488 |
| Cluster-40555.206387 | 0 | 0 | 4.14 | 3.64 | 0.27 | 0.26 | 2.16 | 3.23 | Inf | 1.01E-16 | 3.39 | 5.41E-05 | 1937 |
| Cluster-40555.206413 | 0 | 0 | 6.08 | 9.49 | 0.04 | 0 | 1.2 | 1.09 | Inf | 1.30E-12 | 5.86 | 6.81E-03 | 1346 |
| Cluster-40555.206585 | 0 | 0 | 9.34 | 9.15 | 4.48 | 2.47 | 14.21 | 12.29 | Inf | 1.29E-18 | 2.02 | 1.10E-04 | 1050 |
| Cluster-40555.206636 | 0 | 0 | 1.04 | 0.61 | 0 | 0 | 1.78 | 2.29 | Inf | 7.54E-05 | Inf | 1.69E-13 | 2613 |
| Cluster-40555.206775 | 0 | 0 | 2.39 | 3.91 | 0 | 0 | 0.54 | 0.95 | Inf | 1.49E-09 | Inf | 1.76E-03 | 1946 |
| Cluster-40555.206802 | 0 | 0 | 46.51 | 44.58 | 0.42 | 0 | 3.09 | 2.09 | Inf | 1.39E-58 | 3.63 | 2.16E-02 | 885 |
| Cluster-40555.206999 | 0 | 0 | 4 | 3.77 | 3.07 | 4.59 | 10.02 | 10.16 | Inf | 2.50E-08 | 1.45 | 1.64E-02 | 1085 |

| | | | | | | | | | | | | | |
|---|---|---|---|---|---|---|---|---|---|---|---|---|---|
| Cluster-40555.207004 | 0 | 0 | 18.1 | 13.9 | 0 | 0 | 15.24 | 10.83 | Inf | 2.68E-33 | Inf | 1.73E-27 | 1142 |
| Cluster-40555.207131 | 0 | 0 | 0.77 | 0.44 | 0 | 0 | 1.12 | 1.52 | Inf | 1.88E-04 | Inf | 3.50E-11 | 3239 |
| Cluster-40555.207359 | 0 | 0 | 4.33 | 3.75 | 0 | 0 | 2.49 | 3.4 | Inf | 2.94E-05 | Inf | 3.81E-04 | 773 |
| Cluster-40555.207421 | 0 | 0 | 1.78 | 2.04 | 0 | 0 | 2.34 | 1.86 | Inf | 1.80E-14 | Inf | 8.33E-17 | 3166 |
| Cluster-40555.207652 | 0 | 0 | 10.03 | 6.78 | 0 | 0 | 8.36 | 6.73 | Inf | 3.02E-23 | Inf | 2.54E-29 | 1851 |
| Cluster-40555.207729 | 0 | 0 | 3.16 | 3.42 | 1.41 | 2.43 | 9.42 | 10.23 | Inf | 2.12E-09 | 2.41 | 1.52E-06 | 1357 |
| Cluster-40555.207731 | 0 | 0 | 17.86 | 14.21 | 0.52 | 0 | 12.32 | 11.2 | Inf | 1.48E-30 | 5.58 | 3.05E-17 | 1056 |
| Cluster-40555.207749 | 0 | 0 | 5.77 | 5.02 | 0 | 0 | 1.21 | 1.88 | Inf | 1.34E-11 | Inf | 1.50E-03 | 1091 |
| Cluster-40555.207785 | 0 | 0 | 6.02 | 9.1 | 0 | 0 | 3.64 | 1.61 | Inf | 4.78E-09 | Inf | 3.30E-03 | 711 |
| Cluster-40555.207794 | 0 | 0 | 0.4 | 0.37 | 0.05 | 0 | 0.8 | 0.81 | Inf | 1.47E-03 | 5.07 | 8.35E-06 | 3855 |
| Cluster-40555.207857 | 0 | 0 | 1.81 | 1.49 | 0 | 0 | 1.98 | 1.19 | Inf | 9.43E-13 | Inf | 2.87E-11 | 3269 |
| Cluster-40555.207876 | 0 | 0 | 4.72 | 3.79 | 0 | 0 | 1.52 | 1.37 | Inf | 3.19E-19 | Inf | 2.09E-07 | 2058 |
| Cluster-40555.207924 | 0 | 0 | 1.17 | 0.96 | 0 | 0 | 0.6 | 0.98 | Inf | 2.19E-03 | Inf | 9.96E-03 | 1529 |
| Cluster-40555.208083 | 0 | 0 | 1.97 | 3.61 | 0 | 0.11 | 2.93 | 4.76 | Inf | 8.47E-04 | 6.35 | 7.27E-05 | 781 |
| Cluster-40555.208105 | 0 | 0 | 4.37 | 6.4 | 0 | 0 | 5.16 | 2.33 | Inf | 1.01E-04 | Inf | 1.97E-03 | 599 |
| Cluster-40555.208114 | 0 | 0 | 14.22 | 9.37 | 0 | 0 | 14.27 | 26.33 | Inf | 2.32E-08 | Inf | 1.46E-06 | 540 |
| Cluster-40555.208143 | 0 | 0 | 0.48 | 0.5 | 0 | 0 | 0.47 | 0.35 | Inf | 2.15E-02 | Inf | 4.02E-02 | 2215 |
| Cluster-40555.208146 | 0 | 0 | 1.7 | 2.43 | 0.08 | 0.3 | 2.71 | 2.7 | Inf | 3.00E-06 | 3.85 | 9.14E-05 | 1404 |
| Cluster-40555.208280 | 0 | 0 | 7.31 | 4.17 | 0 | 0 | 4.37 | 3.47 | Inf | 1.87E-11 | Inf | 2.68E-16 | 1771 |
| Cluster-40555.208510 | 0 | 0 | 5.38 | 6.95 | 1.64 | 1.33 | 8.23 | 8.45 | Inf | 1.53E-13 | 2.55 | 3.29E-05 | 1097 |
| Cluster-40555.208582 | 0 | 0 | 172.17 | 144.76 | 27.69 | 51.64 | 336.75 | 381.2 | Inf | 2.41E-126 | 3.23 | 5.16E-27 | 918 |
| Cluster-40555.209105 | 0 | 0 | 14.74 | 14.21 | 3.87 | 4.6 | 18.97 | 14.61 | Inf | 4.49E-37 | 2.04 | 1.11E-06 | 1353 |
| Cluster-40555.209114 | 0 | 0 | 1.15 | 1.55 | 0 | 0 | 1.83 | 1.49 | Inf | 1.76E-03 | Inf | 1.19E-04 | 1263 |
| Cluster-40555.209201 | 0 | 0 | 2.01 | 1.26 | 0.64 | 0.26 | 2.19 | 2.82 | Inf | 1.92E-03 | 2.58 | 4.70E-02 | 1106 |
| Cluster-40555.209242 | 0 | 0 | 0.72 | 0.61 | 0 | 0 | 0.53 | 0.88 | Inf | 5.41E-12 | Inf | 3.94E-08 | 7246 |
| Cluster-40555.209258 | 0 | 0 | 1.66 | 1.97 | 0 | 0 | 1.05 | 2.37 | Inf | 1.60E-04 | Inf | 5.37E-03 | 1226 |
| Cluster-40555.209479 | 0 | 0 | 0.31 | 0.48 | 0 | 0 | 0.39 | 1.07 | Inf | 1.88E-03 | Inf | 1.63E-02 | 3614 |
| Cluster-40555.209549 | 0 | 0 | 3.12 | 3.42 | 0 | 0 | 1.68 | 1.42 | Inf | 8.03E-12 | Inf | 4.50E-06 | 1646 |
| Cluster-40555.209795 | 0 | 0 | 13.92 | 16.6 | 0 | 0.25 | 11.98 | 8.91 | Inf | 1.46E-21 | 6.29 | 4.24E-13 | 815 |
| Cluster-40555.210448 | 0 | 0 | 10.03 | 9.95 | 0 | 0.32 | 5.49 | 9.85 | Inf | 8.65E-19 | 5.54 | 8.16E-05 | 997 |
| Cluster-40555.210521 | 0 | 0 | 4.19 | 3.34 | 0 | 0.42 | 4.21 | 2.41 | Inf | 3.25E-05 | 3.87 | 5.74E-03 | 808 |
| Cluster-40555.210539 | 0 | 0 | 7.38 | 2.13 | 0 | 0 | 2.86 | 1.29 | Inf | 1.17E-02 | Inf | 8.83E-04 | 1105 |

| | | | | | | | | | | | | | |
|---|---|---|---|---|---|---|---|---|---|---|---|---|---|
| Cluster-40555.210859 | 0 | 0 | 9.08 | 5.62 | 0 | 0 | 3.63 | 5.55 | Inf | 4.85E-18 | Inf | 1.05E-12 | 2718 |
| Cluster-40555.210935 | 0 | 0 | 6.77 | 3.59 | 0 | 0 | 3.21 | 2.18 | Inf | 3.84E-07 | Inf | 1.02E-04 | 888 |
| Cluster-40555.210939 | 0 | 0 | 2.54 | 1.99 | 1.28 | 1.45 | 4.55 | 4.03 | Inf | 6.78E-08 | 1.72 | 1.37E-02 | 1621 |
| Cluster-40555.211100 | 0 | 0 | 1 | 3.26 | 0.66 | 0.25 | 2.44 | 1.68 | Inf | 1.60E-02 | 2.26 | 4.28E-02 | 1564 |
| Cluster-40555.211107 | 0 | 0 | 0.75 | 0.91 | 0.36 | 0.07 | 1.76 | 1.18 | Inf | 5.43E-03 | 2.88 | 2.42E-02 | 1691 |
| Cluster-40555.211110 | 0 | 0 | 1.8 | 0.62 | 0.23 | 0.29 | 2.28 | 1.62 | Inf | 8.26E-03 | 2.93 | 8.83E-04 | 2061 |
| Cluster-40555.211119 | 0 | 0 | 22.58 | 27.85 | 0.5 | 0 | 14.5 | 13.12 | Inf | 1.96E-29 | 5.89 | 1.77E-13 | 733 |
| Cluster-40555.211334 | 0 | 0 | 3.86 | 4.13 | 0 | 0 | 1.49 | 1.39 | Inf | 2.42E-06 | Inf | 2.40E-02 | 874 |
| Cluster-40555.211516 | 0 | 0 | 11.85 | 16.35 | 0 | 0 | 7.87 | 9.41 | Inf | 7.99E-19 | Inf | 2.85E-12 | 774 |
| Cluster-40555.211519 | 0 | 0 | 5.86 | 7.18 | 0 | 0 | 2.17 | 2.72 | Inf | 6.72E-27 | Inf | 1.89E-11 | 1909 |
| Cluster-40555.211692 | 0 | 0 | 44.12 | 46.24 | 0 | 0 | 29.08 | 43.12 | Inf | 3.38E-05 | Inf | 1.28E-03 | 295 |
| Cluster-40555.211734 | 0 | 0 | 2.4 | 2.45 | 0.07 | 0 | 1.99 | 1.45 | Inf | 2.27E-11 | 5.58 | 9.04E-07 | 2065 |
| Cluster-40555.211737 | 0 | 0 | 5.67 | 11.1 | 0 | 0.07 | 11.83 | 11.21 | Inf | 8.91E-07 | 8.60 | 5.09E-23 | 1085 |
| Cluster-40555.211783 | 0 | 0 | 2.34 | 2.59 | 0 | 0 | 23.94 | 16.49 | Inf | 5.69E-07 | Inf | 1.91E-28 | 1352 |
| Cluster-40555.211907 | 0 | 0 | 0.77 | 0.69 | 0 | 0 | 0.54 | 0.21 | Inf | 3.36E-04 | Inf | 2.97E-02 | 2537 |
| Cluster-40555.212352 | 0 | 0 | 6.38 | 5.63 | 0.32 | 0.52 | 9.18 | 8.07 | Inf | 3.12E-20 | 4.43 | 4.22E-16 | 1595 |
| Cluster-40555.212355 | 0 | 0 | 14.67 | 10.65 | 0 | 0 | 16.59 | 17.67 | Inf | 1.91E-18 | Inf | 4.28E-25 | 840 |
| Cluster-40555.212798 | 0 | 0 | 5.57 | 5.2 | 0.24 | 0 | 4.37 | 4.27 | Inf | 3.36E-04 | 4.89 | 1.37E-02 | 570 |
| Cluster-40555.212809 | 0 | 0 | 2.52 | 1.37 | 1.01 | 1.42 | 5.81 | 4.55 | Inf | 1.42E-07 | 2.15 | 1.10E-04 | 1884 |
| Cluster-40555.212813 | 0 | 0 | 0.31 | 0.91 | 0.17 | 0.75 | 1.89 | 1.87 | Inf | 1.73E-02 | 2.04 | 4.75E-03 | 2847 |
| Cluster-40555.212852 | 0 | 0 | 5.71 | 4.51 | 3.81 | 2.11 | 14.61 | 10.81 | Inf | 5.22E-03 | 2.17 | 4.09E-02 | 496 |
| Cluster-40555.212893 | 0 | 0 | 1.54 | 1.85 | 0 | 0 | 2.45 | 1.62 | Inf | 4.59E-03 | Inf | 5.78E-04 | 964 |
| Cluster-40555.212907 | 0 | 0 | 0.32 | 0.72 | 0 | 0 | 1.06 | 1.17 | Inf | 1.73E-03 | Inf | 9.66E-11 | 3646 |
| Cluster-40555.213030 | 0 | 0 | 5.64 | 3.88 | 0 | 0 | 2.38 | 2.28 | Inf | 1.07E-12 | Inf | 6.78E-07 | 1307 |
| Cluster-40555.213136 | 0 | 0 | 1.49 | 1.51 | 0.08 | 0 | 0.69 | 1.13 | Inf | 9.40E-07 | 4.63 | 4.87E-03 | 2002 |
| Cluster-40555.213225 | 0 | 0 | 2.58 | 1.15 | 0.11 | 0.77 | 2.6 | 2.89 | Inf | 8.65E-06 | 2.68 | 1.86E-07 | 3829 |
| Cluster-40555.213305 | 0 | 0 | 5.94 | 2.82 | 0.25 | 0 | 1.95 | 1.28 | Inf | 2.89E-06 | 3.86 | 5.83E-03 | 1424 |
| Cluster-40555.213340 | 0 | 0 | 2.22 | 1.82 | 0 | 0 | 3.59 | 3.07 | Inf | 4.30E-07 | Inf | 6.51E-13 | 1638 |
| Cluster-40555.213365 | 0 | 0 | 2.03 | 1.26 | 0 | 0 | 0.68 | 1.16 | Inf | 7.76E-04 | Inf | 2.32E-02 | 1202 |
| Cluster-40555.213415 | 0 | 0 | 2.43 | 1.63 | 0 | 0 | 0.93 | 1.16 | Inf | 8.35E-05 | Inf | 1.20E-02 | 1209 |
| Cluster-40555.213499 | 0 | 0 | 0.47 | 0.56 | 0 | 0 | 0.98 | 1.33 | Inf | 2.99E-02 | Inf | 7.07E-06 | 2045 |
| Cluster-40555.213527 | 0 | 0 | 2.19 | 1.54 | 0 | 0 | 3.4 | 2.34 | Inf | 4.27E-03 | Inf | 1.54E-05 | 945 |

| | | | | | | | | | | | | | |
|---|---|---|---|---|---|---|---|---|---|---|---|---|---|
| Cluster-40555.213631 | 0 | 0 | 1.36 | 1.22 | 0.45 | 0.47 | 2.66 | 2.19 | Inf | 1.83E-04 | 2.47 | 7.23E-03 | 1619 |
| Cluster-40555.213806 | 0 | 0 | 1.27 | 2.27 | 0 | 0.1 | 2.9 | 4.26 | Inf | 9.70E-04 | 5.89 | 1.10E-06 | 1066 |
| Cluster-40555.213835 | 0 | 0 | 6.08 | 3.79 | 0 | 0 | 0.83 | 2 | Inf | 5.57E-14 | Inf | 8.07E-03 | 1464 |
| Cluster-40555.213980 | 0 | 0 | 3.35 | 1.18 | 0 | 0.17 | 2.78 | 2.37 | Inf | 1.25E-03 | 4.91 | 6.49E-11 | 2522 |
| Cluster-40555.213994 | 0 | 0 | 1.01 | 1.61 | 0 | 0.05 | 0.93 | 0.95 | Inf | 1.65E-03 | 5.32 | 3.61E-02 | 1300 |
| Cluster-40555.214045 | 0 | 0 | 1.15 | 2.12 | 0.31 | 0 | 3.24 | 3.5 | Inf | 7.12E-03 | 4.71 | 2.76E-04 | 939 |
| Cluster-40555.214131 | 0 | 0 | 2.68 | 1.18 | 0.25 | 0 | 6.18 | 5.66 | Inf | 3.99E-05 | 5.73 | 1.84E-23 | 2578 |
| Cluster-40555.214158 | 0 | 0 | 3.04 | 2.6 | 0 | 0.22 | 3.65 | 5.94 | Inf | 1.01E-12 | 5.45 | 1.93E-06 | 2014 |
| Cluster-40555.214292 | 0 | 0 | 0.65 | 0.84 | 0 | 0 | 0.94 | 1.14 | Inf | 1.12E-02 | Inf | 3.23E-04 | 1699 |
| Cluster-40555.214332 | 0 | 0 | 1.46 | 0.77 | 0.18 | 0.45 | 2.12 | 1.81 | Inf | 1.32E-06 | 2.67 | 3.05E-04 | 2609 |
| Cluster-40555.214537 | 0 | 0 | 5.42 | 2.45 | 0 | 0 | 2.72 | 1.89 | Inf | 3.06E-03 | Inf | 4.76E-02 | 602 |
| Cluster-40555.214608 | 0.01 | 0 | 2.47 | 2.29 | 0.14 | 0.15 | 2.45 | 3.41 | Inf | 6.43E-13 | 4.37 | 2.66E-09 | 2356 |
| Cluster-40555.214639 | 0 | 0 | 7.02 | 7.17 | 0.08 | 0 | 1.1 | 1.17 | Inf | 4.62E-22 | 5.03 | 6.59E-03 | 1492 |
| Cluster-40555.214742 | 0 | 0 | 6.96 | 5.06 | 0.51 | 0 | 3.47 | 2.94 | Inf | 2.88E-14 | 3.79 | 2.25E-04 | 1192 |
| Cluster-40555.214950 | 0 | 0 | 2.96 | 2.55 | 0 | 0 | 1.37 | 1.94 | Inf | 4.30E-10 | Inf | 1.26E-06 | 1678 |
| Cluster-40555.214979 | 0 | 0 | 2.28 | 2.11 | 0 | 0 | 0.98 | 1.16 | Inf | 2.33E-09 | Inf | 5.67E-05 | 1906 |
| Cluster-40555.215102 | 0 | 0 | 17.58 | 25.78 | 4.51 | 3.99 | 21.28 | 21.38 | Inf | 8.75E-16 | 2.39 | 1.24E-05 | 689 |
| Cluster-40555.215316 | 0 | 0 | 1.53 | 1.53 | 0.77 | 0.39 | 3.73 | 2.64 | Inf | 8.29E-09 | 2.57 | 2.77E-05 | 2472 |
| Cluster-40555.215513 | 0 | 0 | 6.82 | 4.9 | 0 | 0 | 5.11 | 4.92 | Inf | 2.05E-03 | Inf | 4.39E-03 | 492 |
| Cluster-40555.215533 | 0 | 0 | 1.29 | 0.8 | 0.33 | 0.07 | 1.72 | 1.67 | Inf | 1.25E-04 | 3.11 | 1.68E-03 | 2055 |
| Cluster-40555.215566 | 0 | 0 | 3.98 | 4.5 | 0.26 | 0 | 6.11 | 6.79 | Inf | 3.21E-07 | 5.59 | 8.06E-09 | 913 |
| Cluster-40555.215567 | 0 | 0 | 9.81 | 13.96 | 0.87 | 1.28 | 15.39 | 9.43 | Inf | 1.06E-08 | 3.57 | 1.27E-04 | 537 |
| Cluster-40555.215570 | 0 | 0 | 1.48 | 0.52 | 0 | 0 | 0.77 | 1.71 | Inf | 1.09E-03 | Inf | 1.58E-04 | 5663 |
| Cluster-40555.215608 | 0 | 0 | 1.46 | 1.73 | 0 | 0 | 1.91 | 2.28 | Inf | 9.29E-04 | Inf | 2.41E-05 | 1175 |
| Cluster-40555.215746 | 0 | 0 | 0.87 | 1.64 | 0 | 0.07 | 2.61 | 2.39 | Inf | 8.41E-03 | 6.52 | 1.56E-05 | 1145 |
| Cluster-40555.215786 | 0 | 0 | 1.58 | 2.99 | 0 | 0 | 4.89 | 2.4 | Inf | 1.36E-05 | Inf | 3.59E-06 | 1482 |
| Cluster-40555.215920 | 0 | 0 | 1.26 | 1.12 | 0.13 | 0 | 1.49 | 1.24 | Inf | 1.91E-09 | 4.61 | 2.04E-07 | 3322 |
| Cluster-40555.215980 | 0 | 0 | 5.35 | 6.5 | 0 | 0 | 5.2 | 2.98 | Inf | 1.70E-12 | Inf | 2.36E-08 | 1063 |
| Cluster-40555.216099 | 0 | 0 | 2.67 | 3 | 0 | 0 | 0.96 | 1.05 | Inf | 1.99E-08 | Inf | 3.66E-03 | 1400 |
| Cluster-40555.216100 | 0 | 0 | 1.34 | 1.41 | 0.39 | 0.2 | 2.73 | 1.59 | Inf | 1.41E-05 | 3.00 | 9.71E-04 | 1849 |
| Cluster-40555.216277 | 0 | 0 | 1.61 | 0.7 | 0 | 0 | 1.56 | 1.7 | Inf | 6.20E-04 | Inf | 1.22E-07 | 1901 |
| Cluster-40555.216826 | 0 | 0 | 3.43 | 1.25 | 0 | 0 | 1.96 | 2.9 | Inf | 3.98E-03 | Inf | 5.32E-07 | 1279 |

| | | | | | | | | | | | | | |
|---|---|---|---|---|---|---|---|---|---|---|---|---|---|
| Cluster-40555.217096 | 0 | 0 | 2.05 | 3.44 | 0 | 0 | 1.23 | 1.85 | Inf | 3.72E-08 | Inf | 2.34E-05 | 1489 |
| Cluster-40555.217316 | 0 | 0 | 4.13 | 3.55 | 0.48 | 0.3 | 7.68 | 6.66 | Inf | 8.38E-05 | 4.26 | 7.58E-06 | 753 |
| Cluster-40555.217393 | 0 | 0 | 2.26 | 1.12 | 0 | 0 | 3.45 | 2.69 | Inf | 8.26E-06 | Inf | 5.73E-13 | 1771 |
| Cluster-40555.217395 | 0 | 0 | 1.01 | 0.52 | 0 | 0 | 1.13 | 0.47 | Inf | 4.41E-03 | Inf | 5.16E-03 | 1930 |
| Cluster-40555.217588 | 0 | 0 | 1.43 | 2.87 | 0 | 0 | 3.13 | 1.48 | Inf | 1.36E-03 | Inf | 5.95E-04 | 902 |
| Cluster-40555.217660 | 0 | 0 | 8.16 | 5.41 | 0.44 | 0.25 | 3.96 | 4.56 | Inf | 3.37E-15 | 3.81 | 1.77E-05 | 1147 |
| Cluster-40555.217701 | 0 | 0 | 10.7 | 8.8 | 0 | 0 | 3.95 | 4.47 | Inf | 1.92E-11 | Inf | 2.89E-05 | 717 |
| Cluster-40555.217887 | 0 | 0 | 1.9 | 1.67 | 0 | 0 | 1.53 | 1.77 | Inf | 9.78E-03 | Inf | 1.02E-02 | 876 |
| Cluster-40555.217936 | 0 | 0 | 7.49 | 6.57 | 0 | 0 | 1.47 | 1.11 | Inf | 4.45E-19 | Inf | 8.03E-04 | 1330 |
| Cluster-40555.218079 | 0 | 0 | 0.53 | 0.41 | 0 | 0 | 0.92 | 0.94 | Inf | 8.01E-03 | Inf | 2.20E-06 | 2668 |
| Cluster-40555.218086 | 0 | 0 | 8.85 | 5.67 | 0 | 0 | 4.93 | 5.83 | Inf | 1.71E-13 | Inf | 4.28E-11 | 996 |
| Cluster-40555.218309 | 0 | 0 | 7.48 | 4.36 | 2.58 | 3.2 | 10.06 | 9.34 | Inf | 7.95E-13 | 1.80 | 2.56E-05 | 2081 |
| Cluster-40555.218492 | 0 | 0 | 0.74 | 0.49 | 0 | 0 | 1.18 | 1.22 | Inf | 1.82E-02 | Inf | 1.23E-05 | 1914 |
| Cluster-40555.218499 | 0 | 0 | 4.21 | 7.89 | 0 | 0 | 8.91 | 9.66 | Inf | 6.55E-05 | Inf | 7.39E-08 | 568 |
| Cluster-40555.218545 | 0 | 0 | 3.63 | 4.64 | 0 | 0 | 3.63 | 2.42 | Inf | 1.76E-22 | Inf | 1.38E-17 | 2389 |
| Cluster-40555.218797 | 0 | 0 | 1.74 | 13.86 | 0.12 | 0.16 | 2.49 | 8.09 | Inf | 4.97E-10 | 4.34 | 2.11E-08 | 2236 |
| Cluster-40555.218824 | 0 | 0 | 0.57 | 1.48 | 0 | 0 | 1.34 | 1.31 | Inf | 5.94E-03 | Inf | 1.46E-05 | 1742 |
| Cluster-40555.218847 | 0 | 0 | 1.6 | 0.54 | 0.49 | 0 | 1.41 | 2.51 | Inf | 8.26E-03 | 3.11 | 3.24E-02 | 2323 |
| Cluster-40555.218901 | 0 | 0 | 1.68 | 0.43 | 0 | 0 | 0.68 | 0.77 | Inf | 3.53E-02 | Inf | 1.71E-05 | 2943 |
| Cluster-40555.219108 | 0 | 0 | 3.44 | 3.34 | 0.24 | 0.08 | 5.66 | 5.43 | Inf | 3.22E-16 | 5.17 | 1.79E-17 | 2119 |
| Cluster-40555.219206 | 0 | 0 | 2.13 | 2.75 | 0 | 0 | 4.67 | 2.79 | Inf | 4.76E-03 | Inf | 4.38E-05 | 753 |
| Cluster-40555.219243 | 0 | 0 | 8.13 | 11.11 | 0.51 | 0 | 3.8 | 5.36 | Inf | 3.40E-11 | 4.22 | 1.97E-03 | 702 |
| Cluster-40555.219332 | 0 | 0 | 0.31 | 0.45 | 0 | 0 | 0.48 | 0.33 | Inf | 1.59E-03 | Inf | 4.09E-04 | 3902 |
| Cluster-40555.219537 | 0 | 0 | 8.9 | 13.24 | 2.35 | 2.68 | 18.06 | 15.65 | Inf | 1.13E-06 | 2.80 | 1.13E-03 | 489 |
| Cluster-40555.219861 | 0 | 0 | 0.63 | 0.78 | 0 | 0 | 0.57 | 0.51 | Inf | 5.43E-03 | Inf | 1.72E-02 | 1944 |
| Cluster-40555.219922 | 0 | 0 | 2.49 | 1.98 | 0 | 0 | 1.36 | 1.81 | Inf | 7.10E-06 | Inf | 1.34E-04 | 1292 |
| Cluster-40555.219949 | 0 | 0 | 4.81 | 3.43 | 0 | 0 | 3.77 | 4.05 | Inf | 1.88E-02 | Inf | 1.71E-02 | 504 |
| Cluster-40555.220286 | 0 | 0 | 0.69 | 0.4 | 0.6 | 0 | 1.66 | 1.44 | Inf | 1.37E-03 | 2.47 | 2.99E-03 | 2952 |
| Cluster-40555.220403 | 0 | 0 | 36.73 | 27.8 | 0 | 0 | 16.52 | 19.98 | Inf | 8.44E-48 | Inf | 6.28E-30 | 942 |
| Cluster-40555.220433 | 0 | 0 | 2.49 | 2.34 | 0 | 0 | 0.68 | 0.73 | Inf | 1.33E-09 | Inf | 5.15E-03 | 1794 |
| Cluster-40555.220500 | 0 | 0 | 3.87 | 3.33 | 1.62 | 0.87 | 3.88 | 4.19 | Inf | 1.37E-13 | 1.78 | 1.12E-02 | 1729 |
| Cluster-40555.220782 | 0 | 0 | 0.73 | 1.03 | 0.04 | 0 | 0.99 | 0.58 | Inf | 4.17E-05 | 5.34 | 8.78E-04 | 2489 |

| | | | | | | | | | | | | | |
|---|---|---|---|---|---|---|---|---|---|---|---|---|---|
| Cluster-40555.220799 | 0 | 0 | 4.58 | 8.93 | 6.18 | 7.01 | 24.69 | 22.11 | Inf | 8.80E-04 | 1.87 | 1.42E-02 | 476 |
| Cluster-40555.220845 | 0 | 0 | 1.13 | 1.54 | 0 | 0 | 0.83 | 0.37 | Inf | 6.10E-06 | Inf | 9.16E-03 | 1964 |
| Cluster-40555.220937 | 0 | 0 | 0.25 | 0.4 | 0 | 0 | 0.46 | 0.38 | Inf | 2.87E-04 | Inf | 5.36E-06 | 5292 |
| Cluster-40555.220949 | 0 | 0 | 0.17 | 0.25 | 0 | 0 | 0.16 | 0.33 | Inf | 2.72E-02 | Inf | 6.49E-03 | 4642 |
| Cluster-40555.220976 | 0 | 0 | 3.13 | 4.75 | 0 | 0.44 | 4.58 | 4.9 | Inf | 2.55E-11 | 4.40 | 1.04E-08 | 1340 |
| Cluster-40555.221043 | 0 | 0 | 2.5 | 3.31 | 0 | 0 | 3.78 | 3.05 | Inf | 1.39E-13 | Inf | 1.11E-16 | 2036 |
| Cluster-40555.221061 | 0 | 0 | 22.29 | 15.49 | 0.13 | 0.05 | 7.79 | 9.83 | Inf | 5.42E-17 | 7.16 | 7.53E-08 | 618 |
| Cluster-40555.221085 | 0 | 0 | 1.36 | 1.31 | 0 | 0 | 3.2 | 2.85 | Inf | 3.49E-08 | Inf | 2.19E-19 | 2630 |
| Cluster-40555.221136 | 0 | 0 | 2.66 | 2.61 | 0 | 0 | 2.39 | 4.23 | Inf | 4.29E-03 | Inf | 3.12E-04 | 727 |
| Cluster-40555.221140 | 0 | 0 | 5.47 | 5.25 | 0.68 | 0.96 | 11.74 | 11.52 | Inf | 1.23E-08 | 3.88 | 1.23E-09 | 881 |
| Cluster-40555.221192 | 0 | 0 | 3.79 | 2.86 | 0.16 | 0.19 | 2.65 | 2.35 | Inf | 5.58E-17 | 3.87 | 2.77E-07 | 2273 |
| Cluster-40555.221637 | 0 | 0 | 3.35 | 2.85 | 0 | 0 | 4.27 | 2.81 | Inf | 5.55E-08 | Inf | 5.51E-10 | 1260 |
| Cluster-40555.221664 | 0 | 0 | 1.74 | 1.68 | 0.13 | 0.16 | 2.12 | 1.91 | Inf | 1.58E-11 | 3.83 | 1.92E-07 | 2868 |
| Cluster-40555.221712 | 0 | 0 | 2.45 | 1.48 | 0.27 | 0 | 4.38 | 4.41 | Inf | 1.30E-12 | 5.15 | 1.47E-22 | 3734 |
| Cluster-40555.221814 | 0 | 0 | 0.73 | 0.31 | 0 | 0 | 0.36 | 0.85 | Inf | 2.14E-02 | Inf | 1.90E-02 | 2257 |
| Cluster-40555.221953 | 0 | 0 | 1.94 | 1.61 | 0.1 | 0.21 | 1.65 | 2.97 | Inf | 2.61E-02 | 4.12 | 3.67E-02 | 787 |
| Cluster-40555.221960 | 0 | 0 | 2.95 | 2.8 | 0 | 0 | 8.7 | 8.52 | Inf | 5.79E-05 | Inf | 5.93E-16 | 939 |
| Cluster-40555.221971 | 0 | 0 | 4.42 | 1.61 | 0.33 | 0.35 | 2.13 | 1.82 | Inf | 9.18E-04 | 2.55 | 1.00E-02 | 1758 |
| Cluster-40555.222131 | 0 | 0 | 0.65 | 0.76 | 0 | 0 | 1.6 | 2.23 | Inf | 1.51E-05 | Inf | 7.92E-15 | 3312 |
| Cluster-40555.222236 | 0 | 0 | 2.1 | 3.97 | 0.32 | 0 | 2.99 | 3.2 | Inf | 6.25E-03 | 4.68 | 3.65E-02 | 630 |
| Cluster-40555.222321 | 0 | 0 | 1.25 | 1.66 | 0.06 | 0 | 1.93 | 1.7 | Inf | 9.84E-03 | 5.94 | 4.26E-03 | 998 |
| Cluster-40555.222356 | 0 | 0 | 2.24 | 1.58 | 0 | 0.12 | 1.69 | 0.83 | Inf | 6.15E-04 | 4.41 | 4.93E-02 | 1091 |
| Cluster-40555.222577 | 0 | 0 | 1.08 | 0.97 | 0 | 0.07 | 1.11 | 0.99 | Inf | 1.83E-04 | 4.64 | 1.41E-03 | 1991 |
| Cluster-40555.222606 | 0 | 0 | 0.89 | 0.96 | 0 | 0.48 | 2.05 | 1.68 | Inf | 2.51E-08 | 2.97 | 1.66E-06 | 3711 |
| Cluster-40555.222668 | 0 | 0 | 0.75 | 0.95 | 0 | 0 | 2.06 | 1.95 | Inf | 8.40E-04 | Inf | 5.96E-10 | 1999 |
| Cluster-40555.222682 | 0 | 0 | 2.04 | 1.84 | 0 | 0 | 2.91 | 3.56 | Inf | 1.83E-04 | Inf | 2.59E-08 | 1167 |
| Cluster-40555.223118 | 0 | 0 | 0.8 | 1.08 | 0 | 0 | 0.81 | 1.91 | Inf | 2.37E-03 | Inf | 7.18E-03 | 1654 |
| Cluster-40555.223269 | 0 | 0 | 2.09 | 1.52 | 0 | 0 | 0.96 | 0.88 | Inf | 9.84E-04 | Inf | 4.70E-02 | 1102 |
| Cluster-40555.223378 | 0 | 0 | 3.6 | 3.67 | 0 | 0 | 3.85 | 4.73 | Inf | 8.95E-08 | Inf | 4.81E-10 | 1087 |
| Cluster-40555.223414 | 0 | 0 | 46.67 | 45.07 | 0 | 0 | 12.11 | 15.13 | Inf | 1.45E-55 | Inf | 1.15E-20 | 837 |
| Cluster-40555.223622 | 0 | 0 | 1.87 | 2.13 | 0.24 | 0 | 2.45 | 3.76 | Inf | 5.25E-03 | 4.80 | 1.58E-03 | 850 |
| Cluster-40555.223623 | 0 | 0 | 1.49 | 1.93 | 0 | 0 | 1.79 | 2.44 | Inf | 9.61E-04 | Inf | 4.69E-05 | 1115 |

| | | | | | | | | | | | | | |
|---|---|---|---|---|---|---|---|---|---|---|---|---|---|
| Cluster-40555.223668 | 0 | 0 | 0.83 | 0.8 | 0 | 0.65 | 2.36 | 2.06 | Inf | 4.26E-05 | 2.76 | 3.76E-05 | 2714 |
| Cluster-40555.223708 | 0 | 0 | 1.23 | 1.76 | 0.05 | 0 | 0.5 | 0.85 | Inf | 1.09E-06 | 4.75 | 2.69E-02 | 1961 |
| Cluster-40555.223721 | 0 | 0 | 0.69 | 1.36 | 0.04 | 0.13 | 1.74 | 1.78 | Inf | 4.42E-02 | 4.30 | 1.49E-02 | 1041 |
| Cluster-40555.223838 | 0 | 0 | 4.97 | 4.87 | 0 | 0 | 3.58 | 2.24 | Inf | 1.65E-04 | Inf | 8.97E-03 | 620 |
| Cluster-40555.223997 | 0 | 0 | 2.61 | 3.04 | 0.53 | 0.41 | 3.66 | 4.28 | Inf | 1.58E-07 | 3.16 | 1.28E-04 | 1284 |
| Cluster-40555.224022 | 0 | 0 | 3.56 | 3.96 | 0 | 0 | 1.18 | 3.09 | Inf | 6.21E-12 | Inf | 1.06E-02 | 1474 |
| Cluster-40555.224037 | 0 | 0 | 4.16 | 3.02 | 0 | 0 | 9.55 | 8.39 | Inf | 2.53E-02 | Inf | 2.27E-06 | 521 |
| Cluster-40555.224061 | 0 | 0 | 3 | 3.39 | 0 | 0 | 8.31 | 5.13 | Inf | 1.35E-04 | Inf | 1.53E-10 | 820 |
| Cluster-40555.224085 | 0 | 0 | 5.89 | 7.61 | 0.17 | 0 | 3.12 | 6.34 | Inf | 6.04E-05 | 5.91 | 6.35E-03 | 543 |
| Cluster-40555.224371 | 0 | 0 | 1.02 | 0.7 | 0.19 | 0 | 2.03 | 1.28 | Inf | 3.16E-03 | 4.28 | 2.55E-04 | 1791 |
| Cluster-40555.224419 | 0 | 0 | 3.16 | 3.5 | 0 | 0 | 5.24 | 3.22 | Inf | 1.87E-03 | Inf | 8.68E-05 | 672 |
| Cluster-40555.224472 | 0 | 0 | 0.49 | 0.72 | 0.02 | 0 | 1.34 | 1.39 | Inf | 3.63E-02 | 6.48 | 1.07E-04 | 1661 |
| Cluster-40555.224481 | 0 | 0 | 1.22 | 2.17 | 0.08 | 0.2 | 2.43 | 2.71 | Inf | 1.11E-07 | 4.28 | 4.45E-11 | 3072 |
| Cluster-40555.224631 | 0 | 0 | 2.24 | 2.25 | 0.36 | 0 | 1.63 | 2.93 | Inf | 4.72E-04 | 3.92 | 1.32E-02 | 970 |
| Cluster-40555.224717 | 0 | 0 | 0.44 | 0.48 | 0 | 0.02 | 0.48 | 0.27 | Inf | 1.87E-03 | 5.48 | 1.76E-02 | 3229 |
| Cluster-40555.224976 | 0 | 0 | 0.82 | 2.55 | 0 | 0 | 4.34 | 7.76 | Inf | 6.93E-03 | Inf | 1.88E-08 | 2507 |
| Cluster-40555.225010 | 0 | 0 | 1.61 | 1.07 | 0.04 | 0 | 1.53 | 2.8 | Inf | 8.04E-04 | 6.86 | 1.53E-04 | 1409 |
| Cluster-40555.225014 | 0 | 0 | 3.24 | 10.65 | 0 | 0 | 5.25 | 6 | Inf | 3.52E-03 | Inf | 5.69E-18 | 1436 |
| Cluster-40555.225097 | 0 | 0 | 4.94 | 4.64 | 0 | 0 | 3.04 | 2.89 | Inf | 6.43E-28 | Inf | 4.24E-19 | 2637 |
| Cluster-40555.225174 | 0 | 0 | 2.71 | 1.51 | 0 | 0 | 1.85 | 2.2 | Inf | 3.16E-08 | Inf | 4.89E-09 | 1829 |
| Cluster-40555.225196 | 0 | 0 | 1.49 | 1.23 | 0.14 | 0.36 | 2.22 | 1.52 | Inf | 1.41E-08 | 2.96 | 7.71E-05 | 2714 |
| Cluster-40555.225235 | 0 | 0 | 4.76 | 3.13 | 0.26 | 0.39 | 5.08 | 6.63 | Inf | 1.47E-03 | 4.17 | 1.32E-03 | 624 |
| Cluster-40555.225238 | 0 | 0 | 4.1 | 4.42 | 0 | 0.62 | 6.86 | 5.28 | Inf | 2.89E-02 | 4.07 | 3.35E-02 | 473 |
| Cluster-40555.225239 | 0 | 0 | 1.74 | 1.23 | 0 | 0.63 | 2.08 | 2.11 | Inf | 1.12E-07 | 2.76 | 3.07E-04 | 2284 |
| Cluster-40555.225245 | 0 | 0 | 1.55 | 1.16 | 0 | 0 | 0.33 | 0.48 | Inf | 1.16E-07 | Inf | 1.97E-02 | 2469 |
| Cluster-40555.225368 | 0 | 0 | 0.4 | 0.25 | 0.12 | 0.19 | 0.75 | 0.8 | Inf | 2.78E-02 | 2.41 | 4.95E-02 | 3126 |
| Cluster-40555.225587 | 0 | 0 | 0.98 | 0.53 | 0 | 0 | 0.52 | 1.5 | Inf | 5.36E-04 | Inf | 2.94E-02 | 2421 |
| Cluster-40555.225619 | 0 | 0 | 0.4 | 0.19 | 0 | 0 | 0.31 | 0.31 | Inf | 2.34E-03 | Inf | 4.68E-04 | 4945 |
| Cluster-40555.225688 | 0 | 0 | 5.44 | 6.28 | 0 | 0 | 3.66 | 6.01 | Inf | 2.35E-08 | Inf | 7.20E-07 | 805 |
| Cluster-40555.225691 | 0 | 0 | 3.84 | 4.19 | 0.19 | 0 | 3.49 | 4.13 | Inf | 3.83E-11 | 5.56 | 1.39E-08 | 1327 |
| Cluster-40555.226006 | 0 | 0 | 2.61 | 1.84 | 0 | 0 | 1.24 | 1.19 | Inf | 1.82E-04 | Inf | 1.23E-02 | 1071 |
| Cluster-40555.226037 | 0 | 0 | 1.28 | 1.39 | 0 | 0 | 1.72 | 1.48 | Inf | 2.89E-02 | Inf | 5.22E-03 | 950 |

| | | | | | | | | | | | | | |
|---|---|---|---|---|---|---|---|---|---|---|---|---|---|
| Cluster-40555.226340 | 0 | 0 | 1.48 | 1.02 | 0.36 | 0.14 | 1.64 | 2.33 | Inf | 7.62E-07 | 3.13 | 1.00E-04 | 2413 |
| Cluster-40555.226502 | 0 | 0 | 9.81 | 10.03 | 0 | 0 | 5.22 | 6.08 | Inf | 8.05E-25 | Inf | 1.36E-15 | 1263 |
| Cluster-40555.226518 | 0 | 0 | 1.79 | 1.85 | 0.04 | 0.37 | 3.49 | 4.01 | Inf | 5.45E-10 | 4.20 | 1.01E-11 | 2366 |
| Cluster-40555.226586 | 0 | 0 | 15.86 | 14.08 | 0 | 0.25 | 11.26 | 3.95 | Inf | 6.83E-29 | 5.93 | 1.88E-02 | 1054 |
| Cluster-40555.226644 | 0 | 0 | 0.89 | 1.12 | 0.13 | 0 | 1.17 | 1.27 | Inf | 1.72E-02 | 4.19 | 4.33E-02 | 1237 |
| Cluster-40555.226876 | 0 | 0 | 1.77 | 1.59 | 0 | 0 | 1.97 | 1.95 | Inf | 6.37E-03 | Inf | 9.33E-04 | 958 |
| Cluster-40555.227101 | 0 | 0 | 34.09 | 28.77 | 2.98 | 2.68 | 23.93 | 37.16 | Inf | 8.70E-06 | 3.56 | 9.51E-03 | 328 |
| Cluster-40555.227177 | 0 | 0 | 1.48 | 1.55 | 0.2 | 0.15 | 3.5 | 1.79 | Inf | 7.56E-04 | 4.03 | 2.98E-03 | 1259 |
| Cluster-40555.227288 | 0 | 0 | 2 | 1.21 | 0.05 | 0.29 | 2.8 | 2.32 | Inf | 3.83E-08 | 3.95 | 9.38E-08 | 2277 |
| Cluster-40555.227383 | 0 | 0 | 2.6 | 3.41 | 0 | 0 | 5.93 | 4.23 | Inf | 4.45E-16 | Inf | 2.60E-26 | 2313 |
| Cluster-40555.227512 | 0 | 0 | 4.7 | 4.24 | 0 | 0.39 | 6.84 | 8.24 | Inf | 8.58E-03 | 5.23 | 5.99E-04 | 512 |
| Cluster-40555.227862 | 0 | 0 | 0.53 | 0.63 | 0 | 0.01 | 0.69 | 0.79 | Inf | 1.43E-03 | 6.17 | 3.25E-04 | 2635 |
| Cluster-40555.228022 | 0 | 0 | 0.95 | 0.86 | 0.3 | 0.49 | 1.98 | 1.89 | Inf | 9.22E-08 | 2.36 | 1.69E-04 | 3572 |
| Cluster-40555.228057 | 0 | 0 | 1.61 | 1.55 | 0 | 0 | 1.8 | 0.77 | Inf | 2.75E-05 | Inf | 2.44E-03 | 1557 |
| Cluster-40555.228137 | 0 | 0 | 0.95 | 1.3 | 0 | 0 | 0.81 | 0.86 | Inf | 3.65E-03 | Inf | 1.71E-02 | 1361 |
| Cluster-40555.228396 | 0 | 0 | 2.53 | 1.77 | 0 | 0 | 3.38 | 3.96 | Inf | 1.40E-02 | Inf | 6.77E-05 | 748 |
| Cluster-40555.228657 | 0 | 0 | 2.07 | 3.77 | 0 | 0 | 4.84 | 6.05 | Inf | 7.42E-04 | Inf | 1.01E-07 | 767 |
| Cluster-40555.228753 | 0 | 0 | 3.06 | 5.11 | 0 | 0 | 3.24 | 4.99 | Inf | 1.62E-02 | Inf | 1.41E-02 | 502 |
| Cluster-40555.229009 | 0 | 0 | 0.93 | 1.34 | 0 | 0 | 0.98 | 0.59 | Inf | 2.77E-04 | Inf | 3.82E-03 | 1715 |
| Cluster-40555.229257 | 0 | 0 | 1.32 | 1 | 0 | 0 | 2.04 | 2.74 | Inf | 1.31E-06 | Inf | 5.80E-15 | 2510 |
| Cluster-40555.229273 | 0 | 0 | 5.73 | 2.74 | 0.26 | 0.1 | 3.3 | 2.61 | Inf | 1.43E-06 | 4.16 | 6.11E-07 | 1741 |
| Cluster-40555.229433 | 0 | 0 | 1.67 | 1.75 | 0 | 0.09 | 1.17 | 1.11 | Inf | 5.05E-08 | 4.83 | 3.37E-04 | 2068 |
| Cluster-40555.229493 | 0 | 0 | 2.71 | 3.29 | 0.51 | 0.03 | 2.35 | 1.78 | Inf | 4.37E-15 | 3.07 | 2.77E-04 | 2184 |
| Cluster-40555.229653 | 0 | 0 | 1.76 | 1.85 | 0 | 0 | 1.63 | 1.21 | Inf | 6.69E-06 | Inf | 5.08E-05 | 1526 |
| Cluster-40555.229661 | 0 | 0 | 3.64 | 2.21 | 0.13 | 0 | 3.55 | 3.82 | Inf | 7.37E-12 | 5.79 | 1.72E-12 | 1875 |
| Cluster-40555.229732 | 0 | 0 | 1.08 | 1.56 | 0.11 | 0 | 2.03 | 1.5 | Inf | 5.83E-04 | 4.99 | 3.54E-04 | 1427 |
| Cluster-40555.229769 | 0 | 0 | 1.51 | 1.06 | 0.46 | 0.42 | 2.08 | 1.23 | Inf | 5.82E-07 | 1.98 | 3.10E-02 | 2392 |
| Cluster-40555.229831 | 0 | 0 | 2.01 | 1.55 | 0.79 | 0.41 | 4.46 | 3.78 | Inf | 1.14E-06 | 2.86 | 1.70E-05 | 1725 |
| Cluster-40555.229997 | 0 | 0 | 1.11 | 0.93 | 0 | 0 | 2.1 | 1.52 | Inf | 1.30E-04 | Inf | 2.79E-09 | 2069 |
| Cluster-40555.230104 | 0 | 0 | 2.19 | 2.61 | 0.06 | 0 | 1.8 | 2.82 | Inf | 1.04E-04 | 6.35 | 3.17E-04 | 1022 |
| Cluster-40555.230178 | 0 | 0 | 0.42 | 0.54 | 0 | 0 | 0.6 | 0.36 | Inf | 1.28E-02 | Inf | 7.58E-03 | 2418 |
| Cluster-40555.230221 | 0 | 0 | 13.65 | 16.01 | 0 | 0 | 16.89 | 10.21 | Inf | 1.43E-03 | Inf | 3.85E-03 | 353 |

| | | | | | | | | | | | | | |
|---|---|---|---|---|---|---|---|---|---|---|---|---|---|
| Cluster-40555.230289 | 0 | 0 | 1.14 | 0.94 | 0 | 0 | 0.65 | 0.44 | Inf | 2.02E-04 | Inf | 1.48E-02 | 1971 |
| Cluster-40555.230360 | 0 | 0 | 6.18 | 9.15 | 0 | 0.35 | 6.63 | 7.15 | Inf | 3.20E-05 | 5.14 | 1.10E-03 | 520 |
| Cluster-40555.230397 | 0 | 0 | 0.58 | 0.28 | 0.23 | 0 | 1.31 | 1.2 | Inf | 1.65E-02 | 3.54 | 4.74E-04 | 2654 |
| Cluster-40555.230423 | 0 | 0 | 5.09 | 3.56 | 0 | 0 | 3.7 | 4.68 | Inf | 2.24E-06 | Inf | 7.67E-07 | 846 |
| Cluster-40555.230428 | 0 | 0 | 3.92 | 1.99 | 0 | 0 | 1.62 | 2.19 | Inf | 2.38E-04 | Inf | 4.27E-03 | 859 |
| Cluster-40555.230476 | 0 | 0 | 2.42 | 0.88 | 0.33 | 0 | 3.16 | 3.19 | Inf | 1.24E-03 | 4.38 | 2.06E-11 | 2649 |
| Cluster-40555.230647 | 0 | 0 | 2.46 | 2.18 | 0 | 0.09 | 2.33 | 1.96 | Inf | 1.22E-05 | 5.39 | 1.58E-04 | 1208 |
| Cluster-40555.230865 | 0 | 0 | 0.62 | 0.24 | 0 | 0 | 0.54 | 0.55 | Inf | 9.52E-03 | Inf | 2.33E-04 | 3103 |
| Cluster-40555.230888 | 0 | 0 | 0.71 | 1.01 | 0 | 0 | 1.99 | 2.46 | Inf | 1.16E-02 | Inf | 1.01E-07 | 1481 |
| Cluster-40555.231030 | 0 | 0 | 1.2 | 0.73 | 0.48 | 0.64 | 2.49 | 3.18 | Inf | 1.54E-02 | 2.41 | 1.40E-02 | 1341 |
| Cluster-40555.231051 | 0 | 0 | 4.65 | 6.44 | 0 | 0 | 6.25 | 4.83 | Inf | 1.32E-02 | Inf | 1.25E-02 | 446 |
| Cluster-40555.231071 | 0 | 0 | 2.32 | 2.66 | 0 | 0 | 2.77 | 2.2 | Inf | 1.06E-03 | Inf | 4.78E-04 | 848 |
| Cluster-40555.231124 | 0 | 0 | 2.63 | 1.77 | 0 | 0.09 | 3 | 3.87 | Inf | 2.03E-08 | 6.15 | 8.81E-12 | 1764 |
| Cluster-40555.231182 | 0 | 0 | 1.4 | 1.81 | 1.37 | 0.49 | 6.53 | 3.69 | Inf | 3.09E-03 | 2.53 | 1.31E-02 | 1053 |
| Cluster-40555.231202 | 0 | 0 | 6.25 | 3.05 | 0.13 | 0 | 15.43 | 14.05 | Inf | 3.49E-06 | 8.09 | 1.80E-26 | 1062 |
| Cluster-40555.231962 | 0 | 0 | 0.34 | 0.76 | 0 | 0 | 0.46 | 0.97 | Inf | 4.58E-02 | Inf | 5.86E-03 | 1741 |
| Cluster-40555.232154 | 0 | 0 | 0.46 | 0.47 | 0.29 | 0 | 2.05 | 1.42 | Inf | 8.87E-03 | 3.69 | 1.17E-05 | 2672 |
| Cluster-40555.232200 | 0 | 0 | 1.22 | 1.15 | 0 | 0.05 | 0.7 | 0.51 | Inf | 6.48E-07 | 4.85 | 6.67E-03 | 2519 |
| Cluster-40555.232229 | 0 | 0 | 5.55 | 5.3 | 0 | 0 | 6.75 | 7.92 | Inf | 9.66E-06 | Inf | 1.87E-08 | 675 |
| Cluster-40555.232267 | 0 | 0 | 4.28 | 3.57 | 0 | 0.04 | 2.61 | 2.56 | Inf | 7.07E-05 | Inf | 1.85E-03 | 750 |
| Cluster-40555.232383 | 0 | 0 | 2.56 | 1.47 | 0 | 0 | 1.11 | 1.51 | Inf | 5.62E-07 | Inf | 4.69E-05 | 1633 |
| Cluster-40555.232480 | 0 | 0 | 1.56 | 1.23 | 0.14 | 0.21 | 1.36 | 1.96 | Inf | 6.83E-05 | 3.31 | 4.92E-03 | 1636 |
| Cluster-40555.232571 | 0 | 0 | 3.86 | 5.01 | 0.4 | 0.38 | 3.15 | 4.39 | Inf | 1.45E-04 | 3.42 | 4.08E-02 | 660 |
| Cluster-40555.232722 | 0 | 0 | 0.98 | 0.89 | 0 | 0 | 1.97 | 1.24 | Inf | 1.32E-02 | Inf | 6.14E-05 | 1370 |
| Cluster-40555.232888 | 0 | 0 | 0.94 | 0.79 | 0 | 0 | 0.42 | 0.66 | Inf | 1.92E-03 | Inf | 2.76E-02 | 1855 |
| Cluster-40555.232980 | 0 | 0 | 2.22 | 1.89 | 0 | 0 | 0.54 | 0.87 | Inf | 2.33E-12 | Inf | 1.31E-04 | 2603 |
| Cluster-40555.233084 | 0 | 0 | 2.61 | 0.78 | 0 | 0 | 1.86 | 2.51 | Inf | 7.78E-03 | Inf | 3.68E-15 | 2748 |
| Cluster-40555.233087 | 0 | 0 | 2.77 | 4.67 | 0.53 | 0 | 2.31 | 1.61 | Inf | 1.62E-09 | 3.00 | 1.11E-03 | 2035 |
| Cluster-40555.233148 | 0 | 0 | 3.75 | 2.57 | 0.47 | 0.44 | 3.1 | 4.12 | Inf | 4.34E-19 | 3.03 | 6.10E-08 | 2676 |
| Cluster-40555.233282 | 0 | 0 | 2.72 | 2.55 | 0.42 | 0.22 | 1.47 | 2.6 | Inf | 9.54E-08 | 2.79 | 3.52E-02 | 1399 |
| Cluster-40555.233380 | 0 | 0 | 0.97 | 0.8 | 0.12 | 0 | 1.36 | 1.39 | Inf | 5.21E-05 | 4.57 | 1.55E-05 | 2495 |
| Cluster-40555.233429 | 0 | 0 | 3.93 | 3.72 | 0 | 0 | 0.33 | 0.92 | Inf | 6.03E-19 | Inf | 4.57E-02 | 2202 |

| | | | | | | | | | | | | | |
|---|---|---|---|---|---|---|---|---|---|---|---|---|---|
| Cluster-40555.233534 | 0 | 0 | 0.54 | 0.31 | 0 | 0 | 1.27 | 0.82 | Inf | 4.23E-02 | Inf | 3.93E-06 | 2327 |
| Cluster-40555.233605 | 0 | 0 | 2.39 | 2.17 | 0 | 0 | 1.12 | 1.53 | Inf | 1.45E-05 | Inf | 1.80E-03 | 1215 |
| Cluster-40555.233665 | 0 | 0 | 3 | 3.51 | 0 | 0.07 | 1.87 | 2.86 | Inf | 9.72E-06 | 6.04 | 8.28E-04 | 939 |
| Cluster-40555.233777 | 0 | 0 | 0.48 | 0.33 | 0.2 | 0.14 | 1.36 | 1.04 | Inf | 4.51E-02 | 2.91 | 7.99E-03 | 2386 |
| Cluster-40555.233892 | 0 | 0 | 1.38 | 0.89 | 0 | 0 | 2.78 | 3.73 | Inf | 2.45E-02 | Inf | 5.96E-08 | 1122 |
| Cluster-40555.233997 | 0 | 0 | 1.74 | 1.84 | 0 | 0 | 4.4 | 3.57 | Inf | 4.64E-21 | Inf | 3.96E-40 | 4891 |
| Cluster-40555.234102 | 0 | 0 | 12.06 | 15.62 | 0 | 0.13 | 11.44 | 14.33 | Inf | 1.01E-13 | 7.61 | 3.06E-12 | 638 |
| Cluster-40555.234784 | 0 | 0 | 4.7 | 4.08 | 0 | 0 | 3.92 | 3.54 | Inf | 1.42E-03 | Inf | 3.71E-03 | 582 |
| Cluster-40555.234976 | 0 | 0 | 0.35 | 0.9 | 0.13 | 0 | 1.8 | 1.96 | Inf | 2.04E-02 | 5.19 | 2.01E-06 | 1937 |
| Cluster-40555.234986 | 0 | 0 | 0.38 | 0.71 | 0.98 | 0.93 | 4.05 | 3.86 | Inf | 3.67E-04 | 2.11 | 1.91E-05 | 3145 |
| Cluster-40555.235109 | 0 | 0 | 2.01 | 2.98 | 0 | 0 | 2.92 | 1.92 | Inf | 2.72E-02 | Inf | 2.90E-02 | 620 |
| Cluster-40555.235200 | 0 | 0 | 0.33 | 0.94 | 0.31 | 0 | 2.56 | 3.61 | Inf | 2.04E-02 | 4.40 | 9.00E-08 | 1881 |
| Cluster-40555.235203 | 0 | 0 | 2.79 | 1.58 | 0.05 | 0.15 | 2.8 | 2.15 | Inf | 5.71E-08 | 4.67 | 1.12E-06 | 1708 |
| Cluster-40555.235378 | 0 | 0 | 12.29 | 12.01 | 2.84 | 3.95 | 17.31 | 14.12 | Inf | 2.20E-31 | 2.26 | 1.35E-07 | 1330 |
| Cluster-40555.235441 | 0 | 0 | 1.07 | 0.46 | 0 | 0.05 | 1.53 | 1.21 | Inf | 9.76E-04 | 6.00 | 2.64E-07 | 2516 |
| Cluster-40555.235472 | 0 | 0 | 9.47 | 8.25 | 0.31 | 0.16 | 7.11 | 7.06 | Inf | 7.98E-14 | 4.99 | 3.02E-08 | 868 |
| Cluster-40555.235615 | 0 | 0 | 1.62 | 1.82 | 0.17 | 0.11 | 1.62 | 1.02 | Inf | 6.44E-05 | 3.45 | 3.81E-02 | 1379 |
| Cluster-40555.235896 | 0 | 0 | 0.92 | 1.59 | 0.19 | 0 | 1.93 | 1.56 | Inf | 7.29E-06 | 4.36 | 2.42E-05 | 2034 |
| Cluster-40555.235900 | 0 | 0 | 2.13 | 2.49 | 0 | 0 | 0.69 | 1.18 | Inf | 9.13E-07 | Inf | 8.35E-03 | 1381 |
| Cluster-40555.236332 | 0 | 0 | 2.99 | 4.41 | 0 | 0 | 8.57 | 9.66 | Inf | 5.83E-04 | Inf | 1.86E-10 | 674 |
| Cluster-40555.236732 | 0 | 0 | 35.74 | 20.74 | 0.33 | 0.35 | 22.78 | 29.37 | Inf | 1.16E-09 | 6.30 | 1.35E-08 | 410 |
| Cluster-40555.237193 | 0 | 0 | 1.39 | 0.87 | 0 | 0 | 0.65 | 0.77 | Inf | 2.02E-05 | Inf | 7.71E-04 | 2168 |
| Cluster-40555.237216 | 0 | 0 | 4.15 | 2.85 | 0 | 0 | 0.86 | 1.26 | Inf | 9.02E-07 | Inf | 3.89E-02 | 1022 |
| Cluster-40555.237236 | 0 | 0 | 6.17 | 10.65 | 0.57 | 0 | 16.58 | 16.31 | Inf | 3.71E-08 | 6.00 | 6.20E-19 | 854 |
| Cluster-40555.237260 | 0 | 0 | 3.44 | 3.26 | 0 | 0.18 | 2.72 | 2.7 | Inf | 5.26E-06 | 4.68 | 1.06E-03 | 952 |
| Cluster-40555.237442 | 0 | 0 | 8.02 | 7.49 | 0 | 0 | 5.27 | 6.39 | Inf | 1.29E-07 | Inf | 4.28E-06 | 640 |
| Cluster-40555.237552 | 0 | 0 | 0.72 | 1.36 | 0 | 0 | 2.26 | 1.45 | Inf | 4.05E-05 | Inf | 5.54E-10 | 2149 |
| Cluster-40555.237655 | 0 | 0 | 8.25 | 12.37 | 0 | 0 | 3.2 | 5.99 | Inf | 3.20E-05 | Inf | 2.74E-02 | 454 |
| Cluster-40555.237667 | 0 | 0 | 2.79 | 2.55 | 0 | 0 | 0.8 | 0.9 | Inf | 3.26E-08 | Inf | 8.59E-03 | 1446 |
| Cluster-40555.237673 | 0 | 0 | 9.2 | 9.4 | 1.32 | 0.34 | 8.13 | 14.46 | Inf | 7.02E-21 | 3.87 | 2.78E-03 | 1146 |
| Cluster-40555.237724 | 0 | 0 | 3.02 | 3.11 | 0.21 | 0.12 | 3.27 | 2.26 | Inf | 4.13E-04 | 4.40 | 1.18E-02 | 786 |
| Cluster-40555.237837 | 0 | 0 | 3.95 | 2.18 | 0 | 0 | 1.71 | 1.68 | Inf | 8.83E-09 | Inf | 4.05E-07 | 1730 |

| | | | | | | | | | | | | | |
|---|---|---|---|---|---|---|---|---|---|---|---|---|---|
| Cluster-40555.237961 | 0 | 0 | 4.1 | 3.39 | 0 | 0 | 6.09 | 7.69 | Inf | 6.41E-10 | Inf | 5.04E-19 | 1287 |
| Cluster-40555.238018 | 0 | 0 | 1.8 | 2.5 | 0.14 | 0.37 | 2.57 | 2.28 | Inf | 8.71E-04 | 3.31 | 2.71E-02 | 942 |
| Cluster-40555.238082 | 0 | 0 | 1.76 | 1.29 | 0.16 | 0.04 | 1.27 | 1.44 | Inf | 3.73E-06 | 4.03 | 1.61E-03 | 1852 |
| Cluster-40555.239240 | 0 | 0 | 1.02 | 0.82 | 0 | 0 | 1 | 0.85 | Inf | 3.59E-04 | Inf | 9.93E-05 | 2065 |
| Cluster-40555.239471 | 0 | 0 | 0.77 | 0.27 | 0 | 0.03 | 0.55 | 0.65 | Inf | 2.60E-02 | 5.57 | 1.24E-02 | 2169 |
| Cluster-40555.239746 | 0 | 0 | 2.13 | 1.5 | 0 | 0 | 1.21 | 1.58 | Inf | 1.40E-05 | Inf | 9.47E-05 | 1474 |
| Cluster-40555.239907 | 0 | 0 | 1.31 | 0.97 | 0 | 0.12 | 1.44 | 1.28 | Inf | 1.80E-03 | 4.53 | 2.86E-03 | 1502 |
| Cluster-40555.239970 | 0 | 0 | 5.43 | 5.84 | 0 | 0.14 | 4.27 | 4.84 | Inf | 5.23E-08 | 5.66 | 1.33E-05 | 807 |
| Cluster-40555.240057 | 0 | 0 | 5.5 | 5.04 | 0 | 0 | 2.49 | 2.42 | Inf | 7.41E-07 | Inf | 2.62E-03 | 762 |
| Cluster-40555.240197 | 0 | 0 | 3.78 | 4.42 | 0 | 0 | 1.73 | 1.9 | Inf | 1.18E-04 | Inf | 3.94E-02 | 703 |
| Cluster-40555.240592 | 0 | 0 | 1.5 | 2.01 | 0.61 | 0 | 1.62 | 1.79 | Inf | 3.78E-06 | 2.56 | 4.30E-02 | 1597 |
| Cluster-40555.240599 | 0 | 0 | 0.32 | 0.32 | 0 | 0 | 1.36 | 1.21 | Inf | 4.24E-02 | Inf | 1.63E-09 | 2869 |
| Cluster-40555.240609 | 0 | 0 | 2.4 | 3.34 | 0.1 | 0.61 | 4.29 | 6.28 | Inf | 1.35E-03 | 3.93 | 4.01E-04 | 749 |
| Cluster-40555.240638 | 0 | 0 | 3.25 | 5.26 | 1.32 | 1.66 | 6.74 | 6.15 | Inf | 2.16E-08 | 2.16 | 4.02E-03 | 1003 |
| Cluster-40555.241205 | 0 | 0 | 1.75 | 2.48 | 0 | 0 | 2.86 | 2.83 | Inf | 1.59E-03 | Inf | 3.48E-05 | 911 |
| Cluster-40555.241327 | 0 | 0 | 6.09 | 3.78 | 0.29 | 0 | 8.99 | 6.95 | Inf | 4.96E-02 | 5.88 | 5.91E-03 | 433 |
| Cluster-40555.241612 | 0 | 0 | 3.41 | 1.92 | 0 | 0.14 | 1.45 | 2.76 | Inf | 4.71E-06 | 4.72 | 4.46E-03 | 1168 |
| Cluster-40555.241679 | 0 | 0 | 4.45 | 2.87 | 0 | 0 | 0.75 | 2.23 | Inf | 2.03E-09 | Inf | 4.98E-02 | 1268 |
| Cluster-40555.241683 | 0 | 0 | 2.66 | 3.98 | 0.5 | 0.61 | 6.62 | 4.89 | Inf | 6.55E-06 | 3.48 | 4.44E-05 | 940 |
| Cluster-40555.242052 | 0 | 0 | 2.68 | 2.52 | 0 | 0 | 1.38 | 1.88 | Inf | 5.26E-06 | Inf | 3.81E-04 | 1161 |
| Cluster-40555.242167 | 0 | 0 | 1.38 | 2.47 | 0.43 | 0.58 | 4.59 | 6.04 | Inf | 3.75E-02 | 3.45 | 3.81E-03 | 695 |
| Cluster-40555.242303 | 0 | 0 | 2.1 | 3.14 | 0 | 0 | 1.56 | 1.5 | Inf | 5.83E-04 | Inf | 2.03E-02 | 839 |
| Cluster-40555.242389 | 0 | 0 | 0.26 | 0.31 | 0 | 0 | 0.41 | 0.53 | Inf | 1.96E-02 | Inf | 1.34E-04 | 3727 |
| Cluster-40555.242654 | 0 | 0 | 2.55 | 2.75 | 0 | 0.16 | 1.9 | 1.79 | Inf | 1.42E-08 | 4.56 | 2.06E-04 | 1499 |
| Cluster-40555.243068 | 0 | 0 | 8.48 | 5.31 | 0 | 0 | 8.32 | 7.46 | Inf | 1.15E-02 | Inf | 3.14E-03 | 420 |
| Cluster-40555.243269 | 0 | 0 | 1.09 | 0.6 | 0 | 0 | 1.88 | 3.28 | Inf | 1.49E-02 | Inf | 3.47E-06 | 1518 |
| Cluster-40555.243292 | 0 | 0 | 5.91 | 5.8 | 0 | 0 | 2.48 | 3.1 | Inf | 1.05E-09 | Inf | 5.63E-05 | 897 |
| Cluster-40555.243353 | 0 | 0 | 2.84 | 2.1 | 0 | 0 | 5.03 | 3.78 | Inf | 5.27E-11 | Inf | 1.81E-20 | 2003 |
| Cluster-40555.243520 | 0 | 0 | 0.52 | 1.14 | 0 | 0 | 1.53 | 1.11 | Inf | 1.41E-03 | Inf | 3.25E-06 | 1916 |
| Cluster-40555.243981 | 0 | 0 | 2.61 | 2.66 | 0 | 0 | 2.65 | 2.11 | Inf | 1.46E-06 | Inf | 1.93E-06 | 1222 |
| Cluster-40555.244168 | 0 | 0 | 6 | 2.39 | 0 | 0 | 4.28 | 3.84 | Inf | 3.90E-03 | Inf | 7.92E-04 | 610 |
| Cluster-40555.244224 | 0 | 0 | 1.76 | 1.85 | 0 | 0 | 0.65 | 1.41 | Inf | 1.49E-04 | Inf | 8.24E-03 | 1243 |

| | | | | | | | | | | | | | |
|---|---|---|---|---|---|---|---|---|---|---|---|---|---|
| Cluster-40555.244880 | 0 | 0 | 3.67 | 4.97 | 0 | 0 | 3.36 | 2.59 | Inf | 6.43E-04 | Inf | 8.84E-03 | 613 |
| Cluster-40555.245223 | 0 | 0 | 0.9 | 0.55 | 0 | 0 | 0.44 | 0.52 | Inf | 7.76E-04 | Inf | 7.22E-03 | 2384 |
| Cluster-40555.245370 | 0 | 0 | 2.41 | 1.6 | 0 | 0 | 1.86 | 1.88 | Inf | 2.68E-03 | Inf | 2.19E-03 | 929 |
| Cluster-40555.245725 | 0 | 0 | 0.73 | 0.39 | 0.1 | 0 | 0.9 | 0.96 | Inf | 1.70E-02 | 4.34 | 4.19E-03 | 2112 |
| Cluster-40555.245778 | 0 | 0 | 1.48 | 4.46 | 0 | 0.36 | 2.14 | 4.59 | Inf | 1.74E-02 | 4.25 | 2.90E-02 | 945 |
| Cluster-40555.245847 | 0 | 0 | 1.93 | 2.99 | 0 | 0 | 1.29 | 0.52 | Inf | 1.54E-08 | Inf | 9.21E-03 | 1564 |
| Cluster-40555.246172 | 0 | 0 | 2.17 | 1.33 | 0.12 | 0 | 1.5 | 2.87 | Inf | 1.92E-03 | 5.33 | 5.40E-03 | 1058 |
| Cluster-40555.246279 | 0 | 0 | 6.45 | 4.55 | 0.84 | 0 | 16.33 | 11.21 | Inf | 1.40E-05 | 5.15 | 2.72E-10 | 660 |
| Cluster-40555.246854 | 0 | 0 | 1.19 | 0.89 | 0 | 0.04 | 0.89 | 1.17 | Inf | 1.33E-03 | 5.89 | 2.17E-03 | 1657 |
| Cluster-40555.247123 | 0 | 0 | 0.55 | 0.31 | 0 | 0 | 0.36 | 0.59 | Inf | 6.34E-04 | Inf | 5.48E-05 | 3985 |
| Cluster-40555.247219 | 0 | 0 | 4.47 | 4.75 | 1.63 | 1.39 | 6.89 | 5 | Inf | 1.20E-08 | 2.04 | 1.22E-02 | 978 |
| Cluster-40555.247426 | 0 | 0 | 2.18 | 3.57 | 0.68 | 0.7 | 4.55 | 6.22 | Inf | 2.15E-06 | 3.02 | 9.48E-05 | 1095 |
| Cluster-40555.247554 | 0 | 0 | 4.64 | 4.75 | 0 | 0 | 1.89 | 2.95 | Inf | 4.47E-20 | Inf | 5.66E-09 | 1942 |
| Cluster-40555.248284 | 0 | 0 | 0.37 | 0.53 | 0.3 | 0.33 | 1.11 | 1.21 | Inf | 1.18E-03 | 1.96 | 3.03E-02 | 3404 |
| Cluster-40555.248385 | 0 | 0 | 0.73 | 0.56 | 0 | 0 | 0.78 | 0.32 | Inf | 5.96E-03 | Inf | 1.32E-02 | 2100 |
| Cluster-40555.248441 | 0 | 0 | 0.69 | 0.41 | 0 | 0 | 0.7 | 0.51 | Inf | 3.71E-02 | Inf | 1.05E-02 | 1909 |
| Cluster-40555.248521 | 0 | 0 | 2.23 | 1.67 | 0 | 0 | 2.96 | 3.32 | Inf | 1.71E-05 | Inf | 6.77E-10 | 1367 |
| Cluster-40555.248590 | 0 | 0 | 2.07 | 2.22 | 0.08 | 0.01 | 2.8 | 2.69 | Inf | 6.70E-18 | 6.08 | 4.09E-18 | 3500 |
| Cluster-40555.249069 | 0 | 0 | 0.66 | 0.59 | 0 | 0 | 2.03 | 1.59 | Inf | 5.83E-11 | Inf | 4.52E-30 | 7064 |
| Cluster-40555.249334 | 0 | 0 | 0.37 | 0.33 | 0 | 0.04 | 1.9 | 1.49 | Inf | 2.01E-02 | 6.59 | 2.46E-11 | 3021 |
| Cluster-40555.249491 | 0 | 0 | 0.94 | 0.95 | 0 | 0 | 0.94 | 0.91 | Inf | 6.00E-05 | Inf | 2.47E-05 | 2313 |
| Cluster-40555.249540 | 0 | 0 | 1.63 | 1.2 | 0.8 | 0.73 | 2.6 | 2.28 | Inf | 2.48E-12 | 1.73 | 2.79E-03 | 3662 |
| Cluster-40555.250076 | 0 | 0 | 0.95 | 0.38 | 0 | 0 | 1.52 | 1.88 | Inf | 2.45E-02 | Inf | 3.28E-07 | 1750 |
| Cluster-40555.250253 | 0 | 0 | 3.2 | 5.09 | 0 | 0.22 | 2.68 | 3.68 | Inf | 2.91E-03 | 5.29 | 4.13E-02 | 563 |
| Cluster-40555.250312 | 0 | 0 | 2.69 | 3.14 | 0 | 0 | 1.92 | 1.25 | Inf | 4.03E-06 | Inf | 1.62E-03 | 1067 |
| Cluster-40555.250476 | 0 | 0 | 2.68 | 2.66 | 0 | 0.26 | 4.63 | 6.62 | Inf | 2.41E-05 | 5.49 | 2.78E-09 | 1036 |
| Cluster-40555.250602 | 0 | 0 | 0.37 | 0.4 | 0 | 0 | 0.46 | 0.9 | Inf | 3.28E-02 | Inf | 1.17E-03 | 2606 |
| Cluster-40555.250700 | 0 | 0 | 1.36 | 1.13 | 0.04 | 0 | 1.44 | 1.05 | Inf | 8.91E-04 | 6.10 | 1.68E-03 | 1469 |
| Cluster-40555.250912 | 0 | 0 | 1.12 | 0.99 | 0.16 | 0.17 | 1.95 | 4.02 | Inf | 2.70E-02 | 4.27 | 1.90E-02 | 1143 |
| Cluster-40555.251085 | 0 | 0 | 0.6 | 0.53 | 0 | 0 | 0.78 | 1.06 | Inf | 4.82E-02 | Inf | 1.08E-03 | 1721 |
| Cluster-40555.252530 | 0 | 0 | 0.74 | 0.56 | 0.24 | 0.44 | 1.58 | 1.34 | Inf | 1.14E-06 | 2.15 | 1.07E-03 | 4267 |
| Cluster-40555.253049 | 0 | 0 | 1.59 | 1.67 | 0 | 0 | 1.93 | 1.42 | Inf | 2.99E-07 | Inf | 4.01E-08 | 1969 |

| | | | | | | | | | | | | | |
|---|---|---|---|---|---|---|---|---|---|---|---|---|---|
| Cluster-40555.253415 | 0 | 0 | 0.87 | 0.37 | 0.05 | 0 | 0.4 | 0.36 | Inf | 4.45E-04 | 3.94 | 3.35E-02 | 3759 |
| Cluster-40555.253646 | 0 | 0 | 2.68 | 2.36 | 0 | 0 | 2.48 | 1.99 | Inf | 3.16E-06 | Inf | 4.53E-06 | 1217 |
| Cluster-40555.253948 | 0 | 0 | 0.49 | 0.52 | 0 | 0 | 0.65 | 0.32 | Inf | 1.05E-02 | Inf | 6.47E-03 | 2439 |
| Cluster-40555.254785 | 0 | 0 | 0.87 | 0.47 | 0 | 0 | 1.05 | 0.64 | Inf | 3.59E-02 | Inf | 3.82E-03 | 1614 |
| Cluster-40555.255669 | 0 | 0 | 1.26 | 1.41 | 0 | 0 | 1.77 | 2.27 | Inf | 4.49E-10 | Inf | 4.05E-16 | 3134 |
| Cluster-40555.256157 | 0 | 0 | 0.32 | 0.47 | 0 | 0.07 | 1.47 | 1.14 | Inf | 1.78E-02 | 5.06 | 1.17E-06 | 2715 |
| Cluster-40555.256228 | 0 | 0 | 3.01 | 2.45 | 0.23 | 0 | 2.72 | 2.06 | Inf | 9.81E-15 | 4.55 | 1.49E-08 | 2370 |
| Cluster-40555.256676 | 0 | 0 | 0.44 | 0.36 | 0 | 0.03 | 0.89 | 0.86 | Inf | 5.22E-03 | 5.79 | 3.39E-06 | 3318 |
| Cluster-40555.256823 | 0 | 0 | 13.05 | 13.42 | 0.12 | 0 | 11.37 | 9.59 | Inf | 1.31E-14 | 7.67 | 2.42E-11 | 687 |
| Cluster-40555.256913 | 0.02 | 0 | 1.77 | 1 | 0 | 0 | 1.14 | 1.31 | Inf | 5.36E-04 | Inf | 5.46E-04 | 1432 |
| Cluster-40555.256943 | 0 | 0 | 16.54 | 14.5 | 0.21 | 0 | 7.41 | 9.52 | Inf | 2.83E-12 | 6.87 | 2.55E-06 | 567 |
| Cluster-40555.256954 | 0 | 0 | 3.25 | 2.66 | 0.06 | 0 | 4.28 | 3.87 | Inf | 1.35E-05 | 7.13 | 1.10E-07 | 1005 |
| Cluster-40555.256955 | 0 | 0 | 0.36 | 0.51 | 0 | 0 | 0.4 | 0.64 | Inf | 4.13E-02 | Inf | 7.08E-03 | 2230 |
| Cluster-40555.257378 | 0 | 0 | 1 | 0.76 | 0.29 | 0.67 | 1.64 | 1.69 | Inf | 1.77E-06 | 1.86 | 1.32E-02 | 3149 |
| Cluster-40555.258104 | 0 | 0 | 1.06 | 0.84 | 0 | 0.42 | 2.52 | 1.82 | Inf | 3.97E-02 | 3.35 | 8.18E-03 | 1179 |
| Cluster-40555.258145 | 0 | 0 | 0.38 | 0.32 | 0.2 | 0.29 | 1 | 1 | Inf | 2.01E-02 | 2.08 | 4.97E-02 | 3078 |
| Cluster-40555.258289 | 0 | 0 | 0.91 | 0.59 | 0 | 0 | 3.63 | 2.57 | Inf | 5.56E-03 | Inf | 4.27E-14 | 1887 |
| Cluster-40555.258565 | 0 | 0 | 3.54 | 2.4 | 1.88 | 2.47 | 26.25 | 15.25 | Inf | 6.56E-03 | 3.27 | 4.10E-04 | 657 |
| Cluster-40555.258852 | 0 | 0 | 0.44 | 0.74 | 0 | 0.23 | 0.98 | 0.87 | Inf | 8.11E-03 | 2.97 | 3.74E-02 | 2120 |
| Cluster-40555.258998 | 0 | 0 | 2.92 | 1.95 | 0 | 0 | 4.08 | 3.82 | Inf | 3.61E-03 | Inf | 6.27E-06 | 795 |
| Cluster-40555.259027 | 0 | 0 | 1.67 | 1.06 | 0 | 0 | 4.48 | 2.71 | Inf | 9.60E-06 | Inf | 6.61E-12 | 1938 |
| Cluster-40555.259787 | 0.02 | 0 | 1.65 | 1.93 | 0 | 0 | 1.29 | 1.7 | Inf | 2.97E-06 | Inf | 1.19E-05 | 1590 |
| Cluster-40555.261360 | 0 | 0 | 1.86 | 2.57 | 0 | 0.28 | 2.92 | 2.95 | Inf | 9.06E-08 | 4.33 | 8.57E-07 | 1595 |
| Cluster-40555.261548 | 0 | 0 | 5.95 | 4.6 | 0 | 0.27 | 4.01 | 6.11 | Inf | 7.93E-21 | 5.21 | 8.95E-08 | 1842 |
| Cluster-40555.261763 | 0 | 0 | 3.81 | 1.73 | 0 | 0 | 1.78 | 1.32 | Inf | 1.02E-04 | Inf | 4.81E-04 | 1196 |
| Cluster-40555.262548 | 0 | 0 | 1.2 | 1.05 | 0.22 | 0.23 | 2.06 | 2.46 | Inf | 2.70E-02 | 3.33 | 1.58E-02 | 1083 |
| Cluster-40555.262911 | 0 | 0 | 0.51 | 0.43 | 0 | 0 | 0.58 | 0.85 | Inf | 1.27E-02 | Inf | 1.58E-04 | 2532 |
| Cluster-40555.264091 | 0 | 0 | 0.25 | 0.63 | 0 | 0 | 0.58 | 0.24 | Inf | 3.12E-02 | Inf | 4.13E-02 | 2224 |
| Cluster-40555.264243 | 0 | 0 | 2.88 | 2.87 | 0 | 0 | 4.68 | 5.25 | Inf | 9.88E-04 | Inf | 3.97E-07 | 774 |
| Cluster-40555.266871 | 0 | 0 | 2.82 | 2.43 | 0 | 0 | 3.63 | 3.78 | Inf | 4.10E-02 | Inf | 3.10E-03 | 588 |
| Cluster-40555.266872 | 0 | 0 | 1.83 | 0.87 | 0 | 0 | 0.46 | 0.8 | Inf | 1.70E-04 | Inf | 1.96E-02 | 1699 |
| Cluster-40555.267023 | 0 | 0 | 0.57 | 0.68 | 0.33 | 0.27 | 1.66 | 1.14 | Inf | 1.93E-03 | 2.31 | 1.92E-02 | 2436 |

| | | | | | | | | | | | | | |
|---|---|---|---|---|---|---|---|---|---|---|---|---|---|
| Cluster-40555.267085 | 0 | 0 | 2.01 | 3.12 | 0.05 | 0 | 0.5 | 0.91 | Inf | 3.51E-11 | 5.12 | 4.07E-03 | 2325 |
| Cluster-40555.267155 | 0 | 0 | 0.97 | 1 | 0 | 0 | 2.73 | 2.03 | Inf | 1.26E-04 | Inf | 3.19E-12 | 2078 |
| Cluster-40555.267242 | 0 | 0 | 1.98 | 3.11 | 0.06 | 0.06 | 3.89 | 3.07 | Inf | 1.87E-05 | 5.95 | 1.42E-06 | 1084 |
| Cluster-40555.269550 | 0 | 0 | 1.12 | 0.87 | 0.57 | 0 | 1.77 | 1.25 | Inf | 2.49E-06 | 2.55 | 3.69E-03 | 2759 |
| Cluster-40555.270583 | 0 | 0 | 2.33 | 3.9 | 0 | 0 | 7.91 | 6.27 | Inf | 3.92E-03 | Inf | 1.53E-07 | 644 |
| Cluster-40555.270812 | 0 | 0 | 1.13 | 1.16 | 0 | 0 | 1.11 | 1.27 | Inf | 2.45E-02 | Inf | 1.21E-02 | 1081 |
| Cluster-40555.271455 | 0 | 0 | 1.11 | 1.75 | 0.06 | 0 | 1.62 | 0.73 | Inf | 5.11E-03 | 5.43 | 4.41E-02 | 1081 |
| Cluster-40555.271687 | 0 | 0 | 0.62 | 0.28 | 0.1 | 0 | 0.7 | 0.65 | Inf | 3.61E-03 | 3.95 | 3.49E-03 | 3189 |
| Cluster-40555.271791 | 0 | 0 | 0.62 | 0.34 | 0 | 0 | 1.15 | 0.4 | Inf | 3.59E-02 | Inf | 2.07E-02 | 2190 |
| Cluster-40555.272397 | 0 | 0 | 0.69 | 0.8 | 0 | 0 | 1.43 | 1.43 | Inf | 1.89E-02 | Inf | 2.93E-05 | 1555 |
| Cluster-40555.272849 | 0 | 0 | 4.8 | 6.43 | 0 | 0 | 4.99 | 2.88 | Inf | 2.33E-04 | Inf | 3.85E-03 | 561 |
| Cluster-40555.272850 | 0 | 0 | 6.41 | 5.54 | 0 | 0 | 7.37 | 5.88 | Inf | 5.61E-06 | Inf | 3.55E-07 | 651 |
| Cluster-40555.273062 | 0 | 0 | 1.62 | 1.3 | 0 | 0.19 | 2.47 | 2.61 | Inf | 9.45E-03 | 4.72 | 7.89E-04 | 1023 |
| Cluster-40555.273179 | 0 | 0 | 2.69 | 3.59 | 0 | 0 | 2.94 | 6.98 | Inf | 2.67E-04 | Inf | 3.20E-03 | 796 |
| Cluster-40555.273683 | 0 | 0 | 1.14 | 2.57 | 0 | 0 | 1.81 | 1.7 | Inf | 3.82E-03 | Inf | 4.39E-03 | 909 |
| Cluster-40555.273829 | 0 | 0 | 5.78 | 4.54 | 0 | 0 | 5.85 | 4.37 | Inf | 9.63E-06 | Inf | 3.25E-06 | 698 |
| Cluster-40555.275078 | 0 | 0 | 0.84 | 1.04 | 0.06 | 0 | 0.93 | 1.23 | Inf | 1.07E-04 | 5.61 | 1.12E-04 | 2199 |
| Cluster-40555.275265 | 0 | 0 | 1.5 | 0.7 | 0 | 0 | 0.79 | 1.12 | Inf | 1.98E-03 | Inf | 2.13E-03 | 1548 |
| Cluster-40555.275687 | 0 | 0 | 2.35 | 3.94 | 0 | 0 | 1.87 | 1.53 | Inf | 9.46E-05 | Inf | 1.24E-02 | 831 |
| Cluster-40555.275910 | 0 | 0 | 0.41 | 0.25 | 0 | 0.04 | 0.48 | 0.51 | Inf | 3.71E-02 | 4.75 | 1.16E-02 | 2914 |
| Cluster-40555.276370 | 0 | 0 | 5.07 | 6.73 | 0 | 0 | 4.82 | 7.5 | Inf | 2.00E-03 | Inf | 1.07E-03 | 487 |
| Cluster-40555.276748 | 0 | 0 | 1.74 | 2.96 | 0 | 0 | 5.14 | 2.13 | Inf | 5.29E-03 | Inf | 3.12E-03 | 758 |
| Cluster-40555.277613 | 0 | 0 | 2.32 | 2.29 | 0 | 0 | 2.64 | 2.08 | Inf | 2.41E-05 | Inf | 5.40E-06 | 1164 |
| Cluster-40555.278020 | 0 | 0 | 0.63 | 0.41 | 0.03 | 0.09 | 1.14 | 1.21 | Inf | 4.37E-02 | 4.38 | 1.23E-03 | 1920 |
| Cluster-40555.285305 | 0 | 0 | 1.1 | 0.38 | 0 | 0 | 0.71 | 0.74 | Inf | 2.38E-02 | Inf | 7.27E-03 | 1705 |
| Cluster-40555.289809 | 0 | 0 | 2 | 1.76 | 0 | 0.05 | 1.51 | 1.5 | Inf | 1.14E-04 | 5.91 | 1.90E-03 | 1233 |
| Cluster-40555.291041 | 0 | 0 | 1.54 | 1.87 | 0.01 | 0 | 1.32 | 2.05 | Inf | 1.40E-04 | Inf | 6.54E-05 | 1300 |
| Cluster-40555.295437 | 0 | 0 | 1.09 | 3.13 | 0 | 0.12 | 1.88 | 2.6 | Inf | 2.22E-02 | 5.89 | 2.17E-03 | 899 |
| Cluster-40555.295955 | 0 | 0 | 0.65 | 0.79 | 0 | 0 | 0.59 | 0.77 | Inf | 6.19E-03 | Inf | 5.08E-03 | 1878 |
| Cluster-40555.303033 | 0 | 0 | 1.16 | 1.49 | 0.21 | 0 | 1.14 | 1.14 | Inf | 1.42E-05 | 3.63 | 9.92E-03 | 1882 |
| Cluster-40555.306180 | 0 | 0 | 0.67 | 1.03 | 0 | 0 | 0.55 | 0.39 | Inf | 6.72E-05 | Inf | 7.48E-03 | 2445 |
| Cluster-40555.313063 | 0 | 0 | 2.35 | 2.15 | 0 | 0.08 | 1.46 | 1.46 | Inf | 1.09E-03 | 5.28 | 4.21E-02 | 917 |

| | | | | | | | | | | | | | |
|---|---|---|---|---|---|---|---|---|---|---|---|---|---|
| Cluster-40555.31746 | 0 | 0 | 0.55 | 0.71 | 0 | 0 | 0.83 | 1.04 | Inf | 1.28E-02 | Inf | 2.71E-04 | 1887 |
| Cluster-40555.32204 | 0.01 | 0 | 0.61 | 0.6 | 0 | 0 | 0.67 | 0.66 | Inf | 1.55E-05 | Inf | 1.13E-06 | 3817 |
| Cluster-40555.329210 | 0 | 0 | 1.87 | 1.9 | 0 | 0.1 | 3.52 | 2.67 | Inf | 1.83E-02 | 6.04 | 7.55E-04 | 787 |
| Cluster-40555.36155 | 0 | 0 | 1.14 | 0.55 | 0 | 0 | 1.71 | 1.78 | Inf | 9.12E-03 | Inf | 1.12E-06 | 1609 |
| Cluster-40555.36882 | 0 | 0 | 1.05 | 1.05 | 0 | 0 | 1.19 | 0.89 | Inf | 1.83E-02 | Inf | 1.25E-02 | 1192 |
| Cluster-40555.46776 | 0 | 0 | 1.64 | 2.28 | 0 | 0 | 1.42 | 0.82 | Inf | 1.35E-03 | Inf | 4.08E-02 | 974 |
| Cluster-40555.47003 | 0 | 0 | 1.9 | 2.07 | 0 | 0 | 2.59 | 2.28 | Inf | 6.61E-04 | Inf | 2.97E-05 | 1033 |
| Cluster-40555.49295 | 0 | 0 | 4.04 | 4.23 | 0 | 0 | 1.57 | 1.84 | Inf | 3.38E-05 | Inf | 3.32E-02 | 749 |
| Cluster-40555.49308 | 0 | 0 | 0.54 | 0.62 | 0 | 0 | 0.64 | 0.77 | Inf | 3.85E-02 | Inf | 7.22E-03 | 1774 |
| Cluster-40555.50221 | 0 | 0 | 0.78 | 0.91 | 0 | 0 | 2.07 | 2.07 | Inf | 5.62E-05 | Inf | 2.88E-13 | 2532 |
| Cluster-40555.53685 | 0 | 0 | 3.4 | 1.82 | 0 | 0 | 2.62 | 1.67 | Inf | 4.53E-04 | Inf | 1.15E-03 | 890 |
| Cluster-40555.56327 | 0 | 0 | 0.24 | 0.66 | 0 | 0 | 0.43 | 0.37 | Inf | 2.17E-02 | Inf | 6.24E-03 | 2912 |
| Cluster-40555.56331 | 0 | 0 | 4.56 | 3.19 | 0.12 | 0.42 | 2.87 | 4.63 | Inf | 8.31E-04 | 3.96 | 1.69E-02 | 659 |
| Cluster-40555.57413 | 0 | 0 | 0.84 | 0.66 | 0 | 0 | 1.09 | 1.54 | Inf | 1.27E-02 | Inf | 2.81E-05 | 1688 |
| Cluster-40555.57832 | 0 | 0 | 6.74 | 7.2 | 0.57 | 0.6 | 11.51 | 11.44 | Inf | 5.11E-12 | 4.36 | 1.62E-11 | 922 |
| Cluster-40555.61087 | 0 | 0 | 2.27 | 2.47 | 0 | 0.14 | 3.45 | 1.55 | Inf | 2.75E-04 | 5.16 | 6.54E-03 | 968 |
| Cluster-40555.61897 | 0 | 0 | 1.63 | 1.12 | 0 | 0.24 | 2.76 | 1.7 | Inf | 1.88E-02 | 4.06 | 7.22E-03 | 991 |
| Cluster-40555.62916 | 0 | 0 | 1.36 | 1.01 | 0.11 | 0 | 1.3 | 1.33 | Inf | 2.22E-02 | 4.64 | 4.20E-02 | 1085 |
| Cluster-40555.66184 | 0 | 0 | 0.63 | 1.18 | 0.12 | 0.08 | 2.46 | 1.93 | Inf | 8.41E-03 | 4.55 | 6.52E-05 | 1472 |
| Cluster-40555.66628 | 0 | 0 | 1.35 | 1.79 | 0.15 | 0.05 | 1.56 | 1.88 | Inf | 1.14E-03 | 4.14 | 1.04E-02 | 1173 |
| Cluster-40555.67360 | 0 | 0 | 3.16 | 3.32 | 0.44 | 0.7 | 7.74 | 4.55 | Inf | 5.23E-05 | 3.38 | 1.86E-04 | 862 |
| Cluster-40555.68594 | 0 | 0 | 2.22 | 3.3 | 0 | 0 | 2.47 | 2.54 | Inf | 1.75E-06 | Inf | 3.10E-06 | 1152 |
| Cluster-40555.73437 | 0 | 0 | 2.91 | 1.6 | 0 | 0 | 5.57 | 5.65 | Inf | 2.69E-02 | Inf | 1.33E-06 | 684 |
| Cluster-40555.81208 | 0 | 0 | 3.13 | 1.9 | 0.4 | 1.55 | 9.85 | 5.79 | Inf | 3.71E-02 | 3.02 | 3.32E-03 | 616 |
| Cluster-40555.81760 | 0 | 0 | 0.29 | 0.8 | 0 | 0 | 1.22 | 0.91 | Inf | 1.11E-02 | Inf | 9.02E-11 | 3803 |
| Cluster-40555.83141 | 0 | 0 | 2.64 | 2.78 | 0 | 0 | 2.71 | 2.3 | Inf | 1.17E-09 | Inf | 1.00E-09 | 1626 |
| Cluster-40555.84116 | 0 | 0 | 0.72 | 0.58 | 0.11 | 0 | 0.65 | 0.42 | Inf | 1.49E-04 | 3.31 | 4.87E-02 | 3038 |
| Cluster-40555.84240 | 0 | 0 | 4.1 | 2.38 | 0 | 0 | 2.88 | 2.53 | Inf | 7.54E-05 | Inf | 1.67E-04 | 863 |
| Cluster-40555.84347 | 0 | 0 | 4.61 | 1.57 | 0.41 | 0.43 | 7.26 | 6.61 | Inf | 2.06E-02 | 4.11 | 3.33E-05 | 723 |
| Cluster-40555.84598 | 0 | 0 | 1.02 | 0.9 | 0.15 | 0.04 | 2.45 | 2.63 | Inf | 4.73E-03 | 4.86 | 3.62E-06 | 1526 |
| Cluster-40555.84626 | 0 | 0 | 2.11 | 1.5 | 0.08 | 0 | 2.36 | 1.98 | Inf | 1.40E-02 | 5.86 | 6.85E-03 | 845 |
| Cluster-40555.85306 | 0 | 0 | 9.08 | 7.29 | 1.18 | 0 | 8.45 | 6.57 | Inf | 8.41E-06 | 3.74 | 4.99E-03 | 539 |

| | | | | | | | | | | | | | |
|---|---|---|---|---|---|---|---|---|---|---|---|---|---|
| Cluster-40555.85840 | 0 | 0 | 1.31 | 1.31 | 0 | 0 | 2.5 | 2.29 | Inf | 2.26E-06 | Inf | 1.19E-12 | 2139 |
| Cluster-40555.86123 | 0 | 0 | 4.82 | 4.88 | 0 | 0 | 1.23 | 2.26 | Inf | 9.40E-07 | Inf | 1.65E-02 | 798 |
| Cluster-40555.86638 | 0 | 0 | 5.91 | 10.09 | 0 | 0.41 | 5.95 | 9.49 | Inf | 2.27E-04 | 4.99 | 3.02E-03 | 469 |
| Cluster-40555.86905 | 0 | 0 | 2.79 | 2.33 | 0 | 0 | 3.42 | 3.99 | Inf | 2.30E-02 | Inf | 9.21E-04 | 636 |
| Cluster-40555.87138 | 0 | 0 | 1.4 | 1.3 | 0 | 0 | 1.21 | 1.61 | Inf | 1.15E-02 | Inf | 4.27E-03 | 1058 |
| Cluster-40555.87549 | 0 | 0 | 0.67 | 1.91 | 0.48 | 0 | 3.37 | 3.15 | Inf | 7.85E-03 | 3.85 | 2.90E-07 | 1877 |
| Cluster-40555.87638 | 0 | 0 | 1.15 | 1.19 | 0 | 0 | 1.26 | 0.86 | Inf | 1.83E-02 | Inf | 2.07E-02 | 1104 |
| Cluster-40555.87974 | 0 | 0 | 2.35 | 1.56 | 0.18 | 0 | 2.68 | 1.32 | Inf | 1.09E-03 | 4.53 | 8.03E-03 | 1023 |
| Cluster-40555.88052 | 0 | 0 | 0.63 | 0.27 | 0 | 0 | 0.72 | 0.51 | Inf | 4.71E-03 | Inf | 7.17E-05 | 3082 |
| Cluster-40555.88283 | 0 | 0 | 0.8 | 1.21 | 0 | 0 | 1.05 | 0.75 | Inf | 9.03E-04 | Inf | 1.35E-03 | 1713 |
| Cluster-40555.89142 | 0 | 0 | 1.07 | 1.55 | 0 | 0 | 1.63 | 0.72 | Inf | 2.81E-03 | Inf | 3.90E-03 | 1237 |
| Cluster-40555.90423 | 0 | 0 | 4.38 | 2.42 | 0 | 0.21 | 3.91 | 3.8 | Inf | 6.51E-08 | 5.36 | 1.88E-08 | 1300 |
| Cluster-40555.90767 | 0 | 0 | 1.58 | 1.47 | 0 | 0 | 2.68 | 2.46 | Inf | 1.15E-02 | Inf | 4.93E-05 | 962 |
| Cluster-40555.90799 | 0 | 0 | 2.92 | 2.51 | 0 | 0 | 0.72 | 1.36 | Inf | 2.48E-09 | Inf | 7.40E-04 | 1591 |
| Cluster-40555.91685 | 0 | 0 | 1.94 | 2.95 | 0 | 0.17 | 3.83 | 4.2 | Inf | 3.22E-06 | 5.73 | 1.18E-08 | 1232 |
| Cluster-40555.91861 | 0 | 0 | 9.11 | 8.92 | 0.04 | 0 | 3.76 | 4.04 | Inf | 1.13E-23 | 7.57 | 1.43E-10 | 1309 |
| Cluster-40555.92377 | 0 | 0 | 1.28 | 0.58 | 0 | 0.11 | 2.12 | 1.6 | Inf | 4.66E-02 | 5.13 | 1.14E-03 | 1183 |
| Cluster-40555.92711 | 0 | 0 | 2.27 | 2.04 | 0 | 0 | 1.55 | 1.15 | Inf | 4.48E-11 | Inf | 1.30E-07 | 2251 |
| Cluster-40555.93962 | 0 | 0 | 0.67 | 0.98 | 0 | 0 | 1.17 | 1.08 | Inf | 5.66E-05 | Inf | 2.46E-07 | 2558 |
| Cluster-40555.93963 | 0 | 0 | 0.74 | 0.61 | 0 | 0 | 0.73 | 0.39 | Inf | 1.27E-02 | Inf | 2.10E-02 | 1830 |
| Cluster-40555.94247 | 0 | 0 | 0.99 | 0.66 | 0.49 | 0 | 0.92 | 1.06 | Inf | 5.72E-08 | 2.11 | 3.05E-02 | 4085 |
| Cluster-40555.94592 | 0 | 0 | 0.53 | 0.63 | 0 | 0.11 | 0.98 | 0.96 | Inf | 5.25E-03 | 4.04 | 1.98E-03 | 2297 |
| Cluster-40555.94685 | 0 | 0 | 3.15 | 5 | 0 | 0 | 6.87 | 4.56 | Inf | 4.37E-06 | Inf | 4.68E-09 | 825 |
| Cluster-40555.95205 | 0 | 0 | 1.54 | 2.56 | 0 | 0 | 2.72 | 2.65 | Inf | 3.75E-02 | Inf | 5.19E-03 | 675 |
| Cluster-40555.96008 | 0 | 0 | 0.33 | 0.53 | 0 | 0.02 | 0.57 | 0.61 | Inf | 3.20E-02 | 5.63 | 9.10E-03 | 2291 |
| Cluster-40555.96059 | 0 | 0 | 1.69 | 1.37 | 0 | 0 | 1.46 | 1.23 | Inf | 9.51E-08 | Inf | 1.51E-07 | 2222 |
| Cluster-40555.96269 | 0 | 0 | 11.52 | 9.97 | 0 | 0 | 2.52 | 7.12 | Inf | 2.12E-07 | Inf | 4.57E-02 | 528 |
| Cluster-40555.97674 | 0 | 0 | 11.47 | 10.93 | 0.15 | 0 | 4.57 | 3.13 | Inf | 1.57E-09 | 5.82 | 8.11E-03 | 587 |
| Cluster-40555.98228 | 0 | 0 | 0.94 | 1.01 | 0 | 0 | 0.93 | 0.65 | Inf | 1.41E-02 | Inf | 3.41E-02 | 1312 |
| Cluster-40555.98229 | 0 | 0 | 0.68 | 0.49 | 0 | 0 | 0.89 | 0.82 | Inf | 2.77E-03 | Inf | 1.76E-05 | 2518 |
| Cluster-40555.99463 | 0 | 0 | 0.74 | 1.39 | 0 | 0 | 0.64 | 1.69 | Inf | 2.36E-04 | Inf | 1.74E-02 | 1812 |
| Cluster-40555.99544 | 0 | 0 | 0.71 | 0.51 | 0 | 0.03 | 1.29 | 1.72 | Inf | 3.37E-02 | 6.53 | 1.48E-05 | 1732 |

| | | | | | | | | | | | | | | |
|---|---|---|---|---|---|---|---|---|---|---|---|---|---|---|
| Cluster-40555.99547 | 0 | 0 | 0.7 | 1.01 | 0 | 0 | 0.88 | 1.23 | Inf | 2.03E-02 | Inf | 3.59E-03 | 1365 |
| Cluster-40555.99731 | 0 | 0 | 1.26 | 2.5 | 0 | 0 | 1.61 | 1.93 | Inf | 3.07E-05 | Inf | 1.86E-08 | 1939 |
| Cluster-74297.0 | 0 | 0 | 3.57 | 4.76 | 0 | 0 | 1.88 | 2.08 | Inf | 1.11E-04 | Inf | 2.82E-02 | 693 |
| Cluster-75360.0 | 0 | 0 | 3.44 | 4.43 | 0.07 | 0 | 2.61 | 4.11 | Inf | 3.12E-07 | 6.77 | 7.65E-06 | 961 |
| Cluster-75628.0 | 0 | 0 | 2.29 | 2.23 | 0.04 | 0.04 | 2.95 | 2.47 | Inf | 9.88E-08 | 6.28 | 1.70E-08 | 1581 |
| Cluster-79702.0 | 0 | 0 | 1.77 | 1.41 | 0 | 0.08 | 1.89 | 2.75 | Inf | 2.61E-02 | 5.81 | 3.48E-03 | 854 |
| Cluster-80091.0 | 0 | 0 | 0.54 | 0.93 | 0 | 0 | 0.9 | 0.59 | Inf | 7.12E-03 | Inf | 4.51E-03 | 1790 |
| Cluster-84106.2 | 0 | 0 | 1.05 | 0.86 | 0 | 0 | 1.29 | 1.32 | Inf | 4.57E-03 | Inf | 9.73E-05 | 1552 |
| Cluster-85051.0 | 0 | 0 | 3.47 | 3.31 | 0 | 0 | 1.18 | 1.72 | Inf | 2.38E-10 | Inf | 9.33E-05 | 1435 |
| Cluster-85371.0 | 0 | 0 | 0.6 | 0.85 | 0 | 0 | 0.49 | 0.94 | Inf | 4.93E-03 | Inf | 2.95E-03 | 1904 |
| Cluster-92982.0 | 0 | 0 | 12.69 | 8.67 | 0 | 0 | 6.66 | 5.48 | Inf | 1.13E-04 | Inf | 1.05E-02 | 435 |
| Cluster-94127.0 | 0 | 0 | 2.1 | 3.43 | 0 | 0 | 6.18 | 3.49 | Inf | 1.06E-02 | Inf | 4.41E-05 | 643 |
| Cluster-95109.0 | 0 | 0 | 0.73 | 0.69 | 0 | 0.03 | 1.15 | 0.91 | Inf | 1.77E-02 | 5.90 | 1.94E-03 | 1681 |
| Cluster-98526.0 | 0 | 0 | 0.8 | 0.81 | 0 | 0 | 1.28 | 1.01 | Inf | 1.83E-02 | Inf | 6.76E-04 | 1494 |
| Cluster-98739.0 | 0 | 0 | 11.95 | 9.01 | 0 | 0 | 9.91 | 9.7 | Inf | 9.29E-06 | Inf | 1.05E-05 | 477 |
| Cluster-99674.0 | 0 | 0 | 0.53 | 0.51 | 0.02 | 0 | 0.83 | 1.08 | Inf | 3.62E-03 | 6.76 | 7.84E-06 | 2698 |

FC: Fold change

## Table S2: Putative transcription factors from DEGs

| Gene | TF_type | subject_id | Evalue | bit_score | log2FC(B14/Y14) | Padj | log2FC(B28/Y28) | Padj |
|---|---|---|---|---|---|---|---|---|
| Cluster-40555.169774 | AP2 | LOC_Os04g55560.3 | 0.00E+00 | 1199 | 3.21 | 3.40E-15 | 2.61 | 2.27E-05 |
| Cluster-40555.209356 | BES1 | LOC_Os01g10610.1 | 0.00E+00 | 972 | 4.49 | 9.00E-07 | 2.36 | 2.98E-03 |
| Cluster-40555.146730 | bHLH | LOC_Os10g42430.1 | 0.00E+00 | 1360 | -5.72 | 3.56E-18 | 0.00 | 1.54E-07 |
| Cluster-40555.177017 | bHLH | LOC_Os03g53020.1 | 2.00E-131 | 468 | -2.06 | 7.12E-06 | -1.30 | 4.35E-02 |
| Cluster-40555.184841 | bHLH | LOC_Os09g31300.1 | 3.00E-120 | 429 | -4.61 | 2.85E-08 | -3.27 | 9.96E-08 |
| Cluster-40555.201242 | bHLH | LOC_Os10g42430.1 | 0.00E+00 | 1845 | -5.65 | 3.09E-51 | -1.91 | 1.29E-05 |
| Cluster-40555.234751 | bHLH | LOC_Os02g02480.1 | 1.00E-119 | 427 | -5.49 | 2.60E-41 | -3.13 | 7.82E-11 |
| Cluster-40555.272493 | bHLH | LOC_Os05g07120.1 | 3.00E-52 | 204 | -2.79 | 6.27E-05 | -3.26 | 2.70E-03 |
| Cluster-40555.158717 | bHLH | LOC_Os05g38140.1 | 7.00E-123 | 438 | Inf | 1.53E-17 | 3.59 | 3.25E-05 |
| Cluster-40555.160644 | bHLH | LOC_Os03g43810.1 | 3.00E-68 | 257 | 2.38 | 2.94E-02 | 2.34 | 2.70E-03 |
| Cluster-40555.182851 | bHLH | LOC_Os05g38140.1 | 1.00E-149 | 527 | Inf | 3.39E-08 | 5.05 | 1.27E-04 |
| Cluster-40555.187730 | bHLH | LOC_Os03g46860.1 | 5.00E-83 | 307 | Inf | 1.76E-02 | Inf | 2.92E-07 |
| Cluster-40555.188801 | bHLH | LOC_Os07g43530.2 | 5.00E-102 | 370 | 2.36 | 3.97E-18 | 1.41 | 5.58E-04 |
| Cluster-40555.198935 | bHLH | LOC_Os02g47660.1 | 6.00E-57 | 220 | 2.97 | 8.36E-08 | 1.55 | 9.97E-03 |
| Cluster-40555.201846 | bHLH | LOC_Os03g53020.1 | 2.00E-131 | 468 | 5.79 | 4.69E-11 | 5.74 | 2.63E-15 |
| Cluster-40555.201847 | bHLH | LOC_Os03g53020.1 | 1.00E-53 | 209 | 3.15 | 4.06E-04 | 2.22 | 9.04E-03 |
| Cluster-40555.214608 | bHLH | LOC_Os09g31300.2 | 9.00E-69 | 259 | Inf | 6.43E-13 | 4.37 | 2.66E-09 |
| Cluster-40555.226537 | bHLH | LOC_Os10g42430.1 | 0.00E+00 | 737 | 1.67 | 3.93E-02 | 4.59 | 1.81E-06 |
| Cluster-40555.230888 | bHLH | LOC_Os05g46370.1 | 4.00E-95 | 346 | Inf | 1.16E-02 | Inf | 1.01E-07 |
| Cluster-40555.239070 | bHLH | LOC_Os02g02480.1 | 1.00E-71 | 268 | 3.76 | 8.21E-03 | 3.62 | 1.67E-02 |
| Cluster-40555.97572 | bHLH | LOC_Os05g07120.1 | 1.00E-21 | 102 | 5.72 | 3.27E-04 | 3.85 | 1.53E-02 |
| Cluster-40555.171315 | bZIP | LOC_Os08g36790.1 | 3.00E-14 | 78.7 | -6.31 | 2.74E-06 | -2.52 | 1.60E-02 |
| Cluster-40555.203533 | bZIP | LOC_Os01g07880.1 | 2.00E-107 | 387 | -2.29 | 2.97E-11 | -1.97 | 3.85E-06 |
| Cluster-40555.218108 | bZIP | LOC_Os06g45140.3 | 4.00E-18 | 89.8 | -5.47 | 2.87E-04 | -5.21 | 5.15E-06 |
| Cluster-40555.178265 | bZIP | LOC_Os01g07880.1 | 6.00E-103 | 372 | 4.82 | 7.32E-10 | 3.90 | 1.11E-05 |
| Cluster-40555.179322 | bZIP | LOC_Os06g41770.1 | 1.00E-169 | 593 | Inf | 2.07E-29 | Inf | 9.34E-14 |
| Cluster-40555.195157 | bZIP | LOC_Os11g05640.1 | 1.00E-38 | 158 | 4.68 | 7.92E-18 | 1.84 | 3.80E-05 |
| Cluster-40555.219797 | bZIP | LOC_Os12g13170.2 | 0.00E+00 | 1319 | 3.93 | 6.23E-06 | 1.49 | 1.84E-02 |
| Cluster-40555.142929 | C2H2 | LOC_Os03g32230.1 | 2.00E-66 | 250 | -4.85 | 1.75E-12 | -6.26 | 3.11E-09 |

| | | | | | | | | |
|---|---|---|---|---|---|---|---|---|
| Cluster-40555.175851 | C2H2 | LOC_Os12g39400.1 | 2.00E-79 | 294 | -4.79 | 2.05E-15 | -8.93 | 2.69E-45 |
| Cluster-40555.141702 | C2H2 | LOC_Os03g32230.1 | 2.00E-66 | 250 | 1.57 | 1.06E-02 | 1.59 | 2.42E-02 |
| Cluster-40555.150134 | C2H2 | LOC_Os03g13600.1 | 6.00E-41 | 165 | 3.84 | 7.23E-04 | 3.97 | 1.03E-04 |
| Cluster-40555.180184 | C2H2 | LOC_Os12g39400.1 | 3.00E-86 | 316 | 2.58 | 5.38E-16 | 0.95 | 3.43E-02 |
| Cluster-40555.180185 | C2H2 | LOC_Os12g39400.1 | 7.00E-88 | 322 | Inf | 3.05E-62 | 4.05 | 1.43E-25 |
| Cluster-40555.180186 | C2H2 | LOC_Os12g39400.1 | 1.00E-89 | 327 | 3.69 | 3.80E-31 | 2.52 | 2.83E-13 |
| Cluster-40555.194927 | C2H2 | LOC_Os01g62190.1 | 3.00E-28 | 122 | 3.71 | 2.20E-09 | 3.85 | 3.59E-07 |
| Cluster-40555.204350 | C3H | LOC_Os01g42970.2 | 3.00E-134 | 477 | -1.30 | 4.30E-02 | -2.03 | 4.04E-03 |
| Cluster-40555.190917 | C3H | LOC_Os05g10670.1 | 1.00E-54 | 211 | Inf | 1.15E-34 | 6.61 | 4.69E-43 |
| Cluster-40555.212569 | C3H | LOC_Os02g10080.1 | 6.00E-178 | 621 | 3.66 | 1.20E-09 | 3.14 | 1.02E-07 |
| Cluster-40555.233380 | C3H | LOC_Os02g58440.1 | 1.00E-146 | 518 | Inf | 5.21E-05 | 4.57 | 1.55E-05 |
| Cluster-40555.235817 | C3H | LOC_Os04g57600.1 | 0.00E+00 | 1561 | 4.63 | 2.92E-09 | 2.62 | 5.19E-06 |
| Cluster-40555.191531 | CO-like | LOC_Os02g39710.1 | 0.00E+00 | 660 | Inf | 3.11E-17 | Inf | 3.08E-06 |
| Cluster-40555.206298 | CO-like | LOC_Os09g06464.1 | 5.00E-95 | 346 | 1.50 | 3.22E-05 | 1.78 | 2.72E-03 |
| Cluster-40555.233365 | CO-like | LOC_Os02g49880.1 | 1.00E-176 | 617 | 4.14 | 5.19E-11 | Inf | 1.53E-11 |
| Cluster-40555.179654 | DBB | LOC_Os02g39360.1 | 2.00E-141 | 499 | -2.68 | 2.97E-12 | -2.55 | 1.71E-11 |
| Cluster-40555.194971 | DBB | LOC_Os02g39360.1 | 9.00E-140 | 494 | -1.94 | 2.79E-04 | -2.76 | 4.35E-10 |
| Cluster-40555.194972 | DBB | LOC_Os04g41560.4 | 5.00E-139 | 492 | -1.66 | 1.70E-03 | -1.86 | 3.33E-03 |
| Cluster-40555.194976 | DBB | LOC_Os02g39360.1 | 9.00E-140 | 494 | Inf | 6.15E-31 | Inf | 4.19E-24 |
| Cluster-40555.199338 | DBB | LOC_Os02g39360.1 | 2.00E-141 | 499 | Inf | 8.48E-47 | 6.81 | 9.12E-34 |
| Cluster-40555.183211 | Dof | LOC_Os01g55340.1 | 2.00E-58 | 224 | -1.82 | 4.46E-05 | -1.95 | 7.35E-07 |
| Cluster-40555.188528 | Dof | LOC_Os02g49440.1 | 6.00E-127 | 451 | -4.47 | 7.19E-15 | -2.73 | 8.30E-05 |
| Cluster-40555.194651 | Dof | LOC_Os01g55340.1 | 4.00E-55 | 213 | -5.00 | 3.63E-03 | -4.98 | 1.06E-07 |
| Cluster-40555.208868 | Dof | LOC_Os09g29960.1 | 1.00E-36 | 152 | -2.50 | 3.08E-11 | -2.40 | 7.35E-05 |
| Cluster-40555.230137 | Dof | LOC_Os03g07360.1 | 0.00E+00 | 787 | -4.43 | 3.89E-06 | -4.75 | 1.22E-06 |
| Cluster-40555.168102 | Dof | LOC_Os01g55340.1 | 1.00E-58 | 224 | Inf | 4.41E-15 | 6.17 | 1.31E-13 |
| Cluster-40555.171911 | Dof | LOC_Os09g29960.1 | 1.00E-36 | 152 | Inf | 1.35E-20 | Inf | 4.07E-13 |
| Cluster-40555.161069 | ERF | LOC_Os06g47590.1 | 3.00E-138 | 490 | -4.55 | 9.77E-04 | -3.17 | 1.00E-04 |
| Cluster-40555.161377 | ERF | LOC_Os03g08470.3 | 1.00E-55 | 215 | -4.50 | 1.61E-49 | -3.32 | 7.28E-03 |
| Cluster-40555.165988 | ERF | LOC_Os03g09170.1 | 5.00E-62 | 235 | 0.00 | 4.90E-27 | -7.11 | 1.31E-15 |
| Cluster-40555.192007 | ERF | LOC_Os03g08500.1 | 3.00E-89 | 326 | -1.79 | 5.17E-13 | -1.53 | 6.89E-06 |
| Cluster-40555.175287 | ERF | LOC_Os08g45110.1 | 1.00E-89 | 326 | 2.77 | 2.43E-11 | 2.60 | 2.95E-03 |

| | | | | | | | | |
|---|---|---|---|---|---|---|---|---|
| Cluster-40555.187700 | ERF | LOC_Os05g29810.1 | 4.00E-69 | 259 | 1.84 | 2.17E-13 | 3.29 | 1.64E-02 |
| Cluster-40555.188580 | ERF | LOC_Os06g47590.1 | 2.00E-125 | 448 | Inf | 4.03E-02 | Inf | 1.97E-02 |
| Cluster-40555.201722 | ERF | LOC_Os03g09170.1 | 5.00E-62 | 235 | 5.21 | 4.25E-22 | 3.30 | 7.68E-08 |
| Cluster-40555.241796 | FAR1 | LOC_Os02g39520.1 | 0.00E+00 | 2333 | 0.00 | 7.38E-05 | -3.85 | 7.60E-07 |
| Cluster-40555.209479 | FAR1 | LOC_Os04g40060.1 | 0.00E+00 | 2532 | Inf | 1.88E-03 | Inf | 1.63E-02 |
| Cluster-40555.240402 | FAR1 | LOC_Os12g06380.1 | 0.00E+00 | 2311 | 3.10 | 9.44E-04 | 2.85 | 1.92E-02 |
| Cluster-40555.166373 | G2-like | LOC_Os01g13740.1 | 1.00E-106 | 385 | Inf | 8.75E-31 | Inf | 6.87E-05 |
| Cluster-40555.174898 | G2-like | LOC_Os01g13740.1 | 4.00E-106 | 383 | 4.60 | 9.13E-13 | 3.66 | 4.19E-04 |
| Cluster-40555.183351 | G2-like | LOC_Os03g55590.1 | 9.00E-93 | 339 | 0.96 | 1.18E-03 | 1.29 | 2.93E-04 |
| Cluster-40555.196765 | G2-like | LOC_Os03g55590.1 | 1.00E-55 | 215 | 3.57 | 2.60E-02 | 2.17 | 4.30E-02 |
| Cluster-40555.196766 | G2-like | LOC_Os03g55590.1 | 1.00E-60 | 231 | 1.55 | 9.59E-05 | 1.30 | 5.75E-03 |
| Cluster-40555.233429 | G2-like | LOC_Os01g13740.1 | 5.00E-101 | 366 | Inf | 6.03E-19 | Inf | 4.57E-02 |
| Cluster-40555.198012 | GATA | LOC_Os04g45650.2 | 2.00E-131 | 466 | -5.31 | 2.69E-04 | -2.60 | 1.75E-03 |
| Cluster-40555.208893 | GATA | LOC_Os04g45650.2 | 4.00E-93 | 339 | -3.28 | 4.14E-04 | -2.57 | 4.91E-03 |
| Cluster-40555.188605 | GATA | LOC_Os01g54210.1 | 0.00E+00 | 754 | 0.95 | 7.69E-04 | 0.82 | 4.70E-02 |
| Cluster-40555.208892 | GATA | LOC_Os04g45650.2 | 2.00E-147 | 520 | 2.40 | 1.43E-03 | 1.80 | 2.67E-02 |
| Cluster-40555.158020 | GRAS | LOC_Os10g40390.1 | 1.00E-123 | 440 | 3.24 | 1.24E-03 | 1.83 | 4.31E-02 |
| Cluster-40555.204370 | GRAS | LOC_Os01g71970.1 | 0.00E+00 | 651 | 3.31 | 1.35E-04 | 2.76 | 5.74E-04 |
| Cluster-40555.203336 | GRF | LOC_Os04g51190.1 | 1.00E-34 | 145 | Inf | 1.22E-04 | 3.60 | 6.67E-04 |
| Cluster-40555.184232 | HD-ZIP | LOC_Os08g04190.1 | 0.00E+00 | 883 | -2.83 | 3.33E-10 | -1.25 | 2.07E-02 |
| Cluster-40555.206892 | HD-ZIP | LOC_Os07g39320.1 | 4.00E-154 | 542 | -3.03 | 1.37E-06 | -1.75 | 2.44E-02 |
| Cluster-40555.225157 | HD-ZIP | LOC_Os02g43330.1 | 2.00E-52 | 204 | 0.00 | 1.54E-13 | -6.99 | 2.11E-25 |
| Cluster-40555.170890 | HD-ZIP | LOC_Os03g12860.1 | 4.00E-114 | 409 | 2.41 | 3.08E-05 | 1.71 | 2.56E-02 |
| Cluster-40555.180440 | HD-ZIP | LOC_Os04g46350.1 | 3.00E-48 | 189 | 5.07 | 3.19E-08 | 2.31 | 7.63E-05 |
| Cluster-40555.188403 | HD-ZIP | LOC_Os04g45810.1 | 0.00E+00 | 682 | 4.29 | 1.48E-46 | 1.69 | 1.02E-06 |
| Cluster-40555.195704 | HD-ZIP | LOC_Os02g49700.1 | 0.00E+00 | 756 | 1.30 | 1.02E-02 | 1.90 | 2.26E-05 |
| Cluster-40555.213365 | HD-ZIP | LOC_Os03g12860.1 | 1.00E-94 | 344 | Inf | 7.76E-04 | Inf | 2.32E-02 |
| Cluster-40555.306180 | HD-ZIP | LOC_Os01g55549.1 | 0.00E+00 | 1064 | Inf | 6.72E-05 | Inf | 7.48E-03 |
| Cluster-40555.172251 | HSF | LOC_Os03g53340.2 | 5.00E-124 | 442 | -4.33 | 2.20E-06 | -3.54 | 4.38E-13 |
| Cluster-40555.198525 | HSF | LOC_Os09g35790.1 | 4.00E-120 | 429 | -4.93 | 7.46E-41 | -2.53 | 5.44E-08 |
| Cluster-40555.225819 | HSF | LOC_Os03g58160.1 | 7.00E-167 | 584 | -1.14 | 3.61E-02 | -1.26 | 2.99E-02 |
| Cluster-40555.183779 | HSF | LOC_Os03g58160.1 | 7.00E-167 | 584 | Inf | 3.49E-11 | Inf | 2.28E-12 |

| | | | | | | | | |
|---|---|---|---|---|---|---|---|---|
| Cluster-40555.192124 | HSF | LOC_Os01g53220.1 | 1.00E-115 | 414 | 4.76 | 6.48E-33 | 1.92 | 2.33E-04 |
| Cluster-40555.198527 | HSF | LOC_Os09g35790.1 | 2.00E-123 | 440 | 2.85 | 1.89E-10 | 2.33 | 1.10E-04 |
| Cluster-40555.157232 | LSD | LOC_Os12g41700.1 | 1.00E-106 | 385 | 3.54 | 9.12E-09 | 2.72 | 3.12E-04 |
| Cluster-40555.158078 | MIKC_MADS | LOC_Os06g06750.1 | 4.00E-122 | 435 | 6.58 | 1.43E-04 | 5.90 | 1.94E-03 |
| Cluster-40555.175507 | MIKC_MADS | LOC_Os12g10540.4 | 2.00E-167 | 586 | Inf | 3.73E-42 | Inf | 7.04E-27 |
| Cluster-40555.190290 | MIKC_MADS | LOC_Os12g10540.5 | 3.00E-161 | 566 | 5.42 | 1.00E-20 | Inf | 2.16E-10 |
| Cluster-40555.89704 | M-type_MADS | LOC_Os06g11970.1 | 1.00E-64 | 244 | 6.18 | 6.17E-03 | Inf | 5.71E-05 |
| Cluster-40555.200316 | MYB | LOC_Os04g50770.1 | 3.00E-106 | 383 | 0.00 | 2.47E-02 | 0.00 | 2.46E-05 |
| Cluster-40555.131430 | MYB | LOC_Os07g04700.1 | 0.00E+00 | 1565 | 1.90 | 3.96E-03 | 1.09 | 2.43E-02 |
| Cluster-40555.174498 | MYB | LOC_Os09g01960.1 | 3.00E-25 | 113 | Inf | 2.30E-36 | 9.65 | 6.52E-37 |
| Cluster-40555.179825 | MYB | LOC_Os11g45740.1 | 1.00E-100 | 364 | 3.39 | 5.50E-07 | 1.86 | 3.45E-02 |
| Cluster-40555.184294 | MYB | LOC_Os06g10350.1 | 2.00E-56 | 217 | 1.53 | 8.82E-04 | 3.10 | 1.06E-16 |
| Cluster-40555.184530 | MYB | LOC_Os01g50110.1 | 2.00E-29 | 126 | Inf | 7.56E-18 | 6.35 | 2.85E-04 |
| Cluster-40555.189597 | MYB | LOC_Os06g10350.1 | 2.00E-56 | 217 | 4.67 | 1.48E-35 | 5.68 | 1.97E-32 |
| Cluster-40555.211498 | MYB | LOC_Os04g50770.1 | 6.00E-118 | 422 | 4.02 | 2.31E-17 | 2.91 | 1.09E-08 |
| Cluster-40555.215102 | MYB | LOC_Os08g43550.1 | 2.00E-40 | 163 | Inf | 8.75E-16 | 2.39 | 1.24E-05 |
| Cluster-40555.191840 | MYB_related | LOC_Os01g34060.1 | 4.00E-82 | 303 | Inf | 3.00E-05 | Inf | 1.82E-03 |
| Cluster-40555.151396 | NAC | LOC_Os03g21030.1 | 3.00E-111 | 398 | 0.00 | 4.95E-06 | -5.73 | 1.23E-11 |
| Cluster-40555.151794 | NAC | LOC_Os02g57650.1 | 6.00E-51 | 198 | -3.18 | 2.49E-02 | -2.48 | 6.17E-03 |
| Cluster-40555.194173 | NAC | LOC_Os04g42940.1 | 1.00E-136 | 484 | 0.00 | 1.33E-05 | 0.00 | 1.78E-04 |
| Cluster-40555.197659 | NAC | LOC_Os02g57650.1 | 4.00E-53 | 207 | -2.76 | 1.10E-07 | -2.40 | 9.83E-09 |
| Cluster-40555.200046 | NAC | LOC_Os05g35170.4 | 0.00E+00 | 636 | -4.48 | 4.90E-03 | -3.12 | 4.13E-04 |
| Cluster-40555.202205 | NAC | LOC_Os02g57650.1 | 4.00E-53 | 207 | -3.86 | 4.78E-11 | -2.43 | 1.90E-06 |
| Cluster-40555.225773 | NAC | LOC_Os01g71790.1 | 9.00E-92 | 335 | -2.04 | 5.66E-05 | -2.63 | 2.97E-03 |
| Cluster-40555.163727 | NAC | LOC_Os03g21030.1 | 3.00E-111 | 398 | Inf | 3.65E-18 | 6.51 | 6.82E-16 |
| Cluster-40555.168512 | NAC | LOC_Os05g35170.4 | 0.00E+00 | 632 | 2.28 | 6.38E-05 | 1.42 | 7.28E-03 |
| Cluster-40555.186331 | NAC | LOC_Os05g34830.1 | 1.00E-57 | 220 | 6.46 | 1.38E-03 | Inf | 1.29E-02 |
| Cluster-40555.187541 | NAC | LOC_Os01g66120.1 | 3.00E-101 | 364 | 6.79 | 9.51E-50 | 5.90 | 9.53E-39 |
| Cluster-40555.192230 | NAC | LOC_Os04g42940.1 | 4.00E-146 | 516 | Inf | 6.18E-07 | Inf | 5.85E-05 |
| Cluster-40555.192486 | NAC | LOC_Os02g57650.1 | 4.00E-53 | 207 | 5.43 | 1.36E-32 | 5.02 | 1.81E-27 |
| Cluster-40555.202381 | NAC | LOC_Os07g48450.1 | 2.00E-47 | 187 | 1.68 | 1.49E-06 | 1.24 | 3.11E-03 |
| Cluster-40555.224037 | NAC | LOC_Os11g03300.1 | 1.00E-106 | 383 | Inf | 2.53E-02 | Inf | 2.27E-06 |

| | | | | | | | | |
|---|---|---|---|---|---|---|---|---|
| Cluster-40555.206159 | NF-YA | LOC_Os03g07880.1 | 2.00E-145 | 512 | 3.78 | 1.25E-10 | 2.76 | 9.74E-03 |
| Cluster-40555.203810 | NF-YC | LOC_Os04g58680.1 | 1.00E-68 | 257 | 2.14 | 7.59E-04 | 2.83 | 5.52E-06 |
| Cluster-40555.196262 | SRS | LOC_Os01g72490.1 | 8.00E-119 | 425 | 4.40 | 2.27E-04 | 2.06 | 3.62E-02 |
| Cluster-40555.237203 | Trihelix | LOC_Os04g40930.1 | 2.00E-148 | 523 | 0.00 | 9.83E-07 | 0.00 | 2.58E-03 |
| Cluster-40555.218797 | WOX | LOC_Os01g60270.2 | 7.00E-179 | 625 | Inf | 4.97E-10 | 4.34 | 2.11E-08 |
| Cluster-40555.143406 | WRKY | LOC_Os03g55080.1 | 6.00E-109 | 392 | -3.25 | 1.80E-05 | 0.00 | 2.84E-06 |
| Cluster-40555.147227 | WRKY | LOC_Os09g25060.1 | 7.00E-172 | 601 | 0.00 | 2.78E-02 | 0.00 | 4.83E-04 |
| Cluster-40555.183899 | WRKY | LOC_Os01g53260.1 | 2.00E-35 | 147 | 0.00 | 2.09E-32 | -5.67 | 9.90E-03 |
| Cluster-40555.192086 | WRKY | LOC_Os09g25060.1 | 7.00E-18 | 89.8 | -9.64 | 1.71E-73 | -6.39 | 1.57E-32 |
| Cluster-40555.99532 | WRKY | LOC_Os01g43550.2 | 2.00E-128 | 457 | 0.00 | 8.56E-06 | 0.00 | 3.45E-04 |
| Cluster-40555.162685 | WRKY | LOC_Os01g53260.1 | 2.00E-116 | 416 | 3.91 | 8.80E-06 | 1.96 | 5.83E-03 |
| Cluster-40555.173367 | WRKY | LOC_Os01g54600.1 | 5.00E-60 | 230 | 4.17 | 2.40E-13 | 5.16 | 1.19E-13 |
| Cluster-40555.182385 | WRKY | LOC_Os01g53260.1 | 7.00E-97 | 351 | 2.61 | 4.04E-02 | Inf | 1.54E-05 |
| Cluster-40555.182392 | WRKY | LOC_Os01g53260.1 | 2.00E-35 | 147 | Inf | 2.74E-05 | Inf | 8.55E-09 |
| Cluster-40555.185107 | WRKY | LOC_Os04g21950.1 | 0.00E+00 | 649 | 1.16 | 3.87E-04 | 1.36 | 4.72E-04 |
| Cluster-40555.199963 | WRKY | LOC_Os03g55080.1 | 4.00E-101 | 366 | Inf | 5.34E-19 | Inf | 3.89E-02 |
| Cluster-40555.203890 | WRKY | LOC_Os09g25060.1 | 7.00E-18 | 89.8 | 7.74 | 1.41E-64 | Inf | 9.39E-52 |
| Cluster-40555.216207 | WRKY | LOC_Os09g25060.1 | 3.00E-165 | 579 | 5.62 | 1.57E-10 | 2.86 | 8.03E-03 |
| Cluster-40555.202559 | ZF-HD | LOC_Os09g29130.1 | 4.00E-174 | 608 | 1.47 | 3.26E-03 | 1.38 | 3.02E-02 |

Note: B means brown seeds,Y means yellow seeds, FC means fold change

# Table S3: Correlation analysis of TFs with *ANR* and *ANS* at transcript level

A: Pearson correlation analysis

| GeneID | Type | Cluster-40555.170438 | | Cluster-40555.182940 | | Cluster-40555.226813 | | Cluster-40555.154499 | | Cluster-40555.182911 | | Cluster-40555.183520 | | Cluster-40555.186665 | |
|---|---|---|---|---|---|---|---|---|---|---|---|---|---|---|---|
| | | r | p | r | p | r | p | r | p | r | p | r | p | r | p |
| Cluster-40555.170438 | ANR | 1 | 0 | 0.82 | 0.01 | -0.82 | 0.01 | 0.64 | 0.09 | 0.58 | 0.13 | 0.55 | 0.15 | 0.64 | 0.09 |
| Cluster-40555.182940 | ANR | 0.82 | 0.01 | 1 | 0 | -0.84 | 0.01 | 0.94 | 0 | 0.91 | 0 | 0.88 | 0 | 0.95 | 0 |
| Cluster-40555.226813 | ANR | -0.82 | 0.01 | -0.84 | 0.01 | 1 | 0 | -0.79 | 0.02 | -0.75 | 0.03 | -0.76 | 0.03 | -0.8 | 0.02 |
| Cluster-40555.154499 | ANS | 0.64 | 0.09 | 0.94 | 0 | -0.79 | 0.02 | 1 | 0 | 0.99 | 0 | 0.98 | 0 | 1 | 0 |
| Cluster-40555.182911 | ANS | 0.58 | 0.13 | 0.91 | 0 | -0.75 | 0.03 | 0.99 | 0 | 1 | 0 | 0.99 | 0 | 0.99 | 0 |
| Cluster-40555.183520 | ANS | 0.55 | 0.15 | 0.88 | 0 | -0.76 | 0.03 | 0.98 | 0 | 0.99 | 0 | 1 | 0 | 0.98 | 0 |
| Cluster-40555.186665 | ANS | 0.64 | 0.09 | 0.95 | 0 | -0.8 | 0.02 | 1 | 0 | 0.99 | 0 | 0.98 | 0 | 1 | 0 |
| Cluster-40555.169774 | AP2 | 0.86 | 0.01 | 0.47 | 0.24 | -0.59 | 0.12 | 0.24 | 0.56 | 0.19 | 0.65 | 0.18 | 0.67 | 0.25 | 0.56 |
| Cluster-40555.209356 | BES1 | 0.89 | 0 | 0.87 | 0 | -0.86 | 0.01 | 0.8 | 0.02 | 0.78 | 0.02 | 0.77 | 0.02 | 0.8 | 0.02 |
| Cluster-40555.146730 | bHLH | -0.7 | 0.05 | -0.81 | 0.02 | 0.5 | 0.21 | -0.73 | 0.04 | -0.73 | 0.04 | -0.69 | 0.06 | -0.73 | 0.04 |
| Cluster-40555.177017 | bHLH | -0.71 | 0.05 | -0.82 | 0.01 | 0.51 | 0.2 | -0.73 | 0.04 | -0.75 | 0.03 | -0.72 | 0.04 | -0.74 | 0.04 |
| Cluster-40555.184841 | bHLH | -0.88 | 0 | -0.89 | 0 | 0.98 | 0 | -0.81 | 0.01 | -0.77 | 0.03 | -0.77 | 0.03 | -0.82 | 0.01 |
| Cluster-40555.201242 | bHLH | -0.75 | 0.03 | -0.81 | 0.02 | 0.5 | 0.2 | -0.71 | 0.05 | -0.7 | 0.05 | -0.66 | 0.07 | -0.71 | 0.05 |
| Cluster-40555.234751 | bHLH | -0.74 | 0.04 | -0.83 | 0.01 | 0.51 | 0.2 | -0.73 | 0.04 | -0.73 | 0.04 | -0.69 | 0.06 | -0.73 | 0.04 |
| Cluster-40555.272493 | bHLH | -0.7 | 0.05 | -0.84 | 0.01 | 0.5 | 0.21 | -0.77 | 0.03 | -0.76 | 0.03 | -0.72 | 0.05 | -0.76 | 0.03 |
| Cluster-40555.158717 | bHLH | 0.9 | 0 | 0.57 | 0.14 | -0.64 | 0.09 | 0.33 | 0.42 | 0.28 | 0.5 | 0.27 | 0.51 | 0.34 | 0.41 |
| Cluster-40555.160644 | bHLH | 0.93 | 0 | 0.67 | 0.07 | -0.76 | 0.03 | 0.51 | 0.2 | 0.45 | 0.26 | 0.44 | 0.27 | 0.51 | 0.2 |
| Cluster-40555.182851 | bHLH | 0.94 | 0 | 0.89 | 0 | -0.89 | 0 | 0.77 | 0.03 | 0.74 | 0.04 | 0.73 | 0.04 | 0.77 | 0.02 |
| Cluster-40555.187730 | bHLH | 0.79 | 0.02 | 0.89 | 0 | -0.82 | 0.01 | 0.92 | 0 | 0.88 | 0 | 0.88 | 0 | 0.9 | 0 |
| Cluster-40555.188801 | bHLH | 0.87 | 0.01 | 0.44 | 0.28 | -0.61 | 0.11 | 0.21 | 0.61 | 0.14 | 0.74 | 0.14 | 0.74 | 0.21 | 0.62 |
| Cluster-40555.198935 | bHLH | 0.83 | 0.01 | 0.43 | 0.28 | -0.51 | 0.2 | 0.19 | 0.65 | 0.13 | 0.76 | 0.11 | 0.79 | 0.19 | 0.65 |
| Cluster-40555.201846 | bHLH | 0.83 | 0.01 | 0.45 | 0.26 | -0.59 | 0.13 | 0.2 | 0.63 | 0.15 | 0.72 | 0.15 | 0.73 | 0.21 | 0.62 |
| Cluster-40555.201847 | bHLH | 0.76 | 0.03 | 0.29 | 0.48 | -0.42 | 0.3 | 0.08 | 0.85 | 0.01 | 0.98 | 0 | 1 | 0.07 | 0.88 |
| Cluster-40555.214608 | bHLH | 0.96 | 0 | 0.88 | 0 | -0.88 | 0 | 0.75 | 0.03 | 0.71 | 0.05 | 0.7 | 0.05 | 0.75 | 0.03 |

| | | | | | | | | | | | | | | | |
|---|---|---|---|---|---|---|---|---|---|---|---|---|---|---|---|
| Cluster-40555.226537 | bHLH | 0.74 | 0.03 | 0.31 | 0.45 | -0.52 | 0.19 | 0.08 | 0.85 | 0.02 | 0.96 | 0.02 | 0.95 | 0.09 | 0.84 |
| Cluster-40555.230888 | bHLH | 0.6 | 0.12 | 0.79 | 0.02 | -0.79 | 0.02 | 0.87 | 0.01 | 0.89 | 0 | 0.93 | 0 | 0.87 | 0.01 |
| Cluster-40555.239070 | bHLH | 0.93 | 0 | 0.6 | 0.11 | -0.62 | 0.1 | 0.38 | 0.35 | 0.29 | 0.49 | 0.25 | 0.54 | 0.36 | 0.37 |
| Cluster-40555.97572 | bHLH | 0.86 | 0.01 | 0.43 | 0.29 | -0.55 | 0.16 | 0.2 | 0.64 | 0.12 | 0.77 | 0.12 | 0.78 | 0.18 | 0.66 |
| Cluster-40555.171315 | bZIP | -0.72 | 0.04 | -0.79 | 0.02 | 0.57 | 0.14 | -0.7 | 0.05 | -0.69 | 0.06 | -0.66 | 0.07 | -0.7 | 0.05 |
| Cluster-40555.203533 | bZIP | -0.75 | 0.03 | -0.88 | 0 | 0.62 | 0.1 | -0.82 | 0.01 | -0.81 | 0.01 | -0.78 | 0.02 | -0.81 | 0.01 |
| Cluster-40555.218108 | bZIP | -0.86 | 0.01 | -0.91 | 0 | 0.88 | 0 | -0.85 | 0.01 | -0.82 | 0.01 | -0.8 | 0.02 | -0.85 | 0.01 |
| Cluster-40555.178265 | bZIP | 0.98 | 0 | 0.77 | 0.03 | -0.81 | 0.01 | 0.57 | 0.14 | 0.52 | 0.19 | 0.5 | 0.21 | 0.57 | 0.14 |
| Cluster-40555.179322 | bZIP | 0.93 | 0 | 0.57 | 0.14 | -0.66 | 0.08 | 0.34 | 0.41 | 0.27 | 0.51 | 0.26 | 0.53 | 0.33 | 0.42 |
| Cluster-40555.195157 | bZIP | 0.51 | 0.2 | 0.88 | 0 | -0.7 | 0.05 | 0.97 | 0 | 0.99 | 0 | 0.99 | 0 | 0.98 | 0 |
| Cluster-40555.219797 | bZIP | 0.46 | 0.25 | 0.82 | 0.01 | -0.56 | 0.15 | 0.94 | 0 | 0.96 | 0 | 0.95 | 0 | 0.93 | 0 |
| Cluster-40555.142929 | C2H2 | -0.78 | 0.02 | -0.88 | 0 | 0.62 | 0.1 | -0.81 | 0.01 | -0.8 | 0.02 | -0.76 | 0.03 | -0.81 | 0.02 |
| Cluster-40555.175851 | C2H2 | -0.75 | 0.03 | -0.77 | 0.03 | 0.98 | 0 | -0.73 | 0.04 | -0.69 | 0.06 | -0.71 | 0.05 | -0.74 | 0.04 |
| Cluster-40555.141702 | C2H2 | 0.68 | 0.06 | 0.18 | 0.66 | -0.46 | 0.25 | -0.05 | 0.91 | -0.11 | 0.79 | -0.1 | 0.81 | -0.05 | 0.92 |
| Cluster-40555.150134 | C2H2 | 0.84 | 0.01 | 0.41 | 0.31 | -0.56 | 0.15 | 0.16 | 0.71 | 0.09 | 0.82 | 0.09 | 0.84 | 0.16 | 0.7 |
| Cluster-40555.180184 | C2H2 | 0.7 | 0.05 | 0.23 | 0.58 | -0.36 | 0.38 | 0 | 1 | -0.06 | 0.9 | -0.07 | 0.87 | 0 | 1 |
| Cluster-40555.180185 | C2H2 | 0.84 | 0.01 | 0.43 | 0.28 | -0.56 | 0.15 | 0.18 | 0.67 | 0.12 | 0.77 | 0.12 | 0.78 | 0.19 | 0.66 |
| Cluster-40555.180186 | C2H2 | 0.8 | 0.02 | 0.36 | 0.38 | -0.5 | 0.21 | 0.11 | 0.8 | 0.05 | 0.9 | 0.04 | 0.92 | 0.11 | 0.79 |
| Cluster-40555.194927 | C2H2 | 0.81 | 0.01 | 0.36 | 0.38 | -0.51 | 0.19 | 0.11 | 0.8 | 0.04 | 0.92 | 0.04 | 0.92 | 0.11 | 0.8 |
| Cluster-40555.204350 | C3H | -0.57 | 0.14 | -0.81 | 0.01 | 0.42 | 0.3 | -0.78 | 0.02 | -0.8 | 0.02 | -0.76 | 0.03 | -0.79 | 0.02 |
| Cluster-40555.190917 | C3H | 0.53 | 0.18 | 0.85 | 0.01 | -0.76 | 0.03 | 0.96 | 0 | 0.98 | 0 | 1 | 0 | 0.96 | 0 |
| Cluster-40555.212569 | C3H | 0.98 | 0 | 0.91 | 0 | -0.88 | 0 | 0.77 | 0.02 | 0.72 | 0.05 | 0.7 | 0.05 | 0.77 | 0.03 |
| Cluster-40555.233380 | C3H | 0.67 | 0.07 | 0.96 | 0 | -0.79 | 0.02 | 0.99 | 0 | 0.98 | 0 | 0.96 | 0 | 0.99 | 0 |
| Cluster-40555.235817 | C3H | 0.89 | 0 | 0.96 | 0 | -0.9 | 0 | 0.86 | 0.01 | 0.84 | 0.01 | 0.83 | 0.01 | 0.88 | 0 |
| Cluster-40555.191531 | CO-like | 0.85 | 0.01 | 0.45 | 0.26 | -0.57 | 0.14 | 0.19 | 0.65 | 0.14 | 0.75 | 0.13 | 0.76 | 0.2 | 0.64 |
| Cluster-40555.206298 | CO-like | 0.67 | 0.07 | 0.21 | 0.61 | -0.51 | 0.2 | -0.03 | 0.95 | -0.09 | 0.84 | -0.08 | 0.85 | -0.01 | 0.97 |
| Cluster-40555.233365 | CO-like | 0.81 | 0.01 | 0.46 | 0.25 | -0.61 | 0.11 | 0.2 | 0.64 | 0.15 | 0.72 | 0.14 | 0.75 | 0.22 | 0.61 |
| Cluster-40555.179654 | DBB | -0.9 | 0 | -0.93 | 0 | 0.95 | 0 | -0.85 | 0.01 | -0.81 | 0.01 | -0.8 | 0.02 | -0.86 | 0.01 |

| | | | | | | | | | | | | | | | |
|---|---|---|---|---|---|---|---|---|---|---|---|---|---|---|---|
| Cluster-40555.194971 | DBB | -0.78 | 0.02 | -0.8 | 0.02 | 0.99 | 0 | -0.76 | 0.03 | -0.72 | 0.04 | -0.74 | 0.04 | -0.77 | 0.03 |
| Cluster-40555.194972 | DBB | -0.69 | 0.06 | -0.86 | 0.01 | 0.6 | 0.11 | -0.83 | 0.01 | -0.83 | 0.01 | -0.8 | 0.02 | -0.83 | 0.01 |
| Cluster-40555.194976 | DBB | 0.77 | 0.03 | 0.94 | 0 | -0.89 | 0 | 0.93 | 0 | 0.93 | 0 | 0.93 | 0 | 0.94 | 0 |
| Cluster-40555.199338 | DBB | 0.68 | 0.06 | 0.96 | 0 | -0.84 | 0.01 | 0.99 | 0 | 0.98 | 0 | 0.98 | 0 | 0.99 | 0 |
| Cluster-40555.183211 | Dof | -0.86 | 0.01 | -0.86 | 0.01 | 0.96 | 0 | -0.77 | 0.03 | -0.73 | 0.04 | -0.74 | 0.04 | -0.78 | 0.02 |
| Cluster-40555.188528 | Dof | -0.78 | 0.02 | -0.86 | 0.01 | 0.56 | 0.15 | -0.76 | 0.03 | -0.75 | 0.03 | -0.71 | 0.05 | -0.76 | 0.03 |
| Cluster-40555.194651 | Dof | -0.81 | 0.01 | -0.81 | 0.01 | 0.99 | 0 | -0.78 | 0.02 | -0.73 | 0.04 | -0.74 | 0.03 | -0.78 | 0.02 |
| Cluster-40555.208868 | Dof | -0.62 | 0.1 | -0.79 | 0.02 | 0.4 | 0.32 | -0.72 | 0.04 | -0.73 | 0.04 | -0.69 | 0.06 | -0.72 | 0.04 |
| Cluster-40555.230137 | Dof | -0.85 | 0.01 | -0.93 | 0 | 0.8 | 0.02 | -0.85 | 0.01 | -0.83 | 0.01 | -0.81 | 0.01 | -0.86 | 0.01 |
| Cluster-40555.168102 | Dof | 0.79 | 0.02 | 0.47 | 0.24 | -0.6 | 0.11 | 0.26 | 0.54 | 0.23 | 0.58 | 0.24 | 0.56 | 0.27 | 0.52 |
| Cluster-40555.171911 | Dof | 0.96 | 0 | 0.85 | 0.01 | -0.88 | 0 | 0.72 | 0.04 | 0.69 | 0.06 | 0.69 | 0.06 | 0.73 | 0.04 |
| Cluster-40555.161069 | ERF | -0.77 | 0.02 | -0.85 | 0.01 | 0.68 | 0.06 | -0.77 | 0.03 | -0.75 | 0.03 | -0.71 | 0.05 | -0.77 | 0.03 |
| Cluster-40555.161377 | ERF | -0.52 | 0.18 | -0.65 | 0.08 | 0.24 | 0.57 | -0.57 | 0.14 | -0.58 | 0.13 | -0.53 | 0.17 | -0.57 | 0.14 |
| Cluster-40555.165988 | ERF | -0.83 | 0.01 | -0.92 | 0 | 0.69 | 0.06 | -0.82 | 0.01 | -0.81 | 0.01 | -0.79 | 0.02 | -0.83 | 0.01 |
| Cluster-40555.192007 | ERF | -0.69 | 0.06 | -0.86 | 0.01 | 0.52 | 0.19 | -0.79 | 0.02 | -0.8 | 0.02 | -0.76 | 0.03 | -0.8 | 0.02 |
| Cluster-40555.175287 | ERF | 0.83 | 0.01 | 0.39 | 0.34 | -0.56 | 0.15 | 0.15 | 0.73 | 0.08 | 0.85 | 0.08 | 0.85 | 0.14 | 0.73 |
| Cluster-40555.187700 | ERF | 0.7 | 0.06 | 0.22 | 0.59 | -0.5 | 0.21 | -0.02 | 0.96 | -0.08 | 0.85 | -0.07 | 0.87 | -0.01 | 0.98 |
| Cluster-40555.188580 | ERF | 0.78 | 0.02 | 0.88 | 0 | -0.85 | 0.01 | 0.76 | 0.03 | 0.77 | 0.03 | 0.75 | 0.03 | 0.8 | 0.02 |
| Cluster-40555.201722 | ERF | 0.86 | 0.01 | 0.54 | 0.17 | -0.65 | 0.08 | 0.3 | 0.48 | 0.25 | 0.55 | 0.24 | 0.56 | 0.31 | 0.45 |
| Cluster-40555.241796 | FAR1 | -0.77 | 0.03 | -0.74 | 0.04 | 0.98 | 0 | -0.71 | 0.05 | -0.66 | 0.07 | -0.69 | 0.06 | -0.71 | 0.05 |
| Cluster-40555.209479 | FAR1 | 0.6 | 0.11 | 0.81 | 0.01 | -0.74 | 0.03 | 0.85 | 0.01 | 0.87 | 0 | 0.9 | 0 | 0.85 | 0.01 |
| Cluster-40555.240402 | FAR1 | 0.85 | 0.01 | 0.57 | 0.14 | -0.65 | 0.08 | 0.31 | 0.46 | 0.27 | 0.52 | 0.25 | 0.55 | 0.33 | 0.42 |
| Cluster-40555.166373 | G2-like | 0.84 | 0.01 | 0.42 | 0.3 | -0.55 | 0.16 | 0.16 | 0.7 | 0.1 | 0.81 | 0.09 | 0.82 | 0.17 | 0.69 |
| Cluster-40555.174898 | G2-like | 0.81 | 0.01 | 0.42 | 0.31 | -0.56 | 0.15 | 0.16 | 0.71 | 0.11 | 0.8 | 0.1 | 0.81 | 0.17 | 0.69 |
| Cluster-40555.183351 | G2-like | 0.56 | 0.15 | 0.03 | 0.94 | -0.35 | 0.39 | -0.18 | 0.68 | -0.24 | 0.56 | -0.23 | 0.58 | -0.18 | 0.67 |
| Cluster-40555.196765 | G2-like | 0.81 | 0.01 | 0.51 | 0.2 | -0.43 | 0.29 | 0.23 | 0.58 | 0.2 | 0.64 | 0.17 | 0.69 | 0.25 | 0.56 |
| Cluster-40555.196766 | G2-like | 0.78 | 0.02 | 0.33 | 0.43 | -0.52 | 0.18 | 0.09 | 0.83 | 0.03 | 0.95 | 0.03 | 0.95 | 0.09 | 0.83 |
| Cluster-40555.233429 | G2-like | 0.83 | 0.01 | 0.41 | 0.31 | -0.55 | 0.16 | 0.16 | 0.7 | 0.1 | 0.81 | 0.1 | 0.81 | 0.16 | 0.7 |

| | | | | | | | | | | | | | | | |
|---|---|---|---|---|---|---|---|---|---|---|---|---|---|---|---|
| Cluster-40555.198012 | GATA | -0.85 | 0.01 | -0.87 | 0 | 0.79 | 0.02 | -0.79 | 0.02 | -0.76 | 0.03 | -0.73 | 0.04 | -0.79 | 0.02 |
| Cluster-40555.208893 | GATA | -0.84 | 0.01 | -0.92 | 0 | 0.78 | 0.02 | -0.82 | 0.01 | -0.79 | 0.02 | -0.76 | 0.03 | -0.83 | 0.01 |
| Cluster-40555.188605 | GATA | 0.92 | 0 | 0.76 | 0.03 | -0.8 | 0.02 | 0.64 | 0.09 | 0.61 | 0.11 | 0.6 | 0.11 | 0.64 | 0.09 |
| Cluster-40555.208892 | GATA | 0.93 | 0 | 0.91 | 0 | -0.88 | 0 | 0.85 | 0.01 | 0.79 | 0.02 | 0.78 | 0.02 | 0.83 | 0.01 |
| Cluster-40555.158020 | GRAS | 0.85 | 0.01 | 0.44 | 0.27 | -0.57 | 0.14 | 0.19 | 0.65 | 0.13 | 0.76 | 0.12 | 0.78 | 0.19 | 0.65 |
| Cluster-40555.204370 | GRAS | 0.84 | 0.01 | 0.48 | 0.23 | -0.6 | 0.12 | 0.28 | 0.5 | 0.24 | 0.57 | 0.24 | 0.57 | 0.28 | 0.5 |
| Cluster-40555.203336 | GRF | 0.93 | 0 | 0.96 | 0 | -0.89 | 0 | 0.85 | 0.01 | 0.81 | 0.01 | 0.79 | 0.02 | 0.86 | 0.01 |
| Cluster-40555.184232 | HD-ZIP | -0.92 | 0 | -0.88 | 0 | 0.79 | 0.02 | -0.77 | 0.03 | -0.75 | 0.03 | -0.74 | 0.04 | -0.77 | 0.03 |
| Cluster-40555.206892 | HD-ZIP | -0.58 | 0.13 | -0.72 | 0.05 | 0.36 | 0.38 | -0.64 | 0.09 | -0.64 | 0.08 | -0.6 | 0.11 | -0.64 | 0.09 |
| Cluster-40555.225157 | HD-ZIP | -0.8 | 0.02 | -0.83 | 0.01 | 0.99 | 0 | -0.77 | 0.02 | -0.73 | 0.04 | -0.75 | 0.03 | -0.78 | 0.02 |
| Cluster-40555.170890 | HD-ZIP | 0.84 | 0.01 | 0.43 | 0.29 | -0.6 | 0.11 | 0.2 | 0.64 | 0.14 | 0.74 | 0.14 | 0.74 | 0.2 | 0.63 |
| Cluster-40555.180440 | HD-ZIP | 0.82 | 0.01 | 0.96 | 0 | -0.8 | 0.02 | 0.91 | 0 | 0.89 | 0 | 0.85 | 0.01 | 0.92 | 0 |
| Cluster-40555.188403 | HD-ZIP | 0.85 | 0.01 | 0.44 | 0.28 | -0.53 | 0.18 | 0.19 | 0.65 | 0.13 | 0.75 | 0.12 | 0.78 | 0.19 | 0.65 |
| Cluster-40555.195704 | HD-ZIP | 0.85 | 0.01 | 0.55 | 0.16 | -0.74 | 0.04 | 0.35 | 0.39 | 0.3 | 0.47 | 0.29 | 0.49 | 0.36 | 0.37 |
| Cluster-40555.213365 | HD-ZIP | 0.86 | 0.01 | 0.42 | 0.3 | -0.54 | 0.17 | 0.19 | 0.65 | 0.11 | 0.79 | 0.11 | 0.8 | 0.17 | 0.68 |
| Cluster-40555.306180 | HD-ZIP | 0.75 | 0.03 | 0.38 | 0.35 | -0.52 | 0.19 | 0.12 | 0.78 | 0.08 | 0.84 | 0.08 | 0.86 | 0.14 | 0.74 |
| Cluster-40555.172251 | HSF | -0.68 | 0.06 | -0.62 | 0.1 | 0.92 | 0 | -0.6 | 0.12 | -0.54 | 0.16 | -0.56 | 0.15 | -0.6 | 0.12 |
| Cluster-40555.198525 | HSF | -0.73 | 0.04 | -0.82 | 0.01 | 0.5 | 0.21 | -0.72 | 0.04 | -0.72 | 0.04 | -0.68 | 0.06 | -0.72 | 0.04 |
| Cluster-40555.225819 | HSF | -0.8 | 0.02 | -0.92 | 0 | 0.83 | 0.01 | -0.83 | 0.01 | -0.83 | 0.01 | -0.82 | 0.01 | -0.85 | 0.01 |
| Cluster-40555.183779 | HSF | 0.81 | 0.01 | 0.94 | 0 | -0.83 | 0.01 | 0.86 | 0.01 | 0.86 | 0.01 | 0.85 | 0.01 | 0.87 | 0 |
| Cluster-40555.192124 | HSF | 0.94 | 0 | 0.65 | 0.08 | -0.7 | 0.05 | 0.44 | 0.27 | 0.39 | 0.34 | 0.39 | 0.35 | 0.44 | 0.27 |
| Cluster-40555.198527 | HSF | 0.96 | 0 | 0.77 | 0.03 | -0.83 | 0.01 | 0.59 | 0.12 | 0.54 | 0.16 | 0.54 | 0.17 | 0.6 | 0.12 |
| Cluster-40555.157232 | LSD | 0.91 | 0 | 0.56 | 0.15 | -0.66 | 0.08 | 0.33 | 0.43 | 0.27 | 0.52 | 0.26 | 0.54 | 0.33 | 0.43 |
| Cluster-40555.158078 | MIKC_MADS | 0.82 | 0.01 | 0.49 | 0.22 | -0.61 | 0.11 | 0.23 | 0.59 | 0.19 | 0.66 | 0.17 | 0.68 | 0.25 | 0.55 |
| Cluster-40555.175507 | MIKC_MADS | 0.82 | 0.01 | 0.4 | 0.32 | -0.53 | 0.17 | 0.14 | 0.73 | 0.09 | 0.84 | 0.08 | 0.85 | 0.15 | 0.73 |
| Cluster-40555.190290 | MIKC_MADS | 0.8 | 0.02 | 0.39 | 0.35 | -0.53 | 0.18 | 0.12 | 0.77 | 0.07 | 0.87 | 0.07 | 0.88 | 0.13 | 0.75 |
| Cluster-40555.89704 | M-type_MADS | 0.83 | 0.01 | 0.41 | 0.31 | -0.54 | 0.16 | 0.15 | 0.72 | 0.09 | 0.83 | 0.09 | 0.84 | 0.16 | 0.71 |
| Cluster-40555.200316 | MYB | -0.8 | 0.02 | -0.83 | 0.01 | 0.98 | 0 | -0.77 | 0.03 | -0.73 | 0.04 | -0.74 | 0.03 | -0.78 | 0.02 |

| | | | | | | | | | | | | | | | |
|---|---|---|---|---|---|---|---|---|---|---|---|---|---|---|---|
| Cluster-40555.131430 | MYB | 0.45 | 0.27 | 0.83 | 0.01 | -0.49 | 0.22 | 0.9 | 0 | 0.9 | 0 | 0.86 | 0.01 | 0.89 | 0 |
| Cluster-40555.174498 | MYB | 0.93 | 0 | 0.6 | 0.11 | -0.69 | 0.06 | 0.38 | 0.36 | 0.32 | 0.44 | 0.31 | 0.46 | 0.38 | 0.36 |
| Cluster-40555.179825 | MYB | 0.77 | 0.02 | 0.32 | 0.44 | -0.48 | 0.23 | 0.07 | 0.86 | 0.01 | 0.97 | 0.01 | 0.98 | 0.08 | 0.86 |
| Cluster-40555.184294 | MYB | 0.67 | 0.07 | 0.15 | 0.72 | -0.41 | 0.31 | -0.08 | 0.86 | -0.15 | 0.72 | -0.14 | 0.74 | -0.08 | 0.84 |
| Cluster-40555.184530 | MYB | 0.83 | 0.01 | 0.4 | 0.33 | -0.53 | 0.18 | 0.14 | 0.73 | 0.08 | 0.85 | 0.08 | 0.85 | 0.14 | 0.73 |
| Cluster-40555.189597 | MYB | 0.8 | 0.02 | 0.38 | 0.35 | -0.53 | 0.18 | 0.12 | 0.77 | 0.07 | 0.87 | 0.06 | 0.88 | 0.13 | 0.76 |
| Cluster-40555.211498 | MYB | 0.79 | 0.02 | 0.37 | 0.37 | -0.52 | 0.19 | 0.11 | 0.79 | 0.06 | 0.89 | 0.06 | 0.89 | 0.12 | 0.78 |
| Cluster-40555.215102 | MYB | 0.89 | 0 | 0.81 | 0.02 | -0.76 | 0.03 | 0.62 | 0.1 | 0.6 | 0.12 | 0.58 | 0.14 | 0.64 | 0.08 |
| Cluster-40555.191840 | MYB_related | 0.73 | 0.04 | 0.38 | 0.36 | -0.51 | 0.19 | 0.11 | 0.79 | 0.08 | 0.85 | 0.08 | 0.86 | 0.14 | 0.74 |
| Cluster-40555.151396 | NAC | -0.68 | 0.06 | -0.65 | 0.08 | 0.92 | 0 | -0.65 | 0.08 | -0.6 | 0.12 | -0.62 | 0.1 | -0.65 | 0.08 |
| Cluster-40555.151794 | NAC | -0.82 | 0.01 | -0.8 | 0.02 | 0.91 | 0 | -0.73 | 0.04 | -0.7 | 0.06 | -0.71 | 0.05 | -0.74 | 0.04 |
| Cluster-40555.194173 | NAC | -0.8 | 0.02 | -0.87 | 0 | 0.72 | 0.05 | -0.8 | 0.02 | -0.78 | 0.02 | -0.76 | 0.03 | -0.8 | 0.02 |
| Cluster-40555.197659 | NAC | -0.88 | 0 | -0.91 | 0 | 0.88 | 0 | -0.82 | 0.01 | -0.8 | 0.02 | -0.8 | 0.02 | -0.83 | 0.01 |
| Cluster-40555.200046 | NAC | -0.81 | 0.01 | -0.83 | 0.01 | 0.83 | 0.01 | -0.74 | 0.03 | -0.73 | 0.04 | -0.74 | 0.04 | -0.75 | 0.03 |
| Cluster-40555.202205 | NAC | -0.91 | 0 | -0.93 | 0 | 0.88 | 0 | -0.82 | 0.01 | -0.79 | 0.02 | -0.79 | 0.02 | -0.83 | 0.01 |
| Cluster-40555.225773 | NAC | -0.58 | 0.13 | -0.78 | 0.02 | 0.4 | 0.33 | -0.73 | 0.04 | -0.74 | 0.04 | -0.69 | 0.06 | -0.73 | 0.04 |
| Cluster-40555.163727 | NAC | 0.84 | 0.01 | 0.47 | 0.24 | -0.58 | 0.13 | 0.21 | 0.61 | 0.17 | 0.69 | 0.16 | 0.71 | 0.23 | 0.59 |
| Cluster-40555.168512 | NAC | 0.82 | 0.01 | 0.57 | 0.14 | -0.55 | 0.15 | 0.47 | 0.25 | 0.42 | 0.3 | 0.41 | 0.32 | 0.44 | 0.27 |
| Cluster-40555.186331 | NAC | 0.92 | 0 | 0.54 | 0.17 | -0.63 | 0.1 | 0.3 | 0.47 | 0.22 | 0.59 | 0.21 | 0.62 | 0.29 | 0.48 |
| Cluster-40555.187541 | NAC | 0.89 | 0 | 0.52 | 0.19 | -0.63 | 0.1 | 0.28 | 0.5 | 0.22 | 0.6 | 0.21 | 0.61 | 0.28 | 0.5 |
| Cluster-40555.192230 | NAC | 0.84 | 0.01 | 0.38 | 0.35 | -0.5 | 0.21 | 0.16 | 0.7 | 0.08 | 0.85 | 0.07 | 0.86 | 0.14 | 0.74 |
| Cluster-40555.192486 | NAC | 0.97 | 0 | 0.69 | 0.06 | -0.77 | 0.03 | 0.51 | 0.19 | 0.45 | 0.26 | 0.45 | 0.26 | 0.5 | 0.2 |
| Cluster-40555.202381 | NAC | 0.65 | 0.08 | 0.15 | 0.72 | -0.37 | 0.37 | -0.07 | 0.87 | -0.13 | 0.75 | -0.14 | 0.75 | -0.07 | 0.86 |
| Cluster-40555.224037 | NAC | 0.91 | 0 | 0.52 | 0.19 | -0.62 | 0.1 | 0.29 | 0.49 | 0.22 | 0.61 | 0.21 | 0.62 | 0.28 | 0.5 |
| Cluster-40555.206159 | NF-YA | 0.78 | 0.02 | 0.38 | 0.35 | -0.52 | 0.19 | 0.11 | 0.79 | 0.06 | 0.88 | 0.05 | 0.9 | 0.13 | 0.76 |
| Cluster-40555.203810 | NF-YC | 0.68 | 0.06 | 0.25 | 0.56 | -0.46 | 0.25 | -0.01 | 0.98 | -0.06 | 0.89 | -0.06 | 0.89 | 0.01 | 0.99 |
| Cluster-40555.196262 | SRS | 0.87 | 0 | 0.6 | 0.11 | -0.61 | 0.11 | 0.38 | 0.36 | 0.34 | 0.41 | 0.32 | 0.43 | 0.39 | 0.34 |
| Cluster-40555.237203 | Trihelix | -0.77 | 0.03 | -0.87 | 0.01 | 0.57 | 0.14 | -0.77 | 0.03 | -0.77 | 0.03 | -0.73 | 0.04 | -0.77 | 0.02 |

| | | | | | | | | | | | | | | | |
|---|---|---|---|---|---|---|---|---|---|---|---|---|---|---|---|
| Cluster-40555.218797 | WOX | 0.52 | 0.19 | 0.5 | 0.21 | -0.59 | 0.12 | 0.37 | 0.37 | 0.4 | 0.32 | 0.42 | 0.3 | 0.42 | 0.3 |
| Cluster-40555.143406 | WRKY | -0.7 | 0.05 | -0.85 | 0.01 | 0.53 | 0.18 | -0.78 | 0.02 | -0.79 | 0.02 | -0.75 | 0.03 | -0.79 | 0.02 |
| Cluster-40555.147227 | WRKY | -0.83 | 0.01 | -0.85 | 0.01 | 0.97 | 0 | -0.83 | 0.01 | -0.78 | 0.02 | -0.79 | 0.02 | -0.82 | 0.01 |
| Cluster-40555.183899 | WRKY | -0.56 | 0.15 | -0.67 | 0.07 | 0.29 | 0.49 | -0.58 | 0.13 | -0.59 | 0.12 | -0.54 | 0.16 | -0.58 | 0.13 |
| Cluster-40555.192086 | WRKY | -0.78 | 0.02 | -0.87 | 0.01 | 0.6 | 0.11 | -0.78 | 0.02 | -0.78 | 0.02 | -0.74 | 0.04 | -0.78 | 0.02 |
| Cluster-40555.99532 | WRKY | -0.84 | 0.01 | -0.94 | 0 | 0.72 | 0.04 | -0.83 | 0.01 | -0.82 | 0.01 | -0.8 | 0.02 | -0.84 | 0.01 |
| Cluster-40555.162685 | WRKY | 0.81 | 0.02 | 0.35 | 0.4 | -0.47 | 0.24 | 0.11 | 0.79 | 0.05 | 0.91 | 0.04 | 0.92 | 0.1 | 0.81 |
| Cluster-40555.173367 | WRKY | 0.95 | 0 | 0.73 | 0.04 | -0.8 | 0.02 | 0.53 | 0.18 | 0.48 | 0.23 | 0.48 | 0.23 | 0.54 | 0.17 |
| Cluster-40555.182385 | WRKY | 0.74 | 0.03 | 0.41 | 0.31 | -0.57 | 0.14 | 0.14 | 0.75 | 0.1 | 0.82 | 0.08 | 0.85 | 0.17 | 0.69 |
| Cluster-40555.182392 | WRKY | 0.84 | 0.01 | 0.4 | 0.33 | -0.53 | 0.18 | 0.15 | 0.72 | 0.08 | 0.84 | 0.08 | 0.85 | 0.15 | 0.73 |
| Cluster-40555.185107 | WRKY | 0.76 | 0.03 | 0.31 | 0.46 | -0.58 | 0.13 | 0.11 | 0.8 | 0.04 | 0.93 | 0.04 | 0.92 | 0.11 | 0.8 |
| Cluster-40555.199963 | WRKY | 0.89 | 0 | 0.52 | 0.19 | -0.63 | 0.09 | 0.28 | 0.5 | 0.22 | 0.6 | 0.22 | 0.6 | 0.28 | 0.5 |
| Cluster-40555.203890 | WRKY | 0.86 | 0.36 | 0.52 | 0.19 | -0.64 | 0.09 | 0.28 | 0.5 | 0.24 | 0.57 | 0.23 | 0.58 | 0.29 | 0.48 |
| Cluster-40555.216207 | WRKY | 0.79 | 0.44 | 0.37 | 0.36 | -0.5 | 0.21 | 0.11 | 0.79 | 0.06 | 0.89 | 0.05 | 0.9 | 0.12 | 0.77 |
| Cluster-40555.202559 | ZF-HD | 0.75 | 0.44 | 0.32 | 0.44 | -0.53 | 0.18 | 0.08 | 0.86 | 0.02 | 0.96 | 0.02 | 0.96 | 0.09 | 0.84 |

B: Spearman correlation analysis

| GeneID | Type | Cluster-40555.170438 | | Cluster-40555.182940 | | Cluster-40555.226813 | | Cluster-40555.154499 | | Cluster-40555.182911 | | Cluster-40555.183520 | | Cluster-40555.186665 | |
|---|---|---|---|---|---|---|---|---|---|---|---|---|---|---|---|
| | | r | p | r | p | r | p | r | p | r | p | r | p | r | p |
| Cluster-40555.170438 | ANR | 1 | 0 | 0.76 | 0.04 | -0.64 | 0.1 | 0.79 | 0.03 | 0.74 | 0.05 | 0.76 | 0.04 | 0.81 | 0.02 |
| Cluster-40555.182940 | ANR | 0.76 | 0.04 | 1 | 0 | -0.64 | 0.1 | 0.93 | 0 | 0.93 | 0 | 0.95 | 0 | 0.95 | 0 |
| Cluster-40555.226813 | ANR | -0.64 | 0.1 | -0.64 | 0.1 | 1 | 0 | -0.62 | 0.11 | -0.67 | 0.08 | -0.6 | 0.13 | -0.64 | 0.1 |
| Cluster-40555.154499 | ANS | 0.79 | 0.03 | 0.93 | 0 | -0.62 | 0.11 | 1 | 0 | 0.95 | 0 | 0.98 | 0 | 0.98 | 0 |
| Cluster-40555.182911 | ANS | 0.74 | 0.05 | 0.93 | 0 | -0.67 | 0.08 | 0.95 | 0 | 1 | 0 | 0.98 | 0 | 0.93 | 0 |
| Cluster-40555.183520 | ANS | 0.76 | 0.04 | 0.95 | 0 | -0.6 | 0.13 | 0.98 | 0 | 0.98 | 0 | 1 | 0 | 0.95 | 0 |
| Cluster-40555.186665 | ANS | 0.81 | 0.02 | 0.95 | 0 | -0.64 | 0.1 | 0.98 | 0 | 0.93 | 0 | 0.95 | 0 | 1 | 0 |
| Cluster-40555.169774 | AP2 | 0.83 | 0.02 | 0.52 | 0.2 | -0.52 | 0.2 | 0.64 | 0.1 | 0.64 | 0.1 | 0.67 | 0.08 | 0.6 | 0.13 |
| Cluster-40555.209356 | BES1 | 0.93 | 0 | 0.64 | 0.1 | -0.74 | 0.05 | 0.76 | 0.04 | 0.74 | 0.05 | 0.71 | 0.06 | 0.74 | 0.05 |
| Cluster-40555.146730 | bHLH | -0.78 | 0.02 | -0.92 | 0 | 0.71 | 0.05 | -0.97 | 0 | -0.97 | 0 | -0.95 | 0 | -0.97 | 0 |
| Cluster-40555.177017 | bHLH | -0.79 | 0.03 | -0.83 | 0.02 | 0.67 | 0.08 | -0.86 | 0.01 | -0.9 | 0 | -0.88 | 0.01 | -0.79 | 0.03 |
| Cluster-40555.184841 | bHLH | -0.74 | 0.05 | -0.6 | 0.13 | 0.95 | 0 | -0.6 | 0.13 | -0.62 | 0.11 | -0.57 | 0.15 | -0.64 | 0.1 |
| Cluster-40555.201242 | bHLH | -0.95 | 0 | -0.76 | 0.04 | 0.6 | 0.13 | -0.79 | 0.03 | -0.79 | 0.03 | -0.81 | 0.02 | -0.76 | 0.04 |
| Cluster-40555.234751 | bHLH | -0.93 | 0 | -0.79 | 0.03 | 0.62 | 0.11 | -0.76 | 0.04 | -0.76 | 0.04 | -0.79 | 0.03 | -0.74 | 0.05 |
| Cluster-40555.272493 | bHLH | -0.86 | 0.01 | -0.95 | 0 | 0.64 | 0.1 | -0.9 | 0 | -0.83 | 0.02 | -0.88 | 0.01 | -0.95 | 0 |
| Cluster-40555.158717 | bHLH | 0.95 | 0 | 0.73 | 0.04 | -0.68 | 0.06 | 0.78 | 0.02 | 0.8 | 0.02 | 0.78 | 0.02 | 0.75 | 0.03 |
| Cluster-40555.160644 | bHLH | 0.9 | 0 | 0.62 | 0.11 | -0.69 | 0.07 | 0.74 | 0.05 | 0.69 | 0.07 | 0.71 | 0.06 | 0.71 | 0.06 |
| Cluster-40555.182851 | bHLH | 0.93 | 0 | 0.68 | 0.06 | -0.73 | 0.04 | 0.76 | 0.03 | 0.78 | 0.02 | 0.76 | 0.03 | 0.73 | 0.04 |
| Cluster-40555.187730 | bHLH | 0.8 | 0.02 | 0.86 | 0.01 | -0.7 | 0.05 | 0.89 | 0 | 0.83 | 0.01 | 0.89 | 0 | 0.86 | 0.01 |
| Cluster-40555.188801 | bHLH | 0.69 | 0.07 | 0.38 | 0.36 | -0.74 | 0.05 | 0.5 | 0.22 | 0.43 | 0.3 | 0.45 | 0.27 | 0.45 | 0.27 |
| Cluster-40555.198935 | bHLH | 0.76 | 0.04 | 0.4 | 0.33 | -0.31 | 0.46 | 0.38 | 0.36 | 0.43 | 0.3 | 0.45 | 0.27 | 0.43 | 0.3 |
| Cluster-40555.201846 | bHLH | 0.83 | 0.02 | 0.55 | 0.17 | -0.83 | 0.02 | 0.67 | 0.08 | 0.64 | 0.1 | 0.62 | 0.11 | 0.64 | 0.1 |
| Cluster-40555.201847 | bHLH | 0.68 | 0.06 | 0.11 | 0.8 | -0.32 | 0.43 | 0.3 | 0.47 | 0.2 | 0.63 | 0.23 | 0.59 | 0.3 | 0.47 |
| Cluster-40555.214608 | bHLH | 0.93 | 0 | 0.65 | 0.08 | -0.68 | 0.06 | 0.77 | 0.03 | 0.71 | 0.05 | 0.73 | 0.04 | 0.74 | 0.03 |
| Cluster-40555.226537 | bHLH | 0.57 | 0.15 | 0.33 | 0.43 | -0.64 | 0.1 | 0.26 | 0.54 | 0.31 | 0.46 | 0.33 | 0.43 | 0.29 | 0.5 |
| Cluster-40555.230888 | bHLH | 0.71 | 0.05 | 0.81 | 0.01 | -0.86 | 0.01 | 0.86 | 0.01 | 0.91 | 0 | 0.86 | 0.01 | 0.81 | 0.01 |
| Cluster-40555.239070 | bHLH | 0.79 | 0.03 | 0.45 | 0.27 | -0.64 | 0.1 | 0.43 | 0.3 | 0.31 | 0.46 | 0.33 | 0.43 | 0.5 | 0.22 |
| Cluster-40555.97572 | bHLH | 0.86 | 0.01 | 0.43 | 0.3 | -0.45 | 0.27 | 0.55 | 0.17 | 0.5 | 0.22 | 0.52 | 0.2 | 0.57 | 0.15 |

| | | | | | | | | | | | | | |
|---|---|---|---|---|---|---|---|---|---|---|---|---|---|
| Cluster-40555.171315 | bZIP | -0.93 | 0 | -0.74 | 0.05 | 0.71 | 0.06 | -0.76 | 0.04 | -0.76 | 0.04 | -0.74 | 0.05 | -0.74 | 0.05 |
| Cluster-40555.203533 | bZIP | -0.79 | 0.03 | -0.93 | 0 | 0.62 | 0.11 | -1 | 0 | -0.95 | 0 | -0.98 | 0 | -0.98 | 0 |
| Cluster-40555.218108 | bZIP | -0.85 | 0.01 | -0.75 | 0.03 | 0.83 | 0.01 | -0.78 | 0.02 | -0.71 | 0.05 | -0.71 | 0.05 | -0.85 | 0.01 |
| Cluster-40555.178265 | bZIP | 0.83 | 0.02 | 0.64 | 0.1 | -0.74 | 0.05 | 0.62 | 0.11 | 0.64 | 0.1 | 0.67 | 0.08 | 0.64 | 0.1 |
| Cluster-40555.179322 | bZIP | 0.93 | 0 | 0.78 | 0.02 | -0.73 | 0.04 | 0.71 | 0.05 | 0.68 | 0.06 | 0.71 | 0.05 | 0.73 | 0.04 |
| Cluster-40555.195157 | bZIP | 0.74 | 0.05 | 0.93 | 0 | -0.71 | 0.06 | 0.95 | 0 | 0.95 | 0 | 0.93 | 0 | 0.93 | 0 |
| Cluster-40555.219797 | bZIP | 0.71 | 0.06 | 0.88 | 0.01 | -0.45 | 0.27 | 0.98 | 0 | 0.9 | 0 | 0.95 | 0 | 0.95 | 0 |
| Cluster-40555.142929 | C2H2 | -0.81 | 0.02 | -0.95 | 0 | 0.64 | 0.1 | -0.98 | 0 | -0.93 | 0 | -0.95 | 0 | -1 | 0 |
| Cluster-40555.175851 | C2H2 | -0.63 | 0.09 | -0.68 | 0.06 | 0.99 | 0 | -0.65 | 0.08 | -0.68 | 0.06 | -0.62 | 0.1 | -0.68 | 0.06 |
| Cluster-40555.141702 | C2H2 | 0.31 | 0.46 | -0.26 | 0.54 | -0.33 | 0.43 | -0.14 | 0.75 | -0.14 | 0.75 | -0.17 | 0.7 | -0.17 | 0.7 |
| Cluster-40555.150134 | C2H2 | 0.62 | 0.11 | 0.21 | 0.62 | -0.6 | 0.13 | 0.19 | 0.66 | 0.14 | 0.75 | 0.17 | 0.7 | 0.29 | 0.5 |
| Cluster-40555.180184 | C2H2 | 0.45 | 0.27 | -0.12 | 0.79 | -0.14 | 0.75 | -0.05 | 0.93 | -0.05 | 0.93 | -0.02 | 0.98 | -0.02 | 0.98 |
| Cluster-40555.180185 | C2H2 | 0.91 | 0 | 0.71 | 0.05 | -0.76 | 0.03 | 0.74 | 0.04 | 0.71 | 0.05 | 0.74 | 0.04 | 0.71 | 0.05 |
| Cluster-40555.180186 | C2H2 | 0.45 | 0.27 | -0.12 | 0.79 | -0.14 | 0.75 | -0.05 | 0.93 | -0.05 | 0.93 | -0.02 | 0.98 | -0.02 | 0.98 |
| Cluster-40555.194927 | C2H2 | 0.31 | 0.46 | -0.21 | 0.62 | -0.24 | 0.58 | -0.14 | 0.75 | -0.14 | 0.75 | -0.12 | 0.79 | -0.17 | 0.7 |
| Cluster-40555.204350 | C3H | -0.79 | 0.03 | -0.98 | 0 | 0.62 | 0.11 | -0.95 | 0 | -0.95 | 0 | -0.98 | 0 | -0.98 | 0 |
| Cluster-40555.190917 | C3H | 0.76 | 0.03 | 0.9 | 0 | -0.66 | 0.08 | 0.98 | 0 | 0.95 | 0 | 0.98 | 0 | 0.95 | 0 |
| Cluster-40555.212569 | C3H | 0.79 | 0.02 | 0.79 | 0.02 | -0.73 | 0.04 | 0.65 | 0.08 | 0.66 | 0.08 | 0.71 | 0.05 | 0.69 | 0.06 |
| Cluster-40555.233380 | C3H | 0.78 | 0.02 | 0.99 | 0 | -0.66 | 0.08 | 0.95 | 0 | 0.92 | 0 | 0.95 | 0 | 0.97 | 0 |
| Cluster-40555.235817 | C3H | 0.81 | 0.02 | 0.86 | 0.01 | -0.79 | 0.03 | 0.79 | 0.03 | 0.88 | 0.01 | 0.81 | 0.02 | 0.81 | 0.02 |
| Cluster-40555.191531 | CO-like | 0.91 | 0 | 0.71 | 0.05 | -0.86 | 0.01 | 0.68 | 0.06 | 0.71 | 0.05 | 0.68 | 0.06 | 0.71 | 0.05 |
| Cluster-40555.206298 | CO-like | 0.38 | 0.36 | -0.12 | 0.79 | -0.5 | 0.22 | -0.1 | 0.84 | -0.1 | 0.84 | -0.12 | 0.79 | -0.05 | 0.93 |
| Cluster-40555.233365 | CO-like | 0.71 | 0.05 | 0.42 | 0.3 | -0.87 | 0 | 0.42 | 0.3 | 0.4 | 0.33 | 0.37 | 0.37 | 0.47 | 0.24 |
| Cluster-40555.179654 | DBB | -0.69 | 0.07 | -0.69 | 0.07 | 0.9 | 0 | -0.62 | 0.11 | -0.67 | 0.08 | -0.64 | 0.1 | -0.69 | 0.07 |
| Cluster-40555.194971 | DBB | -0.64 | 0.1 | -0.6 | 0.13 | 0.95 | 0 | -0.67 | 0.08 | -0.71 | 0.06 | -0.64 | 0.1 | -0.64 | 0.1 |
| Cluster-40555.194972 | DBB | -0.76 | 0.04 | -0.95 | 0 | 0.64 | 0.1 | -0.98 | 0 | -0.93 | 0 | -0.95 | 0 | -0.95 | 0 |
| Cluster-40555.194976 | DBB | 0.71 | 0.05 | 0.81 | 0.01 | -0.86 | 0.01 | 0.86 | 0.01 | 0.91 | 0 | 0.86 | 0.01 | 0.81 | 0.01 |
| Cluster-40555.199338 | DBB | 0.73 | 0.04 | 0.95 | 0 | -0.71 | 0.05 | 0.95 | 0 | 0.97 | 0 | 0.95 | 0 | 0.92 | 0 |
| Cluster-40555.183211 | Dof | -0.76 | 0.04 | -0.62 | 0.11 | 0.83 | 0.02 | -0.55 | 0.17 | -0.55 | 0.17 | -0.57 | 0.15 | -0.57 | 0.15 |
| Cluster-40555.188528 | Dof | -0.95 | 0 | -0.86 | 0.01 | 0.6 | 0.13 | -0.81 | 0.02 | -0.79 | 0.03 | -0.83 | 0.02 | -0.86 | 0.01 |
| Cluster-40555.194651 | Dof | -0.77 | 0.03 | -0.67 | 0.07 | 0.79 | 0.02 | -0.68 | 0.06 | -0.6 | 0.12 | -0.66 | 0.08 | -0.72 | 0.04 |
| Cluster-40555.208868 | Dof | -0.76 | 0.04 | -0.95 | 0 | 0.69 | 0.07 | -0.93 | 0 | -0.98 | 0 | -0.95 | 0 | -0.95 | 0 |

| | | | | | | | | | | | | | | |
|---|---|---|---|---|---|---|---|---|---|---|---|---|---|---|
| Cluster-40555.230137 | Dof | -0.71 | 0.05 | -0.8 | 0.02 | 0.83 | 0.01 | -0.72 | 0.04 | -0.85 | 0.01 | -0.79 | 0.02 | -0.75 | 0.03 |
| Cluster-40555.168102 | Dof | 0.73 | 0.04 | 0.37 | 0.37 | -0.34 | 0.41 | 0.49 | 0.22 | 0.51 | 0.19 | 0.49 | 0.22 | 0.39 | 0.34 |
| Cluster-40555.171911 | Dof | 0.91 | 0 | 0.71 | 0.05 | -0.76 | 0.03 | 0.74 | 0.04 | 0.71 | 0.05 | 0.74 | 0.04 | 0.71 | 0.05 |
| Cluster-40555.161069 | ERF | -0.9 | 0 | -0.86 | 0.01 | 0.74 | 0.05 | -0.83 | 0.02 | -0.83 | 0.02 | -0.81 | 0.02 | -0.9 | 0 |
| Cluster-40555.161377 | ERF | -0.4 | 0.33 | -0.74 | 0.05 | 0.24 | 0.58 | -0.76 | 0.04 | -0.76 | 0.04 | -0.74 | 0.05 | -0.79 | 0.03 |
| Cluster-40555.165988 | ERF | -0.88 | 0 | -0.83 | 0.01 | 0.66 | 0.08 | -0.83 | 0.01 | -0.85 | 0.01 | -0.85 | 0.01 | -0.78 | 0.02 |
| Cluster-40555.192007 | ERF | -0.76 | 0.04 | -0.95 | 0 | 0.69 | 0.07 | -0.93 | 0 | -0.98 | 0 | -0.95 | 0 | -0.95 | 0 |
| Cluster-40555.175287 | ERF | 0.57 | 0.15 | 0.33 | 0.43 | -0.64 | 0.1 | 0.26 | 0.54 | 0.31 | 0.46 | 0.33 | 0.43 | 0.29 | 0.5 |
| Cluster-40555.187700 | ERF | 0.31 | 0.46 | -0.14 | 0.75 | -0.43 | 0.3 | -0.07 | 0.88 | -0.02 | 0.98 | -0.05 | 0.93 | -0.12 | 0.79 |
| Cluster-40555.188580 | ERF | 0.79 | 0.02 | 0.79 | 0.02 | -0.94 | 0 | 0.76 | 0.03 | 0.84 | 0.01 | 0.76 | 0.03 | 0.79 | 0.02 |
| Cluster-40555.201722 | ERF | 0.86 | 0.01 | 0.57 | 0.15 | -0.86 | 0.01 | 0.64 | 0.1 | 0.62 | 0.11 | 0.6 | 0.13 | 0.67 | 0.08 |
| Cluster-40555.241796 | FAR1 | -0.71 | 0.05 | -0.61 | 0.11 | 0.88 | 0 | -0.66 | 0.08 | -0.63 | 0.09 | -0.63 | 0.09 | -0.61 | 0.11 |
| Cluster-40555.209479 | FAR1 | 0.71 | 0.05 | 0.85 | 0.01 | -0.81 | 0.02 | 0.85 | 0.01 | 0.9 | 0 | 0.85 | 0.01 | 0.81 | 0.02 |
| Cluster-40555.240402 | FAR1 | 0.73 | 0.04 | 0.59 | 0.13 | -0.71 | 0.05 | 0.44 | 0.27 | 0.56 | 0.15 | 0.54 | 0.17 | 0.49 | 0.22 |
| Cluster-40555.166373 | G2-like | 0.87 | 0 | 0.55 | 0.16 | -0.65 | 0.08 | 0.46 | 0.25 | 0.44 | 0.28 | 0.46 | 0.25 | 0.55 | 0.16 |
| Cluster-40555.174898 | G2-like | 0.75 | 0.03 | 0.56 | 0.15 | -0.87 | 0 | 0.56 | 0.15 | 0.61 | 0.11 | 0.59 | 0.13 | 0.56 | 0.15 |
| Cluster-40555.183351 | G2-like | 0.33 | 0.43 | -0.24 | 0.58 | -0.26 | 0.54 | -0.12 | 0.79 | -0.17 | 0.7 | -0.14 | 0.75 | -0.14 | 0.75 |
| Cluster-40555.196765 | G2-like | 0.79 | 0.03 | 0.52 | 0.2 | -0.38 | 0.36 | 0.4 | 0.33 | 0.45 | 0.27 | 0.43 | 0.3 | 0.45 | 0.27 |
| Cluster-40555.196766 | G2-like | 0.33 | 0.43 | -0.19 | 0.66 | -0.26 | 0.54 | -0.17 | 0.7 | -0.17 | 0.7 | -0.14 | 0.75 | -0.14 | 0.75 |
| Cluster-40555.233429 | G2-like | 0.79 | 0.02 | 0.46 | 0.25 | -0.57 | 0.14 | 0.55 | 0.16 | 0.52 | 0.19 | 0.55 | 0.16 | 0.46 | 0.25 |
| Cluster-40555.198012 | GATA | -0.95 | 0 | -0.67 | 0.08 | 0.76 | 0.04 | -0.74 | 0.05 | -0.71 | 0.06 | -0.69 | 0.07 | -0.76 | 0.04 |
| Cluster-40555.208893 | GATA | -0.9 | 0 | -0.86 | 0.01 | 0.74 | 0.05 | -0.83 | 0.02 | -0.83 | 0.02 | -0.81 | 0.02 | -0.9 | 0 |
| Cluster-40555.188605 | GATA | 0.86 | 0.01 | 0.62 | 0.11 | -0.74 | 0.05 | 0.69 | 0.07 | 0.74 | 0.05 | 0.71 | 0.06 | 0.67 | 0.08 |
| Cluster-40555.208892 | GATA | 0.86 | 0.01 | 0.76 | 0.04 | -0.69 | 0.07 | 0.83 | 0.02 | 0.69 | 0.07 | 0.76 | 0.04 | 0.86 | 0.01 |
| Cluster-40555.158020 | GRAS | 0.61 | 0.11 | 0.34 | 0.42 | -0.51 | 0.19 | 0.26 | 0.53 | 0.34 | 0.42 | 0.36 | 0.38 | 0.3 | 0.47 |
| Cluster-40555.204370 | GRAS | 0.81 | 0.02 | 0.5 | 0.22 | -0.6 | 0.13 | 0.62 | 0.11 | 0.67 | 0.08 | 0.64 | 0.1 | 0.57 | 0.15 |
| Cluster-40555.203336 | GRF | 0.85 | 0.01 | 0.9 | 0 | -0.76 | 0.03 | 0.78 | 0.02 | 0.76 | 0.03 | 0.78 | 0.02 | 0.85 | 0.01 |
| Cluster-40555.184232 | HD-ZIP | -0.95 | 0 | -0.76 | 0.04 | 0.64 | 0.1 | -0.79 | 0.03 | -0.74 | 0.05 | -0.76 | 0.04 | -0.76 | 0.04 |
| Cluster-40555.206892 | HD-ZIP | -0.76 | 0.04 | -0.95 | 0 | 0.74 | 0.05 | -0.93 | 0 | -0.93 | 0 | -0.9 | 0 | -0.95 | 0 |
| Cluster-40555.225157 | HD-ZIP | -0.75 | 0.03 | -0.56 | 0.15 | 0.87 | 0 | -0.59 | 0.13 | -0.56 | 0.15 | -0.56 | 0.15 | -0.56 | 0.15 |
| Cluster-40555.170890 | HD-ZIP | 0.52 | 0.2 | 0.29 | 0.5 | -0.69 | 0.07 | 0.26 | 0.54 | 0.36 | 0.39 | 0.33 | 0.43 | 0.24 | 0.58 |
| Cluster-40555.180440 | HD-ZIP | 0.81 | 0.02 | 0.9 | 0 | -0.74 | 0.05 | 0.86 | 0.01 | 0.93 | 0 | 0.88 | 0.01 | 0.9 | 0 |

| | | | | | | | | | | | | | | | |
|---|---|---|---|---|---|---|---|---|---|---|---|---|---|---|---|
| Cluster-40555.188403 | HD-ZIP | 0.86 | 0.01 | 0.52 | 0.2 | -0.38 | 0.36 | 0.55 | 0.17 | 0.57 | 0.15 | 0.6 | 0.13 | 0.57 | 0.15 |
| Cluster-40555.195704 | HD-ZIP | 0.76 | 0.04 | 0.57 | 0.15 | -0.88 | 0.01 | 0.55 | 0.17 | 0.6 | 0.13 | 0.57 | 0.15 | 0.57 | 0.15 |
| Cluster-40555.213365 | HD-ZIP | 0.93 | 0 | 0.73 | 0.04 | -0.78 | 0.02 | 0.73 | 0.04 | 0.7 | 0.05 | 0.73 | 0.04 | 0.73 | 0.04 |
| Cluster-40555.306180 | HD-ZIP | 0.73 | 0.04 | 0.23 | 0.58 | -0.55 | 0.16 | 0.23 | 0.58 | 0.26 | 0.53 | 0.23 | 0.58 | 0.23 | 0.58 |
| Cluster-40555.172251 | HSF | -0.81 | 0.02 | -0.57 | 0.15 | 0.83 | 0.02 | -0.6 | 0.13 | -0.55 | 0.17 | -0.57 | 0.15 | -0.62 | 0.11 |
| Cluster-40555.198525 | HSF | -0.9 | 0 | -0.81 | 0.02 | 0.74 | 0.05 | -0.76 | 0.04 | -0.83 | 0.02 | -0.79 | 0.03 | -0.81 | 0.02 |
| Cluster-40555.225819 | HSF | -0.67 | 0.08 | -0.76 | 0.04 | 0.83 | 0.02 | -0.69 | 0.07 | -0.83 | 0.02 | -0.76 | 0.04 | -0.67 | 0.08 |
| Cluster-40555.183779 | HSF | 0.71 | 0.05 | 0.85 | 0.01 | -0.81 | 0.02 | 0.85 | 0.01 | 0.9 | 0 | 0.85 | 0.01 | 0.81 | 0.02 |
| Cluster-40555.192124 | HSF | 0.95 | 0 | 0.76 | 0.04 | -0.6 | 0.13 | 0.79 | 0.03 | 0.79 | 0.03 | 0.81 | 0.02 | 0.76 | 0.04 |
| Cluster-40555.198527 | HSF | 0.71 | 0.06 | 0.57 | 0.15 | -0.88 | 0.01 | 0.55 | 0.17 | 0.6 | 0.13 | 0.57 | 0.15 | 0.52 | 0.2 |
| Cluster-40555.157232 | LSD | 0.83 | 0.02 | 0.74 | 0.05 | -0.76 | 0.04 | 0.62 | 0.11 | 0.62 | 0.11 | 0.64 | 0.1 | 0.64 | 0.1 |
| Cluster-40555.158078 | MIKC_MADS | 0.85 | 0.01 | 0.61 | 0.11 | -0.9 | 0 | 0.63 | 0.09 | 0.61 | 0.11 | 0.59 | 0.13 | 0.66 | 0.08 |
| Cluster-40555.175507 | MIKC_MADS | 0.79 | 0.02 | 0.46 | 0.25 | -0.57 | 0.14 | 0.55 | 0.16 | 0.52 | 0.19 | 0.55 | 0.16 | 0.46 | 0.25 |
| Cluster-40555.190290 | MIKC_MADS | 0.28 | 0.5 | -0.2 | 0.63 | -0.38 | 0.35 | -0.23 | 0.59 | -0.18 | 0.67 | -0.2 | 0.63 | -0.23 | 0.59 |
| Cluster-40555.89704 | M-type_MADS | 0.66 | 0.08 | 0.39 | 0.34 | -0.67 | 0.07 | 0.24 | 0.57 | 0.33 | 0.43 | 0.3 | 0.46 | 0.33 | 0.43 |
| Cluster-40555.200316 | MYB | -0.71 | 0.05 | -0.71 | 0.05 | 0.94 | 0 | -0.71 | 0.05 | -0.68 | 0.06 | -0.68 | 0.06 | -0.71 | 0.05 |
| Cluster-40555.131430 | MYB | 0.64 | 0.1 | 0.88 | 0.01 | -0.33 | 0.43 | 0.9 | 0 | 0.81 | 0.02 | 0.88 | 0.01 | 0.93 | 0 |
| Cluster-40555.174498 | MYB | 0.98 | 0 | 0.73 | 0.04 | -0.68 | 0.06 | 0.76 | 0.03 | 0.73 | 0.04 | 0.76 | 0.03 | 0.78 | 0.02 |
| Cluster-40555.179825 | MYB | 0.45 | 0.27 | -0.17 | 0.7 | -0.24 | 0.58 | -0.05 | 0.93 | -0.05 | 0.93 | -0.07 | 0.88 | -0.02 | 0.98 |
| Cluster-40555.184294 | MYB | 0.33 | 0.43 | -0.19 | 0.66 | -0.26 | 0.54 | -0.17 | 0.7 | -0.17 | 0.7 | -0.14 | 0.75 | -0.14 | 0.75 |
| Cluster-40555.184530 | MYB | 0.71 | 0.05 | 0.14 | 0.75 | -0.16 | 0.7 | 0.22 | 0.6 | 0.19 | 0.65 | 0.22 | 0.6 | 0.22 | 0.6 |
| Cluster-40555.189597 | MYB | 0.29 | 0.5 | -0.24 | 0.58 | -0.31 | 0.46 | -0.17 | 0.7 | -0.12 | 0.79 | -0.14 | 0.75 | -0.19 | 0.66 |
| Cluster-40555.211498 | MYB | 0.4 | 0.33 | -0.17 | 0.7 | -0.19 | 0.66 | -0.05 | 0.93 | 0 | 1 | -0.02 | 0.98 | -0.07 | 0.88 |
| Cluster-40555.215102 | MYB | 0.97 | 0 | 0.75 | 0.03 | -0.71 | 0.05 | 0.75 | 0.03 | 0.78 | 0.02 | 0.75 | 0.03 | 0.78 | 0.02 |
| Cluster-40555.191840 | MYB_related | 0.73 | 0.04 | 0.23 | 0.58 | -0.55 | 0.16 | 0.23 | 0.58 | 0.26 | 0.53 | 0.23 | 0.58 | 0.23 | 0.58 |
| Cluster-40555.151396 | NAC | -0.75 | 0.03 | -0.56 | 0.15 | 0.83 | 0.01 | -0.59 | 0.13 | -0.61 | 0.11 | -0.61 | 0.11 | -0.56 | 0.15 |
| Cluster-40555.151794 | NAC | -0.74 | 0.05 | -0.55 | 0.17 | 0.86 | 0.01 | -0.57 | 0.15 | -0.62 | 0.11 | -0.6 | 0.13 | -0.55 | 0.17 |
| Cluster-40555.194173 | NAC | -0.94 | 0 | -0.89 | 0 | 0.71 | 0.05 | -0.94 | 0 | -0.91 | 0 | -0.91 | 0 | -0.94 | 0 |
| Cluster-40555.197659 | NAC | -0.71 | 0.06 | -0.71 | 0.06 | 0.71 | 0.06 | -0.67 | 0.08 | -0.67 | 0.08 | -0.71 | 0.06 | -0.62 | 0.11 |
| Cluster-40555.200046 | NAC | -0.68 | 0.06 | -0.73 | 0.04 | 0.73 | 0.04 | -0.69 | 0.06 | -0.71 | 0.05 | -0.74 | 0.03 | -0.63 | 0.09 |
| Cluster-40555.202205 | NAC | -0.81 | 0.02 | -0.71 | 0.06 | 0.74 | 0.05 | -0.64 | 0.1 | -0.64 | 0.1 | -0.67 | 0.08 | -0.62 | 0.11 |
| Cluster-40555.225773 | NAC | -0.79 | 0.03 | -0.98 | 0 | 0.67 | 0.08 | -0.95 | 0 | -0.9 | 0 | -0.93 | 0 | -0.98 | 0 |

| | | | | | | | | | | | | | | | |
|---|---|---|---|---|---|---|---|---|---|---|---|---|---|---|---|
| Cluster-40555.163727 | NAC | 0.95 | 0 | 0.71 | 0.05 | -0.76 | 0.03 | 0.73 | 0.04 | 0.76 | 0.03 | 0.73 | 0.04 | 0.76 | 0.03 |
| Cluster-40555.168512 | NAC | 0.83 | 0.02 | 0.4 | 0.33 | -0.43 | 0.3 | 0.57 | 0.15 | 0.52 | 0.2 | 0.55 | 0.17 | 0.55 | 0.17 |
| Cluster-40555.186331 | NAC | 0.83 | 0.01 | 0.68 | 0.06 | -0.78 | 0.02 | 0.61 | 0.11 | 0.63 | 0.09 | 0.66 | 0.08 | 0.63 | 0.09 |
| Cluster-40555.187541 | NAC | 0.98 | 0 | 0.74 | 0.05 | -0.62 | 0.11 | 0.81 | 0.02 | 0.76 | 0.04 | 0.79 | 0.03 | 0.79 | 0.03 |
| Cluster-40555.192230 | NAC | 0.76 | 0.03 | 0.26 | 0.53 | -0.45 | 0.26 | 0.26 | 0.53 | 0.23 | 0.58 | 0.26 | 0.53 | 0.26 | 0.53 |
| Cluster-40555.192486 | NAC | 0.93 | 0 | 0.65 | 0.08 | -0.71 | 0.05 | 0.77 | 0.03 | 0.68 | 0.06 | 0.71 | 0.05 | 0.74 | 0.03 |
| Cluster-40555.202381 | NAC | 0.45 | 0.27 | -0.12 | 0.79 | -0.14 | 0.75 | -0.05 | 0.93 | -0.05 | 0.93 | -0.02 | 0.98 | -0.02 | 0.98 |
| Cluster-40555.224037 | NAC | 0.94 | 0 | 0.74 | 0.04 | -0.79 | 0.02 | 0.71 | 0.05 | 0.68 | 0.06 | 0.71 | 0.05 | 0.74 | 0.04 |
| Cluster-40555.206159 | NF-YA | 0.43 | 0.29 | -0.04 | 0.93 | -0.29 | 0.49 | -0.08 | 0.84 | 0.01 | 0.98 | -0.01 | 0.98 | -0.02 | 0.96 |
| Cluster-40555.203810 | NF-YC | 0.31 | 0.46 | -0.21 | 0.62 | -0.33 | 0.43 | -0.19 | 0.66 | -0.14 | 0.75 | -0.17 | 0.7 | -0.17 | 0.7 |
| Cluster-40555.196262 | SRS | 0.93 | 0 | 0.74 | 0.05 | -0.67 | 0.08 | 0.76 | 0.04 | 0.81 | 0.02 | 0.79 | 0.03 | 0.74 | 0.05 |
| Cluster-40555.237203 | Trihelix | -0.89 | 0 | -0.94 | 0 | 0.71 | 0.05 | -0.89 | 0 | -0.91 | 0 | -0.91 | 0 | -0.89 | 0 |
| Cluster-40555.218797 | WOX | 0.85 | 0.01 | 0.71 | 0.05 | -0.76 | 0.03 | 0.81 | 0.02 | 0.85 | 0.01 | 0.81 | 0.02 | 0.76 | 0.03 |
| Cluster-40555.143406 | WRKY | -0.75 | 0.03 | -0.95 | 0 | 0.68 | 0.06 | -0.95 | 0 | -0.99 | 0 | -0.97 | 0 | -0.95 | 0 |
| Cluster-40555.147227 | WRKY | -0.81 | 0.01 | -0.71 | 0.05 | 0.86 | 0.01 | -0.81 | 0.01 | -0.76 | 0.03 | -0.76 | 0.03 | -0.81 | 0.01 |
| Cluster-40555.183899 | WRKY | -0.93 | 0 | -0.78 | 0.02 | 0.66 | 0.08 | -0.88 | 0 | -0.85 | 0.01 | -0.85 | 0.01 | -0.83 | 0.01 |
| Cluster-40555.192086 | WRKY | -0.9 | 0 | -0.8 | 0.02 | 0.59 | 0.13 | -0.91 | 0 | -0.85 | 0.01 | -0.89 | 0 | -0.85 | 0.01 |
| Cluster-40555.99532 | WRKY | -0.89 | 0 | -0.94 | 0 | 0.71 | 0.05 | -0.89 | 0 | -0.91 | 0 | -0.91 | 0 | -0.89 | 0 |
| Cluster-40555.162685 | WRKY | 0.61 | 0.11 | 0.2 | 0.63 | -0.22 | 0.61 | 0.25 | 0.55 | 0.31 | 0.45 | 0.34 | 0.42 | 0.23 | 0.59 |
| Cluster-40555.173367 | WRKY | 0.79 | 0.03 | 0.69 | 0.07 | -0.81 | 0.02 | 0.62 | 0.11 | 0.67 | 0.08 | 0.64 | 0.1 | 0.6 | 0.13 |
| Cluster-40555.182385 | WRKY | 0.44 | 0.28 | 0.1 | 0.82 | -0.59 | 0.13 | -0.02 | 0.95 | 0.05 | 0.91 | 0.02 | 0.95 | 0.05 | 0.91 |
| Cluster-40555.182392 | WRKY | 0.76 | 0.03 | 0.26 | 0.53 | -0.45 | 0.26 | 0.26 | 0.53 | 0.23 | 0.58 | 0.26 | 0.53 | 0.26 | 0.53 |
| Cluster-40555.185107 | WRKY | 0.4 | 0.33 | 0.02 | 0.98 | -0.52 | 0.2 | 0 | 1 | 0 | 1 | 0.02 | 0.98 | 0.02 | 0.98 |
| Cluster-40555.199963 | WRKY | 0.91 | 0 | 0.71 | 0.05 | -0.76 | 0.03 | 0.74 | 0.04 | 0.71 | 0.05 | 0.74 | 0.04 | 0.71 | 0.05 |
| Cluster-40555.203890 | WRKY | 0.74 | 0.05 | 0.64 | 0.1 | -0.83 | 0.02 | 0.57 | 0.15 | 0.64 | 0.1 | 0.62 | 0.11 | 0.55 | 0.17 |
| Cluster-40555.216207 | WRKY | 0.56 | 0.15 | 0.1 | 0.82 | -0.24 | 0.56 | 0.07 | 0.86 | 0.17 | 0.69 | 0.15 | 0.73 | 0.12 | 0.77 |
| Cluster-40555.202559 | ZF-HD | 0.31 | 0.46 | -0.2 | 0.62 | -0.33 | 0.43 | -0.19 | 0.66 | -0.14 | 0.75 | -0.17 | 0.7 | -0.17 | 0.7 |

r: correlation coeffcient; p: proability

**Table S4: Selected transcription factors**

| GeneID | TFs | r | p | r | p | r | p | r | p | r | p | r | p | r | p | r | p | r | p | r | p | r | p | r | p |
|---|---|---|---|---|---|---|---|---|---|---|---|---|---|---|---|---|---|---|---|---|---|---|---|---|---|
| Cluster-40555.177017 | bHLH | -0.71 | 0.05 | -0.82 | 0.01 | -0.73 | 0.04 | -0.75 | 0.03 | -0.72 | 0.04 | -0.74 | 0.04 | -0.79 | 0.03 | -0.83 | 0.02 | -0.86 | 0.01 | -0.9 | 0 | -0.88 | 0.01 | -0.79 | 0.03 |
| Cluster-40555.272493 | bHLH | -0.7 | 0.05 | -0.84 | 0.01 | -0.77 | 0.03 | -0.76 | 0.03 | -0.72 | 0.05 | -0.76 | 0.03 | -0.86 | 0.01 | -0.95 | 0 | -0.9 | 0 | -0.83 | 0.02 | -0.88 | 0.01 | -0.95 | 0 |
| Cluster-40555.187730 | bHLH | 0.79 | 0.02 | 0.89 | 0 | 0.92 | 0 | 0.88 | 0 | 0.88 | 0 | 0.9 | 0 | 0.8 | 0.02 | 0.86 | 0.01 | 0.89 | 0 | 0.83 | 0.01 | 0.89 | 0 | 0.86 | 0.01 |
| Cluster-40555.203533 | bZIP | -0.75 | 0.03 | -0.88 | 0 | -0.82 | 0.01 | -0.81 | 0.01 | -0.78 | 0.02 | -0.81 | 0.01 | -0.79 | 0.03 | -0.93 | 0 | -1 | 0 | -0.95 | 0 | -0.98 | 0 | -0.98 | 0 |
| Cluster-40555.218108 | bZIP | -0.86 | 0.01 | -0.91 | 0 | -0.85 | 0.01 | -0.82 | 0.01 | -0.8 | 0.02 | -0.85 | 0.01 | -0.85 | 0.01 | -0.75 | 0.03 | -0.78 | 0.02 | -0.71 | 0.05 | -0.71 | 0.05 | -0.85 | 0.01 |
| Cluster-40555.142929 | C2H2 | -0.78 | 0.02 | -0.88 | 0 | -0.81 | 0.01 | -0.8 | 0.02 | -0.76 | 0.03 | -0.81 | 0.02 | -0.81 | 0.02 | -0.95 | 0 | -0.98 | 0 | -0.93 | 0 | -0.95 | 0 | -1 | 0 |
| Cluster-40555.235817 | C3H | 0.89 | 0 | 0.96 | 0 | 0.86 | 0.01 | 0.84 | 0.01 | 0.83 | 0.01 | 0.88 | 0 | 0.81 | 0.02 | 0.86 | 0.01 | 0.79 | 0.03 | 0.88 | 0.01 | 0.81 | 0.02 | 0.81 | 0.02 |
| Cluster-40555.194976 | DBB | 0.77 | 0.03 | 0.94 | 0 | 0.93 | 0 | 0.93 | 0 | 0.93 | 0 | 0.94 | 0 | 0.71 | 0.05 | 0.81 | 0.01 | 0.86 | 0.01 | 0.91 | 0 | 0.86 | 0.01 | 0.81 | 0.01 |
| Cluster-40555.188528 | Dof | -0.78 | 0.02 | -0.86 | 0.01 | -0.76 | 0.03 | -0.75 | 0.03 | -0.71 | 0.05 | -0.76 | 0.03 | -0.95 | 0 | -0.86 | 0.01 | -0.81 | 0.02 | -0.79 | 0.03 | -0.83 | 0.02 | -0.86 | 0.01 |
| Cluster-40555.230137 | Dof | -0.85 | 0.01 | -0.93 | 0 | -0.85 | 0.01 | -0.83 | 0.01 | -0.81 | 0.01 | -0.86 | 0.01 | -0.71 | 0.05 | -0.8 | 0.02 | -0.72 | 0.04 | -0.85 | 0.01 | -0.79 | 0.02 | -0.75 | 0.03 |
| Cluster-40555.161069 | ERF | -0.77 | 0.02 | -0.85 | 0.01 | -0.77 | 0.03 | -0.75 | 0.03 | -0.71 | 0.05 | -0.77 | 0.03 | -0.9 | 0 | -0.86 | 0.01 | -0.83 | 0.02 | -0.83 | 0.02 | -0.81 | 0.02 | -0.9 | 0 |
| Cluster-40555.165988 | ERF | -0.83 | 0.01 | -0.92 | 0 | -0.82 | 0.01 | -0.81 | 0.01 | -0.79 | 0.02 | -0.83 | 0.01 | -0.88 | 0 | -0.83 | 0.01 | -0.83 | 0.01 | -0.85 | 0.01 | -0.85 | 0.01 | -0.78 | 0.02 |
| Cluster-40555.188580 | ERF | 0.78 | 0.02 | 0.88 | 0 | 0.76 | 0.03 | 0.77 | 0.03 | 0.75 | 0.03 | 0.8 | 0.02 | 0.79 | 0.02 | 0.79 | 0.02 | 0.76 | 0.03 | 0.84 | 0.01 | 0.76 | 0.03 | 0.79 | 0.02 |
| Cluster-40555.208893 | GATA | -0.84 | 0.01 | -0.92 | 0 | -0.82 | 0.01 | -0.79 | 0.02 | -0.76 | 0.03 | -0.83 | 0.01 | -0.9 | 0 | -0.86 | 0.01 | -0.83 | 0.02 | -0.83 | 0.02 | -0.81 | 0.02 | -0.9 | 0 |
| Cluster-40555.203336 | GRF | 0.93 | 0 | 0.96 | 0 | 0.85 | 0.01 | 0.81 | 0.01 | 0.79 | 0.02 | 0.86 | 0.01 | 0.85 | 0.01 | 0.9 | 0 | 0.78 | 0.02 | 0.76 | 0.03 | 0.78 | 0.02 | 0.85 | 0.01 |
| Cluster-40555.184232 | HD-ZIP | -0.92 | 0 | -0.88 | 0 | -0.77 | 0.03 | -0.75 | 0.03 | -0.74 | 0.04 | -0.77 | 0.03 | -0.95 | 0 | -0.76 | 0.04 | -0.79 | 0.03 | -0.74 | 0.05 | -0.76 | 0.04 | -0.76 | 0.04 |
| Cluster-40555.180440 | HD-ZIP | 0.82 | 0.01 | 0.96 | 0 | 0.91 | 0 | 0.89 | 0 | 0.85 | 0.01 | 0.92 | 0 | 0.81 | 0.02 | 0.9 | 0 | 0.86 | 0.01 | 0.93 | 0 | 0.88 | 0.01 | 0.9 | 0 |
| Cluster-40555.183779 | HSF | 0.81 | 0.01 | 0.94 | 0 | 0.86 | 0.01 | 0.86 | 0.01 | 0.85 | 0.01 | 0.87 | 0 | 0.71 | 0.05 | 0.85 | 0.01 | 0.85 | 0.01 | 0.9 | 0 | 0.85 | 0.01 | 0.81 | 0.02 |
| Cluster-40555.194173 | NAC | -0.8 | 0.02 | -0.87 | 0 | -0.8 | 0.02 | -0.78 | 0.02 | -0.76 | 0.03 | -0.8 | 0.02 | -0.94 | 0 | -0.89 | 0 | -0.94 | 0 | -0.91 | 0 | -0.91 | 0 | -0.94 | 0 |
| Cluster-40555.237203 | Trihelix | -0.77 | 0.03 | -0.87 | 0.01 | -0.77 | 0.03 | -0.77 | 0.03 | -0.73 | 0.04 | -0.77 | 0.02 | -0.89 | 0 | -0.94 | 0 | -0.89 | 0 | -0.91 | 0 | -0.91 | 0 | -0.89 | 0 |
| Cluster-40555.143406 | WRKY | -0.7 | 0.05 | -0.85 | 0.01 | -0.78 | 0.02 | -0.79 | 0.02 | -0.75 | 0.03 | -0.79 | 0.02 | -0.75 | 0.03 | -0.95 | 0 | -0.95 | 0 | -0.99 | 0 | -0.97 | 0 | -0.95 | 0 |

| | | | | | | | | | | | | | | | | | | | | | | | |
|---|---|---|---|---|---|---|---|---|---|---|---|---|---|---|---|---|---|---|---|---|---|---|---|
| Cluster-40555.147227 | WRKY | -0.83 | 0.01 | -0.85 | 0.01 | -0.83 | 0.01 | -0.78 | 0.02 | -0.79 | 0.02 | -0.82 | 0.01 | -0.81 | 0.01 | -0.71 | 0.05 | -0.81 | 0.01 | -0.76 | 0.03 | -0.76 | 0.03 | -0.81 | 0.01 |
| Cluster-40555.192086 | WRKY | -0.78 | 0.02 | -0.87 | 0.01 | -0.78 | 0.02 | -0.78 | 0.02 | -0.74 | 0.04 | -0.78 | 0.02 | -0.9 | 0 | -0.8 | 0.02 | -0.91 | 0 | -0.85 | 0.01 | -0.89 | 0 | -0.85 | 0.01 |
| Cluster-40555.99532 | WRKY | -0.84 | 0.01 | -0.94 | 0 | -0.83 | 0.01 | -0.82 | 0.01 | -0.8 | 0.02 | -0.84 | 0.01 | -0.89 | 0 | -0.94 | 0 | -0.89 | 0 | -0.91 | 0 | -0.91 | 0 | -0.89 | 0 |

Selection criterion: $|r| \geq 0.7$，$p \leq 0.05$; r: correlation coeffcient; p: proability

# Table S5 Primer sequences used in the present study

| Primers | Sequence (5'-3', the restriction sites were underlined) | Purpose | Restriction sites |
|---|---|---|---|
| LcbHLH92-F | ACACAGACCGCAGCAGCAACTCATC | gene amplification | ------ |
| LcbHLH92-R | ACAACCAGCAGTACGCAGGCAAGG | gene amplification | ------ |
| pSN1301-LcbHLH92a/LcbHLH92b-F | CCCCTCTAGAATGCAGATGGACTCCTACTACTTC | genetic transformation | *Xba I* |
| pSN1301-LcbHLH92a/LcbHLH92b-R | CCCGGTACCTCAGAAAACATTTTGCACGTGTG | genetic transformation | *Kpn I* |
| pCAMBIA1302-LcbHLH92a/LcbHLH92b-F | CCCCAGATCTATGCAGATGGACTCCTACTACTTC | subcellular location | *Bgl II* |
| pCAMBIA1302-LcbHLH92a/LcbHLH92b-R | CCCCACTAGTGAAAACATTTTGCACGTGTGATG | subcellular location | *Spe I* |
| pRT-BD-LcbHLH92a/LcbHLH92b-F | CCCCTCTAGAATGCAGATGGACTCCTACTACTTC | transcriptional activation activity (in protoplast) | *Xba I* |
| pRT-BD-LcbHLH92a/LcbHLH92b-R | CCCGGTACCTCAGAAAACATTTTGCACGTGTG | transcriptional activation activity | *Kpn I* |
| pBridge-LcbHLH92a/LcbHLH92b-F | CCCCGAATTCATGCAGATGGACTCCTACTACTTC | transcriptional activation activity (in yeast) | *EcoR I* |
| pBridge-LcbHLH92a/LcbHLH92b-R | GGGTCTGCAGGAAAACATTTTGCACGTGTGATG | transcriptional activation activity | *Pst I* |
| pET30a-LcbHLH92a/LcbHLH92b-F | CCCCGAATTCATGCAGATGGACTCCTACTACTTC | prokaryotic expression | *EcoR I* |
| pET30a-LcbHLH92a/LcbHLH92b-R | GGGGAAGCTTGAAAACATTTTGCACGTGTGATG | prokaryotic expression | *HindIII* |
| qPCR-AtACTIN-F | TGCTGACCGTATGAGCAAAG | expression analysis | ------ |
| qPCR-AtACTIN-R | GATTGATCCTCCGATCCAGA | expression analysis | ------ |
| qPCR-AtDFR-F | AGCGGAGAAAGCAGCGTGGGATT | expression analysis | ------ |
| qPCR-AtDFR-R | GCGGTGATAAGGCTAGGCGGCATAG | expression analysis | ------ |
| qPCR-AtANS-F(LDOX) | CGTGGGTTGGTGAATAAGGAGAAGGTTAGG | expression analysis | ------ |
| qPCR-AtANS-R(LDOX) | CGTGGAGGAAACTTAGCCGGAGACT | expression analysis | ------ |
| qPCR-AtANR-F | CTCCTCTCCGATCCTCCGAGCAGTT | expression analysis | ------ |
| qPCR-AtANR-R | CACAGCAAATGTAGCGACCAGAAGCAGTT | expression analysis | ------ |
| qPCR-LcbHLH92a-F | GCACCACCAGCAACAGCAGCAG | expression pattern | ------ |
| qPCR-LcbHLH92a-R | TGGCGTAGAGGTCGGCGTAGC | expression pattern | ------ |
| qPCR-LcbHLH92b-F | GGAAGAGCGATTGACGACGGAGATGAATGT | expression pattern | ------ |
| qPCR-LcbHLH92b-R | AGCCTCCTCCACTGCCTTTTCTACCT | expression pattern | ------ |
| qPCR-LcACTIN-F | GTGCTTTCCCTCTATGCAAGTGGT | expression pattern | ------ |
| qPCR-LcACTIN-R | CTGTTCTTGGCAGTCTCCAGCTC | expression pattern | ------ |
| BP-F | CTGCACACGTGTCGCCTCCCATATC | EMSA | ------ |
| BP-R | GATATGGGAGGCGACACGTGTGCAG | EMSA | ------ |
| qPCR-JAZ1-CHIP-F1(JAZ1P1) | AGCAGCGTATAAGAGCCCTGGATACTGAAAT | CHIP | ------ |
| qPCR-JAZ1-CHIP-R1 | AAAGAAGCAAAGAAGAAGGAAACTACAAGGAAAGAAGGT | CHIP | ------ |
| qPCR-JAZ1-CHIP-F2(JAZ1P2) | AAGGAGGGCAAACTCGTAATTTCAAAG | CHIP | ------ |
| qPCR-JAZ1-CHIP-R2 | GGAATGTGTCTCGGTTCACTGTTC | CHIP | ------ |
| qPCR-JAZ2-CHIP-F1(JAZ2P1) | CGGCTATTGTGCGTAGGAGAAC | CHIP | ------ |
| qPCR-JAZ2-CHIP-R1 | TTGTTGGTGTCGTAGTCAGAGTAATCA | CHIP | ------ |
| qPCR-JAZ2-CHIP-F2(JAZ2P2) | TGATTACTCTGACTACGACACCAACAAG | CHIP | ------ |
| qPCR-JAZ2-CHIP-R2 | AGCACGTCTCCTCCATGAATCG | CHIP | ------ |
| qPCR-JAZ8-CHIP-F1(JAZ8P1) | GAAATGCCTCCAAAACCGCCGTAGAAA | CHIP | ------ |
| qPCR-JAZ8-CHIP-R1 | GGACAGAGAGACAGAACACAACTTGCTTCA | CHIP | ------ |
| qPCR-JAZ8-CHIP-F2(JAZ8P2) | GCGTAATGCGAATGGTCAGACA | CHIP | ------ |
| qPCR-JAZ8-CHIP-R2 | TGTAAACATCTGAGCCGAAAGTAACG | CHIP | ------ |

# Table S6 Promoter sequences of some JAZ genes

## 1. Promoter sequences

>JAZ1-P+

GTCGTAAATTAATGGTAAACTCAACTAATGCATGAATAGTTGTAAAAGTATATATATTTGGACACGTTACTCAACTTGCATCGCGGATCTGTATTCCTT

CCCAACCACTACGTACACGCATTTCCCACGAGTTTGACGATCTTTCTCGTTTAATCTAATCATATCTACTTTTATAGGCTCCATAGCCCACATTTCATA

TCTTATAGTACGTTCCAACTTCTAACCAATAAACTCAAAACTAGTTGAGTTTTTTAATTAGTGAAATATATCTTGAATTTTATATATCTAATCTTGCAC

CAATAAAGTTTCTGTCAATTATGTAAAAGAAAGACTCGTTGTATCAGTATGGGACGACTTTTTCTTCTGAATTGTGTCGATCGAGTCCATATTATAATA

CTTTGTCACGTACTTTGTTTACTGATTAATTTAATATAATTGTGTCGCATTATCCAAGTCTAATTAAACATTAATGTGTATATTATACACCAAGTTACA

ATTACAGATCTATCTCTTAGTCGTTTTATTATGTATAGCCTACAATGAAACATGACATCAACGTATAGGTGAAGCTGAAGCCTATACATAAAAGTAAGG

TTTTGGAGTTTGTTTCATGTTTTAAGGTTTTAGATATTTTGTGATCGATTTGCATGTATAGGGTTCCAAATATCTGAGCTTTGGCTTGGTCTACTTTGA

GAAAGCAGCTGTAGAGATCAACTTTAGCCGTTTAGGTGATAAAATCACATTAGAACTCTATAGGTCGCGTAGCAGCGTATAAGAGCCCTGGATACTGAA

ATACAAGAATCTCTCCACGCGTAGAAAAACGAACCAACCATGATAAAACACGTGTCATATCGAATAGGTACAGACAACGTGCACTTGCCAACCTTCTTT

CCTTGTAGTTTCCTTCTTCTTTGCTTCTTTTTTTCGTTAAATATTGATATTGTTCAAAAAACGCATAACAACAAAAACGTGGTCGACGGAACATGTGGG

TGGTGGAGTTGTCTTCTTTAGGGGACCACTCACTAACGCCGTTTACTTTCCCGCTTGGACGATAACACCCTTGATATATGGATTAGTCTCTCTTTTGCT

TTTGGATATCTTAATATATTTTAATATGTCCATTTTTATATTGACGACCATTTAATTAGGTCTCAGTTTGTATTATTGATTTTTAGGTGCCAAGCATTA

CTTTCGTTACGTGTAATCTTTTGATTATTCAATCAAATTATCATATGGTTCATGGCATTTTATCAATTGTGACGGTCGACATGTGGGTTGACTTTGATG

TATGACTTTTAGCTTGTGCCTCATTTTTATTCCAACTCGACAACTAGCAGACTTTATATTTTAATTTATTTCTTTCTAGATATTAGACAATTCTTAGCT

TGTCTCTCTGCATGAATCACGCGTTAATATACTTATAATAGTAAGTTTTAAAATGTTTCGAGTATAATATCATATTAGATTTCAATTATAATTGTAAGA

TTATTTAGTCTCAATGAAGAGCATTTAAAAATGTGAAAGAGTTTAGGCTTTGACTGTTGACTGGTTCTAACGCGTTTTGCTTCTTATTTATACAAAAAG

ATTTTATTTTATTTTAATTAGAAATATTAAGTACTTTTTAGACGTGTTTAGGTTGATTAATTTCTTGTTAACATATCCGATAAAAAAAAACAGCACCAA

AATCACGACGAGAATTATGCCTACGTTACGTTGATAATAAAGATTAGAGATAAAATGTAACCTCTCTTCTCTTTACATCATTACATGCATCTTGTGTTT

TATTTGACTTTATTCTTACCAACAAACAACCAATGGATAAATTAGGCTCGTGAAGGAGGGCAAACTCGTAATTTCAAAGAAACGGAAAGCGTCAGAACG

TGGAGAACAGTGAACCGAGACACATTCCCGATTTTTCTAAAGAAACAAAATAAAACACGTGTTTTTTATACCATAAGTAAAGTAGTAGTATTTATTAAA

CTATTAATATATATAGCTTTGTT

>JAZ2-P+

ACTCTTTTGACCTTTCTATATTTATTACTTTGGCTGAGATGGATCATTGGCAATAATGAATTGGAAGTTTGTGCAAGTTCCCTTTGAAGTATTGTCGGT

CCAGAAATGCTCTAAAATGATTTGGGATGGACTAAGAATTTGTTATGAAGCTGAGTGTTTAAATTATGTGCAGTGTTTTACAAGAAGTTTCCCATGTTA

TGCTAGTGTTACGTCGAGGGAATCAACAGAGTTTGTTCAACTACCAAGAGCTAAGCTAAGTTTCTTAGGATCAAGATCTGATGAGCCAAAGACTTGGAC

AGGAGCTAAAACGTGCTAGAGATATCAGAGTTTGGATTCGCCATTAAAATTCTGTTTCTTGTGATACCTTCTTATTGGAAATCTTTGTAGTCTTTACAT

TTCTATTGTTTAACATGAAATCTCAAAAAATGCCAAATCAATTCTCAATTTTAAATTTAGTAGCTCTTGACAGTTTTCCCTTTGTTAATTACATATATA

GAGAGTAACTAAATCATCTTTTGAAAGGTAAAAACTAAAAAAATATATTCCAAGGGCCCAATGTTTTACCACTTTCATATGTAAGCTTATTTTCTGTTA

AATATGTGAAAGAAAGAAGAGGAATGAATCAGCTGTGAAAACCAAGAGCAAGTATAAAAACATGGCTAAAAGGGTATCAATCAGCCTTGTATAAGAGAG

CCATAAGTAGATAAAGTTTCTACAGTAAAAACAGGAATATAGAGTACTATTCCTTAATTAGCTTATGTTCTTCTATTCTTCTTGTGACCTCTATCTTGC

ATTTTTGTGACCACCCCACTTGCCTTCTTCTTTTTGGTTGCATACTCTAAATAATATCCAGACTTTGATGTCTTTATGCGCTCTGGGTTCTTCTTGTGA

CCCCTCTTCCTTGTAATTGTATGTACCTGCAGCTTCTTCTGATTTTGTTTCTTATGGATATGTTTGTGATGTATAAAATTTTGTGAAGATGTTTATAAA

ACAGTTAAAAACCAAATTAACCAATCATCCTTATTTTCTCACGAGCCACTCTATGAACCTTACGTTATCTCGATGTACCTGTAATGAGTTAGCAATTAC

TACAATCCAAGTCAATAAAATAACAAGTTGGACGTTACGTTATAAAAGAAAATCAAACTTATTTGTGTGTTCCTAACAAACGCATGGGATAATAACAAG

AGAATAAAAGACAGAGTGATAGGATCAACGGCTATTGTGCGTAGGAGAACACAGCTGCTATATAAGCCGCGTAACAGGTGATTACTCTGACTACGACAC

CAACAAGAAAAATTCTCTCAAATGTGGGCAGTTAAGTTAGACACGTGTAAGGTCGCGATTCATGGAGGAGACGTGCTCTTCCCGCTTTTTGTAATTATT

TATTACTGTTGTCATAAATAAAACACTAAAAAAAAATTAATAAAAGGGAAAATACAAAACGTGATTTATGGATCAAATTTGCATTGGTGGTGGAGTTTT

GGTTATGTAAGACCCACACCGTGTTCCCTCTATCTAACGCCGTTAATCCTCTTTTTTCCCCCAAATCATACCGTTAATTCTCTTTGAGTGCTAAAATAT

CACGCCGGTCTATGAGATTAAAATAGTAGACTAATTAATAGTGCTAAAAATACATAAATTAATGCCAAGAGATTCATCTTAAAAAAAAGGATATATGAA

CCAACTAAATGGTATAACTGAAAGACTATCATAATCAAATTTCATCTGAACATTGATTTGTTTGAACTAATTAATTTTAGGATTTTACGTTTTCATAAT

TTTTTATTTTATTTGAACATCTTGTAAGGGTAAAATGGTAAAAACAGGAGTAGTAAGAGGGTGAAATGGTTATTTCAAGTGTGAAGCGTGGGGACGTGG

GTGACGGTGGTGATCTGAAACAGTGGTCTGCCGTTAAAACATTAAATTAAAACACGTGTTTTAAATGTCTCTATGTTAATTAATACTATAAGAGTATAT

TTTACTAATTTCCGTATCTGC

>JAZ5-P+

GCGTGGTCTCCGCCAAGGGTGGGTTGGTTTAAGCTCAATACTGATGGTGCTTCGCGTGGTAACCCGAGACTAGCTACAGCAGGTGGAGTAGTTCGAGAC

GGGGATGGAAATTGGTGTTATGGGTTTTCGTTGAATATTGGAATTTGTTCGGCTCCGCTTGCGGAACTATGGGGAGCATATTACGGTTTAAATATCGCT

TGGGAGCGCGGTGTCACACAGTTGGAGATGGAGATTGATTCGGAGATGGTAGTGGGTTTTCTTCGGACAGGGATTGATGATTCGCATCCGCTGTCCTTC

CTGGTGCGGTTGTGCCATGGCTTACTTTCAAAGGACTGGTCAGTCCGGATTTCGCATGTGTATAGAGAAGCTAATCGTCTCGCGGATGGGTTAGCTAAC

TATGCTTTTTTTTTACCGTTAGGTTTTCATTTGTTTAATTCTACTCCGGATATTGTTATGTCGATTGTTCACGATGATGTAGCGGGGTCTGCGTACCCC

CGGAACGTTCAAGTGTAATTTTTTTAGTTTTTTCAGTTTTAATAAAAATGGGGGTTCGCCCCCCTCTTCTACAAAAAAAAATATCACTTTCAAAAGGAC

CCATTCGCTACCCAATTTTTCTAAACCCCAGAACGTTTTTTTTTTTTCCAAAACACAACAGCCACTTGGTCTTTGCAACAATGCAAGTAGTGTAGTGGC

GTAGCTAGGGGTAACGAGTTACCAAACCGGTTTGACAATCTGGTTCGATCAATTGGCCGATCACTCGGTTTGCTCTAATCTTGCTTCATTTAGTCCCAT

CAACAACACTTATTAAACTAAGTCACATCTAAACTTTTTATATCTTTGGTATCATTTTCCCCGAACACGTTTTTTTGAACGAGTCGTAGTTTTGAAGCA

ACCAACGAACGTATTGATTATTATGGGCATCCGTGAAAGGGGCAGTTCGGTCAATTGATCCATCAATTTTATTTTTGACCAAACTGATTAGGTGATCTT

AAGATTTTTCCTTTTTTCTTTCTATAGGAAAGTGGGAAAAACGCGATTCACACGATACTGTTGCTAGTGCGTCGTACAGATTGCGATACGTGTCACAAT

CTTGACGGTCTGTTCTATTTAAACCACGTGTTTTAATCAAAAAACTTCTGTCGGATCGTATCGGAGACTTTGAATTTTTATGGCACATGAAACTTCGTA

GGGGTTTGATGGTAGGATCCCACATTGGTCCACTTACTATTCAATTCGCACCGCCGTTAAGTTTGTTAACTAACGTGAGGAACGTCGCGGTGATGTAAA

AAAAGGCCATGAAAGTTGTTTCTATTACGATGATGCCATCACTGGAGTTGGTAATGTGCTCTTGTGGAAGGGCAAATTTTGTAAATACGATCAAATTTC

GAGTCTTTTACGAATTTGTGACATACGCCGTTTAATAACGTTTGCCGTCGGTTCTGAAATCTTTATTCTTTTTCTCTTTTATCGTTACGCGTAAGGAAA

ACGTGCGGTAAATTCTAACCGAAAATATATATAAAAAAGTAGTCCAATTTTGAAATCTGGCCGTTGATGGTAGACAACTAAACTGTGCCAAACAAGAAA

CAAGAGGTGAACATATTTTTATGATAAGACAGGGCAATAAATTTGTTGTACCAACTCATAAAACAATAGATAAATTACTTGCACAATGATGGATTATTA

ACATTAATTTTCTTACTATTAATCATGGATGAAAATTCCTTTCTTCACACTAGATATAGTTCTTTAACTAGTTAAAAATGCATGCGATGGAATATTACT

AAATATGATATAATCTCATGGCTTTATGTAAGATTTGTTTTTTGGTTTTTTTGGTTGTTGTTAATAAATTTATTATTGAGAAGTTTAATTCTATTTTGG

TCACAATATATTGAAATATTTTTAAGAAACTAAAAAGTTCCTATTTATTTTTGTTTTCATTAATTTATGAGAGGCTATTAAAGTCACAGAAACTTATTG

GGTGAATGAGTTTATAAACAC

>JAZ6-P+

AAAGAACCAATTTGTTTTAGTCATTGTTGTAAAACGGACCTACATTCGTAGTCGTTACCCCTAAATTTTAAACAAATTTTATGTTTAGTATTGTCATAC

GTCAATGCAAATGAGTTGTATAATCTTTCAATATTGTCTTCTAATTTTATTTTATTTTCTGATAAGTACCATTTTATTTATGAAGATAACGACTGTACA

GAGAATTCCCACCACAAGCTCAAGCTGCAAGACCAAACTTACCCGAAGACAACCTGTTACAGAGACACGCTAGCTCGTAACACTAGATAGAAAGGAAAT

CTTACCCAGCTCAAAGATTAAGTCTGAAGCACACCTAACCCAAAACTGTTGACACCATCTTGATTATGTCCATATATGAACTTAGGTCTCAAAACAACA

ACTATTTATTTGATAAATGTTGATATACTCTATTGCTTAAAGGAGAGTCCAACTTCGAGTGGTGACCGGAAGGTTTCGCCGTTGCCTTTTCTTTTTTGC

CGTTAACGTTGGTTTGGTAGTCTTAGTAGAATCTTCTCACAATCTTGATTTTTGCATATCAAATCTGTTAGAATATGACATTCCCTTATTTTGGACATG

ATCAAATATGTGATCGAAACCCAACTTGCGCTTCCAACAATGTAGATAATCTTCATGTGAAATAGCGAATATTATTTGGTCTTTTGTATGTATTAGAAA

ATGACAATGGATCTATATTGTGTAATTAAATACACACATACATGTGTATATATTAGACTCAAGTTGTTGTTCAACTCTTGAAATCATATGATATTGAAG

AGTCTTCAAAGTTCAAAGTATTATGTTCTCATTTTGCAAGTTGCGATAAATAATTCAATAAAAATGATTTTTTCCCTTGGCGCGATCGTCTACGACATT

TTGTTTTAAATTCTTTTTTGGTACTCTTTGGCATTGCCTCTTATTCAAATAAAATTCAACGCTCGACTTCATTGAACAAAACGATTAAAAGATTTTATT

ATTATTATTGTTATAGAAATAATATTTAACTGAGCAAGTAACAAAATTCATATTAGGACTAAACAGAAATCGTTAACCTTTTACCAACATACAAAACCA

CAAATTCTAATATTAATTACACTCAGTTTTTGTAATTAAAAACAGAAAAGAAAATTAGGCCTATGAAGTATGAACGCTATAATATCCTTTAGCAAAGAG

GGAAAAAAAACGCAAATCATGCATGAAATAAGGTTGGTACCGCCAAGATTCCGACACGTGTTGACATCCTAAACGCACATAACAATACTTCACGTGTCC

TTAAACTTTGTCGGGGATAAGTAATTGACACCATTCATCGATAAATATCCACTCACAAGTCGTACTGTTGCTAATGGATCCCACATATTAAATTCGTAT

TAGCCATCGCAGCCGACGCGAGACTTAACTCCGTCAGAACTCCGTTAGACTATAGCGTTTGCAAATGCCCTCATTTACGCCCTTTAATAACGAATATCC

TAACCACCACTAAACGAATTACCTCAGAAGGGTAAATTAGTATTTAACAGAAACTCTTGGGACCCGAATGAATTAAATGGGTTAACGCCTTTATGTAAT

GTCGCCGTCAACTTACAACAGTTTCTTCACGGCTTGTGCAATGAAAACGTGCCGGAACTTGTAACGAACATTGTAGTTTGATTTTTGTAATCAACCATA

GGAATAAATCATTTTTTCCGCATATAATTTATTTCCCTATTTCTATTCGTGACAAAACATTTGAGGATATAGGAAATTAATAGCCTATAATATGTTTGA

CCATAAAAAGAATTTCTTCTTCTTGAACCATCATAAGAAAAATGTGTGTTTAGTCTATTGATCAGTTTTGTGTTCAAAAAAAAAAAAAAAATGTCTATC

GATCAGTTAGGTGTAAAAAAAAAAAGTTACAAAACTCGTGACAAAAACATTCTATATTGGACACACATCACTGTCACTTCAGACTAAATAAAAAAAAAAGA

ACACGTTATTTCGTTTTCTTT

>JAZ8-P+

TCTTTCACTTAATTATTATTATTATTATTATTATTTTTCAGTCTTTAAGAAATTATTTTCAGTCTTTTCTTTTCAATTGAGTTCATTTTTATCTAGCTA

AATTCCATATTTGGTAATTTTGTATTCCAAATGTATTCTAAAATTTTCACATCTTTTCTTCTTCTTTCAGGGATAATTTTTGCTAAGGATTTTAGTTTC

ATTAAATATAATTTTTAAAAATGTTTTATCATAAAAAAAAAAGTTTAATACAGTGTTTAGTTCACATATATAGGTTTTGGTTTATTATCTAATCCCACG
TATTTTTAGATTATTTTCTACTAAATAACTTATATTCTCATAAAGAAAATTATAGATGGTTGTCAGTTTTCATTTCTGTTTAAATATTATTGGTTTTAT
TTTAATGTTTACCACATATATCGACATTGTTCTTTGCTATAAACATGGAGTAAAGAAACTTTTTAAAATTTTCAAAATATGATAACAACTATATAATAA
AAAAATTATTACTATATTAATTGTAATATTACTAAATAATTTTTATGATGAAAATTCATGTATTACCAGAAATAACATCTCATTCTAGATTTAAGTTTT
GAGTAAAAGTATATATATTTTATTACCAGTAATATGATATTTATTTTATATTTTCTTGCAAAATACATCATATTTTTCTTTAATCTATTTTTTATTGTA
ATAAATAAATATTCACACAATATATTATTATATTATTTCATTTTTATTAAAAAATTATTATTTTTCGTTGTGCTTTTTAAATTTCCATAATGAGTTATA
TACATTATGCGTAAATTTTATGTTTGAAAGATATAATAAATATTTACTATAAATGTTTTTAATGTAAATATCAAAACATCAAATTTTATTATAATATAT
ATAGTTATATAATATTATATACATTACTATATTAATATAACTCACGCAACGCGTGAGATATTTTCTAGTATATATATATACTGTTGTTGTTCATTAATC
AAGTAAGATCATCAATCCCGTTTAACTCACAATGTGTTTGTGTGTGTGTTTTCACGAATCTTTACCACTTACATATTTCATAGTCCTGAAAGAATAAAA
TAAATTATAATATAATAGAAAAATTAAACAAGGAGATATGGAAAAGCGTAATGCGAATGGTCAGACAAAATTAGACGTGCCTTAATGAAAGTTATTAAT
ATTGACTCAAATGAGTTAGGTGCACGAAATGACATACACTAAAACGCTTGGAAGCCTATTTTTATACTAAATGAATTTGAAGAAGAAATACAAATTGTA
ACTAACTTGGAACGTTACTTTCGGCTCAGATGTTTACATTTAACTAGTAATAAGGTATTACACACGTTTCTTAATTAATTATCTTAATTCGAATCAAAA
AATGAATTTAAAGTTGTGTAAAACACTTTTAACAACAACAACAAAATATCTCAAAAATACCGCCAATATTTTATTCAGTGCGTGAGCAATGGATCACAT
ATACTAATTGATACGCCGCCTGAAATATATCAGAAAATATACAATGCATTACGGTGGCGGATACCTCCGCAGCCCACAACTTTGACAAATAAAAAATAG
ACTTCACACTATACCGATTAGTGAGTGATTAAATACTACACATATAATATAAAGTATTTAACAAATAAATAAATCATTCAGAAGGACTGAAATATATAT
ATTAATTTATTTATTAAAAAAAAAAGGGAAAATGTGCGAAATGCCTCCAAAACCGCCGTAGAAATTGAGGATAAGTATTAAACTAAAACAAAACTGGGT
CAAAAAACAAATCCAAAAGGACCAAAACCTTAAAGTTGCACAAATGTAAGTGTGGAAAAAAACATTTTCCACGTGAGAGAATTGAAGCAAGTTGTGTTC
TGTCTCTCTGTCCTAAGAGTCCGCCGTTGTAAAACTCGTGTTTTGTTTCCACCGTCTCTCCCGTGTCCTCTCTCTCCTCACAAATAGATATAAAGATCA
ACATAATCTCAATCCATATAC

>JAZ9-P+

TTACTTGTCGAAATGTTACGCGCGTTTCAGTGTTGGTGGTAGTCACGCGCGTCAGACCCCAGGCCCAAACTTTGGTCATGGTCAGTCTTCCTCTAAACT
TATTTTAACTAAACTAGATAAAAAAATAAAAAGTCGTAATCACTAAAAAAAAAACCTTTTTACGTAACCTCAGATGAAAATTTAAATATTAGACTGATC
TTAACAATTAGTCTAATGGTTTATATATTTGTGTACGACGCTAAATTTTTAAACCCATAAAATTGATAGTGTTGACCATAAATAAAAATATGAACATTA
TACTTGATGGAGAAAACAAATTTATTAAAAACTTTAGAGGTGGATGGATAGTTTTAAAATTATATATATATATATTTGGCAGAAGGAGAGAAGGGCAAT
AAGGATGATAATGGAGTGGGAAAAACATTGGCGATAATAATAGGAATCGTCACCTTAATCATCTTGCTCGTTGTGTTTCTCGCTTTTGTTGGAAAGTGT
TGTAGAAAATTACAAGGTATTTTTGATATTGATTTACGTTTTTTTTTCAGTTCTTTTATCGATCATAAAATCTAAATTACTATGTATATGCCTTTTGCA
GATGAGAAATGGTGTAAATGAAAGGACAAAGTGGGAAAGATATCAATCAACAACTCGAGTCGAGCCACACTGTTAGTTGTATTTTTGTAATTTTTTTG
TTCTCCCTCATAAGATGTACGTAGTTATAGTATATTGATAATTTGATATAGTATTGATATGATTGTATCTCCGAATCGATTTTTGTATAATTGTCGGAC
TTATATAGGCCAAAGCGATATCTTCTTCTTTGATGTGAATGAAACCTTCGATTTCTTATCTTCTATAAATGCCATGTTTTTCGTCTATGCAAAAAATAA
CAGTAATTAAAAGGGTTTGAGCTTCTTTTATCAATACACAAACAAGTTAAAATAAATGAACGTGTCGAGAATAATGGAACATATTAAACCGTAAATCAG
GATGGAGATCCAAAAAATGATAACTGTGTTCGTCCTATTGCGAATGTCTGAACGAATCTTTTCATCGTATTCATCATTATTTATTCTCATATTGTCTAA
AGAACTCAATTGAAGACTTGGCCTAATGTCCTTTAATCATTAAAAATGATAATTTAAAATTGTAAGCTGACTACCAAATCGTTTTAGGACTTTGAAAGA
CCAACTAACGTCTAATTCTTTTCCATATTTTTTTTAATTTTCTGAAATTCTTAATGTACTACCGATGATTGCTTCATAACCAGGTTCTTCTTTTAATTT
GTACATGAGTAACATGACTAATAAATGACTTGCGTCTGTTTGAAACATTATATGAATAAGAAAAATTGGTCTCAAATAACAACATGATGTATCAAAGAT
TCAAAAATTTCACGTGTTATGAAATGAGCTTAGCAAAAGTTCACTCAAGTTCTTGGAGGTCTAGTTTGGAATGCAATTTAGCCAAATCTGTACAGTTAT
ACTGAACAGATTTAATGTAAGTCGGGCCGTAAACAAAATTATGGGCCTTAGATAAGCAAATCAAACTTCTTTCAGCCCATTGAGCATTCTCGTGATTTG
GTTATTGTATATCCAATACGTGACATTACTTTTGCCTCAACGAAAGAGTCTTGGCTTATCATTTCATTTATTATTATAGTTGAAATAATTTTCAAAGAA
AAGAAAAAAATATGTGATAAATACCAAACCGTTGCATAAGAAAAGCAACCACACATTTTACATGTGTGTGTGAGTTCACGAAGAAAATATCCAATAGCC
AAACTAATTAATCCACCTTTGAAAAAAAACATGCACTATTATAAACTAGAGCCACCGACGGTCGACGGATGTCTAATTGTATATGGTCCTCAGAAATGA
AAAAATCAAAAGAATAAACACGTGTGAATTAACCGGTTTAACCAGTAGCACCAACCGTTTGTACCGGTCAGAAAAAAGCCCCACATGCTTTAATCAAGC
AAGCCTTTAAAAACAGGAAAG

>JAZ10-P+

TATTGGAAATGAACGACAACAAGAACAACCGTTTCATTTACAGATTTACTTCTTCAAATGGGCTTTTCTTCTCTAAATGGGTTTACTACCTTAAGGGGC
TTTTCAACTATAGGTTCAATTATATTCTTATGTAAGGGTACTAACTAGTGTGGGCTGTAAACGACTTAAAGTTTCGGCCATTTAGTTTTATGCAAGAAA
AAAAATATAGATAATACAGTAATGATGAATCCACTATACATGTTAAATCCAATATAATAAATCCACTATACATTAGTGTTCTCATATTTCGTTTCAAGA
TTCGATTCAAAAAAATTTGAAAATGTTAAATATGTGTTTCTGAGATTAAAGGCCAAAAGAAGGGTAAGGTCGTATATGAAAGGTGGACAGAGGGGCAAAT
TAGTCAGCTTGAAAAATACATCGACATTAAAAATCGGCAGCACTGACAAAACGTGTGATAACACAAATAAAAATAATCGGAAGAAAAAGAATAAATAAG

GCGTATTGACTCTGGAACGTGAGAAGATGAAAGAGAGCGACGAAAACCTCTAAAGACTTATCGAGAAGTGCACCTTAGCCTAGTGAATAATGATATGTA

TTGACATTAACTACTACTACTACAGCTCCCAACACGTGAAAGACATGCATCTAACTCTTTCACTTTCGGTTGCCAAGAAAAAAAAAAAAAAAAAACTTT

CACTTTCGGCATCAAATATATACTACTAAAAAAGTTTTTTATACGTAATTCTTTTTTAATATTTGATTAGTTCAGAATATTAATTAAAAAAATTACTTT

TCGTAATCACTACATTTTGTAGTTATATGATTTTTAATTTAAAATAGTAAAAAGGAGAAATGTAACTAATTAAGCTAACATGCGCGCGATAGAATAAGG

TGAAGTTTAAATTTGAGCACAAGATAGAAAATTGAGGTCAATTTAATTCGGAAGTGGTTCAAGGTATATTTTCACAAGAAAGAAAAACGTGAGTTCATT

GAATCCCTCTATAACTTTACTTGAGAACAATGTAATAATATGTAAATCTGTTAGGAAACGAGCAGCCACATATGCTTTAAACAAGAGAACAAGAAAAAG

AGCGGCCACATGTGAAAAAAAAACACCAGCCAAAATGTGAAACTAAGAAAAGAAAAGAAAAACCATCGACATCGTCCAAAGGATCGACCCGTCTTTTCT

GGTTTGGCAGAAAACAAACTTCCAAGTTCCGACTTGACCTAGTCTTTATGCATCTGCAGTGGTGTTTTCTTTAAATCTTTTTTTGAATGGATTTGAAAA

TCTTATTTTTTCTTCTTCTTTCAAAACACTAACTGTGAATCACCACCAACATTTTATTGTTCTAAAATCCCTATTTGTATGTTTTATTGGTTACTTTCC

CAAATATGATGAAAGTTTATTGGTGTACTTTAGTTTAGAAATGTAAGAAATTTCAGTTGTATTATTAGGTCAACCAAATATTATTCTTGAATAGTAATG

TCAAATTTTGTTTATATTGAAAAATCAATAAGACTGATATCTACTAAATGTGATTTGCTATACTAGTAGAGCTAGTAGTAGCAAGATTAAAATTCATAT

TAAGAAAAAATGGAAAAGAAAAACATGTTTCGAGAAAAATATACAGATCAGAGACTAGAGTAGAAAACAAATATAATGTCAGAAGAACCCAATGTGACG

AACAGTAACAGAAACATGTTACCAAATTTTTGAAGATATATTTTTATAAAACATTACAATGTTCATTGGACTTCCGTTATTTCCGGATATAAAATATCT

TCTTTCTGCGTAGCAAGCAATTACATTACATTAGGTATATTAAGAATTTAAACATGTGTTTTGAATCATTAAATTAATTAGATAAGTAAGAGAAGAGGT

TGGGAGCCTAATAAAAACAAAACAAAAAGGAAAAAGTGGATAGGTTGCCAATTGTGCCGAAAAGAAAGAGAAAGCATGAACCTCGTGCGTTAAATAAGT

ATCAGAGACATACTACATTTT

>JAZ11-P+

TATCTCAAGTACTAACAACAGCTCTATGTTTCTCAAAAGGAAAGTCTCAAAGCGACGACGTATTATTTTCACGCACGTTTTAAATTCTTGATGCCTTTT

TTTGTTTTATTCTTTTTTCTTAAGTTACACATTTAAACAAACAAAAAAGAACAAAATGAGAAAGAAATAGATTGTTTTCGAGCTTTCTCTCTCTTCTCT

CTCTAAAAAATCTTAGAGAAAAGGGAAAGTTTTCTCTTTTGTTTCGCTGGCCTGATGGCATTCCGACGTTTATGTGTTTTAATATAGTTGGGTTTTATA

TCTAACATCTCTGTCGTTTTTAGCAGTGATGGCTGGCTCTTGCCTGACAGCGTTCGAATTCCATGACCATCGTTGGCCGGATCTACTTTTCTTATCAGT

GTATCTGATGTTTTTTTTTTTTTTTTTCTTTCAATAATCTCATAGATCCTCTATTGGAAATTTAGGTGTTTTTTTGCTTAAAGGTGTTTATTATCCAAT

TTGATCTTTTGAGTTTGCGATTTGCAGGAATCAATCCAAAGAGTTACATCTTAAGTTGCAGAAAAGAAAACAATTTCATTGCACACTAATATTGCTTTT

AGCTGTAAATCTCTATTTTGATGTTCGATTGGTTTATGAACAATGAAACCTAGAAGAATCAAGCTTGCTTTTATAATCAATGATGATTTTTCTCACTGG

AATCGATCTCAGATGGCGAATGATCTACAATTGAGGCTATTTGTCTAATTGATCTATGGACACGAGTGTTTTCCTTTTTGTTTTTAGGTGTTGCTGAAA

AGCTCCATCGGCTTAATATCTACGTTGATGTTCTTTGGAGTTCGGGAGTCATTGCCCTTGTGAAGATCTTTTCTTTACAGATTTGATGATGTAGTTGGT

AGGATTCTCTAGTTAAAAACCTGTGGACTTATTTCTTGAAGCTCTGACGGTTTTCGGTAGTTTTGGAGTTCCCTTACGCTTCGTACCATATCGGCTATG

AGCGGCGAACGGCGACAACATCTACTTCAACTTTACTTCATAGGCTCACACATGGTTTTGTGTTGGATTTGTTATATTTCTTAGTTCCTTAAAGGGGTA

TCTTTATGGATTGGACTTTTTAAGTTGGGTCTCCGCTTATAAGCTGAATTGTTGGTGAAGTGGAAGCTTCCACTGTTGTACCTTTTACGACATAGAGTT

TTAAATAGAAGAGAAAATTCCCAAAAAAAACTTCAACTATTAAAATTTGCCAAAAAAACCTCAACTTTTTCGGTTGTCAAAAAACTCAAACTAATTTAT

TAGTGACAAATAAAACCCAAACTAGAAATATTGGCAGATTATACATACGCTCTAAAAACAAATTATAGTTTTATCCTTAAAAAATTAATTCTTTTTTTT

TTGAAACGAAAAGAAATTTCTTATTTACTTTGTATTTTTTTAGTTTATACGTTTTTGCCTTTTTGGAAATTATCATAGAAAAATGATATGAGTGTCTCA

TTTTTAATTCTCTTTTCATATATTTTTACTTTTTAGATTTTCAAACTTTAAAAACCAAATCGTTTCTCTATTTCTTTTTTTTTTGCTTATTTCTTTCTA

ATTTTTGGTTCCTCAATATCAAAATGATTCAATGTTCTTCGACTAATTTACATATTTTGATTTTTTCATCATAATTATATAGTTAGCTTTGTGTTAATA

TATCTTATTTAATTTATTTTAGCAATACGATAACGGCGGTATGCATGTACTAAAAAGGATGCGTAAAGAGTATTAAGTTTTTCACATCGAACAAAATTG

TCCACTATTTTATTTTTTTAAATAGTTGTCTATATTACAAGAACAAATGAAAATAAATAGGTATTATTAGATAAGTAGTTTTTTTTTTTGGTAAGGCAT

TATTACTTATTAGATAAGTAGTTAGATATATAAAATAATCAAATAGTTGAGGTTTTTTTGGAATATTTCTGAGAAAAACAAAAAGAAAACAAAATTGAT

TGAATAATGAATACACCGAAT

---

P+:Forward sequence

The 2002bp upstream of TSS(transcription start site) was used to search E-box

## 2.Promoter analysis of some JAZs genes

| promoter | position | elements |
|---|---|---|
| JAZ1-P+ | -1304 | CAGCTG |
| JAZ1-P+ | -1162 | CACGTG |
| JAZ1-P+ | -1020 | CATGTG |
| JAZ1-P+ | -773 | CATATG |
| JAZ1-P+ | -735 | CATGTG |
| JAZ1-P+ | -66 | CACGTG |
| JAZ2-P+ | -1432 | CATATG |
| JAZ2-P+ | -1379 | CAGCTG |
| JAZ2-P+ | -763 | CAGCTG |
| JAZ2-P+ | -740 | CAGGTG |
| JAZ2-P+ | -697 | CAAATG |
| JAZ2-P+ | -674 | CACGTG |
| JAZ2-P+ | -277 | CATCTG |
| JAZ2-P+ | -145 | CAAGTG |
| JAZ2-P+ | -69 | CACGTG |
| JAZ5-P+ | -1923 | CAGGTG |
| JAZ5-P+ | -1786 | CAGTTG |
| JAZ5-P+ | -1651 | CATGTG |
| JAZ5-P+ | -1498 | CAAGTG |
| JAZ5-P+ | -1407 | CATTCG |
| JAZ5-P+ | -1248 | CACTCG |
| JAZ5-P+ | -889 | CACGTG |
| JAZ5-P+ | -830 | CACATG |
| JAZ6-P+ | -1960 | CATTCG |
| JAZ6-P+ | -1896 | CAAATG |
| JAZ6-P+ | -1355 | CATGTG |
| JAZ6-P+ | -1269 | CATGTG |
| JAZ6-P+ | -1225 | CATATG |
| JAZ6-P+ | -760 | CACGTG |
| JAZ6-P+ | -724 | CACGTG |
| JAZ6-P+ | -555 | CAAATG |
| JAZ8-P+ | -1876 | CAAATG |
| JAZ8-P+ | -807 | CAAATG |
| JAZ8-P+ | -689 | CAGATG |
| JAZ8-P+ | -180 | CAAATG |
| JAZ8-P+ | -151 | CACGTG |
| JAZ9-P+ | -1833 | CAGATG |
| JAZ9-P+ | -1410 | CAGATG |
| JAZ9-P+ | -606 | CACGTG |
| JAZ9-P+ | -259 | CATGTG |
| JAZ9-P+ | -103 | CACGTG |
| JAZ9-P+ | -40 | CACATG |
| JAZ10-P+ | -1948 | CAAATG |
| JAZ10-P+ | -1376 | CACGTG |

| | | |
|---|---|---|
| JAZ10-P+ | -944 | CATATG |
| JAZ10-P+ | -907 | CACATG |
| JAZ10-P+ | -905 | CATGTG |
| JAZ10-P+ | -764 | CATCTG |
| JAZ10-P+ | -563 | CAGTTG |
| JAZ10-P+ | -168 | CATGTG |
| JAZ11-P+ | -1300 | CAGATG |
| JAZ11-P+ | -964 | CACATG |
| JAZ11-P+ | -177 | CAAATG |
